# Independent prognostic implications of *RRM2* in lung adenocarcinoma

**DOI:** 10.7150/jca.47895

**Published:** 2020-10-17

**Authors:** Chao Ma, Huan Luo, Jing Cao, Chengshan Gao, Xianen Fa, Guangsuo Wang

**Affiliations:** 1 Department of Cardiothoracic Surgery, Second Affiliated Hospital of Zhengzhou University, Zhengzhou, China.; 2 Charité - Universitätsmedizin Berlin, corporate member of Freie Universität Berlin, Humboldt-Universität zu Berlin, and the Berlin Institute of Health.; 3 Charité - Universitätsmedizin Berlin, BCRT - Berlin Institute of Health Center for Regenerative Therapies, Berlin, Germany.; 4 Klinik für Augenheilkunde, Charité - Universitätsmedizin Berlin, Corporate Member of Freie Universität Berlin, Humboldt-Universität zu Berlin, and Berlin Institute of Health, Berlin, Germany.; 5 Department of Human Anatomy, School of Basic Medicine, Zhengzhou University, Zhengzhou, China.; 6 Department of Thoracic Surgery, the First Affiliated Hospital of Southern University of Sciences and Technology, Shenzhen People's Hospital, Shenzhen, China.

**Keywords:** *RRM2*, lung cancer, lung adenocarcinoma, prognosis, immune infiltrates, TIL

## Abstract

**Background:** Ribonucleoside-diphosphate reductase subunit M2 (*RRM2*) is the catalytic subunit of ribonucleotide reductase and modulates the enzymatic activity, which is essential for DNA replication and repair. However, the role of *RRM2* in lung adenocarcinoma (LUAD) remains unclear.

**Methods:** In this study, we explored the expression pattern and prognostic value of *RRM2* in LUAD across TCGA, GEO, Oncomine, UALCAN, PrognoScan, and Kaplan-Meier Plotter, and confirmed its independent prognostic value via Cox analyses. LinkedOmics and GEPIA2 were applied to investigate co-expression and functional networks associated with *RRM2*. Besides, we used TIMER to assess the correlation between *RRM2* and the main six types of tumor-infiltrating immune cells. Lastly, the correlations between immune signatures of immunomodulators, chemokines, and 28 tumor-infiltrating lymphocytes (TILs) and *RRM2* were examined by tumor purity-corrected partial Spearman's rank correlation coefficient through TIMER portal.

**Results:** *RRM2* was found upregulated in tumor tissues in TCGA-LUAD, and validated in multiple independent cohorts. Moreover, whether in TCGA or other cohorts, high *RRM2* expression was found to be associated with poor survival. Cox analyses showed that high *RRM2* expression was an independent risk factor for overall survival, disease-specific survival, and progression-free survival of LUAD. Functional network analysis suggested that *RRM2* regulates RNA transport, oocyte meiosis, spliceosome, ribosome biogenesis in eukaryotes, and cellular senescence signaling through pathways involving multiple cancer-related kinases and E2F family. Also, *RRM2* expression correlated with infiltrating levels of B cells, CD4+ T cells, and neutrophils. Subsequent analysis found that B cells and dendritic cells could predict the outcome of LUAD. B cells were identified as an independent risk factor among six types of immune cells through Cox analyses. At last, the correlation analysis showed *RRM2* correlated with 67.68% (624/922) of the immune signatures we performed.

**Conclusion:** Our research showed that *RRM2* could independently predict the prognosis of LUAD and was associated with immune infiltration. In particular, the tight relationship between *RRM2* and B cell marker genes are the potential epicenter of the immune response and one of the critical factors affecting the prognosis. Our findings laid the foundation for further research on the immunomodulatory role of *RRM2* in LUAD.

## Introduction

Lung cancer is the leading cause of cancer-associated deaths worldwide 1-3. The survival rate of lung cancer depends mainly on the stage of diagnosis. In general, the current 5-year survival rate is about 18%; however, the prognosis can be improved when confirmed early 4. Unfortunately, only about 15% of cases were at the early stage when diagnosed, while the vast majority (57%) were already at the advanced stage 4. Lung adenocarcinoma (LUAD) is a subclass of non-small cell lung cancer (NSCLC), which develops along the outer edge of the lungs within glandular cells in the small airways. LUAD accounted for approximately 40% of all lung cancer cases being the most common type of histology 5.

Whereas, due to the combination of adverse factors that span a range of different biological and clinical behaviors and the increased resistance to anti lung cancer drugs, existing targeted drugs have shown unsatisfactory efficacy 6. In NSCLC, little is known about the genomic and host factors that drive the progression of pre-invasive lesions. Investigating these factors can enhance our understanding of lung cancer biology, help to develop better screening strategies, and improve patient prognosis 7. Furthermore, the lack of specific markers for disease stages or tumor types represents a fundamental gap in the current understanding and treatment of LUAD.

Ribonucleoside-diphosphate reductase subunit M2 (*RRM2*) is the catalytic subunit of ribonucleotide reductase and modulates the enzymatic activity, which is essential for DNA replication and repair 8. According to recent reports, *RRM2* is involved in the progression of various cancers, including glioma, colorectal cancer, and bladder cancer 9, 10. Compared with normal tissues, *RRM2* is overexpressed in breast cancer patients and is associated with poor survival 11. Recent studies have shown that *RRM2* upregulation occurs in multiple myeloma tumors, and *RRM2* inhibition can inhibit multiple myeloma cell proliferation 12. Rahman et al. demonstrated that alteration of *RRM2* induces apoptosis by modulating Bcl-2 expression in lung cancer 13. Low expression of *RRM2* has been reported can be used to value the treatment response to platinum-based chemotherapy of lung cancer 14. Immunohistochemical evaluation of *RRM2* indicates that it has strong prognostic significance in some subsets of NSCLC patients (primarily women, non-smokers, and former smokers quitting longer than ten years) 15. Previous researches on the relationship between *RRM2* and lung cancer were too specific but short of a comprehensive view 16-18. Moreover, whether *RRM2* is a robust biomarker for LUAD, existing studies do not present a clear answer. Furthermore, the biological function of *RRM2* in LUAD remains to be established.

In this study, we examined the expression and prognostic value of *RRM2* in LUAD patients in the Cancer Genome Atlas (TCGA) and validated them in multiple independent cohorts. Moreover, using multidimensional analysis, we assessed the co-expression and functional network associated with *RRM2* in LUAD and studied its part in tumor immunity. The present study may potentially reveal new direction, biological targets, and strategies for the diagnosis, treatment, and prognosis assessment of LUAD.

## Materials and Methods

### Data mining from TCGA

LUAD patients' gene expression profiles, along with their clinical data such as age, gender, tumor stage, TNM classification, and survival status, were downloaded from the TCGA portal (v22.0, https://portal.gdc.cancer.gov/) with project ID: TCGA-LUAD.

### *RRM2* differential expression

In TCGA-LUAD cohort, the analysis of differential mRNA expression of *RRM2* in tumor and healthy tissues were examined by the Wilcoxon test, including unpaired or paired test. Oncomine (version 4.5, https://www.oncomine.org/) is a cancer microarray database and web-based data-mining platform. The mRNA expression level or copy number of *RRM2* in LUAD and normal tissue were examined in Oncomine. In order to select the dataset to be included in this study from Oncomine, the screening parameters were set as follows: 1) Set “Analysis Type” as “Cancer vs. Normal Analysis” and “Cancer Type” as “Lung Adenocarcinoma”; 2) Set “THRESHOLD” as “P-value<0.05”, “FOLD CHANGE” as “ALL”, and “GENE RANK” as “ALL”. UALCAN (http://ualcan.path.uab.edu/) is an online tool for analyzing cancer transcriptome data, which is based on public cancer transcriptome data (TCGA and MET500 transcriptome sequencing) 19. The "CPTAC analysis" module of UALCAN provides protein expression analysis option using data from Clinical Proteomic Tumor Analysis Consortium (CPTAC) Confirmatory/Discovery dataset 20. The comparison of *RRM2* protein expression between LUAD and normal lung was examined in UALCAN. Analyses in TCGA-LUAD and Oncomine were visualized through R package “beeswarm”. A difference was defined as significant at *p*-value < 0.05.

### *RRM2* expression in clinical characteristics sub-groups

The associations of *RRM2* expression with clinical were examined by a non-parametric test (i.e., if the data were divided into two groups, the Wilcoxon test was performed; if the data were divided into three groups or more, the Kruskal-Wallis test was performed). R software was used for the visualization.

### Survival analyses of *RRM2*

Survival analyses in the TCGA-LUAD cohort were conducted between high and low *RRM2* expression groups through Kaplan-Meier analysis with the log-rank test. PrognoScan (http://dna00.bio.kyutech.ac.jp/PrognoScan/) is a database for meta-analysis of the prognostic value of genes 21. In order to select the dataset to be included in this study from PrognoScan, the screening parameters were set as follows: 1) Set “Cancer Type” as “Lung cancer” and “Subtype” as “Adenocarcinoma”; 2) Set “THRESHOLD” as “P-value<0.05”. Kaplan-Meier Plotter (https://kmplot.com/) is a web application developed for meta-analysis-based biomarker assessment that can be used for breast, ovarian, lung, gastric, and liver cancer 22. The correlations between *RRM2* expression and survival in LUAD were additionally analyzed in PrognoScan and Kaplan-Meier Plotter. Analyses from TCGA-LUAD and PrognoScan were visualized through “survminer” and “survival” packages in R.

### The independent prognostic value of *RRM2*

To identify the independent prognostic value of RRM2 in LUAD and assess the correlation between important clinical characteristics and prognosis, we performed Cox analyses. First, we conducted univariate Cox analysis on every variable, in turn, to check their correlation with prognosis. Then, all variables were gathered for a multivariate Cox analysis to evaluate whether each of them has an independent prognostic value.

### LinkedOmics and GEPIA2 databases analysis

LinkedOmics (http://www.linkedomics.org) is a publicly available portal that includes multi-omics data from all 32 TCGA Cancer types^23^. In the “LinkFinder” module of LinkedOmics, we used the Pearson test to perform statistical analysis on *RRM2* co-expression, and the results were displayed in the form of volcano, heat, or scatter plots. “LinkInterpreter” module of LinkedOmics was applied to conducted analyses of Gene Ontology (Biological Process), Kyoto Encyclopedia of Genes and Genomes (KEGG) pathways, kinase-target enrichment, miRNA-target enrichment and transcription factor-target enrichment through Gene Set Enrichment Analysis (GSEA). The rank criterion was false discovery rate (FDR) < 0.05, and simulations was 1000. The Gene Expression Profiling Interactive Analysis (GEPIA2) database (http://gepia2.cancer-pku.cn/) is a web server for analyzing the RNA sequencing expression data of 9,736 tumors and 8,587 normal samples from the TCGA and the GTEx projects, using a standard processing pipeline^24^. GEPIA2 was applied to plot survival heatmaps of top co-expression genes and survival curves of top kinase regulators.

### The correlation between *RRM2* and six types of infiltrating immune cells

The Tumor Immune Estimation Resource (TIMER, https://cistrome.shinyapps.io/timer/) is a comprehensive resource for systematical analysis of immune infiltrates across diverse cancer types 25, 26. “Gene” module of TIMER was applied to explore the correlation between *RRM2* expression and abundance of six types of immune cells infiltrates, including B cells, CD4+ T cells, CD8+ T cells, neutrophils, macrophages, and dendritic cells, in LUAD, by tumor purity-corrected partial Spearman's correlation. Kaplan-Meier analysis was conducted to assess the prognostic value of each immune infiltrate. Multivariate Cox analysis was used to evaluate how *RRM2* and these six types of immune cells together affect outcomes.

### Correlation between *RRM2* and immune signatures

TISIDB is a central portal for tumor and immune system interactions, which integrates multiple heterogeneous data types 27. This online tool contained various immune gene signatures categorized by type of immune or their function. Gene signatures of chemokine, receptor, major histocompatibility complex (MHC), immunoinhibitor, immunostimulator, and 28 tumor-infiltrating lymphocytes (TILs) 28 were downloaded. The correlations between *RRM2* and these gene signatures were calculated via the “Correlation” module of TIMER with tumor purity-corrected partial Spearman's correlation.

## Results

### Patient characteristics

The clinical characteristics of 522 cases were obtained from the TCGA-LUAD cohort, including age, gender, tumor stage and TNM classification. As shown in **Table 1**, 241 (46.17%) cases were younger than or equal to 65 years old, 262 (50.19%) were older than 60 years old, while 19 (3.64%) were unknown. 280 (53.64%) were females, and 242 (46.36%) were males. Stage I was found in 279 patients (53.45%), stage II in 124 (23.75%), stage III in 85 (16.28%), stage IV in 26 (4.98%), and unknown in 8 (1.53%). The patients with T1 (32.95%) and T2 (53.83%) made up the majority of the total patients, and the remaining are T3 (9.00%), T4 (3.64%), and unknown (0.57%). 353 (67.62%) were at M0, 25 (4.79%) were at M1, while the rest 144 cases (27.59%) were unknown. In the distribution of N classification, N0, N1, N2, N3, and unknown accounted for 64.18%, 18.77%, 14.37%, 0.38%, and 2.30%, respectively**.**

### High *RRM2* expression in LUAD

**Figure 1:**
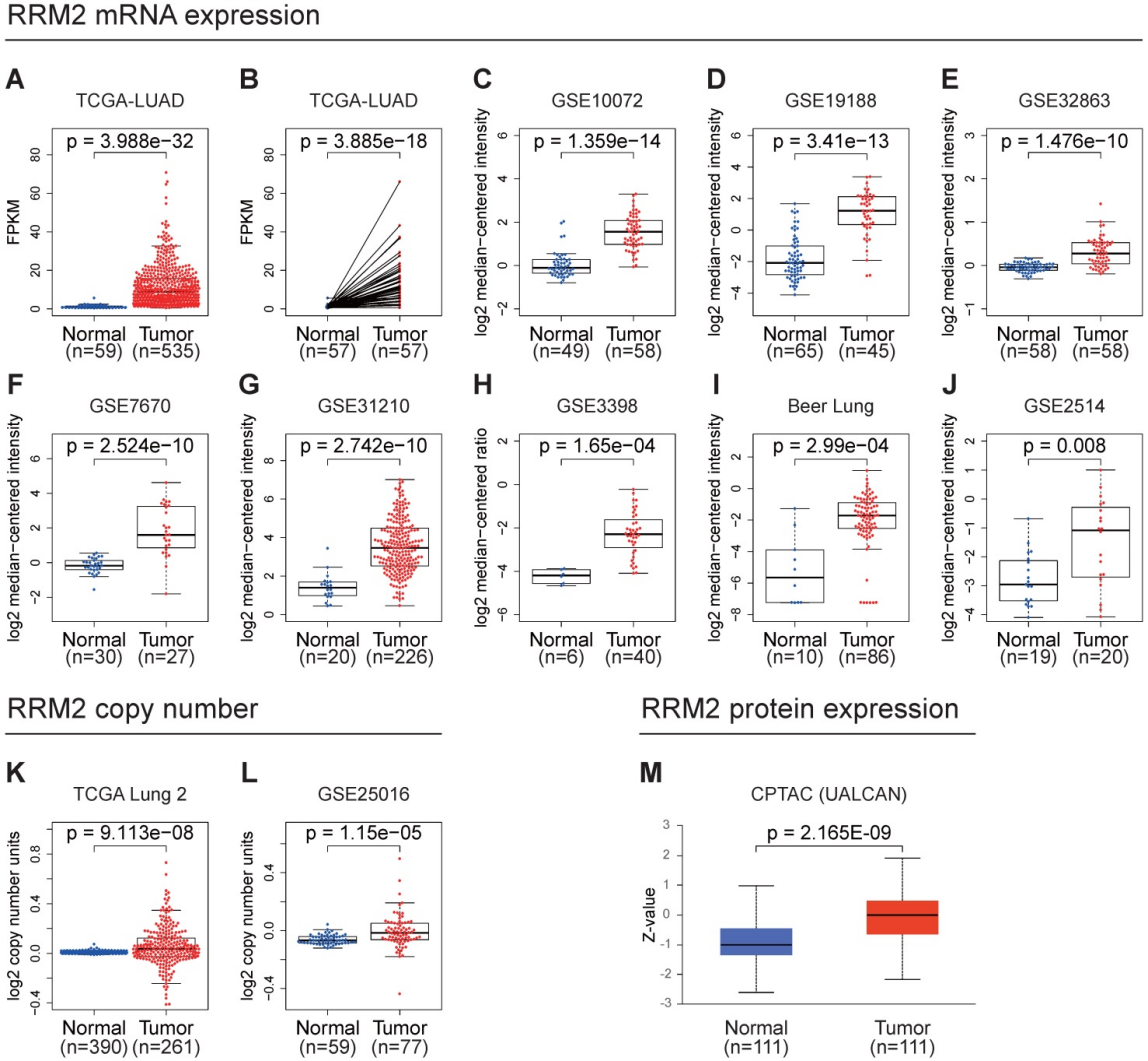
***RRM2* is highly expressed in LUAD.** (**A**) *RRM2* mRNA expression comparison between normal and tumor tissues in the TCGA-LUAD cohort (unpaired Wilcoxon test). (**B**) *RRM2* mRNA expression comparison between normal and adjacent tissues in the TCGA-LUAD cohort (paired Wilcoxon test). (**C - J**) *RRM2* mRNA expression comparisons between normal and tumor tissues obtained from the Oncomine web tool (Wilcoxon test). (**K - L**) *RRM2* copy number comparisons between normal and tumor tissues obtained from the Oncomine web tool (Wilcoxon test). (**M**) *RRM2* protein expression comparison between normal and tumor tissues obtained from the UALCAN web tool (Wilcoxon test). TCGA: The Cancer Genome Atlas; LUAD: lung adenocarcinoma; CPTAC: Clinical Proteomic Tumor Analysis Consortium; The title of each graphic refers to the project name in TCGA, GEO, Oncomine, or UALCAN; P-value < 0.05 was used to assess differences.

In the TCGA-LUAD cohort, we compared the mRNA expression of *RRM2* in tumor and normal or their adjacent tissues. The unpaired and paired tests both indicated that the mRNA expression of *RRM2* in LUAD was elevated (**Figure 1A, 1B**). Moreover, we checked the Oncomine online database finding that many datasets suggested that the mRNA expression of *RRM2* increased in tumor tissues (**Figure 1C-J**). Besides, we found that the copy number of *RRM2* also increased in tumor tissue (**Figure 1K, 1L**). We examined *RRM2* protein expression in UALCAN database, discovering it was highly expressed in tumor tissues (**Figure 1M**).

### Distribution of *RRM2* expression in clinical characteristics sub-groups

**Figure 2:**
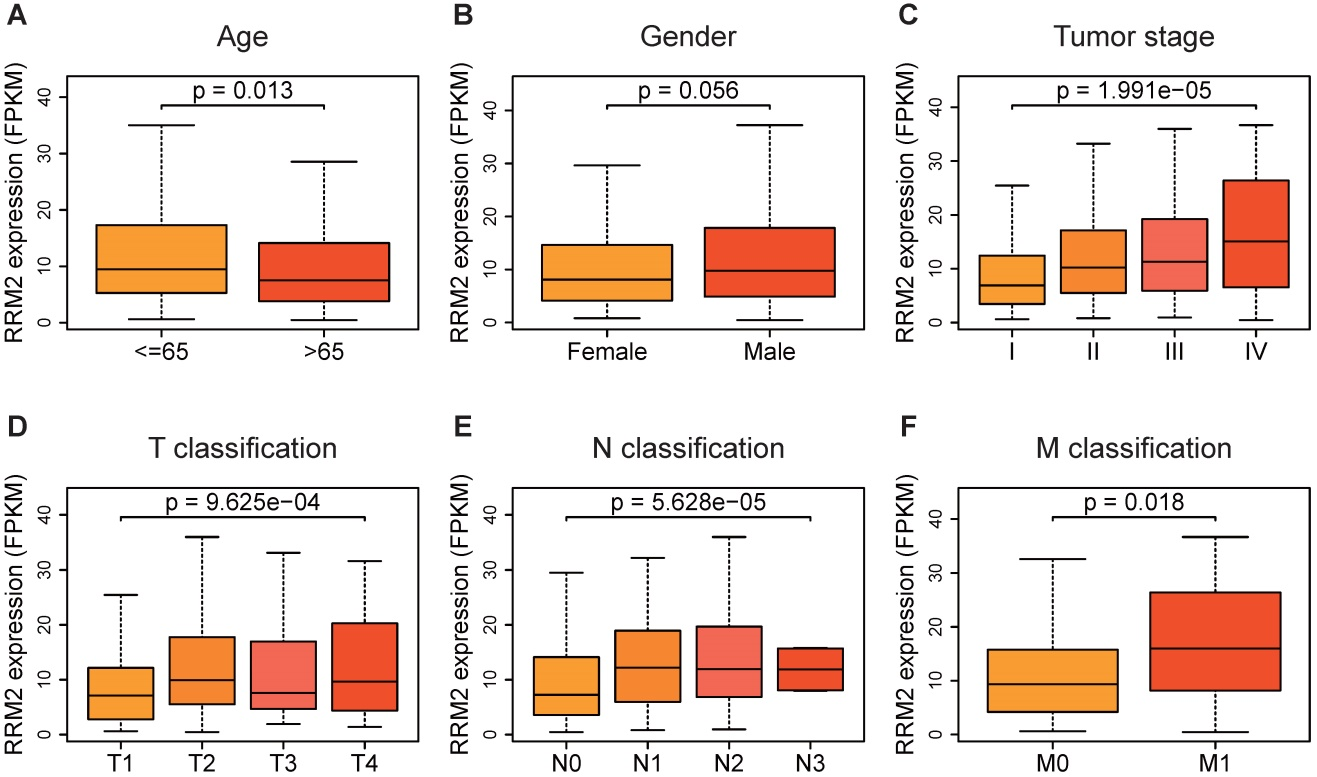
***RRM2* expression in sub-groups of clinical characteristics.** (**A, B, F**) *RRM2* expression distribution analyses stratified based on age, gender, and M classification (Wilcoxon test). (**C - E**)* RRM2* expression distribution analyses stratified based on tumor stage and TN classification (Kruskal-Wallis test). P-value < 0.05 was used to assess differences.

Based on the gene profile and clinical data extracted from TCGA-LUAD, the expression of *RRM2* in patients with younger age (≤ 65 years old) was found significantly higher compared to patients who were older than 65 years old (*p*-value = 0.013, **Figure 2A**). The distribution of *RRM2* showed a significant difference among the tumor stages. *RRM2* was highly expressed as tumor stage increased (*p*-value = 1.991e-05, **Figure 2C**). Similarly, *RRM2* increased with the N classification (*p*-value = 9.625e-04, **Figure 2E**). Moreover, *RRM2* became increased expression in patients with tumor metastasis (*p*-value = 0.018, **Figure 2F**). As shown in** Figure 2D**, *RRM2* was significantly differently distributed in the sub-groups of T classification, with highly expressed in T2 and T4 classification (*p*-value = 9.625e-04). Furthermore, different genders were not associated with the expression of *RRM2* (**Figure 2B**).

### High *RRM2* expression indicates significant worse survival in LUAD

**Figure 3:**
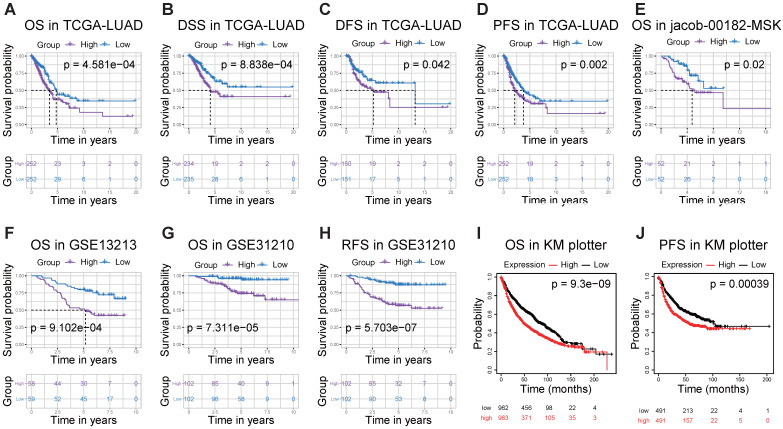
***RRM2* is associated with survival outcome.** (**A - D**) Survival analyses of *RRM2* in the TCGA-LUAD cohort by Kaplan-Meier estimator with a log-rank test. (**E - H**) Survival analyses of *RRM2* by Kaplan-Meier estimator with log-rank test obtained from PrognoScan web tool. (**I, J**) Survival analyses of *RRM2* by Kaplan-Meier estimator with log-rank test obtained from the Kaplan Meier plotter web tool. Survival differences are compared between patients with high and low (grouped according to median) expression of *RRM2*; The numbers below the figures denote the number of patients at risk in each group; The title of each graphic refers to the project name in TCGA, GEO, PrognoScan, or Kaplan Meier plotter web tool; TCGA: The Cancer Genome Atlas; LUAD: lung adenocarcinoma; OS: overall survival; DSS: disease-specific survival; DFS: disease-free survival; PFS: progression-free survival; RFS: Relapse free survival; P-value < 0.05 was used to assess differences.

Then, to understand the correlation between *RRM2* expression and patients' outcomes, we used the Kaplan-Meier survival curves to evaluate and compare the survival differences between patients with high and low (grouped according to median) expression of *RRM2* (**Figure 3**). In the TCGA-LUAD cohort, the high *RRM2* expression group had significantly shorter overall survival, and the median overall survival of group of high expression vs. low expression was 3.47 years vs. 4.73 years (log-rank test, *p*-value = 4.581e-04, **Figure 3A**). The high expression group also had a significant unfavorable disease-specific survival (log-rank test, *p*-value = 8.838e-04, **Figure 3B**) and disease-free survival (log-rank test, *p*-value = 0.042, **Figure 3C**) than that in low expression group. Furthermore, in the comparison of progression-free survival between high and low expression patients, the high expression group had a worse median survival than that in low expression group (2.13 years vs. 3.78 years, log-rank test, *p*-value = 0.002, **Figure 3D**). Besides, we checked the PrognoScan and Kaplan-Meier Plotter finding that high *RRM2* expression associated with poor overall survival, relapse-free survival, and progression-free survival (**Figure 3E-J**).

### High expression of *RRM2* is a potential independent risk factor

As shown above, a higher expression of *RRM2* was related to a higher tumor stage (**Figure 2**). Kaplan-Meier analysis indicated that high expression of *RRM2* was associated with poor survival (**Figure 3**). To find more evidence, the Cox proportional-hazards model was constructed. Univariate Cox analyses in overall survival showed that tumor stage, TNM classification, and *RRM2* were acting potential risk roles in LUAD. Additionally, the multivariate Cox analyses confirmed the critical value of T classification and *RRM2*, proving that they can predict tumor prognosis independently of other factors in overall survival (**Table 2**). Also, the Cox analysis based on disease-specific survival only showed that RRM2 was the riskiest factor. Consistently, in the progression-free survival Cox analysis, *RRM2* was the only marker that can predict survival independently. Taking together, *RRM2* is a potential independent risk factor in LUAD.

### *RRM2* co-expression networks in LUAD

**Figure 4:**
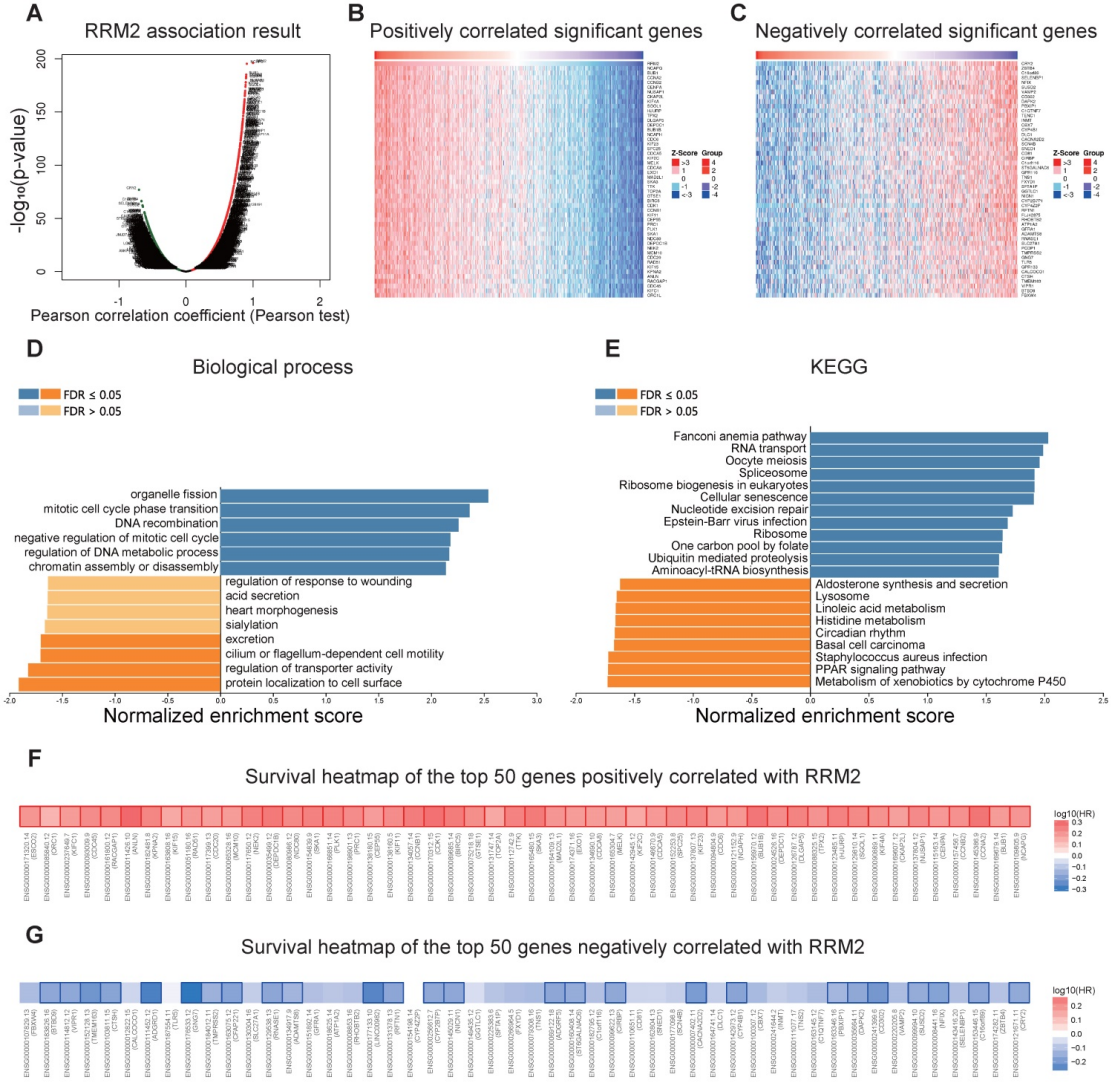
***RRM2* co-expression genes in LUAD (LinkedOmics).** (**A**) The global *RRM2* highly correlated genes identified by the Pearson test in LUAD. Red and green dots represent positively and negatively significantly correlated genes with *RRM2*, respectively. (**B and C**) Heatmaps showing the top 50 genes positively and negatively correlated with *RRM2* in LUAD. (**D and E**) Significantly enriched GO: Biological process annotations and KEGG pathways of *RRM2* in LUAD. (**F and G**) Survival heatmaps of the top 50 genes positively and negatively correlated with *RRM2* in LUAD. The survival heatmaps show the hazard ratios in the logarithmic scale (log10) for different genes. The red and blue blocks denote higher and lower risks, respectively. The rectangles with frames mean the significant unfavorable and favorable results in prognostic analyses (*p*-value < 0.05). FDR: false discovery rate; KEGG: Kyoto Encyclopedia of Genes and Genomes; GO: Gene Ontology; LUAD: Lung adenocarcinoma.

In order to better understand the biological meaning of *RRM2* in LUAD, the “LinkFinder” module in LinkedOmics was applied to check the co-expression pattern of *RRM2*. As plotted in **Figure 4A**, it shows that 6152 genes (red dots) positively correlated with *RRM2*, and 7399 genes (green dots) negatively correlated (*p*-value < 0.05). **Figures 4B** and **4C** show the heatmaps of the top 50 genes positively and negatively associated with *RRM2*, respectively. Moreover, **Table S1** detailed lists the co-expressed genes.

Significant Gene Ontology term annotation by GSEA showed that *RRM2* co-expressed genes involved mainly in the organelle fission, mitotic cell cycle phase transition, DNA recombination, negative regulation of mitotic cell cycle, regulation of DNA metabolic process, and chromatin assembly or disassembly. In contrast, the protein localization to cell surface, regulation of transporter activity, cilium or flagellum-dependent cell motility, and excretion were inhibited (**Figure 4D** and **Table S2**). KEGG analysis showed genes were primarily enriched in the Fanconi anemia pathway, RNA transport, oocyte meiosis, spliceosome, ribosome biogenesis in eukaryotes, and cellular senescence pathways, etc. (**Figure 4E** and **Table S3**).

*RRM2* expression displayed a strong positive association with the expression of *NCAPG* (positive rank #1, *r* = 0.908, *p*-value = 5.28E-196), *BUB1* (*r* = 0.899, *p*-value = 8.98E-186), and *CCNA2* (*r* = 0.897, *p*-value = 3.79E-184), etc. Remarkably, the top 50 positively genes owned highly probability of becoming high-risk markers in LUAD, interesting, of which 50/50 genes had high hazard ratio (HR, *p*-value < 0.05) (**Figure 4F**). In comparison, 26 of the top 50 negatively correlated genes had low HR (*p*-value <0.05) (**Figure 4G**).

### Regulators of *RRM2* networks in LUAD

To understand the regulatory factors of *RRM2* in LUAD, we further analyzed the enrichment of kinases, miRNAs, and transcription factors of *RRM2* co-expressed genes. The top 5 kinases related mainly to *CDK1*, *PLK1*, *CDK2*, *AURKB*, and *ATM* (**Table 3** and **Table S4**). In fact, 3 of the top 5 kinase genes include *CDK1*, *PLK1*, and *AURKB,* were significantly highly expressed in tumor tissues and significantly related to the overall survival of LUAD (**Figure S1**). Interestingly, the co-expressed genes of *RRM2* were not enriched on any miRNA targets significantly (**Table 3** and **Table S5**). Transcription factor enrichment results showed that the co-expressed genes of *RRM2* were mainly enriched in the *E2F* transcription factor family (**Table 3** and**Table S6**), including V$E2F_Q6, V$E2F_Q4, V$E2F1_Q6, V$E2F4DP1_01, and V$E2F1DP1_01.

### Correlation analysis between *RRM2* expression and six main infiltrating immune cells

**Figure 5:**
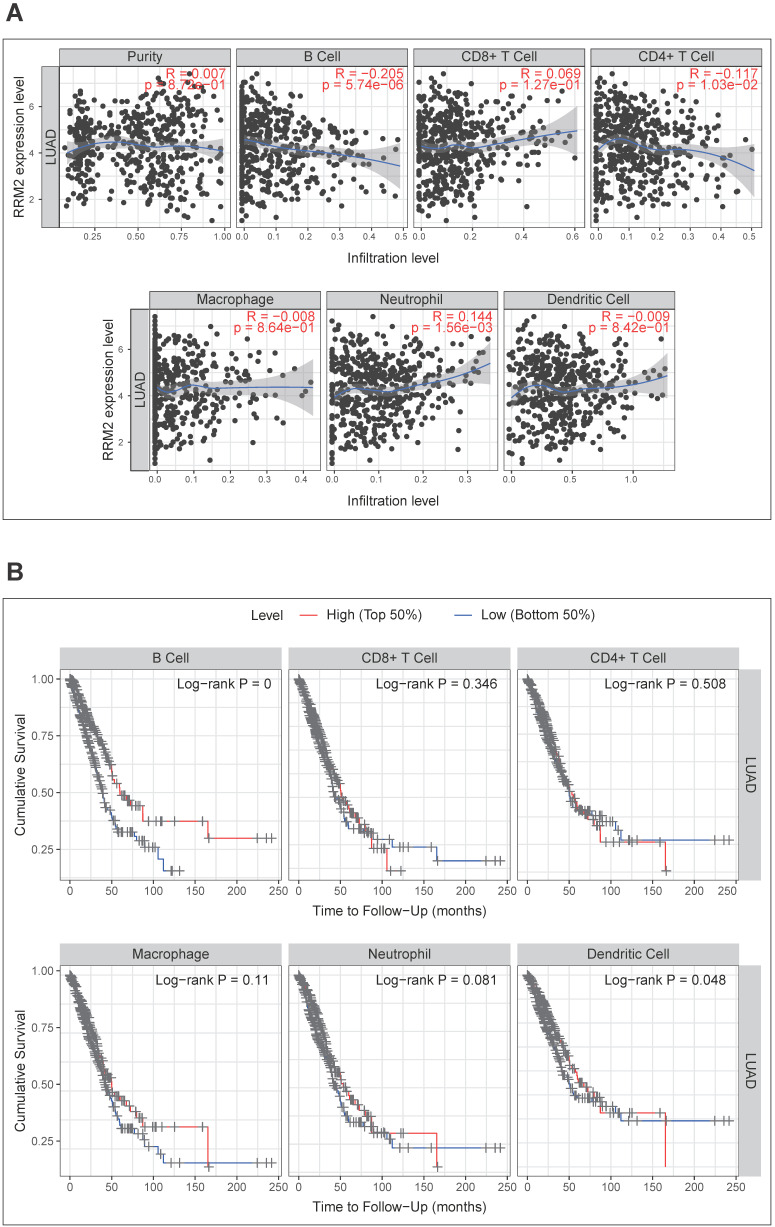
**Correlation analysis between *RRM2* expression and six types of infiltrating immune cells in LUAD.** (**A**) Correlation of *RRM2* expression with six types of immune infiltration cells obtained from TIMER (purity-corrected Spearman test). (**B**) Overall survival curve of each of the six types of immune cells produced by Kaplan-Meier estimator from TIMER. Survival differences are compared between patients with high and low (grouped according to median) infiltrating of each kind of immune cells*;* LUAD: Lung adenocarcinoma; TIMER: The Tumor Immune Estimation Resource.

Then, we investigated whether *RRM2* expression was correlated with six main infiltrating immune cells (B cells, CD4 T cells, CD8+ T cells, neutrophils, macrophages, and dendritic cells) in LUAD using TIMER database. The analysis showed that *RRM2* expression levels correlated with B cells (*r* = -0.205, *p*-value = 5.74e-06), CD4+ T cells (*r* = -0.117, *p*-value = 1.03e-02), and neutrophils (*r* = 0.144, *p*-value = 1.56e-03) (**Figure 5A**). Moreover, we evaluated the prognostic value of each of the six types of immune cells via Kaplan-Meier analysis, finding B cells (*p*-value = 0 in log-rank test) and dendritic cells (*p*-value = 0.048 in log-rank test) can predict the outcome of LUAD (**Figure 5B**). At last, Cox proportional hazard models were applied to assess the impacts of *RRM2* expression and the six types of immune cells on the overall survival of LUAD. *RRM2* showed significant risk in univariate analyses (HR = 1.291, 95% CI = 1.150-1.450, *p*-value = 0), and multivariate analyses (HR = 1.255, 95% CI = 1.112-1.415, *p*-value = 0), indicating it can predict tumor outcomes independently of the other six immune cells. Interestingly, B cells also displayed the similar performance in univariate (HR = 0.024, 95% CI = 0.004-0.142, *p*-value = 0) and multivariate analyses (HR = 0.008, 95% CI = 0.001-0.106, *p*-value = 0) (**Table 4**). Taking together, the significantly infiltrating with B cells seemed like one of the critical factors that *RRM2* holds to influence the outcome of LUAD pronounced.

### Correlation between *RRM2* expression and immune signatures

Lastly, to expand the understanding of the crosstalk between *RRM2* and multiple immune marker genes of 28 TILs, immune inhibitory or stimulatory, cytokine-related, cancer-testis antigen, and MHC, we did correlation analysis between them. The analysis showed that the expression level of *RRM2* in LUAD was significantly correlated with 67.68% (624/922) immune marker genes (**Table S7**). In the significant correlated immune markers, 352/624 (56.41%) were positively, while, 272/624 (43.59%) were negatively related. On the whole, the top 5 positively correlated marker genes with *RRM2* were *CCNA2* (*r* = 0.901976588, *p*-value = 3.2894E-181), *CCNB1* (*r* = 0.874181398, *p*-value = 3.775E-156), *EXO1* (*r* = 0.868718344, *p*-value = 6.3113E-152), *PRC1* (*r* = 0.862049011, *p*-value = 5.0769E-147), and *KIF11* (*r* = 0.858239682, *p*-value = 2.474E-144). Besides, the top 5 negatively correlated markers with *RRM2* were *CD302* (*r* = -0.563236799, *p*-value = 1.30947E-42), *DAPK2* (*r* = -0.550326103, *p*-value = 2.23251E-40), *GNG7* (*r* = -0.542454019, *p*-value = 4.59789E-39), *DLC1* (*r* = -0.525310139, *p*-value = 2.55007E-36), and *GPRC5C* (*r* = -0.524798161, *p*-value = 3.06273E-36).

As for immunoinhibitory genes, results showed *CD274 LAG3, PDCD1LG2, PDCD1, IDO1, KIR2DL3, PVRL2, TIGIT, IL10RB, HAVCR2, CTLA4, IL10, TGFBR1* have positively correlations with *RRM2* expression, while, *ADORA2A* and *BTLA* have negatively correlations with *RRM2* expression. Moreover, the top 5 immunostimulatory genes positively correlated *RRM2* expression were *PVR, CD276, MICB, TNFSF4*, and *TNFRSF9*, besides, the top 5 negative markers were *TNFSF13, TMEM173, IL6R, TNFRSF13B*, and *CD40LG* (**Table S7**).

In the previous section, we found that B cell infiltration may be one of the key reasons that caused *RRM2* to become a prognostic factor. Thus, we were very interested in the correlation between *RRM2* and B cell marker genes. **Table 5,** which was extracted from **Table S7,** shows the purity-corrected partial Spearman's correlation between *RRM2* and B cell markers. In B cells, *RRM2* is highly correlated with *CCNA2* (#1, *r* = 0.901976588, *p*-value = 3.2894E-181), *CDKN3* (#2, *r* = 0.828306212, *p*-value = 1.2672E-125), *GNG7* (#3, *r* = -0.542454019, *p*-value = 4.59789E-39), *FCER1A* (#4, *r* = -0.498295422, *p*-value = 2.66219E-32), and *MICAL3* (#5, *r* = 0.344177052, *p*-value = 3.71747E-15). In total, 35/57 of the B cell marker genes associated significantly to *RRM2* expression, of which the number of positive correlations was 9/35 (25.71%), and the negative was 26/35 (74.29%). We plotted the survival heatmaps of the significant B cell markers correlated significantly with *RRM2* expression in **Figure S2**. Notably, almost all of the positively related markers showed a high probability of becoming high-risk factors in LUAD, of which 3/9 markers had elevated HR (*p*-value < 0.05) (**Figure S2A**). In comparison, there were 20/26 genes with low HR (*p*-value < 0.05) in negatively related markers (**Figure S2B**).

## Discussion

The present study found that *RRM2* was highly expressed in LUAD tumor tissue and significantly predicts a poor prognosis; also, the higher tumor stage got a higher expression. Univariate and multivariate Cox analyses indicated the *RRM2* might be a potential independent biomarker for LUAD prognosis. Then we examined the co-expression and regulators networks of *RRM2*. At last, we conducted a correlation analysis between *RRM2* and immune infiltration or immune signatures, finding that *RRM2* was related to most of the immune marker genes, and its infiltration in B cells may be one of the factors for its prognostic ability. Such work we have done aimed to guide future research in LUAD.

The dysregulated cell cycle has been identified in many types of cancer 29. Ribonucleotide reductase is an enzyme involved in the cell cycle. It consists of two subunits, namely the regulatory subunit *RRM1* and the catalytic subunit *RRM2*, which is essential for DNA replication and repair 30, 31. *RRM2* is a rate-limiting enzyme used for DNA synthesis and repair, plays a vital role in many critical cellular processes, such as cell proliferation, invasiveness, migration, angiogenesis, and aging 8. The present bioinformatics analysis showed that *RRM2* was overexpressed in breast cancer patients to normal tissues and was associated with worse survival 32. Overexpression of *RRM2* was shown to be associated with an unfavorable prognosis in HER-2 positive breast cancer patients 33. A recent study indicated that *RRM2* upregulation occurred in multiple myeloma tumors and that *RRM2* knockdown inhibited multiple myeloma cell proliferation 12. Li et al. illustrated that *RRM2* was overexpressed in human glioblastoma cells, and promoted proliferation, migration, and invasion of human glioblastoma cells 34. Suppression of *RRM2* inhibits cell proliferation, causes cell cycle arrest, and promotes the apoptosis of human neuroblastoma cells 35. In our story, the investigation of differential expression in LUAD found that *RRM2* was highly expressed in tumor tissues, which was subsequently examined in multiple independent cohorts.

Then, we found that distinct histologic staging was associated with *RRM2* expression. High expression of *RRM2* happened in the more upper stage, which indicated *RRM2* is mainly involved in the advanced period in LUAD, indicating a possible relationship existed between *RRM2* expression and disease outcomes in LUAD. Thus, we carried out survival analysis in TCGA-LUAD, revealing that high *RRM2* expression was associated with poor outcomes, which was also checked in the other independent cohorts. Besides, the Cox analyses further proved that *RRM2* was an independent risk factor in LUAD. Therefore, our results indicate that *RRM2* upregulation occurs in LUAD, and as a potential diagnostic and prognostic marker, it is worthy of further clinical verification.

We explored the regulators responsible for *RRM2* dysregulation and found that *RRM2* was related to kinase networks, such as *CDK1, PLK1, CDK2, AURKB,* and* ATM.* These kinases mainly regulated mitosis, genome stability, and cell cycle, and showed survival prognosis value and differential expression in LUAD. *CDK1* is a prototype kinase, a central regulator that drives cells through G2 phase and mitosis 36. *CDK1* orchestrates the transition from the G2 phase into mitosis, and as cancer cells often display enhanced *CDK1* activity, it has been proposed as a tumor-specific anti-cancer target 37. Data mining from different databases demonstrated *CDK1* upregulation in LUAD. Furthermore, *CDK1* upregulation is associated with poor prognosis 38. However, the molecular mechanism and potential application of *CDK1* in lung cancer have not been determined 39. *PLK1* is indispensable for finely regulating cell division and maintenance of genomic stability in mitosis, spindle assembly, and DNA damage response 40. Studies have shown that *PLK1* is highly expressed in most human carcinoma, and its overexpression is associated with an unfavorable prognosis 41-43. In human tumors, the overexpression of *AURKB* is associated with poor prognosis. *AURKB* inhibitors are in clinical trials for stage I-II leukemia 44. *AURKB* is also involved in resistance to specific anti-tumor agents, such as paclitaxel in NSCLC 45. Bertran-Alamillo et al. revealed that *AURKB* is related to acquired resistance to *EGFR* TKIs, and *AURKB* can become a potential biological target for anti-*EGFR* therapy of NSCLC without carrying resistance mutations 46.

In this study, we found that the *E2F* family was the main transcription factor constituting *RRM2* dysregulation. *E2F* is a group of genes that encodes a family of transcription factors in advanced eukaryotes. They participate in regulating the cell cycle and DNA synthesis in mammalian cells 47. Our analysis did not find miRNAs that are significantly associated with *RRM2*, which may be since *RRM2* is involved in the role of mRNA spliceosomes and is far away from miRNA cellular. Our results indicate that *E2F1* is a vital regulator of *RRM2*, and *RRM2* may play a role in regulating the cell cycle and proliferation ability of LUAD through this factor.

Our study found that *RRM2* expression levels had significant correlations with B cells, CD4+ T cells, and neutrophils infiltrating (**Figure 5A**). Moreover, the subsequent Kaplan-Meier analysis found that B cells and dendritic cells could predict the outcome of LUAD (**Figure 5B**). Cox analyses showed that B cells and *RRM2* were significant independent risk factors among all variables (**Table 4**). These findings indicated that B cell infiltration might be one of the critical factors of *RRM2* with prognostic value.

Next, we conducted correlation analyses between *RRM2* and several immune signatures. We detailed analyzed the correlation between *RRM2* and B cell signatures finding 35/57 (61.40%) of the B cell marker genes associated significantly to *RRM2* expression, including *CCNA2, CDKN3,* and* GNG7*. *CCNA2*, also known as cyclin A2, belongs to the highly conserved cyclin family and plays a key role in cell cycle control 48. A recent study demonstrated that *CCNA2* is a crucial regulator of NSCLC cells metastasis promoting invasion and migration of NSCLC cells through integrin αVβ3 signaling pathway 49. *CDKN3* gene encodes a dual-specificity protein phosphatase, which was previously thought to suppress tumors by controlling mitosis via *CDK1*/*CDK2* 50. It is well known that *CDKN3* is overexpressed in multiple human tumor tissues and cell lines 51, 52. The high expression of *CDKN3* in human cancer tissue may reflect the increased proportion of mitotic cells in the tumor 53. Elevated *CDKN3* expression is associated with the adverse outcome of LUAD. Overexpression of *CDKN3* in LUAD is not because of alternative splicing or mutation, but increased mitotic activity, which is related to *CDKN3* as a tumor suppressor 53. *GNG7* is a subunit of heterotrimeric G protein, which is commonly expressed in various tissues, but low in cancer 54. It has been speculated that *GNG7* may be involved in cell contact-induced growth arrest and thus blocks uncontrolled cell proliferation in multicellular organisms 55. Correlate analysis provides an exhaustive characterization of the association between *RRM2* and immune signatures in LUAD patients, indicating that *RRM2* is a crucial player in immune escape in the tumor microenvironment. Also, the correlation between *RRM2* and B cell markers is particularly vital to the prognosis of LUAD patients. It is worth noting that *RRM2* may be a key factor mediating B cell therapy, which is needed to be clarified in further research.

At present, how *RRM2* affects the prognosis of LUAD and the biological function of *RRM2* in LUAD is still in its infancy. Souglakos' study revealed that the efficacy of docetaxel/gemcitabine in lung adenocarcinoma patients was associated with *RRM2* mRNA expression from 42 patients 18. Huang and colleagues studied 44 patient samples and found that the overexpression of *RRM2* promoted proliferation, inhibited apoptosis, and increased the chemotherapy resistance of NSCLC cells through upregulating EGFR expression and AKT phosphorylation 17. Recently, Yang's team worked on 30 patients and found that *RRM2* was upregulated in NSCLC tumors and cell lines, leading to poor prognosis 16. Nevertheless, these are not enough, and there are still many unknowns to be elaborated. Insufficient sample inclusion in previous researches will lead to statistical bias. So, in our study, we attempted a comprehensive and multi-angle analysis of RRM2 by combining multiple databases to explain the role of *RRM2* in LUAD. The previous studies did not highlight the independent prognostic capabilities of *RRM2*, which has been confirmed in our study. Immunity plays a crucial role in the development of tumors. Existing research is failed to declare how *RRM2* affects LUAD immunity. To this end, we not only elaborated the relationship between various immune cells and *RRM2*, but also discussed in detail the immune signature genes related to *RRM2* that affect the prognosis. Such a research design for *RRM2* and LUAD has never reported before, which are aiming at providing one more possibility and direction for future LUAD research.

Our research also has some limitations, described as follows. 1) The results came from retrospective data, and more prospective data were needed for proving the clinical utility of it. 2) There is lacking wet experimental data in this study explaining the relationship between *RRM2* and their mechanism in LUAD samples. More effort is needed to clarify the potential relationship between *RRM2* and LUAD.

## Conclusions

In summary, this study provided all-round evidence for the value of *RRM2* in the progress of lung cancer and its potential as a bio-target and prognostic predictor of LUAD. Our results showed that the up-regulation of *RRM2* in LUAD indicates an adverse outcome, which may be caused by multiple steps that weaken genomic stability or disturb the cell cycle. Furthermore, we find that *RRM2* has a significant correlation with mostly immune signatures. Moreover, the connection between *RRM2* and B cell markers needs to be noted, which may be the new direction of future LUAD research.

## Supplementary Material

Supplementary figures and tables.

## Figures and Tables

**Table 1:** The characteristics of patients in the TCGA-LUAD cohort

Characteristic	Total (522)	Percentage (%)
**Age**		
≤65	241	46.17
>60	262	50.19
unknown	19	3.64
**Gender**		
Female	280	53.64
Male	242	46.36
**Tumor stage**		
Stage I	279	53.45
Stage II	124	23.75
Stage III	85	16.28
Stage IV	26	4.98
unknown	8	1.53
**T classification**		
T1	172	32.95
T2	281	53.83
T3	47	9.00
T4	19	3.64
unknown	3	0.57
**N classification**		
N0	335	64.18
N1	98	18.77
N2	75	14.37
N3	2	0.38
unknown	12	2.30
**M classification**		
M0	353	67.62
M1	25	4.79
unknown	144	27.59

TCGA: The Cancer Genome Atlas; LUAD: lung adenocarcinoma.

**Table 2:** Univariate analysis and multivariate analysis of the correlation of *RRM2* expression and important clinical characteristics with survival among lung adenocarcinoma patients

Parameter	Univariate analysis			Multivariate analysis		
	HR	95% CI	*P*-value	HR	95% CI	*P*-value
**Overall survival**						
Age	1.000	0.983-1.018	9.685E-01	1.012	0.994-1.030	2.059E-01
Gender	1.074	0.768-1.503	6.757E-01	0.939	0.668-1.321	7.191E-01
Tumor stage	1.584	1.355-1.852	**7.826E-09**	1.469	0.975-2.215	6.616E-02
T classification	1.607	1.322-1.954	**1.953E-06**	1.293	1.030-1.624	**2.667E-02**
N classification	1.724	1.422-2.091	**3.055E-08**	1.083	0.755-1.552	6.661E-01
M classification	1.825	1.028-3.240	**3.982E-02**	0.737	0.261-2.082	5.642E-01
*RRM2*	1.028	1.015-1.041	**1.542E-05**	1.286	1.118-1.480	**4.324E-04**
**Disease-specific survival**						
Age	0.981	0.960-1.002	7.232E-02	0.995	0.973-1.017	6.300E-01
Gender	0.900	0.576-1.406	6.424E-01	0.781	0.497-1.226	2.832E-01
Tumor stage	1.597	1.301-1.960	**7.515E-06**	1.473	0.840-2.580	1.763E-01
T classification	1.514	1.157-1.982	**2.534E-03**	1.242	0.918-1.680	1.602E-01
N classification	1.628	1.267-2.094	**1.428E-04**	1.014	0.623-1.650	9.562E-01
M classification	2.258	1.126-4.530	**2.184E-02**	0.818	0.183-3.653	7.922E-01
*RRM2*	1.035	1.020-1.051	**5.702E-06**	1.330	1.106-1.601	**2.483E-03**
**Progression-free survival**						
Age	0.993	0.977-1.010	4.155E-01	0.998	0.981-1.015	8.134E-01
Gender	0.987	0.708-1.375	9.384E-01	0.920	0.657-1.287	6.255E-01
Tumor stage	1.320	1.123-1.551	**7.468E-04**	1.475	0.975-2.231	6.606E-02
T classification	1.356	1.112-1.653	**2.653E-03**	1.185	0.932-1.505	1.660E-01
N classification	1.299	1.060-1.591	**1.152E-02**	0.824	0.570-1.191	3.026E-01
M classification	1.432	0.771-2.658	2.551E-01	0.508	0.168-1.536	2.304E-01
*RRM2*	1.022	1.009-1.035	**6.605E-04**	1.215	1.064-1.388	**4.101E-03**

HR: hazard ratio; CI: confidence interval. Bold values indicate *p*-value < 0.05.

**Table 3:** The kinases, miRNAs, and transcription factors-target networks of *RRM2* in LUAD

Enriched category	Gene set	Leading edge number	NES	FDR
Kinase target	Kinase_CDK1	67	2.3829	0
	Kinase_PLK1	31	2.2374	0
	Kinase_CDK2	84	2.2281	0
	Kinase_AURKB	31	2.1503	0
	Kinase_ATM	38	2.1011	0
miRNA Target	AGCGCTT,MIR-518F,MIR-518E,MIR-518A	5	-1.4072	0.48209
	CCCAGAG,MIR-326	30	-1.4088	0.55154
	AGGGCAG,MIR-18A	34	-1.4499	0.55987
	GAGCTGG,MIR-337	34	-1.2417	0.61109
	ACACTGG,MIR-199A,MIR-199B	36	-1.3139	0.61239
Transcription Factor	V$E2F_Q6	87	2.1966	0
	V$E2F_Q4	87	2.1905	0
	V$E2F1_Q6	91	2.1891	0
	V$E2F4DP1_01	91	2.1752	0
	V$E2F1DP1_01	90	2.1731	0

LUAD: Lung adenocarcinoma; NES: normalized enrichment score; FDR: false discovery rate.

**Table 4:** Cox analysis of the correlation between *RRM2* expression and six types of immune cells and prognosis in patients with lung adenocarcinoma

Variables	Univariate analysis				Multivariate analysis			
	coef	HR	95% CI	P-value	coef	HR	95% CI	P-value
B cell	-3.715	0.024	0.004-0.142	**0**	-4.822	0.008	0.001-0.106	**0**
CD8+ T cell	-1.209	0.299	0.083-1.074	0.064	0.382	1.466	0.244-8.813	0.676
CD4+ T cell	-1.061	0.346	0.078-1.541	0.164	3.101	22.212	1.494-330.241	**0.024**
Macrophage	-0.55	0.577	0.094-3.539	0.552	0.385	1.469	0.112-19.184	0.769
Neutrophil	-1.006	0.366	0.055-2.452	0.3	-2.146	0.117	0.002-5.856	0.283
Dendritic	-0.592	0.553	0.308-0.993	**0.047**	-0.026	0.974	0.258-3.683	0.97
*RRM2*	*0.255*	1.291	1.150-1.450	**0**	0.227	1.255	1.112-1.415	**0**

coef: regression coefficient; HR: hazard ratio; CI: confidence interval; Bold values indicate *p*-value < 0.05.

**Table 5:** Correlation analysis between *RRM2* and of B cell markers in LUAD

Variables	None adjusted		Tumor purity adjusted	
	Cor	*P*-value	Cor	*P*-value
**Activated B cell**				
GNG7	-0.526	0.000E+00	-0.542	**4.598E-39**
MICAL3	0.340	2.102E-15	0.344	**3.717E-15**
CLEC9A	-0.292	1.334E-11	-0.300	**1.108E-11**
BLK	-0.233	9.074E-08	-0.255	**9.844E-09**
HLA-DOB	-0.226	2.137E-07	-0.240	**6.752E-08**
MS4A1	-0.189	1.720E-05	-0.207	**3.462E-06**
SPIB	-0.167	1.407E-04	-0.181	**5.204E-05**
BACH2	0.157	3.387E-04	0.178	**6.919E-05**
CD79B	-0.159	3.009E-04	-0.173	**1.157E-04**
CR2	-0.174	7.130E-05	-0.170	**1.548E-04**
CLECL1	-0.163	1.945E-04	-0.166	**2.140E-04**
CLEC17A	-0.128	3.637E-03	-0.143	**1.490E-03**
AKNA	-0.135	2.078E-03	-0.142	**1.535E-03**
CD27	-0.132	2.703E-03	-0.139	**1.934E-03**
TNFRSF17	-0.140	1.499E-03	-0.139	**1.990E-03**
ARHGAP25	-0.122	5.639E-03	-0.126	**5.098E-03**
CD19	-0.107	1.507E-02	-0.119	**8.208E-03**
FCRL2	-0.097	2.726E-02	-0.095	**3.513E-02**
PNOC	-0.072	1.046E-01	-0.069	1.235E-01
TCL1A	-0.060	1.770E-01	-0.058	1.986E-01
CCL21	0.023	6.025E-01	0.047	2.948E-01
ADAM28	0.026	5.587E-01	0.029	5.238E-01
CD38	-0.001	9.755E-01	0.014	7.587E-01
CD180	-0.022	6.135E-01	-0.003	9.491E-01
**Immature B cell**				
TXNIP	-0.338	4.403E-15	-0.340	**8.067E-15**
CD22	-0.257	3.589E-09	-0.285	**1.217E-10**
KIAA0226	0.278	1.540E-10	0.278	**3.555E-10**
FCRL1	-0.232	9.644E-08	-0.257	**7.175E-09**
STAP1	-0.199	5.553E-06	-0.223	**5.747E-07**
FAM129C	-0.190	1.495E-05	-0.203	**5.791E-06**
SP100	0.142	1.190E-03	0.155	**5.319E-04**
FCRLA	-0.134	2.245E-03	-0.143	**1.401E-03**
HLA-DQA1	-0.131	2.895E-03	-0.137	**2.230E-03**
HDAC9	0.096	2.981E-02	0.087	5.282E-02
FCRL3	-0.084	5.697E-02	-0.082	6.954E-02
CYBB	0.027	5.347E-01	0.054	2.292E-01
TAGAP	-0.047	2.859E-01	-0.044	3.262E-01
ZCCHC2	0.038	3.895E-01	0.042	3.524E-01
HVCN1	-0.052	2.396E-01	-0.038	3.953E-01
NCF1	0.020	6.561E-01	0.037	4.110E-01
FCRL5	0.017	7.015E-01	0.027	5.563E-01
NCF1B	-0.028	5.302E-01	-0.019	6.759E-01
P2RY10	0.000	9.974E-01	0.018	6.836E-01
**Memory B cell**				
CCNA2	0.901	0.000E+00	0.902	**3.289E-181**
CDKN3	0.824	0.000E+00	0.828	**1.267E-125**
FCER1A	-0.490	2.167E-32	-0.498	**2.662E-32**
ENPP1	0.326	4.798E-14	0.334	**2.405E-14**
MYC	0.264	1.275E-09	0.263	**2.929E-09**
SOX5	-0.092	3.720E-02	-0.103	**2.249E-02**
SORL1	-0.096	2.959E-02	-0.099	**2.866E-02**
RUNX2	0.083	5.829E-02	0.092	**4.067E-02**
AICDA	0.045	3.095E-01	0.069	1.252E-01
FCRL4	-0.042	3.400E-01	-0.050	2.667E-01
CLCN5	-0.038	3.852E-01	-0.037	4.185E-01
STAT5B	-0.032	4.666E-01	-0.036	4.298E-01
TLR9	0.013	7.642E-01	0.030	5.049E-01
STAT5A	-0.033	4.490E-01	-0.021	6.418E-01

Cor: Correlation coefficient; LUAD: Lung adenocarcinoma; Bold values indicate *p*-value < 0.05.

## Abbreviations

*RRM2*: ribonucleoside-diphosphate reductase subunit M2
LUAD: lung adenocarcinoma
NSCLC: non-small cell lung cancer
TCGA: The Cancer Genome Atlas
GEO: Gene Expression Omnibus
CPTAC: Clinical Proteomic Tumor Analysis Consortium
KEGG: Kyoto Encyclopedia of Genes and Genomes
GO: Gene Ontology
BP: Biological Process
GSEA: Gene Set Enrichment Analysis
GEPIA: The Gene Expression Profiling Interactive Analysis
TIMER: The Tumor Immune Estimation Resource
TILs: tumor-infiltrating lymphocytes
MHC: major histocompatibility complex
