# Supplementary material for: Independent prognostic implications of RRM2 in lung adenocarcinoma
Source: J Cancer. 2020 Oct 17;11(23):7009–22. doi: 10.7150/jca.47895 (PMC7592001; doi:10.7150/jca.47895)
Supplement: Supplementary file 1 — Supplementary figures and tables. [file jcav11p7009s1.pdf]

Figure S1

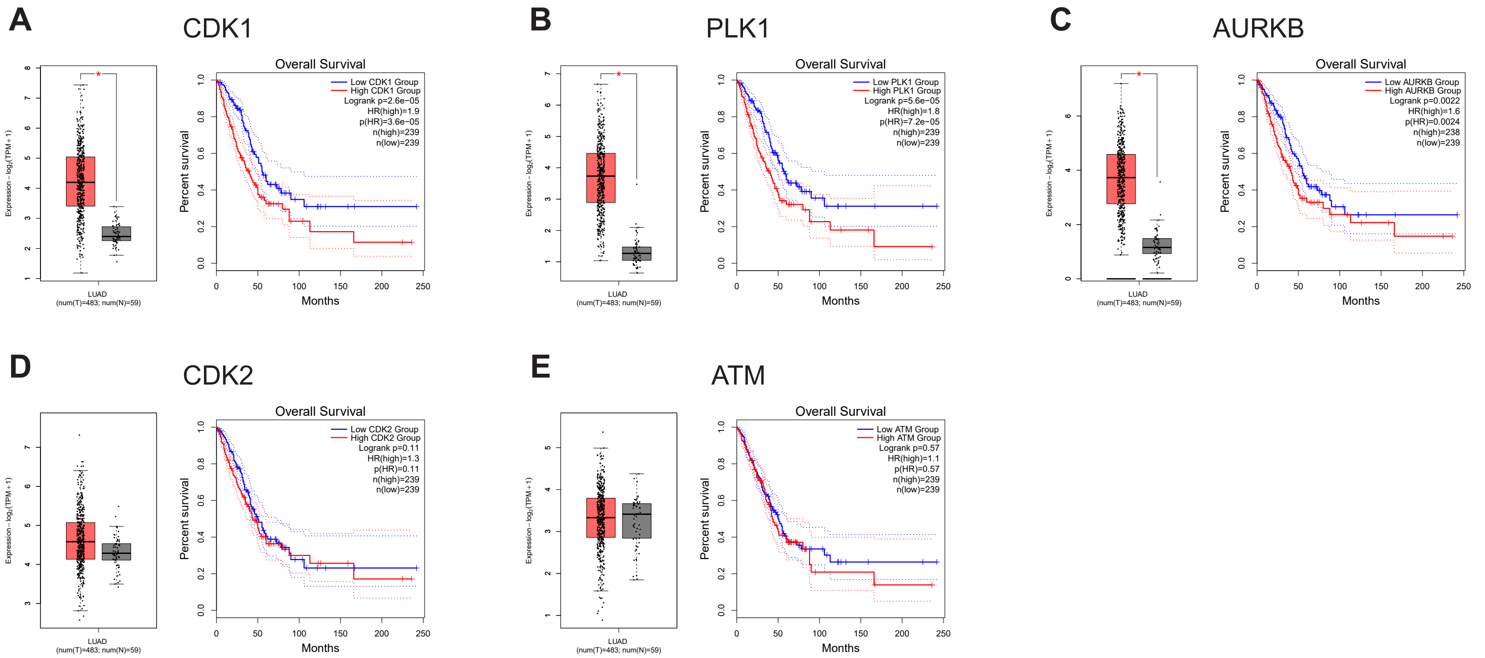

A
CDK1
Overall Survival
Low CDK1 Group
High CDK1 Group
Logrank p=2.6e−05
HR(high)=1.9
p(HR)=3.6e−05
n(high)=239
n(low)=239
Percent survival
Months
LUAD
(num(T)=483; num(N)=59)
B
PLK1
Overall Survival
Low PLK1 Group
High PLK1 Group
Logrank p=5.6e−05
HR(high)=1.8
p(HR)=7.2e−05
n(high)=239
n(low)=239
C
AURKB
Overall Survival
Low AURKB Group
High AURKB Group
Logrank p=0.0022
HR(high)=1.6
p(HR)=0.0024
n(high)=238
n(low)=239
D
CDK2
Overall Survival
Low CDK2 Group
High CDK2 Group
Logrank p=0.11
HR(high)=1.3
p(HR)=0.11
n(high)=239
n(low)=239
E
ATM
Overall Survival
Low ATM Group
High ATM Group
Logrank p=0.57
HR(high)=1.1
p(HR)=0.57
n(high)=239
n(low)=239

Figure S2

A

Survival heatmap of B cell markers positively correlated with RRM2

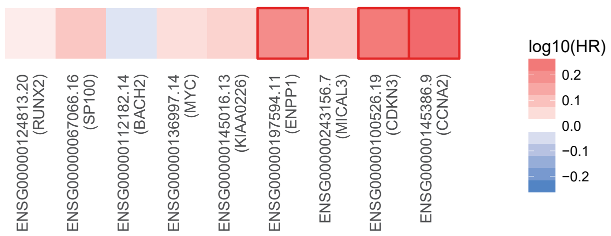

B

Survival heatmap of B cell markers negatively correlated with RRM2

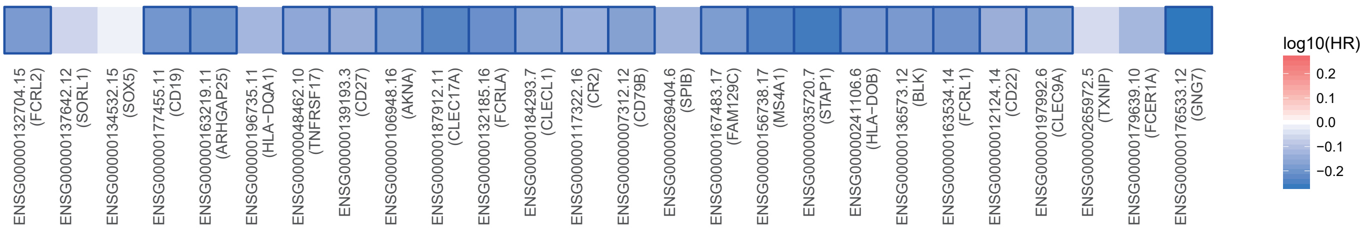

Table S1. *RRM2* co-expressed genes.

Query	Correlation coefficient	P-value	FDR (BH)
A1BG	-0.0316	4.74E-01	5.40E-01
A1CF	0.086186	5.06E-02	7.46E-02
A2BP1	-0.13047	3.01E-03	5.61E-03
A2LD1	-0.02937	5.06E-01	5.70E-01
A2ML1	0.101783	2.09E-02	3.33E-02
A2M	-0.48817	3.40E-32	1.00E-30
A4GALT	0.142791	1.16E-03	2.30E-03
A4GNT	-0.18635	2.08E-05	5.34E-05
AAA1	0.036435	4.09E-01	4.76E-01
AAAS	0.134573	2.21E-03	4.20E-03
AACSL	-0.09888	2.48E-02	3.90E-02
AACS	0.322311	6.50E-14	4.29E-13
AADACL2	-0.02885	5.14E-01	5.77E-01
AADACL3	0.102615	1.98E-02	3.18E-02
AADACL4	-0.11582	8.52E-03	1.46E-02
AADAC	-0.23809	4.53E-08	1.60E-07
AADAT	-0.17668	5.54E-05	1.34E-04
AAGAB	0.356692	6.76E-17	5.95E-16
AAK1	-0.04779	2.79E-01	3.41E-01
AAMP	0.047406	2.83E-01	3.45E-01
AANAT	0.001069	9.81E-01	9.85E-01
AARS2	0.031148	4.81E-01	5.46E-01
AARSD1	0.123703	4.93E-03	8.88E-03
AARS	0.091783	3.73E-02	5.65E-02
AASDHPPT	0.128745	3.42E-03	6.32E-03
AASDH	-0.07938	7.19E-02	1.03E-01
AASS	-0.3325	9.26E-15	6.60E-14
AATF	0.3225	6.27E-14	4.15E-13
AATK	-0.34451	8.49E-16	6.72E-15
ABAT	-0.38862	5.18E-20	6.05E-19
ABCA10	-0.40972	2.88E-22	4.05E-21
ABCA11P	0.079943	6.99E-02	1.00E-01
ABCA12	0.172648	8.21E-05	1.94E-04
ABCA13	-0.20287	3.47E-06	9.86E-06
ABCA17P	-0.00583	8.95E-01	9.15E-01
ABCA1	-0.00335	9.40E-01	9.53E-01
ABCA2	-0.23239	9.59E-08	3.28E-07
ABCA3	-0.47785	9.74E-31	2.64E-29
ABCA4	-0.09957	2.38E-02	3.76E-02
ABCA5	-0.24222	2.60E-08	9.44E-08
ABCA6	-0.35907	4.06E-17	3.68E-16
ABCA7	-0.18772	1.80E-05	4.67E-05

ABCA8	-0.55129	2.79E-42	1.44E-40
ABCA9	-0.3459	6.41E-16	5.13E-15
ABCB10	0.053028	2.30E-01	2.88E-01
ABCB11	0.026768	5.44E-01	6.07E-01
ABCB1	-0.15577	3.88E-04	8.29E-04
ABCB4	-0.13932	1.53E-03	2.98E-03
ABCB5	-0.04535	3.04E-01	3.68E-01
ABCB6	0.10085	2.21E-02	3.51E-02
ABCB7	0.155918	3.83E-04	8.19E-04
ABCB8	-0.03334	4.50E-01	5.17E-01
ABCB9	0.133348	2.43E-03	4.58E-03
ABCC10	-0.1357	2.03E-03	3.88E-03
ABCC11	0.038591	3.82E-01	4.49E-01
ABCC12	-0.31203	4.30E-13	2.62E-12
ABCC13	-0.17342	7.62E-05	1.81E-04
ABCC1	0.107398	1.48E-02	2.43E-02
ABCC2	0.280616	8.97E-11	4.28E-10
ABCC3	-0.33497	5.72E-15	4.16E-14
ABCC4	-0.28283	6.29E-11	3.04E-10
ABCC5	0.028173	5.24E-01	5.87E-01
ABCC6P1	-0.32725	2.55E-14	1.75E-13
ABCC6P2	-0.42354	7.80E-24	1.26E-22
ABCC6	-0.5498	5.12E-42	2.60E-40
ABCC8	-0.14064	1.38E-03	2.70E-03
ABCC9	-0.27581	1.92E-10	8.89E-10
ABCD1	0.148968	6.96E-04	1.43E-03
ABCD2	-0.0346	4.33E-01	5.00E-01
ABCD3	-0.13778	1.72E-03	3.34E-03
ABCD4	-0.20737	2.07E-06	6.04E-06
ABCE1	0.535919	1.26E-39	5.70E-38
ABCF1	0.187553	1.84E-05	4.75E-05
ABCF2	0.44702	1.15E-26	2.30E-25
ABCF3	0.241892	2.72E-08	9.85E-08
ABCG1	-0.03555	4.21E-01	4.87E-01
ABCG2	-0.0418	3.44E-01	4.10E-01
ABCG4	0.087012	4.84E-02	7.16E-02
ABCG5	0.024029	5.86E-01	6.45E-01
ABCG8	0.119458	6.65E-03	1.17E-02
ABHD10	0.29872	4.48E-12	2.46E-11
ABHD11	-0.01299	7.69E-01	8.08E-01
ABHD12B	-0.12424	4.75E-03	8.57E-03
ABHD12	0.055022	2.13E-01	2.69E-01
ABHD13	-0.05985	1.75E-01	2.27E-01
ABHD14A	-0.48269	2.05E-31	5.75E-30

ABHD14B	-0.42222	1.11E-23	1.77E-22
ABHD15	-0.13622	1.95E-03	3.74E-03
ABHD1	0.066117	1.34E-01	1.79E-01
ABHD2	-0.16025	2.61E-04	5.72E-04
ABHD3	0.244032	2.03E-08	7.46E-08
ABHD4	0.024755	5.75E-01	6.35E-01
ABHD5	0.175477	6.24E-05	1.50E-04
ABHD6	-0.0745	9.12E-02	1.27E-01
ABHD8	0.008477	8.48E-01	8.75E-01
ABI1	0.126038	4.17E-03	7.59E-03
ABI2	0.066412	1.32E-01	1.77E-01
ABI3BP	-0.40579	7.80E-22	1.06E-20
ABI3	-0.08963	4.20E-02	6.30E-02
ABL1	-0.07884	7.38E-02	1.05E-01
ABL2	0.035757	4.18E-01	4.84E-01
ABLIM1	-0.22321	3.09E-07	9.99E-07
ABLIM2	-0.29386	1.02E-11	5.39E-11
ABLIM3	-0.07635	8.34E-02	1.17E-01
ABO	-0.44037	7.66E-26	1.45E-24
ABP1	-0.17275	8.13E-05	1.92E-04
ABRA	-0.2502	8.62E-09	3.31E-08
ABR	-0.20352	3.22E-06	9.19E-06
ABT1	0.148741	7.09E-04	1.46E-03
ABTB1	-0.45668	6.74E-28	1.49E-26
ABTB2	0.080403	6.83E-02	9.80E-02
ACAA1	-0.39497	1.13E-20	1.40E-19
ACAA2	0.043785	3.21E-01	3.86E-01
ACACA	0.011028	8.03E-01	8.37E-01
ACACB	-0.33586	4.80E-15	3.52E-14
ACAD10	-0.22853	1.58E-07	5.27E-07
ACAD11	-0.1371	1.82E-03	3.51E-03
ACAD8	-0.38178	2.57E-19	2.83E-18
ACAD9	0.238648	4.20E-08	1.49E-07
ACADL	-0.34865	3.64E-16	2.97E-15
ACADM	-0.03099	4.83E-01	5.48E-01
ACADSB	-0.45742	5.40E-28	1.21E-26
ACADS	-0.35309	1.45E-16	1.23E-15
ACADVL	-0.27986	1.01E-10	4.81E-10
ACAN	0.145825	9.03E-04	1.83E-03
ACAP1	-0.13414	2.28E-03	4.33E-03
ACAP2	0.265444	9.40E-10	4.03E-09
ACAP3	-0.17384	7.32E-05	1.74E-04
ACAT1	-0.27319	2.88E-10	1.32E-09
ACAT2	0.358492	4.60E-17	4.15E-16

ACBD3	-0.02431	5.82E-01	6.41E-01
ACBD4	-0.34461	8.33E-16	6.59E-15
ACBD5	0.141385	1.30E-03	2.56E-03
ACBD6	0.100911	2.20E-02	3.49E-02
ACBD7	0.254553	4.64E-09	1.84E-08
ACCN1	-0.05915	1.80E-01	2.33E-01
ACCN2	0.107396	1.48E-02	2.43E-02
ACCN3	-0.08794	4.61E-02	6.85E-02
ACCN4	-0.04322	3.28E-01	3.93E-01
ACCN5	-0.05188	2.40E-01	2.99E-01
ACCSL	-0.07262	9.97E-02	1.37E-01
ACCS	-0.45013	4.65E-27	9.59E-26
ACD	0.117262	7.73E-03	1.34E-02
ACE2	-0.29142	1.54E-11	7.94E-11
ACER1	-0.16745	1.35E-04	3.08E-04
ACER2	-0.09079	3.94E-02	5.94E-02
ACER3	0.225592	2.29E-07	7.53E-07
ACE	-0.18703	1.94E-05	5.00E-05
ACHE	-0.15612	3.76E-04	8.06E-04
ACIN1	-0.09617	2.91E-02	4.50E-02
ACLY	0.420555	1.73E-23	2.70E-22
ACMSD	-0.10867	1.36E-02	2.26E-02
ACN9	0.253478	5.41E-09	2.13E-08
ACO1	0.008595	8.46E-01	8.74E-01
ACO2	0.040145	3.63E-01	4.30E-01
ACOT11	0.081847	6.35E-02	9.17E-02
ACOT12	0.088927	4.37E-02	6.52E-02
ACOT13	0.068975	1.18E-01	1.60E-01
ACOT1	-0.21493	8.50E-07	2.60E-06
ACOT2	-0.11874	6.98E-03	1.22E-02
ACOT4	0.108507	1.38E-02	2.28E-02
ACOT6	0.142851	1.15E-03	2.29E-03
ACOT7	0.441927	4.93E-26	9.51E-25
ACOT8	0.022106	6.17E-01	6.73E-01
ACOT9	0.280532	9.09E-11	4.33E-10
ACOX1	-0.13525	2.10E-03	4.00E-03
ACOX2	-0.37523	1.16E-18	1.19E-17
ACOX3	-0.27777	1.41E-10	6.61E-10
ACOXL	-0.4884	3.15E-32	9.34E-31
ACP1	0.471564	7.13E-30	1.82E-28
ACP2	0.097838	2.64E-02	4.12E-02
ACP5	-0.13593	1.99E-03	3.82E-03
ACP6	-0.07739	7.93E-02	1.12E-01
ACPL2	0.110512	1.21E-02	2.02E-02

ACPP	-0.04215	3.40E-01	4.05E-01
ACPT	0.090015	4.12E-02	6.18E-02
ACRBP	0.051824	2.40E-01	3.00E-01
ACRC	-0.03432	4.37E-01	5.04E-01
ACRV1	0.416848	4.57E-23	6.89E-22
ACR	-0.07669	8.21E-02	1.16E-01
ACSBG1	-0.36146	2.43E-17	2.25E-16
ACSBG2	-0.05021	2.55E-01	3.16E-01
ACSF2	-0.14118	1.32E-03	2.60E-03
ACSF3	-0.2967	6.32E-12	3.41E-11
ACSL1	-0.13267	2.55E-03	4.81E-03
ACSL3	0.095052	3.10E-02	4.78E-02
ACSL4	0.108818	1.35E-02	2.24E-02
ACSL5	-0.43856	1.28E-25	2.38E-24
ACSL6	-0.01842	6.77E-01	7.27E-01
ACSM1	-0.3828	2.03E-19	2.26E-18
ACSM2A	-0.14955	6.62E-04	1.37E-03
ACSM2B	-0.09936	2.41E-02	3.80E-02
ACSM3	-0.43775	1.60E-25	2.94E-24
ACSM4	0.073757	9.45E-02	1.31E-01
ACSM5	-0.30923	7.11E-13	4.24E-12
ACSS1	-0.53072	9.28E-39	4.00E-37
ACSS2	-0.28056	9.05E-11	4.31E-10
ACSS3	-0.02948	5.04E-01	5.69E-01
ACTA1	0.045211	3.06E-01	3.70E-01
ACTA2	-0.07346	9.58E-02	1.33E-01
ACTBL2	0.020453	6.43E-01	6.98E-01
ACTB	0.185652	2.24E-05	5.72E-05
ACTC1	-0.08369	5.77E-02	8.40E-02
ACTG1	0.3263	3.06E-14	2.08E-13
ACTG2	-0.11782	7.44E-03	1.29E-02
ACTL6A	0.556985	2.67E-43	1.44E-41
ACTL6B	0.125158	4.45E-03	8.05E-03
ACTL7A	-0.0675	1.26E-01	1.69E-01
ACTL7B	-0.11425	9.46E-03	1.61E-02
ACTL8	0.240448	3.30E-08	1.19E-07
ACTL9	0.118606	7.05E-03	1.23E-02
ACTN1	0.281269	8.08E-11	3.87E-10
ACTN2	-0.35488	9.91E-17	8.59E-16
ACTN3	0.118646	7.03E-03	1.23E-02
ACTN4	0.118104	7.29E-03	1.27E-02
ACTR10	0.051574	2.43E-01	3.03E-01
ACTR1A	-0.0419	3.43E-01	4.08E-01
ACTR1B	-0.17912	4.35E-05	1.07E-04

ACTR2	0.390745	3.12E-20	3.72E-19
ACTR3B	0.165452	1.62E-04	3.67E-04
ACTR3C	-0.09944	2.40E-02	3.78E-02
ACTR3	0.552274	1.87E-42	9.73E-41
ACTR5	0.117767	7.46E-03	1.30E-02
ACTR6	0.311054	5.13E-13	3.11E-12
ACTR8	-0.01184	7.89E-01	8.25E-01
ACTRT1	0.005723	8.97E-01	9.17E-01
ACTRT2	-0.01784	6.86E-01	7.36E-01
ACVR1B	-0.18939	1.51E-05	3.96E-05
ACVR1C	0.231728	1.05E-07	3.56E-07
ACVR1	-0.09742	2.71E-02	4.21E-02
ACVR2A	-0.31414	2.94E-13	1.83E-12
ACVR2B	-0.01317	7.66E-01	8.05E-01
ACVRL1	-0.22693	1.94E-07	6.41E-07
ACY1	-0.04948	2.62E-01	3.24E-01
ACY3	0.010322	8.15E-01	8.48E-01
ACYP1	0.300988	3.03E-12	1.69E-11
ACYP2	-0.20028	4.64E-06	1.30E-05
ADAD1	0.090146	4.09E-02	6.14E-02
ADAD2	-0.02031	6.46E-01	7.00E-01
ADAL	-0.0013	9.77E-01	9.82E-01
ADAM10	0.258002	2.82E-09	1.15E-08
ADAM11	0.125925	4.21E-03	7.65E-03
ADAM12	0.414918	7.56E-23	1.11E-21
ADAM15	-0.06724	1.28E-01	1.71E-01
ADAM17	0.464517	6.32E-29	1.51E-27
ADAM18	0.082719	6.07E-02	8.80E-02
ADAM19	0.202071	3.79E-06	1.07E-05
ADAM20	-0.10178	2.09E-02	3.33E-02
ADAM21P1	0.215628	7.82E-07	2.40E-06
ADAM21	0.249648	9.31E-09	3.57E-08
ADAM22	0.222074	3.56E-07	1.14E-06
ADAM23	0.098686	2.51E-02	3.94E-02
ADAM28	-0.01416	7.48E-01	7.90E-01
ADAM29	-0.14652	8.53E-04	1.73E-03
ADAM2	0.131142	2.87E-03	5.35E-03
ADAM30	-0.01444	7.44E-01	7.87E-01
ADAM32	0.168735	1.19E-04	2.75E-04
ADAM33	-0.25402	5.01E-09	1.98E-08
ADAM3A	0.025128	5.69E-01	6.29E-01
ADAM5P	0.095006	3.11E-02	4.79E-02
ADAM6	-0.13807	1.68E-03	3.27E-03
ADAM7	0.010694	8.09E-01	8.42E-01

ADAM8	-0.03415	4.39E-01	5.06E-01
ADAM9	0.191124	1.26E-05	3.33E-05
ADAMDEC1	0.273808	2.62E-10	1.20E-09
ADAMTS10	-0.35032	2.57E-16	2.14E-15
ADAMTS12	0.234888	6.92E-08	2.40E-07
ADAMTS13	-0.25199	6.69E-09	2.61E-08
ADAMTS14	0.106882	1.52E-02	2.50E-02
ADAMTS15	-0.15442	4.37E-04	9.25E-04
ADAMTS16	-0.0032	9.42E-01	9.55E-01
ADAMTS17	-0.38304	1.92E-19	2.14E-18
ADAMTS18	0.072657	9.96E-02	1.37E-01
ADAMTS19	0.003232	9.42E-01	9.54E-01
ADAMTS1	-0.18221	3.18E-05	7.95E-05
ADAMTS20	0.32199	6.90E-14	4.54E-13
ADAMTS2	0.089014	4.35E-02	6.49E-02
ADAMTS3	0.16171	2.29E-04	5.07E-04
ADAMTS4	0.381504	2.75E-19	3.01E-18
ADAMTS5	0.264771	1.04E-09	4.43E-09
ADAMTS6	0.205678	2.52E-06	7.26E-06
ADAMTS7	0.066601	1.31E-01	1.76E-01
ADAMTS8	-0.57082	7.35E-46	4.45E-44
ADAMTS9	0.035274	4.24E-01	4.90E-01
ADAMTSL1	-0.03986	3.67E-01	4.33E-01
ADAMTSL2	-0.47149	7.29E-30	1.85E-28
ADAMTSL3	-0.45248	2.34E-27	4.98E-26
ADAMTSL4	-0.0865	4.98E-02	7.34E-02
ADAMTSL5	0.040898	3.54E-01	4.20E-01
ADAP1	-0.08989	4.14E-02	6.22E-02
ADAP2	0.041595	3.46E-01	4.12E-01
ADARB1	-0.33265	9.00E-15	6.42E-14
ADARB2	-0.12771	3.69E-03	6.78E-03
ADAR	0.184763	2.45E-05	6.23E-05
ADAT1	0.088308	4.52E-02	6.73E-02
ADAT2	-0.08445	5.55E-02	8.10E-02
ADAT3	-0.0099	8.23E-01	8.54E-01
ADA	0.459851	2.61E-28	5.93E-27
ADCK1	-0.17369	7.43E-05	1.76E-04
ADCK2	0.132032	2.68E-03	5.03E-03
ADCK4	-0.16674	1.44E-04	3.27E-04
ADCK5	-0.08468	5.48E-02	8.01E-02
ADCY10	-0.01424	7.47E-01	7.90E-01
ADCY1	-0.09384	3.33E-02	5.09E-02
ADCY2	-0.3823	2.28E-19	2.52E-18
ADCY3	0.316259	2.00E-13	1.26E-12

ADCY4	-0.28719	3.09E-11	1.55E-10
ADCY5	-0.09571	2.99E-02	4.61E-02
ADCY6	-0.31354	3.28E-13	2.03E-12
ADCY7	-0.2597	2.20E-09	9.05E-09
ADCY8	-0.09888	2.48E-02	3.90E-02
ADCY9	-0.48056	4.08E-31	1.13E-29
ADCYAP1R1	-0.15632	3.70E-04	7.93E-04
ADCYAP1	-0.12473	4.59E-03	8.29E-03
ADC	-0.35911	4.03E-17	3.65E-16
ADD1	-0.28435	4.92E-11	2.40E-10
ADD2	-0.05349	2.26E-01	2.83E-01
ADD3	0.030787	4.86E-01	5.51E-01
ADH1A	-0.5241	1.12E-37	4.49E-36
ADH1B	-0.54715	1.49E-41	7.35E-40
ADH1C	-0.31014	6.05E-13	3.63E-12
ADH4	0.008104	8.54E-01	8.81E-01
ADH5	-0.07727	7.98E-02	1.13E-01
ADH6	-0.11923	6.75E-03	1.18E-02
ADH7	-0.1803	3.86E-05	9.53E-05
ADHFE1	-0.51889	7.70E-37	2.94E-35
ADI1	-0.18644	2.06E-05	5.29E-05
ADIG	0.002653	9.52E-01	9.62E-01
ADIPOQ	0.001252	9.77E-01	9.83E-01
ADIPOR1	-0.23347	8.34E-08	2.87E-07
ADIPOR2	0.313902	3.07E-13	1.90E-12
ADK	-0.03448	4.35E-01	5.01E-01
ADM2	-0.01705	6.99E-01	7.48E-01
ADM	0.368568	5.14E-18	5.00E-17
ADNP2	0.237031	5.22E-08	1.83E-07
ADNP	0.200309	4.62E-06	1.30E-05
ADORA1	0.023639	5.92E-01	6.51E-01
ADORA2A	-0.14444	1.01E-03	2.03E-03
ADORA2B	-0.08082	6.69E-02	9.62E-02
ADORA3	-0.09656	2.84E-02	4.41E-02
ADO	0.121483	5.77E-03	1.03E-02
ADPGK	0.063251	1.52E-01	2.00E-01
ADPRHL1	-0.15952	2.79E-04	6.09E-04
ADPRHL2	-0.0275	5.33E-01	5.97E-01
ADPRH	-0.23806	4.55E-08	1.61E-07
ADRA1A	-0.40178	2.12E-21	2.79E-20
ADRA1B	-0.16628	1.50E-04	3.41E-04
ADRA1D	-0.27987	1.01E-10	4.80E-10
ADRA2A	-0.38247	2.19E-19	2.43E-18
ADRA2B	-0.10888	1.34E-02	2.23E-02

ADRA2C	-0.1815	3.42E-05	8.51E-05
ADRB1	-0.40852	3.91E-22	5.45E-21
ADRB2	-0.36428	1.32E-17	1.24E-16
ADRB3	-0.28059	9.00E-11	4.29E-10
ADRBK1	0.119198	6.77E-03	1.18E-02
ADRBK2	-0.12727	3.82E-03	6.98E-03
ADRM1	0.259337	2.32E-09	9.51E-09
ADSL	0.342529	1.27E-15	9.79E-15
ADSSL1	-0.00874	8.43E-01	8.72E-01
ADSS	-0.0031	9.44E-01	9.56E-01
AEBP1	-0.1099	1.26E-02	2.10E-02
AEBP2	0.151974	5.39E-04	1.13E-03
AEN	0.15686	3.53E-04	7.59E-04
AES	-0.19935	5.15E-06	1.43E-05
AFAP1L1	-0.02404	5.86E-01	6.45E-01
AFAP1L2	0.053886	2.22E-01	2.80E-01
AFAP1	0.09375	3.34E-02	5.11E-02
AFARP1	0.143926	1.06E-03	2.11E-03
AFF1	-0.15638	3.68E-04	7.89E-04
AFF2	-0.21112	1.34E-06	3.99E-06
AFF3	-0.49863	1.01E-33	3.23E-32
AFF4	-0.13239	2.61E-03	4.90E-03
AFG3L1	-0.17234	8.46E-05	1.99E-04
AFG3L2	0.148468	7.25E-04	1.49E-03
AFMID	0.049046	2.67E-01	3.28E-01
AFM	0.073824	9.42E-02	1.31E-01
AFP	0.123013	5.18E-03	9.28E-03
AFTPH	-0.05938	1.78E-01	2.31E-01
AG2	-0.34646	5.70E-16	4.59E-15
AGAP11	-0.40391	1.25E-21	1.67E-20
AGAP1	0.025332	5.66E-01	6.26E-01
AGAP2	-0.0806	6.76E-02	9.72E-02
AGAP3	-0.27907	1.15E-10	5.42E-10
AGAP4	-0.21797	5.89E-07	1.84E-06
AGAP5	-0.36107	2.65E-17	2.44E-16
AGAP6	-0.1594	2.81E-04	6.15E-04
AGAP7	-0.15767	3.28E-04	7.10E-04
AGAP8	-0.19662	6.95E-06	1.90E-05
AGA	-0.2652	9.74E-10	4.17E-09
AGBL1	-0.17635	5.73E-05	1.38E-04
AGBL2	-0.19565	7.73E-06	2.10E-05
AGBL3	-0.01956	6.58E-01	7.11E-01
AGBL4	-0.15078	5.97E-04	1.24E-03
AGBL5	0.184356	2.56E-05	6.47E-05

AGER	-0.37833	5.71E-19	6.04E-18
AGFG1	0.365162	1.09E-17	1.03E-16
AGFG2	-0.24103	3.05E-08	1.10E-07
AGGF1	-0.17602	5.91E-05	1.42E-04
AGK	0.150405	6.16E-04	1.28E-03
AGL	-0.01053	8.12E-01	8.44E-01
AGMAT	0.487861	3.77E-32	1.11E-30
AGPAT1	-0.01842	6.77E-01	7.27E-01
AGPAT2	-0.24563	1.63E-08	6.05E-08
AGPAT3	-0.17055	1.00E-04	2.34E-04
AGPAT4	-0.09906	2.46E-02	3.86E-02
AGPAT5	0.311637	4.62E-13	2.81E-12
AGPAT6	-0.01833	6.78E-01	7.29E-01
AGPAT9	0.055067	2.12E-01	2.68E-01
AGPHD1	-0.09091	3.92E-02	5.91E-02
AGPS	0.45377	1.60E-27	3.44E-26
AGR2	-0.1645	1.77E-04	3.98E-04
AGR3	-0.41376	1.02E-22	1.49E-21
AGRN	-0.09434	3.23E-02	4.96E-02
AGRP	-0.24445	1.92E-08	7.07E-08
AGTPBP1	0.033814	4.44E-01	5.10E-01
AGTR1	-0.26962	4.99E-10	2.21E-09
AGTR2	-0.20774	1.98E-06	5.80E-06
AGTRAP	-0.02901	5.11E-01	5.75E-01
AGT	-0.01849	6.76E-01	7.26E-01
AGXT2L1	0.055876	2.06E-01	2.61E-01
AGXT2L2	-0.43486	3.58E-25	6.40E-24
AGXT2	-0.15937	2.82E-04	6.16E-04
AGXT	0.141399	1.29E-03	2.56E-03
AHCTF1	0.13608	1.97E-03	3.77E-03
AHCYL1	-0.12351	5.00E-03	8.98E-03
AHCYL2	-0.42881	1.89E-24	3.20E-23
AHCY	0.387377	6.95E-20	8.01E-19
AHDC1	-0.19417	9.07E-06	2.44E-05
AHI1	-0.18286	2.98E-05	7.47E-05
AHNAK2	0.139292	1.53E-03	2.98E-03
AHNAK	-0.26682	7.63E-10	3.31E-09
AHRR	0.049258	2.65E-01	3.26E-01
AHR	-0.10517	1.70E-02	2.76E-02
AHSA1	0.301602	2.72E-12	1.53E-11
AHSA2	-0.1983	5.78E-06	1.60E-05
AHSG	0.254794	4.49E-09	1.78E-08
AHSP	-0.01763	6.90E-01	7.39E-01
AICDA	0.075446	8.72E-02	1.22E-01

AIDA	-0.08056	6.77E-02	9.73E-02
AIF1L	-0.07599	8.49E-02	1.19E-01
AIF1	-0.05076	2.50E-01	3.11E-01
AIFM1	-0.04561	3.02E-01	3.65E-01
AIFM2	0.043988	3.19E-01	3.84E-01
AIFM3	0.04858	2.71E-01	3.33E-01
AIG1	0.15651	3.64E-04	7.81E-04
AIM1L	0.303065	2.11E-12	1.20E-11
AIM1	0.095645	3.00E-02	4.63E-02
AIM2	0.215687	7.77E-07	2.39E-06
AIMP1	0.238169	4.48E-08	1.59E-07
AIMP2	0.427202	2.92E-24	4.89E-23
AIPL1	0.107732	1.44E-02	2.38E-02
AIP	-0.03121	4.80E-01	5.45E-01
AIRE	-0.11866	7.02E-03	1.22E-02
AJAP1	-0.25764	2.98E-09	1.21E-08
AK1	-0.50406	1.54E-34	5.16E-33
AK2	-0.07955	7.13E-02	1.02E-01
AK3L1	0.529092	1.72E-38	7.27E-37
AK3	-0.15924	2.85E-04	6.23E-04
AK5	0.126848	3.94E-03	7.19E-03
AK7	-0.16595	1.55E-04	3.51E-04
AKAP10	-0.01386	7.54E-01	7.95E-01
AKAP11	-0.16557	1.60E-04	3.63E-04
AKAP12	0.174369	6.95E-05	1.66E-04
AKAP13	-0.46265	1.12E-28	2.61E-27
AKAP14	-0.21042	1.45E-06	4.31E-06
AKAP1	-0.3094	6.90E-13	4.12E-12
AKAP2	0.025933	5.57E-01	6.18E-01
AKAP3	-0.17177	8.94E-05	2.10E-04
AKAP4	-0.02975	5.00E-01	5.65E-01
AKAP5	0.245612	1.63E-08	6.07E-08
AKAP6	-0.2376	4.83E-08	1.70E-07
AKAP7	-0.19919	5.24E-06	1.46E-05
AKAP8L	-0.10871	1.36E-02	2.25E-02
AKAP8	-0.18296	2.95E-05	7.40E-05
AKAP9	-0.13071	2.96E-03	5.52E-03
AKD1	-0.25031	8.48E-09	3.26E-08
AKIRIN1	0.312745	3.78E-13	2.32E-12
AKIRIN2	0.319935	1.01E-13	6.55E-13
AKNAD1	0.106676	1.54E-02	2.53E-02
AKNA	-0.26683	7.62E-10	3.31E-09
AKR1A1	0.010814	8.07E-01	8.40E-01
AKR1B10	0.148004	7.54E-04	1.54E-03

AKR1B15	0.236097	5.90E-08	2.06E-07
AKR1B1	0.201692	3.96E-06	1.12E-05
AKR1C1	-0.0583	1.87E-01	2.40E-01
AKR1C2	0.016865	7.03E-01	7.51E-01
AKR1C3	-0.01354	7.59E-01	8.00E-01
AKR1C4	0.165145	1.67E-04	3.76E-04
AKR1CL1	0.111195	1.16E-02	1.94E-02
AKR1D1	0.126838	3.94E-03	7.19E-03
AKR1E2	0.147208	8.06E-04	1.64E-03
AKR7A2	-0.10749	1.47E-02	2.42E-02
AKR7A3	0.043961	3.19E-01	3.84E-01
AKR7L	-0.04235	3.37E-01	4.03E-01
AKT1S1	0.110769	1.19E-02	1.99E-02
AKT1	-0.11497	9.02E-03	1.54E-02
AKT2	0.164105	1.84E-04	4.12E-04
AKT3	0.062306	1.58E-01	2.07E-01
AKTIP	-0.32728	2.54E-14	1.74E-13
ALAD	-0.43962	9.46E-26	1.78E-24
ALAS1	-0.08995	4.13E-02	6.20E-02
ALAS2	-0.08218	6.24E-02	9.02E-02
ALB	-0.1127	1.05E-02	1.77E-02
ALCAM	-0.28532	4.20E-11	2.06E-10
ALDH16A1	-0.14182	1.25E-03	2.48E-03
ALDH18A1	0.172697	8.17E-05	1.93E-04
ALDH1A1	-0.14847	7.25E-04	1.49E-03
ALDH1A2	-0.04786	2.78E-01	3.40E-01
ALDH1A3	0.047334	2.84E-01	3.46E-01
ALDH1B1	0.339692	2.24E-15	1.69E-14
ALDH1L1	-0.03298	4.55E-01	5.21E-01
ALDH1L2	0.212145	1.19E-06	3.56E-06
ALDH2	-0.51249	7.83E-36	2.83E-34
ALDH3A1	-0.23236	9.64E-08	3.30E-07
ALDH3A2	-0.36279	1.82E-17	1.70E-16
ALDH3B1	-0.42822	2.22E-24	3.74E-23
ALDH3B2	0.16155	2.32E-04	5.13E-04
ALDH4A1	-0.25219	6.50E-09	2.54E-08
ALDH5A1	-0.39125	2.76E-20	3.32E-19
ALDH6A1	-0.35359	1.30E-16	1.11E-15
ALDH7A1	-0.20284	3.48E-06	9.89E-06
ALDH8A1	-0.02707	5.40E-01	6.03E-01
ALDH9A1	-0.24363	2.14E-08	7.85E-08
ALDOA	0.361437	2.45E-17	2.26E-16
ALDOB	-0.25089	7.81E-09	3.02E-08
ALDOC	0.105823	1.63E-02	2.66E-02

ALG10B	0.111443	1.14E-02	1.91E-02
ALG10	0.460165	2.37E-28	5.41E-27
ALG11	0.013107	7.67E-01	8.06E-01
ALG12	-0.22895	1.50E-07	5.02E-07
ALG13	-0.15297	4.95E-04	1.04E-03
ALG14	0.078137	7.65E-02	1.09E-01
ALG1L2	0.066664	1.31E-01	1.75E-01
ALG1L	-0.0035	9.37E-01	9.50E-01
ALG1	-0.34902	3.37E-16	2.77E-15
ALG2	-0.11202	1.10E-02	1.85E-02
ALG3	0.33346	7.69E-15	5.51E-14
ALG5	-0.1695	1.11E-04	2.57E-04
ALG6	0.206593	2.27E-06	6.57E-06
ALG8	0.279729	1.03E-10	4.90E-10
ALG9	-0.01588	7.19E-01	7.66E-01
ALKBH1	0.05494	2.13E-01	2.70E-01
ALKBH2	0.331194	1.20E-14	8.43E-14
ALKBH3	-0.21978	4.72E-07	1.49E-06
ALKBH4	0.116824	7.96E-03	1.37E-02
ALKBH5	-0.11372	9.80E-03	1.67E-02
ALKBH6	0.089666	4.20E-02	6.29E-02
ALKBH7	-0.14633	8.67E-04	1.76E-03
ALKBH8	-0.04815	2.75E-01	3.37E-01
ALK	-0.05133	2.45E-01	3.05E-01
ALLC	0.059523	1.77E-01	2.30E-01
ALMS1P	-0.06297	1.54E-01	2.02E-01
ALMS1	0.027395	5.35E-01	5.98E-01
ALOX12B	0.085552	5.23E-02	7.69E-02
ALOX12P2	-0.073	9.80E-02	1.35E-01
ALOX12	0.027132	5.39E-01	6.01E-01
ALOX15B	-0.45183	2.83E-27	5.99E-26
ALOX15	-0.24532	1.70E-08	6.31E-08
ALOX5AP	-0.10423	1.80E-02	2.91E-02
ALOX5	-0.15601	3.80E-04	8.13E-04
ALOXE3	0.208638	1.79E-06	5.26E-06
ALPI	0.140861	1.35E-03	2.66E-03
ALPK1	-0.22093	4.10E-07	1.31E-06
ALPK2	0.244126	2.00E-08	7.37E-08
ALPK3	0.058356	1.86E-01	2.39E-01
ALPL	-0.40596	7.46E-22	1.01E-20
ALPPL2	-0.29247	1.29E-11	6.73E-11
ALPP	-0.25499	4.36E-09	1.73E-08
ALS2CL	-0.3238	4.91E-14	3.28E-13
ALS2CR11	-0.09354	3.38E-02	5.17E-02

ALS2CR12	-0.30517	1.46E-12	8.45E-12
ALS2CR4	0.34782	4.32E-16	3.51E-15
ALS2CR8	-0.4469	1.19E-26	2.37E-25
ALS2	0.100501	2.26E-02	3.57E-02
ALX1	0.332017	1.02E-14	7.23E-14
ALX3	-0.00039	9.93E-01	9.95E-01
ALX4	0.010702	8.09E-01	8.42E-01
AMAC1L2	-0.13915	1.55E-03	3.01E-03
AMAC1L3	-0.19361	9.65E-06	2.59E-05
AMAC1	0.032825	4.57E-01	5.24E-01
AMACR	-0.05124	2.46E-01	3.06E-01
AMBN	0.101408	2.14E-02	3.40E-02
AMBP	-0.21278	1.10E-06	3.31E-06
AMBRA1	-0.25239	6.33E-09	2.47E-08
AMD1	0.242315	2.56E-08	9.32E-08
AMDHD1	0.098634	2.52E-02	3.95E-02
AMDHD2	-0.00055	9.90E-01	9.93E-01
AMELX	-0.06784	1.24E-01	1.67E-01
AMELY	0.040933	3.54E-01	4.20E-01
AMFR	0.214075	9.42E-07	2.86E-06
AMHR2	-0.22059	4.28E-07	1.36E-06
AMH	0.08135	6.51E-02	9.38E-02
AMICA1	-0.3108	5.37E-13	3.24E-12
AMIGO1	-0.39158	2.55E-20	3.08E-19
AMIGO2	0.114275	9.44E-03	1.61E-02
AMIGO3	-0.06443	1.44E-01	1.91E-01
AMMECR1L	0.060764	1.69E-01	2.19E-01
AMMECR1	0.218199	5.73E-07	1.79E-06
AMN1	0.128975	3.37E-03	6.22E-03
AMN	-0.28819	2.63E-11	1.32E-10
AMOTL1	-0.13652	1.90E-03	3.66E-03
AMOTL2	-0.11337	1.00E-02	1.70E-02
AMOT	-0.27251	3.20E-10	1.45E-09
AMPD1	-0.3015	2.77E-12	1.56E-11
AMPD2	0.029895	4.98E-01	5.63E-01
AMPD3	-0.08027	6.87E-02	9.86E-02
AMPH	-0.00892	8.40E-01	8.69E-01
AMTN	-0.06961	1.15E-01	1.56E-01
AMT	-0.37119	2.87E-18	2.87E-17
AMY1A	-0.36481	1.18E-17	1.11E-16
AMY2A	-0.36646	8.19E-18	7.84E-17
AMY2B	-0.45413	1.44E-27	3.10E-26
AMZ1	0.070072	1.12E-01	1.53E-01
AMZ2P1	-0.11353	9.93E-03	1.69E-02

AMZ2	0.218126	5.78E-07	1.81E-06
ANAPC10	0.211638	1.26E-06	3.77E-06
ANAPC11	0.247003	1.35E-08	5.05E-08
ANAPC13	0.014386	7.45E-01	7.87E-01
ANAPC16	-0.29567	7.54E-12	4.02E-11
ANAPC1	0.358136	4.97E-17	4.45E-16
ANAPC2	-0.15262	5.10E-04	1.07E-03
ANAPC4	-0.05043	2.53E-01	3.14E-01
ANAPC5	0.30646	1.16E-12	6.78E-12
ANAPC7	0.465418	4.79E-29	1.16E-27
ANGEL1	-0.08297	5.99E-02	8.70E-02
ANGEL2	-0.31925	1.15E-13	7.40E-13
ANGPT1	-0.39974	3.52E-21	4.55E-20
ANGPT2	0.253126	5.69E-09	2.24E-08
ANGPT4	-0.2331	8.75E-08	3.01E-07
ANGPTL1	-0.26314	1.33E-09	5.59E-09
ANGPTL2	0.11396	9.64E-03	1.64E-02
ANGPTL3	-0.03499	4.28E-01	4.94E-01
ANGPTL4	0.290437	1.81E-11	9.29E-11
ANGPTL5	-0.44045	7.49E-26	1.42E-24
ANGPTL6	-0.18478	2.45E-05	6.22E-05
ANGPTL7	-0.31014	6.05E-13	3.63E-12
ANG	-0.29238	1.31E-11	6.82E-11
ANK1	-0.02119	6.31E-01	6.87E-01
ANK2	-0.15554	3.96E-04	8.44E-04
ANK3	-0.25501	4.35E-09	1.73E-08
ANKAR	-0.27881	1.19E-10	5.64E-10
ANKDD1A	-0.11785	7.42E-03	1.29E-02
ANKFN1	-0.46071	2.01E-28	4.61E-27
ANKFY1	-0.32276	5.97E-14	3.96E-13
ANKHD1-EIF4EBP3	-0.37268	2.05E-18	2.08E-17
ANKHD1	-0.27165	3.66E-10	1.65E-09
ANKH	-0.07446	9.14E-02	1.27E-01
ANKIB1	0.450025	4.80E-27	9.88E-26
ANKK1	-0.31099	5.19E-13	3.14E-12
ANKLE1	0.309867	6.35E-13	3.81E-12
ANKLE2	0.356264	7.40E-17	6.49E-16
ANKMY1	-0.41931	2.40E-23	3.70E-22
ANKMY2	-0.10048	2.26E-02	3.58E-02
ANKRA2	-0.15989	2.69E-04	5.90E-04
ANKRD10	-0.30555	1.37E-12	7.91E-12
ANKRD11	-0.17673	5.51E-05	1.33E-04
ANKRD12	-0.21207	1.20E-06	3.59E-06
ANKRD13A	-0.09337	3.41E-02	5.21E-02

ANKRD13B	0.11002	1.25E-02	2.08E-02
ANKRD13C	-0.06154	1.63E-01	2.13E-01
ANKRD13D	-0.03413	4.40E-01	5.06E-01
ANKRD16	-0.05158	2.43E-01	3.03E-01
ANKRD17	0.013572	7.59E-01	7.99E-01
ANKRD19	-0.11045	1.21E-02	2.03E-02
ANKRD1	0.044082	3.18E-01	3.83E-01
ANKRD20A3	-0.06485	1.42E-01	1.88E-01
ANKRD20A4	-0.06966	1.14E-01	1.55E-01
ANKRD20B	0.01804	6.83E-01	7.33E-01
ANKRD22	0.092537	3.58E-02	5.44E-02
ANKRD23	-0.08814	4.56E-02	6.78E-02
ANKRD24	-0.29357	1.07E-11	5.64E-11
ANKRD26P1	0.08606	5.10E-02	7.50E-02
ANKRD26	0.067594	1.26E-01	1.69E-01
ANKRD27	0.228859	1.51E-07	5.07E-07
ANKRD28	-0.02802	5.26E-01	5.89E-01
ANKRD29	-0.34999	2.76E-16	2.28E-15
ANKRD2	0.188513	1.66E-05	4.32E-05
ANKRD30A	0.032059	4.68E-01	5.34E-01
ANKRD30B	0.034207	4.39E-01	5.05E-01
ANKRD31	0.076602	8.24E-02	1.16E-01
ANKRD32	0.437793	1.58E-25	2.91E-24
ANKRD33	0.228743	1.54E-07	5.15E-07
ANKRD34A	-0.07191	1.03E-01	1.41E-01
ANKRD34B	0.060204	1.73E-01	2.24E-01
ANKRD34C	-0.1341	2.29E-03	4.34E-03
ANKRD35	-0.18365	2.75E-05	6.92E-05
ANKRD36BP1	-0.01954	6.58E-01	7.11E-01
ANKRD36B	0.050939	2.49E-01	3.09E-01
ANKRD36	-0.03012	4.95E-01	5.60E-01
ANKRD37	-0.05128	2.45E-01	3.05E-01
ANKRD39	0.099951	2.33E-02	3.68E-02
ANKRD40	0.019005	6.67E-01	7.18E-01
ANKRD42	-0.26618	8.41E-10	3.63E-09
ANKRD43	0.026064	5.55E-01	6.16E-01
ANKRD44	-0.31915	1.17E-13	7.53E-13
ANKRD45	-0.25881	2.51E-09	1.02E-08
ANKRD46	-0.06543	1.38E-01	1.84E-01
ANKRD49	0.034303	4.37E-01	5.04E-01
ANKRD50	-0.17707	5.33E-05	1.29E-04
ANKRD52	0.176318	5.74E-05	1.39E-04
ANKRD53	-0.06098	1.67E-01	2.18E-01
ANKRD54	-0.12353	5.00E-03	8.97E-03

ANKRD55	-0.25289	5.89E-09	2.31E-08
ANKRD56	-0.01223	7.82E-01	8.19E-01
ANKRD57	0.082494	6.14E-02	8.89E-02
ANKRD58	-0.06204	1.60E-01	2.09E-01
ANKRD5	0.040955	3.54E-01	4.20E-01
ANKRD6	-0.27674	1.66E-10	7.72E-10
ANKRD7	0.269501	5.08E-10	2.25E-09
ANKRD9	-0.06332	1.51E-01	1.99E-01
ANKS1A	-0.16815	1.26E-04	2.90E-04
ANKS1B	-0.0758	8.57E-02	1.20E-01
ANKS3	-0.30726	1.01E-12	5.93E-12
ANKS4B	-0.02575	5.60E-01	6.21E-01
ANKS6	-0.36521	1.08E-17	1.02E-16
ANKZF1	-0.09657	2.84E-02	4.41E-02
ANLN	0.844985	1.51E-141	6.54E-139
ANO10	0.049119	2.66E-01	3.27E-01
ANO1	-0.03575	4.18E-01	4.84E-01
ANO2	-0.33927	2.44E-15	1.83E-14
ANO3	0.025329	5.66E-01	6.26E-01
ANO4	-0.25184	6.84E-09	2.66E-08
ANO5	-0.20228	3.71E-06	1.05E-05
ANO6	0.16166	2.30E-04	5.09E-04
ANO7	0.176105	5.86E-05	1.41E-04
ANO8	-0.16885	1.18E-04	2.72E-04
ANO9	-0.25203	6.65E-09	2.59E-08
ANP32A	0.083543	5.81E-02	8.46E-02
ANP32B	0.313818	3.12E-13	1.93E-12
ANP32C	0.107987	1.42E-02	2.35E-02
ANP32D	-0.04819	2.75E-01	3.37E-01
ANP32E	0.383576	1.70E-19	1.90E-18
ANPEP	-0.02394	5.88E-01	6.46E-01
ANTXR1	-0.03203	4.68E-01	5.34E-01
ANTXR2	-0.06111	1.66E-01	2.16E-01
ANTXRL	-0.07084	1.08E-01	1.48E-01
ANUBL1	-0.1227	5.30E-03	9.47E-03
ANXA10	0.058781	1.83E-01	2.36E-01
ANXA11	-0.26776	6.63E-10	2.90E-09
ANXA13	0.139567	1.50E-03	2.93E-03
ANXA1	0.049887	2.58E-01	3.20E-01
ANXA2P1	0.112055	1.09E-02	1.84E-02
ANXA2P2	0.093586	3.37E-02	5.15E-02
ANXA2P3	0.066751	1.30E-01	1.75E-01
ANXA2	0.090394	4.03E-02	6.07E-02
ANXA3	-0.06524	1.39E-01	1.85E-01

ANXA4	-0.14531	9.42E-04	1.90E-03
ANXA5	0.028185	5.23E-01	5.87E-01
ANXA6	-0.15966	2.75E-04	6.01E-04
ANXA7	0.158345	3.09E-04	6.71E-04
ANXA8L1	0.07065	1.09E-01	1.49E-01
ANXA8L2	0.030547	4.89E-01	5.54E-01
ANXA8	0.02982	5.00E-01	5.64E-01
ANXA9	-0.10223	2.03E-02	3.25E-02
AOAH	0.002739	9.51E-01	9.61E-01
AOC2	0.167863	1.30E-04	2.97E-04
AOC3	-0.45019	4.57E-27	9.46E-26
AOX1	-0.12869	3.44E-03	6.34E-03
AOX2P	0.193312	9.96E-06	2.66E-05
AP1AR	0.338747	2.71E-15	2.03E-14
AP1B1	-0.00835	8.50E-01	8.77E-01
AP1G1	-0.15559	3.94E-04	8.41E-04
AP1G2	-0.22316	3.11E-07	1.01E-06
AP1M1	-0.11404	9.60E-03	1.63E-02
AP1M2	-0.10522	1.69E-02	2.75E-02
AP1S1	0.223126	3.12E-07	1.01E-06
AP1S2	-0.03677	4.05E-01	4.71E-01
AP1S3	0.281839	7.37E-11	3.55E-10
AP2A1	0.24163	2.81E-08	1.02E-07
AP2A2	-0.41743	3.93E-23	5.96E-22
AP2B1	0.193037	1.03E-05	2.74E-05
AP2M1	0.334721	6.01E-15	4.36E-14
AP2S1	0.409831	2.80E-22	3.94E-21
AP3B1	-0.03862	3.82E-01	4.48E-01
AP3B2	-0.12235	5.43E-03	9.69E-03
AP3D1	0.053727	2.24E-01	2.81E-01
AP3M1	0.085089	5.36E-02	7.86E-02
AP3M2	0.099427	2.40E-02	3.79E-02
AP3S1	0.228009	1.69E-07	5.62E-07
AP3S2	-0.16498	1.69E-04	3.82E-04
AP4B1	-0.14142	1.29E-03	2.55E-03
AP4E1	0.066175	1.34E-01	1.79E-01
AP4M1	0.211813	1.23E-06	3.69E-06
AP4S1	0.051824	2.40E-01	3.00E-01
APAF1	0.190859	1.30E-05	3.41E-05
APBA1	-0.29451	9.16E-12	4.85E-11
APBA2	-0.01543	7.27E-01	7.72E-01
APBA3	0.042819	3.32E-01	3.98E-01
APBB1IP	-0.0933	3.43E-02	5.23E-02
APBB1	-0.33462	6.13E-15	4.44E-14

APBB2	-0.11725	7.73E-03	1.34E-02
APBB3	-0.27252	3.20E-10	1.45E-09
APC2	0.099303	2.42E-02	3.81E-02
APCDD1L	0.40095	2.61E-21	3.40E-20
APCDD1	-0.1868	1.99E-05	5.11E-05
APCS	0.086307	5.03E-02	7.41E-02
APC	-0.25674	3.39E-09	1.36E-08
APEH	-0.10649	1.56E-02	2.56E-02
APEX1	0.274938	2.20E-10	1.01E-09
APEX2	0.366909	7.42E-18	7.12E-17
APH1A	0.078838	7.38E-02	1.05E-01
APH1B	-0.2187	5.39E-07	1.69E-06
API5	0.091933	3.70E-02	5.61E-02
APIP	-0.06535	1.39E-01	1.84E-01
APITD1	-0.03383	4.44E-01	5.10E-01
APLF	0.070787	1.09E-01	1.48E-01
APLNR	-0.13044	3.02E-03	5.62E-03
APLN	0.070145	1.12E-01	1.52E-01
APLP1	0.137927	1.70E-03	3.30E-03
APLP2	-0.38351	1.72E-19	1.93E-18
APOA1BP	0.108892	1.34E-02	2.23E-02
APOA1	-0.16021	2.62E-04	5.74E-04
APOA2	0.15555	3.96E-04	8.43E-04
APOA4	0.074071	9.31E-02	1.29E-01
APOA5	-0.1386	1.62E-03	3.14E-03
APOB48R	-0.22734	1.84E-07	6.09E-07
APOBEC1	0.221419	3.86E-07	1.23E-06
APOBEC2	-0.18509	2.37E-05	6.03E-05
APOBEC3A	0.19497	8.32E-06	2.25E-05
APOBEC3B	0.382565	2.15E-19	2.38E-18
APOBEC3C	-0.0352	4.25E-01	4.91E-01
APOBEC3D	0.111181	1.16E-02	1.94E-02
APOBEC3F	-0.005	9.10E-01	9.28E-01
APOBEC3G	0.106894	1.52E-02	2.50E-02
APOBEC3H	-0.13581	2.01E-03	3.85E-03
APOBEC4	-0.29208	1.38E-11	7.15E-11
APOB	-0.0482	2.75E-01	3.37E-01
APOC1P1	0.108032	1.42E-02	2.34E-02
APOC1	0.042559	3.35E-01	4.01E-01
APOC2	-0.01553	7.25E-01	7.71E-01
APOC3	0.086099	5.08E-02	7.49E-02
APOC4	-0.03849	3.83E-01	4.50E-01
APOD	-0.37052	3.33E-18	3.31E-17
APOE	0.103592	1.87E-02	3.01E-02

APOF	0.156949	3.50E-04	7.53E-04
APOH	-0.26815	6.24E-10	2.74E-09
APOL1	0.1658	1.57E-04	3.56E-04
APOL2	0.192389	1.10E-05	2.93E-05
APOL3	-0.0375	3.96E-01	4.62E-01
APOL4	0.073932	9.37E-02	1.30E-01
APOL5	0.065233	1.39E-01	1.85E-01
APOL6	0.152379	5.21E-04	1.09E-03
APOLD1	0.013156	7.66E-01	8.05E-01
APOM	-0.21083	1.39E-06	4.12E-06
APOOL	0.110297	1.23E-02	2.05E-02
APOO	0.350556	2.45E-16	2.04E-15
APPBP2	-0.00403	9.27E-01	9.42E-01
APPL1	-0.01587	7.19E-01	7.66E-01
APPL2	0.105828	1.63E-02	2.66E-02
APP	0.058673	1.84E-01	2.37E-01
APRT	-0.00398	9.28E-01	9.43E-01
APTX	0.256338	3.59E-09	1.44E-08
AQP10	-0.26986	4.81E-10	2.14E-09
AQP11	0.232633	9.30E-08	3.19E-07
AQP12A	0.113511	9.94E-03	1.69E-02
AQP12B	0.11564	8.62E-03	1.48E-02
AQP1	-0.32152	7.53E-14	4.94E-13
AQP2	-0.35284	1.52E-16	1.29E-15
AQP3	-0.51847	8.99E-37	3.42E-35
AQP4	-0.41256	1.39E-22	2.01E-21
AQP5	-0.39109	2.87E-20	3.44E-19
AQP6	-0.22676	1.98E-07	6.54E-07
AQP7P1	-0.17327	7.73E-05	1.83E-04
AQP7P3	-0.01545	7.26E-01	7.72E-01
AQP7	-0.49789	1.30E-33	4.10E-32
AQP8	-0.10547	1.66E-02	2.71E-02
AQP9	0.209174	1.68E-06	4.96E-06
AQPEP	0.21075	1.40E-06	4.16E-06
AQR	0.146264	8.71E-04	1.77E-03
ARAF	-0.26403	1.16E-09	4.93E-09
ARAP1	-0.3371	3.76E-15	2.78E-14
ARAP2	-0.14336	1.10E-03	2.20E-03
ARAP3	-0.21412	9.38E-07	2.85E-06
ARCN1	0.17367	7.44E-05	1.77E-04
ARC	-0.32284	5.88E-14	3.90E-13
AREG	0.082699	6.07E-02	8.81E-02
ARF1	-0.08628	5.04E-02	7.42E-02
ARF3	0.134528	2.22E-03	4.21E-03

ARF4	0.055885	2.05E-01	2.61E-01
ARF5	0.178801	4.49E-05	1.10E-04
ARF6	0.308634	7.92E-13	4.70E-12
ARFGAP1	0.036394	4.10E-01	4.76E-01
ARFGAP2	-0.37636	8.95E-19	9.31E-18
ARFGAP3	0.084746	5.46E-02	7.99E-02
ARFGEF1	-0.01968	6.56E-01	7.09E-01
ARFGEF2	0.128616	3.46E-03	6.37E-03
ARFIP1	-0.04906	2.66E-01	3.28E-01
ARFIP2	-0.05176	2.41E-01	3.01E-01
ARFRP1	-0.20617	2.38E-06	6.88E-06
ARG1	0.02273	6.07E-01	6.64E-01
ARG2	0.16041	2.57E-04	5.65E-04
ARGFXP2	-0.16692	1.42E-04	3.23E-04
ARGFX	0.042874	3.32E-01	3.97E-01
ARGLU1	-0.26254	1.45E-09	6.08E-09
ARHGAP10	0.082177	6.24E-02	9.03E-02
ARHGAP11A	0.822675	6.50E-128	1.73E-125
ARHGAP11B	0.683858	3.01E-72	3.25E-70
ARHGAP12	-0.09925	2.43E-02	3.82E-02
ARHGAP15	-0.19006	1.41E-05	3.69E-05
ARHGAP17	-0.06963	1.15E-01	1.55E-01
ARHGAP18	-0.07683	8.15E-02	1.15E-01
ARHGAP19	0.008486	8.48E-01	8.75E-01
ARHGAP1	-0.29834	4.78E-12	2.62E-11
ARHGAP20	-0.29596	7.17E-12	3.84E-11
ARHGAP21	0.077894	7.74E-02	1.10E-01
ARHGAP22	0.005412	9.02E-01	9.21E-01
ARHGAP23	-0.29664	6.38E-12	3.44E-11
ARHGAP24	-0.45087	3.74E-27	7.82E-26
ARHGAP25	-0.20759	2.02E-06	5.90E-06
ARHGAP26	-0.1217	5.68E-03	1.01E-02
ARHGAP27	-0.20766	2.00E-06	5.85E-06
ARHGAP28	-0.05889	1.82E-01	2.35E-01
ARHGAP29	-0.02747	5.34E-01	5.97E-01
ARHGAP30	-0.19798	5.99E-06	1.65E-05
ARHGAP31	-0.37466	1.32E-18	1.35E-17
ARHGAP32	-0.0439	3.20E-01	3.85E-01
ARHGAP33	0.105424	1.67E-02	2.72E-02
ARHGAP36	0.132013	2.68E-03	5.03E-03
ARHGAP39	0.016388	7.11E-01	7.58E-01
ARHGAP42	-0.13968	1.48E-03	2.90E-03
ARHGAP4	0.031574	4.75E-01	5.40E-01
ARHGAP5	0.012281	7.81E-01	8.18E-01

ARHGAP6	-0.46278	1.07E-28	2.51E-27
ARHGAP8	-0.24344	2.20E-08	8.05E-08
ARHGAP9	-0.10876	1.35E-02	2.24E-02
ARHGDIA	0.076907	8.12E-02	1.14E-01
ARHGDIB	-0.37306	1.89E-18	1.92E-17
ARHGDIG	-0.03304	4.54E-01	5.21E-01
ARHGEF10L	-0.12227	5.46E-03	9.74E-03
ARHGEF10	-0.20165	3.98E-06	1.12E-05
ARHGEF11	-0.22094	4.10E-07	1.30E-06
ARHGEF12	-0.34788	4.27E-16	3.47E-15
ARHGEF15	-0.41017	2.56E-22	3.62E-21
ARHGEF16	-0.02663	5.47E-01	6.09E-01
ARHGEF17	-0.52759	3.04E-38	1.26E-36
ARHGEF18	-0.05974	1.76E-01	2.28E-01
ARHGEF19	-0.28269	6.43E-11	3.11E-10
ARHGEF1	-0.3444	8.69E-16	6.85E-15
ARHGEF2	-0.42486	5.49E-24	8.97E-23
ARHGEF33	-0.01162	7.92E-01	8.28E-01
ARHGEF35	0.025618	5.62E-01	6.22E-01
ARHGEF37	-0.34381	9.80E-16	7.67E-15
ARHGEF38	-0.20422	2.98E-06	8.52E-06
ARHGEF3	-0.17952	4.18E-05	1.03E-04
ARHGEF4	0.205583	2.55E-06	7.34E-06
ARHGEF5	-0.02312	6.01E-01	6.58E-01
ARHGEF6	-0.22055	4.30E-07	1.36E-06
ARHGEF7	-0.17221	8.57E-05	2.02E-04
ARHGEF9	-0.39922	3.99E-21	5.14E-20
ARID1A	-0.08673	4.92E-02	7.26E-02
ARID1B	-0.14368	1.08E-03	2.15E-03
ARID2	0.00955	8.29E-01	8.59E-01
ARID3A	0.285945	3.79E-11	1.87E-10
ARID3B	0.066695	1.31E-01	1.75E-01
ARID3C	0.243955	2.05E-08	7.54E-08
ARID4A	-0.38042	3.53E-19	3.81E-18
ARID4B	-0.2431	2.30E-08	8.42E-08
ARID5A	0.051896	2.40E-01	2.99E-01
ARID5B	-0.29257	1.27E-11	6.61E-11
ARIH1	-0.06229	1.58E-01	2.07E-01
ARIH2	-0.03637	4.10E-01	4.76E-01
ARL10	0.029602	5.03E-01	5.67E-01
ARL11	0.077479	7.90E-02	1.12E-01
ARL13A	-0.08771	4.66E-02	6.92E-02
ARL13B	0.141416	1.29E-03	2.55E-03
ARL14	0.212186	1.18E-06	3.54E-06

ARL15	-0.23784	4.69E-08	1.65E-07
ARL16	0.074001	9.34E-02	1.30E-01
ARL17A	-0.00897	8.39E-01	8.68E-01
ARL17B	-0.11254	1.06E-02	1.79E-02
ARL1	0.109407	1.30E-02	2.16E-02
ARL2BP	-0.18837	1.69E-05	4.38E-05
ARL2	0.113418	1.00E-02	1.70E-02
ARL3	-0.04842	2.73E-01	3.35E-01
ARL4A	0.248129	1.15E-08	4.35E-08
ARL4C	0.148073	7.50E-04	1.54E-03
ARL4D	0.123599	4.97E-03	8.94E-03
ARL5A	0.192685	1.07E-05	2.84E-05
ARL5B	0.442885	3.76E-26	7.31E-25
ARL5C	-0.04842	2.73E-01	3.35E-01
ARL6IP1	0.487053	4.92E-32	1.43E-30
ARL6IP4	0.040861	3.55E-01	4.21E-01
ARL6IP5	-0.10294	1.95E-02	3.12E-02
ARL6IP6	0.508648	3.08E-35	1.07E-33
ARL6	0.088014	4.59E-02	6.82E-02
ARL8A	-0.26177	1.62E-09	6.77E-09
ARL8B	-0.0893	4.28E-02	6.40E-02
ARL9	0.302409	2.37E-12	1.34E-11
ARMC10	0.199592	5.01E-06	1.40E-05
ARMC1	0.360781	2.82E-17	2.59E-16
ARMC2	-0.22403	2.79E-07	9.07E-07
ARMC3	-0.0878	4.64E-02	6.89E-02
ARMC4	-0.12409	4.80E-03	8.65E-03
ARMC5	-0.33102	1.24E-14	8.69E-14
ARMC6	0.087639	4.68E-02	6.94E-02
ARMC7	-0.1689	1.17E-04	2.71E-04
ARMC8	0.29279	1.22E-11	6.38E-11
ARMC9	-0.17929	4.27E-05	1.05E-04
ARMCX1	-0.09749	2.69E-02	4.20E-02
ARMCX2	-0.13088	2.92E-03	5.45E-03
ARMCX3	-0.15873	2.99E-04	6.50E-04
ARMCX5	-0.14138	1.30E-03	2.56E-03
ARMCX6	-0.09582	2.97E-02	4.59E-02
ARMS2	-0.06749	1.26E-01	1.70E-01
ARNT2	-0.14802	7.53E-04	1.54E-03
ARNTL2	0.562056	3.18E-44	1.79E-42
ARNTL	-0.19175	1.18E-05	3.12E-05
ARNT	-0.20382	3.11E-06	8.90E-06
ARPC1A	0.403803	1.28E-21	1.71E-20
ARPC1B	0.14691	8.26E-04	1.68E-03

ARPC2	0.262947	1.36E-09	5.74E-09
ARPC3	0.353847	1.23E-16	1.06E-15
ARPC4	0.120529	6.17E-03	1.09E-02
ARPC5L	0.129684	3.20E-03	5.92E-03
ARPC5	0.178454	4.65E-05	1.14E-04
ARPM1	-0.0123	7.81E-01	8.18E-01
ARPP19	0.17176	8.95E-05	2.10E-04
ARPP21	0.002749	9.50E-01	9.61E-01
ARR3	0.047236	2.85E-01	3.47E-01
ARRB1	-0.39882	4.40E-21	5.66E-20
ARRB2	-0.22834	1.62E-07	5.39E-07
ARRDC1	-0.20803	1.92E-06	5.63E-06
ARRDC2	-0.30461	1.61E-12	9.28E-12
ARRDC3	-0.28614	3.68E-11	1.82E-10
ARRDC4	-0.22376	2.88E-07	9.36E-07
ARRDC5	-0.07193	1.03E-01	1.41E-01
ARSA	-0.24134	2.93E-08	1.06E-07
ARSB	0.085904	5.14E-02	7.56E-02
ARSD	-0.35043	2.52E-16	2.09E-15
ARSE	-0.21935	4.98E-07	1.57E-06
ARSF	-0.07358	9.53E-02	1.32E-01
ARSG	-0.10334	1.90E-02	3.06E-02
ARSH	0.120815	6.05E-03	1.07E-02
ARSI	0.101588	2.11E-02	3.36E-02
ARSJ	0.097458	2.70E-02	4.21E-02
ARSK	-0.03285	4.57E-01	5.23E-01
ART1	-0.08914	4.32E-02	6.45E-02
ART3	0.132657	2.56E-03	4.81E-03
ART4	-0.3996	3.64E-21	4.69E-20
ART5	-0.07817	7.63E-02	1.08E-01
ARTN	0.309244	7.10E-13	4.24E-12
ARV1	-0.29502	8.41E-12	4.47E-11
ARVCF	-0.27472	2.27E-10	1.05E-09
ARX	-0.18087	3.65E-05	9.04E-05
AR	-0.35818	4.92E-17	4.41E-16
AS3MT	0.03718	4.00E-01	4.66E-01
ASAH1	-0.39712	6.68E-21	8.42E-20
ASAH2B	0.135825	2.01E-03	3.85E-03
ASAH2	0.113693	9.82E-03	1.67E-02
ASAM	0.19157	1.20E-05	3.18E-05
ASAP1IT1	0.002634	9.52E-01	9.62E-01
ASAP1	0.364478	1.26E-17	1.19E-16
ASAP2	0.116333	8.23E-03	1.42E-02
ASAP3	-0.38762	6.55E-20	7.58E-19

ASB10	0.161263	2.38E-04	5.26E-04
ASB11	-0.07981	7.03E-02	1.01E-01
ASB12	-0.19826	5.81E-06	1.60E-05
ASB13	-0.01556	7.25E-01	7.70E-01
ASB14	-0.23554	6.35E-08	2.21E-07
ASB15	0.050052	2.57E-01	3.18E-01
ASB16	-0.20548	2.58E-06	7.42E-06
ASB17	0.02217	6.16E-01	6.72E-01
ASB18	0.063078	1.53E-01	2.01E-01
ASB1	0.09706	2.76E-02	4.29E-02
ASB2	-0.06146	1.64E-01	2.14E-01
ASB3	-0.07849	7.51E-02	1.07E-01
ASB4	-0.11761	7.55E-03	1.31E-02
ASB5	-0.04508	3.07E-01	3.71E-01
ASB6	-0.05062	2.51E-01	3.12E-01
ASB7	0.092598	3.57E-02	5.42E-02
ASB8	-0.04929	2.64E-01	3.26E-01
ASB9	-0.05836	1.86E-01	2.39E-01
ASCC1	0.166938	1.41E-04	3.22E-04
ASCC2	-0.01776	6.88E-01	7.37E-01
ASCC3	0.23885	4.09E-08	1.45E-07
ASCL1	0.026364	5.51E-01	6.12E-01
ASCL2	0.044446	3.14E-01	3.79E-01
ASCL3	-0.16763	1.32E-04	3.03E-04
ASCL4	-0.26464	1.06E-09	4.52E-09
ASF1A	0.084889	5.42E-02	7.94E-02
ASF1B	0.836786	2.67E-136	8.91E-134
ASFMR1	-0.00625	8.88E-01	9.09E-01
ASGR1	0.027185	5.38E-01	6.01E-01
ASGR2	0.131202	2.85E-03	5.33E-03
ASH1L	-0.24251	2.50E-08	9.09E-08
ASH2L	0.104927	1.72E-02	2.79E-02
ASIP	0.041564	3.47E-01	4.12E-01
ASL	-0.07386	9.40E-02	1.30E-01
ASMTL	-0.31777	1.51E-13	9.63E-13
ASMT	0.083436	5.85E-02	8.50E-02
ASNA1	0.145466	9.30E-04	1.88E-03
ASNSD1	0.169286	1.13E-04	2.62E-04
ASNS	0.454802	1.18E-27	2.56E-26
ASPA	-0.40727	5.36E-22	7.37E-21
ASPDH	-0.08974	4.18E-02	6.26E-02
ASPG	-0.31238	4.04E-13	2.47E-12
ASPHD1	0.019544	6.58E-01	7.11E-01
ASPHD2	0.158486	3.05E-04	6.63E-04

ASPH	0.243392	2.21E-08	8.11E-08
ASPM	0.811481	8.94E-122	2.21E-119
ASPN	-0.07094	1.08E-01	1.47E-01
ASPRV1	-0.25464	4.59E-09	1.82E-08
ASPSCR1	-0.086	5.11E-02	7.52E-02
ASRGL1	0.092406	3.60E-02	5.47E-02
ASS1	-0.14799	7.55E-04	1.55E-03
ASTE1	-0.0219	6.20E-01	6.76E-01
ASTL	-0.0501	2.56E-01	3.17E-01
ASTN1	-0.10706	1.51E-02	2.48E-02
ASTN2	-0.18153	3.41E-05	8.48E-05
ASXL1	0.037522	3.95E-01	4.62E-01
ASXL2	0.148247	7.39E-04	1.51E-03
ASXL3	-0.03158	4.75E-01	5.40E-01
ASZ1	-0.07517	8.84E-02	1.23E-01
ATAD1	0.079943	6.99E-02	1.00E-01
ATAD2B	0.037253	3.99E-01	4.65E-01
ATAD2	0.703589	3.84E-78	4.60E-76
ATAD3A	0.273233	2.86E-10	1.31E-09
ATAD3B	0.134312	2.25E-03	4.28E-03
ATAD3C	-0.35211	1.77E-16	1.50E-15
ATAD5	0.644877	7.02E-62	6.38E-60
ATCAY	0.082934	6.00E-02	8.71E-02
ATE1	0.119487	6.63E-03	1.16E-02
ATF1	0.209479	1.62E-06	4.79E-06
ATF2	0.196502	7.04E-06	1.92E-05
ATF3	0.015172	7.31E-01	7.76E-01
ATF4	0.108988	1.33E-02	2.21E-02
ATF5	0.257257	3.14E-09	1.27E-08
ATF6B	-0.14162	1.27E-03	2.51E-03
ATF6	-0.03623	4.12E-01	4.78E-01
ATF7IP2	-0.35008	2.70E-16	2.24E-15
ATF7IP	0.163275	1.98E-04	4.43E-04
ATF7	-0.34545	7.02E-16	5.59E-15
ATG10	0.062577	1.56E-01	2.05E-01
ATG12	0.1947	8.57E-06	2.32E-05
ATG16L1	0.228489	1.59E-07	5.30E-07
ATG16L2	-0.29936	4.01E-12	2.21E-11
ATG2A	-0.14839	7.30E-04	1.50E-03
ATG2B	-0.26809	6.30E-10	2.76E-09
ATG3	0.294009	9.97E-12	5.26E-11
ATG4A	0.131842	2.72E-03	5.09E-03
ATG4B	-0.04784	2.79E-01	3.40E-01
ATG4C	0.159117	2.89E-04	6.29E-04

ATG4D	0.046385	2.93E-01	3.56E-01
ATG5	0.25039	8.39E-09	3.23E-08
ATG7	0.067538	1.26E-01	1.69E-01
ATG9A	0.002755	9.50E-01	9.61E-01
ATG9B	0.086564	4.96E-02	7.32E-02
ATHL1	-0.20656	2.28E-06	6.60E-06
ATIC	0.400356	3.02E-21	3.93E-20
ATL1	0.053588	2.25E-01	2.83E-01
ATL2	-0.06569	1.37E-01	1.82E-01
ATL3	0.397752	5.73E-21	7.27E-20
ATMIN	0.064823	1.42E-01	1.88E-01
ATM	-0.14677	8.36E-04	1.70E-03
ATN1	-0.12319	5.12E-03	9.17E-03
ATOH1	0.011006	8.03E-01	8.38E-01
ATOH7	0.271078	3.99E-10	1.79E-09
ATOH8	-0.5038	1.69E-34	5.64E-33
ATOX1	0.176323	5.74E-05	1.39E-04
ATP10A	-0.19983	4.87E-06	1.36E-05
ATP10B	-0.0929	3.51E-02	5.34E-02
ATP10D	0.036453	4.09E-01	4.76E-01
ATP11A	-0.40224	1.89E-21	2.50E-20
ATP11B	0.225969	2.19E-07	7.19E-07
ATP11C	0.168117	1.26E-04	2.91E-04
ATP12A	-0.05812	1.88E-01	2.41E-01
ATP13A1	-0.13425	2.26E-03	4.29E-03
ATP13A2	-0.10191	2.07E-02	3.31E-02
ATP13A3	0.468401	1.91E-29	4.74E-28
ATP13A4	-0.46751	2.51E-29	6.17E-28
ATP13A5	-0.17933	4.26E-05	1.05E-04
ATP1A1	-0.39451	1.26E-20	1.56E-19
ATP1A2	-0.57433	1.58E-46	9.78E-45
ATP1A3	0.139185	1.54E-03	3.01E-03
ATP1A4	-0.18766	1.81E-05	4.70E-05
ATP1B1	-0.31287	3.70E-13	2.27E-12
ATP1B2	-0.53911	3.63E-40	1.71E-38
ATP1B3	0.462336	1.23E-28	2.86E-27
ATP1B4	-0.02731	5.36E-01	5.99E-01
ATP2A1	0.238168	4.48E-08	1.59E-07
ATP2A2	0.23825	4.43E-08	1.57E-07
ATP2A3	0.019698	6.56E-01	7.09E-01
ATP2B1	0.291746	1.46E-11	7.54E-11
ATP2B2	-0.07068	1.09E-01	1.49E-01
ATP2B3	-0.09243	3.60E-02	5.47E-02
ATP2B4	-0.23473	7.06E-08	2.45E-07

ATP2C1	0.299139	4.17E-12	2.30E-11
ATP2C2	-0.31051	5.66E-13	3.41E-12
ATP4A	0.128893	3.39E-03	6.25E-03
ATP4B	-0.22837	1.61E-07	5.37E-07
ATP5A1	-0.00941	8.31E-01	8.61E-01
ATP5B	0.438231	1.40E-25	2.59E-24
ATP5C1	0.278094	1.34E-10	6.29E-10
ATP5D	0.048388	2.73E-01	3.35E-01
ATP5EP2	0.034665	4.32E-01	4.99E-01
ATP5E	0.084731	5.47E-02	7.99E-02
ATP5F1	0.193362	9.91E-06	2.65E-05
ATP5G1	0.216683	6.89E-07	2.13E-06
ATP5G2	0.068734	1.19E-01	1.61E-01
ATP5G3	0.411281	1.93E-22	2.75E-21
ATP5H	0.257392	3.08E-09	1.25E-08
ATP5I	0.131404	2.81E-03	5.25E-03
ATP5J2	0.296124	6.97E-12	3.74E-11
ATP5J	0.146652	8.44E-04	1.71E-03
ATP5L2	0.054592	2.16E-01	2.73E-01
ATP5L	0.118634	7.03E-03	1.23E-02
ATP5O	0.102612	1.99E-02	3.18E-02
ATP5SL	0.059301	1.79E-01	2.31E-01
ATP5S	0.048703	2.70E-01	3.32E-01
ATP6AP1L	-0.1132	1.01E-02	1.72E-02
ATP6AP1	-0.11214	1.09E-02	1.83E-02
ATP6AP2	0.095287	3.06E-02	4.72E-02
ATP6V0A1	-0.36458	1.24E-17	1.17E-16
ATP6V0A2	0.227331	1.84E-07	6.10E-07
ATP6V0A4	-0.02925	5.08E-01	5.72E-01
ATP6V0B	0.116106	8.35E-03	1.44E-02
ATP6V0C	-0.09049	4.01E-02	6.03E-02
ATP6V0D1	-0.2406	3.24E-08	1.16E-07
ATP6V0D2	-0.16332	1.97E-04	4.41E-04
ATP6V0E1	-0.17306	7.89E-05	1.87E-04
ATP6V0E2	0.037413	3.97E-01	4.63E-01
ATP6V1A	0.163798	1.89E-04	4.23E-04
ATP6V1B1	-0.15639	3.68E-04	7.88E-04
ATP6V1B2	-0.02466	5.77E-01	6.36E-01
ATP6V1C1	0.167522	1.34E-04	3.06E-04
ATP6V1C2	-0.02618	5.53E-01	6.15E-01
ATP6V1D	0.116872	7.93E-03	1.37E-02
ATP6V1E1	0.071368	1.06E-01	1.45E-01
ATP6V1E2	0.152769	5.03E-04	1.06E-03
ATP6V1F	0.272586	3.17E-10	1.44E-09

ATP6V1G1	0.040003	3.65E-01	4.31E-01
ATP6V1G2	0.004792	9.14E-01	9.31E-01
ATP6V1G3	-0.12313	5.14E-03	9.21E-03
ATP6V1H	-0.02295	6.03E-01	6.60E-01
ATP7A	-0.21548	7.97E-07	2.44E-06
ATP7B	0.017567	6.91E-01	7.40E-01
ATP8A1	-0.50496	1.13E-34	3.79E-33
ATP8A2	-0.19221	1.12E-05	2.98E-05
ATP8B1	-0.07476	9.01E-02	1.26E-01
ATP8B2	-0.17279	8.10E-05	1.91E-04
ATP8B3	0.276702	1.67E-10	7.76E-10
ATP8B4	-0.12314	5.14E-03	9.20E-03
ATP8B5P	-0.27852	1.25E-10	5.89E-10
ATP9A	-0.25231	6.39E-09	2.50E-08
ATP9B	-0.37254	2.12E-18	2.14E-17
ATPAF1	-0.22714	1.89E-07	6.25E-07
ATPAF2	-0.09329	3.43E-02	5.23E-02
ATPBD4	0.177703	5.01E-05	1.22E-04
ATPIF1	-0.20861	1.80E-06	5.28E-06
ATRIP	0.061804	1.61E-01	2.11E-01
ATRNL1	-0.13076	2.95E-03	5.50E-03
ATRN	-0.2105	1.44E-06	4.27E-06
ATRX	-0.22608	2.16E-07	7.10E-07
ATR	0.19799	5.98E-06	1.65E-05
ATXN10	-0.05921	1.80E-01	2.32E-01
ATXN1L	-0.27269	3.12E-10	1.42E-09
ATXN1	-0.14829	7.36E-04	1.51E-03
ATXN2L	0.160997	2.44E-04	5.38E-04
ATXN2	-0.03902	3.77E-01	4.43E-01
ATXN3L	0.069559	1.15E-01	1.56E-01
ATXN3	-0.1261	4.15E-03	7.56E-03
ATXN7L1	-0.19464	8.63E-06	2.33E-05
ATXN7L2	-0.00108	9.81E-01	9.85E-01
ATXN7L3B	0.168974	1.17E-04	2.69E-04
ATXN7L3	0.251209	7.47E-09	2.89E-08
ATXN7	-0.31652	1.90E-13	1.20E-12
ATXN8OS	0.045266	3.05E-01	3.69E-01
AUH	-0.25791	2.86E-09	1.16E-08
AUP1	0.195295	8.04E-06	2.18E-05
AURKAIP1	0.144421	1.01E-03	2.03E-03
AURKAPS1	0.347658	4.46E-16	3.62E-15
AURKA	0.787101	1.01E-109	1.95E-107
AURKB	0.826124	6.82E-130	1.92E-127
AURKC	0.123529	5.00E-03	8.97E-03

AUTS2	-0.41051	2.35E-22	3.33E-21
AVEN	0.318936	1.22E-13	7.82E-13
AVIL	-0.07252	1.00E-01	1.38E-01
AVL9	0.482841	1.95E-31	5.49E-30
AVPI1	-0.21409	9.41E-07	2.86E-06
AVPR1A	0.198856	5.43E-06	1.51E-05
AVPR1B	-0.04802	2.77E-01	3.39E-01
AVPR2	-0.20595	2.44E-06	7.05E-06
AVP	0.063116	1.53E-01	2.01E-01
AWAT1	0.215583	7.87E-07	2.42E-06
AWAT2	0.058092	1.88E-01	2.42E-01
AXIN1	-0.08503	5.38E-02	7.88E-02
AXIN2	-0.45498	1.12E-27	2.44E-26
AXL	-0.05414	2.20E-01	2.77E-01
AZGP1	-0.12933	3.28E-03	6.07E-03
AZI1	0.089417	4.25E-02	6.36E-02
AZI2	-0.01587	7.19E-01	7.66E-01
AZIN1	0.28264	6.48E-11	3.14E-10
AZU1	-0.34914	3.29E-16	2.70E-15
B2M	0.022761	6.06E-01	6.63E-01
B3GALNT1	0.358052	5.06E-17	4.53E-16
B3GALNT2	0.115316	8.81E-03	1.51E-02
B3GALT1	0.067653	1.25E-01	1.68E-01
B3GALT2	-0.43127	9.65E-25	1.67E-23
B3GALT4	-0.25107	7.62E-09	2.95E-08
B3GALT5	0.079211	7.25E-02	1.03E-01
B3GALT6	-0.05128	2.45E-01	3.05E-01
B3GALTL	-0.10025	2.29E-02	3.62E-02
B3GAT1	-0.2462	1.51E-08	5.62E-08
B3GAT2	-0.15562	3.93E-04	8.39E-04
B3GAT3	0.07695	8.11E-02	1.14E-01
B3GNT1	-0.32539	3.63E-14	2.45E-13
B3GNT2	0.203791	3.12E-06	8.93E-06
B3GNT3	0.162458	2.14E-04	4.75E-04
B3GNT4	0.316252	2.00E-13	1.26E-12
B3GNT5	0.528093	2.51E-38	1.05E-36
B3GNT6	-0.09194	3.70E-02	5.61E-02
B3GNT7	-0.14186	1.25E-03	2.47E-03
B3GNT8	-0.49584	2.60E-33	8.14E-32
B3GNT9	-0.33349	7.64E-15	5.48E-14
B3GNTL1	0.002638	9.52E-01	9.62E-01
B4GALNT1	0.31604	2.08E-13	1.30E-12
B4GALNT2	0.083728	5.76E-02	8.39E-02
B4GALNT3	-0.26422	1.13E-09	4.80E-09

B4GALNT4	0.18222	3.18E-05	7.94E-05
B4GALT1	0.264018	1.16E-09	4.94E-09
B4GALT2	0.202667	3.55E-06	1.01E-05
B4GALT3	0.140791	1.36E-03	2.67E-03
B4GALT4	0.296917	6.09E-12	3.29E-11
B4GALT5	0.296597	6.44E-12	3.47E-11
B4GALT6	0.318245	1.38E-13	8.85E-13
B4GALT7	-0.09988	2.34E-02	3.70E-02
B9D1	-0.08587	5.15E-02	7.57E-02
B9D2	-0.28846	2.51E-11	1.27E-10
BAALC	-0.05668	1.99E-01	2.54E-01
BAAT	-0.26023	2.04E-09	8.41E-09
BACE1	-0.14284	1.15E-03	2.29E-03
BACE2	0.015153	7.32E-01	7.76E-01
BACH1	0.253139	5.68E-09	2.23E-08
BACH2	0.079183	7.26E-02	1.04E-01
BAD	-0.1475	7.87E-04	1.61E-03
BAG1	-0.26105	1.81E-09	7.49E-09
BAG2	0.300723	3.17E-12	1.77E-11
BAG3	-0.01498	7.34E-01	7.79E-01
BAG4	0.252044	6.64E-09	2.59E-08
BAG5	0.117623	7.54E-03	1.31E-02
BAGE2	0.140742	1.36E-03	2.68E-03
BAGE	0.220837	4.15E-07	1.32E-06
BAHCC1	-0.35373	1.26E-16	1.08E-15
BAHD1	-0.19504	8.26E-06	2.24E-05
BAI1	-0.20282	3.49E-06	9.91E-06
BAI2	0.210702	1.41E-06	4.18E-06
BAI3	-0.32572	3.41E-14	2.31E-13
BAIAP2L1	0.16464	1.75E-04	3.94E-04
BAIAP2L2	0.053027	2.30E-01	2.88E-01
BAIAP2	-0.37622	9.23E-19	9.59E-18
BAIAP3	-0.26533	9.55E-10	4.09E-09
BAK1	0.190843	1.30E-05	3.42E-05
BAMBI	-0.02077	6.38E-01	6.93E-01
BANF1	0.315562	2.27E-13	1.42E-12
BANF2	0.082428	6.16E-02	8.92E-02
BANK1	-0.34987	2.83E-16	2.34E-15
BANP	0.080984	6.63E-02	9.54E-02
BAP1	-0.13963	1.49E-03	2.91E-03
BARD1	0.488912	2.66E-32	7.91E-31
BARHL1	-0.05395	2.22E-01	2.79E-01
BARHL2	0.1374	1.78E-03	3.43E-03
BARX1	0.028963	5.12E-01	5.76E-01

BARX2	-0.06073	1.69E-01	2.20E-01
BASE	-0.07243	1.01E-01	1.38E-01
BASP1	0.192635	1.07E-05	2.85E-05
BAT1	-0.06568	1.37E-01	1.82E-01
BAT2L1	-0.06214	1.59E-01	2.08E-01
BAT2L2	0.072341	1.01E-01	1.39E-01
BAT2	0.116306	8.24E-03	1.42E-02
BAT3	-0.04354	3.24E-01	3.89E-01
BAT4	0.015292	7.29E-01	7.74E-01
BAT5	-0.16688	1.42E-04	3.23E-04
BATF2	0.21118	1.33E-06	3.96E-06
BATF3	0.23658	5.54E-08	1.94E-07
BATF	0.019754	6.55E-01	7.08E-01
BAX	0.134534	2.22E-03	4.21E-03
BAZ1A	0.174867	6.62E-05	1.58E-04
BAZ1B	0.178515	4.62E-05	1.13E-04
BAZ2A	0.14431	1.02E-03	2.05E-03
BAZ2B	-0.18765	1.82E-05	4.70E-05
BBC3	-0.1944	8.86E-06	2.39E-05
BBOX1	0.056553	2.00E-01	2.55E-01
BBS10	-0.02478	5.75E-01	6.34E-01
BBS12	-0.07698	8.09E-02	1.14E-01
BBS1	-0.5095	2.27E-35	7.99E-34
BBS2	-0.3169	1.77E-13	1.12E-12
BBS4	-0.25906	2.42E-09	9.89E-09
BBS5	-0.32045	9.20E-14	5.98E-13
BBS7	0.275628	1.97E-10	9.14E-10
BBS9	-0.09199	3.69E-02	5.59E-02
BBX	0.25145	7.22E-09	2.80E-08
BCAM	-0.4717	6.83E-30	1.75E-28
BCAN	0.161705	2.29E-04	5.07E-04
BCAP29	0.132768	2.54E-03	4.77E-03
BCAP31	-0.04256	3.35E-01	4.01E-01
BCAR1	0.006038	8.91E-01	9.12E-01
BCAR3	0.239307	3.85E-08	1.37E-07
BCAR4	0.136728	1.87E-03	3.60E-03
BCAS1	-0.19888	5.42E-06	1.50E-05
BCAS2	0.258732	2.54E-09	1.04E-08
BCAS3	-0.24711	1.33E-08	4.98E-08
BCAS4	-0.06472	1.42E-01	1.89E-01
BCAT1	-0.05795	1.89E-01	2.43E-01
BCAT2	-0.30151	2.77E-12	1.55E-11
BCCIP	0.356406	7.18E-17	6.31E-16
BCDIN3D	-0.1479	7.61E-04	1.56E-03

BCHE	-0.30802	8.84E-13	5.22E-12
BCKDHA	-0.32162	7.40E-14	4.85E-13
BCKDHB	-0.1678	1.30E-04	2.99E-04
BCKDK	0.016745	7.05E-01	7.53E-01
BCL10	0.219258	5.04E-07	1.59E-06
BCL11A	-0.25977	2.18E-09	8.95E-09
BCL11B	-0.13209	2.67E-03	5.01E-03
BCL2A1	0.154257	4.43E-04	9.37E-04
BCL2L10	0.343548	1.03E-15	8.07E-15
BCL2L11	0.22739	1.83E-07	6.06E-07
BCL2L12	0.353783	1.25E-16	1.07E-15
BCL2L13	0.19441	8.85E-06	2.39E-05
BCL2L14	-0.1268	3.95E-03	7.21E-03
BCL2L15	-0.20555	2.55E-06	7.36E-06
BCL2L1	0.093796	3.33E-02	5.10E-02
BCL2L2	-0.22208	3.56E-07	1.14E-06
BCL2	-0.14166	1.27E-03	2.51E-03
BCL3	0.134594	2.21E-03	4.19E-03
BCL6B	-0.13103	2.89E-03	5.39E-03
BCL6	-0.26992	4.77E-10	2.12E-09
BCL7A	-0.03516	4.26E-01	4.92E-01
BCL7B	0.090022	4.11E-02	6.18E-02
BCL7C	-0.04166	3.45E-01	4.11E-01
BCL8	0.012047	7.85E-01	8.22E-01
BCL9L	0.010641	8.10E-01	8.42E-01
BCL9	-0.00758	8.64E-01	8.89E-01
BCLAF1	-0.04014	3.63E-01	4.30E-01
BCMO1	-0.17006	1.05E-04	2.45E-04
BCO2	-0.22339	3.02E-07	9.79E-07
BCORL1	-0.00123	9.78E-01	9.83E-01
BCORL2	-0.00648	8.83E-01	9.05E-01
BCOR	-0.17852	4.62E-05	1.13E-04
BCR	-0.11118	1.16E-02	1.94E-02
BCS1L	0.140576	1.38E-03	2.72E-03
BCYRN1	0.015421	7.27E-01	7.72E-01
BDH1	-0.02176	6.22E-01	6.78E-01
BDH2	-0.34971	2.92E-16	2.41E-15
BDKRB1	0.25181	6.86E-09	2.67E-08
BDKRB2	0.014716	7.39E-01	7.83E-01
BDNFOS	-0.3822	2.34E-19	2.58E-18
BDNF	-0.06382	1.48E-01	1.95E-01
BDP1	0.025532	5.63E-01	6.24E-01
BEAN	0.046582	2.91E-01	3.54E-01
BECN1	-0.02093	6.36E-01	6.91E-01

BEGAIN	-0.05796	1.89E-01	2.43E-01
BEND2	-0.11055	1.21E-02	2.02E-02
BEND3	0.110835	1.18E-02	1.98E-02
BEND4	0.096002	2.94E-02	4.54E-02
BEND5	-0.27565	1.97E-10	9.10E-10
BEND6	0.401762	2.13E-21	2.80E-20
BEND7	-0.23567	6.25E-08	2.17E-07
BEST1	0.084344	5.58E-02	8.14E-02
BEST2	0.138755	1.60E-03	3.10E-03
BEST3	0.347269	4.83E-16	3.91E-15
BEST4	-0.12556	4.32E-03	7.84E-03
BET1L	-0.29748	5.54E-12	3.01E-11
BET1	0.074186	9.26E-02	1.29E-01
BET3L	0.105751	1.64E-02	2.67E-02
BEX1	0.014515	7.42E-01	7.86E-01
BEX2	-0.18395	2.67E-05	6.73E-05
BEX4	-0.31407	2.98E-13	1.85E-12
BEX5	-0.20442	2.91E-06	8.33E-06
BEYLA	-0.02274	6.07E-01	6.64E-01
BFAR	-0.30718	1.03E-12	6.01E-12
BFSP1	-0.04465	3.12E-01	3.76E-01
BFSP2	0.042464	3.36E-01	4.02E-01
BGLAP	-0.11748	7.61E-03	1.32E-02
BGN	-0.16208	2.21E-04	4.91E-04
BHLHA15	0.039126	3.76E-01	4.42E-01
BHLHB9	-0.0443	3.16E-01	3.80E-01
BHLHE22	-0.21317	1.05E-06	3.17E-06
BHLHE23	0.064759	1.42E-01	1.89E-01
BHLHE40	-0.02816	5.24E-01	5.87E-01
BHLHE41	-0.05119	2.46E-01	3.06E-01
BHMT2	0.019964	6.51E-01	7.05E-01
BHMT	-0.06729	1.27E-01	1.71E-01
BICC1	-0.02531	5.67E-01	6.27E-01
BICD1	0.39317	1.74E-20	2.12E-19
BICD2	-0.11995	6.42E-03	1.13E-02
BID	0.352918	1.50E-16	1.27E-15
BIK	0.210364	1.46E-06	4.34E-06
BIN1	0.074105	9.30E-02	1.29E-01
BIN2	-0.07611	8.44E-02	1.18E-01
BIN3	-0.15458	4.31E-04	9.13E-04
BIRC2	0.171527	9.15E-05	2.14E-04
BIRC3	0.091772	3.73E-02	5.65E-02
BIRC5	0.862957	3.32E-154	2.21E-151
BIRC6	0.064821	1.42E-01	1.88E-01

BIRC7	-0.16745	1.35E-04	3.08E-04
BIRC8	0.04847	2.72E-01	3.34E-01
BIVM	-0.16702	1.40E-04	3.20E-04
BLCAP	-0.12356	4.99E-03	8.96E-03
BLID	0.184769	2.45E-05	6.22E-05
BLK	-0.25379	5.18E-09	2.04E-08
BLMH	0.197177	6.54E-06	1.79E-05
BLM	0.741691	4.99E-91	7.03E-89
BLNK	-0.21603	7.45E-07	2.30E-06
BLOC1S1	-0.02021	6.47E-01	7.01E-01
BLOC1S2	0.128151	3.58E-03	6.57E-03
BLOC1S3	0.023026	6.02E-01	6.60E-01
BLVRA	-0.21261	1.12E-06	3.38E-06
BLVRB	-0.03216	4.66E-01	5.32E-01
BLZF1	0.128245	3.55E-03	6.53E-03
BMF	-0.22963	1.37E-07	4.62E-07
BMI1	0.097359	2.72E-02	4.23E-02
BMP10	0.03075	4.86E-01	5.51E-01
BMP15	-0.09616	2.91E-02	4.51E-02
BMP1	0.160657	2.51E-04	5.53E-04
BMP2K	0.067141	1.28E-01	1.72E-01
BMP2	-0.29347	1.09E-11	5.73E-11
BMP3	-0.37093	3.04E-18	3.03E-17
BMP4	-0.22984	1.33E-07	4.50E-07
BMP5	-0.26591	8.76E-10	3.77E-09
BMP6	-0.11262	1.05E-02	1.78E-02
BMP7	-0.13054	3.00E-03	5.58E-03
BMP8A	0.117895	7.40E-03	1.29E-02
BMP8B	0.08145	6.48E-02	9.34E-02
BMPER	-0.25182	6.86E-09	2.67E-08
BMPR1A	-0.03501	4.28E-01	4.94E-01
BMPR1B	0.069427	1.16E-01	1.57E-01
BMPR2	-0.17573	6.08E-05	1.46E-04
BMS1P4	-0.2424	2.53E-08	9.22E-08
BMS1P5	-0.28566	3.98E-11	1.96E-10
BMS1	0.237211	5.09E-08	1.79E-07
BMX	-0.26768	6.71E-10	2.93E-09
BNC1	0.074518	9.12E-02	1.27E-01
BNC2	-0.08723	4.79E-02	7.08E-02
BNIP1	0.146031	8.88E-04	1.80E-03
BNIP2	0.046653	2.91E-01	3.53E-01
BNIP3L	-0.17256	8.29E-05	1.95E-04
BNIP3	0.073223	9.69E-02	1.34E-01
BNIPL	-0.40847	3.95E-22	5.50E-21

BOC	-0.21467	8.77E-07	2.68E-06
BOD1L	-0.07991	7.00E-02	1.00E-01
BOD1	0.202183	3.75E-06	1.06E-05
BOK	-0.35623	7.46E-17	6.54E-16
BOLA1	0.055025	2.13E-01	2.69E-01
BOLA2	0.344559	8.41E-16	6.65E-15
BOLA3	0.516414	1.90E-36	7.11E-35
BOLL	0.077888	7.74E-02	1.10E-01
BOP1	0.27653	1.71E-10	7.97E-10
BPESC1	0.073338	9.64E-02	1.33E-01
BPGM	0.071284	1.06E-01	1.45E-01
BPHL	0.015539	7.25E-01	7.71E-01
BPIL1	-0.11164	1.12E-02	1.89E-02
BPIL2	0.005064	9.09E-01	9.27E-01
BPIL3	-0.00394	9.29E-01	9.43E-01
BPI	-0.15918	2.87E-04	6.26E-04
BPNT1	-0.00686	8.77E-01	9.00E-01
BPTF	-0.06321	1.52E-01	2.00E-01
BPY2	0.029965	4.97E-01	5.62E-01
BRAF	0.170551	1.00E-04	2.34E-04
BRAP	0.235789	6.15E-08	2.14E-07
BRCA1	0.781288	4.41E-107	8.01E-105
BRCA2	0.647364	1.70E-62	1.57E-60
BRCC3	0.205509	2.57E-06	7.40E-06
BRD1	-0.32257	6.19E-14	4.09E-13
BRD2	-0.078	7.70E-02	1.09E-01
BRD3	-0.04906	2.66E-01	3.28E-01
BRD4	0.117648	7.53E-03	1.31E-02
BRD7P3	0.057764	1.91E-01	2.44E-01
BRD7	0.007437	8.66E-01	8.91E-01
BRD8	-0.15746	3.34E-04	7.22E-04
BRD9	0.097364	2.71E-02	4.23E-02
BRDT	-0.02696	5.42E-01	6.04E-01
BREA2	-0.17371	7.41E-05	1.76E-04
BRE	-0.0133	7.63E-01	8.03E-01
BRF1	-0.21058	1.43E-06	4.24E-06
BRF2	0.129667	3.20E-03	5.93E-03
BRI3BP	0.621585	2.24E-56	1.78E-54
BRI3	-0.00341	9.38E-01	9.52E-01
BRIP1	0.750659	2.14E-94	3.09E-92
BRIX1	0.508092	3.74E-35	1.29E-33
BRMS1L	0.117239	7.74E-03	1.34E-02
BRMS1	0.178418	4.66E-05	1.14E-04
BRP44L	-0.22723	1.86E-07	6.17E-07

BRP44	-0.05103	2.48E-01	3.08E-01
BRPF1	0.026354	5.51E-01	6.12E-01
BRPF3	-0.03041	4.91E-01	5.56E-01
BRS3	0.042387	3.37E-01	4.02E-01
BRSK1	0.218665	5.41E-07	1.70E-06
BRSK2	-0.04165	3.45E-01	4.11E-01
BRWD1	-0.21515	8.29E-07	2.54E-06
BRWD3	0.186955	1.95E-05	5.03E-05
BSCL2	-0.25153	7.14E-09	2.77E-08
BSDC1	-0.32018	9.68E-14	6.27E-13
BSG	0.041945	3.42E-01	4.08E-01
BSND	0.161722	2.28E-04	5.06E-04
BSN	-0.07747	7.90E-02	1.12E-01
BSPRY	-0.02225	6.14E-01	6.71E-01
BST1	0.041762	3.44E-01	4.10E-01
BST2	0.002858	9.48E-01	9.59E-01
BSX	0.077061	8.06E-02	1.14E-01
BTAF1	-0.10563	1.65E-02	2.69E-02
BTBD10	0.219422	4.94E-07	1.56E-06
BTBD11	-0.00486	9.12E-01	9.30E-01
BTBD12	0.01853	6.75E-01	7.26E-01
BTBD16	0.109359	1.30E-02	2.17E-02
BTBD17	0.150881	5.92E-04	1.23E-03
BTBD18	-0.1676	1.33E-04	3.04E-04
BTBD19	-0.07339	9.62E-02	1.33E-01
BTBD1	0.112866	1.04E-02	1.75E-02
BTBD2	0.041526	3.47E-01	4.13E-01
BTBD3	0.028377	5.21E-01	5.84E-01
BTBD6	-0.07917	7.26E-02	1.04E-01
BTBD7	-0.14034	1.41E-03	2.76E-03
BTBD8	0.072556	1.00E-01	1.38E-01
BTBD9	-0.55866	1.33E-43	7.25E-42
BTC	-0.22992	1.32E-07	4.46E-07
BTD	-0.45411	1.45E-27	3.12E-26
BTF3L1	0.006698	8.79E-01	9.02E-01
BTF3L4	0.267749	6.64E-10	2.90E-09
BTF3	0.008515	8.47E-01	8.75E-01
BTG1	-0.20914	1.69E-06	4.98E-06
BTG2	-0.47825	8.57E-31	2.34E-29
BTG3	-0.17202	8.73E-05	2.05E-04
BTG4	-0.05728	1.94E-01	2.49E-01
BTK	-0.16547	1.62E-04	3.66E-04
BTLA	-0.15435	4.39E-04	9.30E-04
BTN1A1	-0.07687	8.14E-02	1.15E-01

BTN2A1	-0.11211	1.09E-02	1.84E-02
BTN2A2	-0.17226	8.53E-05	2.01E-04
BTN2A3	-0.07667	8.22E-02	1.16E-01
BTN3A1	-0.0648	1.42E-01	1.88E-01
BTN3A2	-0.08516	5.34E-02	7.83E-02
BTN3A3	-0.14038	1.40E-03	2.76E-03
BTNL2	0.026179	5.53E-01	6.15E-01
BTNL3	-0.04747	2.82E-01	3.44E-01
BTNL8	-0.21687	6.73E-07	2.09E-06
BTNL9	-0.54757	1.26E-41	6.27E-40
BTRC	-0.21461	8.84E-07	2.70E-06
BUB1B	0.880872	9.38E-169	1.25E-165
BUB1	0.898702	8.98E-186	5.98E-182
BUB3	0.573651	2.13E-46	1.31E-44
BUD13	0.197457	6.34E-06	1.74E-05
BUD31	0.290473	1.80E-11	9.24E-11
BVES	0.218681	5.40E-07	1.70E-06
BYSL	0.4075	5.06E-22	6.97E-21
BZRAP1	-0.40954	3.01E-22	4.23E-21
BZW1	0.430369	1.23E-24	2.12E-23
BZW2	0.386918	7.74E-20	8.89E-19
C10orf105	-0.3946	1.24E-20	1.53E-19
C10orf107	-0.41163	1.77E-22	2.52E-21
C10orf108	-0.00431	9.22E-01	9.38E-01
C10orf10	-0.16379	1.89E-04	4.24E-04
C10orf110	-0.11994	6.43E-03	1.13E-02
C10orf111	-0.13527	2.09E-03	4.00E-03
C10orf113	-0.00072	9.87E-01	9.90E-01
C10orf114	0.169977	1.06E-04	2.46E-04
C10orf116	-0.45309	1.95E-27	4.19E-26
C10orf118	-0.20233	3.68E-06	1.04E-05
C10orf119	0.340258	2.00E-15	1.51E-14
C10orf11	-0.22972	1.36E-07	4.57E-07
C10orf120	-0.05356	2.25E-01	2.83E-01
C10orf122	0.044047	3.18E-01	3.83E-01
C10orf125	0.143089	1.13E-03	2.25E-03
C10orf128	-0.17663	5.57E-05	1.35E-04
C10orf129	0.009152	8.36E-01	8.65E-01
C10orf12	0.327361	2.50E-14	1.71E-13
C10orf131	0.027868	5.28E-01	5.91E-01
C10orf137	-0.06133	1.65E-01	2.15E-01
C10orf140	-0.07832	7.58E-02	1.08E-01
C10orf18	0.223463	2.99E-07	9.70E-07
C10orf25	0.00602	8.92E-01	9.12E-01

C10orf26	-0.28684	3.28E-11	1.63E-10
C10orf27	0.057265	1.94E-01	2.49E-01
C10orf28	-0.09265	3.56E-02	5.41E-02
C10orf2	0.279155	1.13E-10	5.35E-10
C10orf32	-0.47671	1.40E-30	3.75E-29
C10orf35	0.008706	8.44E-01	8.72E-01
C10orf40	0.064263	1.45E-01	1.92E-01
C10orf41	-0.0444	3.15E-01	3.79E-01
C10orf46	0.247891	1.19E-08	4.49E-08
C10orf47	-0.09286	3.51E-02	5.35E-02
C10orf4	-0.09344	3.40E-02	5.19E-02
C10orf50	-0.17352	7.55E-05	1.79E-04
C10orf53	-0.11654	8.12E-03	1.40E-02
C10orf54	-0.21033	1.47E-06	4.35E-06
C10orf55	0.262171	1.53E-09	6.40E-09
C10orf57	-0.13292	2.51E-03	4.72E-03
C10orf58	0.064681	1.43E-01	1.89E-01
C10orf62	0.107194	1.49E-02	2.46E-02
C10orf67	-0.14191	1.24E-03	2.46E-03
C10orf68	-0.35114	2.17E-16	1.82E-15
C10orf71	-0.06858	1.20E-01	1.62E-01
C10orf72	-0.33285	8.66E-15	6.18E-14
C10orf75	-0.11447	9.33E-03	1.59E-02
C10orf76	-0.45056	4.11E-27	8.54E-26
C10orf78	0.287749	2.82E-11	1.42E-10
C10orf79	-0.26755	6.84E-10	2.98E-09
C10orf81	-0.18768	1.81E-05	4.69E-05
C10orf82	0.029936	4.98E-01	5.62E-01
C10orf84	0.110228	1.23E-02	2.06E-02
C10orf88	0.253351	5.51E-09	2.17E-08
C10orf90	0.229637	1.37E-07	4.62E-07
C10orf91	0.113801	9.75E-03	1.66E-02
C10orf93	-0.24887	1.04E-08	3.95E-08
C10orf95	-0.35495	9.77E-17	8.48E-16
C10orf96	0.058717	1.83E-01	2.36E-01
C10orf99	0.104852	1.73E-02	2.81E-02
C11orf10	0.178047	4.84E-05	1.18E-04
C11orf16	-0.34495	7.77E-16	6.16E-15
C11orf17	0.010065	8.20E-01	8.52E-01
C11orf1	-0.20287	3.47E-06	9.86E-06
C11orf20	0.088974	4.36E-02	6.50E-02
C11orf21	-0.24852	1.09E-08	4.14E-08
C11orf24	0.246197	1.51E-08	5.62E-08
C11orf2	-0.27	4.71E-10	2.10E-09

C11orf30	0.106804	1.53E-02	2.51E-02
C11orf31	0.119186	6.77E-03	1.19E-02
C11orf34	-0.10866	1.36E-02	2.26E-02
C11orf35	-0.31234	4.07E-13	2.49E-12
C11orf36	0.058937	1.82E-01	2.34E-01
C11orf41	0.110189	1.23E-02	2.06E-02
C11orf42	-0.15391	4.56E-04	9.64E-04
C11orf45	-0.09439	3.22E-02	4.94E-02
C11orf46	-0.11548	8.71E-03	1.49E-02
C11orf48	0.229319	1.43E-07	4.80E-07
C11orf49	-0.28595	3.79E-11	1.87E-10
C11orf51	0.011944	7.87E-01	8.23E-01
C11orf52	-0.42034	1.83E-23	2.85E-22
C11orf53	0.042152	3.40E-01	4.05E-01
C11orf54	-0.3072	1.02E-12	5.99E-12
C11orf57	0.068544	1.20E-01	1.62E-01
C11orf58	0.003633	9.34E-01	9.48E-01
C11orf59	0.080314	6.86E-02	9.85E-02
C11orf61	-0.35162	1.96E-16	1.65E-15
C11orf63	-0.2257	2.26E-07	7.43E-07
C11orf64	0.029857	4.99E-01	5.64E-01
C11orf65	-0.11398	9.63E-03	1.64E-02
C11orf66	-0.3361	4.58E-15	3.37E-14
C11orf67	-0.07904	7.31E-02	1.04E-01
C11orf68	-0.10346	1.88E-02	3.03E-02
C11orf70	-0.11641	8.18E-03	1.41E-02
C11orf71	-0.09549	3.03E-02	4.67E-02
C11orf73	0.209465	1.62E-06	4.80E-06
C11orf74	-0.05375	2.23E-01	2.81E-01
C11orf75	-0.18638	2.07E-05	5.32E-05
C11orf80	0.143951	1.05E-03	2.11E-03
C11orf82	0.785786	4.05E-109	7.71E-107
C11orf83	0.180013	3.98E-05	9.80E-05
C11orf84	0.434924	3.52E-25	6.31E-24
C11orf85	0.067127	1.28E-01	1.72E-01
C11orf86	0.165334	1.64E-04	3.70E-04
C11orf87	0.036117	4.13E-01	4.80E-01
C11orf88	-0.28384	5.34E-11	2.60E-10
C11orf90	0.217951	5.91E-07	1.84E-06
C11orf92	-0.37812	5.98E-19	6.31E-18
C11orf93	-0.29428	9.53E-12	5.04E-11
C11orf94	0.01279	7.72E-01	8.10E-01
C11orf95	-0.06072	1.69E-01	2.20E-01
C11orf9	-0.0333	4.51E-01	5.17E-01

C12orf10	0.163628	1.92E-04	4.30E-04
C12orf11	0.570555	8.26E-46	4.97E-44
C12orf12	0.061698	1.62E-01	2.12E-01
C12orf23	0.378314	5.72E-19	6.05E-18
C12orf24	0.334178	6.68E-15	4.83E-14
C12orf26	-0.00763	8.63E-01	8.88E-01
C12orf27	-0.2199	4.66E-07	1.47E-06
C12orf29	0.422384	1.06E-23	1.70E-22
C12orf32	0.473819	3.51E-30	9.15E-29
C12orf34	0.354902	9.87E-17	8.56E-16
C12orf35	0.106888	1.52E-02	2.50E-02
C12orf36	0.151951	5.40E-04	1.13E-03
C12orf39	0.339992	2.11E-15	1.59E-14
C12orf40	0.019208	6.64E-01	7.16E-01
C12orf41	0.312075	4.27E-13	2.60E-12
C12orf42	-0.03583	4.17E-01	4.84E-01
C12orf43	0.434415	4.06E-25	7.21E-24
C12orf44	0.26726	7.15E-10	3.11E-09
C12orf45	0.324914	3.98E-14	2.68E-13
C12orf47	0.100084	2.31E-02	3.66E-02
C12orf48	0.804357	4.45E-118	9.98E-116
C12orf49	0.109532	1.29E-02	2.14E-02
C12orf4	0.350653	2.40E-16	2.00E-15
C12orf50	-0.02305	6.02E-01	6.59E-01
C12orf51	-0.08011	6.93E-02	9.93E-02
C12orf52	0.247801	1.21E-08	4.55E-08
C12orf53	-0.00852	8.47E-01	8.75E-01
C12orf54	0.015319	7.29E-01	7.74E-01
C12orf56	0.301056	2.99E-12	1.68E-11
C12orf57	0.02621	5.53E-01	6.14E-01
C12orf59	-0.05134	2.45E-01	3.05E-01
C12orf5	0.261985	1.57E-09	6.58E-09
C12orf60	0.122963	5.20E-03	9.31E-03
C12orf61	0.099936	2.33E-02	3.68E-02
C12orf62	-0.11668	8.04E-03	1.39E-02
C12orf63	-0.14092	1.35E-03	2.65E-03
C12orf65	0.15357	4.70E-04	9.90E-04
C12orf66	0.267711	6.67E-10	2.92E-09
C12orf68	-0.09792	2.63E-02	4.10E-02
C12orf69	-0.40139	2.34E-21	3.06E-20
C12orf70	0.386362	8.83E-20	1.01E-18
C12orf71	-0.11607	8.38E-03	1.44E-02
C12orf72	-0.23192	1.02E-07	3.48E-07
C12orf73	0.307331	9.98E-13	5.86E-12

C12orf74	-0.14692	8.25E-04	1.68E-03
C12orf75	0.172938	7.99E-05	1.89E-04
C12orf76	-0.26419	1.13E-09	4.82E-09
C12orf77	-0.01915	6.65E-01	7.17E-01
C13orf15	-0.42504	5.23E-24	8.57E-23
C13orf16	0.123402	5.04E-03	9.05E-03
C13orf18	-0.09284	3.52E-02	5.36E-02
C13orf1	-0.01725	6.96E-01	7.45E-01
C13orf23	0.045465	3.03E-01	3.67E-01
C13orf26	-0.21734	6.36E-07	1.98E-06
C13orf27	0.513219	6.02E-36	2.18E-34
C13orf29	0.350576	2.44E-16	2.03E-15
C13orf30	-0.31858	1.30E-13	8.33E-13
C13orf31	0.025975	5.56E-01	6.18E-01
C13orf33	0.135371	2.08E-03	3.97E-03
C13orf34	0.623831	6.92E-57	5.57E-55
C13orf35	-0.13107	2.88E-03	5.38E-03
C13orf36	-0.12225	5.47E-03	9.75E-03
C13orf37	0.497901	1.29E-33	4.09E-32
C13orf38	0.248666	1.07E-08	4.06E-08
C13orf39	-0.24579	1.59E-08	5.93E-08
C14orf101	-0.03401	4.41E-01	5.08E-01
C14orf102	-0.13516	2.11E-03	4.02E-03
C14orf104	0.158735	2.99E-04	6.50E-04
C14orf105	-0.18076	3.69E-05	9.14E-05
C14orf106	0.347638	4.48E-16	3.64E-15
C14orf109	0.118465	7.12E-03	1.24E-02
C14orf115	-0.01917	6.64E-01	7.16E-01
C14orf118	0.334846	5.86E-15	4.27E-14
C14orf119	0.116859	7.94E-03	1.37E-02
C14orf126	0.091114	3.87E-02	5.84E-02
C14orf128	0.070017	1.13E-01	1.53E-01
C14orf129	0.335449	5.21E-15	3.81E-14
C14orf132	-0.27543	2.03E-10	9.41E-10
C14orf135	0.130701	2.96E-03	5.52E-03
C14orf138	-0.05448	2.17E-01	2.74E-01
C14orf139	-0.2628	1.39E-09	5.86E-09
C14orf142	0.033752	4.45E-01	5.11E-01
C14orf143	0.316797	1.81E-13	1.14E-12
C14orf145	0.370805	3.13E-18	3.11E-17
C14orf147	0.076547	8.27E-02	1.16E-01
C14orf148	-0.07991	7.00E-02	1.00E-01
C14orf149	0.077215	8.00E-02	1.13E-01
C14orf153	0.233994	7.79E-08	2.69E-07

C14orf156	0.308004	8.85E-13	5.23E-12
C14orf159	-0.33869	2.74E-15	2.05E-14
C14orf162	0.098634	2.52E-02	3.95E-02
C14orf165	-0.00941	8.31E-01	8.61E-01
C14orf166B	-0.07797	7.71E-02	1.09E-01
C14orf166	0.268575	5.85E-10	2.57E-09
C14orf167	-0.16136	2.36E-04	5.21E-04
C14orf169	0.070893	1.08E-01	1.48E-01
C14orf174	-0.06183	1.61E-01	2.11E-01
C14orf176	-0.08318	5.92E-02	8.61E-02
C14orf178	-0.18386	2.69E-05	6.78E-05
C14orf179	-0.00077	9.86E-01	9.89E-01
C14orf180	-0.36814	5.66E-18	5.48E-17
C14orf181	-0.07372	9.47E-02	1.31E-01
C14orf182	0.023147	6.00E-01	6.58E-01
C14orf183	-0.04107	3.52E-01	4.18E-01
C14orf184	-0.12576	4.26E-03	7.74E-03
C14orf19	-0.03958	3.70E-01	4.36E-01
C14orf1	0.03461	4.33E-01	5.00E-01
C14orf21	0.099589	2.38E-02	3.76E-02
C14orf23	0.185975	2.16E-05	5.54E-05
C14orf28	-0.3084	8.26E-13	4.90E-12
C14orf2	0.240275	3.38E-08	1.21E-07
C14orf33	0.27734	1.51E-10	7.05E-10
C14orf34	0.234979	6.84E-08	2.38E-07
C14orf37	0.063399	1.51E-01	1.99E-01
C14orf39	-0.05782	1.90E-01	2.44E-01
C14orf43	-0.07308	9.76E-02	1.35E-01
C14orf45	-0.23992	3.55E-08	1.27E-07
C14orf48	0.038975	3.77E-01	4.44E-01
C14orf49	-0.06585	1.36E-01	1.81E-01
C14orf4	-0.03237	4.64E-01	5.29E-01
C14orf50	0.000934	9.83E-01	9.87E-01
C14orf53	-0.0132	7.65E-01	8.05E-01
C14orf64	-0.34387	9.67E-16	7.58E-15
C14orf68	0.111195	1.16E-02	1.94E-02
C14orf70	0.070983	1.08E-01	1.47E-01
C14orf72	-0.0622	1.59E-01	2.08E-01
C14orf73	-0.18277	3.01E-05	7.53E-05
C14orf79	-0.24712	1.33E-08	4.98E-08
C14orf80	0.370409	3.41E-18	3.39E-17
C14orf86	-0.1391	1.55E-03	3.03E-03
C14orf93	-0.16397	1.86E-04	4.17E-04
C15orf17	-0.48739	4.40E-32	1.29E-30

C15orf21	0.263843	1.19E-09	5.06E-09
C15orf23	0.671682	7.71E-69	7.90E-67
C15orf24	0.036827	4.04E-01	4.71E-01
C15orf26	-0.20907	1.70E-06	5.01E-06
C15orf27	-0.10582	1.63E-02	2.66E-02
C15orf28	-0.26371	1.22E-09	5.15E-09
C15orf29	0.107044	1.51E-02	2.48E-02
C15orf2	-0.18307	2.92E-05	7.32E-05
C15orf32	0.043051	3.30E-01	3.95E-01
C15orf33	-0.12918	3.32E-03	6.13E-03
C15orf34	-0.29315	1.15E-11	6.03E-11
C15orf37	-0.15088	5.92E-04	1.23E-03
C15orf38	-0.27759	1.45E-10	6.80E-10
C15orf39	-0.13049	3.01E-03	5.60E-03
C15orf40	-0.04635	2.94E-01	3.57E-01
C15orf41	0.411273	1.93E-22	2.75E-21
C15orf42	0.779616	2.45E-106	4.41E-104
C15orf43	-0.12103	5.96E-03	1.06E-02
C15orf44	0.163835	1.88E-04	4.22E-04
C15orf48	0.410936	2.11E-22	2.99E-21
C15orf50	-0.25317	5.65E-09	2.22E-08
C15orf51	-0.21159	1.27E-06	3.79E-06
C15orf52	-0.31742	1.61E-13	1.03E-12
C15orf53	0.070484	1.10E-01	1.50E-01
C15orf54	-0.12635	4.08E-03	7.43E-03
C15orf55	-0.00018	9.97E-01	9.97E-01
C15orf56	-0.36954	4.14E-18	4.07E-17
C15orf57	-0.12747	3.76E-03	6.89E-03
C15orf58	0.011565	7.93E-01	8.29E-01
C15orf59	-0.50597	7.91E-35	2.68E-33
C15orf5	-0.10273	1.97E-02	3.16E-02
C15orf60	-0.09204	3.68E-02	5.58E-02
C15orf61	-0.02272	6.07E-01	6.64E-01
C15orf62	-0.24006	3.48E-08	1.25E-07
C15orf63	0.138862	1.58E-03	3.08E-03
C16orf11	0.112234	1.08E-02	1.82E-02
C16orf13	-0.13193	2.70E-03	5.06E-03
C16orf3	-0.21459	8.86E-07	2.70E-06
C16orf42	-0.11287	1.04E-02	1.75E-02
C16orf45	-0.22732	1.84E-07	6.11E-07
C16orf46	-0.18031	3.86E-05	9.53E-05
C16orf48	-0.39359	1.58E-20	1.93E-19
C16orf52	-0.18554	2.26E-05	5.78E-05
C16orf53	0.023753	5.91E-01	6.49E-01

C16orf54	-0.28459	4.73E-11	2.31E-10
C16orf55	-0.0743	9.21E-02	1.28E-01
C16orf57	0.136559	1.90E-03	3.65E-03
C16orf58	-0.43576	2.79E-25	5.03E-24
C16orf59	0.620077	4.91E-56	3.85E-54
C16orf5	-0.30873	7.78E-13	4.62E-12
C16orf61	0.394795	1.18E-20	1.46E-19
C16orf62	-0.19723	6.50E-06	1.78E-05
C16orf63	0.133997	2.31E-03	4.37E-03
C16orf68	0.085319	5.30E-02	7.77E-02
C16orf70	0.109233	1.31E-02	2.18E-02
C16orf71	-0.35708	6.22E-17	5.50E-16
C16orf72	-0.04762	2.81E-01	3.43E-01
C16orf73	0.233684	8.11E-08	2.80E-07
C16orf74	0.07901	7.32E-02	1.04E-01
C16orf75	0.52896	1.81E-38	7.61E-37
C16orf78	-0.01268	7.74E-01	8.12E-01
C16orf79	-0.10668	1.54E-02	2.53E-02
C16orf7	-0.12202	5.56E-03	9.90E-03
C16orf80	0.089601	4.21E-02	6.31E-02
C16orf81	-0.17322	7.77E-05	1.84E-04
C16orf82	0.002353	9.58E-01	9.66E-01
C16orf86	-0.30764	9.45E-13	5.57E-12
C16orf87	0.312475	3.97E-13	2.43E-12
C16orf88	0.382332	2.27E-19	2.51E-18
C16orf89	-0.66419	8.05E-67	8.09E-65
C16orf90	0.059204	1.80E-01	2.32E-01
C16orf91	0.091283	3.84E-02	5.79E-02
C16orf92	0.005268	9.05E-01	9.23E-01
C16orf93	-0.2883	2.58E-11	1.30E-10
C17orf100	-0.06947	1.15E-01	1.56E-01
C17orf101	-0.11179	1.11E-02	1.87E-02
C17orf102	-0.34297	1.16E-15	9.01E-15
C17orf103	-0.50916	2.57E-35	8.99E-34
C17orf104	0.301876	2.60E-12	1.46E-11
C17orf105	-0.01914	6.65E-01	7.17E-01
C17orf106	0.01086	8.06E-01	8.40E-01
C17orf107	-0.21283	1.09E-06	3.29E-06
C17orf108	-0.51702	1.52E-36	5.72E-35
C17orf28	-0.2197	4.77E-07	1.51E-06
C17orf37	0.146103	8.83E-04	1.79E-03
C17orf39	-0.22347	2.99E-07	9.70E-07
C17orf42	0.377166	7.44E-19	7.80E-18
C17orf44	-0.32471	4.14E-14	2.78E-13

C17orf46	-0.10813	1.41E-02	2.33E-02
C17orf47	-0.14741	7.92E-04	1.62E-03
C17orf48	-0.22846	1.59E-07	5.32E-07
C17orf49	0.019878	6.53E-01	7.06E-01
C17orf50	-0.33371	7.32E-15	5.27E-14
C17orf51	0.065777	1.36E-01	1.81E-01
C17orf53	0.730114	7.02E-87	9.42E-85
C17orf54	-0.20103	4.27E-06	1.20E-05
C17orf55	-0.27288	3.02E-10	1.38E-09
C17orf56	-0.12686	3.93E-03	7.18E-03
C17orf57	-0.03943	3.72E-01	4.38E-01
C17orf58	0.401605	2.22E-21	2.91E-20
C17orf59	-0.25126	7.42E-09	2.87E-08
C17orf60	0.008094	8.55E-01	8.81E-01
C17orf61	0.03898	3.77E-01	4.44E-01
C17orf62	0.057774	1.91E-01	2.44E-01
C17orf63	0.105796	1.63E-02	2.66E-02
C17orf64	0.202914	3.45E-06	9.81E-06
C17orf65	-0.1565	3.64E-04	7.81E-04
C17orf66	-0.00188	9.66E-01	9.74E-01
C17orf67	0.119469	6.64E-03	1.16E-02
C17orf68	-0.28464	4.69E-11	2.29E-10
C17orf69	-0.0683	1.22E-01	1.64E-01
C17orf70	0.000128	9.98E-01	9.98E-01
C17orf71	0.333238	8.03E-15	5.74E-14
C17orf72	-0.34144	1.58E-15	1.21E-14
C17orf73	0.075893	8.53E-02	1.20E-01
C17orf74	0.128426	3.51E-03	6.45E-03
C17orf75	0.385427	1.10E-19	1.24E-18
C17orf76	-0.07642	8.32E-02	1.17E-01
C17orf77	0.081363	6.50E-02	9.38E-02
C17orf78	0.029043	5.11E-01	5.75E-01
C17orf79	0.321118	8.12E-14	5.31E-13
C17orf80	0.228662	1.55E-07	5.19E-07
C17orf81	0.088065	4.58E-02	6.81E-02
C17orf82	-0.13416	2.28E-03	4.32E-03
C17orf85	0.023457	5.95E-01	6.53E-01
C17orf86	-0.01769	6.89E-01	7.38E-01
C17orf87	-0.07573	8.60E-02	1.20E-01
C17orf88	-0.05976	1.76E-01	2.28E-01
C17orf89	0.161023	2.43E-04	5.37E-04
C17orf90	0.016313	7.12E-01	7.59E-01
C17orf91	-0.42354	7.81E-24	1.26E-22
C17orf93	0.20477	2.79E-06	8.02E-06

C17orf95	0.134477	2.23E-03	4.22E-03
C17orf96	0.406866	5.94E-22	8.15E-21
C17orf97	-0.28285	6.27E-11	3.04E-10
C17orf98	-0.02517	5.69E-01	6.29E-01
C17orf99	0.069675	1.14E-01	1.55E-01
C18orf10	0.032535	4.61E-01	5.27E-01
C18orf16	-0.32188	7.04E-14	4.63E-13
C18orf18	0.028059	5.25E-01	5.89E-01
C18orf19	0.443069	3.56E-26	6.95E-25
C18orf1	-0.37455	1.35E-18	1.38E-17
C18orf20	-0.12023	6.30E-03	1.11E-02
C18orf21	0.149428	6.69E-04	1.38E-03
C18orf22	0.016677	7.06E-01	7.54E-01
C18orf25	0.046983	2.87E-01	3.50E-01
C18orf26	0.044847	3.10E-01	3.74E-01
C18orf2	0.244858	1.81E-08	6.70E-08
C18orf32	-0.03867	3.81E-01	4.48E-01
C18orf34	-0.05965	1.77E-01	2.29E-01
C18orf45	-0.01117	8.00E-01	8.35E-01
C18orf54	0.599728	1.29E-51	9.02E-50
C18orf55	0.24104	3.05E-08	1.10E-07
C18orf56	0.494445	4.18E-33	1.29E-31
C18orf62	0.136761	1.87E-03	3.59E-03
C18orf8	0.075181	8.83E-02	1.23E-01
C19orf10	0.101286	2.15E-02	3.42E-02
C19orf12	0.134228	2.27E-03	4.30E-03
C19orf18	-0.21408	9.42E-07	2.86E-06
C19orf20	-0.0416	3.46E-01	4.12E-01
C19orf21	-0.04423	3.16E-01	3.81E-01
C19orf22	0.067907	1.24E-01	1.67E-01
C19orf23	0.158254	3.12E-04	6.76E-04
C19orf24	0.065413	1.38E-01	1.84E-01
C19orf25	-0.06246	1.57E-01	2.06E-01
C19orf26	0.240326	3.36E-08	1.21E-07
C19orf28	0.263627	1.23E-09	5.21E-09
C19orf29	0.020511	6.42E-01	6.97E-01
C19orf2	0.118203	7.25E-03	1.26E-02
C19orf30	0.128807	3.41E-03	6.29E-03
C19orf33	-0.07363	9.51E-02	1.32E-01
C19orf34	-0.09951	2.39E-02	3.77E-02
C19orf35	-0.20191	3.86E-06	1.09E-05
C19orf36	-0.28767	2.86E-11	1.44E-10
C19orf38	-0.0014	9.75E-01	9.81E-01
C19orf39	-0.18879	1.61E-05	4.21E-05

C19orf40	0.569057	1.58E-45	9.40E-44
C19orf41	-0.10211	2.05E-02	3.27E-02
C19orf42	-0.14998	6.39E-04	1.32E-03
C19orf43	0.022036	6.18E-01	6.74E-01
C19orf44	-0.31029	5.89E-13	3.54E-12
C19orf45	0.107745	1.44E-02	2.38E-02
C19orf46	-0.08833	4.51E-02	6.72E-02
C19orf47	0.319143	1.17E-13	7.54E-13
C19orf48	0.496338	2.20E-33	6.90E-32
C19orf50	0.097359	2.72E-02	4.23E-02
C19orf51	-0.12136	5.82E-03	1.03E-02
C19orf52	0.073925	9.38E-02	1.30E-01
C19orf53	0.118132	7.28E-03	1.27E-02
C19orf54	-0.03448	4.35E-01	5.01E-01
C19orf55	0.200157	4.70E-06	1.32E-05
C19orf56	-0.18489	2.42E-05	6.15E-05
C19orf57	0.242428	2.53E-08	9.19E-08
C19orf59	-0.15597	3.81E-04	8.16E-04
C19orf60	-0.07882	7.39E-02	1.05E-01
C19orf61	0.08898	4.36E-02	6.50E-02
C19orf62	0.134673	2.19E-03	4.17E-03
C19orf63	-0.19622	7.27E-06	1.98E-05
C19orf66	-0.20829	1.86E-06	5.47E-06
C19orf69	-0.15439	4.38E-04	9.27E-04
C19orf6	-0.10237	2.01E-02	3.22E-02
C19orf70	-0.00293	9.47E-01	9.58E-01
C19orf71	-0.06851	1.20E-01	1.63E-01
C19orf73	0.013349	7.62E-01	8.02E-01
C19orf75	-0.03559	4.20E-01	4.86E-01
C19orf76	-0.02223	6.15E-01	6.71E-01
C19orf77	0.098374	2.56E-02	4.00E-02
C1D	0.16728	1.37E-04	3.12E-04
C1GALT1C1	0.044449	3.14E-01	3.79E-01
C1GALT1	0.256196	3.67E-09	1.47E-08
C1QA	0.041691	3.45E-01	4.11E-01
C1QBP	0.21811	5.79E-07	1.81E-06
C1QB	0.066361	1.33E-01	1.77E-01
C1QC	0.093641	3.36E-02	5.14E-02
C1QL1	0.366695	7.77E-18	7.45E-17
C1QL2	-0.15524	4.07E-04	8.65E-04
C1QL3	-0.04647	2.93E-01	3.55E-01
C1QL4	0.437221	1.86E-25	3.40E-24
C1QTNF1	-0.04915	2.66E-01	3.27E-01
C1QTNF2	-0.2201	4.54E-07	1.44E-06

C1QTNF3	-0.12138	5.81E-03	1.03E-02
C1QTNF4	-0.0961	2.92E-02	4.52E-02
C1QTNF6	0.36888	4.80E-18	4.68E-17
C1QTNF7	-0.61635	3.35E-55	2.54E-53
C1QTNF8	-0.16045	2.56E-04	5.63E-04
C1QTNF9B	-0.30311	2.10E-12	1.20E-11
C1QTNF9	-0.32618	3.13E-14	2.13E-13
C1RL	-0.15714	3.44E-04	7.41E-04
C1R	-0.07668	8.21E-02	1.16E-01
C1S	-0.06171	1.62E-01	2.12E-01
C1orf100	-0.06297	1.54E-01	2.02E-01
C1orf101	-0.51667	1.73E-36	6.50E-35
C1orf103	0.299599	3.85E-12	2.13E-11
C1orf104	-0.12655	4.02E-03	7.33E-03
C1orf105	0.275699	1.95E-10	9.04E-10
C1orf106	-0.09245	3.60E-02	5.46E-02
C1orf107	0.038445	3.84E-01	4.50E-01
C1orf109	0.233899	7.88E-08	2.72E-07
C1orf110	-0.20161	4.00E-06	1.13E-05
C1orf111	-0.16293	2.05E-04	4.56E-04
C1orf112	0.657739	3.94E-65	3.81E-63
C1orf113	-0.03857	3.82E-01	4.49E-01
C1orf114	-0.04199	3.42E-01	4.07E-01
C1orf115	-0.10641	1.57E-02	2.57E-02
C1orf116	-0.59236	4.29E-50	2.91E-48
C1orf122	0.153258	4.83E-04	1.01E-03
C1orf123	-0.14442	1.01E-03	2.04E-03
C1orf124	0.169792	1.08E-04	2.51E-04
C1orf125	-0.04591	2.98E-01	3.62E-01
C1orf126	-0.03242	4.63E-01	5.29E-01
C1orf127	-0.30795	8.95E-13	5.28E-12
C1orf128	-0.13934	1.52E-03	2.97E-03
C1orf129	-0.29026	1.87E-11	9.56E-11
C1orf130	-0.26928	5.26E-10	2.32E-09
C1orf131	0.175897	5.99E-05	1.44E-04
C1orf133	-0.38846	5.38E-20	6.28E-19
C1orf135	0.807575	9.94E-120	2.31E-117
C1orf141	-0.23677	5.40E-08	1.89E-07
C1orf144	0.208075	1.91E-06	5.60E-06
C1orf146	-0.04162	3.46E-01	4.12E-01
C1orf14	0.098375	2.56E-02	4.00E-02
C1orf150	-0.2623	1.50E-09	6.29E-09
C1orf151	0.151595	5.57E-04	1.16E-03
C1orf152	-0.33685	3.95E-15	2.91E-14

C1orf156	-0.02527	5.67E-01	6.27E-01
C1orf157	0.058621	1.84E-01	2.37E-01
C1orf158	-0.29506	8.35E-12	4.44E-11
C1orf159	0.111068	1.17E-02	1.96E-02
C1orf161	-0.08043	6.82E-02	9.79E-02
C1orf162	-0.13039	3.03E-03	5.64E-03
C1orf163	0.471846	6.53E-30	1.67E-28
C1orf168	-0.29663	6.40E-12	3.45E-11
C1orf170	-0.0948	3.15E-02	4.84E-02
C1orf172	-0.06951	1.15E-01	1.56E-01
C1orf173	-0.25958	2.24E-09	9.19E-09
C1orf174	0.297067	5.94E-12	3.22E-11
C1orf175	-0.29791	5.14E-12	2.80E-11
C1orf177	0.024934	5.72E-01	6.32E-01
C1orf180	0.095397	3.04E-02	4.69E-02
C1orf182	0.298153	4.93E-12	2.70E-11
C1orf183	-0.27612	1.83E-10	8.48E-10
C1orf185	-0.00681	8.77E-01	9.01E-01
C1orf186	-0.42622	3.81E-24	6.31E-23
C1orf187	0.275845	1.91E-10	8.85E-10
C1orf189	-0.23279	9.11E-08	3.12E-07
C1orf190	-0.25104	7.66E-09	2.96E-08
C1orf192	-0.25621	3.66E-09	1.47E-08
C1orf194	-0.26801	6.38E-10	2.79E-09
C1orf198	-0.40918	3.30E-22	4.62E-21
C1orf200	-0.04681	2.89E-01	3.52E-01
C1orf201	-0.18639	2.07E-05	5.32E-05
C1orf203	-0.2943	9.49E-12	5.02E-11
C1orf204	-0.27061	4.29E-10	1.92E-09
C1orf210	-0.34568	6.69E-16	5.35E-15
C1orf212	0.237189	5.11E-08	1.79E-07
C1orf213	-0.13715	1.81E-03	3.50E-03
C1orf216	0.128586	3.47E-03	6.39E-03
C1orf21	-0.46337	8.96E-29	2.12E-27
C1orf220	0.055153	2.11E-01	2.68E-01
C1orf223	-0.09518	3.08E-02	4.75E-02
C1orf226	-0.19755	6.28E-06	1.73E-05
C1orf227	-0.10728	1.49E-02	2.45E-02
C1orf228	-0.19898	5.36E-06	1.49E-05
C1orf229	-0.19101	1.27E-05	3.36E-05
C1orf230	-0.24716	1.32E-08	4.95E-08
C1orf25	-0.15347	4.74E-04	9.98E-04
C1orf26	-0.2182	5.73E-07	1.79E-06
C1orf27	-0.15275	5.05E-04	1.06E-03

C1orf31	0.278664	1.22E-10	5.77E-10
C1orf35	-0.02765	5.31E-01	5.94E-01
C1orf38	0.007896	8.58E-01	8.84E-01
C1orf43	0.102828	1.96E-02	3.14E-02
C1orf49	-0.00617	8.89E-01	9.10E-01
C1orf50	-0.04798	2.77E-01	3.39E-01
C1orf51	0.008903	8.40E-01	8.69E-01
C1orf52	0.193754	9.50E-06	2.55E-05
C1orf53	-0.16132	2.37E-04	5.23E-04
C1orf54	0.023142	6.00E-01	6.58E-01
C1orf55	0.041935	3.42E-01	4.08E-01
C1orf56	-0.10949	1.29E-02	2.15E-02
C1orf57	0.021742	6.23E-01	6.79E-01
C1orf58	0.063678	1.49E-01	1.97E-01
C1orf59	0.264342	1.11E-09	4.72E-09
C1orf61	0.370056	3.69E-18	3.65E-17
C1orf63	-0.21417	9.32E-07	2.83E-06
C1orf64	-0.1367	1.88E-03	3.61E-03
C1orf65	-0.08526	5.32E-02	7.79E-02
C1orf66	-0.24234	2.56E-08	9.30E-08
C1orf68	0.075086	8.87E-02	1.24E-01
C1orf69	-0.32167	7.33E-14	4.81E-13
C1orf70	-0.15246	5.17E-04	1.08E-03
C1orf74	0.273389	2.80E-10	1.28E-09
C1orf77	0.011044	8.03E-01	8.37E-01
C1orf83	0.121287	5.85E-03	1.04E-02
C1orf84	-0.02525	5.67E-01	6.27E-01
C1orf85	0.124527	4.65E-03	8.40E-03
C1orf86	-0.07694	8.11E-02	1.14E-01
C1orf87	-0.32458	4.24E-14	2.85E-13
C1orf88	-0.44531	1.88E-26	3.71E-25
C1orf89	-0.26395	1.17E-09	4.98E-09
C1orf91	-0.06637	1.33E-01	1.77E-01
C1orf92	-0.29656	6.48E-12	3.48E-11
C1orf93	-0.1865	2.05E-05	5.26E-05
C1orf94	0.061471	1.64E-01	2.14E-01
C1orf95	-0.19914	5.27E-06	1.46E-05
C1orf96	0.445641	1.71E-26	3.38E-25
C1orf97	-0.06718	1.28E-01	1.72E-01
C1orf9	0.190158	1.40E-05	3.66E-05
C20orf103	-0.29675	6.27E-12	3.38E-11
C20orf106	-0.07165	1.04E-01	1.43E-01
C20orf107	0.021525	6.26E-01	6.82E-01
C20orf108	-0.41814	3.26E-23	4.97E-22

C20orf111	0.239516	3.74E-08	1.34E-07
C20orf112	-0.10961	1.28E-02	2.13E-02
C20orf114	-0.19234	1.11E-05	2.94E-05
C20orf117	0.057202	1.95E-01	2.49E-01
C20orf118	0.089831	4.16E-02	6.23E-02
C20orf11	0.057409	1.93E-01	2.48E-01
C20orf123	-0.1577	3.27E-04	7.08E-04
C20orf12	-0.22196	3.61E-07	1.16E-06
C20orf132	-0.35586	8.07E-17	7.04E-16
C20orf134	-0.17161	9.08E-05	2.13E-04
C20orf135	-0.1276	3.73E-03	6.83E-03
C20orf141	0.337201	3.68E-15	2.72E-14
C20orf144	0.038547	3.83E-01	4.49E-01
C20orf151	-0.20067	4.44E-06	1.25E-05
C20orf152	-0.00933	8.33E-01	8.62E-01
C20orf160	-0.33182	1.06E-14	7.50E-14
C20orf165	0.00659	8.81E-01	9.04E-01
C20orf166	-0.05872	1.83E-01	2.36E-01
C20orf173	0.050353	2.54E-01	3.15E-01
C20orf177	0.076399	8.33E-02	1.17E-01
C20orf185	0.036249	4.12E-01	4.78E-01
C20orf186	-0.03985	3.67E-01	4.33E-01
C20orf191	0.071808	1.04E-01	1.42E-01
C20orf194	-0.41165	1.76E-22	2.51E-21
C20orf195	-0.08876	4.41E-02	6.58E-02
C20orf196	0.054616	2.16E-01	2.73E-01
C20orf197	-0.2073	2.09E-06	6.09E-06
C20orf199	0.090792	3.94E-02	5.94E-02
C20orf200	-0.32018	9.67E-14	6.27E-13
C20orf201	-0.05727	1.94E-01	2.49E-01
C20orf202	-0.18438	2.55E-05	6.46E-05
C20orf203	-0.01078	8.07E-01	8.41E-01
C20orf20	0.434565	3.89E-25	6.93E-24
C20orf24	0.422337	1.08E-23	1.72E-22
C20orf26	-0.23985	3.58E-08	1.28E-07
C20orf27	0.19457	8.69E-06	2.35E-05
C20orf29	-0.07132	1.06E-01	1.45E-01
C20orf30	0.023784	5.90E-01	6.48E-01
C20orf3	-0.02284	6.05E-01	6.62E-01
C20orf43	0.107815	1.44E-02	2.37E-02
C20orf46	-0.05275	2.32E-01	2.90E-01
C20orf4	-0.05043	2.53E-01	3.14E-01
C20orf54	-0.13557	2.05E-03	3.92E-03
C20orf56	-0.47311	4.38E-30	1.13E-28

C20orf70	-0.01743	6.93E-01	7.42E-01
C20orf71	0.028672	5.16E-01	5.80E-01
C20orf72	0.348659	3.63E-16	2.97E-15
C20orf7	0.176474	5.66E-05	1.37E-04
C20orf85	-0.28344	5.70E-11	2.77E-10
C20orf94	0.006686	8.80E-01	9.02E-01
C20orf96	-0.2212	3.97E-07	1.27E-06
C21orf119	0.056897	1.97E-01	2.52E-01
C21orf121	-0.18416	2.61E-05	6.60E-05
C21orf122	-0.22199	3.60E-07	1.15E-06
C21orf125	-0.01445	7.44E-01	7.87E-01
C21orf128	-0.17481	6.66E-05	1.59E-04
C21orf129	-0.10437	1.78E-02	2.89E-02
C21orf130	-0.15713	3.44E-04	7.42E-04
C21orf131	-0.02583	5.59E-01	6.20E-01
C21orf15	-0.26787	6.52E-10	2.85E-09
C21orf29	-0.25555	4.02E-09	1.61E-08
C21orf2	-0.50032	5.64E-34	1.84E-32
C21orf33	-0.18182	3.31E-05	8.25E-05
C21orf34	-0.40814	4.30E-22	5.97E-21
C21orf45	0.625867	2.36E-57	1.94E-55
C21orf49	-0.33476	5.97E-15	4.34E-14
C21orf54	-0.06213	1.59E-01	2.08E-01
C21orf56	0.066665	1.31E-01	1.75E-01
C21orf57	-0.09691	2.79E-02	4.33E-02
C21orf58	0.097527	2.69E-02	4.19E-02
C21orf59	-0.05598	2.05E-01	2.60E-01
C21orf62	-0.24482	1.82E-08	6.74E-08
C21orf63	-0.44156	5.48E-26	1.05E-24
C21orf67	-0.0618	1.61E-01	2.11E-01
C21orf70	0.278129	1.33E-10	6.26E-10
C21orf71	-0.26582	8.88E-10	3.82E-09
C21orf7	-0.11743	7.64E-03	1.32E-02
C21orf81	-0.19725	6.49E-06	1.78E-05
C21orf82	-0.30284	2.20E-12	1.25E-11
C21orf84	-0.05679	1.98E-01	2.53E-01
C21orf88	0.035225	4.25E-01	4.91E-01
C21orf90	-0.18892	1.59E-05	4.15E-05
C21orf91	0.05923	1.80E-01	2.32E-01
C21orf94	-0.04635	2.94E-01	3.57E-01
C21orf96	0.017617	6.90E-01	7.39E-01
C21orf99	0.086409	5.00E-02	7.38E-02
C22orf13	-0.14356	1.09E-03	2.17E-03
C22orf15	-0.26012	2.07E-09	8.53E-09

C22orf23	-0.17861	4.58E-05	1.12E-04
C22orf24	0.042745	3.33E-01	3.98E-01
C22orf25	-0.11902	6.85E-03	1.20E-02
C22orf26	-0.12852	3.48E-03	6.41E-03
C22orf27	-0.06875	1.19E-01	1.61E-01
C22orf28	0.19786	6.07E-06	1.67E-05
C22orf29	-0.2573	3.12E-09	1.26E-08
C22orf30	0.053893	2.22E-01	2.80E-01
C22orf31	-0.18318	2.89E-05	7.25E-05
C22orf32	-0.3351	5.58E-15	4.07E-14
C22orf33	-0.13162	2.76E-03	5.17E-03
C22orf34	-0.10609	1.60E-02	2.62E-02
C22orf36	-0.40668	6.22E-22	8.53E-21
C22orf39	-0.19787	6.06E-06	1.67E-05
C22orf40	-0.15326	4.83E-04	1.01E-03
C22orf41	-0.07584	8.55E-02	1.20E-01
C22orf42	-0.14499	9.67E-04	1.95E-03
C22orf43	-0.07795	7.72E-02	1.09E-01
C22orf45	-0.14858	7.19E-04	1.48E-03
C22orf46	-0.15481	4.22E-04	8.96E-04
C22orf9	0.068255	1.22E-01	1.64E-01
C2CD2L	-0.34786	4.28E-16	3.48E-15
C2CD2	-0.20671	2.24E-06	6.49E-06
C2CD3	0.087224	4.79E-02	7.08E-02
C2CD4A	0.248403	1.11E-08	4.20E-08
C2CD4B	-0.0015	9.73E-01	9.79E-01
C2CD4C	0.073822	9.42E-02	1.31E-01
C2CD4D	0.000442	9.92E-01	9.94E-01
C2orf14	0.051526	2.43E-01	3.03E-01
C2orf15	0.01105	8.02E-01	8.37E-01
C2orf16	0.159348	2.83E-04	6.17E-04
C2orf18	0.31693	1.76E-13	1.12E-12
C2orf24	-0.38695	7.69E-20	8.83E-19
C2orf27A	0.073695	9.48E-02	1.31E-01
C2orf28	0.038597	3.82E-01	4.49E-01
C2orf29	0.197428	6.36E-06	1.75E-05
C2orf34	-0.21387	9.66E-07	2.93E-06
C2orf39	-0.17813	4.80E-05	1.17E-04
C2orf3	0.266707	7.77E-10	3.37E-09
C2orf40	-0.49893	9.06E-34	2.92E-32
C2orf42	-0.06131	1.65E-01	2.15E-01
C2orf43	0.298591	4.58E-12	2.51E-11
C2orf44	0.381882	2.52E-19	2.77E-18
C2orf47	0.344413	8.67E-16	6.84E-15

C2orf48	0.397521	6.06E-21	7.68E-20
C2orf49	0.440475	7.44E-26	1.41E-24
C2orf50	-0.10497	1.72E-02	2.79E-02
C2orf51	0.14513	9.57E-04	1.93E-03
C2orf52	0.141525	1.28E-03	2.53E-03
C2orf53	0.038518	3.83E-01	4.49E-01
C2orf54	-0.19034	1.37E-05	3.59E-05
C2orf55	-0.26872	5.73E-10	2.52E-09
C2orf56	0.134197	2.27E-03	4.31E-03
C2orf57	-0.04559	3.02E-01	3.65E-01
C2orf58	-0.4512	3.40E-27	7.14E-26
C2orf60	0.080698	6.73E-02	9.67E-02
C2orf61	0.092084	3.67E-02	5.56E-02
C2orf62	-0.23573	6.20E-08	2.16E-07
C2orf63	-0.34883	3.51E-16	2.87E-15
C2orf64	-0.01931	6.62E-01	7.14E-01
C2orf65	-0.13941	1.52E-03	2.96E-03
C2orf66	-0.03294	4.56E-01	5.22E-01
C2orf67	-0.34254	1.27E-15	9.78E-15
C2orf68	-0.05292	2.31E-01	2.89E-01
C2orf69	0.413458	1.10E-22	1.60E-21
C2orf70	-0.00757	8.64E-01	8.89E-01
C2orf71	-0.36539	1.03E-17	9.82E-17
C2orf72	0.159444	2.80E-04	6.12E-04
C2orf73	-0.27836	1.28E-10	6.03E-10
C2orf74	-0.23093	1.16E-07	3.94E-07
C2orf76	0.112034	1.10E-02	1.85E-02
C2orf77	-0.15619	3.74E-04	8.01E-04
C2orf78	0.207382	2.07E-06	6.03E-06
C2orf79	-0.05538	2.10E-01	2.66E-01
C2orf7	0.177924	4.90E-05	1.19E-04
C2orf80	0.148692	7.12E-04	1.46E-03
C2orf81	-0.14746	7.89E-04	1.61E-03
C2orf82	0.17993	4.01E-05	9.87E-05
C2orf83	0.132974	2.50E-03	4.70E-03
C2orf84	0.075054	8.88E-02	1.24E-01
C2orf85	-0.11113	1.16E-02	1.95E-02
C2orf86	-0.05685	1.98E-01	2.52E-01
C2orf88	0.051962	2.39E-01	2.99E-01
C2orf89	-0.15802	3.18E-04	6.89E-04
C2	-0.32331	5.38E-14	3.58E-13
C3AR1	-0.00809	8.55E-01	8.81E-01
C3P1	0.141322	1.30E-03	2.57E-03
C3orf10	-0.01678	7.04E-01	7.52E-01

C3orf14	0.035306	4.24E-01	4.90E-01
C3orf15	-0.29439	9.35E-12	4.95E-11
C3orf16	-0.16803	1.27E-04	2.93E-04
C3orf17	0.249047	1.01E-08	3.86E-08
C3orf18	-0.40972	2.88E-22	4.05E-21
C3orf19	-0.31382	3.11E-13	1.93E-12
C3orf1	0.28557	4.03E-11	1.98E-10
C3orf20	0.071563	1.05E-01	1.43E-01
C3orf21	0.355159	9.35E-17	8.12E-16
C3orf22	-0.01164	7.92E-01	8.28E-01
C3orf23	-0.14601	8.89E-04	1.80E-03
C3orf24	-0.01432	7.46E-01	7.89E-01
C3orf26	0.514032	4.50E-36	1.65E-34
C3orf27	-0.02507	5.70E-01	6.30E-01
C3orf30	-0.00977	8.25E-01	8.56E-01
C3orf31	-0.08687	4.88E-02	7.21E-02
C3orf32	-0.16082	2.48E-04	5.46E-04
C3orf33	0.019105	6.65E-01	7.17E-01
C3orf34	0.142023	1.23E-03	2.44E-03
C3orf35	-0.24604	1.54E-08	5.74E-08
C3orf36	-0.24145	2.88E-08	1.04E-07
C3orf37	0.357106	6.19E-17	5.48E-16
C3orf38	0.216274	7.24E-07	2.23E-06
C3orf39	0.102264	2.03E-02	3.24E-02
C3orf42	-0.32047	9.16E-14	5.96E-13
C3orf43	0.075577	8.66E-02	1.21E-01
C3orf45	-0.08766	4.68E-02	6.94E-02
C3orf47	-0.30868	7.85E-13	4.66E-12
C3orf48	-0.0979	2.63E-02	4.11E-02
C3orf49	-0.08395	5.69E-02	8.30E-02
C3orf50	-0.03703	4.02E-01	4.68E-01
C3orf51	-0.05084	2.49E-01	3.10E-01
C3orf52	-0.02864	5.17E-01	5.81E-01
C3orf54	-0.25858	2.59E-09	1.06E-08
C3orf55	-0.03627	4.11E-01	4.78E-01
C3orf57	0.142842	1.15E-03	2.29E-03
C3orf58	-0.01432	7.46E-01	7.89E-01
C3orf59	0.249924	8.96E-09	3.43E-08
C3orf62	-0.41566	6.24E-23	9.26E-22
C3orf63	0.033363	4.50E-01	5.16E-01
C3orf64	-0.10386	1.84E-02	2.97E-02
C3orf65	0.172058	8.69E-05	2.04E-04
C3orf66	0.200661	4.45E-06	1.25E-05
C3orf67	0.344813	7.99E-16	6.34E-15

C3orf70	-0.13901	1.57E-03	3.05E-03
C3orf71	-0.2136	9.97E-07	3.02E-06
C3orf72	0.224995	2.47E-07	8.09E-07
C3orf74	0.064996	1.41E-01	1.87E-01
C3orf75	0.092428	3.60E-02	5.47E-02
C3orf77	0.088598	4.45E-02	6.63E-02
C3orf79	-0.00874	8.43E-01	8.72E-01
C3	-0.36166	2.33E-17	2.16E-16
C4A	-0.43525	3.22E-25	5.78E-24
C4BPA	-0.41223	1.51E-22	2.18E-21
C4BPB	-0.01389	7.53E-01	7.95E-01
C4orf10	-0.18499	2.40E-05	6.09E-05
C4orf12	-0.27207	3.43E-10	1.55E-09
C4orf14	-0.00917	8.36E-01	8.65E-01
C4orf17	0.059125	1.80E-01	2.33E-01
C4orf19	-0.18308	2.92E-05	7.32E-05
C4orf21	0.437957	1.51E-25	2.79E-24
C4orf22	-0.19351	9.75E-06	2.61E-05
C4orf23	-0.21458	8.87E-07	2.70E-06
C4orf26	0.238453	4.32E-08	1.53E-07
C4orf27	0.185438	2.29E-05	5.83E-05
C4orf29	0.083381	5.86E-02	8.53E-02
C4orf31	-0.43025	1.28E-24	2.18E-23
C4orf32	-0.016	7.17E-01	7.64E-01
C4orf33	-0.04071	3.56E-01	4.22E-01
C4orf34	-0.09993	2.33E-02	3.69E-02
C4orf35	0.085383	5.28E-02	7.75E-02
C4orf36	0.055515	2.08E-01	2.64E-01
C4orf37	-0.09993	2.33E-02	3.69E-02
C4orf38	-0.15857	3.03E-04	6.58E-04
C4orf39	-0.12465	4.61E-03	8.33E-03
C4orf3	-0.00562	8.99E-01	9.18E-01
C4orf40	0.06403	1.47E-01	1.94E-01
C4orf41	-0.10568	1.64E-02	2.68E-02
C4orf42	-0.07298	9.80E-02	1.35E-01
C4orf43	0.417325	4.04E-23	6.11E-22
C4orf44	-0.27903	1.15E-10	5.45E-10
C4orf45	-0.09602	2.93E-02	4.54E-02
C4orf46	0.622506	1.38E-56	1.11E-54
C4orf47	-0.02558	5.62E-01	6.23E-01
C4orf48	0.1915	1.21E-05	3.20E-05
C4orf49	-0.01953	6.58E-01	7.11E-01
C4orf50	0.109632	1.28E-02	2.13E-02
C4orf51	0.134382	2.24E-03	4.25E-03

C4orf52	0.041443	3.48E-01	4.14E-01
C4orf6	0.093026	3.48E-02	5.30E-02
C4orf7	-0.20174	3.94E-06	1.11E-05
C5AR1	0.015933	7.18E-01	7.65E-01
C5orf13	-0.07089	1.08E-01	1.48E-01
C5orf15	0.1468	8.33E-04	1.69E-03
C5orf20	-0.15604	3.79E-04	8.11E-04
C5orf22	0.330159	1.46E-14	1.02E-13
C5orf23	-0.2245	2.63E-07	8.58E-07
C5orf24	-0.06101	1.67E-01	2.17E-01
C5orf25	-0.04926	2.65E-01	3.26E-01
C5orf27	-0.06502	1.41E-01	1.87E-01
C5orf28	0.238571	4.25E-08	1.51E-07
C5orf30	0.116786	7.98E-03	1.38E-02
C5orf32	-0.1958	7.61E-06	2.07E-05
C5orf33	0.205441	2.59E-06	7.45E-06
C5orf34	0.678705	8.73E-71	9.28E-69
C5orf35	0.019543	6.58E-01	7.11E-01
C5orf36	-0.12828	3.54E-03	6.52E-03
C5orf38	-0.43704	1.96E-25	3.58E-24
C5orf39	-0.11385	9.71E-03	1.65E-02
C5orf40	0.051193	2.46E-01	3.06E-01
C5orf41	-0.39708	6.76E-21	8.51E-20
C5orf42	-0.10442	1.78E-02	2.88E-02
C5orf43	0.174128	7.12E-05	1.70E-04
C5orf44	0.062445	1.57E-01	2.06E-01
C5orf45	-0.41496	7.48E-23	1.10E-21
C5orf46	0.370375	3.44E-18	3.41E-17
C5orf47	-0.07658	8.25E-02	1.16E-01
C5orf48	0.070489	1.10E-01	1.50E-01
C5orf49	-0.43942	1.00E-25	1.88E-24
C5orf4	-0.22215	3.53E-07	1.13E-06
C5orf51	0.125581	4.31E-03	7.83E-03
C5orf52	-0.05805	1.88E-01	2.42E-01
C5orf53	-0.53217	5.34E-39	2.33E-37
C5orf54	-0.03004	4.96E-01	5.61E-01
C5orf55	0.028434	5.20E-01	5.84E-01
C5orf56	-0.17073	9.87E-05	2.30E-04
C5orf58	0.06429	1.45E-01	1.92E-01
C5orf60	0.031821	4.71E-01	5.37E-01
C5orf62	0.020298	6.46E-01	7.00E-01
C5	-0.21872	5.38E-07	1.69E-06
C6orf103	-0.31367	3.20E-13	1.98E-12
C6orf105	-0.0584	1.86E-01	2.39E-01

C6orf106	-0.15865	3.01E-04	6.54E-04
C6orf108	-0.01559	7.24E-01	7.70E-01
C6orf10	0.049976	2.58E-01	3.19E-01
C6orf114	-0.02438	5.81E-01	6.40E-01
C6orf115	0.378811	5.11E-19	5.44E-18
C6orf118	-0.26573	9.00E-10	3.87E-09
C6orf120	0.256078	3.73E-09	1.49E-08
C6orf122	0.068503	1.21E-01	1.63E-01
C6orf123	-0.17696	5.39E-05	1.31E-04
C6orf124	-0.14067	1.37E-03	2.70E-03
C6orf125	0.074184	9.26E-02	1.29E-01
C6orf126	0.081166	6.57E-02	9.47E-02
C6orf127	0.084889	5.42E-02	7.94E-02
C6orf129	0.370692	3.21E-18	3.19E-17
C6orf130	-0.01495	7.35E-01	7.79E-01
C6orf132	-0.11972	6.53E-03	1.15E-02
C6orf134	-0.09311	3.47E-02	5.28E-02
C6orf136	0.066263	1.33E-01	1.78E-01
C6orf138	-0.39369	1.54E-20	1.88E-19
C6orf141	0.124769	4.57E-03	8.26E-03
C6orf142	0.093262	3.44E-02	5.24E-02
C6orf145	-0.11558	8.66E-03	1.48E-02
C6orf146	-0.07581	8.57E-02	1.20E-01
C6orf147	0.07935	7.20E-02	1.03E-01
C6orf150	0.560594	5.89E-44	3.30E-42
C6orf153	0.36605	8.96E-18	8.55E-17
C6orf154	-0.19083	1.30E-05	3.42E-05
C6orf155	-0.28808	2.67E-11	1.35E-10
C6orf15	0.09354	3.38E-02	5.17E-02
C6orf162	0.197909	6.03E-06	1.66E-05
C6orf163	-0.14631	8.68E-04	1.76E-03
C6orf164	-0.21503	8.40E-07	2.57E-06
C6orf165	-0.16968	1.09E-04	2.53E-04
C6orf167	0.565077	8.79E-45	5.09E-43
C6orf168	-0.1056	1.65E-02	2.69E-02
C6orf170	0.020699	6.39E-01	6.94E-01
C6orf174	-0.0688	1.19E-01	1.61E-01
C6orf176	0.076187	8.41E-02	1.18E-01
C6orf182	0.508984	2.73E-35	9.54E-34
C6orf186	-0.11564	8.62E-03	1.48E-02
C6orf191	0.038954	3.78E-01	4.44E-01
C6orf192	0.022327	6.13E-01	6.70E-01
C6orf195	0.289918	1.97E-11	1.01E-10
C6orf1	-0.13766	1.74E-03	3.37E-03

C6orf201	-0.3222	6.64E-14	4.38E-13
C6orf203	-0.14492	9.73E-04	1.96E-03
C6orf204	0.079294	7.22E-02	1.03E-01
C6orf208	-0.05763	1.92E-01	2.46E-01
C6orf211	0.058819	1.83E-01	2.35E-01
C6orf217	-0.38606	9.49E-20	1.08E-18
C6orf218	0.219452	4.92E-07	1.55E-06
C6orf221	-0.04099	3.53E-01	4.19E-01
C6orf222	-0.02194	6.19E-01	6.75E-01
C6orf223	0.108209	1.40E-02	2.32E-02
C6orf225	-0.09505	3.10E-02	4.78E-02
C6orf226	-0.08858	4.45E-02	6.64E-02
C6orf227	-0.1667	1.44E-04	3.28E-04
C6orf25	-0.14173	1.26E-03	2.49E-03
C6orf26	-0.15847	3.06E-04	6.64E-04
C6orf27	-0.22938	1.42E-07	4.77E-07
C6orf35	0.043942	3.20E-01	3.84E-01
C6orf41	-0.27676	1.65E-10	7.71E-10
C6orf47	-0.25324	5.60E-09	2.20E-08
C6orf48	-0.13371	2.36E-03	4.46E-03
C6orf52	0.085839	5.16E-02	7.58E-02
C6orf57	0.098621	2.52E-02	3.95E-02
C6orf58	-0.06358	1.50E-01	1.97E-01
C6orf59	-0.20046	4.54E-06	1.28E-05
C6orf62	-0.02731	5.36E-01	5.99E-01
C6orf64	-0.15934	2.83E-04	6.17E-04
C6orf70	-0.18699	1.95E-05	5.01E-05
C6orf72	0.040231	3.62E-01	4.29E-01
C6orf81	-0.03	4.97E-01	5.62E-01
C6orf89	-0.33123	1.19E-14	8.37E-14
C6orf94	-0.03503	4.28E-01	4.94E-01
C6orf97	-0.29804	5.03E-12	2.75E-11
C6	-0.39352	1.60E-20	1.96E-19
C7orf10	0.028628	5.17E-01	5.81E-01
C7orf11	0.33884	2.66E-15	1.99E-14
C7orf13	0.086738	4.91E-02	7.26E-02
C7orf16	-0.05786	1.90E-01	2.44E-01
C7orf23	-0.43407	4.46E-25	7.90E-24
C7orf25	0.278673	1.22E-10	5.76E-10
C7orf26	-0.0877	4.67E-02	6.93E-02
C7orf27	0.001186	9.79E-01	9.84E-01
C7orf28A	0.308069	8.75E-13	5.18E-12
C7orf28B	0.236203	5.82E-08	2.03E-07
C7orf29	0.14399	1.05E-03	2.10E-03

C7orf30	0.187707	1.81E-05	4.68E-05
C7orf31	-0.0746	9.08E-02	1.26E-01
C7orf33	0.105678	1.64E-02	2.68E-02
C7orf34	-0.03721	3.99E-01	4.66E-01
C7orf36	0.147681	7.75E-04	1.58E-03
C7orf40	0.187564	1.83E-05	4.74E-05
C7orf41	-0.55357	1.09E-42	5.77E-41
C7orf42	-0.12529	4.41E-03	7.98E-03
C7orf43	0.056681	1.99E-01	2.54E-01
C7orf44	0.262912	1.37E-09	5.77E-09
C7orf45	-0.11735	7.68E-03	1.33E-02
C7orf46	-0.09128	3.84E-02	5.79E-02
C7orf47	0.156473	3.65E-04	7.83E-04
C7orf49	0.470854	8.90E-30	2.25E-28
C7orf4	-0.05629	2.02E-01	2.58E-01
C7orf50	0.055364	2.10E-01	2.66E-01
C7orf51	0.16489	1.71E-04	3.85E-04
C7orf52	0.129907	3.14E-03	5.83E-03
C7orf53	0.0285	5.19E-01	5.83E-01
C7orf54	-0.08695	4.86E-02	7.18E-02
C7orf55	-0.06906	1.18E-01	1.59E-01
C7orf57	-0.29317	1.15E-11	6.01E-11
C7orf58	-0.29268	1.25E-11	6.49E-11
C7orf59	0.02661	5.47E-01	6.09E-01
C7orf60	0.005166	9.07E-01	9.25E-01
C7orf61	0.219438	4.93E-07	1.55E-06
C7orf63	-0.2515	7.17E-09	2.78E-08
C7orf64	0.069354	1.16E-01	1.57E-01
C7orf65	0.166393	1.49E-04	3.38E-04
C7orf66	0.075892	8.53E-02	1.20E-01
C7orf68	0.498236	1.15E-33	3.65E-32
C7orf69	0.083596	5.80E-02	8.44E-02
C7orf70	0.371346	2.77E-18	2.77E-17
C7orf71	0.018545	6.75E-01	7.26E-01
C7orf72	0.013082	7.67E-01	8.06E-01
C7	-0.5067	6.11E-35	2.08E-33
C8A	-0.07932	7.21E-02	1.03E-01
C8B	-0.22162	3.77E-07	1.21E-06
C8G	0.020536	6.42E-01	6.96E-01
C8ORFK29	0.098982	2.47E-02	3.88E-02
C8orf12	-0.17581	6.03E-05	1.45E-04
C8orf22	-0.08164	6.41E-02	9.26E-02
C8orf30A	0.004383	9.21E-01	9.37E-01
C8orf31	-0.01653	7.08E-01	7.56E-01

C8orf33	0.205977	2.43E-06	7.02E-06
C8orf34	-0.47713	1.23E-30	3.29E-29
C8orf37	0.089729	4.18E-02	6.27E-02
C8orf38	0.005514	9.01E-01	9.20E-01
C8orf39	0.100807	2.21E-02	3.51E-02
C8orf40	-0.06152	1.63E-01	2.13E-01
C8orf41	0.127808	3.67E-03	6.73E-03
C8orf42	-0.40786	4.62E-22	6.39E-21
C8orf44	-0.04414	3.17E-01	3.82E-01
C8orf45	0.046552	2.92E-01	3.54E-01
C8orf46	-0.25227	6.43E-09	2.51E-08
C8orf47	-0.01559	7.24E-01	7.70E-01
C8orf48	-0.26645	8.08E-10	3.50E-09
C8orf4	-0.21174	1.24E-06	3.73E-06
C8orf51	0.086977	4.85E-02	7.17E-02
C8orf55	-0.22039	4.38E-07	1.39E-06
C8orf56	-0.01639	7.11E-01	7.58E-01
C8orf58	-0.16033	2.59E-04	5.68E-04
C8orf59	0.187777	1.79E-05	4.65E-05
C8orf71	0.034005	4.41E-01	5.08E-01
C8orf73	-0.16462	1.75E-04	3.94E-04
C8orf74	0.110182	1.24E-02	2.06E-02
C8orf75	0.180767	3.69E-05	9.13E-05
C8orf76	0.353858	1.23E-16	1.05E-15
C8orf77	-0.14355	1.09E-03	2.17E-03
C8orf79	-0.3757	1.04E-18	1.08E-17
C8orf80	-0.09731	2.72E-02	4.24E-02
C8orf83	-0.1395	1.51E-03	2.94E-03
C8orf84	-0.2616	1.66E-09	6.94E-09
C8orf85	-0.32037	9.33E-14	6.06E-13
C8orf86	0.098564	2.53E-02	3.97E-02
C9orf100	0.64141	4.96E-61	4.41E-59
C9orf102	0.023177	6.00E-01	6.57E-01
C9orf103	-0.30148	2.78E-12	1.56E-11
C9orf106	-0.21563	7.83E-07	2.41E-06
C9orf109	0.186953	1.95E-05	5.03E-05
C9orf110	0.149248	6.79E-04	1.40E-03
C9orf114	0.013901	7.53E-01	7.94E-01
C9orf116	-0.18432	2.57E-05	6.49E-05
C9orf117	-0.28943	2.14E-11	1.09E-10
C9orf119	0.093548	3.38E-02	5.16E-02
C9orf11	-0.05786	1.90E-01	2.44E-01
C9orf122	-0.07322	9.69E-02	1.34E-01
C9orf123	0.079423	7.17E-02	1.02E-01

C9orf125	-0.08357	5.81E-02	8.45E-02
C9orf128	-0.28667	3.37E-11	1.67E-10
C9orf129	0.025962	5.57E-01	6.18E-01
C9orf130	-0.32327	5.42E-14	3.61E-13
C9orf131	-0.24709	1.33E-08	4.99E-08
C9orf135	-0.32359	5.11E-14	3.40E-13
C9orf139	-0.15561	3.94E-04	8.40E-04
C9orf140	0.677417	2.00E-70	2.11E-68
C9orf142	-0.03145	4.76E-01	5.42E-01
C9orf144B	-0.10423	1.80E-02	2.91E-02
C9orf144	-0.07827	7.60E-02	1.08E-01
C9orf150	-0.19172	1.18E-05	3.13E-05
C9orf152	-0.43782	1.57E-25	2.89E-24
C9orf153	-0.02555	5.63E-01	6.23E-01
C9orf156	-0.36	3.33E-17	3.05E-16
C9orf163	-0.06264	1.56E-01	2.04E-01
C9orf167	-0.19086	1.29E-05	3.41E-05
C9orf169	0.00328	9.41E-01	9.54E-01
C9orf16	0.032953	4.56E-01	5.22E-01
C9orf170	0.020601	6.41E-01	6.95E-01
C9orf171	-0.26542	9.42E-10	4.04E-09
C9orf172	0.027363	5.36E-01	5.98E-01
C9orf173	-0.08498	5.40E-02	7.90E-02
C9orf21	0.122406	5.41E-03	9.65E-03
C9orf23	0.094494	3.20E-02	4.92E-02
C9orf24	-0.26788	6.50E-10	2.85E-09
C9orf25	0.097808	2.64E-02	4.13E-02
C9orf27	0.0942	3.26E-02	4.99E-02
C9orf30	0.487193	4.70E-32	1.37E-30
C9orf37	-0.01406	7.50E-01	7.92E-01
C9orf3	-0.1288	3.41E-03	6.29E-03
C9orf40	0.55463	7.08E-43	3.76E-41
C9orf41	0.161669	2.29E-04	5.08E-04
C9orf43	0.004384	9.21E-01	9.37E-01
C9orf44	-0.17562	6.15E-05	1.48E-04
C9orf45	-0.32154	7.51E-14	4.92E-13
C9orf46	0.237384	4.98E-08	1.75E-07
C9orf47	-0.14545	9.32E-04	1.88E-03
C9orf4	0.19779	6.11E-06	1.68E-05
C9orf50	-0.13938	1.52E-03	2.97E-03
C9orf53	0.158034	3.18E-04	6.89E-04
C9orf57	0.041657	3.45E-01	4.11E-01
C9orf5	-0.26354	1.25E-09	5.28E-09
C9orf64	-0.00094	9.83E-01	9.87E-01

C9orf66	-0.23012	1.29E-07	4.35E-07
C9orf68	-0.42495	5.35E-24	8.77E-23
C9orf69	0.154949	4.17E-04	8.86E-04
C9orf6	0.140054	1.44E-03	2.82E-03
C9orf70	-0.03173	4.72E-01	5.38E-01
C9orf71	-0.18524	2.33E-05	5.95E-05
C9orf72	0.037596	3.95E-01	4.61E-01
C9orf78	0.023786	5.90E-01	6.48E-01
C9orf79	-0.01902	6.67E-01	7.18E-01
C9orf7	-0.3644	1.29E-17	1.21E-16
C9orf80	0.097901	2.63E-02	4.11E-02
C9orf82	0.108157	1.41E-02	2.32E-02
C9orf84	0.191896	1.16E-05	3.08E-05
C9orf85	0.032774	4.58E-01	5.24E-01
C9orf86	0.006443	8.84E-01	9.06E-01
C9orf89	0.070958	1.08E-01	1.47E-01
C9orf91	-0.2214	3.87E-07	1.24E-06
C9orf93	-0.13364	2.37E-03	4.48E-03
C9orf95	-0.27216	3.38E-10	1.53E-09
C9orf96	-0.32701	2.67E-14	1.82E-13
C9orf98	-0.36784	6.04E-18	5.82E-17
C9orf9	-0.43629	2.41E-25	4.39E-24
C9	0.148413	7.29E-04	1.50E-03
CA10	-0.30083	3.11E-12	1.74E-11
CA11	-0.02617	5.53E-01	6.15E-01
CA12	0.080419	6.82E-02	9.80E-02
CA13	-0.36407	1.38E-17	1.30E-16
CA14	-0.07729	7.97E-02	1.13E-01
CA1	-0.16746	1.34E-04	3.07E-04
CA2	-0.15319	4.86E-04	1.02E-03
CA3	-0.50917	2.56E-35	8.98E-34
CA4	-0.30943	6.87E-13	4.11E-12
CA5A	0.176922	5.41E-05	1.31E-04
CA5BP	0.025849	5.58E-01	6.20E-01
CA5B	-0.40515	9.15E-22	1.23E-20
CA6	-0.06721	1.28E-01	1.71E-01
CA7	-0.06065	1.69E-01	2.20E-01
CA8	-0.14801	7.53E-04	1.54E-03
CA9	0.163536	1.94E-04	4.33E-04
CAB39L	-0.31586	2.15E-13	1.34E-12
CAB39	0.025518	5.63E-01	6.24E-01
CABC1	-0.46525	5.04E-29	1.21E-27
CABIN1	-0.28988	1.99E-11	1.01E-10
CABLES1	-0.44962	5.39E-27	1.11E-25

CABLES2	0.197077	6.61E-06	1.81E-05
CABP1	0.003567	9.36E-01	9.49E-01
CABP2	0.05264	2.33E-01	2.92E-01
CABP4	-0.01641	7.10E-01	7.58E-01
CABP5	-0.04084	3.55E-01	4.21E-01
CABP7	-0.00697	8.75E-01	8.98E-01
CABYR	0.173046	7.90E-05	1.87E-04
CACHD1	-0.33014	1.46E-14	1.02E-13
CACNA1A	0.129773	3.17E-03	5.89E-03
CACNA1B	0.040767	3.56E-01	4.22E-01
CACNA1C	-0.33942	2.37E-15	1.78E-14
CACNA1D	-0.38964	4.06E-20	4.79E-19
CACNA1E	0.172983	7.95E-05	1.88E-04
CACNA1F	-0.24833	1.12E-08	4.23E-08
CACNA1G	-0.19271	1.06E-05	2.83E-05
CACNA1H	-0.21119	1.33E-06	3.96E-06
CACNA1I	0.025742	5.60E-01	6.21E-01
CACNA1S	-0.05676	1.98E-01	2.53E-01
CACNA2D1	0.124256	4.74E-03	8.55E-03
CACNA2D2	-0.59723	4.27E-51	2.95E-49
CACNA2D3	-0.30001	3.59E-12	1.99E-11
CACNA2D4	-0.17174	8.96E-05	2.10E-04
CACNB1	-0.45496	1.12E-27	2.45E-26
CACNB2	-0.05978	1.76E-01	2.28E-01
CACNB3	-0.21551	7.93E-07	2.44E-06
CACNB4	-0.32354	5.16E-14	3.43E-13
CACNG1	0.072654	9.96E-02	1.37E-01
CACNG2	0.133271	2.44E-03	4.61E-03
CACNG3	0.01654	7.08E-01	7.56E-01
CACNG4	-0.0455	3.03E-01	3.66E-01
CACNG5	0.063414	1.51E-01	1.99E-01
CACNG6	-0.14412	1.04E-03	2.08E-03
CACNG7	0.186143	2.13E-05	5.45E-05
CACNG8	0.072339	1.01E-01	1.39E-01
CACYBP	0.399644	3.60E-21	4.64E-20
CADM1	-0.27955	1.06E-10	5.03E-10
CADM2	0.052052	2.38E-01	2.98E-01
CADM3	-0.26221	1.52E-09	6.37E-09
CADM4	-0.07677	8.18E-02	1.15E-01
CADPS2	-0.30787	9.07E-13	5.35E-12
CADPS	-0.14698	8.21E-04	1.67E-03
CAD	0.409421	3.11E-22	4.36E-21
CAGE1	0.274884	2.22E-10	1.02E-09
CALB1	0.147444	7.90E-04	1.61E-03

CALB2	0.156517	3.63E-04	7.80E-04
CALCA	0.057751	1.91E-01	2.45E-01
CALCB	0.169543	1.11E-04	2.56E-04
CALCOCO1	-0.56277	2.35E-44	1.33E-42
CALCOCO2	-0.29307	1.17E-11	6.10E-11
CALCRL	-0.26609	8.53E-10	3.67E-09
CALCR	0.093488	3.39E-02	5.18E-02
CALD1	0.061832	1.61E-01	2.11E-01
CALHM1	0.035096	4.27E-01	4.93E-01
CALHM2	0.06534	1.39E-01	1.84E-01
CALHM3	0.060976	1.67E-01	2.18E-01
CALM1	-0.11725	7.73E-03	1.34E-02
CALM2	0.1637	1.91E-04	4.27E-04
CALM3	0.282544	6.58E-11	3.18E-10
CALML3	0.116528	8.12E-03	1.40E-02
CALML4	-0.32998	1.51E-14	1.06E-13
CALML5	0.079422	7.17E-02	1.02E-01
CALML6	-0.15569	3.91E-04	8.35E-04
CALN1	0.02257	6.09E-01	6.66E-01
CALR3	0.136736	1.87E-03	3.60E-03
CALR	0.156524	3.63E-04	7.80E-04
CALU	0.518603	8.55E-37	3.26E-35
CALY	0.16784	1.30E-04	2.98E-04
CAMK1D	-0.20757	2.03E-06	5.92E-06
CAMK1G	-0.15861	3.02E-04	6.57E-04
CAMK1	-0.16176	2.28E-04	5.05E-04
CAMK2A	-0.28118	8.19E-11	3.93E-10
CAMK2B	0.090732	3.96E-02	5.96E-02
CAMK2D	-0.35986	3.43E-17	3.13E-16
CAMK2G	-0.19156	1.20E-05	3.19E-05
CAMK2N1	0.057579	1.92E-01	2.46E-01
CAMK2N2	0.387826	6.25E-20	7.24E-19
CAMK4	-0.03378	4.44E-01	5.11E-01
CAMKK1	-0.35439	1.10E-16	9.48E-16
CAMKK2	0.201399	4.09E-06	1.15E-05
CAMKV	0.121884	5.61E-03	9.99E-03
CAMLG	-0.13983	1.47E-03	2.87E-03
CAMP	-0.02408	5.86E-01	6.44E-01
CAMSAP1L1	-0.02604	5.55E-01	6.17E-01
CAMSAP1	0.082632	6.10E-02	8.84E-02
CAMTA1	-0.11398	9.63E-03	1.64E-02
CAMTA2	-0.3756	1.06E-18	1.10E-17
CAND1	0.387805	6.28E-20	7.27E-19
CAND2	-0.02373	5.91E-01	6.49E-01

CANT1	0.290305	1.85E-11	9.49E-11
CANX	-0.02372	5.91E-01	6.49E-01
CAP1	0.105946	1.62E-02	2.64E-02
CAP2	0.013189	7.65E-01	8.05E-01
CAPG	0.005004	9.10E-01	9.28E-01
CAPN10	-0.12007	6.37E-03	1.12E-02
CAPN11	-0.16995	1.06E-04	2.47E-04
CAPN12	-0.22161	3.77E-07	1.21E-06
CAPN13	-0.23498	6.84E-08	2.38E-07
CAPN14	0.052123	2.38E-01	2.97E-01
CAPN1	-0.05272	2.32E-01	2.91E-01
CAPN2	-0.19357	9.68E-06	2.60E-05
CAPN3	-0.51573	2.44E-36	9.02E-35
CAPN5	-0.18519	2.35E-05	5.98E-05
CAPN6	-0.3136	3.24E-13	2.00E-12
CAPN7	-0.12937	3.27E-03	6.05E-03
CAPN8	-0.43071	1.13E-24	1.93E-23
CAPN9	-0.34543	7.04E-16	5.61E-15
CAPNS1	0.002915	9.47E-01	9.59E-01
CAPNS2	-0.11791	7.39E-03	1.28E-02
CAPRIN1	0.17014	1.04E-04	2.43E-04
CAPRIN2	-0.03506	4.27E-01	4.94E-01
CAPS2	-0.10317	1.92E-02	3.08E-02
CAPSL	-0.22455	2.61E-07	8.54E-07
CAPS	-0.35076	2.35E-16	1.96E-15
CAPZA1	0.45542	9.80E-28	2.15E-26
CAPZA2	0.215641	7.81E-07	2.40E-06
CAPZA3	-0.04829	2.74E-01	3.36E-01
CAPZB	0.162636	2.10E-04	4.68E-04
CARD10	-0.05229	2.36E-01	2.95E-01
CARD11	-0.10676	1.54E-02	2.52E-02
CARD14	0.128263	3.55E-03	6.53E-03
CARD16	-0.1391	1.55E-03	3.03E-03
CARD17	0.169218	1.14E-04	2.64E-04
CARD18	0.166952	1.41E-04	3.22E-04
CARD6	-0.15421	4.45E-04	9.40E-04
CARD8	-0.27217	3.38E-10	1.53E-09
CARD9	-0.11439	9.37E-03	1.60E-02
CARHSP1	0.285934	3.80E-11	1.88E-10
CARKD	-0.22144	3.85E-07	1.23E-06
CARM1	0.245644	1.63E-08	6.05E-08
CARNS1	-0.34408	9.27E-16	7.28E-15
CARS2	-0.11451	9.30E-03	1.59E-02
CARS	0.339112	2.52E-15	1.89E-14

CARTPT	0.010866	8.06E-01	8.40E-01
CASC1	-0.3373	3.61E-15	2.67E-14
CASC2	-0.32043	9.23E-14	6.00E-13
CASC3	0.039566	3.70E-01	4.36E-01
CASC4	-0.0911	3.88E-02	5.85E-02
CASC5	0.786726	1.50E-109	2.88E-107
CASD1	-0.22912	1.46E-07	4.92E-07
CASKIN1	0.070804	1.09E-01	1.48E-01
CASKIN2	-0.18227	3.17E-05	7.91E-05
CASK	0.054955	2.13E-01	2.69E-01
CASP10	-0.18608	2.14E-05	5.48E-05
CASP12	-0.38197	2.46E-19	2.72E-18
CASP14	0.158796	2.97E-04	6.47E-04
CASP1	-0.10691	1.52E-02	2.50E-02
CASP2	0.280588	9.01E-11	4.30E-10
CASP3	0.368709	4.98E-18	4.85E-17
CASP4	0.046074	2.97E-01	3.60E-01
CASP5	0.288821	2.37E-11	1.20E-10
CASP6	0.174762	6.69E-05	1.60E-04
CASP7	0.007935	8.57E-01	8.84E-01
CASP8AP2	0.28298	6.14E-11	2.97E-10
CASP8	0.080136	6.92E-02	9.92E-02
CASP9	-0.01364	7.57E-01	7.98E-01
CASQ1	-0.19331	9.96E-06	2.67E-05
CASQ2	-0.42549	4.63E-24	7.61E-23
CASR	-0.30189	2.59E-12	1.46E-11
CASS4	-0.34476	8.08E-16	6.41E-15
CAST	-0.09392	3.31E-02	5.07E-02
CASZ1	-0.44891	6.64E-27	1.35E-25
CATSPER1	0.198205	5.84E-06	1.61E-05
CATSPER2P1	0.075351	8.76E-02	1.22E-01
CATSPER2	-0.21616	7.34E-07	2.26E-06
CATSPER3	-0.14294	1.14E-03	2.27E-03
CATSPER4	0.043742	3.22E-01	3.87E-01
CATSPERB	-0.08129	6.53E-02	9.41E-02
CATSPERG	-0.24712	1.33E-08	4.98E-08
CAT	-0.45368	1.64E-27	3.53E-26
CAV1	-0.16316	2.00E-04	4.47E-04
CAV2	-0.13125	2.84E-03	5.31E-03
CAV3	-0.51617	2.08E-36	7.73E-35
CBARA1	-0.08029	6.87E-02	9.85E-02
CBFA2T2	-0.00416	9.25E-01	9.40E-01
CBFA2T3	-0.34673	5.40E-16	4.35E-15
CBFB	0.142969	1.14E-03	2.27E-03

CBLB	0.108575	1.37E-02	2.27E-02
CBLC	0.0933	3.43E-02	5.23E-02
CBLL1	0.322319	6.49E-14	4.28E-13
CBLN1	-0.06106	1.67E-01	2.17E-01
CBLN2	0.147113	8.12E-04	1.65E-03
CBLN3	-0.04265	3.34E-01	4.00E-01
CBLN4	-0.24665	1.41E-08	5.29E-08
CBL	0.075642	8.64E-02	1.21E-01
CBR1	-0.19069	1.32E-05	3.47E-05
CBR3	-0.01934	6.61E-01	7.14E-01
CBR4	-0.28821	2.62E-11	1.32E-10
CBS	0.276884	1.62E-10	7.56E-10
CBWD1	0.082259	6.21E-02	8.99E-02
CBWD2	-0.01006	8.20E-01	8.52E-01
CBWD3	0.155872	3.85E-04	8.22E-04
CBWD5	0.118928	6.89E-03	1.20E-02
CBWD6	0.114588	9.25E-03	1.58E-02
CBX1	0.405405	8.58E-22	1.16E-20
CBX2	0.29227	1.33E-11	6.94E-11
CBX3	0.440743	6.90E-26	1.31E-24
CBX4	0.021168	6.32E-01	6.87E-01
CBX5	0.170393	1.02E-04	2.38E-04
CBX6	-0.0434	3.26E-01	3.91E-01
CBX7	-0.60062	8.37E-52	5.93E-50
CBX8	0.105146	1.70E-02	2.76E-02
CBY1	-0.16437	1.79E-04	4.03E-04
CC2D1A	-0.10584	1.63E-02	2.66E-02
CC2D1B	-0.16441	1.79E-04	4.01E-04
CC2D2A	-0.32455	4.26E-14	2.86E-13
CC2D2B	-0.27233	3.29E-10	1.49E-09
CCAR1	0.225474	2.33E-07	7.63E-07
CCBE1	-0.02923	5.08E-01	5.72E-01
CCBL1	-0.03925	3.74E-01	4.40E-01
CCBL2	-0.00227	9.59E-01	9.68E-01
CCBP2	0.006797	8.78E-01	9.01E-01
CCDC101	-0.35315	1.43E-16	1.22E-15
CCDC102A	-0.31589	2.13E-13	1.34E-12
CCDC102B	-0.06718	1.28E-01	1.72E-01
CCDC103	-0.01262	7.75E-01	8.13E-01
CCDC104	-0.03099	4.83E-01	5.48E-01
CCDC105	0.055356	2.10E-01	2.66E-01
CCDC106	-0.11927	6.73E-03	1.18E-02
CCDC107	-0.23187	1.03E-07	3.50E-07
CCDC108	-0.30216	2.47E-12	1.40E-11

CCDC109A	-0.01105	8.03E-01	8.37E-01
CCDC109B	0.335193	5.48E-15	3.99E-14
CCDC110	0.143988	1.05E-03	2.10E-03
CCDC111	0.036516	4.08E-01	4.75E-01
CCDC112	-0.07748	7.90E-02	1.12E-01
CCDC113	-0.10623	1.59E-02	2.60E-02
CCDC114	-0.29825	4.85E-12	2.65E-11
CCDC115	-0.36948	4.20E-18	4.13E-17
CCDC116	-0.09886	2.49E-02	3.90E-02
CCDC117	0.032849	4.57E-01	5.23E-01
CCDC11	-0.22598	2.18E-07	7.19E-07
CCDC120	-0.14286	1.15E-03	2.29E-03
CCDC121	-0.15338	4.78E-04	1.01E-03
CCDC122	-0.00472	9.15E-01	9.32E-01
CCDC123	0.266393	8.15E-10	3.52E-09
CCDC124	0.086856	4.88E-02	7.22E-02
CCDC125	-0.22146	3.84E-07	1.23E-06
CCDC126	0.105788	1.63E-02	2.66E-02
CCDC127	0.034059	4.41E-01	5.07E-01
CCDC129	-0.14802	7.53E-04	1.54E-03
CCDC12	-0.19921	5.22E-06	1.45E-05
CCDC130	-0.19041	1.36E-05	3.57E-05
CCDC132	0.231827	1.03E-07	3.52E-07
CCDC134	0.088437	4.49E-02	6.68E-02
CCDC135	-0.2286	1.56E-07	5.23E-07
CCDC136	0.060485	1.71E-01	2.22E-01
CCDC137	0.329735	1.58E-14	1.10E-13
CCDC138	0.503593	1.81E-34	6.04E-33
CCDC13	-0.35104	2.22E-16	1.86E-15
CCDC140	0.131669	2.75E-03	5.16E-03
CCDC141	-0.25665	3.43E-09	1.38E-08
CCDC142	0.156887	3.52E-04	7.57E-04
CCDC144A	-0.13195	2.70E-03	5.06E-03
CCDC144B	-0.16056	2.54E-04	5.58E-04
CCDC144C	0.076266	8.38E-02	1.18E-01
CCDC144NL	0.085081	5.37E-02	7.86E-02
CCDC146	-0.35857	4.52E-17	4.08E-16
CCDC147	-0.35198	1.82E-16	1.53E-15
CCDC148	-0.03821	3.87E-01	4.53E-01
CCDC149	-0.26106	1.80E-09	7.49E-09
CCDC14	-0.00681	8.77E-01	9.01E-01
CCDC150	0.518575	8.64E-37	3.29E-35
CCDC151	-0.16094	2.45E-04	5.40E-04
CCDC152	-0.3636	1.53E-17	1.43E-16

CCDC153	-0.14874	7.09E-04	1.46E-03
CCDC154	-0.08233	6.19E-02	8.97E-02
CCDC155	-0.00468	9.16E-01	9.33E-01
CCDC157	-0.30408	1.77E-12	1.01E-11
CCDC158	-0.00314	9.43E-01	9.56E-01
CCDC159	-0.4495	5.59E-27	1.14E-25
CCDC15	0.232992	8.88E-08	3.05E-07
CCDC160	0.048575	2.71E-01	3.33E-01
CCDC163P	0.162896	2.05E-04	4.58E-04
CCDC17	-0.25336	5.50E-09	2.17E-08
CCDC18	0.463113	9.70E-29	2.28E-27
CCDC19	-0.21677	6.82E-07	2.11E-06
CCDC21	0.311857	4.44E-13	2.71E-12
CCDC22	-0.00472	9.15E-01	9.32E-01
CCDC23	0.050428	2.53E-01	3.14E-01
CCDC24	-0.23312	8.72E-08	3.00E-07
CCDC25	-0.07629	8.37E-02	1.18E-01
CCDC27	0.019343	6.61E-01	7.14E-01
CCDC28A	-0.37291	1.95E-18	1.98E-17
CCDC28B	0.184909	2.42E-05	6.14E-05
CCDC30	-0.4114	1.87E-22	2.67E-21
CCDC33	-0.2257	2.26E-07	7.43E-07
CCDC34	0.523659	1.32E-37	5.23E-36
CCDC36	-0.12987	3.15E-03	5.85E-03
CCDC37	-0.27016	4.59E-10	2.05E-09
CCDC38	0.077959	7.71E-02	1.09E-01
CCDC39	-0.28968	2.06E-11	1.05E-10
CCDC3	-0.20305	3.40E-06	9.68E-06
CCDC40	-0.22252	3.37E-07	1.08E-06
CCDC41	-0.00732	8.68E-01	8.93E-01
CCDC42B	-0.29932	4.04E-12	2.23E-11
CCDC42	-0.14313	1.13E-03	2.24E-03
CCDC43	0.456359	7.42E-28	1.63E-26
CCDC45	-0.01798	6.84E-01	7.34E-01
CCDC46	-0.42193	1.20E-23	1.91E-22
CCDC47	-0.02061	6.41E-01	6.95E-01
CCDC48	-0.54991	4.90E-42	2.49E-40
CCDC50	0.03769	3.93E-01	4.60E-01
CCDC51	0.204593	2.85E-06	8.18E-06
CCDC52	0.235975	6.00E-08	2.09E-07
CCDC53	-0.0104	8.14E-01	8.46E-01
CCDC54	-0.04696	2.87E-01	3.50E-01
CCDC55	0.009371	8.32E-01	8.62E-01
CCDC56	-0.07461	9.07E-02	1.26E-01

CCDC57	-0.34867	3.63E-16	2.97E-15
CCDC58	0.559587	9.00E-44	4.95E-42
CCDC59	0.47765	1.04E-30	2.81E-29
CCDC60	-0.29854	4.62E-12	2.53E-11
CCDC61	-0.30853	8.06E-13	4.78E-12
CCDC62	0.075969	8.50E-02	1.19E-01
CCDC63	0.088389	4.50E-02	6.70E-02
CCDC64B	-0.45751	5.26E-28	1.18E-26
CCDC64	0.049357	2.64E-01	3.25E-01
CCDC65	-0.269	5.49E-10	2.42E-09
CCDC66	-0.31619	2.02E-13	1.27E-12
CCDC67	0.067602	1.25E-01	1.69E-01
CCDC68	-0.23077	1.18E-07	4.01E-07
CCDC69	-0.37998	3.90E-19	4.20E-18
CCDC6	-0.08026	6.88E-02	9.86E-02
CCDC70	0.082837	6.03E-02	8.75E-02
CCDC71	-0.1617	2.29E-04	5.07E-04
CCDC72	0.013138	7.66E-01	8.06E-01
CCDC73	-0.22329	3.06E-07	9.89E-07
CCDC74A	0.069096	1.17E-01	1.59E-01
CCDC74B	-0.00332	9.40E-01	9.53E-01
CCDC75	0.302659	2.27E-12	1.29E-11
CCDC76	0.037601	3.94E-01	4.61E-01
CCDC77	0.381844	2.54E-19	2.79E-18
CCDC78	-0.2433	2.24E-08	8.21E-08
CCDC79	-0.07757	7.86E-02	1.11E-01
CCDC7	0.063848	1.48E-01	1.95E-01
CCDC80	0.033651	4.46E-01	5.12E-01
CCDC81	-0.29046	1.80E-11	9.26E-11
CCDC82	-0.079	7.33E-02	1.04E-01
CCDC83	0.074434	9.15E-02	1.27E-01
CCDC84	-0.22905	1.48E-07	4.96E-07
CCDC85A	-0.27816	1.32E-10	6.23E-10
CCDC85B	0.088497	4.47E-02	6.66E-02
CCDC85C	0.194407	8.85E-06	2.39E-05
CCDC86	0.441391	5.74E-26	1.10E-24
CCDC87	0.052602	2.33E-01	2.92E-01
CCDC88A	0.29535	7.95E-12	4.23E-11
CCDC88B	-0.2117	1.25E-06	3.74E-06
CCDC88C	-0.17345	7.60E-05	1.80E-04
CCDC89	-0.18244	3.11E-05	7.77E-05
CCDC8	-0.231	1.15E-07	3.90E-07
CCDC90A	-0.04062	3.58E-01	4.24E-01
CCDC90B	0.42957	1.53E-24	2.61E-23

CCDC91	0.171251	9.39E-05	2.20E-04
CCDC92	-0.40792	4.55E-22	6.30E-21
CCDC93	-0.08575	5.18E-02	7.61E-02
CCDC94	-0.09588	2.96E-02	4.57E-02
CCDC96	-0.26837	6.04E-10	2.65E-09
CCDC97	-0.10377	1.85E-02	2.98E-02
CCDC99	0.646838	2.30E-62	2.11E-60
CCDC9	-0.0422	3.39E-01	4.05E-01
CCHCR1	0.132677	2.55E-03	4.80E-03
CCIN	0.117466	7.62E-03	1.32E-02
CCKAR	-0.09647	2.86E-02	4.43E-02
CCKBR	-0.0375	3.96E-01	4.62E-01
CCK	-0.04186	3.43E-01	4.09E-01
CCL11	0.282848	6.27E-11	3.04E-10
CCL13	-0.12663	4.00E-03	7.29E-03
CCL14-CCL15	-0.11589	8.48E-03	1.46E-02
CCL14	-0.41757	3.79E-23	5.75E-22
CCL15	-0.05716	1.95E-01	2.50E-01
CCL16	-0.34734	4.77E-16	3.86E-15
CCL17	-0.29425	9.57E-12	5.05E-11
CCL18	0.044758	3.11E-01	3.75E-01
CCL19	-0.23024	1.27E-07	4.29E-07
CCL1	0.132184	2.65E-03	4.97E-03
CCL20	0.171205	9.43E-05	2.21E-04
CCL21	-0.02449	5.79E-01	6.39E-01
CCL22	-0.19003	1.41E-05	3.70E-05
CCL23	-0.16487	1.71E-04	3.86E-04
CCL24	0.12084	6.04E-03	1.07E-02
CCL25	0.111485	1.13E-02	1.91E-02
CCL26	0.42221	1.11E-23	1.77E-22
CCL27	0.003042	9.45E-01	9.57E-01
CCL28	0.004581	9.17E-01	9.34E-01
CCL2	0.078575	7.48E-02	1.06E-01
CCL3L1	0.207915	1.95E-06	5.69E-06
CCL3L3	0.023461	5.95E-01	6.53E-01
CCL3	0.210915	1.37E-06	4.08E-06
CCL4L2	0.270702	4.23E-10	1.89E-09
CCL4	0.265237	9.69E-10	4.15E-09
CCL5	0.110718	1.19E-02	2.00E-02
CCL7	0.385627	1.05E-19	1.19E-18
CCL8	0.322106	6.75E-14	4.45E-13
CCM2	0.190636	1.33E-05	3.49E-05
CCNA1	0.028132	5.24E-01	5.88E-01
CCNA2	0.897129	3.79E-184	1.89E-180

CCNB1IP1	0.132735	2.54E-03	4.78E-03
CCNB1	0.86136	5.21E-153	3.26E-150
CCNB2	0.896301	2.65E-183	1.06E-179
CCNB3	0.078495	7.51E-02	1.07E-01
CCNC	0.277742	1.41E-10	6.64E-10
CCND1	-0.09541	3.04E-02	4.69E-02
CCND2	-0.26286	1.38E-09	5.81E-09
CCND3	-0.3648	1.18E-17	1.12E-16
CCNDBP1	-0.31609	2.06E-13	1.29E-12
CCNE1	0.698817	1.13E-76	1.32E-74
CCNE2	0.707477	2.32E-79	2.84E-77
CCNF	0.639453	1.48E-60	1.30E-58
CCNG1	-0.1503	6.21E-04	1.29E-03
CCNG2	0.146907	8.26E-04	1.68E-03
CCNH	-0.05694	1.97E-01	2.52E-01
CCNI2	0.109325	1.31E-02	2.17E-02
CCNI	0.082218	6.23E-02	9.01E-02
CCNJL	-0.21001	1.52E-06	4.51E-06
CCNJ	0.216043	7.44E-07	2.29E-06
CCNK	0.300777	3.14E-12	1.76E-11
CCNL1	-0.07905	7.31E-02	1.04E-01
CCNL2	-0.24822	1.14E-08	4.30E-08
CCNO	-0.00204	9.63E-01	9.71E-01
CCNT1	0.186601	2.03E-05	5.21E-05
CCNT2	-0.11846	7.12E-03	1.24E-02
CCNYL1	0.227888	1.71E-07	5.70E-07
CCNY	0.096081	2.92E-02	4.52E-02
CCPG1	-0.31228	4.12E-13	2.51E-12
CCR10	0.119018	6.85E-03	1.20E-02
CCR1	0.048155	2.75E-01	3.37E-01
CCR2	-0.18333	2.84E-05	7.14E-05
CCR3	-0.10207	2.05E-02	3.28E-02
CCR4	-0.16732	1.36E-04	3.11E-04
CCR5	-0.00117	9.79E-01	9.84E-01
CCR6	-0.34201	1.41E-15	1.08E-14
CCR7	-0.21654	7.01E-07	2.17E-06
CCR8	0.054878	2.14E-01	2.70E-01
CCR9	-0.14964	6.57E-04	1.36E-03
CCRL1	-0.07164	1.04E-01	1.43E-01
CCRL2	-0.11958	6.59E-03	1.16E-02
CCRN4L	0.509832	2.02E-35	7.12E-34
CCS	-0.12526	4.42E-03	8.00E-03
CCT2	0.425859	4.19E-24	6.93E-23
CCT3	0.41823	3.18E-23	4.87E-22

CCT4	0.561913	3.38E-44	1.90E-42
CCT5	0.565479	7.40E-45	4.30E-43
CCT6A	0.546012	2.36E-41	1.15E-39
CCT6B	-0.27471	2.28E-10	1.05E-09
CCT6P1	-0.13988	1.46E-03	2.86E-03
CCT7	0.572849	3.03E-46	1.85E-44
CCT8L2	-0.21089	1.38E-06	4.09E-06
CCT8	0.428371	2.13E-24	3.59E-23
CD101	-0.10491	1.72E-02	2.80E-02
CD109	0.390877	3.02E-20	3.61E-19
CD14	0.050927	2.49E-01	3.09E-01
CD151	-0.32439	4.40E-14	2.95E-13
CD160	-0.11202	1.10E-02	1.85E-02
CD163L1	0.187787	1.79E-05	4.64E-05
CD163	0.066424	1.32E-01	1.77E-01
CD164L2	-0.3905	3.31E-20	3.94E-19
CD164	0.013082	7.67E-01	8.06E-01
CD177	0.052159	2.37E-01	2.97E-01
CD180	-0.06814	1.22E-01	1.65E-01
CD19	-0.09933	2.42E-02	3.81E-02
CD1A	-0.3579	5.22E-17	4.67E-16
CD1B	-0.31901	1.20E-13	7.71E-13
CD1C	-0.43793	1.52E-25	2.81E-24
CD1D	-0.12409	4.80E-03	8.65E-03
CD1E	-0.46409	7.19E-29	1.71E-27
CD200R1L	-0.09623	2.90E-02	4.49E-02
CD200R1	-0.19164	1.19E-05	3.16E-05
CD200	0.009572	8.28E-01	8.59E-01
CD207	-0.44368	2.99E-26	5.86E-25
CD209	0.060813	1.68E-01	2.19E-01
CD226	0.023857	5.89E-01	6.47E-01
CD22	-0.29889	4.35E-12	2.39E-11
CD244	0.016007	7.17E-01	7.64E-01
CD247	-0.04307	3.29E-01	3.95E-01
CD248	0.017563	6.91E-01	7.40E-01
CD24	0.017079	6.99E-01	7.48E-01
CD274	0.28702	3.18E-11	1.59E-10
CD276	0.305863	1.29E-12	7.51E-12
CD27	-0.14209	1.22E-03	2.43E-03
CD28	-0.12886	3.40E-03	6.27E-03
CD2AP	0.188014	1.75E-05	4.54E-05
CD2BP2	-0.0184	6.77E-01	7.28E-01
CD2	-0.05394	2.22E-01	2.79E-01
CD300A	0.129279	3.29E-03	6.09E-03

CD300C	-0.07135	1.06E-01	1.45E-01
CD300E	0.117958	7.37E-03	1.28E-02
CD300LB	-0.14915	6.85E-04	1.41E-03
CD300LD	0.019793	6.54E-01	7.07E-01
CD300LF	-0.16448	1.77E-04	3.99E-04
CD300LG	-0.50396	1.60E-34	5.34E-33
CD302	-0.62088	3.24E-56	2.56E-54
CD320	0.01823	6.80E-01	7.30E-01
CD33	-0.17017	1.04E-04	2.43E-04
CD34	-0.37435	1.41E-18	1.45E-17
CD36	-0.21364	9.93E-07	3.01E-06
CD37	-0.2613	1.74E-09	7.24E-09
CD38	-0.03687	4.04E-01	4.70E-01
CD3D	0.018202	6.80E-01	7.31E-01
CD3EAP	0.215992	7.49E-07	2.31E-06
CD3E	-0.06556	1.37E-01	1.83E-01
CD3G	0.019502	6.59E-01	7.11E-01
CD40LG	-0.35107	2.20E-16	1.84E-15
CD40	-0.01057	8.11E-01	8.44E-01
CD44	-0.13877	1.59E-03	3.10E-03
CD46	-0.14785	7.63E-04	1.56E-03
CD47	-0.23153	1.07E-07	3.66E-07
CD48	-0.09713	2.75E-02	4.28E-02
CD4	-0.18404	2.64E-05	6.67E-05
CD52	-0.20671	2.24E-06	6.49E-06
CD53	-0.0172	6.97E-01	7.46E-01
CD55	-0.30924	7.11E-13	4.24E-12
CD58	0.015811	7.20E-01	7.67E-01
CD59	-0.32783	2.29E-14	1.57E-13
CD5L	-0.14866	7.14E-04	1.47E-03
CD5	-0.17081	9.80E-05	2.29E-04
CD63	-0.23296	8.91E-08	3.06E-07
CD68	0.040866	3.55E-01	4.21E-01
CD69	-0.13103	2.89E-03	5.39E-03
CD6	-0.09283	3.52E-02	5.36E-02
CD70	0.18508	2.37E-05	6.04E-05
CD72	0.141928	1.24E-03	2.46E-03
CD74	-0.33879	2.69E-15	2.01E-14
CD79A	-0.11903	6.85E-03	1.20E-02
CD79B	-0.15396	4.54E-04	9.60E-04
CD7	0.145105	9.58E-04	1.93E-03
CD80	0.005516	9.01E-01	9.20E-01
CD81	-0.59391	2.06E-50	1.41E-48
CD82	-0.2659	8.77E-10	3.77E-09

CD83	-0.23185	1.03E-07	3.51E-07
CD84	-0.02359	5.93E-01	6.51E-01
CD86	0.063716	1.49E-01	1.96E-01
CD8A	0.131185	2.86E-03	5.34E-03
CD8B	0.167534	1.34E-04	3.06E-04
CD93	-0.1944	8.86E-06	2.39E-05
CD96	-0.08685	4.89E-02	7.22E-02
CD97	-0.26566	9.09E-10	3.91E-09
CD99L2	-0.1377	1.74E-03	3.36E-03
CD99	0.190288	1.38E-05	3.61E-05
CD9	-0.1414	1.29E-03	2.56E-03
CDADC1	-0.24645	1.45E-08	5.43E-08
CDAN1	-0.13196	2.70E-03	5.05E-03
CDA	0.195948	7.48E-06	2.04E-05
CDC123	0.327425	2.47E-14	1.69E-13
CDC14A	-0.0753	8.78E-02	1.23E-01
CDC14B	-0.21608	7.41E-07	2.28E-06
CDC14C	-0.06566	1.37E-01	1.82E-01
CDC16	-0.08901	4.35E-02	6.49E-02
CDC20B	-0.12759	3.73E-03	6.83E-03
CDC20	0.852952	5.97E-147	2.84E-144
CDC23	0.172596	8.25E-05	1.95E-04
CDC25A	0.806548	3.37E-119	7.74E-117
CDC25B	0.215478	7.97E-07	2.44E-06
CDC25C	0.841158	4.62E-139	1.74E-136
CDC26	0.090213	4.07E-02	6.12E-02
CDC27	0.521197	3.30E-37	1.28E-35
CDC34	0.33043	1.39E-14	9.70E-14
CDC37L1	-0.13294	2.50E-03	4.71E-03
CDC37	0.088841	4.39E-02	6.55E-02
CDC40	0.044574	3.13E-01	3.77E-01
CDC42BPA	-0.29658	6.45E-12	3.47E-11
CDC42BPB	-0.0012	9.78E-01	9.84E-01
CDC42BPG	-0.19764	6.22E-06	1.71E-05
CDC42EP1	-0.18456	2.50E-05	6.35E-05
CDC42EP2	0.174166	7.09E-05	1.69E-04
CDC42EP3	-0.10274	1.97E-02	3.16E-02
CDC42EP4	-0.19629	7.21E-06	1.97E-05
CDC42EP5	-0.03727	3.99E-01	4.65E-01
CDC42SE1	0.073609	9.52E-02	1.32E-01
CDC42SE2	-0.14657	8.50E-04	1.73E-03
CDC42	0.033892	4.43E-01	5.09E-01
CDC45	0.844645	2.52E-141	1.05E-138
CDC5L	0.31035	5.83E-13	3.51E-12

CDC6	0.879085	3.36E-167	3.95E-164
CDC73	0.213656	9.91E-07	3.00E-06
CDC7	0.711955	8.62E-81	1.06E-78
CDCA2	0.838496	2.27E-137	7.70E-135
CDCA3	0.815764	4.48E-124	1.12E-121
CDCA4	0.685862	7.96E-73	8.74E-71
CDCA5	0.873586	1.43E-162	1.43E-159
CDCA7L	0.039268	3.74E-01	4.40E-01
CDCA7	0.409803	2.82E-22	3.97E-21
CDCA8	0.869786	1.70E-159	1.48E-156
CDCP1	0.259221	2.36E-09	9.67E-09
CDCP2	-0.08132	6.52E-02	9.40E-02
CDH10	-0.03139	4.77E-01	5.42E-01
CDH11	-0.08487	5.43E-02	7.94E-02
CDH12	0.101691	2.10E-02	3.34E-02
CDH13	-0.03577	4.18E-01	4.84E-01
CDH15	-0.36913	4.54E-18	4.44E-17
CDH16	-0.18674	2.00E-05	5.14E-05
CDH17	0.083134	5.94E-02	8.63E-02
CDH18	0.346033	6.23E-16	4.99E-15
CDH19	-0.17015	1.04E-04	2.43E-04
CDH1	-0.05999	1.74E-01	2.26E-01
CDH20	-0.32814	2.15E-14	1.48E-13
CDH22	-0.09892	2.48E-02	3.89E-02
CDH23	-0.32178	7.17E-14	4.72E-13
CDH24	0.427945	2.39E-24	4.01E-23
CDH26	-0.26489	1.02E-09	4.36E-09
CDH2	0.220512	4.32E-07	1.37E-06
CDH3	0.205737	2.50E-06	7.21E-06
CDH4	-0.19548	7.87E-06	2.14E-05
CDH5	-0.29124	1.58E-11	8.16E-11
CDH6	-0.13616	1.96E-03	3.75E-03
CDH7	0.316515	1.90E-13	1.20E-12
CDH8	-0.06334	1.51E-01	1.99E-01
CDH9	0.02169	6.23E-01	6.79E-01
CDHR1	-0.16691	1.42E-04	3.23E-04
CDHR2	0.082663	6.09E-02	8.82E-02
CDHR3	-0.36972	3.98E-18	3.92E-17
CDHR4	-0.25315	5.68E-09	2.23E-08
CDHR5	-0.03675	4.05E-01	4.72E-01
CDIPT	-0.32028	9.50E-14	6.16E-13
CDK10	-0.35271	1.57E-16	1.33E-15
CDK11A	-0.14251	1.18E-03	2.35E-03
CDK11B	-0.06623	1.33E-01	1.78E-01

CDK12	0.285681	3.96E-11	1.95E-10
CDK13	0.000953	9.83E-01	9.87E-01
CDK14	-0.08081	6.69E-02	9.62E-02
CDK15	-0.20628	2.35E-06	6.80E-06
CDK16	0.352417	1.66E-16	1.41E-15
CDK17	0.18907	1.57E-05	4.09E-05
CDK18	-0.37518	1.17E-18	1.21E-17
CDK19	-0.0844	5.56E-02	8.12E-02
CDK1	0.862616	5.99E-154	3.86E-151
CDK20	-0.34995	2.78E-16	2.30E-15
CDK2AP1	0.214002	9.51E-07	2.88E-06
CDK2AP2	-0.2164	7.13E-07	2.20E-06
CDK2	0.694848	1.80E-75	2.06E-73
CDK3	-0.18006	3.96E-05	9.75E-05
CDK4	0.420388	1.80E-23	2.82E-22
CDK5R1	0.430823	1.09E-24	1.88E-23
CDK5R2	0.302639	2.28E-12	1.29E-11
CDK5RAP1	0.159449	2.80E-04	6.12E-04
CDK5RAP2	0.050315	2.54E-01	3.15E-01
CDK5RAP3	-0.1689	1.17E-04	2.71E-04
CDK5	0.196138	7.33E-06	2.00E-05
CDK6	0.226105	2.15E-07	7.08E-07
CDK7	0.074168	9.27E-02	1.29E-01
CDK8	0.419315	2.39E-23	3.70E-22
CDK9	-0.23763	4.81E-08	1.70E-07
CDKAL1	0.233599	8.20E-08	2.82E-07
CDKL1	-0.30308	2.11E-12	1.20E-11
CDKL2	-0.47977	5.26E-31	1.44E-29
CDKL3	-0.12932	3.28E-03	6.07E-03
CDKL4	0.04841	2.73E-01	3.35E-01
CDKL5	-0.20774	1.99E-06	5.81E-06
CDKN1A	0.032429	4.63E-01	5.29E-01
CDKN1B	0.041955	3.42E-01	4.08E-01
CDKN1C	-0.23742	4.95E-08	1.74E-07
CDKN2AIPNL	0.227575	1.78E-07	5.92E-07
CDKN2AIP	-0.1041	1.81E-02	2.93E-02
CDKN2A	0.166198	1.51E-04	3.43E-04
CDKN2BAS	0.191196	1.25E-05	3.30E-05
CDKN2B	-0.12755	3.74E-03	6.85E-03
CDKN2C	0.341305	1.62E-15	1.24E-14
CDKN2D	0.335759	4.90E-15	3.59E-14
CDKN3	0.836389	4.72E-136	1.52E-133
CDNF	-0.29492	8.55E-12	4.54E-11
CDO1	-0.37653	8.60E-19	8.98E-18

CDON	-0.24407	2.02E-08	7.42E-08
CDR1	-0.10355	1.87E-02	3.02E-02
CDR2L	0.107569	1.46E-02	2.41E-02
CDR2	0.170565	1.00E-04	2.34E-04
CDRT15P	-0.22449	2.63E-07	8.59E-07
CDRT15	-0.00208	9.62E-01	9.71E-01
CDRT1	0.031723	4.73E-01	5.38E-01
CDRT4	-0.34436	8.75E-16	6.90E-15
CDS1	-0.3051	1.48E-12	8.55E-12
CDS2	-0.11289	1.04E-02	1.75E-02
CDSN	-0.16699	1.41E-04	3.20E-04
CDT1	0.776159	8.09E-105	1.39E-102
CDV3	0.256831	3.34E-09	1.35E-08
CDX1	0.16248	2.13E-04	4.74E-04
CDX2	0.176074	5.88E-05	1.42E-04
CDY2B	-0.06555	1.37E-01	1.83E-01
CDYL2	0.044077	3.18E-01	3.83E-01
CDYL	0.155627	3.93E-04	8.39E-04
CEACAM16	0.012115	7.84E-01	8.21E-01
CEACAM18	-0.01354	7.59E-01	8.00E-01
CEACAM19	0.137395	1.78E-03	3.43E-03
CEACAM1	-0.04773	2.80E-01	3.41E-01
CEACAM20	0.018893	6.69E-01	7.20E-01
CEACAM21	-0.19656	7.00E-06	1.91E-05
CEACAM22P	0.063333	1.51E-01	1.99E-01
CEACAM3	-0.01708	6.99E-01	7.48E-01
CEACAM4	-0.20037	4.59E-06	1.29E-05
CEACAM5	-0.03599	4.15E-01	4.81E-01
CEACAM6	-0.27449	2.36E-10	1.08E-09
CEACAM7	-0.07409	9.30E-02	1.29E-01
CEACAM8	-0.37819	5.89E-19	6.22E-18
CEBPA	-0.36964	4.06E-18	3.99E-17
CEBPB	0.084203	5.62E-02	8.20E-02
CEBPD	-0.29893	4.32E-12	2.37E-11
CEBPE	-0.01613	7.15E-01	7.62E-01
CEBPG	0.380488	3.47E-19	3.76E-18
CEBPZ	0.406562	6.41E-22	8.77E-21
CECR1	-0.19538	7.96E-06	2.16E-05
CECR2	-0.23205	1.00E-07	3.43E-07
CECR4	-0.15549	3.98E-04	8.48E-04
CECR5	0.171382	9.28E-05	2.17E-04
CECR6	0.07691	8.12E-02	1.14E-01
CECR7	0.098262	2.58E-02	4.03E-02
CELA1	0.029497	5.04E-01	5.69E-01

CELA2A	0.042398	3.37E-01	4.02E-01
CELA2B	-0.0861	5.09E-02	7.49E-02
CELA3A	0.02066	6.40E-01	6.94E-01
CELA3B	-0.03203	4.68E-01	5.34E-01
CELF1	-0.01992	6.52E-01	7.06E-01
CELF2	-0.49858	1.02E-33	3.28E-32
CELF3	-0.06947	1.15E-01	1.56E-01
CELF4	-0.00299	9.46E-01	9.58E-01
CELF5	-0.12881	3.41E-03	6.29E-03
CELF6	-0.31859	1.30E-13	8.32E-13
CELP	0.02845	5.19E-01	5.83E-01
CELSR1	-0.31926	1.15E-13	7.39E-13
CELSR2	-0.14236	1.20E-03	2.38E-03
CELSR3	0.258799	2.51E-09	1.03E-08
CEL	-0.02059	6.41E-01	6.95E-01
CEMP1	-0.22998	1.31E-07	4.43E-07
CEND1	0.173333	7.69E-05	1.82E-04
CENPA	0.895374	2.29E-182	7.64E-179
CENPBD1	0.018279	6.79E-01	7.29E-01
CENPB	-0.0054	9.03E-01	9.21E-01
CENPC1	-0.20696	2.17E-06	6.31E-06
CENPE	0.819531	3.80E-126	9.86E-124
CENPF	0.74216	3.36E-91	4.76E-89
CENPH	0.727137	7.56E-86	9.87E-84
CENPI	0.83889	1.28E-137	4.50E-135
CENPJ	0.279036	1.15E-10	5.45E-10
CENPK	0.797196	1.63E-114	3.44E-112
CENPL	0.696119	7.45E-76	8.61E-74
CENPM	0.725645	2.46E-85	3.15E-83
CENPN	0.753857	1.24E-95	1.85E-93
CENPO	0.760635	2.56E-98	4.03E-96
CENPP	0.506061	7.65E-35	2.59E-33
CENPQ	0.488832	2.73E-32	8.11E-31
CENPT	-0.05743	1.93E-01	2.47E-01
CENPV	0.006679	8.80E-01	9.03E-01
CENPW	0.793435	1.07E-112	2.18E-110
CEP110	-0.0674	1.27E-01	1.70E-01
CEP120	-0.18047	3.80E-05	9.39E-05
CEP135	0.326899	2.73E-14	1.86E-13
CEP152	0.415155	7.11E-23	1.05E-21
CEP164	-0.02813	5.24E-01	5.88E-01
CEP170L	0.042634	3.34E-01	4.00E-01
CEP170	0.106585	1.55E-02	2.54E-02
CEP192	0.081273	6.53E-02	9.42E-02

CEP250	0.131959	2.70E-03	5.05E-03
CEP290	0.00768	8.62E-01	8.87E-01
CEP350	-0.04622	2.95E-01	3.58E-01
CEP55	0.859243	1.90E-151	1.12E-148
CEP57	0.080552	6.78E-02	9.74E-02
CEP63	0.020087	6.49E-01	7.03E-01
CEP68	-0.35652	7.01E-17	6.17E-16
CEP70	-0.16222	2.18E-04	4.85E-04
CEP72	0.30453	1.63E-12	9.41E-12
CEP76	0.391156	2.83E-20	3.40E-19
CEP78	0.546557	1.89E-41	9.28E-40
CEP97	0.286332	3.56E-11	1.77E-10
CEPT1	-0.04105	3.52E-01	4.18E-01
CER1	0.162002	2.23E-04	4.94E-04
CERCAM	0.169184	1.14E-04	2.64E-04
CERKL	-0.30531	1.43E-12	8.25E-12
CERK	-0.19436	8.90E-06	2.40E-05
CES1	-0.25453	4.66E-09	1.85E-08
CES2	-0.44684	1.21E-26	2.42E-25
CES3	-0.17828	4.73E-05	1.15E-04
CES4	-0.20306	3.40E-06	9.67E-06
CES7	-0.04284	3.32E-01	3.97E-01
CES8	-0.33957	2.30E-15	1.73E-14
CETN1	0.048252	2.74E-01	3.36E-01
CETN2	-0.05779	1.90E-01	2.44E-01
CETN3	-0.07695	8.10E-02	1.14E-01
CETN4P	0.123281	5.09E-03	9.12E-03
CETP	-0.09547	3.03E-02	4.67E-02
CFB	-0.03287	4.57E-01	5.23E-01
CFC1B	-0.1161	8.36E-03	1.44E-02
CFDP1	-0.05418	2.20E-01	2.77E-01
CFD	-0.3297	1.60E-14	1.11E-13
CFHR1	0.049761	2.60E-01	3.21E-01
CFHR2	0.051358	2.45E-01	3.05E-01
CFHR3	-0.015	7.34E-01	7.78E-01
CFHR4	0.102935	1.95E-02	3.13E-02
CFHR5	0.159735	2.73E-04	5.98E-04
CFH	-0.09491	3.13E-02	4.81E-02
CFI	-0.37869	5.25E-19	5.59E-18
CFL1	0.401097	2.51E-21	3.29E-20
CFL2	0.205414	2.60E-06	7.47E-06
CFLAR	0.019064	6.66E-01	7.18E-01
CFLP1	-0.13225	2.64E-03	4.95E-03
CFP	-0.26845	5.97E-10	2.62E-09

CFTR	-0.34045	1.93E-15	1.46E-14
CG030	-0.33903	2.56E-15	1.92E-14
CGA	0.133716	2.36E-03	4.46E-03
CGB1	0.088987	4.35E-02	6.50E-02
CGB2	0.114919	9.05E-03	1.55E-02
CGB5	-0.03671	4.06E-01	4.72E-01
CGB7	-0.15307	4.90E-04	1.03E-03
CGB8	0.081552	6.44E-02	9.30E-02
CGB	0.043781	3.21E-01	3.86E-01
CGGBP1	-0.04451	3.13E-01	3.78E-01
CGNL1	-0.52209	2.37E-37	9.26E-36
CGN	-0.18615	2.13E-05	5.44E-05
CGREF1	0.232659	9.27E-08	3.18E-07
CGRRF1	-0.0152	7.31E-01	7.75E-01
CH25H	-0.29088	1.68E-11	8.65E-11
CHAC1	0.244173	1.99E-08	7.33E-08
CHAC2	0.526739	4.19E-38	1.72E-36
CHADL	-0.48659	5.73E-32	1.66E-30
CHAD	-0.40448	1.08E-21	1.45E-20
CHAF1A	0.642419	2.82E-61	2.53E-59
CHAF1B	0.680459	2.79E-71	3.00E-69
CHAT	0.032303	4.64E-01	5.30E-01
CHCHD10	0.056994	1.97E-01	2.51E-01
CHCHD1	0.094615	3.18E-02	4.88E-02
CHCHD2	0.367246	6.88E-18	6.62E-17
CHCHD3	0.557082	2.56E-43	1.39E-41
CHCHD4	0.093436	3.40E-02	5.19E-02
CHCHD5	-0.0103	8.16E-01	8.48E-01
CHCHD6	0.051893	2.40E-01	2.99E-01
CHCHD7	-0.0391	3.76E-01	4.42E-01
CHCHD8	0.280764	8.76E-11	4.18E-10
CHD1L	0.118247	7.22E-03	1.26E-02
CHD1	0.096714	2.82E-02	4.37E-02
CHD2	-0.31024	5.94E-13	3.57E-12
CHD3	-0.2865	3.47E-11	1.72E-10
CHD4	0.051213	2.46E-01	3.06E-01
CHD5	-0.03553	4.21E-01	4.87E-01
CHD6	-0.16322	1.99E-04	4.45E-04
CHD7	0.179806	4.06E-05	9.99E-05
CHD8	0.058186	1.87E-01	2.41E-01
CHD9	-0.32129	7.87E-14	5.15E-13
CHDH	-0.21949	4.89E-07	1.54E-06
CHEK1	0.792617	2.62E-112	5.30E-110
CHEK2	0.534207	2.44E-39	1.09E-37

CHERP	0.118552	7.07E-03	1.23E-02
CHFR	0.370324	3.48E-18	3.45E-17
CHGA	0.050875	2.49E-01	3.09E-01
CHGB	0.120795	6.06E-03	1.07E-02
CHI3L1	-0.07796	7.71E-02	1.09E-01
CHI3L2	-0.33423	6.61E-15	4.78E-14
CHIA	-0.53129	7.47E-39	3.23E-37
CHIC1	-0.17226	8.53E-05	2.01E-04
CHIC2	0.123192	5.12E-03	9.17E-03
CHID1	-0.17647	5.65E-05	1.37E-04
CHIT1	-0.28737	3.01E-11	1.51E-10
CHKA	-0.28009	9.75E-11	4.64E-10
CHKB-CPT1B	-0.24584	1.58E-08	5.89E-08
CHKB	-0.23151	1.08E-07	3.67E-07
CHL1	-0.18098	3.61E-05	8.94E-05
CHML	0.130659	2.97E-03	5.53E-03
CHMP1A	0.059517	1.77E-01	2.30E-01
CHMP1B	-0.01641	7.10E-01	7.58E-01
CHMP2A	-0.03824	3.87E-01	4.53E-01
CHMP2B	0.209671	1.59E-06	4.69E-06
CHMP4A	0.091767	3.74E-02	5.65E-02
CHMP4B	0.116039	8.39E-03	1.44E-02
CHMP4C	0.119799	6.49E-03	1.14E-02
CHMP5	0.181393	3.46E-05	8.60E-05
CHMP6	-0.17962	4.14E-05	1.02E-04
CHMP7	-0.11057	1.20E-02	2.02E-02
CHM	0.140302	1.41E-03	2.77E-03
CHN1	0.174303	7.00E-05	1.67E-04
CHN2	-0.10368	1.86E-02	3.00E-02
CHODL	0.054465	2.17E-01	2.74E-01
CHORDC1	0.422636	9.94E-24	1.60E-22
CHP2	-0.09213	3.66E-02	5.55E-02
CHPF2	0.06695	1.29E-01	1.73E-01
CHPF	0.006758	8.78E-01	9.01E-01
CHPT1	-0.12781	3.67E-03	6.73E-03
CHP	-0.25065	8.09E-09	3.12E-08
CHRAC1	0.194499	8.76E-06	2.36E-05
CHRDL1	-0.47394	3.38E-30	8.83E-29
CHRDL2	-0.20848	1.82E-06	5.35E-06
CHRD	-0.12528	4.41E-03	7.99E-03
CHRFAM7A	0.051517	2.43E-01	3.03E-01
CHRM1	-0.3256	3.49E-14	2.36E-13
CHRM2	-0.19024	1.38E-05	3.63E-05
CHRM3	0.035234	4.25E-01	4.91E-01

CHRM4	0.179439	4.21E-05	1.03E-04
CHRM5	0.123431	5.03E-03	9.03E-03
CHRNA10	-0.20292	3.45E-06	9.81E-06
CHRNA1	0.047569	2.81E-01	3.43E-01
CHRNA2	-0.38579	1.01E-19	1.15E-18
CHRNA3	0.042452	3.36E-01	4.02E-01
CHRNA4	-0.14254	1.18E-03	2.35E-03
CHRNA5	0.326822	2.77E-14	1.89E-13
CHRNA6	-0.14655	8.51E-04	1.73E-03
CHRNA7	0.049449	2.63E-01	3.24E-01
CHRNA9	0.263094	1.33E-09	5.62E-09
CHRNB1	0.053828	2.23E-01	2.80E-01
CHRNB2	0.141765	1.26E-03	2.49E-03
CHRNB3	-0.26612	8.49E-10	3.66E-09
CHRNB4	0.184417	2.54E-05	6.43E-05
CHRND	0.117226	7.74E-03	1.34E-02
CHRNE	-0.03612	4.13E-01	4.80E-01
CHRNG	0.045531	3.02E-01	3.66E-01
CHST10	-0.2103	1.47E-06	4.37E-06
CHST11	0.298116	4.97E-12	2.71E-11
CHST12	-0.16345	1.95E-04	4.36E-04
CHST13	-0.02784	5.28E-01	5.92E-01
CHST14	-0.01572	7.22E-01	7.68E-01
CHST15	-0.04287	3.32E-01	3.97E-01
CHST1	0.178427	4.66E-05	1.14E-04
CHST2	-0.07638	8.33E-02	1.17E-01
CHST3	0.056219	2.03E-01	2.58E-01
CHST4	0.07013	1.12E-01	1.52E-01
CHST5	-0.23362	8.17E-08	2.82E-07
CHST6	0.086082	5.09E-02	7.49E-02
CHST7	-0.24373	2.11E-08	7.76E-08
CHST8	-0.2366	5.52E-08	1.93E-07
CHST9	-0.19559	7.78E-06	2.11E-05
CHSY1	0.174802	6.66E-05	1.59E-04
CHSY3	-0.02789	5.28E-01	5.91E-01
CHTF18	0.26693	7.51E-10	3.26E-09
CHTF8	-0.07948	7.15E-02	1.02E-01
CHUK	0.242765	2.41E-08	8.79E-08
CHURC1	-0.15421	4.45E-04	9.40E-04
CIAO1	0.166623	1.45E-04	3.31E-04
CIAPIN1	0.198687	5.54E-06	1.54E-05
CIB1	0.001119	9.80E-01	9.85E-01
CIB2	0.340567	1.88E-15	1.43E-14
CIB3	0.070038	1.12E-01	1.53E-01

CIB4	0.076272	8.38E-02	1.18E-01
CIC	-0.16126	2.38E-04	5.26E-04
CIDEA	-0.05832	1.86E-01	2.40E-01
CIDEB	-0.17268	8.19E-05	1.93E-04
CIDECP	0.149722	6.53E-04	1.35E-03
CIDEC	0.166375	1.49E-04	3.38E-04
CIITA	-0.29185	1.43E-11	7.42E-11
CILP2	0.069743	1.14E-01	1.55E-01
CILP	0.097749	2.65E-02	4.14E-02
CINP	0.168911	1.17E-04	2.71E-04
CIR1	-0.1285	3.49E-03	6.42E-03
CIRBP	-0.59386	2.11E-50	1.44E-48
CIRH1A	0.321684	7.31E-14	4.80E-13
CISD1	0.298971	4.29E-12	2.36E-11
CISD2	0.419317	2.39E-23	3.70E-22
CISD3	0.00057	9.90E-01	9.92E-01
CISH	-0.49848	1.06E-33	3.39E-32
CITED1	-0.07002	1.12E-01	1.53E-01
CITED2	-0.45159	3.03E-27	6.41E-26
CITED4	-0.04123	3.50E-01	4.16E-01
CIT	-0.31874	1.26E-13	8.10E-13
CIZ1	0.033515	4.48E-01	5.14E-01
CKAP2L	0.891289	2.45E-178	6.12E-175
CKAP2	0.694719	1.96E-75	2.24E-73
CKAP4	0.392969	1.83E-20	2.23E-19
CKAP5	0.44974	5.21E-27	1.07E-25
CKB	-0.19968	4.96E-06	1.38E-05
CKLF	0.202006	3.82E-06	1.08E-05
CKMT1A	0.057722	1.91E-01	2.45E-01
CKMT1B	0.101964	2.06E-02	3.30E-02
CKMT2	-0.22146	3.84E-07	1.23E-06
CKM	0.113357	1.00E-02	1.70E-02
CKS1B	0.637395	4.64E-60	3.99E-58
CKS2	0.653873	3.89E-64	3.70E-62
CLASP1	0.017188	6.97E-01	7.46E-01
CLASP2	-0.11598	8.43E-03	1.45E-02
CLCA1	0.046662	2.91E-01	3.53E-01
CLCA2	-0.05963	1.77E-01	2.29E-01
CLCA3P	0.21258	1.13E-06	3.39E-06
CLCA4	-0.03149	4.76E-01	5.41E-01
CLCC1	-0.23099	1.15E-07	3.91E-07
CLCF1	0.061786	1.61E-01	2.11E-01
CLCN1	0.141037	1.33E-03	2.63E-03
CLCN2	0.174014	7.19E-05	1.71E-04

CLCN3	0.022964	6.03E-01	6.60E-01
CLCN4	0.055583	2.08E-01	2.64E-01
CLCN5	-0.13664	1.88E-03	3.62E-03
CLCN6	-0.37867	5.28E-19	5.61E-18
CLCN7	-0.19988	4.85E-06	1.36E-05
CLCNKA	-0.32031	9.45E-14	6.13E-13
CLCNKB	-0.27486	2.22E-10	1.03E-09
CLC	0.056294	2.02E-01	2.57E-01
CLDN10	0.071788	1.04E-01	1.42E-01
CLDN11	-0.07285	9.87E-02	1.36E-01
CLDN12	0.22866	1.55E-07	5.19E-07
CLDN14	0.285793	3.89E-11	1.92E-10
CLDN15	-0.10556	1.66E-02	2.70E-02
CLDN16	-0.17827	4.73E-05	1.16E-04
CLDN17	0.035514	4.21E-01	4.87E-01
CLDN18	-0.30815	8.63E-13	5.11E-12
CLDN19	0.112203	1.08E-02	1.83E-02
CLDN1	-0.02167	6.24E-01	6.80E-01
CLDN20	-0.1162	8.30E-03	1.43E-02
CLDN22	0.015713	7.22E-01	7.68E-01
CLDN23	-0.35023	2.63E-16	2.18E-15
CLDN25	0.047664	2.80E-01	3.42E-01
CLDN2	-0.34603	6.23E-16	4.99E-15
CLDN3	-0.06882	1.19E-01	1.61E-01
CLDN4	-0.03499	4.28E-01	4.94E-01
CLDN5	-0.2168	6.79E-07	2.10E-06
CLDN6	0.02003	6.50E-01	7.04E-01
CLDN7	-0.09245	3.59E-02	5.46E-02
CLDN8	-0.20557	2.55E-06	7.35E-06
CLDN9	-0.11922	6.76E-03	1.18E-02
CLDND1	0.346515	5.64E-16	4.54E-15
CLDND2	-0.15411	4.48E-04	9.48E-04
CLEC10A	-0.19911	5.28E-06	1.47E-05
CLEC11A	-0.08907	4.33E-02	6.47E-02
CLEC12A	-0.12041	6.22E-03	1.10E-02
CLEC12B	-0.17515	6.44E-05	1.54E-04
CLEC14A	-0.40362	1.34E-21	1.78E-20
CLEC16A	-0.38705	7.50E-20	8.62E-19
CLEC17A	-0.14277	1.16E-03	2.30E-03
CLEC18A	-0.03094	4.84E-01	5.49E-01
CLEC18B	-0.01297	7.69E-01	8.08E-01
CLEC18C	0.085976	5.12E-02	7.53E-02
CLEC1A	-0.25068	8.06E-09	3.11E-08
CLEC1B	-0.10135	2.14E-02	3.41E-02

CLEC2A	0.057	1.97E-01	2.51E-01
CLEC2B	0.135713	2.02E-03	3.88E-03
CLEC2D	-0.00334	9.40E-01	9.53E-01
CLEC2L	0.105638	1.65E-02	2.69E-02
CLEC3A	-0.11149	1.13E-02	1.91E-02
CLEC3B	-0.43849	1.30E-25	2.42E-24
CLEC4A	-0.03821	3.87E-01	4.53E-01
CLEC4C	0.057788	1.90E-01	2.44E-01
CLEC4D	0.207055	2.15E-06	6.25E-06
CLEC4E	0.180887	3.64E-05	9.02E-05
CLEC4F	-0.48789	3.73E-32	1.10E-30
CLEC4GP1	-0.14781	7.66E-04	1.57E-03
CLEC4G	-0.05667	1.99E-01	2.54E-01
CLEC4M	-0.01743	6.93E-01	7.42E-01
CLEC5A	-0.03067	4.87E-01	5.52E-01
CLEC6A	0.246693	1.41E-08	5.26E-08
CLEC7A	-0.00888	8.41E-01	8.70E-01
CLEC9A	-0.31871	1.27E-13	8.14E-13
CLECL1	-0.18047	3.80E-05	9.38E-05
CLGN	0.350976	2.25E-16	1.88E-15
CLIC1	0.29702	5.99E-12	3.24E-11
CLIC2	-0.21237	1.15E-06	3.47E-06
CLIC3	-0.41852	2.95E-23	4.52E-22
CLIC4	0.19385	9.40E-06	2.53E-05
CLIC5	-0.4223	1.09E-23	1.73E-22
CLIC6	-0.37115	2.90E-18	2.89E-17
CLINT1	-0.20483	2.78E-06	7.97E-06
CLIP1	0.215411	8.03E-07	2.46E-06
CLIP2	0.061118	1.66E-01	2.16E-01
CLIP3	-0.00942	8.31E-01	8.61E-01
CLIP4	-0.00681	8.77E-01	9.01E-01
CLK1	-0.27425	2.45E-10	1.12E-09
CLK2P	-0.03405	4.41E-01	5.07E-01
CLK2	-0.08108	6.60E-02	9.50E-02
CLK3	-0.13212	2.66E-03	5.00E-03
CLK4	-0.46479	5.81E-29	1.39E-27
CLLU1OS	0.124457	4.68E-03	8.44E-03
CLLU1	0.068627	1.20E-01	1.62E-01
CLMN	0.102575	1.99E-02	3.19E-02
CLN3	-0.31383	3.11E-13	1.93E-12
CLN5	-0.28538	4.16E-11	2.04E-10
CLN6	0.124674	4.60E-03	8.32E-03
CLN8	-0.13205	2.68E-03	5.02E-03
CLNK	-0.10211	2.05E-02	3.27E-02

CLNS1A	0.293878	1.02E-11	5.37E-11
CLOCK	0.089302	4.28E-02	6.40E-02
CLP1	0.155664	3.92E-04	8.36E-04
CLPB	0.368531	5.18E-18	5.03E-17
CLPP	0.133181	2.46E-03	4.64E-03
CLPS	0.096388	2.87E-02	4.45E-02
CLPTM1L	-0.15857	3.03E-04	6.58E-04
CLPTM1	0.028391	5.20E-01	5.84E-01
CLPX	0.091783	3.73E-02	5.65E-02
CLRN1OS	-0.07972	7.07E-02	1.01E-01
CLRN1	0.026633	5.46E-01	6.09E-01
CLRN2	-0.08452	5.53E-02	8.07E-02
CLRN3	-0.10965	1.28E-02	2.13E-02
CLSPN	0.778139	1.10E-105	1.94E-103
CLSTN1	0.097269	2.73E-02	4.25E-02
CLSTN2	0.009124	8.36E-01	8.66E-01
CLSTN3	0.050956	2.48E-01	3.09E-01
CLTA	0.131802	2.73E-03	5.11E-03
CLTB	-0.02928	5.07E-01	5.72E-01
CLTCL1	0.236013	5.97E-08	2.08E-07
CLTC	0.218314	5.65E-07	1.77E-06
CLUAP1	-0.41887	2.70E-23	4.15E-22
CLUL1	-0.42077	1.63E-23	2.56E-22
CLU	-0.45409	1.45E-27	3.14E-26
CLVS1	0.021537	6.26E-01	6.82E-01
CLVS2	0.161529	2.32E-04	5.14E-04
CLYBL	-0.21311	1.06E-06	3.19E-06
CMA1	-0.3489	3.45E-16	2.83E-15
CMAH	-0.46501	5.44E-29	1.31E-27
CMAS	0.507413	4.76E-35	1.63E-33
CMBL	0.102834	1.96E-02	3.14E-02
CMC1	0.169494	1.11E-04	2.57E-04
CMIP	0.038812	3.79E-01	4.46E-01
CMKLR1	-0.06625	1.33E-01	1.78E-01
CMPK1	-0.09047	4.01E-02	6.04E-02
CMPK2	0.132426	2.60E-03	4.89E-03
CMTM1	0.207339	2.08E-06	6.06E-06
CMTM2	-0.0381	3.88E-01	4.55E-01
CMTM3	0.193291	9.98E-06	2.67E-05
CMTM4	-0.20413	3.00E-06	8.60E-06
CMTM5	-0.35211	1.78E-16	1.50E-15
CMTM6	0.085739	5.18E-02	7.62E-02
CMTM7	-0.23703	5.21E-08	1.83E-07
CMTM8	-0.09954	2.39E-02	3.76E-02

CMYA5	-0.27757	1.45E-10	6.82E-10
CN5H6.4	0.104906	1.72E-02	2.80E-02
CNBD1	0.034555	4.34E-01	5.00E-01
CNBP	0.238024	4.57E-08	1.62E-07
CNDP1	-0.00622	8.88E-01	9.09E-01
CNDP2	-0.15007	6.34E-04	1.31E-03
CNFN	0.05878	1.83E-01	2.36E-01
CNGA1	-0.13625	1.94E-03	3.73E-03
CNGA2	0.086586	4.95E-02	7.31E-02
CNGA3	-0.21801	5.86E-07	1.83E-06
CNGA4	-0.33733	3.59E-15	2.66E-14
CNGB1	-0.14821	7.41E-04	1.52E-03
CNGB3	0.204531	2.87E-06	8.23E-06
CNIH2	0.352454	1.65E-16	1.40E-15
CNIH3	-0.02945	5.05E-01	5.69E-01
CNIH4	-0.10362	1.87E-02	3.01E-02
CNIH	0.298993	4.27E-12	2.35E-11
CNKSR1	-0.30188	2.59E-12	1.46E-11
CNKSR2	-0.3116	4.65E-13	2.83E-12
CNKSR3	0.097881	2.63E-02	4.11E-02
CNN1	-0.17313	7.84E-05	1.85E-04
CNN2	-0.14174	1.26E-03	2.49E-03
CNN3	0.014824	7.37E-01	7.81E-01
CNNM1	-0.03659	4.07E-01	4.74E-01
CNNM2	-0.17207	8.68E-05	2.04E-04
CNNM3	-0.38637	8.81E-20	1.01E-18
CNNM4	0.034252	4.38E-01	5.05E-01
CNOT10	0.252527	6.20E-09	2.43E-08
CNOT1	0.024524	5.79E-01	6.38E-01
CNOT2	0.129706	3.19E-03	5.92E-03
CNOT3	0.087246	4.78E-02	7.08E-02
CNOT4	0.014755	7.38E-01	7.82E-01
CNOT6L	-0.18616	2.12E-05	5.44E-05
CNOT6	0.007063	8.73E-01	8.97E-01
CNOT7	0.14925	6.79E-04	1.40E-03
CNOT8	-0.19835	5.75E-06	1.59E-05
CNO	0.173397	7.64E-05	1.81E-04
CNPY1	0.006035	8.91E-01	9.12E-01
CNPY2	0.177875	4.92E-05	1.20E-04
CNPY3	0.031159	4.80E-01	5.46E-01
CNPY4	0.099812	2.35E-02	3.71E-02
CNP	0.318267	1.38E-13	8.82E-13
CNR1	-0.29858	4.58E-12	2.51E-11
CNR2	-0.22376	2.88E-07	9.36E-07

CNRIP1	-0.26051	1.95E-09	8.08E-09
CNST	-0.24517	1.74E-08	6.43E-08
CNTD1	-0.24206	2.65E-08	9.64E-08
CNTD2	0.170688	9.91E-05	2.31E-04
CNTFR	-0.38349	1.73E-19	1.94E-18
CNTF	-0.00619	8.89E-01	9.10E-01
CNTLN	0.229791	1.34E-07	4.53E-07
CNTN1	0.025781	5.59E-01	6.20E-01
CNTN2	-0.1587	2.99E-04	6.51E-04
CNTN3	-0.33354	7.57E-15	5.43E-14
CNTN4	-0.31941	1.12E-13	7.19E-13
CNTN5	-0.02652	5.48E-01	6.10E-01
CNTN6	-0.34623	5.99E-16	4.81E-15
CNTNAP1	-0.13867	1.61E-03	3.12E-03
CNTNAP2	0.14944	6.68E-04	1.38E-03
CNTNAP3	0.007444	8.66E-01	8.91E-01
CNTNAP4	0.193803	9.45E-06	2.54E-05
CNTNAP5	0.074022	9.33E-02	1.30E-01
CNTROB	-0.3898	3.91E-20	4.63E-19
COASY	0.210634	1.42E-06	4.21E-06
COBLL1	-0.36427	1.32E-17	1.24E-16
COBL	-0.13366	2.37E-03	4.48E-03
COBRA1	0.106325	1.58E-02	2.58E-02
COCH	0.430492	1.19E-24	2.05E-23
COG1	-0.09796	2.62E-02	4.09E-02
COG2	-0.08807	4.58E-02	6.81E-02
COG3	-0.11638	8.20E-03	1.41E-02
COG4	-0.08628	5.04E-02	7.42E-02
COG5	0.300523	3.28E-12	1.83E-11
COG6	0.084363	5.57E-02	8.14E-02
COG7	-0.34143	1.58E-15	1.21E-14
COG8	-0.20892	1.73E-06	5.10E-06
COIL	0.3531	1.44E-16	1.23E-15
COL10A1	-0.02234	6.13E-01	6.70E-01
COL11A1	0.401327	2.37E-21	3.10E-20
COL11A2	-0.02517	5.69E-01	6.29E-01
COL12A1	0.260229	2.04E-09	8.40E-09
COL13A1	-0.24087	3.12E-08	1.12E-07
COL14A1	-0.33703	3.81E-15	2.82E-14
COL15A1	0.143915	1.06E-03	2.11E-03
COL16A1	-0.13458	2.21E-03	4.20E-03
COL17A1	-0.10401	1.82E-02	2.94E-02
COL18A1	-0.07349	9.57E-02	1.32E-01
COL19A1	-0.19051	1.34E-05	3.54E-05

COL1A1	0.212975	1.07E-06	3.24E-06
COL1A2	0.159569	2.77E-04	6.06E-04
COL20A1	0.115988	8.42E-03	1.45E-02
COL21A1	-0.32004	9.92E-14	6.43E-13
COL22A1	0.16601	1.54E-04	3.49E-04
COL23A1	-0.06836	1.21E-01	1.64E-01
COL24A1	-0.04748	2.82E-01	3.44E-01
COL25A1	-0.1836	2.76E-05	6.96E-05
COL27A1	-0.26069	1.90E-09	7.88E-09
COL28A1	-0.20655	2.28E-06	6.60E-06
COL29A1	-0.31954	1.09E-13	7.03E-13
COL2A1	0.119315	6.71E-03	1.18E-02
COL3A1	0.21987	4.67E-07	1.48E-06
COL4A1	0.120614	6.13E-03	1.08E-02
COL4A2	0.043968	3.19E-01	3.84E-01
COL4A3BP	-0.29731	5.70E-12	3.09E-11
COL4A3	-0.44193	4.93E-26	9.51E-25
COL4A4	-0.41514	7.13E-23	1.05E-21
COL4A5	-0.16569	1.59E-04	3.59E-04
COL4A6	-0.17541	6.28E-05	1.51E-04
COL5A1	0.125432	4.36E-03	7.91E-03
COL5A2	0.268462	5.96E-10	2.62E-09
COL5A3	0.162256	2.18E-04	4.84E-04
COL6A1	0.104524	1.77E-02	2.86E-02
COL6A2	0.07907	7.30E-02	1.04E-01
COL6A3	0.05305	2.29E-01	2.88E-01
COL6A4P2	-0.17865	4.56E-05	1.12E-04
COL6A6	-0.36573	9.60E-18	9.15E-17
COL7A1	0.253217	5.62E-09	2.21E-08
COL8A1	-0.10234	2.02E-02	3.23E-02
COL8A2	-0.08155	6.44E-02	9.30E-02
COL9A1	-0.09743	2.70E-02	4.21E-02
COL9A2	-0.23733	5.01E-08	1.76E-07
COL9A3	0.020898	6.36E-01	6.91E-01
COLEC10	-0.05771	1.91E-01	2.45E-01
COLEC11	-0.04199	3.42E-01	4.07E-01
COLEC12	-0.33604	4.64E-15	3.41E-14
COLQ	-0.22531	2.38E-07	7.78E-07
COMMD10	-0.00211	9.62E-01	9.70E-01
COMMD1	0.148733	7.09E-04	1.46E-03
COMMD2	0.314533	2.74E-13	1.70E-12
COMMD3	0.062699	1.55E-01	2.04E-01
COMMD4	0.112394	1.07E-02	1.81E-02
COMMD5	0.091395	3.81E-02	5.76E-02

COMMD6	-0.14682	8.32E-04	1.69E-03
COMMD7	0.069873	1.13E-01	1.54E-01
COMMD8	0.342429	1.30E-15	9.99E-15
COMMD9	0.099355	2.41E-02	3.80E-02
COMP	-0.13876	1.60E-03	3.10E-03
COMTD1	0.177364	5.18E-05	1.26E-04
COMT	-0.14377	1.07E-03	2.14E-03
COPA	0.071222	1.06E-01	1.45E-01
COPB1	0.156953	3.50E-04	7.53E-04
COPB2	0.333541	7.57E-15	5.43E-14
COPE	0.12748	3.76E-03	6.88E-03
COPG2	0.129146	3.33E-03	6.15E-03
COPG	0.199384	5.13E-06	1.43E-05
COPS2	0.177582	5.07E-05	1.23E-04
COPS3	0.335636	5.02E-15	3.67E-14
COPS4	0.091593	3.77E-02	5.70E-02
COPS5	0.227273	1.85E-07	6.14E-07
COPS6	0.398714	4.52E-21	5.81E-20
COPS7A	0.053645	2.24E-01	2.82E-01
COPS7B	0.295583	7.64E-12	4.08E-11
COPS8	0.397934	5.48E-21	6.98E-20
COPZ1	0.213687	9.87E-07	2.99E-06
COPZ2	0.001855	9.67E-01	9.74E-01
COQ10A	-0.10079	2.22E-02	3.52E-02
COQ10B	0.118767	6.97E-03	1.22E-02
COQ2	0.266392	8.15E-10	3.52E-09
COQ3	0.345756	6.59E-16	5.27E-15
COQ4	-0.33215	9.93E-15	7.06E-14
COQ5	0.299557	3.88E-12	2.15E-11
COQ6	-0.10185	2.08E-02	3.32E-02
COQ7	-0.28321	5.92E-11	2.87E-10
COQ9	-0.03368	4.46E-01	5.12E-01
CORIN	-0.04843	2.73E-01	3.35E-01
CORO1A	-0.05077	2.50E-01	3.11E-01
CORO1B	0.025773	5.60E-01	6.20E-01
CORO1C	0.400346	3.03E-21	3.93E-20
CORO2A	-0.21074	1.40E-06	4.16E-06
CORO2B	-0.46783	2.28E-29	5.63E-28
CORO6	0.112272	1.08E-02	1.82E-02
CORO7	-0.17792	4.90E-05	1.19E-04
CORT	-0.06817	1.22E-01	1.65E-01
COTL1	0.135517	2.06E-03	3.93E-03
COX10	0.064818	1.42E-01	1.88E-01
COX11	0.060981	1.67E-01	2.18E-01

COX15	0.047998	2.77E-01	3.39E-01
COX16	0.064215	1.46E-01	1.92E-01
COX17	0.226458	2.06E-07	6.78E-07
COX18	-0.11707	7.83E-03	1.35E-02
COX19	-0.0057	8.97E-01	9.17E-01
COX4I1	0.014801	7.38E-01	7.81E-01
COX4I2	-0.33698	3.85E-15	2.84E-14
COX4NB	0.31645	1.93E-13	1.22E-12
COX5A	0.240272	3.38E-08	1.21E-07
COX5B	0.194599	8.67E-06	2.34E-05
COX6A1	0.23646	5.63E-08	1.97E-07
COX6A2	0.12304	5.17E-03	9.26E-03
COX6B1	0.260753	1.89E-09	7.82E-09
COX6B2	0.158225	3.13E-04	6.78E-04
COX6C	0.104116	1.81E-02	2.93E-02
COX7A1	-0.28852	2.49E-11	1.26E-10
COX7A2L	0.281251	8.10E-11	3.89E-10
COX7A2	0.269672	4.95E-10	2.20E-09
COX7B2	0.261473	1.70E-09	7.06E-09
COX7B	0.214208	9.28E-07	2.82E-06
COX7C	-0.05454	2.17E-01	2.73E-01
COX8A	0.241415	2.90E-08	1.05E-07
COX8C	0.023712	5.91E-01	6.49E-01
CP110	-0.16916	1.15E-04	2.65E-04
CPA1	0.004731	9.15E-01	9.32E-01
CPA2	0.090157	4.08E-02	6.14E-02
CPA3	-0.30448	1.65E-12	9.49E-12
CPA4	0.268335	6.07E-10	2.66E-09
CPA5	0.088846	4.39E-02	6.55E-02
CPA6	-0.15525	4.06E-04	8.64E-04
CPAMD8	-0.52574	6.08E-38	2.49E-36
CPB1	0.03857	3.82E-01	4.49E-01
CPB2	-0.3341	6.79E-15	4.90E-14
CPD	0.411365	1.89E-22	2.69E-21
CPEB1	-0.14137	1.30E-03	2.56E-03
CPEB2	-0.27699	1.59E-10	7.44E-10
CPEB3	-0.35088	2.29E-16	1.91E-15
CPEB4	-0.3352	5.46E-15	3.99E-14
CPE	0.191267	1.24E-05	3.28E-05
CPLX1	0.166443	1.48E-04	3.36E-04
CPLX2	0.143671	1.08E-03	2.15E-03
CPLX3	0.039493	3.71E-01	4.37E-01
CPLX4	0.113188	1.01E-02	1.72E-02
CPM	-0.31107	5.12E-13	3.10E-12

CPN1	0.144719	9.89E-04	1.99E-03
CPN2	0.072313	1.01E-01	1.39E-01
CPNE1	0.151993	5.38E-04	1.12E-03
CPNE2	-0.34542	7.07E-16	5.63E-15
CPNE3	-0.09838	2.56E-02	4.00E-02
CPNE4	-0.04023	3.62E-01	4.29E-01
CPNE5	-0.06691	1.29E-01	1.73E-01
CPNE6	-0.00098	9.82E-01	9.86E-01
CPNE7	-0.10714	1.50E-02	2.46E-02
CPNE8	0.081708	6.39E-02	9.23E-02
CPNE9	-0.12059	6.15E-03	1.09E-02
CPOX	0.225577	2.30E-07	7.54E-07
CPO	-0.25199	6.69E-09	2.61E-08
CPPED1	-0.03653	4.08E-01	4.75E-01
CPS1	0.131728	2.74E-03	5.14E-03
CPSF1	-0.00202	9.64E-01	9.71E-01
CPSF2	0.398178	5.16E-21	6.60E-20
CPSF3L	-0.12948	3.24E-03	6.01E-03
CPSF3	0.625405	3.02E-57	2.46E-55
CPSF4L	0.130838	2.93E-03	5.47E-03
CPSF4	0.370265	3.53E-18	3.49E-17
CPSF6	0.176534	5.62E-05	1.36E-04
CPSF7	0.078595	7.47E-02	1.06E-01
CPT1A	-0.05967	1.76E-01	2.28E-01
CPT1B	-0.14407	1.04E-03	2.09E-03
CPT1C	0.263702	1.22E-09	5.16E-09
CPT2	-0.29635	6.71E-12	3.61E-11
CPVL	-0.07841	7.54E-02	1.07E-01
CPXCR1	0.028804	5.14E-01	5.78E-01
CPXM1	0.216311	7.21E-07	2.23E-06
CPXM2	-0.17283	8.07E-05	1.91E-04
CPZ	-0.07283	9.88E-02	1.36E-01
CP	0.066101	1.34E-01	1.79E-01
CR1L	0.170839	9.77E-05	2.28E-04
CR1	-0.16771	1.31E-04	3.01E-04
CR2	-0.18209	3.23E-05	8.04E-05
CRABP1	0.211458	1.29E-06	3.84E-06
CRABP2	0.125853	4.23E-03	7.69E-03
CRADD	-0.08682	4.89E-02	7.23E-02
CRAMP1L	-0.28617	3.66E-11	1.81E-10
CRAT	-0.14754	7.84E-04	1.60E-03
CRB1	-0.10253	1.99E-02	3.20E-02
CRB2	-0.18618	2.12E-05	5.43E-05
CRB3	-0.23728	5.04E-08	1.77E-07

CRBN	-0.23471	7.09E-08	2.46E-07
CRCP	0.114442	9.34E-03	1.59E-02
CRCT1	0.067973	1.23E-01	1.66E-01
CREB1	-0.06953	1.15E-01	1.56E-01
CREB3L1	-0.25677	3.37E-09	1.36E-08
CREB3L2	0.00999	8.21E-01	8.53E-01
CREB3L3	0.103974	1.83E-02	2.95E-02
CREB3L4	-0.11405	9.58E-03	1.63E-02
CREB3	0.024092	5.85E-01	6.44E-01
CREB5	-0.02532	5.66E-01	6.26E-01
CREBBP	-0.27398	2.55E-10	1.17E-09
CREBL2	-0.31328	3.43E-13	2.12E-12
CREBZF	-0.06124	1.65E-01	2.15E-01
CREG1	-0.15884	2.96E-04	6.44E-04
CREG2	0.306872	1.08E-12	6.32E-12
CRELD1	-0.36635	8.38E-18	8.01E-17
CRELD2	-0.08949	4.24E-02	6.34E-02
CREM	0.217977	5.89E-07	1.84E-06
CRHBP	-0.26977	4.88E-10	2.16E-09
CRHR1	-0.01104	8.03E-01	8.37E-01
CRHR2	-0.1673	1.37E-04	3.12E-04
CRH	-0.04194	3.42E-01	4.08E-01
CRIM1	-0.17352	7.55E-05	1.79E-04
CRIP1	-0.17086	9.76E-05	2.28E-04
CRIP2	-0.22378	2.88E-07	9.35E-07
CRIP3	0.009461	8.30E-01	8.60E-01
CRIPAK	-0.2297	1.36E-07	4.58E-07
CRIPT	0.302204	2.45E-12	1.39E-11
CRISP1	-0.02113	6.32E-01	6.88E-01
CRISP2	-0.12814	3.58E-03	6.58E-03
CRISP3	0.023858	5.89E-01	6.47E-01
CRISPLD1	-0.01349	7.60E-01	8.00E-01
CRISPLD2	-0.00263	9.53E-01	9.62E-01
CRKL	0.196338	7.17E-06	1.96E-05
CRK	-0.0473	2.84E-01	3.46E-01
CRLF1	-0.26395	1.18E-09	4.98E-09
CRLF2	0.061511	1.63E-01	2.13E-01
CRLF3	0.199665	4.97E-06	1.39E-05
CRLS1	-0.21822	5.71E-07	1.79E-06
CRMP1	-0.10643	1.57E-02	2.57E-02
CRNKL1	0.118203	7.25E-03	1.26E-02
CRNN	0.097322	2.72E-02	4.23E-02
CROCCL1	-0.2605	1.96E-09	8.10E-09
CROCCL2	-0.36939	4.29E-18	4.21E-17

CROCC	-0.35949	3.72E-17	3.39E-16
CROT	-0.10589	1.62E-02	2.65E-02
CRP	0.092519	3.58E-02	5.44E-02
CRTAC1	-0.38636	8.82E-20	1.01E-18
CRTAM	-0.00324	9.41E-01	9.54E-01
CRTAP	-0.26223	1.52E-09	6.36E-09
CRTC1	-0.39435	1.31E-20	1.61E-19
CRTC2	-0.07602	8.48E-02	1.19E-01
CRTC3	-0.0995	2.39E-02	3.77E-02
CRX	0.056546	2.00E-01	2.55E-01
CRY1	0.206685	2.24E-06	6.51E-06
CRY2	-0.70181	1.36E-77	1.62E-75
CRYAA	-0.06015	1.73E-01	2.25E-01
CRYAB	-0.17949	4.19E-05	1.03E-04
CRYBA1	-0.07732	7.96E-02	1.12E-01
CRYBA2	0.210983	1.36E-06	4.05E-06
CRYBA4	-0.0054	9.03E-01	9.21E-01
CRYBB1	-0.05497	2.13E-01	2.69E-01
CRYBB2	-0.0607	1.69E-01	2.20E-01
CRYBB3	-0.12042	6.22E-03	1.10E-02
CRYBG3	0.001108	9.80E-01	9.85E-01
CRYGA	0.019327	6.62E-01	7.14E-01
CRYGB	0.114185	9.50E-03	1.62E-02
CRYGC	0.08761	4.69E-02	6.95E-02
CRYGD	0.049852	2.59E-01	3.20E-01
CRYGN	-0.00317	9.43E-01	9.55E-01
CRYGS	-0.2061	2.40E-06	6.93E-06
CRYL1	-0.22951	1.39E-07	4.69E-07
CRYM	-0.4807	3.90E-31	1.08E-29
CRYZL1	-0.12024	6.29E-03	1.11E-02
CRYZ	-0.14738	7.94E-04	1.62E-03
CSAD	-0.31504	2.50E-13	1.56E-12
CSAG1	0.314132	2.94E-13	1.83E-12
CSAG2	0.2809	8.57E-11	4.10E-10
CSAG3	0.363746	1.48E-17	1.39E-16
CSDAP1	0.120756	6.07E-03	1.07E-02
CSDA	0.11933	6.71E-03	1.17E-02
CSDC2	-0.28232	6.82E-11	3.29E-10
CSDE1	0.041436	3.48E-01	4.14E-01
CSE1L	0.570174	9.75E-46	5.84E-44
CSF1R	-0.09248	3.59E-02	5.46E-02
CSF1	-0.04639	2.93E-01	3.56E-01
CSF2RA	-0.05522	2.11E-01	2.67E-01
CSF2RB	-0.10853	1.37E-02	2.27E-02

CSF2	-0.14076	1.36E-03	2.68E-03
CSF3R	-0.17724	5.24E-05	1.27E-04
CSF3	-0.02952	5.04E-01	5.68E-01
CSGALNACT1	0.025518	5.63E-01	6.24E-01
CSGALNACT2	-0.04873	2.70E-01	3.32E-01
CSH1	0.098207	2.58E-02	4.04E-02
CSH2	0.183148	2.89E-05	7.27E-05
CSK	0.118237	7.23E-03	1.26E-02
CSMD1	-0.10501	1.71E-02	2.78E-02
CSMD2	0.152139	5.31E-04	1.11E-03
CSMD3	0.012991	7.69E-01	8.08E-01
CSN1S1	0.00217	9.61E-01	9.69E-01
CSN2	0.000344	9.94E-01	9.95E-01
CSN3	-0.12118	5.90E-03	1.05E-02
CSNK1A1L	0.001583	9.71E-01	9.78E-01
CSNK1A1P	0.067911	1.24E-01	1.67E-01
CSNK1A1	-0.05815	1.88E-01	2.41E-01
CSNK1D	-0.10772	1.45E-02	2.38E-02
CSNK1E	0.110991	1.17E-02	1.97E-02
CSNK1G1	0.110008	1.25E-02	2.08E-02
CSNK1G2	0.096625	2.83E-02	4.40E-02
CSNK1G3	-0.02577	5.60E-01	6.20E-01
CSNK2A1P	0.25458	4.63E-09	1.83E-08
CSNK2A1	0.240359	3.34E-08	1.20E-07
CSNK2A2	0.073266	9.67E-02	1.34E-01
CSNK2B	0.223043	3.16E-07	1.02E-06
CSPG4PY2	0.037146	4.00E-01	4.67E-01
CSPG4	-0.12318	5.12E-03	9.18E-03
CSPG5	0.004244	9.23E-01	9.39E-01
CSPP1	-0.01914	6.65E-01	7.17E-01
CSRNP1	-0.34558	6.83E-16	5.45E-15
CSRNP2	0.162208	2.18E-04	4.85E-04
CSRNP3	0.030725	4.87E-01	5.51E-01
CSRP1	-0.29127	1.58E-11	8.13E-11
CSRP2BP	-0.07537	8.75E-02	1.22E-01
CSRP2	0.273407	2.79E-10	1.28E-09
CSRP3	0.118155	7.27E-03	1.26E-02
CST11	0.062698	1.55E-01	2.04E-01
CST1	-0.03062	4.88E-01	5.53E-01
CST2	-0.18337	2.83E-05	7.11E-05
CST3	-0.5072	5.14E-35	1.76E-33
CST4	-0.00791	8.58E-01	8.84E-01
CST5	-0.50794	3.95E-35	1.36E-33
CST6	0.04291	3.31E-01	3.97E-01

CST7	-0.04569	3.01E-01	3.64E-01
CST9L	0.062751	1.55E-01	2.04E-01
CST9	0.069646	1.14E-01	1.55E-01
CSTA	0.036614	4.07E-01	4.73E-01
CSTB	0.087019	4.84E-02	7.16E-02
CSTF1	0.182253	3.17E-05	7.92E-05
CSTF2T	0.039671	3.69E-01	4.35E-01
CSTF2	0.481031	3.51E-31	9.72E-30
CSTF3	0.439185	1.07E-25	2.00E-24
CSTL1	-0.04752	2.82E-01	3.44E-01
CSTT	0.008187	8.53E-01	8.80E-01
CS	0.321697	7.29E-14	4.79E-13
CT45A1	0.12867	3.44E-03	6.35E-03
CT45A2	0.187335	1.88E-05	4.85E-05
CT45A3	0.141586	1.28E-03	2.52E-03
CT45A4	0.186898	1.97E-05	5.06E-05
CT45A5	0.207471	2.05E-06	5.97E-06
CT45A6	0.123108	5.15E-03	9.22E-03
CT47A11	0.034575	4.34E-01	5.00E-01
CT47A1	0.078883	7.37E-02	1.05E-01
CT47A2	0.108026	1.42E-02	2.34E-02
CT47A6	0.122289	5.45E-03	9.73E-03
CT47B1	0.120879	6.02E-03	1.07E-02
CT62	-0.13653	1.90E-03	3.65E-03
CTAG1B	0.24483	1.82E-08	6.73E-08
CTAG2	0.234697	7.10E-08	2.46E-07
CTAGE1	0.055175	2.11E-01	2.67E-01
CTAGE4	-0.09763	2.67E-02	4.17E-02
CTAGE5	0.047117	2.86E-01	3.48E-01
CTAGE6	-0.09071	3.96E-02	5.97E-02
CTAGE9	-0.07868	7.44E-02	1.06E-01
CTBP1	-0.09318	3.45E-02	5.26E-02
CTBP2	0.16904	1.16E-04	2.68E-04
CTBS	0.128161	3.58E-03	6.57E-03
CTCFL	0.249055	1.01E-08	3.86E-08
CTCF	0.019212	6.64E-01	7.16E-01
CTDP1	-0.06303	1.53E-01	2.01E-01
CTDSP1	-0.42478	5.61E-24	9.16E-23
CTDSP2	-0.15373	4.63E-04	9.77E-04
CTDSPL2	0.299359	4.01E-12	2.22E-11
CTDSPL	-0.4642	6.96E-29	1.66E-27
CTF1	-0.43793	1.52E-25	2.81E-24
CTGF	-0.16874	1.19E-04	2.75E-04
CTHRC1	0.41198	1.61E-22	2.32E-21

CTH	0.011498	7.95E-01	8.30E-01
CTLA4	0.084435	5.55E-02	8.11E-02
CTNNA1	-0.19347	9.79E-06	2.62E-05
CTNNA2	0.027736	5.30E-01	5.93E-01
CTNNA3	-0.0866	4.95E-02	7.31E-02
CTNNAL1	0.23187	1.03E-07	3.50E-07
CTNNB1	0.002334	9.58E-01	9.67E-01
CTNNBIP1	-0.34057	1.88E-15	1.43E-14
CTNNBL1	0.291771	1.45E-11	7.51E-11
CTNND1	-0.0909	3.92E-02	5.91E-02
CTNND2	-0.01819	6.81E-01	7.31E-01
CTNS	-0.255	4.35E-09	1.73E-08
CTPS2	-0.00278	9.50E-01	9.61E-01
CTPS	0.529676	1.38E-38	5.85E-37
CTR9	-0.07565	8.63E-02	1.21E-01
CTRB1	0.037997	3.90E-01	4.56E-01
CTRB2	-0.0129	7.70E-01	8.09E-01
CTRC	0.019432	6.60E-01	7.13E-01
CTRL	0.087609	4.69E-02	6.95E-02
CTSA	0.048189	2.75E-01	3.37E-01
CTSB	0.194887	8.40E-06	2.27E-05
CTSC	0.181299	3.49E-05	8.68E-05
CTSD	-0.36641	8.27E-18	7.92E-17
CTSE	-0.34675	5.37E-16	4.33E-15
CTSF	-0.34547	6.99E-16	5.57E-15
CTSG	-0.39468	1.21E-20	1.50E-19
CTSH	-0.56024	6.84E-44	3.81E-42
CTSK	0.054368	2.18E-01	2.75E-01
CTSL1	0.345335	7.18E-16	5.72E-15
CTSL2	0.678114	1.28E-70	1.35E-68
CTSL3	0.053367	2.27E-01	2.85E-01
CTSO	-0.39558	9.72E-21	1.21E-19
CTSS	-0.15503	4.14E-04	8.80E-04
CTSW	-0.07323	9.69E-02	1.34E-01
CTSZ	-0.20996	1.53E-06	4.54E-06
CTTNBP2NL	-0.02519	5.68E-01	6.28E-01
CTTNBP2	-0.20007	4.75E-06	1.33E-05
CTTN	-0.11523	8.86E-03	1.52E-02
CTU1	0.123262	5.09E-03	9.13E-03
CTU2	0.199428	5.10E-06	1.42E-05
CTXN1	0.132851	2.52E-03	4.74E-03
CTXN2	0.146282	8.70E-04	1.76E-03
CTXN3	-0.03645	4.09E-01	4.76E-01
CUBN	-0.35863	4.47E-17	4.03E-16

CUEDC1	0.009548	8.29E-01	8.59E-01
CUEDC2	0.016537	7.08E-01	7.56E-01
CUL1	0.201645	3.98E-06	1.12E-05
CUL2	0.453522	1.72E-27	3.70E-26
CUL3	0.057784	1.90E-01	2.44E-01
CUL4A	0.016005	7.17E-01	7.64E-01
CUL4B	0.290195	1.89E-11	9.65E-11
CUL5	-0.04712	2.86E-01	3.48E-01
CUL7	-0.04049	3.59E-01	4.25E-01
CUL9	-0.2421	2.64E-08	9.59E-08
CUTA	-0.19319	1.01E-05	2.70E-05
CUTC	-0.01993	6.52E-01	7.05E-01
CUX1	-0.15988	2.70E-04	5.91E-04
CUX2	-0.06554	1.37E-01	1.83E-01
CUZD1	0.071756	1.04E-01	1.42E-01
CWC15	0.139697	1.48E-03	2.90E-03
CWC22	0.200249	4.65E-06	1.30E-05
CWC25	-0.10395	1.83E-02	2.95E-02
CWC27	0.215527	7.92E-07	2.43E-06
CWF19L1	0.201543	4.03E-06	1.14E-05
CWF19L2	-0.05625	2.02E-01	2.58E-01
CWH43	-0.16942	1.12E-04	2.59E-04
CX3CL1	-0.25824	2.73E-09	1.11E-08
CX3CR1	-0.45022	4.54E-27	9.40E-26
CXADRP2	-0.05623	2.03E-01	2.58E-01
CXADRP3	0.003384	9.39E-01	9.52E-01
CXADR	-0.09677	2.81E-02	4.36E-02
CXCL10	0.349667	2.95E-16	2.43E-15
CXCL11	0.269766	4.88E-10	2.17E-09
CXCL12	-0.17809	4.82E-05	1.18E-04
CXCL13	0.07186	1.03E-01	1.42E-01
CXCL14	-0.23759	4.84E-08	1.70E-07
CXCL16	-0.43735	1.79E-25	3.28E-24
CXCL17	-0.3922	2.20E-20	2.66E-19
CXCL1	0.087637	4.68E-02	6.95E-02
CXCL2	-0.1341	2.29E-03	4.34E-03
CXCL3	0.040502	3.59E-01	4.25E-01
CXCL5	0.215226	8.21E-07	2.52E-06
CXCL6	0.146674	8.42E-04	1.71E-03
CXCL9	0.212992	1.07E-06	3.23E-06
CXCR1	-0.09279	3.53E-02	5.37E-02
CXCR2P1	0.129555	3.23E-03	5.98E-03
CXCR2	-0.16431	1.80E-04	4.05E-04
CXCR3	-0.02329	5.98E-01	6.56E-01

CXCR4	-0.04456	3.13E-01	3.77E-01
CXCR5	-0.15561	3.93E-04	8.40E-04
CXCR6	0.043285	3.27E-01	3.92E-01
CXCR7	0.12745	3.77E-03	6.90E-03
CXXC1	-0.09649	2.86E-02	4.43E-02
CXXC4	-0.13231	2.62E-03	4.93E-03
CXXC5	-0.18756	1.83E-05	4.74E-05
CXorf1	-0.18731	1.88E-05	4.86E-05
CXorf21	-0.22552	2.31E-07	7.59E-07
CXorf22	-0.20681	2.21E-06	6.42E-06
CXorf23	-0.30193	2.57E-12	1.45E-11
CXorf26	0.152431	5.18E-04	1.09E-03
CXorf27	0.076176	8.42E-02	1.18E-01
CXorf30	-0.2435	2.18E-08	7.99E-08
CXorf36	-0.27374	2.65E-10	1.21E-09
CXorf38	0.125127	4.46E-03	8.07E-03
CXorf40A	0.089307	4.28E-02	6.40E-02
CXorf40B	0.25242	6.29E-09	2.46E-08
CXorf41	-0.25334	5.52E-09	2.17E-08
CXorf42	-0.18369	2.74E-05	6.90E-05
CXorf48	0.130208	3.07E-03	5.71E-03
CXorf49B	0.085826	5.16E-02	7.58E-02
CXorf50B	-0.3182	1.40E-13	8.92E-13
CXorf51	0.083374	5.87E-02	8.53E-02
CXorf56	0.161629	2.30E-04	5.10E-04
CXorf57	-0.02109	6.33E-01	6.88E-01
CXorf58	0.080112	6.93E-02	9.93E-02
CXorf59	-0.22978	1.35E-07	4.54E-07
CXorf61	0.121085	5.94E-03	1.05E-02
CXorf64	-0.1463	8.69E-04	1.76E-03
CXorf65	-0.08675	4.91E-02	7.25E-02
CXorf66	0.054721	2.15E-01	2.72E-01
CYB561D1	-0.15517	4.09E-04	8.70E-04
CYB561D2	-0.14074	1.36E-03	2.68E-03
CYB561	-0.22857	1.57E-07	5.25E-07
CYB5A	-0.4328	6.33E-25	1.11E-23
CYB5B	0.068241	1.22E-01	1.64E-01
CYB5D1	-0.31087	5.31E-13	3.21E-12
CYB5D2	-0.42267	9.86E-24	1.58E-22
CYB5R1	-0.37614	9.40E-19	9.76E-18
CYB5R2	-0.03362	4.46E-01	5.13E-01
CYB5R3	-0.40473	1.02E-21	1.36E-20
CYB5R4	0.198346	5.75E-06	1.59E-05
CYB5RL	-0.03321	4.52E-01	5.18E-01

CYBASC3	-0.18735	1.87E-05	4.84E-05
CYBA	-0.06782	1.24E-01	1.67E-01
CYBB	-0.03626	4.12E-01	4.78E-01
CYBRD1	-0.53357	3.12E-39	1.38E-37
CYC1	0.271743	3.61E-10	1.63E-09
CYCSP52	-0.10022	2.29E-02	3.63E-02
CYCS	0.520523	4.23E-37	1.63E-35
CYFIP1	0.047294	2.84E-01	3.46E-01
CYFIP2	-0.39505	1.11E-20	1.38E-19
CYGB	-0.27066	4.26E-10	1.90E-09
CYHR1	-0.21971	4.76E-07	1.50E-06
CYLC1	0.04798	2.77E-01	3.39E-01
CYLC2	-0.02035	6.45E-01	6.99E-01
CYLD	-0.29553	7.71E-12	4.11E-11
CYMP	-0.19362	9.63E-06	2.58E-05
CYP11A1	-0.15011	6.32E-04	1.31E-03
CYP11B1	0.072514	1.00E-01	1.38E-01
CYP11B2	0.025834	5.59E-01	6.20E-01
CYP17A1	-0.28154	7.73E-11	3.71E-10
CYP19A1	0.089232	4.30E-02	6.42E-02
CYP1A1	-0.02792	5.27E-01	5.91E-01
CYP1A2	-0.18226	3.17E-05	7.92E-05
CYP1B1	0.015362	7.28E-01	7.73E-01
CYP20A1	-0.37474	1.29E-18	1.33E-17
CYP21A2	-0.33432	6.50E-15	4.70E-14
CYP24A1	0.177687	5.02E-05	1.22E-04
CYP26A1	0.025682	5.61E-01	6.22E-01
CYP26B1	0.136091	1.97E-03	3.77E-03
CYP26C1	0.032984	4.55E-01	5.21E-01
CYP27A1	-0.31862	1.29E-13	8.28E-13
CYP27B1	0.123653	4.95E-03	8.91E-03
CYP27C1	0.057106	1.96E-01	2.50E-01
CYP2A13	-0.14887	7.01E-04	1.44E-03
CYP2A6	-0.36602	9.01E-18	8.59E-17
CYP2A7	-0.17228	8.51E-05	2.00E-04
CYP2B6	-0.28473	4.62E-11	2.26E-10
CYP2B7P1	-0.57832	2.68E-47	1.70E-45
CYP2C18	-0.13434	2.25E-03	4.27E-03
CYP2C19	-0.05811	1.88E-01	2.41E-01
CYP2C8	-0.15806	3.17E-04	6.87E-04
CYP2C9	-0.06769	1.25E-01	1.68E-01
CYP2D6	-0.17265	8.21E-05	1.94E-04
CYP2D7P1	-0.10981	1.26E-02	2.11E-02
CYP2E1	-0.12174	5.67E-03	1.01E-02

CYP2F1	-0.21825	5.70E-07	1.78E-06
CYP2J2	-0.06567	1.37E-01	1.82E-01
CYP2R1	-0.24866	1.07E-08	4.06E-08
CYP2S1	-0.14091	1.35E-03	2.65E-03
CYP2U1	-0.28371	5.46E-11	2.65E-10
CYP2W1	-0.02614	5.54E-01	6.15E-01
CYP39A1	-0.27482	2.24E-10	1.03E-09
CYP3A43	-0.0717	1.04E-01	1.43E-01
CYP3A4	-0.00255	9.54E-01	9.63E-01
CYP3A5	-0.15088	5.92E-04	1.23E-03
CYP3A7	-0.29352	1.08E-11	5.69E-11
CYP46A1	-0.22859	1.57E-07	5.24E-07
CYP4A11	-0.44715	1.11E-26	2.22E-25
CYP4A22	-0.37972	4.15E-19	4.45E-18
CYP4B1	-0.59975	1.27E-51	8.95E-50
CYP4F11	0.0085	8.47E-01	8.75E-01
CYP4F12	-0.15726	3.40E-04	7.34E-04
CYP4F22	-0.06829	1.22E-01	1.64E-01
CYP4F2	0.113557	9.91E-03	1.68E-02
CYP4F3	0.187254	1.89E-05	4.89E-05
CYP4F8	0.122867	5.24E-03	9.37E-03
CYP4V2	-0.37839	5.63E-19	5.96E-18
CYP4X1	-0.44	8.51E-26	1.60E-24
CYP4Z1	-0.49355	5.64E-33	1.73E-31
CYP4Z2P	-0.57764	3.63E-47	2.29E-45
CYP51A1	0.078122	7.65E-02	1.09E-01
CYP7A1	-0.2287	1.55E-07	5.17E-07
CYP7B1	-0.10684	1.53E-02	2.51E-02
CYP8B1	-0.0237	5.92E-01	6.50E-01
CYR61	-0.08522	5.33E-02	7.81E-02
CYS1	-0.29362	1.06E-11	5.60E-11
CYSLTR1	-0.2632	1.31E-09	5.54E-09
CYSLTR2	-0.0197	6.56E-01	7.09E-01
CYTH1	-0.2211	4.01E-07	1.28E-06
CYTH2	-0.20244	3.64E-06	1.03E-05
CYTH3	-0.23741	4.96E-08	1.75E-07
CYTH4	-0.05516	2.11E-01	2.68E-01
CYTIP	-0.0673	1.27E-01	1.71E-01
CYTL1	-0.02622	5.53E-01	6.14E-01
CYTSA	-0.15376	4.62E-04	9.75E-04
CYTSB	0.222049	3.57E-07	1.15E-06
CYYR1	-0.32606	3.20E-14	2.17E-13
CYorf15A	0.016372	7.11E-01	7.58E-01
CYorf15B	-0.0345	4.35E-01	5.01E-01

D2HGDH	-0.28346	5.68E-11	2.76E-10
D4S234E	-0.2721	3.41E-10	1.54E-09
DAAM1	-0.00611	8.90E-01	9.11E-01
DAAM2	-0.46748	2.54E-29	6.24E-28
DAB1	0.027681	5.31E-01	5.94E-01
DAB2IP	-0.29785	5.20E-12	2.83E-11
DAB2	-0.15445	4.35E-04	9.23E-04
DACH1	-0.2377	4.77E-08	1.68E-07
DACH2	-0.16295	2.04E-04	4.56E-04
DACT1	0.060093	1.73E-01	2.25E-01
DACT2	-0.24432	1.95E-08	7.19E-08
DACT3	-0.26813	6.26E-10	2.74E-09
DAD1L	-0.08595	5.12E-02	7.54E-02
DAD1	0.276797	1.64E-10	7.66E-10
DAG1	0.010106	8.19E-01	8.51E-01
DAGLA	0.031193	4.80E-01	5.45E-01
DAGLB	-0.19225	1.12E-05	2.97E-05
DAK	-0.06741	1.27E-01	1.70E-01
DALRD3	-0.27523	2.10E-10	9.70E-10
DAND5	0.120924	6.00E-03	1.06E-02
DAO	-0.11315	1.02E-02	1.72E-02
DAP3	0.276733	1.66E-10	7.73E-10
DAPK1	-0.44595	1.56E-26	3.11E-25
DAPK2	-0.6201	4.85E-56	3.81E-54
DAPK3	-0.0177	6.89E-01	7.38E-01
DAPL1	0.022791	6.06E-01	6.63E-01
DAPP1	0.045314	3.05E-01	3.68E-01
DAP	-0.07968	7.08E-02	1.01E-01
DARC	-0.39005	3.68E-20	4.37E-19
DARS2	0.413262	1.16E-22	1.68E-21
DARS	0.455643	9.18E-28	2.02E-26
DAXX	0.083307	5.89E-02	8.56E-02
DAZ1	-0.04009	3.64E-01	4.30E-01
DAZ2	0.004179	9.25E-01	9.40E-01
DAZ3	0.010801	8.07E-01	8.40E-01
DAZAP1	0.249889	9.00E-09	3.45E-08
DAZAP2	-0.03265	4.60E-01	5.26E-01
DAZL	0.117139	7.79E-03	1.35E-02
DBC1	-0.12028	6.28E-03	1.11E-02
DBF4B	0.533579	3.11E-39	1.37E-37
DBF4	0.769457	5.98E-102	9.80E-100
DBH	-0.18465	2.48E-05	6.29E-05
DBI	0.055363	2.10E-01	2.66E-01
DBN1	0.277512	1.47E-10	6.87E-10

DBNDD1	0.090798	3.94E-02	5.94E-02
DBNDD2	-0.15739	3.36E-04	7.26E-04
DBNL	-0.01064	8.10E-01	8.42E-01
DBP	-0.3486	3.68E-16	3.01E-15
DBR1	0.275986	1.86E-10	8.66E-10
DBT	-0.17659	5.59E-05	1.35E-04
DBX1	0.126184	4.13E-03	7.52E-03
DBX2	0.018161	6.81E-01	7.31E-01
DCAF10	0.065951	1.35E-01	1.80E-01
DCAF11	-0.24757	1.24E-08	4.69E-08
DCAF12L1	0.093213	3.44E-02	5.25E-02
DCAF12L2	0.090148	4.09E-02	6.14E-02
DCAF12	0.062968	1.54E-01	2.02E-01
DCAF13	0.361605	2.36E-17	2.18E-16
DCAF15	0.049949	2.58E-01	3.19E-01
DCAF16	0.113847	9.72E-03	1.65E-02
DCAF17	0.264582	1.07E-09	4.56E-09
DCAF4L1	-0.05915	1.80E-01	2.33E-01
DCAF4L2	0.168818	1.18E-04	2.73E-04
DCAF4	-0.17644	5.67E-05	1.37E-04
DCAF5	-0.23252	9.44E-08	3.23E-07
DCAF6	-0.34532	7.20E-16	5.73E-15
DCAF7	0.166101	1.53E-04	3.46E-04
DCAF8L1	0.188114	1.73E-05	4.49E-05
DCAF8L2	0.1723	8.49E-05	2.00E-04
DCAF8	-0.42185	1.23E-23	1.94E-22
DCAKD	-0.11197	1.10E-02	1.85E-02
DCBLD1	0.070632	1.09E-01	1.49E-01
DCBLD2	0.226735	1.98E-07	6.56E-07
DCC	-0.09079	3.94E-02	5.94E-02
DCDC1	-0.01688	7.02E-01	7.51E-01
DCDC2B	-0.27843	1.27E-10	5.98E-10
DCDC2	-0.08478	5.45E-02	7.98E-02
DCD	-0.06951	1.15E-01	1.56E-01
DCHS1	-0.09636	2.88E-02	4.46E-02
DCHS2	-0.06474	1.42E-01	1.89E-01
DCI	-0.02759	5.32E-01	5.95E-01
DCK	0.456379	7.37E-28	1.63E-26
DCLK1	0.040072	3.64E-01	4.30E-01
DCLK2	-0.10445	1.77E-02	2.87E-02
DCLK3	0.204124	3.01E-06	8.61E-06
DCLRE1A	0.321515	7.54E-14	4.94E-13
DCLRE1B	0.40592	7.54E-22	1.02E-20
DCLRE1C	0.160363	2.58E-04	5.67E-04

DCN	-0.26229	1.50E-09	6.30E-09
DCP1A	-0.00791	8.58E-01	8.84E-01
DCP1B	-0.3098	6.43E-13	3.86E-12
DCP2	-0.06684	1.30E-01	1.74E-01
DCPS	0.198599	5.59E-06	1.55E-05
DCST1	-0.01968	6.56E-01	7.09E-01
DCST2	-0.14653	8.52E-04	1.73E-03
DCTD	-0.183	2.94E-05	7.36E-05
DCTN1	-0.21553	7.91E-07	2.43E-06
DCTN2	0.223462	2.99E-07	9.70E-07
DCTN3	0.111795	1.11E-02	1.87E-02
DCTN4	-0.21443	9.03E-07	2.75E-06
DCTN5	0.135745	2.02E-03	3.87E-03
DCTN6	-0.00174	9.69E-01	9.76E-01
DCTPP1	0.301612	2.72E-12	1.53E-11
DCT	-0.21865	5.42E-07	1.70E-06
DCUN1D1	0.40417	1.17E-21	1.56E-20
DCUN1D2	-0.15181	5.47E-04	1.14E-03
DCUN1D3	-0.08993	4.13E-02	6.20E-02
DCUN1D4	0.095049	3.10E-02	4.78E-02
DCUN1D5	0.476281	1.61E-30	4.29E-29
DCXR	-0.25501	4.35E-09	1.73E-08
DCX	0.002357	9.57E-01	9.66E-01
DDA1	0.095809	2.97E-02	4.59E-02
DDAH1	-0.37069	3.21E-18	3.19E-17
DDAH2	0.026525	5.48E-01	6.10E-01
DDB1	0.110291	1.23E-02	2.05E-02
DDB2	-0.06562	1.37E-01	1.82E-01
DDC	0.011712	7.91E-01	8.27E-01
DDHD1	-0.00309	9.44E-01	9.56E-01
DDHD2	0.135705	2.03E-03	3.88E-03
DDI1	-0.1329	2.51E-03	4.73E-03
DDI2	0.049128	2.66E-01	3.27E-01
DDIT3	0.30235	2.39E-12	1.35E-11
DDIT4L	-0.07196	1.03E-01	1.41E-01
DDIT4	0.17378	7.36E-05	1.75E-04
DDN	0.368131	5.66E-18	5.48E-17
DDOST	0.229335	1.42E-07	4.79E-07
DDO	-0.11974	6.52E-03	1.15E-02
DDR1	-0.23104	1.14E-07	3.88E-07
DDR2	-0.1252	4.43E-03	8.03E-03
DDRGK1	-0.29779	5.25E-12	2.86E-11
DDTL	0.002071	9.63E-01	9.71E-01
DDT	0.034924	4.29E-01	4.95E-01

DDX10		0.348031	4.13E-16	3.36E-15
DDX11L2		-0.1159	8.47E-03	1.45E-02
DDX11		0.383943	1.56E-19	1.75E-18
DDX12		0.379047	4.84E-19	5.16E-18
DDX17		-0.39082	3.07E-20	3.66E-19
DDX18		0.407611	4.92E-22	6.79E-21
DDX19A		-0.03401	4.41E-01	5.08E-01
DDX19B		-0.16219	2.19E-04	4.86E-04
DDX1		0.365833	9.39E-18	8.95E-17
DDX20		0.143666	1.08E-03	2.15E-03
DDX21		0.335552	5.10E-15	3.73E-14
DDX23		0.393446	1.63E-20	1.99E-19
DDX24		-0.20468	2.82E-06	8.10E-06
DDX25		0.160806	2.48E-04	5.46E-04
DDX26B		-0.09507	3.10E-02	4.77E-02
DDX27		0.2309	1.16E-07	3.95E-07
DDX28		-0.02817	5.24E-01	5.87E-01
DDX31		-0.10407	1.82E-02	2.93E-02
DDX39		0.54836	9.16E-42	4.58E-40
DDX3X		0.053561	2.25E-01	2.83E-01
DDX3Y		0.02573	5.60E-01	6.21E-01
DDX41		0.079956	6.98E-02	1.00E-01
DDX42		-0.15474	4.25E-04	9.01E-04
DDX43		-0.02083	6.37E-01	6.92E-01
DDX46		0.053083	2.29E-01	2.87E-01
DDX47		0.46091	1.89E-28	4.35E-27
DDX49		0.169813	1.08E-04	2.50E-04
DDX4		-0.06165	1.62E-01	2.12E-01
DDX50		0.191268	1.24E-05	3.28E-05
DDX51		0.00382	9.31E-01	9.45E-01
DDX52		0.512059	9.13E-36	3.28E-34
DDX53		0.206747	2.23E-06	6.47E-06
DDX54		0.177031	5.35E-05	1.30E-04
DDX55		0.383036	1.92E-19	2.14E-18
DDX56		0.299426	3.97E-12	2.19E-11
DDX58		0.173732	7.39E-05	1.76E-04
DDX59		0.023486	5.95E-01	6.53E-01
DDX5		-0.20861	1.79E-06	5.28E-06
DDX60L		0.122195	5.49E-03	9.79E-03
DDX60		0.138096	1.68E-03	3.26E-03
DDX6		-0.15533	4.03E-04	8.59E-04
DEAF1		-0.29984	3.70E-12	2.05E-11
	1-Dec	0.01358	7.59E-01	7.99E-01
DECR1		-0.03409	4.40E-01	5.07E-01

DECR2	-0.15644	3.66E-04	7.85E-04
DEDD2	0.023938	5.88E-01	6.46E-01
DEDD	0.085194	5.33E-02	7.82E-02
DEF6	-0.16868	1.20E-04	2.76E-04
DEF8	-0.11034	1.22E-02	2.04E-02
DEFA1B	-0.0246	5.78E-01	6.37E-01
DEFA4	-0.0002	9.96E-01	9.97E-01
DEFA5	0.108426	1.38E-02	2.29E-02
DEFA6	0.086733	4.92E-02	7.26E-02
DEFB103B	0.196416	7.11E-06	1.94E-05
DEFB109P1B	0.056856	1.98E-01	2.52E-01
DEFB115	-0.01113	8.01E-01	8.36E-01
DEFB118	0.065477	1.38E-01	1.83E-01
DEFB119	-0.00787	8.59E-01	8.84E-01
DEFB124	-0.17686	5.44E-05	1.32E-04
DEFB125	0.041127	3.52E-01	4.18E-01
DEFB126	0.056194	2.03E-01	2.58E-01
DEFB131	-0.09597	2.94E-02	4.55E-02
DEFB132	0.061591	1.63E-01	2.13E-01
DEFB1	0.135728	2.02E-03	3.87E-03
DEFB4A	0.155857	3.85E-04	8.23E-04
DEGS1	0.10814	1.41E-02	2.33E-02
DEGS2	-0.22698	1.92E-07	6.37E-07
DEK	0.463068	9.83E-29	2.31E-27
DEM1	0.049879	2.59E-01	3.20E-01
DENND1A	0.250346	8.44E-09	3.25E-08
DENND1B	-0.00631	8.86E-01	9.08E-01
DENND1C	-0.28773	2.83E-11	1.42E-10
DENND2A	-0.25186	6.81E-09	2.65E-08
DENND2C	0.035518	4.21E-01	4.87E-01
DENND2D	-0.14404	1.05E-03	2.09E-03
DENND3	-0.39999	3.30E-21	4.27E-20
DENND4A	-0.08468	5.48E-02	8.01E-02
DENND4B	-0.02703	5.41E-01	6.03E-01
DENND4C	-0.07578	8.58E-02	1.20E-01
DENND5A	-0.08772	4.66E-02	6.92E-02
DENND5B	0.212418	1.15E-06	3.45E-06
DENR	0.629572	3.28E-58	2.73E-56
DEPDC1B	0.856571	1.64E-149	8.42E-147
DEPDC1	0.882634	2.61E-170	3.72E-167
DEPDC4	0.235969	6.00E-08	2.10E-07
DEPDC5	-0.01816	6.81E-01	7.31E-01
DEPDC6	-0.28856	2.47E-11	1.25E-10
DEPDC7	0.126287	4.10E-03	7.46E-03

DERA	0.35587	8.04E-17	7.03E-16
DERL1	0.271086	3.99E-10	1.79E-09
DERL2	0.051267	2.45E-01	3.06E-01
DERL3	-0.08865	4.43E-02	6.61E-02
DES	-0.37341	1.75E-18	1.78E-17
DET1	-0.3295	1.66E-14	1.15E-13
DEXI	-0.52304	1.67E-37	6.54E-36
DFFA	0.24921	9.90E-09	3.78E-08
DFFB	-0.15412	4.48E-04	9.47E-04
DFNA5	0.113529	9.92E-03	1.69E-02
DFNB31	-0.09972	2.36E-02	3.73E-02
DFNB59	-0.17507	6.49E-05	1.55E-04
DGAT1	-0.29137	1.55E-11	8.01E-11
DGAT2L6	0.024147	5.85E-01	6.44E-01
DGAT2	-0.04823	2.75E-01	3.37E-01
DGCR10	-0.10647	1.56E-02	2.56E-02
DGCR11	-0.10409	1.81E-02	2.93E-02
DGCR14	0.029522	5.04E-01	5.68E-01
DGCR2	-0.29014	1.90E-11	9.73E-11
DGCR5	0.02335	5.97E-01	6.55E-01
DGCR6L	-0.09201	3.68E-02	5.58E-02
DGCR6	-0.20256	3.59E-06	1.02E-05
DGCR8	-0.08539	5.28E-02	7.75E-02
DGCR9	-0.04586	2.99E-01	3.62E-01
DGKA	-0.04184	3.43E-01	4.09E-01
DGKB	0.134811	2.17E-03	4.13E-03
DGKD	-0.27629	1.78E-10	8.27E-10
DGKE	0.099309	2.42E-02	3.81E-02
DGKG	0.175741	6.08E-05	1.46E-04
DGKH	0.160962	2.45E-04	5.39E-04
DGKI	0.085471	5.26E-02	7.72E-02
DGKK	0.062523	1.57E-01	2.05E-01
DGKQ	-0.19306	1.02E-05	2.73E-05
DGKZ	0.148851	7.02E-04	1.45E-03
DGUOK	0.322155	6.69E-14	4.41E-13
DHCR24	-0.28966	2.06E-11	1.05E-10
DHCR7	0.262242	1.51E-09	6.35E-09
DHDDS	-0.02002	6.50E-01	7.04E-01
DHDH	-0.14136	1.30E-03	2.56E-03
DHDPSL	-0.08634	5.02E-02	7.40E-02
DHFRL1	-0.25268	6.07E-09	2.38E-08
DHFR	0.44142	5.70E-26	1.09E-24
DHH	-0.17521	6.40E-05	1.53E-04
DHODH	-0.08335	5.87E-02	8.54E-02

DHPS	-0.00877	8.43E-01	8.71E-01
DHRS11	0.035548	4.21E-01	4.87E-01
DHRS12	-0.46419	6.97E-29	1.66E-27
DHRS13	-0.01737	6.94E-01	7.43E-01
DHRS1	-0.35704	6.27E-17	5.54E-16
DHRS2	0.013745	7.56E-01	7.97E-01
DHRS3	-0.32166	7.35E-14	4.82E-13
DHRS4L1	-0.08397	5.69E-02	8.29E-02
DHRS4L2	-0.10049	2.26E-02	3.58E-02
DHRS4	-0.11743	7.64E-03	1.32E-02
DHRS7B	-0.29533	7.97E-12	4.24E-11
DHRS7C	0.012377	7.79E-01	8.17E-01
DHRS7	-0.062	1.60E-01	2.09E-01
DHRS9	-0.01497	7.35E-01	7.79E-01
DHRSX	0.061687	1.62E-01	2.12E-01
DHTKD1	-0.01475	7.38E-01	7.82E-01
DHX15	0.318302	1.37E-13	8.76E-13
DHX16	0.104158	1.81E-02	2.92E-02
DHX29	-0.20186	3.89E-06	1.10E-05
DHX30	0.000477	9.91E-01	9.94E-01
DHX32	-0.10405	1.82E-02	2.94E-02
DHX33	0.232075	1.00E-07	3.42E-07
DHX34	0.094767	3.15E-02	4.85E-02
DHX35	0.154262	4.43E-04	9.36E-04
DHX36	0.483268	1.70E-31	4.80E-30
DHX37	0.410019	2.67E-22	3.76E-21
DHX38	-0.06581	1.36E-01	1.81E-01
DHX40P1	0.024951	5.72E-01	6.32E-01
DHX40	0.032489	4.62E-01	5.28E-01
DHX57	0.383051	1.92E-19	2.14E-18
DHX58	-0.17336	7.66E-05	1.82E-04
DHX8	0.200579	4.49E-06	1.26E-05
DHX9	0.231574	1.07E-07	3.64E-07
DIABLO	0.41425	8.99E-23	1.31E-21
DIAPH1	0.039715	3.68E-01	4.35E-01
DIAPH2	-0.13109	2.88E-03	5.37E-03
DIAPH3	0.800396	4.35E-116	9.45E-114
DICER1	0.050951	2.48E-01	3.09E-01
DIDO1	-0.24032	3.36E-08	1.21E-07
DIMT1L	0.152097	5.33E-04	1.12E-03
DIO1	-0.22084	4.15E-07	1.32E-06
DIO2	0.210596	1.42E-06	4.23E-06
DIO3OS	-0.30436	1.68E-12	9.67E-12
DIO3	-0.25624	3.64E-09	1.46E-08

DIP2A	-0.13453	2.22E-03	4.21E-03
DIP2B	0.304522	1.64E-12	9.42E-12
DIP2C	-0.17715	5.29E-05	1.28E-04
DIRAS1	-0.15385	4.59E-04	9.68E-04
DIRAS2	-0.04568	3.01E-01	3.64E-01
DIRAS3	-0.21948	4.90E-07	1.54E-06
DIRC1	0.224132	2.75E-07	8.96E-07
DIRC2	-0.10375	1.85E-02	2.98E-02
DIRC3	0.074145	9.28E-02	1.29E-01
DIS3L2	-0.17564	6.14E-05	1.48E-04
DIS3L	-0.24417	1.99E-08	7.33E-08
DIS3	0.071676	1.04E-01	1.43E-01
DISC1	-0.1783	4.72E-05	1.15E-04
DISC2	0.075369	8.75E-02	1.22E-01
DISP1	-0.4581	4.41E-28	9.89E-27
DISP2	0.118442	7.13E-03	1.24E-02
DIXDC1	-0.3358	4.86E-15	3.56E-14
DKC1	0.559629	8.84E-44	4.88E-42
DKFZP434H168	0.046451	2.93E-01	3.56E-01
DKFZP434K028	-0.05302	2.30E-01	2.88E-01
DKFZP434L187	0.03222	4.66E-01	5.31E-01
DKFZP586I1420	-0.24028	3.38E-08	1.21E-07
DKFZP686I15217	-0.21056	1.43E-06	4.24E-06
DKFZp434J0226	0.021451	6.27E-01	6.83E-01
DKFZp434L192	0.028376	5.21E-01	5.84E-01
DKFZp566F0947	-0.02333	5.97E-01	6.55E-01
DKFZp686A1627	0.194401	8.86E-06	2.39E-05
DKFZp686O24166	0.160316	2.59E-04	5.69E-04
DKFZp761E198	0.004227	9.24E-01	9.39E-01
DKFZp779M0652	-0.16779	1.30E-04	2.99E-04
DKK1	0.182242	3.17E-05	7.92E-05
DKK2	-0.16513	1.67E-04	3.77E-04
DKK3	-0.25499	4.36E-09	1.73E-08
DKK4	-0.02653	5.48E-01	6.10E-01
DKKL1	-0.07503	8.90E-02	1.24E-01
DLAT	0.261554	1.68E-09	6.98E-09
DLC1	-0.59918	1.67E-51	1.17E-49
DLD	0.3311	1.22E-14	8.57E-14
DLEC1	-0.48295	1.88E-31	5.30E-30
DLEU1	0.131163	2.86E-03	5.35E-03
DLEU2L	0.029799	5.00E-01	5.64E-01
DLEU2	0.359195	3.96E-17	3.59E-16
DLEU7	-0.05438	2.18E-01	2.75E-01
DLG1	0.083817	5.73E-02	8.35E-02

DLG2	-0.08164	6.41E-02	9.26E-02
DLG3	-0.14368	1.08E-03	2.15E-03
DLG4	-0.15819	3.13E-04	6.80E-04
DLG5	0.099127	2.45E-02	3.85E-02
DLGAP1	-0.09553	3.02E-02	4.66E-02
DLGAP2	-0.16922	1.14E-04	2.64E-04
DLGAP3	0.240398	3.32E-08	1.19E-07
DLGAP4	0.182123	3.21E-05	8.01E-05
DLGAP5	0.88266	2.47E-170	3.72E-167
DLK1	-0.04814	2.75E-01	3.37E-01
DLK2	0.138911	1.58E-03	3.07E-03
DLL1	-0.04516	3.06E-01	3.70E-01
DLL3	0.266807	7.65E-10	3.32E-09
DLL4	-0.11464	9.22E-03	1.57E-02
DLST	-0.02302	6.02E-01	6.60E-01
DLX1	0.260038	2.10E-09	8.63E-09
DLX2	0.327291	2.53E-14	1.73E-13
DLX3	-0.37928	4.59E-19	4.90E-18
DLX4	-0.21476	8.68E-07	2.65E-06
DLX5	0.144338	1.02E-03	2.05E-03
DLX6AS	0.187287	1.89E-05	4.87E-05
DLX6	0.237814	4.70E-08	1.66E-07
DMAP1	-0.13394	2.32E-03	4.39E-03
DMBT1	-0.28201	7.18E-11	3.46E-10
DMBX1	0.2549	4.42E-09	1.75E-08
DMC1	0.473763	3.57E-30	9.30E-29
DMD	-0.29109	1.63E-11	8.37E-11
DMGDH	0.015303	7.29E-01	7.74E-01
DMKN	-0.11583	8.51E-03	1.46E-02
DMP1	0.098131	2.60E-02	4.06E-02
DMPK	0.032739	4.58E-01	5.25E-01
DMRT1	0.193978	9.27E-06	2.49E-05
DMRT2	-0.1318	2.73E-03	5.11E-03
DMRT3	0.115554	8.67E-03	1.49E-02
DMRTA1	-0.22157	3.79E-07	1.21E-06
DMRTA2	-0.00443	9.20E-01	9.36E-01
DMRTB1	0.092298	3.63E-02	5.50E-02
DMRTC1B	-0.50659	6.35E-35	2.15E-33
DMRTC1	-0.29102	1.64E-11	8.46E-11
DMRTC2	0.150973	5.87E-04	1.22E-03
DMTF1	-0.1084	1.38E-02	2.29E-02
DMWD	0.093824	3.33E-02	5.09E-02
DMXL1	-0.26731	7.10E-10	3.09E-09
DMXL2	0.090848	3.93E-02	5.93E-02

DNA2	0.604056	1.57E-52	1.13E-50
DNAH10	-0.29578	7.39E-12	3.95E-11
DNAH11	-0.10742	1.47E-02	2.43E-02
DNAH12	-0.26986	4.81E-10	2.14E-09
DNAH14	0.320869	8.51E-14	5.55E-13
DNAH17	-0.06534	1.39E-01	1.84E-01
DNAH1	-0.35198	1.82E-16	1.53E-15
DNAH2	-0.23673	5.43E-08	1.90E-07
DNAH3	-0.16377	1.89E-04	4.24E-04
DNAH5	-0.38314	1.88E-19	2.09E-18
DNAH6	-0.3335	7.64E-15	5.48E-14
DNAH7	-0.35763	5.54E-17	4.94E-16
DNAH8	-0.19381	9.44E-06	2.54E-05
DNAH9	-0.3201	9.82E-14	6.36E-13
DNAI1	-0.22889	1.51E-07	5.06E-07
DNAI2	-0.31607	2.06E-13	1.30E-12
DNAJA1	0.38593	9.77E-20	1.11E-18
DNAJA2	0.032916	4.56E-01	5.22E-01
DNAJA3	0.110377	1.22E-02	2.04E-02
DNAJA4	-0.14812	7.47E-04	1.53E-03
DNAJB11	0.454575	1.26E-27	2.73E-26
DNAJB12	-0.23762	4.82E-08	1.70E-07
DNAJB13	-0.34464	8.27E-16	6.55E-15
DNAJB14	0.079602	7.11E-02	1.02E-01
DNAJB1	0.165189	1.66E-04	3.75E-04
DNAJB2	-0.286	3.76E-11	1.86E-10
DNAJB3	0.149734	6.52E-04	1.35E-03
DNAJB4	0.261575	1.67E-09	6.96E-09
DNAJB5	0.108852	1.35E-02	2.23E-02
DNAJB6	0.234614	7.18E-08	2.49E-07
DNAJB7	-0.06363	1.49E-01	1.97E-01
DNAJB8	0.020134	6.49E-01	7.02E-01
DNAJB9	-0.04162	3.46E-01	4.12E-01
DNAJC10	0.333648	7.41E-15	5.33E-14
DNAJC11	0.209383	1.64E-06	4.84E-06
DNAJC12	0.050347	2.54E-01	3.15E-01
DNAJC13	0.21551	7.94E-07	2.44E-06
DNAJC14	0.332819	8.71E-15	6.22E-14
DNAJC15	0.015902	7.19E-01	7.65E-01
DNAJC16	-0.10161	2.11E-02	3.36E-02
DNAJC17	-0.16055	2.54E-04	5.58E-04
DNAJC18	-0.06761	1.25E-01	1.69E-01
DNAJC19	0.102547	1.99E-02	3.19E-02
DNAJC1	0.131822	2.72E-03	5.10E-03

DNAJC21	0.00173	9.69E-01	9.76E-01
DNAJC22	0.333923	7.02E-15	5.07E-14
DNAJC24	0.079259	7.23E-02	1.03E-01
DNAJC25-GNG10	0.032163	4.66E-01	5.32E-01
DNAJC25	0.06813	1.23E-01	1.65E-01
DNAJC27	-0.30635	1.19E-12	6.91E-12
DNAJC28	-0.32166	7.33E-14	4.82E-13
DNAJC2	0.416242	5.36E-23	8.01E-22
DNAJC30	-0.13497	2.14E-03	4.08E-03
DNAJC3	-0.18472	2.46E-05	6.25E-05
DNAJC4	-0.35911	4.03E-17	3.66E-16
DNAJC5B	-0.09451	3.20E-02	4.91E-02
DNAJC5G	-0.10085	2.21E-02	3.50E-02
DNAJC5	-0.12063	6.13E-03	1.08E-02
DNAJC6	0.237947	4.62E-08	1.63E-07
DNAJC7	0.234431	7.35E-08	2.54E-07
DNAJC8	0.200232	4.66E-06	1.31E-05
DNAJC9	0.684857	1.55E-72	1.70E-70
DNAL1	-0.19652	7.03E-06	1.92E-05
DNAL4	-0.25404	5.00E-09	1.97E-08
DNALI1	-0.37324	1.81E-18	1.84E-17
DNASE1L1	-0.0303	4.93E-01	5.57E-01
DNASE1L2	0.116314	8.24E-03	1.42E-02
DNASE1L3	-0.24903	1.02E-08	3.87E-08
DNASE1	0.077612	7.85E-02	1.11E-01
DNASE2B	-0.28466	4.67E-11	2.29E-10
DNASE2	-0.13072	2.96E-03	5.51E-03
DND1	-0.149	6.93E-04	1.43E-03
DNER	0.107182	1.50E-02	2.46E-02
DNHD1	-0.22442	2.66E-07	8.66E-07
DNLZ	0.013849	7.54E-01	7.95E-01
DNM1L	0.454134	1.44E-27	3.10E-26
DNM1P35	-0.22878	1.53E-07	5.12E-07
DNM1	0.21707	6.57E-07	2.04E-06
DNM2	-0.26528	9.64E-10	4.13E-09
DNM3	-0.16916	1.15E-04	2.65E-04
DNMBP	-0.12236	5.43E-03	9.68E-03
DNMT1	0.451351	3.26E-27	6.85E-26
DNMT3A	0.346884	5.23E-16	4.22E-15
DNMT3B	0.469636	1.30E-29	3.27E-28
DNMT3L	0.015767	7.21E-01	7.67E-01
DNPEP	0.182999	2.94E-05	7.37E-05
DNTTIP1	0.232855	9.04E-08	3.10E-07
DNTTIP2	0.391504	2.60E-20	3.13E-19

DNTT	0.029356	5.06E-01	5.70E-01
DOC2A	0.041006	3.53E-01	4.19E-01
DOC2B	-0.10435	1.78E-02	2.89E-02
DOCK10	-0.13451	2.22E-03	4.21E-03
DOCK11	-0.04098	3.53E-01	4.19E-01
DOCK1	-0.29877	4.44E-12	2.44E-11
DOCK2	-0.14491	9.74E-04	1.96E-03
DOCK3	0.02119	6.31E-01	6.87E-01
DOCK4	-0.20522	2.65E-06	7.64E-06
DOCK5	-0.04961	2.61E-01	3.22E-01
DOCK6	-0.16815	1.26E-04	2.90E-04
DOCK7	0.186716	2.00E-05	5.15E-05
DOCK8	-0.21057	1.43E-06	4.24E-06
DOCK9	-0.37861	5.35E-19	5.68E-18
DOHH	0.020861	6.37E-01	6.91E-01
DOK1	-0.18566	2.23E-05	5.71E-05
DOK2	-0.14016	1.43E-03	2.80E-03
DOK3	-0.01507	7.33E-01	7.77E-01
DOK4	-0.49177	1.03E-32	3.11E-31
DOK5	0.070051	1.12E-01	1.53E-01
DOK6	-0.23237	9.63E-08	3.29E-07
DOK7	-0.19226	1.12E-05	2.97E-05
DOLK	0.006166	8.89E-01	9.10E-01
DOLPP1	0.144378	1.02E-03	2.04E-03
DOM3Z	-0.13485	2.16E-03	4.12E-03
DONSON	0.658606	2.35E-65	2.30E-63
DOPEY1	-0.1627	2.09E-04	4.65E-04
DOPEY2	-0.01696	7.01E-01	7.49E-01
DOT1L	0.10698	1.51E-02	2.49E-02
DPAGT1	-0.03923	3.74E-01	4.41E-01
DPCD	2.44E-05	1.00E+00	1.00E+00
DPCR1	-0.38667	8.22E-20	9.41E-19
DPEP1	-0.13225	2.64E-03	4.95E-03
DPEP2	-0.32791	2.25E-14	1.55E-13
DPEP3	0.016393	7.11E-01	7.58E-01
DPF1	0.355323	9.03E-17	7.86E-16
DPF2	0.05997	1.74E-01	2.26E-01
DPF3	-0.05611	2.04E-01	2.59E-01
DPH1	-0.37066	3.23E-18	3.21E-17
DPH2	0.329643	1.61E-14	1.12E-13
DPH3B	0.071027	1.07E-01	1.47E-01
DPH3	0.154283	4.42E-04	9.35E-04
DPH5	0.126541	4.02E-03	7.34E-03
DPM1	0.357426	5.78E-17	5.15E-16

DPM2	-0.18476	2.45E-05	6.23E-05
DPM3	-0.14537	9.38E-04	1.89E-03
DPP10	-0.15685	3.53E-04	7.59E-04
DPP3	0.237763	4.73E-08	1.67E-07
DPP4	-0.22143	3.85E-07	1.23E-06
DPP6	-0.3002	3.47E-12	1.93E-11
DPP7	-0.30697	1.06E-12	6.23E-12
DPP8	0.121113	5.92E-03	1.05E-02
DPP9	0.0524	2.35E-01	2.94E-01
DPPA2	0.164962	1.70E-04	3.82E-04
DPPA3	0.008181	8.53E-01	8.80E-01
DPPA4	0.040206	3.63E-01	4.29E-01
DPPA5	0.114759	9.15E-03	1.56E-02
DPRXP4	-0.10433	1.79E-02	2.89E-02
DPRX	-0.03843	3.84E-01	4.50E-01
DPT	-0.16737	1.36E-04	3.10E-04
DPY19L1	0.195197	8.12E-06	2.20E-05
DPY19L2P1	0.170554	1.00E-04	2.34E-04
DPY19L2P2	0.01688	7.02E-01	7.51E-01
DPY19L2P4	-0.04363	3.23E-01	3.88E-01
DPY19L2	0.098083	2.60E-02	4.07E-02
DPY19L3	-0.11502	8.99E-03	1.54E-02
DPY19L4	0.145436	9.33E-04	1.88E-03
DPY30	0.303409	1.99E-12	1.14E-11
DPYD	-0.12356	4.99E-03	8.96E-03
DPYSL2	-0.52751	3.13E-38	1.29E-36
DPYSL3	0.042263	3.38E-01	4.04E-01
DPYSL4	0.102689	1.98E-02	3.17E-02
DPYSL5	0.257782	2.91E-09	1.18E-08
DPYS	-0.25906	2.42E-09	9.89E-09
DQX1	0.055548	2.08E-01	2.64E-01
DR1	0.315832	2.16E-13	1.35E-12
DRAM1	-0.44233	4.40E-26	8.52E-25
DRAM2	0.018743	6.71E-01	7.22E-01
DRAP1	0.334468	6.31E-15	4.57E-14
DRD1	-0.30699	1.06E-12	6.21E-12
DRD2	0.002883	9.48E-01	9.59E-01
DRD3	0.064429	1.44E-01	1.91E-01
DRD4	-0.02247	6.11E-01	6.68E-01
DRD5	-0.18028	3.87E-05	9.56E-05
DRG1	0.381711	2.62E-19	2.88E-18
DRG2	0.033638	4.46E-01	5.13E-01
DRGX	-0.0882	4.54E-02	6.76E-02
DRP2	0.434359	4.12E-25	7.32E-24

DSC1	0.248	1.17E-08	4.42E-08
DSC2	0.356457	7.10E-17	6.25E-16
DSC3	0.125819	4.24E-03	7.71E-03
DSCAML1	-0.2101	1.51E-06	4.47E-06
DSCAM	0.120709	6.09E-03	1.08E-02
DSCC1	0.770208	2.88E-102	4.76E-100
DSCR10	0.102134	2.04E-02	3.27E-02
DSCR3	-0.04994	2.58E-01	3.19E-01
DSCR4	0.155242	4.06E-04	8.65E-04
DSCR6	0.264657	1.06E-09	4.51E-09
DSCR8	0.204228	2.97E-06	8.51E-06
DSCR9	0.112856	1.04E-02	1.76E-02
DSEL	-0.2151	8.34E-07	2.55E-06
DSE	0.240626	3.22E-08	1.16E-07
DSG1	0.164526	1.77E-04	3.97E-04
DSG2	0.359956	3.36E-17	3.07E-16
DSG3	0.120442	6.21E-03	1.10E-02
DSG4	0.234478	7.31E-08	2.53E-07
DSN1	0.613166	1.69E-54	1.27E-52
DSPP	-0.00095	9.83E-01	9.87E-01
DSP	0.351634	1.96E-16	1.65E-15
DSTN	-0.23532	6.54E-08	2.28E-07
DSTYK	-0.34381	9.80E-16	7.67E-15
DST	0.047819	2.79E-01	3.41E-01
DTD1	0.269915	4.77E-10	2.12E-09
DTHD1	-0.19839	5.72E-06	1.58E-05
DTL	0.826147	6.60E-130	1.89E-127
DTNA	0.012074	7.85E-01	8.21E-01
DTNBP1	-0.16536	1.64E-04	3.70E-04
DTNB	-0.18632	2.09E-05	5.35E-05
DTWD1	-0.12054	6.17E-03	1.09E-02
DTWD2	-0.13169	2.75E-03	5.15E-03
DTX1	-0.07301	9.79E-02	1.35E-01
DTX2	0.064755	1.42E-01	1.89E-01
DTX3L	0.212724	1.11E-06	3.33E-06
DTX3	-0.14976	6.50E-04	1.35E-03
DTX4	-0.34637	5.82E-16	4.68E-15
DTYMK	0.612805	2.03E-54	1.52E-52
DULLARD	-0.01252	7.77E-01	8.15E-01
DUOX1	-0.46367	8.18E-29	1.93E-27
DUOX2	-0.2804	9.28E-11	4.42E-10
DUOXA1	-0.44491	2.11E-26	4.15E-25
DUOXA2	-0.3005	3.29E-12	1.83E-11
DUPD1	-0.07324	9.69E-02	1.34E-01

DUS1L	0.078134	7.65E-02	1.09E-01
DUS2L	-0.02853	5.18E-01	5.82E-01
DUS3L	0.14941	6.70E-04	1.38E-03
DUS4L	0.465771	4.30E-29	1.04E-27
DUSP10	-0.00965	8.27E-01	8.58E-01
DUSP11	0.3533	1.38E-16	1.18E-15
DUSP12	0.182651	3.05E-05	7.62E-05
DUSP13	0.335921	4.74E-15	3.49E-14
DUSP14	0.417719	3.64E-23	5.54E-22
DUSP15	-0.08009	6.94E-02	9.94E-02
DUSP16	-0.21744	6.28E-07	1.95E-06
DUSP18	-0.21569	7.76E-07	2.39E-06
DUSP19	-0.11651	8.13E-03	1.40E-02
DUSP1	-0.2978	5.24E-12	2.85E-11
DUSP21	0.012356	7.80E-01	8.17E-01
DUSP22	-0.42603	4.00E-24	6.63E-23
DUSP23	-0.01904	6.66E-01	7.18E-01
DUSP26	-0.30622	1.21E-12	7.06E-12
DUSP27	-0.28175	7.48E-11	3.59E-10
DUSP28	-0.16255	2.12E-04	4.71E-04
DUSP2	0.036349	4.10E-01	4.77E-01
DUSP3	-0.07504	8.89E-02	1.24E-01
DUSP4	0.168519	1.22E-04	2.80E-04
DUSP5P	0.268766	5.69E-10	2.50E-09
DUSP5	0.202323	3.69E-06	1.05E-05
DUSP6	-0.09312	3.46E-02	5.28E-02
DUSP7	-0.12103	5.96E-03	1.06E-02
DUSP8	-0.3125	3.96E-13	2.42E-12
DUSP9	0.212878	1.09E-06	3.27E-06
DUT	0.263833	1.20E-09	5.06E-09
DUXA	-0.02128	6.30E-01	6.85E-01
DVL1	-0.01399	7.51E-01	7.93E-01
DVL2	0.128544	3.48E-03	6.40E-03
DVL3	0.288134	2.65E-11	1.34E-10
DVWA	0.033531	4.48E-01	5.14E-01
DYDC1	-0.19459	8.68E-06	2.34E-05
DYDC2	-0.10721	1.49E-02	2.46E-02
DYM	0.105131	1.70E-02	2.76E-02
DYNC1H1	0.036317	4.11E-01	4.77E-01
DYNC1I1	0.116614	8.07E-03	1.39E-02
DYNC1I2	0.233288	8.54E-08	2.94E-07
DYNC1LI1	0.185541	2.26E-05	5.78E-05
DYNC1LI2	-0.28949	2.12E-11	1.08E-10
DYNC2H1	-0.34448	8.55E-16	6.76E-15

DYNC2LI1	-0.19039	1.36E-05	3.58E-05
DYNLL1	0.381635	2.66E-19	2.92E-18
DYNLL2	-0.08452	5.53E-02	8.08E-02
DYNLRB1	0.064639	1.43E-01	1.89E-01
DYNLRB2	-0.34754	4.57E-16	3.71E-15
DYNLT1	0.049288	2.64E-01	3.26E-01
DYNLT3	0.116882	7.93E-03	1.37E-02
DYRK1A	-0.25776	2.92E-09	1.19E-08
DYRK1B	-0.21632	7.20E-07	2.22E-06
DYRK2	0.222725	3.28E-07	1.06E-06
DYRK3	-0.22406	2.78E-07	9.04E-07
DYRK4	-0.09251	3.58E-02	5.45E-02
DYSFIP1	0.197335	6.43E-06	1.77E-05
DYSF	0.069019	1.18E-01	1.59E-01
DYTN	0.023924	5.88E-01	6.46E-01
DYX1C1	-0.21002	1.52E-06	4.51E-06
DZIP1L	-0.08048	6.80E-02	9.77E-02
DZIP1	0.072748	9.91E-02	1.37E-01
DZIP3	-0.05281	2.32E-01	2.90E-01
E2F1	0.651343	1.71E-63	1.62E-61
E2F2	0.737519	1.65E-89	2.31E-87
E2F3	0.472553	5.23E-30	1.34E-28
E2F4	0.202976	3.43E-06	9.75E-06
E2F5	0.092394	3.61E-02	5.47E-02
E2F6	0.445302	1.88E-26	3.71E-25
E2F7	0.684036	2.67E-72	2.90E-70
E2F8	0.728386	2.80E-86	3.71E-84
E4F1	-0.11417	9.51E-03	1.62E-02
EAF1	0.195211	8.11E-06	2.20E-05
EAF2	0.082904	6.01E-02	8.72E-02
EAPP	-0.09035	4.04E-02	6.08E-02
EARS2	-0.19218	1.13E-05	2.99E-05
EBAG9	-0.12983	3.16E-03	5.87E-03
EBF1	-0.15741	3.36E-04	7.25E-04
EBF2	-0.12482	4.56E-03	8.24E-03
EBF3	-0.19897	5.37E-06	1.49E-05
EBF4	-0.39727	6.45E-21	8.14E-20
EBI3	0.009103	8.37E-01	8.66E-01
EBNA1BP2	0.380291	3.63E-19	3.92E-18
EBPL	-0.13316	2.46E-03	4.64E-03
EBP	0.298125	4.96E-12	2.71E-11
ECD	0.137088	1.82E-03	3.51E-03
ECE1	0.105138	1.70E-02	2.76E-02
ECE2	0.494403	4.23E-33	1.31E-31

ECEL1	0.1075	1.47E-02	2.42E-02
ECH1	-0.04885	2.69E-01	3.30E-01
ECHDC1	-0.24872	1.06E-08	4.03E-08
ECHDC2	-0.50253	2.62E-34	8.69E-33
ECHDC3	-0.19405	9.19E-06	2.47E-05
ECHS1	-0.0147	7.39E-01	7.83E-01
ECM1	-0.0315	4.76E-01	5.41E-01
ECM2	-0.26055	1.94E-09	8.04E-09
ECSCR	-0.23384	7.94E-08	2.74E-07
ECSIT	0.016547	7.08E-01	7.56E-01
ECT2L	-0.28097	8.47E-11	4.06E-10
ECT2	0.732666	8.93E-88	1.22E-85
EDA2R	-0.42188	1.21E-23	1.93E-22
EDARADD	0.182788	3.00E-05	7.52E-05
EDAR	-0.15148	5.62E-04	1.17E-03
EDA	-0.30821	8.54E-13	5.06E-12
EDC3	0.196731	6.87E-06	1.88E-05
EDC4	-0.10599	1.61E-02	2.63E-02
EDDM3A	-0.14931	6.76E-04	1.39E-03
EDDM3B	-0.07606	8.46E-02	1.19E-01
EDEM1	-0.31684	1.79E-13	1.13E-12
EDEM2	-0.06529	1.39E-01	1.85E-01
EDEM3	-0.00288	9.48E-01	9.59E-01
EDF1	-0.07379	9.44E-02	1.31E-01
EDIL3	0.02801	5.26E-01	5.89E-01
EDN1	-0.13756	1.75E-03	3.39E-03
EDN2	0.04888	2.68E-01	3.30E-01
EDN3	-0.40568	8.00E-22	1.08E-20
EDNRA	-0.12749	3.76E-03	6.88E-03
EDNRB	-0.42088	1.58E-23	2.48E-22
EEA1	-0.07045	1.10E-01	1.50E-01
EED	0.481391	3.12E-31	8.70E-30
EEF1A1P9	-0.18353	2.78E-05	7.00E-05
EEF1A1	-0.13755	1.76E-03	3.39E-03
EEF1A2	0.015117	7.32E-01	7.77E-01
EEF1B2	0.053392	2.26E-01	2.84E-01
EEF1DP3	-0.08384	5.73E-02	8.34E-02
EEF1D	-0.11758	7.56E-03	1.31E-02
EEF1E1	0.390444	3.35E-20	3.99E-19
EEF1G	0.037239	3.99E-01	4.66E-01
EEF2K	-0.36468	1.21E-17	1.14E-16
EEF2	-0.14851	7.23E-04	1.48E-03
EEFSEC	-0.04132	3.49E-01	4.15E-01
EEPD1	0.140078	1.44E-03	2.82E-03

EFCAB10	-0.25679	3.36E-09	1.35E-08
EFCAB1	-0.26536	9.52E-10	4.08E-09
EFCAB2	-0.22891	1.50E-07	5.04E-07
EFCAB3	0.146453	8.58E-04	1.74E-03
EFCAB4A	-0.2831	6.02E-11	2.92E-10
EFCAB4B	-0.03011	4.95E-01	5.60E-01
EFCAB5	-0.18508	2.37E-05	6.04E-05
EFCAB6	-0.46225	1.26E-28	2.93E-27
EFCAB7	-0.0312	4.80E-01	5.45E-01
EFEMP1	-0.2243	2.70E-07	8.79E-07
EFEMP2	-0.13723	1.80E-03	3.47E-03
EFHA1	-0.11423	9.47E-03	1.61E-02
EFHA2	-0.28095	8.49E-11	4.06E-10
EFHB	-0.30979	6.44E-13	3.87E-12
EFHC1	-0.31039	5.79E-13	3.48E-12
EFHC2	-0.24656	1.43E-08	5.36E-08
EFHD1	-0.1486	7.17E-04	1.47E-03
EFHD2	0.288541	2.48E-11	1.25E-10
EFNA1	-0.28945	2.13E-11	1.08E-10
EFNA2	0.168829	1.18E-04	2.73E-04
EFNA3	0.166079	1.53E-04	3.47E-04
EFNA4	-0.06611	1.34E-01	1.79E-01
EFNA5	-0.01643	7.10E-01	7.57E-01
EFNB1	-0.05358	2.25E-01	2.83E-01
EFNB2	-0.05373	2.24E-01	2.81E-01
EFNB3	-0.11998	6.41E-03	1.13E-02
EFR3A	-0.02933	5.07E-01	5.71E-01
EFR3B	-0.05767	1.91E-01	2.45E-01
EFS	-0.05077	2.50E-01	3.11E-01
EFTUD1	-0.03543	4.22E-01	4.88E-01
EFTUD2	0.542109	1.12E-40	5.36E-39
EGFL6	-0.03263	4.60E-01	5.26E-01
EGFL7	-0.2365	5.60E-08	1.96E-07
EGFL8	-0.04625	2.95E-01	3.58E-01
EGFLAM	0.217992	5.88E-07	1.83E-06
EGFR	-0.04509	3.07E-01	3.71E-01
EGF	0.023518	5.94E-01	6.52E-01
EGLN1	0.069182	1.17E-01	1.58E-01
EGLN2	-0.25146	7.21E-09	2.80E-08
EGLN3	0.408285	4.15E-22	5.77E-21
EGOT	-0.10173	2.09E-02	3.34E-02
EGR1	-0.23898	4.02E-08	1.43E-07
EGR2	-0.08772	4.66E-02	6.92E-02
EGR3	-0.12446	4.68E-03	8.44E-03

EGR4	0.053601	2.25E-01	2.82E-01
EHBP1L1	-0.06399	1.47E-01	1.94E-01
EHBP1	0.376404	8.85E-19	9.23E-18
EHD1	0.014039	7.51E-01	7.92E-01
EHD2	-0.33116	1.20E-14	8.48E-14
EHD3	0.117376	7.67E-03	1.33E-02
EHD4	0.036431	4.09E-01	4.76E-01
EHF	-0.19187	1.16E-05	3.09E-05
EHHADH	0.129503	3.24E-03	6.00E-03
EHMT1	0.056684	1.99E-01	2.54E-01
EHMT2	0.035308	4.24E-01	4.90E-01
EI24	0.088721	4.42E-02	6.59E-02
EID1	-0.20882	1.75E-06	5.16E-06
EID2B	-0.07033	1.11E-01	1.51E-01
EID2	-0.03024	4.93E-01	5.58E-01
EID3	0.210872	1.38E-06	4.10E-06
EIF1AD	0.287647	2.87E-11	1.44E-10
EIF1AX	0.077169	8.02E-02	1.13E-01
EIF1AY	0.030567	4.89E-01	5.54E-01
EIF1B	-0.04739	2.83E-01	3.45E-01
EIF1	0.07953	7.13E-02	1.02E-01
EIF2AK1	0.265078	9.93E-10	4.25E-09
EIF2AK2	0.570385	8.90E-46	5.34E-44
EIF2AK3	0.079721	7.07E-02	1.01E-01
EIF2AK4	0.032057	4.68E-01	5.34E-01
EIF2A	0.169359	1.12E-04	2.60E-04
EIF2B1	0.388411	5.44E-20	6.34E-19
EIF2B2	0.240956	3.08E-08	1.11E-07
EIF2B3	0.236823	5.36E-08	1.88E-07
EIF2B4	0.152201	5.29E-04	1.11E-03
EIF2B5	0.160714	2.50E-04	5.50E-04
EIF2C1	-0.08877	4.41E-02	6.57E-02
EIF2C2	0.411083	2.03E-22	2.89E-21
EIF2C3	0.310594	5.58E-13	3.36E-12
EIF2C4	-0.36798	5.86E-18	5.66E-17
EIF2S1	0.513604	5.25E-36	1.91E-34
EIF2S2	0.53498	1.81E-39	8.15E-38
EIF2S3	0.183609	2.76E-05	6.95E-05
EIF3A	0.122658	5.31E-03	9.49E-03
EIF3B	0.381548	2.72E-19	2.98E-18
EIF3CL	-0.15085	5.93E-04	1.23E-03
EIF3C	0.228016	1.69E-07	5.61E-07
EIF3D	0.131073	2.88E-03	5.38E-03
EIF3E	0.039883	3.66E-01	4.33E-01

EIF3F	-0.07756	7.87E-02	1.11E-01
EIF3G	-0.07579	8.58E-02	1.20E-01
EIF3H	0.065296	1.39E-01	1.85E-01
EIF3IP1	0.053912	2.22E-01	2.80E-01
EIF3I	0.238576	4.25E-08	1.51E-07
EIF3J	0.343546	1.03E-15	8.07E-15
EIF3K	0.10241	2.01E-02	3.22E-02
EIF3L	-0.26602	8.61E-10	3.71E-09
EIF3M	0.308332	8.35E-13	4.95E-12
EIF4A1	0.331243	1.18E-14	8.36E-14
EIF4A2	0.016529	7.08E-01	7.56E-01
EIF4A3	0.527375	3.30E-38	1.36E-36
EIF4B	-0.08932	4.27E-02	6.39E-02
EIF4E1B	0.035943	4.16E-01	4.82E-01
EIF4E2	0.058603	1.84E-01	2.37E-01
EIF4E3	-0.32501	3.91E-14	2.63E-13
EIF4EBP1	0.564128	1.32E-44	7.53E-43
EIF4EBP2	-0.24545	1.67E-08	6.20E-08
EIF4EBP3	-0.40072	2.76E-21	3.60E-20
EIF4ENIF1	-0.21936	4.98E-07	1.57E-06
EIF4E	0.391127	2.85E-20	3.42E-19
EIF4G1	0.271265	3.88E-10	1.74E-09
EIF4G2	0.176228	5.79E-05	1.40E-04
EIF4G3	0.086311	5.03E-02	7.41E-02
EIF4H	0.216992	6.64E-07	2.06E-06
EIF5A2	0.319544	1.09E-13	7.03E-13
EIF5AL1	0.414276	8.93E-23	1.31E-21
EIF5A	0.388978	4.75E-20	5.59E-19
EIF5B	0.287758	2.82E-11	1.42E-10
EIF5	0.112768	1.04E-02	1.77E-02
EIF6	0.220151	4.51E-07	1.43E-06
ELAC1	-0.20752	2.04E-06	5.95E-06
ELAC2	0.006859	8.77E-01	9.00E-01
ELANE	-0.39613	8.51E-21	1.07E-19
ELAVL1	0.348066	4.10E-16	3.34E-15
ELAVL2	0.085542	5.24E-02	7.69E-02
ELAVL3	0.122413	5.41E-03	9.65E-03
ELAVL4	0.11073	1.19E-02	2.00E-02
ELF1	-0.11907	6.83E-03	1.19E-02
ELF2	-0.19411	9.14E-06	2.46E-05
ELF3	-0.12076	6.07E-03	1.07E-02
ELF4	0.111662	1.12E-02	1.89E-02
ELF5	-0.27457	2.33E-10	1.07E-09
ELFN1	0.08085	6.68E-02	9.61E-02

ELFN2	-0.24193	2.70E-08	9.81E-08
ELK1	0.409123	3.35E-22	4.69E-21
ELK3	0.117724	7.49E-03	1.30E-02
ELK4	0.038575	3.82E-01	4.49E-01
ELL2	-0.05175	2.41E-01	3.01E-01
ELL3	-0.22927	1.44E-07	4.83E-07
ELL	-0.12957	3.22E-03	5.97E-03
ELMO1	-0.37354	1.69E-18	1.73E-17
ELMO2	-0.18822	1.71E-05	4.45E-05
ELMO3	-0.25345	5.44E-09	2.14E-08
ELMOD1	-0.1217	5.69E-03	1.01E-02
ELMOD2	0.255774	3.90E-09	1.56E-08
ELMOD3	-0.20837	1.84E-06	5.41E-06
ELN	-0.5095	2.27E-35	7.99E-34
ELOF1	-0.02635	5.51E-01	6.12E-01
ELOVL1	-0.13142	2.81E-03	5.25E-03
ELOVL2	0.161451	2.34E-04	5.18E-04
ELOVL3	0.135206	2.11E-03	4.01E-03
ELOVL4	0.365104	1.10E-17	1.04E-16
ELOVL5	0.165206	1.66E-04	3.74E-04
ELOVL6	0.548595	8.33E-42	4.17E-40
ELOVL7	0.326339	3.04E-14	2.06E-13
ELP2P	-0.03527	4.24E-01	4.90E-01
ELP2	0.073176	9.72E-02	1.34E-01
ELP3	-0.11522	8.87E-03	1.52E-02
ELP4	0.132236	2.64E-03	4.96E-03
ELSPBP1	0.112184	1.08E-02	1.83E-02
ELTD1	-0.03973	3.68E-01	4.34E-01
EMB	-0.24212	2.63E-08	9.56E-08
EMCN	-0.48783	3.81E-32	1.12E-30
EMD	0.053496	2.26E-01	2.83E-01
EME1	0.729894	8.38E-87	1.12E-84
EME2	-0.0709	1.08E-01	1.48E-01
EMG1	0.282657	6.46E-11	3.13E-10
EMID1	-0.15784	3.23E-04	7.00E-04
EMID2	0.054914	2.13E-01	2.70E-01
EMILIN1	-0.00534	9.04E-01	9.22E-01
EMILIN2	0.025038	5.71E-01	6.30E-01
EMILIN3	0.022898	6.04E-01	6.61E-01
EML1	-0.2443	1.96E-08	7.21E-08
EML2	-0.17381	7.34E-05	1.75E-04
EML3	-0.23936	3.82E-08	1.36E-07
EML4	0.12165	5.71E-03	1.01E-02
EML5	-0.24626	1.49E-08	5.58E-08

EML6	-0.02128	6.30E-01	6.85E-01
EMP1	-0.05169	2.42E-01	3.01E-01
EMP2	-0.48859	2.96E-32	8.79E-31
EMP3	0.07146	1.05E-01	1.44E-01
EMR1	0.001204	9.78E-01	9.84E-01
EMR2	0.184265	2.58E-05	6.53E-05
EMR3	-0.16343	1.95E-04	4.37E-04
EMR4P	-0.25044	8.33E-09	3.21E-08
EMX1	0.018828	6.70E-01	7.21E-01
EMX2OS	0.026372	5.50E-01	6.12E-01
EMX2	0.081301	6.52E-02	9.41E-02
EN1	0.121957	5.58E-03	9.94E-03
EN2	-0.08589	5.14E-02	7.56E-02
ENAH	0.143458	1.10E-03	2.19E-03
ENAM	-0.34029	1.99E-15	1.51E-14
ENC1	-0.30739	9.87E-13	5.80E-12
ENDOD1	-0.21965	4.80E-07	1.52E-06
ENDOG	-0.11794	7.38E-03	1.28E-02
ENDOU	-0.26222	1.52E-09	6.36E-09
ENGASE	-0.317	1.74E-13	1.10E-12
ENG	-0.34233	1.32E-15	1.02E-14
ENHO	-0.05458	2.16E-01	2.73E-01
ENKUR	-0.17583	6.03E-05	1.45E-04
ENO1	0.3899	3.82E-20	4.52E-19
ENO2	0.150562	6.08E-04	1.26E-03
ENO3	-0.07474	9.02E-02	1.26E-01
ENOPH1	0.511719	1.03E-35	3.69E-34
ENOSF1	-0.17438	6.95E-05	1.66E-04
ENOX1	0.19678	6.83E-06	1.87E-05
ENOX2	0.22251	3.37E-07	1.09E-06
ENPEP	0.048538	2.72E-01	3.33E-01
ENPP1	0.304343	1.69E-12	9.70E-12
ENPP2	-0.13955	1.50E-03	2.93E-03
ENPP3	-0.16842	1.23E-04	2.83E-04
ENPP4	-0.34764	4.48E-16	3.64E-15
ENPP5	-0.35231	1.70E-16	1.44E-15
ENPP6	-0.22357	2.96E-07	9.58E-07
ENPP7	0.048766	2.69E-01	3.31E-01
ENSA	-0.03986	3.67E-01	4.33E-01
ENTHD1	0.193453	9.81E-06	2.63E-05
ENTPD1	-0.07206	1.02E-01	1.41E-01
ENTPD2	0.068925	1.18E-01	1.60E-01
ENTPD3	-0.41464	8.13E-23	1.19E-21
ENTPD4	-0.03413	4.40E-01	5.06E-01

ENTPD5	0.104577	1.76E-02	2.85E-02
ENTPD6	-0.03765	3.94E-01	4.61E-01
ENTPD7	0.380735	3.28E-19	3.56E-18
ENTPD8	-0.05409	2.20E-01	2.78E-01
ENY2	0.286868	3.26E-11	1.63E-10
EOMES	0.074738	9.02E-02	1.26E-01
EP300	-0.1358	2.01E-03	3.85E-03
EP400NL	0.081734	6.38E-02	9.22E-02
EP400	0.096595	2.84E-02	4.40E-02
EPAS1	-0.31931	1.14E-13	7.32E-13
EPB41L1	-0.34133	1.62E-15	1.23E-14
EPB41L2	-0.0829	6.01E-02	8.73E-02
EPB41L3	0.037651	3.94E-01	4.61E-01
EPB41L4A	-0.45603	8.18E-28	1.80E-26
EPB41L4B	-0.02847	5.19E-01	5.83E-01
EPB41L5	-0.24217	2.61E-08	9.50E-08
EPB41	-0.1779	4.91E-05	1.20E-04
EPB42	-0.04301	3.30E-01	3.95E-01
EPB49	-0.31112	5.08E-13	3.08E-12
EPC1	-0.25074	7.99E-09	3.08E-08
EPC2	-0.19131	1.23E-05	3.27E-05
EPCAM	0.135563	2.05E-03	3.92E-03
EPDR1	-0.38065	3.34E-19	3.63E-18
EPGN	0.07628	8.37E-02	1.18E-01
EPHA10	-0.1884	1.68E-05	4.37E-05
EPHA1	0.039889	3.66E-01	4.33E-01
EPHA2	-0.11162	1.12E-02	1.89E-02
EPHA3	-0.27622	1.80E-10	8.35E-10
EPHA4	-0.40371	1.31E-21	1.75E-20
EPHA5	-0.03581	4.17E-01	4.84E-01
EPHA6	0.279038	1.15E-10	5.45E-10
EPHA7	0.056714	1.99E-01	2.54E-01
EPHA8	0.097032	2.77E-02	4.30E-02
EPHB1	0.039164	3.75E-01	4.41E-01
EPHB2	0.228721	1.54E-07	5.16E-07
EPHB3	-0.06906	1.18E-01	1.59E-01
EPHB4	-0.01601	7.17E-01	7.64E-01
EPHB6	-0.27021	4.56E-10	2.03E-09
EPHX1	-0.42942	1.60E-24	2.72E-23
EPHX2	-0.45475	1.20E-27	2.60E-26
EPHX3	-0.14444	1.01E-03	2.03E-03
EPHX4	0.246399	1.46E-08	5.47E-08
EPM2AIP1	-0.33471	6.02E-15	4.37E-14
EPM2A	-0.22605	2.16E-07	7.13E-07

EPN1	0.051176	2.46E-01	3.06E-01
EPN2	-0.19032	1.37E-05	3.60E-05
EPN3	0.121884	5.61E-03	9.99E-03
EPOR	-0.26653	7.98E-10	3.46E-09
EPO	0.069215	1.17E-01	1.58E-01
EPPK1	-0.12106	5.94E-03	1.05E-02
EPR1	0.816852	1.14E-124	2.89E-122
EPRS	0.180562	3.76E-05	9.30E-05
EPS15L1	0.022762	6.06E-01	6.63E-01
EPS15	-0.15209	5.34E-04	1.12E-03
EPS8L1	-0.17092	9.70E-05	2.27E-04
EPS8L2	-0.30265	2.27E-12	1.29E-11
EPS8L3	0.112758	1.04E-02	1.77E-02
EPS8	-0.22649	2.05E-07	6.76E-07
EPSTI1	0.34139	1.60E-15	1.22E-14
EPT1	0.60153	5.38E-52	3.84E-50
EPX	-0.26413	1.14E-09	4.86E-09
EPYC	0.188029	1.75E-05	4.53E-05
ERAL1	0.355148	9.37E-17	8.14E-16
ERAP1	-0.05665	1.99E-01	2.54E-01
ERAP2	-0.01698	7.01E-01	7.49E-01
ERAS	-0.0745	9.12E-02	1.27E-01
ERBB2IP	-0.10512	1.70E-02	2.76E-02
ERBB2	-0.20005	4.76E-06	1.33E-05
ERBB3	-0.28475	4.61E-11	2.26E-10
ERBB4	-0.44976	5.18E-27	1.07E-25
ERC1	0.034248	4.38E-01	5.05E-01
ERC2	0.023549	5.94E-01	6.52E-01
ERCC1	0.100645	2.24E-02	3.54E-02
ERCC2	0.0848	5.45E-02	7.97E-02
ERCC3	0.207409	2.06E-06	6.01E-06
ERCC4	-0.04437	3.15E-01	3.79E-01
ERCC5	-0.36863	5.07E-18	4.93E-17
ERCC6L	0.80973	7.48E-121	1.80E-118
ERCC6	-0.12792	3.64E-03	6.68E-03
ERCC8	0.250289	8.51E-09	3.27E-08
EREG	0.245081	1.76E-08	6.51E-08
ERF	0.093777	3.34E-02	5.10E-02
ERGIC1	-0.231	1.15E-07	3.90E-07
ERGIC2	0.530465	1.02E-38	4.37E-37
ERGIC3	-0.0366	4.07E-01	4.74E-01
ERG	-0.41169	1.74E-22	2.49E-21
ERH	0.349408	3.11E-16	2.56E-15
ERI1	0.343063	1.14E-15	8.85E-15

ERI2	-0.0171	6.99E-01	7.47E-01
ERI3	0.153911	4.56E-04	9.64E-04
ERICH1	-0.21044	1.45E-06	4.30E-06
ERLEC1	-0.01383	7.54E-01	7.96E-01
ERLIN1	0.428293	2.17E-24	3.66E-23
ERLIN2	0.097863	2.64E-02	4.12E-02
ERMAP	-0.37202	2.38E-18	2.40E-17
ERMN	-0.0732	9.70E-02	1.34E-01
ERMP1	0.262873	1.38E-09	5.80E-09
ERN1	-0.1635	1.94E-04	4.35E-04
ERN2	-0.30742	9.83E-13	5.78E-12
ERO1LB	-0.26207	1.55E-09	6.49E-09
ERO1L	0.564575	1.09E-44	6.27E-43
ERP27	-0.30934	6.98E-13	4.17E-12
ERP29	-0.07414	9.28E-02	1.29E-01
ERP44	0.006899	8.76E-01	8.99E-01
ERRFI1	0.185781	2.21E-05	5.65E-05
ERVFRDE1	-0.41362	1.06E-22	1.54E-21
ESAM	-0.39807	5.30E-21	6.77E-20
ESCO1	0.174804	6.66E-05	1.59E-04
ESCO2	0.842557	5.80E-140	2.27E-137
ESD	-0.09864	2.52E-02	3.95E-02
ESF1	0.248432	1.10E-08	4.18E-08
ESM1	0.234977	6.84E-08	2.38E-07
ESPL1	0.819282	5.22E-126	1.34E-123
ESPNL	-0.05073	2.51E-01	3.11E-01
ESPNP	-0.18558	2.25E-05	5.76E-05
ESPN	0.031843	4.71E-01	5.37E-01
ESR1	0.018147	6.81E-01	7.31E-01
ESR2	-0.08972	4.18E-02	6.27E-02
ESRP1	0.070275	1.11E-01	1.51E-01
ESRP2	-0.36451	1.25E-17	1.18E-16
ESRRA	-0.02253	6.10E-01	6.67E-01
ESRRB	0.008674	8.44E-01	8.73E-01
ESRRG	-0.12548	4.34E-03	7.88E-03
ESX1	0.261325	1.73E-09	7.22E-09
ESYT1	0.179429	4.22E-05	1.04E-04
ESYT2	0.05777	1.91E-01	2.44E-01
ESYT3	-0.50662	6.28E-35	2.14E-33
ETAA1	-0.06182	1.61E-01	2.11E-01
ETF1	0.156126	3.76E-04	8.06E-04
ETFA	0.251481	7.19E-09	2.79E-08
ETFB	-0.10839	1.39E-02	2.29E-02
ETFDH	-0.19197	1.15E-05	3.05E-05

ETHE1	0.15819	3.13E-04	6.80E-04
ETNK1	0.149621	6.58E-04	1.36E-03
ETNK2	0.043542	3.24E-01	3.89E-01
ETS1	-0.07004	1.12E-01	1.53E-01
ETS2	-0.2116	1.26E-06	3.78E-06
ETV1	-0.26168	1.65E-09	6.86E-09
ETV2	-0.09134	3.83E-02	5.78E-02
ETV3L	0.039254	3.74E-01	4.40E-01
ETV3	-0.0528	2.32E-01	2.90E-01
ETV4	-0.14906	6.90E-04	1.42E-03
ETV5	-0.27192	3.51E-10	1.58E-09
ETV6	0.10809	1.41E-02	2.33E-02
ETV7	0.146878	8.28E-04	1.68E-03
EVC2	-0.10682	1.53E-02	2.51E-02
EVC	-0.29499	8.45E-12	4.49E-11
EVI2A	-0.05882	1.83E-01	2.35E-01
EVI2B	-0.16011	2.64E-04	5.79E-04
EVI5L	-0.14229	1.21E-03	2.39E-03
EVI5	-0.23077	1.18E-07	4.01E-07
EVL	-0.22219	3.51E-07	1.13E-06
EVPLL	-0.39475	1.19E-20	1.47E-19
EVPL	-0.30379	1.86E-12	1.07E-11
EVX1	0.194462	8.80E-06	2.37E-05
EVX2	0.08874	4.41E-02	6.58E-02
EWSR1	0.054893	2.14E-01	2.70E-01
EXD1	-0.02577	5.60E-01	6.20E-01
EXD2	-0.00713	8.72E-01	8.96E-01
EXD3	-0.31902	1.20E-13	7.70E-13
EXO1	0.868063	3.93E-158	3.27E-155
EXOC1	0.124102	4.80E-03	8.64E-03
EXOC2	0.174667	6.75E-05	1.61E-04
EXOC3L2	-0.09886	2.49E-02	3.91E-02
EXOC3L	-0.21509	8.34E-07	2.55E-06
EXOC3	-0.01832	6.78E-01	7.29E-01
EXOC4	0.067621	1.25E-01	1.69E-01
EXOC5	0.376434	8.79E-19	9.18E-18
EXOC6B	0.134325	2.25E-03	4.27E-03
EXOC6	0.077495	7.89E-02	1.12E-01
EXOC7	-0.01089	8.05E-01	8.39E-01
EXOC8	-0.3422	1.36E-15	1.04E-14
EXOG	0.046181	2.96E-01	3.59E-01
EXOSC10	0.2698	4.86E-10	2.16E-09
EXOSC1	0.123562	4.98E-03	8.96E-03
EXOSC2	0.457159	5.84E-28	1.30E-26

EXOSC3	0.447408	1.03E-26	2.07E-25
EXOSC4	0.140684	1.37E-03	2.70E-03
EXOSC5	0.163206	1.99E-04	4.45E-04
EXOSC6	0.006631	8.81E-01	9.03E-01
EXOSC7	-0.05821	1.87E-01	2.41E-01
EXOSC8	0.298499	4.65E-12	2.55E-11
EXOSC9	0.422901	9.26E-24	1.49E-22
EXPH5	-0.29368	1.05E-11	5.54E-11
EXT1	0.10151	2.12E-02	3.38E-02
EXT2	0.135797	2.01E-03	3.85E-03
EXTL1	-0.12057	6.15E-03	1.09E-02
EXTL2	0.069747	1.14E-01	1.55E-01
EXTL3	0.054432	2.18E-01	2.74E-01
EYA1	-0.0901	4.10E-02	6.15E-02
EYA2	0.093131	3.46E-02	5.27E-02
EYA3	0.075754	8.59E-02	1.20E-01
EYA4	0.001424	9.74E-01	9.80E-01
EYS	-0.13793	1.70E-03	3.30E-03
EZH1	-0.46943	1.39E-29	3.48E-28
EZH2	0.713366	3.02E-81	3.75E-79
EZR	-0.19473	8.55E-06	2.31E-05
F10	-0.32369	5.01E-14	3.34E-13
F11R	-0.19317	1.01E-05	2.70E-05
F11	-0.42838	2.12E-24	3.58E-23
F12	0.378408	5.60E-19	5.93E-18
F13A1	-0.17475	6.70E-05	1.60E-04
F13B	0.111176	1.16E-02	1.94E-02
F2RL1	-0.06433	1.45E-01	1.92E-01
F2RL2	-0.02269	6.07E-01	6.64E-01
F2RL3	-0.05511	2.12E-01	2.68E-01
F2R	-0.01701	7.00E-01	7.49E-01
F2	0.20856	1.81E-06	5.30E-06
F3	-0.0878	4.64E-02	6.89E-02
F5	0.203046	3.40E-06	9.68E-06
F7	0.128858	3.40E-03	6.27E-03
F8A1	0.085475	5.26E-02	7.71E-02
F8	-0.37841	5.60E-19	5.93E-18
F9	0.004843	9.13E-01	9.30E-01
FA2H	-0.02532	5.66E-01	6.26E-01
FAAH2	-0.18695	1.96E-05	5.04E-05
FAAH	-0.44432	2.49E-26	4.90E-25
FABP12	-0.04175	3.44E-01	4.10E-01
FABP1	0.045967	2.98E-01	3.61E-01
FABP2	0.023224	5.99E-01	6.57E-01

FABP3	-0.15318	4.86E-04	1.02E-03
FABP4	-0.21812	5.79E-07	1.81E-06
FABP5L3	-0.02841	5.20E-01	5.84E-01
FABP5	0.061129	1.66E-01	2.16E-01
FABP6	0.024583	5.78E-01	6.37E-01
FABP7	0.119696	6.54E-03	1.15E-02
FABP9	-0.0056	8.99E-01	9.19E-01
FADD	0.293579	1.07E-11	5.63E-11
FADS1	0.371058	2.95E-18	2.95E-17
FADS2	0.296546	6.49E-12	3.49E-11
FADS3	-0.02216	6.16E-01	6.72E-01
FADS6	-0.13525	2.10E-03	4.00E-03
FAF1	0.357127	6.16E-17	5.46E-16
FAF2	0.026909	5.42E-01	6.05E-01
FAHD1	0.0227	6.07E-01	6.64E-01
FAHD2A	0.128133	3.58E-03	6.58E-03
FAHD2B	-0.0907	3.96E-02	5.97E-02
FAH	-0.16677	1.43E-04	3.27E-04
FAIM2	-0.1999	4.84E-06	1.35E-05
FAIM3	-0.29818	4.92E-12	2.69E-11
FAIM	-0.03197	4.69E-01	5.35E-01
FAM100A	-0.28973	2.04E-11	1.04E-10
FAM100B	0.241174	2.99E-08	1.08E-07
FAM101A	0.309243	7.10E-13	4.24E-12
FAM101B	-0.01911	6.65E-01	7.17E-01
FAM102A	0.105379	1.67E-02	2.72E-02
FAM102B	0.198547	5.62E-06	1.56E-05
FAM103A1	0.207547	2.03E-06	5.93E-06
FAM104A	0.376387	8.89E-19	9.26E-18
FAM104B	-0.1935	9.76E-06	2.61E-05
FAM105A	-0.3382	3.02E-15	2.25E-14
FAM105B	0.259736	2.19E-09	9.00E-09
FAM106A	-0.12694	3.91E-03	7.14E-03
FAM106C	-0.1197	6.54E-03	1.15E-02
FAM107A	-0.41834	3.09E-23	4.73E-22
FAM107B	-0.19527	8.06E-06	2.18E-05
FAM108A1	-0.10909	1.32E-02	2.20E-02
FAM108B1	0.006736	8.79E-01	9.02E-01
FAM108C1	-0.16038	2.58E-04	5.66E-04
FAM109A	-0.01377	7.55E-01	7.96E-01
FAM109B	-0.00992	8.22E-01	8.54E-01
FAM10A4	0.027386	5.35E-01	5.98E-01
FAM110A	-0.10487	1.73E-02	2.80E-02
FAM110B	-0.17364	7.46E-05	1.77E-04

FAM110C	-0.07101	1.07E-01	1.47E-01
FAM111A	0.020622	6.41E-01	6.95E-01
FAM111B	0.761119	1.63E-98	2.59E-96
FAM113A	-0.23058	1.21E-07	4.11E-07
FAM113B	0.057093	1.96E-01	2.50E-01
FAM114A1	0.2543	4.81E-09	1.90E-08
FAM114A2	-0.20611	2.40E-06	6.93E-06
FAM115A	0.03577	4.18E-01	4.84E-01
FAM115C	0.140905	1.35E-03	2.65E-03
FAM116A	0.022699	6.07E-01	6.64E-01
FAM116B	-0.39039	3.40E-20	4.04E-19
FAM117A	-0.51725	1.40E-36	5.30E-35
FAM117B	-0.228	1.69E-07	5.62E-07
FAM118A	-0.14109	1.33E-03	2.61E-03
FAM118B	-0.12706	3.87E-03	7.08E-03
FAM119A	0.298008	5.06E-12	2.76E-11
FAM119B	0.043969	3.19E-01	3.84E-01
FAM120AOS	-0.09246	3.59E-02	5.46E-02
FAM120A	-0.08564	5.21E-02	7.65E-02
FAM120B	-0.08956	4.22E-02	6.32E-02
FAM120C	-0.18254	3.08E-05	7.70E-05
FAM122A	-0.35181	1.89E-16	1.59E-15
FAM122B	0.217275	6.41E-07	1.99E-06
FAM122C	0.025317	5.66E-01	6.26E-01
FAM123A	0.030127	4.95E-01	5.60E-01
FAM123B	-0.04963	2.61E-01	3.22E-01
FAM123C	0.049046	2.67E-01	3.28E-01
FAM124A	0.212701	1.11E-06	3.34E-06
FAM124B	-0.17552	6.21E-05	1.49E-04
FAM125A	-0.03693	4.03E-01	4.69E-01
FAM125B	-0.35599	7.84E-17	6.86E-16
FAM126A	0.120613	6.13E-03	1.08E-02
FAM126B	-0.05826	1.87E-01	2.40E-01
FAM127A	0.028524	5.18E-01	5.82E-01
FAM127B	0.12309	5.15E-03	9.23E-03
FAM127C	0.103909	1.83E-02	2.96E-02
FAM128A	0.277794	1.40E-10	6.59E-10
FAM128B	0.174125	7.12E-05	1.70E-04
FAM129A	-0.25295	5.84E-09	2.29E-08
FAM129B	-0.20803	1.92E-06	5.62E-06
FAM129C	-0.21489	8.55E-07	2.61E-06
FAM131A	0.017902	6.85E-01	7.35E-01
FAM131B	-0.07433	9.20E-02	1.28E-01
FAM131C	0.281704	7.53E-11	3.62E-10

FAM132A	0.105976	1.61E-02	2.64E-02
FAM133A	0.316023	2.08E-13	1.31E-12
FAM133B	0.049288	2.64E-01	3.26E-01
FAM134A	-0.19191	1.16E-05	3.07E-05
FAM134B	-0.15622	3.73E-04	7.99E-04
FAM134C	-0.06472	1.42E-01	1.89E-01
FAM135A	0.248402	1.11E-08	4.20E-08
FAM135B	-0.20292	3.45E-06	9.81E-06
FAM136A	0.586494	6.56E-49	4.36E-47
FAM136B	0.134806	2.17E-03	4.13E-03
FAM138B	-0.17895	4.43E-05	1.08E-04
FAM138D	-0.15262	5.10E-04	1.07E-03
FAM138E	-0.1074	1.48E-02	2.43E-02
FAM138F	-0.27253	3.19E-10	1.45E-09
FAM13AOS	-0.3815	2.75E-19	3.01E-18
FAM13A	-0.26933	5.22E-10	2.31E-09
FAM13B	-0.38054	3.43E-19	3.71E-18
FAM13C	-0.35357	1.31E-16	1.12E-15
FAM149A	-0.37708	7.59E-19	7.95E-18
FAM149B1	-0.15492	4.18E-04	8.88E-04
FAM150A	-0.16898	1.17E-04	2.69E-04
FAM150B	-0.37625	9.17E-19	9.53E-18
FAM151A	-0.10629	1.58E-02	2.59E-02
FAM151B	-0.07005	1.12E-01	1.53E-01
FAM153A	-0.15434	4.40E-04	9.31E-04
FAM153B	-0.15535	4.03E-04	8.58E-04
FAM153C	-0.15268	5.07E-04	1.06E-03
FAM154A	-0.15088	5.92E-04	1.23E-03
FAM154B	-0.30555	1.37E-12	7.91E-12
FAM155A	0.065802	1.36E-01	1.81E-01
FAM155B	-0.06956	1.15E-01	1.56E-01
FAM156A	-0.20882	1.75E-06	5.16E-06
FAM157A	-0.01553	7.25E-01	7.71E-01
FAM157B	-0.05058	2.52E-01	3.13E-01
FAM158A	0.348074	4.10E-16	3.34E-15
FAM159A	-0.08208	6.27E-02	9.07E-02
FAM160A1	-0.06771	1.25E-01	1.68E-01
FAM160A2	-0.3835	1.73E-19	1.93E-18
FAM160B1	-0.1663	1.50E-04	3.41E-04
FAM160B2	-0.2429	2.37E-08	8.65E-08
FAM161A	0.300338	3.39E-12	1.89E-11
FAM161B	-0.26755	6.84E-10	2.98E-09
FAM162A	0.408248	4.18E-22	5.82E-21
FAM162B	-0.35435	1.11E-16	9.55E-16

FAM163A	0.159536	2.78E-04	6.08E-04
FAM163B	0.023766	5.91E-01	6.49E-01
FAM164A	-0.05909	1.81E-01	2.33E-01
FAM164C	-0.30244	2.35E-12	1.33E-11
FAM165B	-0.2587	2.55E-09	1.04E-08
FAM166A	-0.0774	7.93E-02	1.12E-01
FAM166B	-0.22666	2.00E-07	6.62E-07
FAM167A	-0.31101	5.17E-13	3.13E-12
FAM167B	-0.10327	1.91E-02	3.07E-02
FAM168A	0.191247	1.24E-05	3.29E-05
FAM168B	-0.08068	6.73E-02	9.68E-02
FAM169A	0.177299	5.21E-05	1.26E-04
FAM169B	-0.07746	7.91E-02	1.12E-01
FAM170A	-0.00658	8.82E-01	9.04E-01
FAM170B	0.010046	8.20E-01	8.52E-01
FAM171A1	-0.13908	1.56E-03	3.03E-03
FAM171A2	0.308968	7.46E-13	4.44E-12
FAM171B	0.241237	2.97E-08	1.07E-07
FAM172A	-0.23672	5.43E-08	1.90E-07
FAM173A	-0.21027	1.48E-06	4.38E-06
FAM173B	0.062068	1.60E-01	2.09E-01
FAM174A	-0.29619	6.90E-12	3.70E-11
FAM174B	-0.32275	5.98E-14	3.96E-13
FAM175A	-0.12034	6.25E-03	1.10E-02
FAM175B	0.195176	8.14E-06	2.20E-05
FAM176A	-0.14925	6.79E-04	1.40E-03
FAM176B	0.069586	1.15E-01	1.56E-01
FAM177A1	-0.17364	7.46E-05	1.77E-04
FAM177B	-0.02586	5.58E-01	6.19E-01
FAM178A	-0.08691	4.87E-02	7.20E-02
FAM178B	0.006141	8.89E-01	9.10E-01
FAM179A	-0.31125	4.96E-13	3.01E-12
FAM179B	-0.19843	5.69E-06	1.58E-05
FAM180A	-0.29649	6.55E-12	3.52E-11
FAM180B	-0.19089	1.29E-05	3.41E-05
FAM181A	-0.23346	8.35E-08	2.87E-07
FAM181B	-0.03328	4.51E-01	5.17E-01
FAM182A	-0.30964	6.61E-13	3.96E-12
FAM182B	-0.35272	1.56E-16	1.32E-15
FAM183A	-0.25902	2.43E-09	9.94E-09
FAM183B	-0.33236	9.53E-15	6.78E-14
FAM184A	-0.40182	2.10E-21	2.76E-20
FAM184B	-0.18445	2.53E-05	6.41E-05
FAM185A	-0.0746	9.08E-02	1.26E-01

FAM186A	-0.19598	7.46E-06	2.03E-05
FAM186B	-0.12973	3.18E-03	5.91E-03
FAM187B	-0.20446	2.89E-06	8.30E-06
FAM188A	-0.17826	4.74E-05	1.16E-04
FAM188B	-0.26724	7.16E-10	3.12E-09
FAM189A1	-0.11074	1.19E-02	2.00E-02
FAM189A2	-0.52296	1.72E-37	6.72E-36
FAM189B	0.294798	8.73E-12	4.63E-11
FAM18A	-0.26504	9.98E-10	4.27E-09
FAM18B2	-0.06928	1.16E-01	1.58E-01
FAM18B	0.059927	1.75E-01	2.26E-01
FAM190A	-0.0785	7.51E-02	1.07E-01
FAM190B	-0.17629	5.76E-05	1.39E-04
FAM192A	-0.02975	5.01E-01	5.65E-01
FAM193A	-0.16918	1.14E-04	2.64E-04
FAM193B	-0.29389	1.02E-11	5.36E-11
FAM194A	0.089421	4.25E-02	6.36E-02
FAM194B	-0.02321	5.99E-01	6.57E-01
FAM195A	-0.04597	2.98E-01	3.61E-01
FAM195B	0.012624	7.75E-01	8.13E-01
FAM196A	0.048221	2.75E-01	3.37E-01
FAM196B	0.156767	3.56E-04	7.65E-04
FAM197Y2	0.090607	3.98E-02	6.00E-02
FAM198A	-0.28936	2.17E-11	1.10E-10
FAM198B	0.046928	2.88E-01	3.50E-01
FAM199X	0.338303	2.96E-15	2.20E-14
FAM19A1	-0.14405	1.05E-03	2.09E-03
FAM19A2	-0.20222	3.73E-06	1.06E-05
FAM19A3	0.124988	4.50E-03	8.14E-03
FAM19A4	0.128148	3.58E-03	6.58E-03
FAM19A5	-0.158	3.19E-04	6.90E-04
FAM200A	0.019706	6.55E-01	7.08E-01
FAM200B	-0.11776	7.47E-03	1.30E-02
FAM20A	-0.30166	2.70E-12	1.52E-11
FAM20B	0.176375	5.71E-05	1.38E-04
FAM20C	-0.01398	7.52E-01	7.93E-01
FAM21A	-0.05696	1.97E-01	2.51E-01
FAM21B	-0.16997	1.06E-04	2.47E-04
FAM21C	-0.19546	7.90E-06	2.14E-05
FAM22A	-0.4186	2.89E-23	4.43E-22
FAM22D	-0.34928	3.20E-16	2.63E-15
FAM22F	-0.16766	1.32E-04	3.02E-04
FAM22G	-0.06751	1.26E-01	1.69E-01
FAM23A	-0.18382	2.70E-05	6.81E-05

FAM24B	0.30715	1.03E-12	6.04E-12
FAM25A	0.214824	8.62E-07	2.63E-06
FAM25B	0.126798	3.95E-03	7.21E-03
FAM26D	0.107159	1.50E-02	2.46E-02
FAM26E	0.095435	3.04E-02	4.68E-02
FAM26F	0.25115	7.54E-09	2.92E-08
FAM27A	-0.13727	1.79E-03	3.46E-03
FAM27B	-0.06731	1.27E-01	1.71E-01
FAM27C	-0.11022	1.23E-02	2.06E-02
FAM27L	0.070564	1.10E-01	1.50E-01
FAM32A	0.105793	1.63E-02	2.66E-02
FAM35A	0.01524	7.30E-01	7.75E-01
FAM35B2	-0.00052	9.91E-01	9.93E-01
FAM35B	0.011813	7.89E-01	8.25E-01
FAM36A	-0.08418	5.63E-02	8.21E-02
FAM38A	-0.02068	6.40E-01	6.94E-01
FAM38B	-0.26971	4.93E-10	2.19E-09
FAM3A	-0.07574	8.60E-02	1.20E-01
FAM3B	-0.13316	2.46E-03	4.64E-03
FAM3C	0.24329	2.25E-08	8.22E-08
FAM3D	-0.287	3.20E-11	1.59E-10
FAM40A	-0.08621	5.05E-02	7.45E-02
FAM40B	0.429971	1.38E-24	2.35E-23
FAM41AY1	-0.00659	8.81E-01	9.04E-01
FAM41C	-0.21434	9.13E-07	2.77E-06
FAM43A	-0.04168	3.45E-01	4.11E-01
FAM43B	-0.09375	3.34E-02	5.11E-02
FAM45A	0.140767	1.36E-03	2.68E-03
FAM45B	0.14855	7.20E-04	1.48E-03
FAM46A	-0.29333	1.12E-11	5.86E-11
FAM46B	-0.1506	6.06E-04	1.26E-03
FAM46C	-0.32883	1.88E-14	1.30E-13
FAM46D	0.139085	1.56E-03	3.03E-03
FAM47A	0.084136	5.64E-02	8.23E-02
FAM47B	0.01697	7.01E-01	7.49E-01
FAM47C	-0.23034	1.25E-07	4.23E-07
FAM47E	-0.29571	7.48E-12	3.99E-11
FAM48A	-0.15632	3.70E-04	7.93E-04
FAM48B1	-0.01088	8.05E-01	8.39E-01
FAM48B2	0.059201	1.80E-01	2.32E-01
FAM49A	-0.1058	1.63E-02	2.66E-02
FAM49B	0.337361	3.57E-15	2.64E-14
FAM50A	0.157647	3.29E-04	7.11E-04
FAM50B	-0.11482	9.11E-03	1.56E-02

FAM53A	-0.186	2.16E-05	5.52E-05
FAM53B	-0.26135	1.73E-09	7.19E-09
FAM53C	0.035758	4.18E-01	4.84E-01
FAM54A	0.804434	4.06E-118	9.23E-116
FAM54B	-0.27984	1.01E-10	4.82E-10
FAM55A	-0.04738	2.83E-01	3.45E-01
FAM55B	0.061212	1.65E-01	2.16E-01
FAM55C	0.121394	5.81E-03	1.03E-02
FAM55D	-0.18127	3.50E-05	8.70E-05
FAM57A	0.065472	1.38E-01	1.84E-01
FAM57B	0.31874	1.26E-13	8.10E-13
FAM58A	0.168993	1.16E-04	2.69E-04
FAM58B	0.083287	5.89E-02	8.57E-02
FAM59A	-0.14395	1.05E-03	2.11E-03
FAM5B	0.019098	6.65E-01	7.17E-01
FAM5C	-0.25216	6.54E-09	2.55E-08
FAM60A	0.398157	5.19E-21	6.63E-20
FAM63A	-0.33878	2.69E-15	2.01E-14
FAM63B	-0.00725	8.70E-01	8.94E-01
FAM64A	0.828646	2.28E-131	6.81E-129
FAM65A	-0.10178	2.09E-02	3.33E-02
FAM65B	-0.33877	2.70E-15	2.02E-14
FAM65C	0.232032	1.01E-07	3.43E-07
FAM66A	-0.26221	1.52E-09	6.37E-09
FAM66C	-0.03674	4.05E-01	4.72E-01
FAM66D	-0.21002	1.52E-06	4.51E-06
FAM66E	-0.09162	3.77E-02	5.70E-02
FAM69A	0.103993	1.82E-02	2.95E-02
FAM69B	-0.08473	5.47E-02	8.00E-02
FAM69C	-0.01482	7.37E-01	7.81E-01
FAM70A	-0.06276	1.55E-01	2.04E-01
FAM70B	-0.05369	2.24E-01	2.82E-01
FAM71A	-0.10929	1.31E-02	2.17E-02
FAM71B	-0.02665	5.46E-01	6.08E-01
FAM71C	0.008139	8.54E-01	8.81E-01
FAM71D	0.260431	1.98E-09	8.17E-09
FAM71E1	-0.1162	8.30E-03	1.43E-02
FAM71E2	0.143147	1.12E-03	2.24E-03
FAM71F1	0.108355	1.39E-02	2.30E-02
FAM71F2	-0.09015	4.09E-02	6.14E-02
FAM72A	0.773697	9.40E-104	1.59E-101
FAM72B	0.820523	1.06E-126	2.79E-124
FAM72D	0.800418	4.24E-116	9.31E-114
FAM73A	0.088362	4.50E-02	6.71E-02

FAM73B	-0.17095	9.67E-05	2.26E-04
FAM74A1	-0.16333	1.97E-04	4.41E-04
FAM74A3	-0.15206	5.35E-04	1.12E-03
FAM74A4	-0.17507	6.49E-05	1.55E-04
FAM75A2	0.104338	1.79E-02	2.89E-02
FAM75A3	0.100477	2.26E-02	3.58E-02
FAM75A5	0.113243	1.01E-02	1.71E-02
FAM75A6	0.031678	4.73E-01	5.39E-01
FAM75C1	0.018118	6.82E-01	7.32E-01
FAM76A	-0.27264	3.14E-10	1.43E-09
FAM76B	0.038938	3.78E-01	4.44E-01
FAM78A	-0.15048	6.12E-04	1.27E-03
FAM78B	-0.13396	2.32E-03	4.38E-03
FAM7A2	0.072369	1.01E-01	1.39E-01
FAM7A3	0.134694	2.19E-03	4.16E-03
FAM81A	0.198323	5.76E-06	1.59E-05
FAM81B	-0.30185	2.61E-12	1.47E-11
FAM82A1	-0.51338	5.69E-36	2.06E-34
FAM82A2	-0.25159	7.08E-09	2.75E-08
FAM82B	-0.13372	2.36E-03	4.46E-03
FAM83A	0.270764	4.19E-10	1.88E-09
FAM83B	0.219739	4.75E-07	1.50E-06
FAM83C	0.173984	7.22E-05	1.72E-04
FAM83D	0.731249	2.81E-87	3.83E-85
FAM83E	-0.38139	2.82E-19	3.07E-18
FAM83F	0.263342	1.29E-09	5.43E-09
FAM83G	0.176476	5.65E-05	1.37E-04
FAM83H	-0.03821	3.87E-01	4.53E-01
FAM84A	-0.05648	2.01E-01	2.56E-01
FAM84B	-0.07443	9.15E-02	1.27E-01
FAM86A	-0.07442	9.16E-02	1.27E-01
FAM86B1	-0.25821	2.74E-09	1.11E-08
FAM86B2	-0.16924	1.14E-04	2.63E-04
FAM86C	0.063556	1.50E-01	1.97E-01
FAM86D	-0.08602	5.10E-02	7.51E-02
FAM89A	0.02904	5.11E-01	5.75E-01
FAM89B	-0.03381	4.44E-01	5.10E-01
FAM8A1	-0.37396	1.54E-18	1.58E-17
FAM90A1	0.07579	8.58E-02	1.20E-01
FAM90A7	-0.00427	9.23E-01	9.38E-01
FAM91A1	0.333967	6.96E-15	5.03E-14
FAM92A1	0.190642	1.33E-05	3.49E-05
FAM92A3	0.079812	7.03E-02	1.01E-01
FAM92B	-0.23407	7.71E-08	2.66E-07

FAM95B1	-0.27208	3.42E-10	1.55E-09
FAM96A	0.260918	1.84E-09	7.64E-09
FAM96B	0.035164	4.26E-01	4.92E-01
FAM98A	0.392896	1.86E-20	2.26E-19
FAM98B	0.203442	3.25E-06	9.27E-06
FAM98C	-0.15029	6.22E-04	1.29E-03
FAM99A	0.012253	7.81E-01	8.19E-01
FAM99B	0.012441	7.78E-01	8.16E-01
FAM9A	0.125821	4.24E-03	7.70E-03
FAM9B	0.133428	2.41E-03	4.55E-03
FAM9C	0.094889	3.13E-02	4.81E-02
FANCA	0.585866	8.76E-49	5.78E-47
FANCB	0.745658	1.68E-92	2.42E-90
FANCC	0.35665	6.82E-17	6.00E-16
FANCD2	0.69054	3.43E-74	3.85E-72
FANCE	0.174708	6.73E-05	1.61E-04
FANCF	0.179949	4.00E-05	9.86E-05
FANCG	0.636268	8.63E-60	7.34E-58
FANCI	0.823758	1.57E-128	4.30E-126
FANCL	0.412037	1.59E-22	2.29E-21
FANCM	0.481234	3.29E-31	9.11E-30
FANK1	-0.37643	8.80E-19	9.18E-18
FAP	0.227344	1.84E-07	6.09E-07
FAR1	0.100041	2.32E-02	3.66E-02
FAR2	0.047751	2.79E-01	3.41E-01
FARP1	-0.15757	3.31E-04	7.15E-04
FARP2	-0.20655	2.28E-06	6.60E-06
FARS2	-0.16838	1.23E-04	2.84E-04
FARSA	0.230495	1.23E-07	4.15E-07
FARSB	0.538399	4.80E-40	2.25E-38
FASLG	0.12207	5.54E-03	9.87E-03
FASN	-0.05789	1.90E-01	2.43E-01
FASTKD1	0.301278	2.88E-12	1.62E-11
FASTKD2	0.207412	2.06E-06	6.01E-06
FASTKD3	0.095256	3.07E-02	4.73E-02
FASTKD5	-0.15035	6.19E-04	1.28E-03
FASTK	0.030203	4.94E-01	5.59E-01
FAS	-0.05796	1.89E-01	2.43E-01
FAT1	0.089078	4.33E-02	6.47E-02
FAT2	0.002277	9.59E-01	9.68E-01
FAT3	-0.20227	3.71E-06	1.05E-05
FAT4	-0.40377	1.29E-21	1.72E-20
FATE1	0.259471	2.28E-09	9.33E-09
FAU	0.023724	5.91E-01	6.49E-01

FBF1	-0.00513	9.08E-01	9.26E-01
FBLIM1	0.016044	7.16E-01	7.63E-01
FBLL1	0.055034	2.12E-01	2.69E-01
FBLN1	-0.15404	4.51E-04	9.53E-04
FBLN2	-0.06152	1.63E-01	2.13E-01
FBLN5	-0.48783	3.80E-32	1.12E-30
FBLN7	0.243703	2.12E-08	7.78E-08
FBL	0.37655	8.56E-19	8.95E-18
FBN1	-0.00986	8.23E-01	8.54E-01
FBN2	0.220903	4.11E-07	1.31E-06
FBN3	-0.06163	1.63E-01	2.12E-01
FBP1	-0.44222	4.53E-26	8.77E-25
FBP2	-0.16842	1.23E-04	2.83E-04
FBRSL1	0.00206	9.63E-01	9.71E-01
FBRS	-0.15873	2.99E-04	6.50E-04
FBXL12	-0.11636	8.21E-03	1.41E-02
FBXL13	0.127642	3.71E-03	6.81E-03
FBXL14	0.107891	1.43E-02	2.36E-02
FBXL15	-0.26941	5.15E-10	2.28E-09
FBXL16	-0.22293	3.20E-07	1.03E-06
FBXL17	-0.23904	3.99E-08	1.42E-07
FBXL18	0.143272	1.11E-03	2.22E-03
FBXL19	0.13981	1.47E-03	2.88E-03
FBXL20	0.067689	1.25E-01	1.68E-01
FBXL21	0.194931	8.36E-06	2.26E-05
FBXL22	-0.11459	9.25E-03	1.58E-02
FBXL2	-0.05205	2.38E-01	2.98E-01
FBXL3	-0.25527	4.19E-09	1.67E-08
FBXL4	0.13242	2.60E-03	4.89E-03
FBXL5	-0.15088	5.92E-04	1.23E-03
FBXL6	-0.07655	8.26E-02	1.16E-01
FBXL7	-0.21303	1.07E-06	3.22E-06
FBXL8	-0.41955	2.25E-23	3.49E-22
FBXO10	0.141504	1.28E-03	2.54E-03
FBXO11	0.099522	2.39E-02	3.77E-02
FBXO15	-0.2925	1.29E-11	6.69E-11
FBXO16	-0.08273	6.07E-02	8.80E-02
FBXO17	0.113859	9.71E-03	1.65E-02
FBXO18	0.006945	8.75E-01	8.99E-01
FBXO21	0.042781	3.33E-01	3.98E-01
FBXO22OS	0.287933	2.74E-11	1.38E-10
FBXO22	0.189379	1.52E-05	3.96E-05
FBXO24	-0.21127	1.31E-06	3.92E-06
FBXO25	-0.28151	7.77E-11	3.73E-10

FBXO27	0.050768	2.50E-01	3.11E-01
FBXO28	0.073343	9.64E-02	1.33E-01
FBXO2	-0.22693	1.93E-07	6.40E-07
FBXO30	0.354533	1.07E-16	9.20E-16
FBXO31	-0.35938	3.81E-17	3.46E-16
FBXO32	0.306884	1.08E-12	6.31E-12
FBXO33	0.002871	9.48E-01	9.59E-01
FBXO34	-0.2153	8.14E-07	2.50E-06
FBXO36	-0.0172	6.97E-01	7.46E-01
FBXO38	-0.41137	1.89E-22	2.69E-21
FBXO39	0.03838	3.85E-01	4.51E-01
FBXO3	-0.26377	1.21E-09	5.11E-09
FBXO40	-0.01323	7.64E-01	8.04E-01
FBXO41	0.191525	1.21E-05	3.19E-05
FBXO42	-0.1563	3.70E-04	7.94E-04
FBXO43	0.552626	1.61E-42	8.47E-41
FBXO44	-0.28562	4.00E-11	1.97E-10
FBXO45	0.63783	3.64E-60	3.17E-58
FBXO46	0.040311	3.61E-01	4.27E-01
FBXO47	0.16145	2.34E-04	5.18E-04
FBXO48	0.116066	8.38E-03	1.44E-02
FBXO4	-0.06671	1.31E-01	1.75E-01
FBXO5	0.736903	2.76E-89	3.83E-87
FBXO6	0.064316	1.45E-01	1.92E-01
FBXO7	-0.17575	6.08E-05	1.46E-04
FBXO8	-0.18389	2.68E-05	6.77E-05
FBXO9	-0.16914	1.15E-04	2.65E-04
FBXW10	-0.03483	4.30E-01	4.97E-01
FBXW11	-0.21004	1.52E-06	4.50E-06
FBXW12	0.081908	6.33E-02	9.15E-02
FBXW2	-0.15588	3.84E-04	8.22E-04
FBXW4	-0.55741	2.24E-43	1.22E-41
FBXW5	-0.05935	1.79E-01	2.31E-01
FBXW7	-0.04107	3.52E-01	4.18E-01
FBXW8	0.045138	3.07E-01	3.71E-01
FBXW9	-0.04765	2.80E-01	3.42E-01
FCAMR	-0.32909	1.79E-14	1.24E-13
FCAR	0.119959	6.42E-03	1.13E-02
FCER1A	-0.48644	6.02E-32	1.74E-30
FCER1G	0.102321	2.02E-02	3.23E-02
FCER2	-0.28387	5.32E-11	2.59E-10
FCF1	0.139442	1.51E-03	2.95E-03
FCGBP	-0.40745	5.12E-22	7.05E-21
FCGR1A	0.118244	7.22E-03	1.26E-02

FCGR1B	0.118462	7.12E-03	1.24E-02
FCGR1C	0.105062	1.71E-02	2.77E-02
FCGR2A	-0.052	2.39E-01	2.98E-01
FCGR2B	0.024126	5.85E-01	6.44E-01
FCGR2C	0.006903	8.76E-01	8.99E-01
FCGR3A	0.172662	8.20E-05	1.93E-04
FCGR3B	0.089206	4.30E-02	6.43E-02
FCGRT	-0.51142	1.15E-35	4.11E-34
FCHO1	0.091227	3.85E-02	5.81E-02
FCHO2	-0.34401	9.40E-16	7.38E-15
FCHSD1	-0.18124	3.51E-05	8.72E-05
FCHSD2	-0.26562	9.15E-10	3.93E-09
FCN1	-0.19844	5.69E-06	1.57E-05
FCN2	-0.02919	5.09E-01	5.72E-01
FCN3	-0.18765	1.82E-05	4.70E-05
FCRL1	-0.24322	2.27E-08	8.29E-08
FCRL2	-0.09619	2.91E-02	4.50E-02
FCRL3	-0.1255	4.34E-03	7.87E-03
FCRL4	-0.02583	5.59E-01	6.20E-01
FCRL5	0.006192	8.89E-01	9.10E-01
FCRL6	-0.16807	1.27E-04	2.92E-04
FCRLA	-0.11687	7.93E-03	1.37E-02
FCRLB	0.120103	6.36E-03	1.12E-02
FDFT1	-0.09003	4.11E-02	6.17E-02
FDPSL2A	-0.06032	1.72E-01	2.23E-01
FDPS	0.260511	1.96E-09	8.08E-09
FDX1L	-0.07146	1.05E-01	1.44E-01
FDX1	-0.08262	6.10E-02	8.84E-02
FDXACB1	-0.09243	3.60E-02	5.47E-02
FDXR	-0.16167	2.29E-04	5.08E-04
FECH	0.06856	1.20E-01	1.62E-01
FEM1A	-0.16231	2.17E-04	4.81E-04
FEM1B	0.076101	8.45E-02	1.18E-01
FEM1C	-0.28468	4.66E-11	2.28E-10
FEN1	0.770883	1.49E-102	2.49E-100
FER1L4	0.224134	2.75E-07	8.96E-07
FER1L5	-0.20763	2.01E-06	5.87E-06
FER1L6	0.021083	6.33E-01	6.88E-01
FERD3L	0.029371	5.06E-01	5.70E-01
FERMT1	0.072455	1.01E-01	1.38E-01
FERMT2	0.014241	7.47E-01	7.90E-01
FERMT3	-0.04964	2.61E-01	3.22E-01
FER	0.108062	1.41E-02	2.34E-02
FES	-0.21481	8.63E-07	2.64E-06

FETUB	0.16054	2.54E-04	5.58E-04
FEV	0.13285	2.52E-03	4.74E-03
FEZ1	-0.24149	2.87E-08	1.04E-07
FEZ2	0.103518	1.88E-02	3.02E-02
FEZF1	-0.15968	2.75E-04	6.01E-04
FEZF2	0.041675	3.45E-01	4.11E-01
FFAR1	-0.07016	1.12E-01	1.52E-01
FFAR2	0.152538	5.14E-04	1.08E-03
FFAR3	0.131195	2.85E-03	5.33E-03
FGA	0.113718	9.80E-03	1.67E-02
FGB	0.221974	3.60E-07	1.16E-06
FGD1	0.301874	2.60E-12	1.46E-11
FGD2	-0.19815	5.87E-06	1.62E-05
FGD3	-0.24231	2.56E-08	9.32E-08
FGD4	-0.19718	6.54E-06	1.79E-05
FGD5	-0.27509	2.14E-10	9.90E-10
FGD6	-0.0597	1.76E-01	2.28E-01
FGF10	-0.23534	6.52E-08	2.27E-07
FGF11	0.214745	8.70E-07	2.65E-06
FGF12	0.274673	2.29E-10	1.05E-09
FGF13	-0.11717	7.77E-03	1.34E-02
FGF14	-0.47142	7.45E-30	1.89E-28
FGF16	0.030514	4.90E-01	5.54E-01
FGF17	-0.14641	8.60E-04	1.75E-03
FGF18	-0.23455	7.24E-08	2.51E-07
FGF19	0.099661	2.37E-02	3.74E-02
FGF1	-0.13346	2.41E-03	4.54E-03
FGF20	-0.03746	3.96E-01	4.63E-01
FGF21	0.235282	6.57E-08	2.29E-07
FGF22	-0.18549	2.28E-05	5.80E-05
FGF23	0.163	2.03E-04	4.54E-04
FGF2	-0.14337	1.10E-03	2.20E-03
FGF3	0.048357	2.73E-01	3.35E-01
FGF4	0.0416	3.46E-01	4.12E-01
FGF5	0.360483	3.00E-17	2.75E-16
FGF7	-0.14743	7.91E-04	1.61E-03
FGF8	0.083954	5.69E-02	8.30E-02
FGF9	-0.28573	3.93E-11	1.94E-10
FGFBP1	0.114689	9.19E-03	1.57E-02
FGFBP2	-0.24746	1.26E-08	4.76E-08
FGFBP3	-0.09003	4.11E-02	6.17E-02
FGFR1OP2	0.313113	3.54E-13	2.18E-12
FGFR1OP	0.387889	6.15E-20	7.15E-19
FGFR1	-0.0442	3.17E-01	3.81E-01

FGFR2	-0.4456	1.73E-26	3.42E-25
FGFR3	-0.40551	8.35E-22	1.13E-20
FGFR4	-0.17207	8.68E-05	2.04E-04
FGFRL1	-0.10685	1.53E-02	2.51E-02
FGGY	-0.32822	2.12E-14	1.46E-13
FGG	0.069439	1.16E-01	1.57E-01
FGL1	0.081003	6.62E-02	9.54E-02
FGL2	-0.11477	9.14E-03	1.56E-02
FGR	-0.13813	1.68E-03	3.25E-03
FHAD1	-0.13695	1.84E-03	3.55E-03
FHDC1	-0.24869	1.06E-08	4.04E-08
FHIT	-0.34168	1.51E-15	1.16E-14
FHL1	-0.44251	4.18E-26	8.10E-25
FHL2	0.219861	4.68E-07	1.48E-06
FHL3	0.101535	2.12E-02	3.37E-02
FHL5	-0.41923	2.45E-23	3.78E-22
FHOD1	-0.31746	1.60E-13	1.02E-12
FHOD3	0.190695	1.32E-05	3.47E-05
FH	0.195256	8.07E-06	2.19E-05
FIBCD1	0.103268	1.91E-02	3.07E-02
FIBIN	-0.10649	1.56E-02	2.56E-02
FIBP	0.23903	4.00E-08	1.42E-07
FICD	-0.06164	1.63E-01	2.12E-01
FIG4	-0.10255	1.99E-02	3.19E-02
FIGF	-0.5203	4.58E-37	1.76E-35
FIGLA	-0.14448	1.01E-03	2.03E-03
FIGNL1	0.530566	9.85E-39	4.22E-37
FIGNL2	-0.14584	9.02E-04	1.82E-03
FIGN	0.279706	1.04E-10	4.92E-10
FILIP1L	-0.18096	3.61E-05	8.96E-05
FILIP1	-0.30974	6.50E-13	3.89E-12
FIP1L1	0.349343	3.15E-16	2.60E-15
FIS1	-0.05434	2.18E-01	2.75E-01
FITM1	-0.29213	1.37E-11	7.10E-11
FITM2	-0.06688	1.30E-01	1.74E-01
FIZ1	-0.11202	1.10E-02	1.85E-02
FJX1	0.268413	6.00E-10	2.64E-09
FKBP10	0.324397	4.39E-14	2.94E-13
FKBP11	-0.04872	2.70E-01	3.32E-01
FKBP14	0.241838	2.74E-08	9.92E-08
FKBP15	-0.11748	7.61E-03	1.32E-02
FKBP1AP1	0.003095	9.44E-01	9.56E-01
FKBP1A	0.108296	1.39E-02	2.31E-02
FKBP1B	0.044419	3.14E-01	3.79E-01

FKBP2	-0.20806	1.91E-06	5.61E-06
FKBP3	0.344016	9.40E-16	7.38E-15
FKBP4	0.449045	6.38E-27	1.30E-25
FKBP5	0.099112	2.45E-02	3.85E-02
FKBP6	-0.20611	2.40E-06	6.93E-06
FKBP7	-0.1398	1.47E-03	2.88E-03
FKBP8	-0.15294	4.96E-04	1.04E-03
FKBP9L	0.195029	8.27E-06	2.24E-05
FKBP9	0.142824	1.15E-03	2.29E-03
FKBPL	0.19725	6.49E-06	1.78E-05
FKRP	-0.04132	3.49E-01	4.15E-01
FKSG29	-0.00644	8.84E-01	9.06E-01
FKSG83	-0.12368	4.94E-03	8.89E-03
FKTN	0.155127	4.11E-04	8.73E-04
FLAD1	0.314811	2.60E-13	1.62E-12
FLCN	-0.08636	5.02E-02	7.40E-02
FLG2	-0.07282	9.88E-02	1.36E-01
FLG	-0.03981	3.67E-01	4.34E-01
FLI1	-0.27479	2.25E-10	1.04E-09
FLII	-0.07782	7.77E-02	1.10E-01
FLJ10038	-0.38199	2.45E-19	2.71E-18
FLJ10213	-0.15878	2.97E-04	6.47E-04
FLJ10357	-0.25333	5.53E-09	2.18E-08
FLJ10661	-0.27419	2.47E-10	1.13E-09
FLJ11235	-0.39513	1.08E-20	1.35E-19
FLJ12825	-0.06527	1.39E-01	1.85E-01
FLJ13197	-0.33234	9.57E-15	6.80E-14
FLJ13224	0.056787	1.98E-01	2.53E-01
FLJ14107	-0.11785	7.42E-03	1.29E-02
FLJ16779	0.119791	6.50E-03	1.14E-02
FLJ22536	0.295921	7.22E-12	3.87E-11
FLJ23867	-0.1905	1.35E-05	3.54E-05
FLJ25363	0.218858	5.29E-07	1.66E-06
FLJ25758	0.166077	1.53E-04	3.47E-04
FLJ26850	-0.20637	2.33E-06	6.73E-06
FLJ30679	0.204972	2.73E-06	7.85E-06
FLJ32063	0.106654	1.55E-02	2.53E-02
FLJ33360	-0.35576	8.23E-17	7.17E-16
FLJ33630	-0.23106	1.14E-07	3.87E-07
FLJ34503	-0.20661	2.26E-06	6.57E-06
FLJ35024	-0.12679	3.95E-03	7.21E-03
FLJ35220	-0.18981	1.45E-05	3.79E-05
FLJ35390	-0.11191	1.10E-02	1.86E-02
FLJ35776	-0.07308	9.76E-02	1.35E-01

FLJ36000	0.169031	1.16E-04	2.68E-04
FLJ36031	0.360061	3.29E-17	3.01E-16
FLJ36777	-0.35687	6.50E-17	5.74E-16
FLJ37201	0.139531	1.50E-03	2.93E-03
FLJ37307	-0.14877	7.07E-04	1.45E-03
FLJ37453	-0.11002	1.25E-02	2.08E-02
FLJ37543	-0.11598	8.43E-03	1.45E-02
FLJ39582	0.002901	9.48E-01	9.59E-01
FLJ39609	-0.17267	8.19E-05	1.93E-04
FLJ39653	-0.15557	3.95E-04	8.42E-04
FLJ39739	0.15623	3.73E-04	7.99E-04
FLJ40292	0.049062	2.66E-01	3.28E-01
FLJ40330	-0.03836	3.85E-01	4.51E-01
FLJ40504	0.052858	2.31E-01	2.90E-01
FLJ40852	-0.2305	1.23E-07	4.15E-07
FLJ41941	0.058709	1.83E-01	2.36E-01
FLJ42289	-0.27677	1.65E-10	7.70E-10
FLJ42393	-0.10263	1.98E-02	3.18E-02
FLJ42627	-0.17593	5.96E-05	1.44E-04
FLJ42709	-0.13221	2.64E-03	4.96E-03
FLJ42875	-0.57585	8.04E-47	5.05E-45
FLJ43390	-0.25097	7.73E-09	2.99E-08
FLJ43663	-0.25991	2.14E-09	8.78E-09
FLJ43859	0.079949	6.99E-02	1.00E-01
FLJ43860	-0.0414	3.48E-01	4.14E-01
FLJ43950	0.02136	6.29E-01	6.84E-01
FLJ44054	0.106733	1.54E-02	2.52E-02
FLJ44606	-0.27882	1.19E-10	5.64E-10
FLJ44635	-0.11003	1.25E-02	2.08E-02
FLJ45079	-0.11084	1.18E-02	1.98E-02
FLJ45244	-0.33466	6.09E-15	4.41E-14
FLJ45340	-0.22085	4.14E-07	1.32E-06
FLJ45445	-0.05549	2.09E-01	2.65E-01
FLJ45983	0.128295	3.54E-03	6.51E-03
FLJ46111	0.110838	1.18E-02	1.98E-02
FLJ46321	0.042899	3.31E-01	3.97E-01
FLJ46361	-0.07588	8.54E-02	1.20E-01
FLJ90757	-0.47709	1.24E-30	3.33E-29
FLNA	-0.12072	6.09E-03	1.08E-02
FLNB	-0.06631	1.33E-01	1.78E-01
FLNC	0.204334	2.94E-06	8.41E-06
FLOT1	0.012228	7.82E-01	8.19E-01
FLOT2	-0.18202	3.25E-05	8.09E-05
FLRT1	-0.02225	6.14E-01	6.71E-01

FLRT2	0.064294	1.45E-01	1.92E-01
FLRT3	-0.3628	1.82E-17	1.70E-16
FLT1	0.08941	4.25E-02	6.37E-02
FLT3LG	-0.16078	2.49E-04	5.47E-04
FLT3	-0.22002	4.59E-07	1.45E-06
FLT4	-0.20628	2.35E-06	6.80E-06
FLVCR1	0.187057	1.93E-05	4.98E-05
FLVCR2	-0.00584	8.95E-01	9.15E-01
FLYWCH1	-0.3129	3.68E-13	2.26E-12
FLYWCH2	-0.15743	3.35E-04	7.24E-04
FMN1	-0.20164	3.98E-06	1.12E-05
FMN2	-0.09457	3.19E-02	4.89E-02
FMNL1	-0.03315	4.53E-01	5.19E-01
FMNL2	-0.19741	6.38E-06	1.75E-05
FMNL3	-0.09233	3.62E-02	5.49E-02
FMO1	0.117805	7.45E-03	1.29E-02
FMO2	-0.38993	3.79E-20	4.48E-19
FMO3	-0.33183	1.06E-14	7.49E-14
FMO4	-0.36401	1.40E-17	1.31E-16
FMO5	-0.44972	5.24E-27	1.08E-25
FMO6P	-0.10007	2.31E-02	3.66E-02
FMO9P	0.091885	3.71E-02	5.62E-02
FMOD	-0.19391	9.34E-06	2.51E-05
FMR1NB	0.098865	2.49E-02	3.90E-02
FMR1	0.088173	4.55E-02	6.77E-02
FN1	0.178198	4.77E-05	1.16E-04
FN3KRP	0.136438	1.91E-03	3.68E-03
FN3K	-0.22327	3.07E-07	9.92E-07
FNBP1L	-0.08004	6.95E-02	9.96E-02
FNBP1	-0.03405	4.41E-01	5.07E-01
FNBP4	-0.22162	3.76E-07	1.21E-06
FNDC1	0.079257	7.23E-02	1.03E-01
FNDC3A	-0.19392	9.33E-06	2.51E-05
FNDC3B	0.309234	7.11E-13	4.24E-12
FNDC4	0.173499	7.56E-05	1.79E-04
FNDC5	-0.21629	7.22E-07	2.23E-06
FNDC7	0.075831	8.56E-02	1.20E-01
FNDC8	0.176711	5.52E-05	1.34E-04
FNIP1	-0.09508	3.10E-02	4.77E-02
FNIP2	-0.24382	2.09E-08	7.67E-08
FNTA	0.245775	1.60E-08	5.94E-08
FNTB	-0.08302	5.97E-02	8.67E-02
FOLH1B	0.135835	2.01E-03	3.84E-03
FOLH1	0.136095	1.97E-03	3.77E-03

FOLR1	-0.55054	3.78E-42	1.94E-40
FOLR2	-0.23687	5.33E-08	1.87E-07
FOLR3	-0.13201	2.69E-03	5.04E-03
FOLR4	-0.07801	7.70E-02	1.09E-01
FOSB	-0.22904	1.48E-07	4.96E-07
FOSL1	0.377348	7.14E-19	7.49E-18
FOSL2	0.126194	4.13E-03	7.51E-03
FOS	-0.36431	1.31E-17	1.23E-16
FOXA1	0.150122	6.31E-04	1.31E-03
FOXA2	-0.42327	8.40E-24	1.36E-22
FOXA3	-0.02515	5.69E-01	6.29E-01
FOXB1	0.107948	1.42E-02	2.35E-02
FOXB2	0.099263	2.43E-02	3.82E-02
FOXC1	0.080948	6.64E-02	9.56E-02
FOXC2	-0.03736	3.97E-01	4.64E-01
FOXD1	0.271053	4.01E-10	1.80E-09
FOXD2	0.062995	1.53E-01	2.02E-01
FOXD3	0.3013	2.87E-12	1.61E-11
FOXD4L1	-0.17353	7.54E-05	1.79E-04
FOXD4L2	-0.18855	1.65E-05	4.30E-05
FOXD4L3	-0.06088	1.68E-01	2.18E-01
FOXD4L5	-0.17759	5.06E-05	1.23E-04
FOXD4L6	-0.1922	1.12E-05	2.98E-05
FOXD4	-0.24655	1.43E-08	5.36E-08
FOXE1	0.105863	1.62E-02	2.65E-02
FOXE3	0.222754	3.27E-07	1.06E-06
FOXF1	-0.39284	1.89E-20	2.29E-19
FOXF2	-0.38498	1.22E-19	1.38E-18
FOXG1	0.291636	1.48E-11	7.68E-11
FOXH1	0.065204	1.39E-01	1.85E-01
FOXI1	-0.1016	2.11E-02	3.36E-02
FOXI2	-0.34257	1.26E-15	9.73E-15
FOXI3	0.119074	6.82E-03	1.19E-02
FOXJ1	-0.16347	1.95E-04	4.36E-04
FOXJ2	-0.13826	1.66E-03	3.22E-03
FOXJ3	-0.00049	9.91E-01	9.93E-01
FOXK1	0.074987	8.91E-02	1.24E-01
FOXK2	0.290348	1.84E-11	9.43E-11
FOXL1	-0.1239	4.87E-03	8.76E-03
FOXL2	0.222147	3.53E-07	1.13E-06
FOXM1	0.831018	8.88E-133	2.73E-130
FOXN1	-0.26593	8.74E-10	3.76E-09
FOXN2	0.327668	2.36E-14	1.62E-13
FOXN3	-0.2676	6.79E-10	2.96E-09

FOXN4	0.085864	5.15E-02	7.57E-02
FOXO1	-0.33113	1.21E-14	8.52E-14
FOXO3B	-0.18887	1.60E-05	4.17E-05
FOXO3	-0.25589	3.83E-09	1.53E-08
FOXO4	-0.34184	1.46E-15	1.12E-14
FOXP1	-0.36194	2.19E-17	2.04E-16
FOXP2	0.058913	1.82E-01	2.35E-01
FOXP3	0.054813	2.14E-01	2.71E-01
FOXP4	-0.2632	1.31E-09	5.54E-09
FOXQ1	-0.27986	1.01E-10	4.80E-10
FOXR1	0.066724	1.30E-01	1.75E-01
FOXR2	0.107396	1.48E-02	2.43E-02
FOXRED1	0.136808	1.86E-03	3.58E-03
FOXRED2	0.130374	3.04E-03	5.65E-03
FOXS1	-0.26189	1.60E-09	6.67E-09
FPGS	-0.15914	2.88E-04	6.28E-04
FPGT	-0.00103	9.81E-01	9.86E-01
FPR1	0.058902	1.82E-01	2.35E-01
FPR2	0.153821	4.60E-04	9.70E-04
FPR3	0.053831	2.23E-01	2.80E-01
FRAS1	-0.19253	1.08E-05	2.88E-05
FRAT1	-0.52526	7.29E-38	2.96E-36
FRAT2	-0.1177	7.50E-03	1.30E-02
FREM1	-0.10631	1.58E-02	2.59E-02
FREM2	-0.35838	4.72E-17	4.24E-16
FRG1B	-0.13521	2.10E-03	4.01E-03
FRG1	0.160491	2.55E-04	5.61E-04
FRG2B	0.083519	5.82E-02	8.47E-02
FRG2C	0.006633	8.81E-01	9.03E-01
FRG2	0.064764	1.42E-01	1.88E-01
FRK	0.107592	1.46E-02	2.40E-02
FRMD1	-0.05725	1.95E-01	2.49E-01
FRMD3	-0.04102	3.53E-01	4.19E-01
FRMD4A	-0.11001	1.25E-02	2.08E-02
FRMD4B	-0.26091	1.84E-09	7.64E-09
FRMD5	0.380358	3.58E-19	3.87E-18
FRMD6	0.191003	1.28E-05	3.37E-05
FRMD7	0.020966	6.35E-01	6.90E-01
FRMD8	0.100879	2.20E-02	3.50E-02
FRMPD1	0.031624	4.74E-01	5.40E-01
FRMPD2L1	-0.11002	1.25E-02	2.08E-02
FRMPD2	-0.12522	4.43E-03	8.02E-03
FRMPD4	-0.25103	7.67E-09	2.96E-08
FRRS1	0.258383	2.67E-09	1.09E-08

FRS2	-0.04996	2.58E-01	3.19E-01
FRS3	-0.3006	3.24E-12	1.81E-11
FRYL	0.01451	7.43E-01	7.86E-01
FRY	-0.45247	2.34E-27	4.98E-26
FRZB	-0.40514	9.17E-22	1.23E-20
FSCB	-0.02697	5.41E-01	6.04E-01
FSCN1	0.338631	2.77E-15	2.07E-14
FSCN2	-0.2221	3.55E-07	1.14E-06
FSCN3	-0.05618	2.03E-01	2.58E-01
FSD1L	0.182192	3.19E-05	7.96E-05
FSD1	0.380474	3.48E-19	3.77E-18
FSD2	-0.12338	5.05E-03	9.06E-03
FSHB	0.018397	6.77E-01	7.28E-01
FSHR	-0.17293	7.99E-05	1.89E-04
FSIP1	0.040222	3.62E-01	4.29E-01
FSTL1	0.039131	3.76E-01	4.42E-01
FSTL3	-0.05669	1.99E-01	2.54E-01
FSTL4	-0.14403	1.05E-03	2.10E-03
FSTL5	0.246752	1.39E-08	5.22E-08
FST	0.117559	7.57E-03	1.31E-02
FTCD	0.171421	9.24E-05	2.16E-04
FTH1	0.092494	3.59E-02	5.45E-02
FTHL17	0.127454	3.77E-03	6.89E-03
FTHL3	0.130032	3.11E-03	5.78E-03
FTLP10	-0.01418	7.48E-01	7.90E-01
FTL	9.68E-05	9.98E-01	9.99E-01
FTMT	0.023374	5.97E-01	6.55E-01
FTO	-0.30064	3.22E-12	1.80E-11
FTSJ1	0.299984	3.60E-12	2.00E-11
FTSJ2	0.181643	3.37E-05	8.39E-05
FTSJ3	0.243728	2.11E-08	7.76E-08
FTSJD1	-0.09242	3.60E-02	5.47E-02
FTSJD2	0.042834	3.32E-01	3.97E-01
FUBP1	0.283429	5.71E-11	2.77E-10
FUBP3	0.223498	2.98E-07	9.66E-07
FUCA1	-0.37203	2.38E-18	2.40E-17
FUCA2	0.458157	4.33E-28	9.73E-27
FUK	-0.36821	5.56E-18	5.39E-17
FUNDC1	0.087945	4.61E-02	6.85E-02
FUNDC2P2	0.118728	6.99E-03	1.22E-02
FUNDC2	0.061816	1.61E-01	2.11E-01
FURIN	0.013102	7.67E-01	8.06E-01
FUS	0.10682	1.53E-02	2.51E-02
FUT10	-0.05539	2.09E-01	2.65E-01

FUT11	0.129287	3.29E-03	6.09E-03
FUT1	-0.11879	6.96E-03	1.21E-02
FUT2	-0.08759	4.70E-02	6.96E-02
FUT3	-0.17996	4.00E-05	9.84E-05
FUT4	0.124866	4.54E-03	8.21E-03
FUT5	0.120208	6.31E-03	1.11E-02
FUT6	-0.09814	2.59E-02	4.06E-02
FUT7	-0.14133	1.30E-03	2.57E-03
FUT8	0.018568	6.74E-01	7.25E-01
FUT9	0.163073	2.02E-04	4.51E-04
FUZ	-0.20937	1.64E-06	4.85E-06
FXC1	-0.31248	3.97E-13	2.43E-12
FXN	-0.05273	2.32E-01	2.91E-01
FXR1	0.396489	7.80E-21	9.79E-20
FXR2	-0.10247	2.00E-02	3.21E-02
FXYD1	-0.58391	2.14E-48	1.40E-46
FXYD2	0.004956	9.11E-01	9.28E-01
FXYD3	-0.21023	1.49E-06	4.40E-06
FXYD4	-0.15582	3.86E-04	8.25E-04
FXYD5	-0.01308	7.67E-01	8.06E-01
FXYD6	-0.22635	2.08E-07	6.87E-07
FXYD7	-0.13488	2.16E-03	4.11E-03
FYB	0.078116	7.65E-02	1.09E-01
FYCO1	-0.43735	1.79E-25	3.28E-24
FYN	-0.05212	2.38E-01	2.97E-01
FYTTD1	0.390999	2.94E-20	3.52E-19
FZD10	0.088033	4.58E-02	6.82E-02
FZD1	-0.19685	6.78E-06	1.86E-05
FZD2	0.027286	5.37E-01	5.99E-01
FZD3	-0.0473	2.84E-01	3.46E-01
FZD4	-0.19501	8.29E-06	2.24E-05
FZD5	-0.17771	5.00E-05	1.22E-04
FZD6	0.033555	4.47E-01	5.14E-01
FZD7	-0.1903	1.37E-05	3.61E-05
FZD8	-0.29709	5.91E-12	3.20E-11
FZD9	-0.08169	6.40E-02	9.24E-02
FZR1	0.046078	2.97E-01	3.60E-01
G0S2	0.173541	7.53E-05	1.79E-04
G2E3	0.465378	4.85E-29	1.17E-27
G3BP1	0.362013	2.16E-17	2.01E-16
G3BP2	0.114707	9.18E-03	1.57E-02
G6PC2	-0.18065	3.73E-05	9.22E-05
G6PC3	-0.05335	2.27E-01	2.85E-01
G6PC	0.109063	1.33E-02	2.20E-02

G6PD	0.237955	4.61E-08	1.63E-07
GAA	-0.01356	7.59E-01	7.99E-01
GAB1	-0.21391	9.61E-07	2.92E-06
GAB2	-0.20335	3.28E-06	9.36E-06
GAB3	-0.28444	4.84E-11	2.37E-10
GAB4	0.124112	4.79E-03	8.64E-03
GABARAPL1	-0.06682	1.30E-01	1.74E-01
GABARAPL2	-0.20605	2.41E-06	6.97E-06
GABARAPL3	0.004581	9.17E-01	9.34E-01
GABARAP	-0.21005	1.52E-06	4.49E-06
GABBR1	-0.26458	1.07E-09	4.56E-09
GABBR2	-0.11621	8.30E-03	1.43E-02
GABPA	-0.06457	1.43E-01	1.90E-01
GABPB1	0.493028	6.73E-33	2.06E-31
GABPB2	-0.14524	9.48E-04	1.91E-03
GABRA1	0.128438	3.50E-03	6.45E-03
GABRA2	0.206107	2.40E-06	6.93E-06
GABRA3	0.26398	1.17E-09	4.96E-09
GABRA4	-0.13017	3.08E-03	5.73E-03
GABRA5	0.056414	2.01E-01	2.56E-01
GABRA6	-0.00715	8.71E-01	8.95E-01
GABRB1	-0.03404	4.41E-01	5.07E-01
GABRB2	-0.0326	4.60E-01	5.26E-01
GABRB3	-0.08837	4.50E-02	6.71E-02
GABRD	-0.12176	5.66E-03	1.01E-02
GABRE	-0.22093	4.10E-07	1.31E-06
GABRG1	-0.10381	1.85E-02	2.98E-02
GABRG2	0.198282	5.79E-06	1.60E-05
GABRG3	-0.03617	4.13E-01	4.79E-01
GABRP	-0.06588	1.35E-01	1.81E-01
GABRQ	0.303476	1.97E-12	1.13E-11
GABRR1	0.084361	5.57E-02	8.14E-02
GABRR2	-0.02143	6.28E-01	6.83E-01
GABRR3	0.030744	4.86E-01	5.51E-01
GAD1	0.266181	8.41E-10	3.63E-09
GAD2	0.110186	1.23E-02	2.06E-02
GADD45A	0.214757	8.69E-07	2.65E-06
GADD45B	-0.13735	1.78E-03	3.44E-03
GADD45GIP1	0.187531	1.84E-05	4.76E-05
GADD45G	-0.29349	1.09E-11	5.71E-11
GADL1	-0.22011	4.54E-07	1.44E-06
GAGE10	0.072257	1.01E-01	1.39E-01
GAGE12D	0.193878	9.37E-06	2.52E-05
GAGE12F	0.077166	8.02E-02	1.13E-01

GAGE12J	0.177764	4.98E-05	1.21E-04
GAGE13	0.117474	7.61E-03	1.32E-02
GAGE1	0.192882	1.04E-05	2.78E-05
GAGE2A	0.159288	2.84E-04	6.20E-04
GAGE2B	0.175257	6.37E-05	1.53E-04
GAGE2C	0.148367	7.31E-04	1.50E-03
GAGE2D	0.21996	4.62E-07	1.46E-06
GAGE2E	0.122517	5.37E-03	9.58E-03
GAGE4	0.198305	5.78E-06	1.60E-05
GAGE8	0.161373	2.36E-04	5.21E-04
GAK	-0.07672	8.19E-02	1.15E-01
GAL3ST1	-0.28716	3.11E-11	1.55E-10
GAL3ST2	0.190249	1.38E-05	3.63E-05
GAL3ST3	0.174643	6.77E-05	1.62E-04
GAL3ST4	0.110565	1.20E-02	2.02E-02
GALC	-0.16467	1.74E-04	3.93E-04
GALE	-0.02727	5.37E-01	6.00E-01
GALK1	0.204461	2.89E-06	8.30E-06
GALK2	0.0003	9.95E-01	9.96E-01
GALM	0.026014	5.56E-01	6.17E-01
GALNS	0.034143	4.39E-01	5.06E-01
GALNT10	-0.26959	5.02E-10	2.22E-09
GALNT11	-0.45283	2.11E-27	4.50E-26
GALNT12	-0.28997	1.96E-11	1.00E-10
GALNT13	0.098195	2.59E-02	4.04E-02
GALNT14	0.235824	6.12E-08	2.13E-07
GALNT1	0.122201	5.49E-03	9.78E-03
GALNT2	0.174634	6.77E-05	1.62E-04
GALNT3	0.146445	8.58E-04	1.74E-03
GALNT4	0.200085	4.74E-06	1.33E-05
GALNT5	-0.07223	1.02E-01	1.40E-01
GALNT6	0.240201	3.41E-08	1.22E-07
GALNT7	0.0458	3.00E-01	3.63E-01
GALNT8	0.092583	3.57E-02	5.43E-02
GALNT9	0.133081	2.48E-03	4.67E-03
GALNTL1	-0.30791	9.01E-13	5.31E-12
GALNTL2	-0.17015	1.04E-04	2.43E-04
GALNTL4	-0.39459	1.24E-20	1.53E-19
GALNTL5	-0.01659	7.07E-01	7.55E-01
GALNTL6	-0.16072	2.50E-04	5.50E-04
GALP	0.143585	1.09E-03	2.17E-03
GALR1	0.095201	3.08E-02	4.74E-02
GALR2	0.124279	4.74E-03	8.54E-03
GALR3	0.098824	2.49E-02	3.91E-02

GALT	-0.23896	4.03E-08	1.43E-07
GAL	0.429177	1.71E-24	2.90E-23
GAMT	-0.00279	9.50E-01	9.60E-01
GANAB	0.298969	4.29E-12	2.36E-11
GANC	-0.54715	1.49E-41	7.35E-40
GAN	0.185761	2.21E-05	5.66E-05
GAP43	0.283401	5.73E-11	2.78E-10
GAPDHS	0.029387	5.06E-01	5.70E-01
GAPDH	0.606764	4.14E-53	3.02E-51
GAPT	-0.33474	5.98E-15	4.34E-14
GAPVD1	-0.14445	1.01E-03	2.03E-03
GAR1	0.281493	7.79E-11	3.74E-10
GARNL3	-0.35979	3.49E-17	3.18E-16
GARS	0.446645	1.28E-26	2.55E-25
GART	0.452195	2.54E-27	5.39E-26
GAS1	0.024064	5.86E-01	6.44E-01
GAS2L1	0.074644	9.06E-02	1.26E-01
GAS2L2	-0.36284	1.81E-17	1.69E-16
GAS2L3	0.544052	5.16E-41	2.49E-39
GAS2	-0.02164	6.24E-01	6.80E-01
GAS5	-0.13128	2.84E-03	5.30E-03
GAS6	-0.34469	8.20E-16	6.50E-15
GAS7	-0.26357	1.24E-09	5.26E-09
GAS8	-0.32609	3.18E-14	2.16E-13
GAST	0.213108	1.06E-06	3.19E-06
GATA1	-0.3441	9.23E-16	7.25E-15
GATA2	-0.21835	5.63E-07	1.76E-06
GATA3	0.062522	1.57E-01	2.05E-01
GATA4	-0.02919	5.09E-01	5.72E-01
GATA5	-0.42276	9.61E-24	1.55E-22
GATA6	-0.33079	1.29E-14	9.08E-14
GATAD1	-0.07065	1.09E-01	1.49E-01
GATAD2A	0.054489	2.17E-01	2.74E-01
GATAD2B	-0.16741	1.35E-04	3.09E-04
GATC	0.415171	7.08E-23	1.04E-21
GATM	-0.04546	3.03E-01	3.67E-01
GATSL1	0.30109	2.98E-12	1.67E-11
GATSL2	0.183696	2.74E-05	6.90E-05
GATSL3	-0.30771	9.34E-13	5.50E-12
GATS	0.102729	1.97E-02	3.16E-02
GBA2	-0.11965	6.56E-03	1.15E-02
GBA3	-0.05875	1.83E-01	2.36E-01
GBAP1	0.129845	3.16E-03	5.86E-03
GBAS	0.081622	6.42E-02	9.27E-02

GBA	0.118382	7.16E-03	1.25E-02
GBE1	0.250222	8.59E-09	3.30E-08
GBF1	-0.11201	1.10E-02	1.85E-02
GBGT1	-0.25519	4.24E-09	1.69E-08
GBP1	0.358413	4.68E-17	4.21E-16
GBP2	0.184512	2.52E-05	6.38E-05
GBP3	0.0573	1.94E-01	2.49E-01
GBP4	0.122312	5.45E-03	9.71E-03
GBP5	0.251418	7.26E-09	2.81E-08
GBP6	0.050727	2.51E-01	3.11E-01
GBP7	0.161626	2.30E-04	5.10E-04
GBX1	0.154622	4.29E-04	9.10E-04
GBX2	0.403967	1.23E-21	1.64E-20
GCAT	-0.10065	2.24E-02	3.54E-02
GCA	0.119176	6.78E-03	1.19E-02
GCC1	0.070044	1.12E-01	1.53E-01
GCC2	-0.23825	4.43E-08	1.57E-07
GCDH	-0.20189	3.87E-06	1.09E-05
GCET2	-0.25271	6.04E-09	2.36E-08
GCFC1	-0.03157	4.75E-01	5.40E-01
GCGR	0.110427	1.22E-02	2.03E-02
GCG	0.07424	9.24E-02	1.28E-01
GCH1	0.302554	2.31E-12	1.31E-11
GCHFR	-0.05995	1.74E-01	2.26E-01
GCKR	0.104088	1.81E-02	2.93E-02
GCK	-0.02384	5.89E-01	6.48E-01
GCLC	0.344176	9.09E-16	7.16E-15
GCLM	0.297071	5.94E-12	3.21E-11
GCM1	0.022237	6.15E-01	6.71E-01
GCM2	0.074421	9.16E-02	1.27E-01
GCN1L1	0.300041	3.57E-12	1.98E-11
GCNT1	0.083182	5.92E-02	8.61E-02
GCNT2	-0.01795	6.84E-01	7.34E-01
GCNT3	0.016275	7.13E-01	7.60E-01
GCNT4	-0.25769	2.95E-09	1.20E-08
GCNT7	-0.12299	5.19E-03	9.29E-03
GCOM1	-0.23405	7.73E-08	2.67E-07
GCSH	0.166486	1.47E-04	3.35E-04
GC	-0.10958	1.28E-02	2.14E-02
GDAP1L1	-0.05031	2.54E-01	3.15E-01
GDAP1	0.072605	9.98E-02	1.37E-01
GDAP2	0.055682	2.07E-01	2.63E-01
GDA	0.168068	1.27E-04	2.92E-04
GDE1	-0.26931	5.23E-10	2.31E-09

GDEP	-0.06388	1.48E-01	1.95E-01
GDF10	-0.43763	1.66E-25	3.05E-24
GDF11	0.076813	8.16E-02	1.15E-01
GDF15	-0.27961	1.05E-10	4.98E-10
GDF1	0.244257	1.97E-08	7.25E-08
GDF2	-0.15163	5.55E-04	1.16E-03
GDF3	-0.16895	1.17E-04	2.70E-04
GDF5	-0.16021	2.62E-04	5.74E-04
GDF6	0.071286	1.06E-01	1.45E-01
GDF7	-0.25289	5.89E-09	2.31E-08
GDF9	-0.06277	1.55E-01	2.03E-01
GDI1	0.146521	8.53E-04	1.73E-03
GDI2	0.262562	1.44E-09	6.06E-09
GDNF	0.061901	1.61E-01	2.10E-01
GDPD1	0.008075	8.55E-01	8.82E-01
GDPD2	0.004353	9.21E-01	9.37E-01
GDPD3	0.000279	9.95E-01	9.96E-01
GDPD4	0.101446	2.13E-02	3.39E-02
GDPD5	-0.16689	1.42E-04	3.23E-04
GEFT	0.004723	9.15E-01	9.32E-01
GEMIN4	-0.1213	5.85E-03	1.04E-02
GEMIN5	0.078372	7.56E-02	1.07E-01
GEMIN6	0.38192	2.49E-19	2.75E-18
GEMIN7	0.284341	4.92E-11	2.40E-10
GEMIN8P4	0.274437	2.38E-10	1.09E-09
GEMIN8	-0.3195	1.10E-13	7.07E-13
GEM	-0.30302	2.13E-12	1.21E-11
GEN1	0.619217	7.66E-56	5.95E-54
GET4	0.12664	4.00E-03	7.29E-03
GFAP	0.260334	2.01E-09	8.28E-09
GFER	-0.06596	1.35E-01	1.80E-01
GFI1B	-0.32059	8.96E-14	5.84E-13
GFI1	0.104669	1.75E-02	2.84E-02
GFM1	0.384389	1.40E-19	1.58E-18
GFM2	0.215886	7.58E-07	2.33E-06
GFOD1	-0.09937	2.41E-02	3.80E-02
GFOD2	-0.03756	3.95E-01	4.62E-01
GFPT1	0.115537	8.68E-03	1.49E-02
GFPT2	0.248755	1.06E-08	4.01E-08
GFRA1	-0.57298	2.86E-46	1.76E-44
GFRA2	-0.29554	7.70E-12	4.11E-11
GFRA3	-0.25496	4.38E-09	1.74E-08
GFRA4	0.108025	1.42E-02	2.34E-02
GFRAL	0.066841	1.30E-01	1.74E-01

GGA1	-0.2287	1.55E-07	5.17E-07
GGA2	-0.44766	9.52E-27	1.93E-25
GGA3	-0.12339	5.05E-03	9.06E-03
GGCT	0.183452	2.81E-05	7.06E-05
GGCX	-0.21283	1.09E-06	3.29E-06
GGH	0.574708	1.34E-46	8.29E-45
GGNBP1	0.110659	1.20E-02	2.01E-02
GGNBP2	-0.00384	9.31E-01	9.45E-01
GGN	-0.06488	1.41E-01	1.88E-01
GGPS1	-0.15568	3.91E-04	8.35E-04
GGT1	-0.21415	9.34E-07	2.84E-06
GGT3P	-0.3814	2.81E-19	3.07E-18
GGT5	-0.30976	6.47E-13	3.88E-12
GGT6	-0.46416	7.04E-29	1.68E-27
GGT7	-0.317	1.74E-13	1.10E-12
GGT8P	-0.19719	6.53E-06	1.79E-05
GGTA1	-0.28719	3.10E-11	1.55E-10
GGTLC1	-0.5804	1.05E-47	6.78E-46
GGTLC2	-0.27319	2.88E-10	1.32E-09
GH1	-0.0229	6.04E-01	6.61E-01
GH2	0.148892	7.00E-04	1.44E-03
GHDC	-0.42102	1.53E-23	2.40E-22
GHITM	0.086433	5.00E-02	7.37E-02
GHRHR	0.014395	7.45E-01	7.87E-01
GHRH	0.077703	7.81E-02	1.11E-01
GHRLOS	-0.10767	1.45E-02	2.39E-02
GHRL	-0.34926	3.21E-16	2.64E-15
GHR	-0.31995	1.01E-13	6.53E-13
GHSR	0.015241	7.30E-01	7.75E-01
GIF	-0.0444	3.15E-01	3.79E-01
GIGYF1	-0.18018	3.91E-05	9.64E-05
GIGYF2	-0.14935	6.74E-04	1.39E-03
GIMAP1	-0.23498	6.84E-08	2.38E-07
GIMAP2	-0.10587	1.62E-02	2.65E-02
GIMAP4	-0.08167	6.40E-02	9.25E-02
GIMAP5	-0.07662	8.24E-02	1.16E-01
GIMAP6	-0.17361	7.49E-05	1.78E-04
GIMAP7	-0.18536	2.31E-05	5.88E-05
GIMAP8	-0.23068	1.20E-07	4.06E-07
GIN1	-0.24855	1.09E-08	4.12E-08
GINS1	0.759069	1.09E-97	1.68E-95
GINS2	0.773957	7.27E-104	1.24E-101
GINS3	0.698867	1.09E-76	1.28E-74
GINS4	0.699638	6.36E-77	7.48E-75

GIPC1	0.00329	9.41E-01	9.54E-01
GIPC2	-0.0255	5.64E-01	6.24E-01
GIPC3	-0.10152	2.12E-02	3.38E-02
GIPR	-0.33137	1.15E-14	8.15E-14
GIP	0.259937	2.13E-09	8.75E-09
GIT1	0.213203	1.05E-06	3.16E-06
GIT2	-0.06339	1.51E-01	1.99E-01
GIYD2	-0.01134	7.97E-01	8.33E-01
GJA10	0.132943	2.50E-03	4.71E-03
GJA1	0.079654	7.09E-02	1.01E-01
GJA3	0.151431	5.65E-04	1.18E-03
GJA4	-0.1616	2.31E-04	5.11E-04
GJA5	-0.114	9.62E-03	1.64E-02
GJA8	0.047136	2.86E-01	3.48E-01
GJA9	-0.01218	7.83E-01	8.20E-01
GJB1	-0.3983	5.01E-21	6.42E-20
GJB2	0.372653	2.07E-18	2.09E-17
GJB3	0.248976	1.02E-08	3.89E-08
GJB4	0.080097	6.93E-02	9.94E-02
GJB5	0.099257	2.43E-02	3.82E-02
GJB6	0.120179	6.32E-03	1.11E-02
GJB7	-0.03216	4.66E-01	5.32E-01
GJC1	0.195254	8.07E-06	2.19E-05
GJC2	-0.32089	8.47E-14	5.53E-13
GJC3	0.127968	3.63E-03	6.66E-03
GJD2	0.087658	4.68E-02	6.94E-02
GJD3	0.091423	3.81E-02	5.76E-02
GJD4	0.087559	4.70E-02	6.97E-02
GK2	-0.00427	9.23E-01	9.39E-01
GK3P	-0.09463	3.18E-02	4.88E-02
GK5	0.02941	5.05E-01	5.70E-01
GKAP1	-0.14249	1.19E-03	2.35E-03
GKN1	-0.07556	8.67E-02	1.21E-01
GKN2	-0.45023	4.52E-27	9.38E-26
GK	-0.14633	8.66E-04	1.76E-03
GLA	0.416678	4.78E-23	7.19E-22
GLB1L2	-0.33776	3.30E-15	2.45E-14
GLB1L3	-0.29948	3.93E-12	2.17E-11
GLB1L	-0.22902	1.48E-07	4.98E-07
GLB1	-0.14831	7.35E-04	1.51E-03
GLCCI1	-0.33884	2.66E-15	1.99E-14
GLCE	0.029193	5.09E-01	5.72E-01
GLDC	0.288063	2.68E-11	1.35E-10
GLDN	-0.17549	6.23E-05	1.50E-04

GLE1	-0.0307	4.87E-01	5.52E-01
GLG1	-0.05138	2.44E-01	3.05E-01
GLI1	-0.28117	8.21E-11	3.93E-10
GLI2	-0.14009	1.44E-03	2.82E-03
GLI3	-0.04627	2.95E-01	3.58E-01
GLI4	-0.08971	4.19E-02	6.27E-02
GLIPR1L1	0.051506	2.43E-01	3.03E-01
GLIPR1L2	-0.28513	4.34E-11	2.13E-10
GLIPR1	0.026979	5.41E-01	6.04E-01
GLIPR2	-0.03505	4.27E-01	4.94E-01
GLIS1	0.044965	3.08E-01	3.72E-01
GLIS2	-0.20612	2.39E-06	6.92E-06
GLIS3	-0.10002	2.32E-02	3.67E-02
GLMN	0.41958	2.23E-23	3.47E-22
GLO1	0.206244	2.36E-06	6.83E-06
GLOD4	0.121676	5.69E-03	1.01E-02
GLOD5	-0.41713	4.25E-23	6.43E-22
GLP1R	-0.28928	2.19E-11	1.11E-10
GLP2R	-0.04759	2.81E-01	3.43E-01
GLRA1	0.087796	4.64E-02	6.89E-02
GLRA2	0.027911	5.27E-01	5.91E-01
GLRA3	-0.08651	4.98E-02	7.34E-02
GLRA4	-0.01451	7.42E-01	7.86E-01
GLRB	0.027565	5.33E-01	5.96E-01
GLRX2	0.295831	7.33E-12	3.92E-11
GLRX3	0.392223	2.19E-20	2.65E-19
GLRX5	0.300745	3.16E-12	1.76E-11
GLRX	0.156735	3.57E-04	7.66E-04
GLS2	-0.37296	1.93E-18	1.96E-17
GLS	0.011355	7.97E-01	8.32E-01
GLT1D1	0.017456	6.93E-01	7.42E-01
GLT25D1	0.417739	3.62E-23	5.51E-22
GLT25D2	-0.23234	9.66E-08	3.30E-07
GLT8D1	-0.11618	8.32E-03	1.43E-02
GLT8D2	-0.02863	5.17E-01	5.81E-01
GLTPD1	-0.04727	2.84E-01	3.47E-01
GLTPD2	-0.23904	3.99E-08	1.42E-07
GLTP	-0.12116	5.90E-03	1.05E-02
GLTSCR1	-0.16522	1.66E-04	3.74E-04
GLTSCR2	-0.39402	1.42E-20	1.75E-19
GLUD1	0.007945	8.57E-01	8.84E-01
GLUD2	0.077077	8.06E-02	1.14E-01
GLUL	-0.18464	2.48E-05	6.30E-05
GLYATL1	0.163933	1.87E-04	4.18E-04

GLYATL2	-0.03238	4.63E-01	5.29E-01
GLYATL3	0.130434	3.02E-03	5.62E-03
GLYAT	0.023636	5.93E-01	6.51E-01
GLYCTK	-0.04635	2.94E-01	3.57E-01
GLYR1	-0.34186	1.45E-15	1.11E-14
GM2A	-0.0026	9.53E-01	9.63E-01
GMCL1L	0.107753	1.44E-02	2.38E-02
GMCL1	0.197484	6.32E-06	1.74E-05
GMDS	0.135793	2.01E-03	3.85E-03
GMEB1	0.245572	1.64E-08	6.10E-08
GMEB2	0.081696	6.39E-02	9.24E-02
GMFB	0.325736	3.40E-14	2.30E-13
GMFG	-0.14558	9.22E-04	1.86E-03
GMIP	-0.09084	3.93E-02	5.93E-02
GML	0.005984	8.92E-01	9.13E-01
GMNN	0.631112	1.43E-58	1.20E-56
GMPPA	0.118912	6.90E-03	1.21E-02
GMPPB	-0.02636	5.51E-01	6.12E-01
GMPR2	-0.09351	3.39E-02	5.17E-02
GMPR	-0.33837	2.92E-15	2.17E-14
GMPS	0.660165	9.23E-66	9.17E-64
GNA11	-0.04101	3.53E-01	4.19E-01
GNA12	0.238717	4.17E-08	1.48E-07
GNA13	0.429736	1.47E-24	2.50E-23
GNA14	-0.38726	7.15E-20	8.23E-19
GNA15	-0.00378	9.32E-01	9.46E-01
GNAI1	-0.07461	9.07E-02	1.26E-01
GNAI2	-0.10544	1.67E-02	2.71E-02
GNAI3	0.444918	2.10E-26	4.14E-25
GNAL	-0.07317	9.72E-02	1.34E-01
GNAO1	-0.19896	5.37E-06	1.49E-05
GNAQ	-0.35832	4.78E-17	4.29E-16
GNASAS	0.041482	3.47E-01	4.13E-01
GNAS	0.005328	9.04E-01	9.23E-01
GNAT1	0.236552	5.56E-08	1.95E-07
GNAT2	0.141221	1.31E-03	2.59E-03
GNAT3	-0.01873	6.71E-01	7.23E-01
GNAZ	-0.07279	9.89E-02	1.36E-01
GNB1L	0.23074	1.19E-07	4.03E-07
GNB1	0.244768	1.83E-08	6.78E-08
GNB2L1	-0.13288	2.51E-03	4.73E-03
GNB2	0.100532	2.25E-02	3.57E-02
GNB3	-0.04378	3.21E-01	3.86E-01
GNB4	0.30978	6.45E-13	3.87E-12

GNB5	0.030903	4.84E-01	5.49E-01
GNE	0.039409	3.72E-01	4.38E-01
GNG10	0.156424	3.66E-04	7.86E-04
GNG11	-0.11768	7.51E-03	1.30E-02
GNG12	-0.01553	7.25E-01	7.71E-01
GNG13	-0.02868	5.16E-01	5.80E-01
GNG2	-0.17953	4.17E-05	1.03E-04
GNG3	0.096786	2.81E-02	4.36E-02
GNG4	0.466357	3.59E-29	8.73E-28
GNG5	0.093158	3.46E-02	5.27E-02
GNG7	-0.56496	9.25E-45	5.35E-43
GNG8	-0.07058	1.10E-01	1.49E-01
GNGT1	0.120027	6.39E-03	1.12E-02
GNGT2	-0.06745	1.26E-01	1.70E-01
GNL1	0.048973	2.67E-01	3.29E-01
GNL2	0.240882	3.11E-08	1.12E-07
GNL3L	0.131068	2.88E-03	5.38E-03
GNL3	0.282264	6.89E-11	3.32E-10
GNLY	0.170626	9.97E-05	2.33E-04
GNMT	-0.50449	1.32E-34	4.45E-33
GNPAT	-0.05366	2.24E-01	2.82E-01
GNPDA1	0.216182	7.32E-07	2.26E-06
GNPDA2	0.05449	2.17E-01	2.74E-01
GNPNAT1	0.50848	3.26E-35	1.13E-33
GNPTAB	-0.24909	1.01E-08	3.84E-08
GNPTG	-0.3948	1.18E-20	1.46E-19
GNRH1	-0.25404	5.00E-09	1.98E-08
GNRH2	0.036019	4.15E-01	4.81E-01
GNRHR2	-0.45122	3.39E-27	7.12E-26
GNRHR	-0.22508	2.45E-07	8.01E-07
GNS	-0.07159	1.05E-01	1.43E-01
GOLGA1	-0.07863	7.46E-02	1.06E-01
GOLGA2B	-0.29434	9.44E-12	4.99E-11
GOLGA2P3	-0.084	5.68E-02	8.28E-02
GOLGA2	-0.11971	6.53E-03	1.15E-02
GOLGA3	-0.00909	8.37E-01	8.66E-01
GOLGA4	-0.10848	1.38E-02	2.28E-02
GOLGA5	0.184917	2.41E-05	6.14E-05
GOLGA6A	0.051508	2.43E-01	3.03E-01
GOLGA6B	0.01196	7.87E-01	8.23E-01
GOLGA6C	0.129747	3.18E-03	5.90E-03
GOLGA6D	0.106696	1.54E-02	2.53E-02
GOLGA6L10	-0.3712	2.87E-18	2.86E-17
GOLGA6L1	-0.03708	4.01E-01	4.68E-01

GOLGA6L5	-0.31974	1.05E-13	6.78E-13
GOLGA6L6	-0.06496	1.41E-01	1.87E-01
GOLGA6L9	-0.36895	4.72E-18	4.61E-17
GOLGA7B	-0.11893	6.89E-03	1.20E-02
GOLGA7	0.214042	9.46E-07	2.87E-06
GOLGA8A	-0.25165	7.02E-09	2.73E-08
GOLGA8B	-0.2603	2.02E-09	8.32E-09
GOLGA8C	0.16174	2.28E-04	5.06E-04
GOLGA8DP	0.050286	2.55E-01	3.15E-01
GOLGA8E	0.128531	3.48E-03	6.41E-03
GOLGA8F	0.070824	1.08E-01	1.48E-01
GOLGA8G	0.092021	3.68E-02	5.58E-02
GOLGA9P	-0.12801	3.62E-03	6.64E-03
GOLGB1	-0.16765	1.32E-04	3.03E-04
GOLIM4	0.103694	1.86E-02	3.00E-02
GOLM1	0.15726	3.40E-04	7.34E-04
GOLPH3L	-0.26738	7.02E-10	3.06E-09
GOLPH3	-0.01543	7.27E-01	7.72E-01
GOLT1A	-0.15813	3.15E-04	6.83E-04
GOLT1B	0.493916	4.99E-33	1.54E-31
GON4L	-0.07087	1.08E-01	1.48E-01
GOPC	0.148675	7.13E-04	1.47E-03
GORAB	-0.24261	2.47E-08	8.98E-08
GORASP1	-0.37314	1.85E-18	1.88E-17
GORASP2	0.116667	8.04E-03	1.39E-02
GOSR1	0.217791	6.02E-07	1.88E-06
GOSR2	0.248313	1.12E-08	4.24E-08
GOT1L1	0.062435	1.57E-01	2.06E-01
GOT1	0.241002	3.06E-08	1.11E-07
GOT2	0.204155	3.00E-06	8.58E-06
GP1BA	-0.24106	3.04E-08	1.10E-07
GP2	0.034522	4.34E-01	5.01E-01
GP5	-0.12282	5.26E-03	9.39E-03
GP6	-0.12311	5.15E-03	9.22E-03
GP9	-0.32427	4.50E-14	3.01E-13
GPA33	-0.15368	4.66E-04	9.82E-04
GPAA1	-0.08034	6.85E-02	9.83E-02
GPAM	-0.16654	1.47E-04	3.34E-04
GPAT2	-0.00489	9.12E-01	9.29E-01
GPATCH1	0.165665	1.59E-04	3.60E-04
GPATCH2	0.199237	5.21E-06	1.45E-05
GPATCH3	-0.04502	3.08E-01	3.72E-01
GPATCH4	0.113314	1.01E-02	1.71E-02
GPATCH8	0.051531	2.43E-01	3.03E-01

GPBAR1	-0.10633	1.58E-02	2.58E-02
GPBP1L1	-0.26316	1.32E-09	5.58E-09
GPBP1	-0.0991	2.45E-02	3.86E-02
GPC1	0.002412	9.56E-01	9.65E-01
GPC2	0.321021	8.27E-14	5.40E-13
GPC3	-0.25407	4.98E-09	1.97E-08
GPC4	-0.39017	3.58E-20	4.25E-19
GPC5	-0.29367	1.05E-11	5.55E-11
GPC6	0.415541	6.43E-23	9.53E-22
GPCPD1	-0.03082	4.85E-01	5.50E-01
GPD1L	-0.54787	1.12E-41	5.56E-40
GPD1	-0.29301	1.18E-11	6.16E-11
GPD2	0.343265	1.09E-15	8.51E-15
GPER	-0.21094	1.37E-06	4.07E-06
GPHA2	-0.13525	2.10E-03	4.00E-03
GPHN	-0.04942	2.63E-01	3.24E-01
GPIHBP1	-0.36851	5.21E-18	5.05E-17
GPI	0.492123	9.12E-33	2.77E-31
GPKOW	0.166237	1.51E-04	3.42E-04
GPLD1	-0.30061	3.23E-12	1.80E-11
GPM6A	-0.21815	5.77E-07	1.80E-06
GPM6B	-0.27793	1.37E-10	6.46E-10
GPN1	0.57255	3.45E-46	2.10E-44
GPN2	0.04043	3.60E-01	4.26E-01
GPN3	0.385661	1.04E-19	1.18E-18
GPNMB	0.007326	8.68E-01	8.93E-01
GPR101	0.035592	4.20E-01	4.86E-01
GPR107	-0.03848	3.84E-01	4.50E-01
GPR108	-0.37848	5.51E-19	5.85E-18
GPR109A	-0.04223	3.39E-01	4.04E-01
GPR109B	0.041616	3.46E-01	4.12E-01
GPR110	-0.03433	4.37E-01	5.04E-01
GPR111	0.174531	6.84E-05	1.63E-04
GPR112	-0.25661	3.45E-09	1.39E-08
GPR113	0.035842	4.17E-01	4.83E-01
GPR114	-0.08743	4.74E-02	7.02E-02
GPR115	0.4808	3.78E-31	1.05E-29
GPR116	-0.58568	9.53E-49	6.26E-47
GPR119	0.139664	1.49E-03	2.91E-03
GPR120	-0.14045	1.40E-03	2.74E-03
GPR123	-0.2633	1.29E-09	5.46E-09
GPR124	-0.16319	2.00E-04	4.46E-04
GPR125	-0.02763	5.32E-01	5.95E-01
GPR126	0.047148	2.86E-01	3.48E-01

GPR128	0.116883	7.93E-03	1.37E-02
GPR12	-0.27256	3.18E-10	1.44E-09
GPR132	-0.17542	6.27E-05	1.51E-04
GPR133	-0.56431	1.22E-44	6.97E-43
GPR135	-0.07323	9.69E-02	1.34E-01
GPR137B	0.143815	1.06E-03	2.13E-03
GPR137C	0.273186	2.88E-10	1.32E-09
GPR137	-0.08107	6.60E-02	9.51E-02
GPR139	0.095084	3.10E-02	4.77E-02
GPR141	0.076552	8.26E-02	1.16E-01
GPR142	0.031989	4.69E-01	5.35E-01
GPR143	-0.13568	2.03E-03	3.88E-03
GPR144	0.10198	2.06E-02	3.29E-02
GPR146	-0.44697	1.16E-26	2.33E-25
GPR148	-0.01757	6.91E-01	7.40E-01
GPR149	-0.00979	8.25E-01	8.55E-01
GPR150	0.048312	2.74E-01	3.36E-01
GPR151	-0.05749	1.93E-01	2.47E-01
GPR152	0.071786	1.04E-01	1.42E-01
GPR153	-0.05535	2.10E-01	2.66E-01
GPR155	-0.06269	1.55E-01	2.04E-01
GPR156	0.157207	3.42E-04	7.37E-04
GPR157	0.13736	1.78E-03	3.44E-03
GPR158	0.202632	3.56E-06	1.01E-05
GPR15	0.134576	2.21E-03	4.20E-03
GPR160	-0.35226	1.72E-16	1.45E-15
GPR161	0.20459	2.85E-06	8.18E-06
GPR162	-0.25706	3.23E-09	1.30E-08
GPR171	-0.12208	5.54E-03	9.86E-03
GPR172A	0.186432	2.06E-05	5.29E-05
GPR172B	-0.30683	1.09E-12	6.36E-12
GPR173	-0.02223	6.15E-01	6.71E-01
GPR174	-0.04116	3.51E-01	4.17E-01
GPR176	0.203008	3.41E-06	9.72E-06
GPR179	-0.08541	5.27E-02	7.74E-02
GPR17	-0.31346	3.33E-13	2.05E-12
GPR180	0.415748	6.09E-23	9.07E-22
GPR182	-0.17476	6.69E-05	1.60E-04
GPR183	-0.04776	2.79E-01	3.41E-01
GPR18	-0.07303	9.78E-02	1.35E-01
GPR19	0.633274	4.44E-59	3.74E-57
GPR1	0.235698	6.22E-08	2.17E-07
GPR20	-0.29327	1.13E-11	5.92E-11
GPR21	-0.05508	2.12E-01	2.68E-01

GPR22	0.080003	6.97E-02	9.98E-02
GPR25	-0.13011	3.10E-03	5.75E-03
GPR26	0.059952	1.74E-01	2.26E-01
GPR27	-0.04866	2.70E-01	3.32E-01
GPR31	-0.07844	7.53E-02	1.07E-01
GPR32	0.106232	1.59E-02	2.60E-02
GPR34	-0.13824	1.66E-03	3.23E-03
GPR35	0.151996	5.38E-04	1.12E-03
GPR37L1	0.308576	8.00E-13	4.75E-12
GPR37	0.252293	6.41E-09	2.50E-08
GPR39	-0.3298	1.56E-14	1.09E-13
GPR3	0.194042	9.21E-06	2.48E-05
GPR44	-0.4578	4.82E-28	1.08E-26
GPR45	0.044813	3.10E-01	3.74E-01
GPR4	0.070276	1.11E-01	1.51E-01
GPR50	0.125921	4.21E-03	7.65E-03
GPR52	0.016001	7.17E-01	7.64E-01
GPR55	-0.21169	1.25E-06	3.74E-06
GPR56	-0.12437	4.71E-03	8.49E-03
GPR61	0.093246	3.44E-02	5.24E-02
GPR62	-0.05138	2.44E-01	3.05E-01
GPR63	0.212348	1.16E-06	3.48E-06
GPR64	-0.23892	4.05E-08	1.44E-07
GPR65	-0.05674	1.99E-01	2.53E-01
GPR68	-0.05899	1.81E-01	2.34E-01
GPR6	0.078974	7.34E-02	1.05E-01
GPR75	-0.04878	2.69E-01	3.31E-01
GPR77	-0.13955	1.50E-03	2.93E-03
GPR78	0.223997	2.80E-07	9.11E-07
GPR81	-0.03141	4.77E-01	5.42E-01
GPR82	-0.06848	1.21E-01	1.63E-01
GPR83	0.064273	1.45E-01	1.92E-01
GPR84	0.203312	3.30E-06	9.40E-06
GPR85	-0.15784	3.23E-04	7.00E-04
GPR87	0.245252	1.72E-08	6.36E-08
GPR88	-0.04847	2.72E-01	3.34E-01
GPR89A	0.150843	5.94E-04	1.23E-03
GPR89B	0.059384	1.78E-01	2.31E-01
GPR89C	0.040396	3.60E-01	4.26E-01
GPR97	0.333492	7.64E-15	5.48E-14
GPR98	-0.30978	6.46E-13	3.87E-12
GPRASP1	-0.44806	8.49E-27	1.72E-25
GPRASP2	-0.26612	8.49E-10	3.66E-09
GPRC5A	-0.2406	3.23E-08	1.16E-07

GPRC5B	-0.02876	5.15E-01	5.79E-01
GPRC5C	-0.47584	1.85E-30	4.89E-29
GPRC5D	-0.06752	1.26E-01	1.69E-01
GPRC6A	0.030117	4.95E-01	5.60E-01
GPRIN1	0.451007	3.60E-27	7.53E-26
GPRIN2	-0.20802	1.92E-06	5.63E-06
GPRIN3	0.05771	1.91E-01	2.45E-01
GPS1	0.238551	4.26E-08	1.51E-07
GPS2	-0.08076	6.71E-02	9.65E-02
GPSM1	-0.04241	3.37E-01	4.02E-01
GPSM2	0.462944	1.02E-28	2.40E-27
GPSM3	-0.32765	2.37E-14	1.62E-13
GPT2	0.026796	5.44E-01	6.06E-01
GPT	-0.13887	1.58E-03	3.08E-03
GPX1	-0.01215	7.83E-01	8.20E-01
GPX2	0.003513	9.37E-01	9.50E-01
GPX3	-0.25535	4.14E-09	1.65E-08
GPX4	-0.11862	7.04E-03	1.23E-02
GPX5	-0.04305	3.30E-01	3.95E-01
GPX6	0.046976	2.87E-01	3.50E-01
GPX7	0.193689	9.56E-06	2.57E-05
GPX8	0.330572	1.35E-14	9.45E-14
GRAMD1A	-0.05557	2.08E-01	2.64E-01
GRAMD1B	0.148956	6.96E-04	1.43E-03
GRAMD1C	-0.03346	4.49E-01	5.15E-01
GRAMD2	-0.46386	7.71E-29	1.83E-27
GRAMD3	-0.22386	2.85E-07	9.26E-07
GRAMD4	-0.19808	5.92E-06	1.63E-05
GRAP2	-0.1149	9.06E-03	1.55E-02
GRAPL	-0.20935	1.65E-06	4.86E-06
GRAP	-0.18091	3.63E-05	9.01E-05
GRASP	-0.27153	3.73E-10	1.68E-09
GRB10	0.110692	1.19E-02	2.00E-02
GRB14	-0.0346	4.33E-01	5.00E-01
GRB2	0.215926	7.55E-07	2.32E-06
GRB7	-0.11167	1.12E-02	1.89E-02
GREB1L	0.416245	5.35E-23	8.01E-22
GREB1	-0.26389	1.18E-09	5.02E-09
GREM1	0.429584	1.53E-24	2.60E-23
GREM2	-0.23444	7.34E-08	2.54E-07
GRHL1	0.019144	6.65E-01	7.17E-01
GRHL2	-0.09491	3.13E-02	4.81E-02
GRHL3	0.04637	2.94E-01	3.56E-01
GRHPR	0.07205	1.02E-01	1.41E-01

GRIA1	-0.48979	1.99E-32	5.95E-31
GRIA2	0.113511	9.94E-03	1.69E-02
GRIA3	-0.09934	2.42E-02	3.81E-02
GRIA4	-0.04152	3.47E-01	4.13E-01
GRID1	-0.3982	5.13E-21	6.56E-20
GRID2IP	-0.258	2.82E-09	1.15E-08
GRID2	0.06463	1.43E-01	1.89E-01
GRIK1	0.146795	8.34E-04	1.70E-03
GRIK2	-0.04114	3.51E-01	4.18E-01
GRIK3	-0.19609	7.37E-06	2.01E-05
GRIK4	-0.24808	1.16E-08	4.38E-08
GRIK5	0.136436	1.91E-03	3.68E-03
GRIN1	0.06226	1.58E-01	2.07E-01
GRIN2A	-0.28685	3.27E-11	1.63E-10
GRIN2B	0.157658	3.29E-04	7.10E-04
GRIN2C	-0.03389	4.43E-01	5.09E-01
GRIN2D	0.273171	2.89E-10	1.32E-09
GRIN3A	0.100641	2.24E-02	3.55E-02
GRIN3B	-0.0327	4.59E-01	5.25E-01
GRINA	-0.09027	4.06E-02	6.10E-02
GRINL1A	0.081384	6.50E-02	9.37E-02
GRIP1	-0.03344	4.49E-01	5.15E-01
GRIP2	0.097891	2.63E-02	4.11E-02
GRIPAP1	-0.05599	2.05E-01	2.60E-01
GRK1	-0.16835	1.24E-04	2.85E-04
GRK4	0.120533	6.17E-03	1.09E-02
GRK5	-0.1463	8.69E-04	1.76E-03
GRK6	0.080096	6.93E-02	9.94E-02
GRK7	0.111182	1.16E-02	1.94E-02
GRLF1	-0.17391	7.27E-05	1.73E-04
GRM1	0.040748	3.56E-01	4.22E-01
GRM2	0.094804	3.15E-02	4.84E-02
GRM3	-0.18477	2.45E-05	6.22E-05
GRM4	0.133635	2.37E-03	4.49E-03
GRM5	0.200796	4.38E-06	1.23E-05
GRM6	-0.306	1.26E-12	7.33E-12
GRM7	-0.26846	5.96E-10	2.62E-09
GRM8	0.218899	5.26E-07	1.65E-06
GRN	-0.19081	1.30E-05	3.43E-05
GRPEL1	0.258695	2.55E-09	1.04E-08
GRPEL2	0.272452	3.23E-10	1.47E-09
GRPR	0.146422	8.60E-04	1.74E-03
GRP	-0.11947	6.64E-03	1.16E-02
GRRP1	-0.30658	1.14E-12	6.64E-12

GRSF1	0.231524	1.07E-07	3.66E-07
GRTP1	-0.13521	2.11E-03	4.01E-03
GRWD1	0.200398	4.58E-06	1.28E-05
GRXCR1	-0.03089	4.84E-01	5.49E-01
GRXCR2	0.013103	7.67E-01	8.06E-01
GSC2	0.02685	5.43E-01	6.05E-01
GSC	0.055869	2.06E-01	2.61E-01
GSDMA	0.004873	9.12E-01	9.30E-01
GSDMB	-0.13311	2.47E-03	4.66E-03
GSDMC	0.075737	8.60E-02	1.20E-01
GSDMD	-0.12738	3.79E-03	6.93E-03
GSG1L	-0.25089	7.82E-09	3.02E-08
GSG1	0.117508	7.60E-03	1.32E-02
GSG2	0.754831	5.16E-96	7.75E-94
GSK3A	0.2475	1.26E-08	4.73E-08
GSK3B	0.204758	2.80E-06	8.03E-06
GSN	-0.36184	2.24E-17	2.08E-16
GSPT1	-0.17613	5.85E-05	1.41E-04
GSPT2	-0.22182	3.67E-07	1.18E-06
GSR	0.166714	1.44E-04	3.28E-04
GSS	0.365444	1.02E-17	9.72E-17
GSTA1	-0.25251	6.22E-09	2.43E-08
GSTA2	-0.1771	5.32E-05	1.29E-04
GSTA3	-0.24809	1.16E-08	4.38E-08
GSTA4	-0.21938	4.96E-07	1.56E-06
GSTA5	-0.1893	1.53E-05	3.99E-05
GSTCD	0.41232	1.48E-22	2.13E-21
GSTK1	-0.0426	3.35E-01	4.00E-01
GSTM1	-0.02563	5.62E-01	6.22E-01
GSTM2P1	-0.06765	1.25E-01	1.69E-01
GSTM2	-0.21163	1.26E-06	3.77E-06
GSTM3	0.021457	6.27E-01	6.83E-01
GSTM4	-0.11012	1.24E-02	2.07E-02
GSTM5	-0.44878	6.89E-27	1.40E-25
GSTO1	0.216125	7.37E-07	2.27E-06
GSTO2	0.005067	9.09E-01	9.27E-01
GSTP1	-0.08835	4.51E-02	6.71E-02
GSTT1	-0.04725	2.85E-01	3.47E-01
GSTT2	-0.04845	2.72E-01	3.34E-01
GSTTP1	-0.0087	8.44E-01	8.72E-01
GSTTP2	0.012802	7.72E-01	8.10E-01
GSTZ1	0.043252	3.27E-01	3.93E-01
GSX2	0.099795	2.35E-02	3.71E-02
GTDC1	0.193035	1.03E-05	2.74E-05

GTF2A1L	0.063192	1.52E-01	2.00E-01
GTF2A1	0.181265	3.51E-05	8.70E-05
GTF2A2	0.235208	6.64E-08	2.31E-07
GTF2B	0.152859	5.00E-04	1.05E-03
GTF2E1	0.439278	1.04E-25	1.95E-24
GTF2E2	0.31659	1.88E-13	1.19E-12
GTF2F1	-0.0186	6.74E-01	7.25E-01
GTF2F2	0.191191	1.25E-05	3.30E-05
GTF2H1	0.154065	4.50E-04	9.51E-04
GTF2H2B	-0.00083	9.85E-01	9.88E-01
GTF2H2C	-0.03114	4.81E-01	5.46E-01
GTF2H2	0.132548	2.58E-03	4.85E-03
GTF2H3	0.384872	1.25E-19	1.41E-18
GTF2H4	0.220182	4.50E-07	1.42E-06
GTF2H5	0.039106	3.76E-01	4.42E-01
GTF2IP1	-0.27172	3.62E-10	1.63E-09
GTF2IRD1	0.186242	2.10E-05	5.39E-05
GTF2IRD2B	-0.47513	2.32E-30	6.10E-29
GTF2IRD2P1	-0.54634	2.07E-41	1.01E-39
GTF2IRD2	-0.51826	9.69E-37	3.68E-35
GTF2I	-0.06002	1.74E-01	2.26E-01
GTF3A	0.245896	1.57E-08	5.85E-08
GTF3C1	-0.02139	6.28E-01	6.84E-01
GTF3C2	0.386015	9.58E-20	1.09E-18
GTF3C3	0.443278	3.36E-26	6.56E-25
GTF3C4	0.10324	1.91E-02	3.07E-02
GTF3C5	0.250163	8.66E-09	3.33E-08
GTF3C6	0.281799	7.42E-11	3.57E-10
GTPBP10	0.312561	3.91E-13	2.40E-12
GTPBP1	-0.19826	5.80E-06	1.60E-05
GTPBP2	0.176857	5.45E-05	1.32E-04
GTPBP3	0.097983	2.62E-02	4.09E-02
GTPBP4	0.378554	5.42E-19	5.75E-18
GTPBP5	-0.02348	5.95E-01	6.53E-01
GTPBP8	0.347321	4.78E-16	3.87E-15
GTSE1	0.863002	3.07E-154	2.12E-151
GTSF1L	0.042644	3.34E-01	4.00E-01
GTSF1	0.153293	4.81E-04	1.01E-03
GUCA1A	0.446742	1.24E-26	2.48E-25
GUCA1B	0.12038	6.23E-03	1.10E-02
GUCA1C	0.067198	1.28E-01	1.72E-01
GUCA2A	-0.07278	9.90E-02	1.36E-01
GUCA2B	0.111791	1.11E-02	1.87E-02
GUCY1A2	-0.22226	3.48E-07	1.12E-06

GUCY1A3	-0.34415	9.14E-16	7.20E-15
GUCY1B2	-0.22657	2.03E-07	6.69E-07
GUCY1B3	-0.14768	7.74E-04	1.58E-03
GUCY2C	0.095944	2.95E-02	4.56E-02
GUCY2D	-0.28376	5.41E-11	2.63E-10
GUCY2E	0.02581	5.59E-01	6.20E-01
GUCY2F	-0.15782	3.24E-04	7.01E-04
GUCY2GP	-0.07488	8.96E-02	1.25E-01
GUF1	0.171955	8.78E-05	2.06E-04
GUK1	-0.02167	6.24E-01	6.80E-01
GULP1	0.049824	2.59E-01	3.20E-01
GUSBL1	-0.09604	2.93E-02	4.54E-02
GUSBL2	-0.08226	6.21E-02	8.99E-02
GUSBP1	-0.02955	5.03E-01	5.68E-01
GUSBP3	-0.04761	2.81E-01	3.43E-01
GUSB	-0.1433	1.11E-03	2.21E-03
GVIN1	-0.16106	2.43E-04	5.35E-04
GXYLT1	0.114022	9.61E-03	1.64E-02
GXYLT2	0.020895	6.36E-01	6.91E-01
GYG1	0.342101	1.38E-15	1.06E-14
GYG2	-0.12909	3.34E-03	6.17E-03
GYLTL1B	-0.10499	1.72E-02	2.79E-02
GYPA	-0.09122	3.85E-02	5.81E-02
GYPB	-0.17957	4.16E-05	1.02E-04
GYPC	-0.24355	2.17E-08	7.94E-08
GYPE	-0.26594	8.72E-10	3.75E-09
GYS1	0.033441	4.49E-01	5.15E-01
GYS2	0.118928	6.89E-03	1.20E-02
GZF1	-0.07749	7.89E-02	1.12E-01
GZMA	0.178106	4.81E-05	1.17E-04
GZMB	0.385123	1.18E-19	1.33E-18
GZMH	0.125948	4.20E-03	7.64E-03
GZMK	-0.02039	6.44E-01	6.99E-01
GZMM	0.00501	9.10E-01	9.28E-01
H19	0.148981	6.95E-04	1.43E-03
H1F0	0.147128	8.11E-04	1.65E-03
H1FNT	-0.30783	9.14E-13	5.39E-12
H1FOO	0.040454	3.60E-01	4.26E-01
H1FX	0.129986	3.12E-03	5.80E-03
H2AFB1	0.102459	2.00E-02	3.21E-02
H2AFJ	0.135	2.14E-03	4.07E-03
H2AFV	0.291904	1.42E-11	7.36E-11
H2AFX	0.618086	1.37E-55	1.05E-53
H2AFY2	0.049355	2.64E-01	3.25E-01

H2AFY	0.308382	8.28E-13	4.91E-12
H2AFZ	0.725516	2.72E-85	3.46E-83
H2BFM	0.050963	2.48E-01	3.09E-01
H2BFWT	0.113758	9.77E-03	1.66E-02
H2BFXP	0.04761	2.81E-01	3.43E-01
H3F3A	-0.08997	4.13E-02	6.19E-02
H3F3B	0.088203	4.54E-02	6.76E-02
H3F3C	0.085361	5.29E-02	7.75E-02
H6PD	-0.26985	4.82E-10	2.14E-09
HAAO	-0.16754	1.33E-04	3.05E-04
HABP2	-0.36245	1.97E-17	1.83E-16
HABP4	-0.14378	1.07E-03	2.13E-03
HACE1	0.077458	7.91E-02	1.12E-01
HACL1	-0.0279	5.28E-01	5.91E-01
HADHA	0.182749	3.01E-05	7.55E-05
HADHB	0.040395	3.60E-01	4.26E-01
HADH	-0.24841	1.11E-08	4.19E-08
HAGHL	-0.12141	5.80E-03	1.03E-02
HAGH	-0.39793	5.49E-21	6.98E-20
HAL	0.03267	4.59E-01	5.26E-01
HAMP	0.119905	6.44E-03	1.13E-02
HAND1	-0.16129	2.37E-04	5.24E-04
HAND2	0.01268	7.74E-01	8.12E-01
HAO1	-0.03467	4.32E-01	4.99E-01
HAO2	-0.07154	1.05E-01	1.44E-01
HAP1	0.163336	1.97E-04	4.41E-04
HAPLN1	0.11894	6.89E-03	1.20E-02
HAPLN2	-0.02745	5.34E-01	5.97E-01
HAPLN3	0.297454	5.56E-12	3.02E-11
HAPLN4	-0.01802	6.83E-01	7.33E-01
HAR1A	-0.22492	2.50E-07	8.16E-07
HAR1B	-0.06837	1.21E-01	1.64E-01
HARBI1	0.162559	2.12E-04	4.71E-04
HARS2	-0.01275	7.73E-01	8.11E-01
HARS	0.058741	1.83E-01	2.36E-01
HAS1	0.058034	1.89E-01	2.42E-01
HAS2AS	0.079178	7.26E-02	1.04E-01
HAS2	0.012717	7.73E-01	8.12E-01
HAS3	-0.36656	8.00E-18	7.67E-17
HAT1	0.531438	7.06E-39	3.06E-37
HAUS1	0.411372	1.89E-22	2.69E-21
HAUS2	0.422697	9.78E-24	1.57E-22
HAUS3	0.168415	1.23E-04	2.83E-04
HAUS4	-0.12998	3.13E-03	5.80E-03

HAUS5	0.257078	3.23E-09	1.30E-08
HAUS6	0.533794	2.86E-39	1.27E-37
HAUS7	0.311041	5.15E-13	3.12E-12
HAUS8	0.390429	3.36E-20	4.00E-19
HAVCR1	-0.01419	7.48E-01	7.90E-01
HAVCR2	0.076375	8.34E-02	1.17E-01
HAX1	0.195267	8.06E-06	2.19E-05
HBA1	-0.2189	5.26E-07	1.65E-06
HBA2	-0.24925	9.85E-09	3.76E-08
HBBP1	0.099689	2.37E-02	3.74E-02
HBB	-0.21152	1.28E-06	3.81E-06
HBD	0.027655	5.31E-01	5.94E-01
HBE1	0.179955	4.00E-05	9.85E-05
HBEGF	-0.06947	1.15E-01	1.56E-01
HBG1	-0.03127	4.79E-01	5.44E-01
HBG2	-0.00502	9.09E-01	9.27E-01
HBM	-0.10356	1.87E-02	3.02E-02
HBP1	-0.21908	5.14E-07	1.62E-06
HBQ1	0.266769	7.70E-10	3.34E-09
HBS1L	0.387783	6.31E-20	7.31E-19
HBXIP	0.015811	7.20E-01	7.67E-01
HBZ	-0.18898	1.58E-05	4.13E-05
HCCS	0.365397	1.03E-17	9.81E-17
HCFC1R1	-0.1754	6.29E-05	1.51E-04
HCFC1	0.116487	8.14E-03	1.40E-02
HCFC2	-0.06275	1.55E-01	2.04E-01
HCG11	-0.07854	7.49E-02	1.07E-01
HCG18	0.006242	8.88E-01	9.09E-01
HCG22	0.016567	7.08E-01	7.55E-01
HCG26	-0.17363	7.47E-05	1.77E-04
HCG27	-0.19616	7.31E-06	1.99E-05
HCG2P7	-0.32669	2.84E-14	1.94E-13
HCG4P6	-0.15506	4.13E-04	8.78E-04
HCG4	-0.03678	4.05E-01	4.71E-01
HCG9	-0.00124	9.78E-01	9.83E-01
HCK	-0.04244	3.36E-01	4.02E-01
HCLS1	-0.10257	1.99E-02	3.19E-02
HCN1	-0.26279	1.40E-09	5.88E-09
HCN2	0.236172	5.84E-08	2.04E-07
HCN3	0.109298	1.31E-02	2.17E-02
HCN4	-0.30229	2.42E-12	1.37E-11
HCP5	0.105133	1.70E-02	2.76E-02
HCRTR1	-0.08021	6.89E-02	9.89E-02
HCRTR2	-0.10099	2.19E-02	3.48E-02

HCRT	0.047147	2.86E-01	3.48E-01
HCST	-0.06416	1.46E-01	1.93E-01
HDAC10	-0.27225	3.33E-10	1.51E-09
HDAC11	-0.38537	1.11E-19	1.26E-18
HDAC1	0.289732	2.04E-11	1.04E-10
HDAC2	0.529839	1.30E-38	5.51E-37
HDAC3	-0.04436	3.15E-01	3.79E-01
HDAC4	-0.03017	4.94E-01	5.59E-01
HDAC5	-0.3921	2.26E-20	2.73E-19
HDAC6	-0.13709	1.82E-03	3.51E-03
HDAC7	-0.2152	8.24E-07	2.52E-06
HDAC8	-0.01934	6.62E-01	7.14E-01
HDAC9	0.035726	4.18E-01	4.85E-01
HDC	-0.31118	5.02E-13	3.04E-12
HDDC2	0.15459	4.30E-04	9.12E-04
HDDC3	0.019106	6.65E-01	7.17E-01
HDGFL1	0.075858	8.55E-02	1.20E-01
HDGFRP2	0.087976	4.60E-02	6.84E-02
HDGFRP3	0.221363	3.89E-07	1.24E-06
HDGF	0.392046	2.28E-20	2.76E-19
HDHD1A	-0.02815	5.24E-01	5.88E-01
HDHD2	-0.34419	9.08E-16	7.15E-15
HDHD3	-0.04564	3.01E-01	3.65E-01
HDLBP	0.089188	4.31E-02	6.43E-02
HDX	0.131671	2.75E-03	5.16E-03
HEATR1	0.29791	5.14E-12	2.80E-11
HEATR2	0.350479	2.49E-16	2.07E-15
HEATR3	0.137735	1.73E-03	3.35E-03
HEATR4	0.019713	6.55E-01	7.08E-01
HEATR5A	0.074711	9.03E-02	1.26E-01
HEATR5B	-0.11462	9.23E-03	1.58E-02
HEATR6	0.163316	1.97E-04	4.41E-04
HEATR7A	-0.17465	6.76E-05	1.62E-04
HEATR7B2	0.044226	3.16E-01	3.81E-01
HEBP1	-0.15338	4.78E-04	1.01E-03
HEBP2	0.268983	5.50E-10	2.43E-09
HECA	-0.1598	2.71E-04	5.94E-04
HECTD1	-0.10367	1.86E-02	3.00E-02
HECTD2	0.020929	6.36E-01	6.91E-01
HECTD3	-0.14666	8.43E-04	1.71E-03
HECW1	-0.01283	7.71E-01	8.10E-01
HECW2	-0.26471	1.05E-09	4.48E-09
HEG1	-0.12284	5.25E-03	9.38E-03
HELB	0.257406	3.08E-09	1.24E-08

HELLS	0.782606	1.13E-107	2.07E-105
HELQ	-0.19779	6.11E-06	1.68E-05
HELT	-0.07359	9.53E-02	1.32E-01
HELZ	-0.12948	3.24E-03	6.01E-03
HEMGN	-0.23481	7.00E-08	2.43E-07
HEMK1	-0.43153	8.99E-25	1.56E-23
HEPACAM2	0.123736	4.92E-03	8.86E-03
HEPACAM	-0.19125	1.24E-05	3.29E-05
HEPHL1	0.112876	1.04E-02	1.75E-02
HEPH	-0.0813	6.52E-02	9.41E-02
HEPN1	-0.18272	3.02E-05	7.57E-05
HERC1	-0.33003	1.50E-14	1.04E-13
HERC2P2	-0.1911	1.26E-05	3.33E-05
HERC2P4	0.121352	5.83E-03	1.03E-02
HERC2	-0.07247	1.00E-01	1.38E-01
HERC3	-0.17489	6.61E-05	1.58E-04
HERC4	-0.01289	7.70E-01	8.09E-01
HERC5	0.155728	3.89E-04	8.32E-04
HERC6	-0.04582	2.99E-01	3.63E-01
HERPUD1	-0.30956	6.71E-13	4.02E-12
HERPUD2	0.115783	8.54E-03	1.46E-02
HES1	0.12325	5.10E-03	9.14E-03
HES2	0.122783	5.27E-03	9.41E-03
HES3	0.078885	7.37E-02	1.05E-01
HES4	0.22341	3.01E-07	9.76E-07
HES5	-0.21998	4.61E-07	1.46E-06
HES6	0.175223	6.39E-05	1.53E-04
HES7	0.16179	2.27E-04	5.03E-04
HESRG	0.014221	7.47E-01	7.90E-01
HESX1	-0.03346	4.49E-01	5.15E-01
HEXA	-0.18125	3.51E-05	8.71E-05
HEXB	-0.06546	1.38E-01	1.84E-01
HEXDC	-0.26922	5.31E-10	2.34E-09
HEXIM1	0.031772	4.72E-01	5.38E-01
HEXIM2	0.01648	7.09E-01	7.56E-01
HEY1	-0.07037	1.11E-01	1.51E-01
HEY2	-0.11862	7.04E-03	1.23E-02
HEYL	-0.31712	1.71E-13	1.08E-12
HFE2	-0.02201	6.18E-01	6.75E-01
HFE	-0.03349	4.48E-01	5.14E-01
HFM1	-0.07299	9.80E-02	1.35E-01
HGC6.3	-0.03925	3.74E-01	4.40E-01
HGD	0.014578	7.41E-01	7.85E-01
HGFAC	0.005332	9.04E-01	9.22E-01

HGF	-0.19022	1.39E-05	3.64E-05
HGSNAT	-0.28704	3.17E-11	1.58E-10
HGS	0.153031	4.92E-04	1.03E-03
HHATL	-0.16524	1.65E-04	3.73E-04
HHAT	-0.32104	8.25E-14	5.39E-13
HHEX	-0.03112	4.81E-01	5.46E-01
HHIPL1	0.090518	4.00E-02	6.03E-02
HHIPL2	0.221913	3.63E-07	1.16E-06
HHIP	-0.26045	1.97E-09	8.15E-09
HHLA1	0.023276	5.98E-01	6.56E-01
HHLA2	-0.19027	1.38E-05	3.62E-05
HHLA3	-0.0278	5.29E-01	5.92E-01
HIAT1	0.108864	1.34E-02	2.23E-02
HIATL1	0.199393	5.12E-06	1.43E-05
HIATL2	-0.0095	8.30E-01	8.60E-01
HIBADH	0.174918	6.59E-05	1.58E-04
HIBCH	-0.17277	8.12E-05	1.92E-04
HIC1	-0.08734	4.76E-02	7.04E-02
HIC2	0.037898	3.91E-01	4.57E-01
HIF1AN	-0.06418	1.46E-01	1.93E-01
HIF1A	0.40898	3.47E-22	4.86E-21
HIF3A	-0.27576	1.93E-10	8.96E-10
HIGD1A	0.144515	1.01E-03	2.02E-03
HIGD1B	-0.43952	9.73E-26	1.83E-24
HIGD1C	-0.04319	3.28E-01	3.93E-01
HIGD2A	-0.05911	1.80E-01	2.33E-01
HIGD2B	-0.07053	1.10E-01	1.50E-01
HILS1	-0.05051	2.53E-01	3.13E-01
HINFP	0.043358	3.26E-01	3.91E-01
HINT1	0.051796	2.41E-01	3.00E-01
HINT2	-0.04827	2.74E-01	3.36E-01
HINT3	-0.1546	4.30E-04	9.11E-04
HIP1R	-0.08443	5.55E-02	8.11E-02
HIP1	-0.25883	2.50E-09	1.02E-08
HIPK1	-0.15093	5.89E-04	1.23E-03
HIPK2	-0.02414	5.85E-01	6.44E-01
HIPK3	-0.1468	8.33E-04	1.69E-03
HIPK4	0.06924	1.17E-01	1.58E-01
HIRA	0.004679	9.16E-01	9.33E-01
HIRIP3	-0.06497	1.41E-01	1.87E-01
HIST1H1A	0.060747	1.69E-01	2.19E-01
HIST1H1B	0.426752	3.30E-24	5.50E-23
HIST1H1C	0.019802	6.54E-01	7.07E-01
HIST1H1D	0.334836	5.87E-15	4.27E-14

HIST1H1E	0.354715	1.03E-16	8.87E-16
HIST1H1T	0.021768	6.22E-01	6.78E-01
HIST1H2AA	0.041077	3.52E-01	4.18E-01
HIST1H2AB	0.172126	8.64E-05	2.03E-04
HIST1H2AC	-0.05606	2.04E-01	2.60E-01
HIST1H2AD	0.222706	3.29E-07	1.06E-06
HIST1H2AE	0.272963	2.99E-10	1.36E-09
HIST1H2AG	0.37056	3.30E-18	3.28E-17
HIST1H2AH	0.337947	3.18E-15	2.36E-14
HIST1H2AJ	0.338761	2.70E-15	2.02E-14
HIST1H2AK	-0.0153	7.29E-01	7.74E-01
HIST1H2AL	0.371809	2.50E-18	2.51E-17
HIST1H2AM	0.254645	4.58E-09	1.82E-08
HIST1H2BA	0.016052	7.16E-01	7.63E-01
HIST1H2BB	0.139441	1.51E-03	2.95E-03
HIST1H2BC	0.081567	6.44E-02	9.29E-02
HIST1H2BD	0.096184	2.91E-02	4.50E-02
HIST1H2BE	0.119986	6.41E-03	1.13E-02
HIST1H2BF	0.301868	2.60E-12	1.47E-11
HIST1H2BG	0.272601	3.16E-10	1.43E-09
HIST1H2BH	0.35785	5.28E-17	4.72E-16
HIST1H2BI	0.157044	3.47E-04	7.47E-04
HIST1H2BJ	0.386429	8.69E-20	9.94E-19
HIST1H2BK	0.08534	5.29E-02	7.76E-02
HIST1H2BL	0.256585	3.47E-09	1.39E-08
HIST1H2BM	0.150998	5.86E-04	1.22E-03
HIST1H2BN	0.062351	1.58E-01	2.07E-01
HIST1H2BO	0.452483	2.34E-27	4.98E-26
HIST1H3A	0.151461	5.63E-04	1.17E-03
HIST1H3B	0.467612	2.44E-29	6.00E-28
HIST1H3C	0.341408	1.59E-15	1.21E-14
HIST1H3D	0.277833	1.39E-10	6.55E-10
HIST1H3E	0.040987	3.53E-01	4.19E-01
HIST1H3F	0.340109	2.06E-15	1.56E-14
HIST1H3G	0.282193	6.96E-11	3.36E-10
HIST1H3H	0.233107	8.74E-08	3.00E-07
HIST1H3I	0.291444	1.53E-11	7.92E-11
HIST1H3J	0.259193	2.37E-09	9.71E-09
HIST1H4A	0.191145	1.26E-05	3.32E-05
HIST1H4B	0.226462	2.05E-07	6.78E-07
HIST1H4C	0.218087	5.81E-07	1.81E-06
HIST1H4D	0.188435	1.67E-05	4.35E-05
HIST1H4E	0.175784	6.05E-05	1.46E-04
HIST1H4F	0.045524	3.02E-01	3.66E-01

HIST1H4G	0.031207	4.80E-01	5.45E-01
HIST1H4H	-0.00929	8.33E-01	8.63E-01
HIST1H4I	0.172706	8.17E-05	1.93E-04
HIST1H4J	0.127971	3.63E-03	6.66E-03
HIST1H4K	0.105975	1.61E-02	2.64E-02
HIST1H4L	0.185364	2.30E-05	5.88E-05
HIST2H2AA3	0.143375	1.10E-03	2.20E-03
HIST2H2AB	0.270555	4.33E-10	1.93E-09
HIST2H2AC	0.335916	4.75E-15	3.49E-14
HIST2H2BA	0.072627	9.97E-02	1.37E-01
HIST2H2BE	0.053302	2.27E-01	2.85E-01
HIST2H2BF	0.149604	6.59E-04	1.36E-03
HIST2H3C	0.285143	4.32E-11	2.12E-10
HIST2H3D	0.19942	5.10E-06	1.42E-05
HIST2H4A	0.185525	2.27E-05	5.78E-05
HIST3H2A	0.056618	2.00E-01	2.54E-01
HIST3H2BB	0.037217	3.99E-01	4.66E-01
HIST3H3	-0.08703	4.84E-02	7.15E-02
HIST4H4	-0.08222	6.22E-02	9.01E-02
HIVEP1	-0.13647	1.91E-03	3.67E-03
HIVEP2	-0.01418	7.48E-01	7.90E-01
HIVEP3	-0.01088	8.05E-01	8.39E-01
HJURP	0.882976	1.29E-170	2.34E-167
HK1	-0.07856	7.49E-02	1.07E-01
HK2	0.355197	9.27E-17	8.06E-16
HK3	0.030323	4.92E-01	5.57E-01
HKDC1	-0.17683	5.46E-05	1.32E-04
HKR1	-0.20097	4.30E-06	1.21E-05
HLA-A	0.002755	9.50E-01	9.61E-01
HLA-B	-0.0174	6.94E-01	7.43E-01
HLA-C	-0.00738	8.67E-01	8.92E-01
HLA-DMA	-0.39535	1.03E-20	1.28E-19
HLA-DMB	-0.18885	1.60E-05	4.18E-05
HLA-DOA	-0.27159	3.69E-10	1.66E-09
HLA-DOB	-0.22607	2.16E-07	7.11E-07
HLA-DPA1	-0.27272	3.10E-10	1.41E-09
HLA-DPB1	-0.3313	1.17E-14	8.27E-14
HLA-DPB2	-0.12618	4.13E-03	7.52E-03
HLA-DQA1	-0.16755	1.33E-04	3.05E-04
HLA-DQA2	-0.14838	7.31E-04	1.50E-03
HLA-DQB1	-0.27017	4.59E-10	2.04E-09
HLA-DQB2	-0.39408	1.40E-20	1.72E-19
HLA-DRA	-0.25092	7.78E-09	3.00E-08
HLA-DRB1	-0.30677	1.10E-12	6.43E-12

HLA-DRB5	-0.30607	1.25E-12	7.24E-12
HLA-DRB6	-0.11561	8.64E-03	1.48E-02
HLA-E	-0.17004	1.05E-04	2.45E-04
HLA-F	-0.07365	9.50E-02	1.32E-01
HLA-G	0.030523	4.89E-01	5.54E-01
HLA-H	0.031583	4.75E-01	5.40E-01
HLA-J	-0.19908	5.30E-06	1.47E-05
HLA-L	-0.09029	4.05E-02	6.10E-02
HLCS	-0.08499	5.39E-02	7.89E-02
HLF	-0.53437	2.29E-39	1.03E-37
HLTF	0.455325	1.01E-27	2.21E-26
HLX	-0.20293	3.44E-06	9.80E-06
HM13	0.099644	2.37E-02	3.74E-02
HMBOX1	0.01496	7.35E-01	7.79E-01
HMBS	0.307633	9.46E-13	5.57E-12
HMCN1	-0.3792	4.67E-19	4.99E-18
HMG20A	-0.15943	2.81E-04	6.13E-04
HMG20B	0.106361	1.57E-02	2.58E-02
HMGA1	0.57544	9.66E-47	6.03E-45
HMGA2	0.311309	4.90E-13	2.98E-12
HMGB1	0.186345	2.08E-05	5.34E-05
HMGB2	0.643375	1.64E-61	1.48E-59
HMGB3L1	0.093308	3.43E-02	5.23E-02
HMGB3	0.184436	2.54E-05	6.42E-05
HMGB4	-0.00301	9.46E-01	9.57E-01
HMGCLL1	-0.33027	1.43E-14	1.00E-13
HMGCL	-0.27099	4.05E-10	1.81E-09
HMGCR	0.179725	4.09E-05	1.01E-04
HMGCS1	0.24608	1.53E-08	5.71E-08
HMGCS2	-0.34055	1.89E-15	1.43E-14
HMGN1	0.18115	3.55E-05	8.79E-05
HMGN2	0.297347	5.66E-12	3.07E-11
HMGN3	-0.35928	3.89E-17	3.53E-16
HMGN4	0.286587	3.42E-11	1.70E-10
HMGN5	-0.09911	2.45E-02	3.85E-02
HMGXB3	0.037373	3.97E-01	4.64E-01
HMGXB4	0.450116	4.67E-27	9.64E-26
HMHA1	-0.272	3.47E-10	1.56E-09
HMHB1	0.046444	2.93E-01	3.56E-01
HMMR	0.841918	1.50E-139	5.77E-137
HMOX1	0.063482	1.50E-01	1.98E-01
HMOX2	-0.17659	5.59E-05	1.35E-04
HMP19	0.023931	5.88E-01	6.46E-01
HMSD	0.18061	3.74E-05	9.26E-05

HMX1	0.088527	4.46E-02	6.65E-02
HMX2	0.31969	1.06E-13	6.84E-13
HMX3	0.052596	2.33E-01	2.92E-01
HN1L	0.109661	1.28E-02	2.13E-02
HN1	0.400352	3.02E-21	3.93E-20
HNF1A	0.066016	1.35E-01	1.80E-01
HNF1B	-0.43274	6.45E-25	1.12E-23
HNF4A	0.081169	6.57E-02	9.47E-02
HNF4G	0.137453	1.77E-03	3.42E-03
HNMT	-0.41355	1.08E-22	1.57E-21
HNRNPA0	0.018296	6.79E-01	7.29E-01
HNRNPA1L2	0.165699	1.59E-04	3.59E-04
HNRNPA1	0.102435	2.01E-02	3.21E-02
HNRNPA2B1	0.293378	1.11E-11	5.81E-11
HNRNPA3P1	0.296989	6.02E-12	3.26E-11
HNRNPA3	0.156465	3.65E-04	7.84E-04
HNRNPAB	0.322178	6.66E-14	4.39E-13
HNRNPCL1	-0.01652	7.08E-01	7.56E-01
HNRNPC	0.411796	1.69E-22	2.43E-21
HNRNPD	0.307217	1.02E-12	5.97E-12
HNRNPF	0.317188	1.68E-13	1.07E-12
HNRNPH1	-0.02205	6.18E-01	6.74E-01
HNRNPH2	-0.11672	8.02E-03	1.38E-02
HNRNPH3	0.153795	4.61E-04	9.72E-04
HNRNPK	0.282399	6.74E-11	3.26E-10
HNRNPL	0.458622	3.77E-28	8.49E-27
HNRNPM	0.226524	2.04E-07	6.73E-07
HNRNPR	0.430685	1.13E-24	1.94E-23
HNRNPUL1	0.128512	3.48E-03	6.42E-03
HNRNPUL2	0.03766	3.94E-01	4.61E-01
HNRNPU	0.120444	6.21E-03	1.10E-02
HNRPDL	-0.13877	1.59E-03	3.10E-03
HNRPLL	0.383223	1.84E-19	2.06E-18
HOMER1	0.410897	2.13E-22	3.02E-21
HOMER2	-0.20512	2.68E-06	7.72E-06
HOMER3	0.205803	2.48E-06	7.16E-06
HOMEZ	-0.12083	6.04E-03	1.07E-02
HOOK1	0.073995	9.35E-02	1.30E-01
HOOK2	-0.23917	3.92E-08	1.40E-07
HOOK3	-0.16635	1.49E-04	3.39E-04
HOPX	-0.49844	1.07E-33	3.42E-32
HORMAD1	0.038827	3.79E-01	4.46E-01
HORMAD2	0.035547	4.21E-01	4.87E-01
HOTAIR	0.021309	6.29E-01	6.85E-01

HOXA10	0.265334	9.55E-10	4.09E-09
HOXA11AS	0.32234	6.46E-14	4.27E-13
HOXA11	0.369635	4.06E-18	3.99E-17
HOXA13	0.283338	5.79E-11	2.81E-10
HOXA1	0.343439	1.06E-15	8.23E-15
HOXA2	-0.02138	6.28E-01	6.84E-01
HOXA3	0.123838	4.89E-03	8.80E-03
HOXA4	-0.06284	1.54E-01	2.03E-01
HOXA5	-0.1489	7.00E-04	1.44E-03
HOXA6	0.072982	9.80E-02	1.35E-01
HOXA7	-0.07087	1.08E-01	1.48E-01
HOXA9	0.236406	5.67E-08	1.98E-07
HOXB13	0.176329	5.74E-05	1.39E-04
HOXB1	0.057359	1.94E-01	2.48E-01
HOXB2	0.070597	1.10E-01	1.49E-01
HOXB3	0.020703	6.39E-01	6.94E-01
HOXB4	0.046625	2.91E-01	3.54E-01
HOXB5	0.0734	9.61E-02	1.33E-01
HOXB6	0.137269	1.79E-03	3.46E-03
HOXB7	0.226814	1.96E-07	6.50E-07
HOXB8	0.196179	7.30E-06	1.99E-05
HOXB9	0.308042	8.80E-13	5.20E-12
HOXC10	0.080293	6.87E-02	9.85E-02
HOXC11	0.058472	1.85E-01	2.38E-01
HOXC12	0.148839	7.03E-04	1.45E-03
HOXC13	0.025073	5.70E-01	6.30E-01
HOXC4	0.129188	3.31E-03	6.13E-03
HOXC5	0.070589	1.10E-01	1.49E-01
HOXC6	0.216253	7.26E-07	2.24E-06
HOXC8	0.211608	1.26E-06	3.78E-06
HOXC9	0.188745	1.62E-05	4.22E-05
HOXD10	0.204678	2.82E-06	8.10E-06
HOXD11	0.351407	2.05E-16	1.72E-15
HOXD12	0.236084	5.91E-08	2.07E-07
HOXD13	0.350051	2.72E-16	2.26E-15
HOXD1	-0.32514	3.81E-14	2.57E-13
HOXD3	-0.00963	8.27E-01	8.58E-01
HOXD4	-0.03126	4.79E-01	5.44E-01
HOXD8	0.102206	2.03E-02	3.25E-02
HOXD9	0.097999	2.62E-02	4.09E-02
HP1BP3	0.009904	8.23E-01	8.54E-01
HPCAL1	-0.12193	5.60E-03	9.96E-03
HPCAL4	-0.16389	1.87E-04	4.20E-04
HPCA	0.012854	7.71E-01	8.10E-01

HPDL	0.436804	2.09E-25	3.81E-24
HPD	0.029021	5.11E-01	5.75E-01
HPGDS	-0.39803	5.34E-21	6.82E-20
HPGD	-0.32262	6.13E-14	4.06E-13
HPN	-0.2666	7.89E-10	3.42E-09
HPRT1	0.555362	5.23E-43	2.80E-41
HPR	-0.18031	3.86E-05	9.53E-05
HPS1	-0.31061	5.56E-13	3.35E-12
HPS3	0.365473	1.02E-17	9.66E-17
HPS4	-0.1078	1.44E-02	2.37E-02
HPS5	0.119242	6.75E-03	1.18E-02
HPS6	-0.16427	1.81E-04	4.06E-04
HPSE2	-0.46033	2.26E-28	5.17E-27
HPSE	0.1242	4.76E-03	8.59E-03
HPVC1	0.035507	4.21E-01	4.87E-01
HPX	-0.07963	7.10E-02	1.02E-01
HPYR1	-0.10726	1.49E-02	2.45E-02
HP	-0.16929	1.13E-04	2.62E-04
HRASLS2	-0.14321	1.12E-03	2.23E-03
HRASLS5	-0.18327	2.86E-05	7.18E-05
HRASLS	0.15818	3.14E-04	6.80E-04
HRAS	0.06523	1.39E-01	1.85E-01
HRCT1	-0.15482	4.21E-04	8.95E-04
HRC	-0.26326	1.30E-09	5.50E-09
HRG	0.159459	2.80E-04	6.12E-04
HRH1	0.040225	3.62E-01	4.29E-01
HRH2	0.080079	6.94E-02	9.95E-02
HRH3	0.145251	9.47E-04	1.91E-03
HRH4	-0.22636	2.08E-07	6.87E-07
HRK	0.091276	3.84E-02	5.80E-02
HRNBP3	-0.0452	3.06E-01	3.70E-01
HRNR	-0.00587	8.94E-01	9.14E-01
HRSP12	0.044624	3.12E-01	3.76E-01
HR	-0.28645	3.50E-11	1.73E-10
HS1BP3	-0.03449	4.35E-01	5.01E-01
HS2ST1	0.315429	2.32E-13	1.45E-12
HS3ST1	0.025607	5.62E-01	6.23E-01
HS3ST2	-0.24804	1.17E-08	4.40E-08
HS3ST3A1	0.171844	8.87E-05	2.08E-04
HS3ST3B1	0.022598	6.09E-01	6.66E-01
HS3ST4	-0.17633	5.74E-05	1.39E-04
HS3ST5	-0.15014	6.30E-04	1.31E-03
HS3ST6	-0.0585	1.85E-01	2.38E-01
HS6ST1	-0.13744	1.77E-03	3.42E-03

HS6ST2	-0.15318	4.86E-04	1.02E-03
HS6ST3	-0.011	8.03E-01	8.38E-01
HSBP1L1	-0.06318	1.52E-01	2.00E-01
HSBP1	0.013715	7.56E-01	7.97E-01
HSCB	0.056958	1.97E-01	2.51E-01
HSD11B1L	-0.22242	3.41E-07	1.10E-06
HSD11B1	0.088025	4.59E-02	6.82E-02
HSD11B2	-0.22785	1.72E-07	5.72E-07
HSD17B10	0.302426	2.36E-12	1.34E-11
HSD17B11	-0.35466	1.04E-16	8.97E-16
HSD17B12	-0.06441	1.44E-01	1.91E-01
HSD17B13	-0.4152	7.02E-23	1.04E-21
HSD17B14	0.00868	8.44E-01	8.73E-01
HSD17B1	0.059945	1.74E-01	2.26E-01
HSD17B2	-0.19334	9.93E-06	2.66E-05
HSD17B3	-0.09089	3.92E-02	5.91E-02
HSD17B4	-0.42675	3.30E-24	5.50E-23
HSD17B6	-0.48554	8.10E-32	2.34E-30
HSD17B7P2	-0.2245	2.63E-07	8.58E-07
HSD17B7	-0.1055	1.66E-02	2.71E-02
HSD17B8	-0.28606	3.72E-11	1.84E-10
HSD3B1	0.12104	5.95E-03	1.05E-02
HSD3B2	0.045749	3.00E-01	3.64E-01
HSD3B7	0.116802	7.97E-03	1.38E-02
HSDL1	-0.07569	8.62E-02	1.21E-01
HSDL2	-0.33861	2.79E-15	2.08E-14
HSF1	0.185383	2.30E-05	5.87E-05
HSF2BP	0.354862	9.95E-17	8.62E-16
HSF2	0.149809	6.48E-04	1.34E-03
HSF4	-0.26555	9.25E-10	3.97E-09
HSF5	-0.14913	6.86E-04	1.41E-03
HSFX2	-0.34581	6.52E-16	5.21E-15
HSFY2	-0.12729	3.81E-03	6.97E-03
HSFYL1	0.007005	8.74E-01	8.98E-01
HSH2D	-0.07326	9.68E-02	1.34E-01
HSN2	0.19803	5.95E-06	1.64E-05
HSP90AA1	0.372448	2.16E-18	2.19E-17
HSP90AB1	0.216118	7.38E-07	2.27E-06
HSP90AB2P	0.133478	2.40E-03	4.54E-03
HSP90AB4P	0.037729	3.93E-01	4.60E-01
HSP90B1	0.228714	1.54E-07	5.16E-07
HSP90B3P	0.114178	9.51E-03	1.62E-02
HSPA12A	-0.20817	1.89E-06	5.54E-06
HSPA12B	-0.3012	2.92E-12	1.63E-11

HSPA13	0.391457	2.63E-20	3.17E-19
HSPA14	0.441819	5.09E-26	9.80E-25
HSPA1A	0.165517	1.61E-04	3.65E-04
HSPA1B	0.210298	1.47E-06	4.37E-06
HSPA1L	-0.13748	1.76E-03	3.41E-03
HSPA2	0.124024	4.82E-03	8.69E-03
HSPA4L	0.337781	3.28E-15	2.44E-14
HSPA4	0.317982	1.45E-13	9.28E-13
HSPA5	0.035331	4.24E-01	4.90E-01
HSPA6	0.185685	2.23E-05	5.70E-05
HSPA7	-0.04502	3.08E-01	3.72E-01
HSPA8	0.238413	4.34E-08	1.54E-07
HSPA9	0.239733	3.64E-08	1.30E-07
HSPB11	0.204006	3.05E-06	8.72E-06
HSPB1	0.007899	8.58E-01	8.84E-01
HSPB2	-0.29684	6.18E-12	3.33E-11
HSPB3	-0.26148	1.69E-09	7.06E-09
HSPB6	-0.37754	6.84E-19	7.18E-18
HSPB7	-0.4917	1.05E-32	3.18E-31
HSPB8	-0.28777	2.82E-11	1.42E-10
HSPB9	-0.12749	3.76E-03	6.88E-03
HSPBAP1	0.093917	3.31E-02	5.07E-02
HSPBP1	0.169359	1.12E-04	2.60E-04
HSPC072	0.227271	1.85E-07	6.14E-07
HSPC157	-0.15505	4.13E-04	8.78E-04
HSPC159	-0.12565	4.29E-03	7.79E-03
HSPD1	0.55673	2.97E-43	1.60E-41
HSPE1	0.452788	2.13E-27	4.56E-26
HSPG2	-0.28735	3.01E-11	1.51E-10
HSPH1	0.164011	1.85E-04	4.16E-04
HTATIP2	0.180496	3.79E-05	9.36E-05
HTATSF1	0.037192	4.00E-01	4.66E-01
HTA	0.0688	1.19E-01	1.61E-01
HTN1	0.012469	7.78E-01	8.15E-01
HTR1A	0.042851	3.32E-01	3.97E-01
HTR1B	0.03185	4.71E-01	5.36E-01
HTR1D	0.388172	5.76E-20	6.70E-19
HTR1E	0.048282	2.74E-01	3.36E-01
HTR1F	-0.03904	3.77E-01	4.43E-01
HTR2A	-0.14836	7.32E-04	1.50E-03
HTR2B	-0.02861	5.17E-01	5.81E-01
HTR2C	0.220745	4.20E-07	1.33E-06
HTR3A	0.073445	9.59E-02	1.33E-01
HTR3B	-0.07346	9.59E-02	1.33E-01

HTR3C	-0.23939	3.80E-08	1.36E-07
HTR3D	0.019708	6.55E-01	7.08E-01
HTR3E	0.000275	9.95E-01	9.96E-01
HTR4	-0.22107	4.03E-07	1.28E-06
HTR5A	-0.00254	9.54E-01	9.64E-01
HTR6	0.064903	1.41E-01	1.88E-01
HTR7P1	-0.10884	1.35E-02	2.23E-02
HTR7	0.034889	4.29E-01	4.96E-01
HTRA1	-0.05253	2.34E-01	2.93E-01
HTRA2	0.29326	1.13E-11	5.93E-11
HTRA3	0.103491	1.88E-02	3.03E-02
HTRA4	-0.0793	7.22E-02	1.03E-01
HTT	-0.12361	4.97E-03	8.93E-03
HULC	-0.07938	7.19E-02	1.03E-01
HUNK	-0.29031	1.85E-11	9.48E-11
HUS1B	0.187875	1.77E-05	4.60E-05
HUS1	0.219152	5.10E-07	1.61E-06
HUWE1	0.124782	4.57E-03	8.26E-03
HVCN1	-0.11387	9.70E-03	1.65E-02
HYAL1	-0.21494	8.49E-07	2.60E-06
HYAL2	-0.0693	1.16E-01	1.57E-01
HYAL3	0.071369	1.06E-01	1.45E-01
HYAL4	0.138625	1.61E-03	3.13E-03
HYALP1	0.043079	3.29E-01	3.95E-01
HYDIN	-0.1959	7.52E-06	2.05E-05
HYI	-0.3612	2.57E-17	2.37E-16
HYLS1	0.438588	1.27E-25	2.36E-24
HYMAI	-0.03993	3.66E-01	4.32E-01
HYOU1	0.125569	4.32E-03	7.84E-03
IAH1	0.245579	1.64E-08	6.10E-08
IAPP	0.188916	1.59E-05	4.15E-05
IARS2	0.081792	6.36E-02	9.19E-02
IARS	0.389121	4.60E-20	5.41E-19
IBSP	0.182092	3.22E-05	8.04E-05
IBTK	0.105333	1.68E-02	2.73E-02
ICA1L	-0.09528	3.06E-02	4.72E-02
ICA1	-0.07519	8.83E-02	1.23E-01
ICAM1	-0.19979	4.90E-06	1.37E-05
ICAM2	-0.19673	6.87E-06	1.88E-05
ICAM3	-0.14122	1.31E-03	2.59E-03
ICAM4	-0.38856	5.25E-20	6.13E-19
ICAM5	-0.43624	2.44E-25	4.44E-24
ICK	-0.12537	4.38E-03	7.94E-03
ICMT	0.201182	4.19E-06	1.18E-05

ICOSLG	-0.12749	3.76E-03	6.88E-03
ICOS	0.053954	2.22E-01	2.79E-01
ICT1	0.296731	6.29E-12	3.39E-11
ID1	-0.11426	9.45E-03	1.61E-02
ID2B	0.009463	8.30E-01	8.60E-01
ID2	-0.01061	8.10E-01	8.43E-01
ID3	-0.03254	4.61E-01	5.27E-01
ID4	-0.38195	2.47E-19	2.73E-18
IDE	0.023319	5.98E-01	6.55E-01
IDH1	0.078548	7.49E-02	1.07E-01
IDH2	0.199722	4.94E-06	1.38E-05
IDH3A	0.094915	3.13E-02	4.81E-02
IDH3B	0.056729	1.99E-01	2.53E-01
IDH3G	0.05454	2.17E-01	2.73E-01
IDI1	0.034558	4.34E-01	5.00E-01
IDI2	-0.12329	5.08E-03	9.11E-03
IDO1	0.176783	5.49E-05	1.33E-04
IDO2	-0.17253	8.31E-05	1.96E-04
IDS	-0.03242	4.63E-01	5.29E-01
IDUA	-0.42293	9.20E-24	1.48E-22
IER2	-0.15572	3.90E-04	8.33E-04
IER3IP1	-0.04047	3.59E-01	4.25E-01
IER3	0.148985	6.95E-04	1.43E-03
IER5L	0.23124	1.11E-07	3.79E-07
IER5	0.267853	6.53E-10	2.86E-09
IFFO1	-0.15653	3.63E-04	7.80E-04
IFFO2	-0.25961	2.23E-09	9.15E-09
IFI16	0.138415	1.64E-03	3.18E-03
IFI27L1	0.044775	3.11E-01	3.75E-01
IFI27L2	0.008094	8.55E-01	8.81E-01
IFI27	0.095074	3.10E-02	4.77E-02
IFI30	0.086218	5.05E-02	7.44E-02
IFI35	0.238294	4.41E-08	1.56E-07
IFI44L	0.105657	1.65E-02	2.68E-02
IFI44	0.120109	6.35E-03	1.12E-02
IFI6	0.097038	2.77E-02	4.30E-02
IFIH1	0.191456	1.22E-05	3.22E-05
IFIT1B	0.015526	7.25E-01	7.71E-01
IFIT1	0.039485	3.71E-01	4.38E-01
IFIT2	0.044983	3.08E-01	3.72E-01
IFIT3	0.136764	1.87E-03	3.59E-03
IFIT5	-0.06496	1.41E-01	1.87E-01
IFITM1	-0.03207	4.68E-01	5.34E-01
IFITM2	-0.24724	1.30E-08	4.90E-08

IFITM3	-0.04899	2.67E-01	3.29E-01
IFITM4P	0.046407	2.93E-01	3.56E-01
IFITM5	-0.07135	1.06E-01	1.45E-01
IFLTD1	-0.24291	2.36E-08	8.63E-08
IFNA10	0.019799	6.54E-01	7.07E-01
IFNA13	0.057024	1.96E-01	2.51E-01
IFNA14	-0.02958	5.03E-01	5.67E-01
IFNA16	0.047115	2.86E-01	3.48E-01
IFNA1	0.06294	1.54E-01	2.02E-01
IFNA21	-0.11349	9.95E-03	1.69E-02
IFNA2	0.098791	2.50E-02	3.92E-02
IFNA4	-0.02307	6.01E-01	6.59E-01
IFNA5	-0.06846	1.21E-01	1.63E-01
IFNA7	-0.11663	8.06E-03	1.39E-02
IFNA8	0.026387	5.50E-01	6.12E-01
IFNAR1	-0.16043	2.57E-04	5.64E-04
IFNAR2	-0.03758	3.95E-01	4.62E-01
IFNB1	0.18715	1.91E-05	4.94E-05
IFNE	0.188453	1.67E-05	4.35E-05
IFNGR1	-0.08967	4.20E-02	6.29E-02
IFNGR2	0.00084	9.85E-01	9.88E-01
IFNG	0.288385	2.54E-11	1.28E-10
IFNK	0.03693	4.03E-01	4.69E-01
IFNW1	-0.05093	2.49E-01	3.09E-01
IFRD1	0.4393	1.04E-25	1.94E-24
IFRD2	0.03549	4.22E-01	4.88E-01
IFT122	-0.02673	5.45E-01	6.07E-01
IFT140	-0.54751	1.29E-41	6.39E-40
IFT172	-0.42683	3.23E-24	5.39E-23
IFT20	0.035731	4.18E-01	4.85E-01
IFT27	-0.19585	7.56E-06	2.06E-05
IFT46	-0.12494	4.52E-03	8.17E-03
IFT52	0.101442	2.13E-02	3.39E-02
IFT57	-0.2849	4.50E-11	2.21E-10
IFT74	-0.1529	4.98E-04	1.04E-03
IFT80	-0.19253	1.08E-05	2.89E-05
IFT81	0.064876	1.41E-01	1.88E-01
IFT88	-0.29192	1.41E-11	7.33E-11
IGBP1	-0.13621	1.95E-03	3.74E-03
IGDCC3	0.12107	5.94E-03	1.05E-02
IGDCC4	0.118189	7.25E-03	1.26E-02
IGF1R	-0.12382	4.90E-03	8.81E-03
IGF1	-0.11828	7.21E-03	1.26E-02
IGF2AS	0.092632	3.56E-02	5.41E-02

IGF2BP1	0.373086	1.88E-18	1.90E-17
IGF2BP2	0.371516	2.67E-18	2.67E-17
IGF2BP3	0.545356	3.07E-41	1.49E-39
IGF2R	0.019033	6.67E-01	7.18E-01
IGF2	-0.05042	2.53E-01	3.14E-01
IGFALS	-0.40648	6.55E-22	8.94E-21
IGFBP1	0.221529	3.81E-07	1.22E-06
IGFBP2	-0.15179	5.47E-04	1.14E-03
IGFBP3	0.244398	1.93E-08	7.12E-08
IGFBP4	-0.25122	7.46E-09	2.89E-08
IGFBP5	0.047178	2.85E-01	3.48E-01
IGFBP6	-0.13993	1.46E-03	2.85E-03
IGFBP7	-0.24251	2.50E-08	9.09E-08
IGFBPL1	0.092425	3.60E-02	5.47E-02
IGFL1	0.04487	3.09E-01	3.74E-01
IGFL2	0.100453	2.26E-02	3.58E-02
IGFL3	0.010236	8.17E-01	8.49E-01
IGFL4	0.087936	4.61E-02	6.85E-02
IGFN1	-0.29038	1.83E-11	9.38E-11
IGHMBP2	0.047609	2.81E-01	3.43E-01
IGJ	-0.25508	4.30E-09	1.71E-08
IGLL1	-0.06549	1.38E-01	1.83E-01
IGLL3	-0.16669	1.45E-04	3.29E-04
IGLON5	0.239587	3.71E-08	1.32E-07
IGSF10	-0.27011	4.63E-10	2.06E-09
IGSF11	0.083223	5.91E-02	8.59E-02
IGSF1	0.056051	2.04E-01	2.60E-01
IGSF21	-0.07939	7.18E-02	1.03E-01
IGSF22	-0.10257	1.99E-02	3.19E-02
IGSF3	-0.17783	4.94E-05	1.20E-04
IGSF5	0.029215	5.08E-01	5.72E-01
IGSF6	-0.08245	6.15E-02	8.91E-02
IGSF8	-0.14849	7.24E-04	1.49E-03
IGSF9B	-0.43108	1.02E-24	1.76E-23
IGSF9	0.13619	1.95E-03	3.75E-03
IHH	-0.27646	1.73E-10	8.05E-10
IKBIP	0.345021	7.66E-16	6.08E-15
IKBKAP	-0.01705	7.00E-01	7.48E-01
IKBKB	-0.31147	4.77E-13	2.90E-12
IKBKE	0.015057	7.33E-01	7.77E-01
IKBKG	0.039224	3.74E-01	4.41E-01
IKZF1	-0.17044	1.02E-04	2.37E-04
IKZF2	-0.14317	1.12E-03	2.24E-03
IKZF3	-0.09755	2.68E-02	4.18E-02

IKZF4	-0.24464	1.87E-08	6.90E-08
IKZF5	-0.32676	2.80E-14	1.91E-13
IK	-0.23712	5.16E-08	1.81E-07
IL10RA	-0.09486	3.14E-02	4.82E-02
IL10RB	0.113035	1.03E-02	1.74E-02
IL10	0.075648	8.63E-02	1.21E-01
IL11RA	-0.46948	1.37E-29	3.43E-28
IL11	0.309123	7.26E-13	4.32E-12
IL12A	0.088479	4.48E-02	6.67E-02
IL12B	-0.24515	1.74E-08	6.45E-08
IL12RB1	0.016099	7.15E-01	7.62E-01
IL12RB2	0.443084	3.55E-26	6.93E-25
IL13RA1	-0.00102	9.82E-01	9.86E-01
IL13RA2	-0.23452	7.26E-08	2.51E-07
IL13	-0.03248	4.62E-01	5.28E-01
IL15RA	0.222736	3.28E-07	1.06E-06
IL15	0.168716	1.20E-04	2.76E-04
IL16	-0.27976	1.03E-10	4.88E-10
IL17A	0.106447	1.57E-02	2.57E-02
IL17B	0.048341	2.74E-01	3.35E-01
IL17C	0.161047	2.43E-04	5.36E-04
IL17D	-0.23517	6.67E-08	2.32E-07
IL17F	-0.08996	4.13E-02	6.19E-02
IL17RA	-0.09586	2.96E-02	4.58E-02
IL17RB	0.055524	2.08E-01	2.64E-01
IL17RC	-0.28165	7.60E-11	3.65E-10
IL17RD	0.222547	3.36E-07	1.08E-06
IL17REL	-0.12813	3.58E-03	6.58E-03
IL17RE	-0.47751	1.09E-30	2.93E-29
IL18BP	0.098929	2.48E-02	3.89E-02
IL18R1	0.083838	5.73E-02	8.34E-02
IL18RAP	0.069249	1.17E-01	1.58E-01
IL18	-0.0253	5.67E-01	6.27E-01
IL19	-0.02198	6.19E-01	6.75E-01
IL1A	0.239785	3.61E-08	1.29E-07
IL1B	0.157992	3.19E-04	6.91E-04
IL1F10	0.111467	1.14E-02	1.91E-02
IL1F5	0.289188	2.23E-11	1.13E-10
IL1F6	-0.07649	8.29E-02	1.17E-01
IL1F7	-0.20211	3.78E-06	1.07E-05
IL1F8	0.106773	1.53E-02	2.52E-02
IL1F9	0.036469	4.09E-01	4.75E-01
IL1R1	-0.26125	1.75E-09	7.29E-09
IL1R2	0.380157	3.75E-19	4.04E-18

IL1RAPL1	0.036035	4.14E-01	4.81E-01
IL1RAPL2	0.137614	1.75E-03	3.38E-03
IL1RAP	0.132446	2.60E-03	4.88E-03
IL1RL1	-0.0979	2.63E-02	4.11E-02
IL1RL2	-0.00356	9.36E-01	9.49E-01
IL1RN	0.155338	4.03E-04	8.58E-04
IL20RA	-0.02422	5.83E-01	6.42E-01
IL20RB	0.239465	3.77E-08	1.35E-07
IL20	-0.06542	1.38E-01	1.84E-01
IL21R	0.037082	4.01E-01	4.68E-01
IL21	0.160881	2.46E-04	5.43E-04
IL22RA1	0.271864	3.54E-10	1.60E-09
IL22RA2	-0.0522	2.37E-01	2.96E-01
IL22	0.032354	4.64E-01	5.29E-01
IL23A	0.264832	1.03E-09	4.40E-09
IL23R	-0.097	2.77E-02	4.31E-02
IL24	0.010879	8.05E-01	8.39E-01
IL25	-0.11602	8.40E-03	1.44E-02
IL26	0.04791	2.78E-01	3.40E-01
IL27RA	-0.14751	7.86E-04	1.60E-03
IL27	0.306223	1.21E-12	7.06E-12
IL28A	0.18311	2.91E-05	7.29E-05
IL28B	0.167173	1.38E-04	3.15E-04
IL28RA	-0.22506	2.45E-07	8.03E-07
IL29	0.147988	7.55E-04	1.55E-03
IL2RA	0.22516	2.42E-07	7.93E-07
IL2RB	0.150948	5.88E-04	1.22E-03
IL2RG	-0.06118	1.66E-01	2.16E-01
IL2	-0.17764	5.04E-05	1.23E-04
IL31RA	-0.01246	7.78E-01	8.16E-01
IL31	0.130284	3.06E-03	5.68E-03
IL32	0.095786	2.97E-02	4.59E-02
IL33	-0.41734	4.02E-23	6.09E-22
IL34	-0.26957	5.03E-10	2.23E-09
IL3RA	-0.13858	1.62E-03	3.14E-03
IL3	-0.03168	4.73E-01	5.39E-01
IL4I1	0.252491	6.23E-09	2.44E-08
IL4R	-0.10092	2.20E-02	3.49E-02
IL4	-0.09385	3.32E-02	5.09E-02
IL5RA	-0.25777	2.92E-09	1.18E-08
IL5	0.005015	9.10E-01	9.28E-01
IL6R	-0.45487	1.16E-27	2.52E-26
IL6ST	-0.20954	1.61E-06	4.76E-06
IL6	0.254105	4.95E-09	1.96E-08

IL7R	-0.10136	2.14E-02	3.41E-02
IL7	-0.05594	2.05E-01	2.61E-01
IL8	0.368777	4.91E-18	4.79E-17
IL9R	-0.04734	2.84E-01	3.46E-01
IL9	0.054197	2.20E-01	2.77E-01
ILDR1	-0.33219	9.85E-15	7.01E-14
ILDR2	-0.08681	4.90E-02	7.23E-02
ILF2	0.411022	2.06E-22	2.93E-21
ILF3	0.128653	3.45E-03	6.36E-03
ILKAP	0.213454	1.01E-06	3.07E-06
ILK	-0.16804	1.27E-04	2.92E-04
ILVBL	0.056358	2.02E-01	2.57E-01
IMMP1L	0.126537	4.03E-03	7.34E-03
IMMP2L	-0.01566	7.23E-01	7.69E-01
IMMT	0.496729	1.92E-33	6.07E-32
IMP3	-0.16754	1.33E-04	3.05E-04
IMP4	0.224203	2.73E-07	8.89E-07
IMP5	0.095995	2.94E-02	4.55E-02
IMPA1	0.251653	7.02E-09	2.73E-08
IMPA2	-0.12117	5.90E-03	1.05E-02
IMPACT	0.009438	8.31E-01	8.61E-01
IMPAD1	0.188055	1.74E-05	4.52E-05
IMPDH1	0.053076	2.29E-01	2.88E-01
IMPDH2	-0.02518	5.69E-01	6.28E-01
IMPG1	-0.27941	1.09E-10	5.14E-10
IMPG2	0.070652	1.09E-01	1.49E-01
INADL	-0.2351	6.74E-08	2.34E-07
INA	0.328748	1.92E-14	1.32E-13
INCA1	-0.35703	6.29E-17	5.55E-16
INCENP	0.659511	1.37E-65	1.35E-63
INE1	-0.19282	1.05E-05	2.80E-05
INE2	-0.29018	1.89E-11	9.68E-11
INF2	0.013955	7.52E-01	7.94E-01
ING1	0.1384	1.64E-03	3.19E-03
ING2	0.003014	9.46E-01	9.57E-01
ING3	-0.00118	9.79E-01	9.84E-01
ING4	-0.18756	1.83E-05	4.74E-05
ING5	-0.2476	1.24E-08	4.67E-08
INGX	0.061896	1.61E-01	2.10E-01
INHA	0.059145	1.80E-01	2.33E-01
INHBA	0.238771	4.14E-08	1.47E-07
INHBB	-0.10633	1.58E-02	2.58E-02
INHBC	0.107339	1.48E-02	2.44E-02
INHBE	0.379773	4.09E-19	4.39E-18

INMT	-0.60106	6.77E-52	4.81E-50
INO80B	0.034176	4.39E-01	5.05E-01
INO80C	0.01367	7.57E-01	7.98E-01
INO80D	-0.23814	4.50E-08	1.59E-07
INO80E	-0.03583	4.17E-01	4.84E-01
INO80	-0.22217	3.52E-07	1.13E-06
INPP1	0.07231	1.01E-01	1.39E-01
INPP4A	-0.19065	1.32E-05	3.48E-05
INPP4B	0.250287	8.51E-09	3.27E-08
INPP5A	-0.39239	2.11E-20	2.55E-19
INPP5B	-0.34565	6.73E-16	5.38E-15
INPP5D	-0.17306	7.89E-05	1.87E-04
INPP5E	-0.18625	2.10E-05	5.39E-05
INPP5F	0.024781	5.75E-01	6.34E-01
INPP5J	-0.28407	5.14E-11	2.51E-10
INPP5K	-0.49128	1.21E-32	3.64E-31
INPPL1	-0.12837	3.52E-03	6.48E-03
INS-IGF2	0.00546	9.02E-01	9.21E-01
INSC	-0.03556	4.21E-01	4.87E-01
INSIG1	0.124903	4.53E-03	8.19E-03
INSIG2	-0.08232	6.19E-02	8.97E-02
INSL3	0.170017	1.06E-04	2.46E-04
INSL4	0.11889	6.91E-03	1.21E-02
INSL5	-0.1434	1.10E-03	2.20E-03
INSL6	0.05391	2.22E-01	2.80E-01
INSM1	0.07706	8.06E-02	1.14E-01
INSM2	0.043079	3.29E-01	3.95E-01
INSRR	0.08181	6.36E-02	9.19E-02
INSR	-0.15438	4.38E-04	9.28E-04
INS	0.04236	3.37E-01	4.03E-01
INTS10	-0.06805	1.23E-01	1.66E-01
INTS12	-0.00026	9.95E-01	9.96E-01
INTS1	0.136641	1.88E-03	3.62E-03
INTS2	0.294061	9.88E-12	5.21E-11
INTS3	-0.1783	4.72E-05	1.15E-04
INTS4L1	-0.04532	3.05E-01	3.68E-01
INTS4L2	0.0101	8.19E-01	8.51E-01
INTS4	0.188312	1.70E-05	4.40E-05
INTS5	-0.18068	3.72E-05	9.20E-05
INTS6	0.152367	5.21E-04	1.09E-03
INTS7	0.343538	1.04E-15	8.08E-15
INTS8	0.328364	2.06E-14	1.42E-13
INTS9	-0.17447	6.89E-05	1.64E-04
INTU	-0.01298	7.69E-01	8.08E-01

INVS	0.148609	7.17E-04	1.47E-03
IP6K1	-0.26194	1.58E-09	6.61E-09
IP6K2	-0.23639	5.68E-08	1.98E-07
IP6K3	-0.00704	8.73E-01	8.97E-01
IPCEF1	-0.20284	3.48E-06	9.89E-06
IPMK	0.246999	1.35E-08	5.05E-08
IPO11	0.182947	2.95E-05	7.40E-05
IPO13	-0.01324	7.64E-01	8.04E-01
IPO4	0.301211	2.92E-12	1.63E-11
IPO5	0.288781	2.38E-11	1.21E-10
IPO7	0.135173	2.11E-03	4.02E-03
IPO8	0.341519	1.56E-15	1.19E-14
IPO9	0.289963	1.96E-11	1.00E-10
IPPK	0.267558	6.83E-10	2.98E-09
IPP	0.000134	9.98E-01	9.98E-01
IPW	-0.22452	2.62E-07	8.56E-07
IQCA1	-0.22292	3.20E-07	1.03E-06
IQCB1	0.157664	3.28E-04	7.10E-04
IQCC	0.020127	6.49E-01	7.02E-01
IQCD	0.100657	2.23E-02	3.54E-02
IQCE	-0.09931	2.42E-02	3.81E-02
IQCF1	-0.1346	2.20E-03	4.19E-03
IQCF2	0.007401	8.67E-01	8.92E-01
IQCF3	-0.03253	4.61E-01	5.27E-01
IQCF5	0.154433	4.36E-04	9.24E-04
IQCF6	-0.07897	7.33E-02	1.05E-01
IQCG	-0.14766	7.76E-04	1.59E-03
IQCH	-0.02101	6.34E-01	6.89E-01
IQCJ	0.053641	2.24E-01	2.82E-01
IQCK	-0.31696	1.75E-13	1.11E-12
IQGAP1	-0.18107	3.58E-05	8.87E-05
IQGAP2	-0.1674	1.35E-04	3.09E-04
IQGAP3	0.720473	1.38E-83	1.73E-81
IQSEC1	-0.31252	3.94E-13	2.41E-12
IQSEC2	-0.18747	1.85E-05	4.79E-05
IQSEC3	-0.18923	1.54E-05	4.02E-05
IQUB	-0.18561	2.25E-05	5.74E-05
IRAK1BP1	-0.0521	2.38E-01	2.97E-01
IRAK1	0.333862	7.11E-15	5.12E-14
IRAK2	0.062234	1.58E-01	2.08E-01
IRAK3	-0.1199	6.45E-03	1.13E-02
IRAK4	0.098081	2.60E-02	4.07E-02
IREB2	0.127462	3.76E-03	6.89E-03
IRF1	0.18564	2.24E-05	5.72E-05

IRF2BP1	-0.11333	1.01E-02	1.71E-02
IRF2BP2	-0.36134	2.50E-17	2.31E-16
IRF2	-0.25422	4.87E-09	1.93E-08
IRF3	-0.1031	1.93E-02	3.10E-02
IRF4	-0.12108	5.94E-03	1.05E-02
IRF5	0.037516	3.96E-01	4.62E-01
IRF6	-0.14372	1.07E-03	2.14E-03
IRF7	-0.04453	3.13E-01	3.78E-01
IRF8	-0.16258	2.11E-04	4.70E-04
IRF9	0.065063	1.40E-01	1.86E-01
IRGC	0.046018	2.97E-01	3.61E-01
IRGM	-0.05003	2.57E-01	3.18E-01
IRGQ	-0.11471	9.18E-03	1.57E-02
IRS1	0.166549	1.46E-04	3.33E-04
IRS2	-0.14384	1.06E-03	2.13E-03
IRS4	-0.06055	1.70E-01	2.21E-01
IRX1	-0.39098	2.95E-20	3.53E-19
IRX2	-0.41698	4.42E-23	6.67E-22
IRX3	-0.50276	2.42E-34	8.06E-33
IRX4	0.246207	1.50E-08	5.61E-08
IRX5	-0.46744	2.57E-29	6.30E-28
IRX6	-0.25164	7.03E-09	2.73E-08
ISCA1P1	0.19686	6.77E-06	1.85E-05
ISCA1	0.025735	5.60E-01	6.21E-01
ISCA2	0.037546	3.95E-01	4.62E-01
ISCU	-0.37757	6.78E-19	7.13E-18
ISG15	0.30104	3.00E-12	1.68E-11
ISG20L2	0.334525	6.24E-15	4.52E-14
ISG20	0.006102	8.90E-01	9.11E-01
ISL1	0.049947	2.58E-01	3.19E-01
ISL2	0.278228	1.31E-10	6.16E-10
ISLR2	-0.30403	1.78E-12	1.02E-11
ISLR	-0.02604	5.55E-01	6.17E-01
ISM1	-0.26968	4.95E-10	2.19E-09
ISM2	0.349074	3.33E-16	2.74E-15
ISOC1	-0.07797	7.71E-02	1.09E-01
ISOC2	0.050112	2.56E-01	3.17E-01
ISPD	-0.00032	9.94E-01	9.95E-01
ISX	0.091571	3.78E-02	5.71E-02
ISY1	0.441385	5.75E-26	1.10E-24
ISYNA1	-0.10215	2.04E-02	3.26E-02
ITCH	0.294789	8.74E-12	4.64E-11
ITFG1	-0.13835	1.65E-03	3.20E-03
ITFG2	-0.31679	1.81E-13	1.14E-12

ITFG3	-0.18558	2.26E-05	5.76E-05
ITGA10	-0.39777	5.70E-21	7.25E-20
ITGA11	0.135505	2.06E-03	3.93E-03
ITGA1	-0.01467	7.40E-01	7.83E-01
ITGA2B	0.125702	4.28E-03	7.77E-03
ITGA2	0.065231	1.39E-01	1.85E-01
ITGA3	-0.11977	6.50E-03	1.14E-02
ITGA4	-0.10833	1.39E-02	2.30E-02
ITGA5	0.341307	1.62E-15	1.24E-14
ITGA6	0.140863	1.35E-03	2.66E-03
ITGA7	-0.22618	2.13E-07	7.02E-07
ITGA8	-0.41123	1.96E-22	2.78E-21
ITGA9	-0.51716	1.45E-36	5.45E-35
ITGAD	-0.12057	6.15E-03	1.09E-02
ITGAE	0.187871	1.78E-05	4.60E-05
ITGAL	-0.16868	1.20E-04	2.76E-04
ITGAM	-0.1226	5.34E-03	9.53E-03
ITGAV	0.248502	1.09E-08	4.14E-08
ITGAX	-0.05494	2.13E-01	2.70E-01
ITGB1BP1	0.408125	4.32E-22	5.99E-21
ITGB1BP2	-0.0597	1.76E-01	2.28E-01
ITGB1BP3	-0.01647	7.09E-01	7.57E-01
ITGB1	0.330068	1.49E-14	1.04E-13
ITGB2	-0.11002	1.25E-02	2.08E-02
ITGB3BP	0.346302	5.90E-16	4.74E-15
ITGB3	-0.01534	7.28E-01	7.74E-01
ITGB4	-0.0606	1.70E-01	2.21E-01
ITGB5	0.017186	6.97E-01	7.46E-01
ITGB6	-0.28091	8.55E-11	4.09E-10
ITGB7	0.010674	8.09E-01	8.42E-01
ITGB8	0.121715	5.68E-03	1.01E-02
ITGBL1	-0.31317	3.50E-13	2.16E-12
ITIH1	-0.07017	1.12E-01	1.52E-01
ITIH2	-0.2151	8.34E-07	2.55E-06
ITIH3	-0.41192	1.64E-22	2.35E-21
ITIH4	-0.22172	3.72E-07	1.19E-06
ITIH5L	-0.06154	1.63E-01	2.13E-01
ITIH5	-0.53345	3.26E-39	1.44E-37
ITK	-0.09014	4.09E-02	6.14E-02
ITLN1	-0.17799	4.87E-05	1.19E-04
ITLN2	-0.1643	1.80E-04	4.05E-04
ITM2A	-0.2876	2.89E-11	1.45E-10
ITM2B	-0.33379	7.21E-15	5.19E-14
ITM2C	-0.00086	9.84E-01	9.88E-01

ITPA	0.02716	5.39E-01	6.01E-01
ITPK1	-0.06312	1.53E-01	2.01E-01
ITPKA	0.198111	5.90E-06	1.63E-05
ITPKB	-0.3949	1.15E-20	1.43E-19
ITPKC	-0.07131	1.06E-01	1.45E-01
ITPR1	-0.32454	4.27E-14	2.86E-13
ITPR2	-0.26395	1.18E-09	4.98E-09
ITPR3	-0.23357	8.23E-08	2.83E-07
ITPRIPL1	0.172665	8.20E-05	1.93E-04
ITPRIPL2	-0.06672	1.30E-01	1.75E-01
ITPRIP	-0.1121	1.09E-02	1.84E-02
ITSN1	0.108277	1.40E-02	2.31E-02
ITSN2	-0.179	4.40E-05	1.08E-04
IVD	-0.48707	4.89E-32	1.43E-30
IVL	-0.08928	4.28E-02	6.41E-02
IVNS1ABP	0.011533	7.94E-01	8.30E-01
IWS1	0.156639	3.60E-04	7.73E-04
IYD	-0.37509	1.19E-18	1.23E-17
IZUMO1	-0.13462	2.20E-03	4.18E-03
JAG1	0.016381	7.11E-01	7.58E-01
JAG2	0.006306	8.86E-01	9.08E-01
JAGN1	0.041114	3.52E-01	4.18E-01
JAK1	-0.14815	7.45E-04	1.53E-03
JAK2	-0.06845	1.21E-01	1.63E-01
JAK3	0.036093	4.14E-01	4.80E-01
JAKMIP1	-0.00884	8.41E-01	8.70E-01
JAKMIP2	0.031691	4.73E-01	5.39E-01
JAKMIP3	-0.08046	6.81E-02	9.78E-02
JAM2	-0.40522	8.98E-22	1.21E-20
JAM3	-0.12581	4.24E-03	7.71E-03
JARID2	0.08553	5.24E-02	7.69E-02
JAZF1	-0.1669	1.42E-04	3.23E-04
JDP2	-0.2981	4.98E-12	2.72E-11
JHDM1D	-0.17943	4.22E-05	1.03E-04
JKAMP	0.016619	7.07E-01	7.55E-01
JMJD1C	-0.18549	2.27E-05	5.80E-05
JMJD4	0.124424	4.69E-03	8.46E-03
JMJD5	-0.2985	4.65E-12	2.55E-11
JMJD6	0.375484	1.09E-18	1.13E-17
JMJD7-PLA2G4B	-0.49756	1.45E-33	4.58E-32
JMJD8	-0.42004	1.98E-23	3.08E-22
JMY	-0.23226	9.77E-08	3.34E-07
JOSD1	0.167744	1.31E-04	3.00E-04
JOSD2	-0.03937	3.73E-01	4.39E-01

JPH1	-0.28714	3.12E-11	1.56E-10
JPH2	-0.1041	1.81E-02	2.93E-02
JPH3	0.076109	8.44E-02	1.18E-01
JPH4	-0.42596	4.08E-24	6.75E-23
JRKL	-0.13173	2.74E-03	5.14E-03
JRK	-0.01998	6.51E-01	7.05E-01
JSRP1	0.041332	3.49E-01	4.15E-01
JTB	-0.02752	5.33E-01	5.96E-01
JUB	-0.0528	2.32E-01	2.90E-01
JUNB	-0.14545	9.32E-04	1.88E-03
JUND	-0.29145	1.53E-11	7.92E-11
JUN	-0.25867	2.56E-09	1.04E-08
JUP	0.05622	2.03E-01	2.58E-01
KAAG1	0.18285	2.98E-05	7.47E-05
KAL1	-0.30812	8.68E-13	5.14E-12
KALRN	-0.10412	1.81E-02	2.93E-02
KANK1	-0.41535	6.76E-23	1.00E-21
KANK2	-0.43502	3.43E-25	6.15E-24
KANK3	-0.3882	5.72E-20	6.66E-19
KANK4	-0.17247	8.35E-05	1.97E-04
KARS	0.247202	1.31E-08	4.92E-08
KAT2A	0.053804	2.23E-01	2.80E-01
KAT2B	-0.35183	1.88E-16	1.58E-15
KAT5	-0.2626	1.44E-09	6.03E-09
KATNA1	0.25281	5.96E-09	2.33E-08
KATNAL1	0.009919	8.22E-01	8.54E-01
KATNAL2	-0.18142	3.45E-05	8.58E-05
KATNB1	-0.15426	4.43E-04	9.37E-04
KAZALD1	-0.0008	9.86E-01	9.89E-01
KAZ	-0.29548	7.78E-12	4.14E-11
KBTBD10	-0.20857	1.80E-06	5.30E-06
KBTBD11	-0.29178	1.45E-11	7.50E-11
KBTBD12	0.049241	2.65E-01	3.26E-01
KBTBD13	-0.22756	1.79E-07	5.93E-07
KBTBD2	0.220095	4.54E-07	1.44E-06
KBTBD3	-0.27267	3.13E-10	1.42E-09
KBTBD4	-0.15636	3.68E-04	7.90E-04
KBTBD5	-0.0527	2.33E-01	2.91E-01
KBTBD6	-0.01114	8.01E-01	8.36E-01
KBTBD7	-0.14414	1.04E-03	2.08E-03
KBTBD8	-0.01546	7.26E-01	7.72E-01
KC6	0.125084	4.47E-03	8.09E-03
KCMF1	0.484604	1.10E-31	3.14E-30
KCNA10	-0.14094	1.34E-03	2.65E-03

KCNA1	-0.08165	6.41E-02	9.26E-02
KCNA2	-0.13299	2.49E-03	4.70E-03
KCNA3	-0.23933	3.84E-08	1.37E-07
KCNA4	-0.33187	1.05E-14	7.42E-14
KCNA5	-0.41896	2.63E-23	4.05E-22
KCNA6	-0.12433	4.72E-03	8.51E-03
KCNA7	0.153259	4.83E-04	1.01E-03
KCNAB1	-0.25302	5.78E-09	2.27E-08
KCNAB2	-0.11819	7.25E-03	1.26E-02
KCNAB3	-0.17321	7.78E-05	1.84E-04
KCNB1	-0.18198	3.26E-05	8.12E-05
KCNB2	0.000822	9.85E-01	9.88E-01
KCNC1	0.034526	4.34E-01	5.01E-01
KCNC2	0.071646	1.04E-01	1.43E-01
KCNC3	-0.36557	9.95E-18	9.47E-17
KCNC4	-0.2034	3.27E-06	9.32E-06
KCND1	0.031915	4.70E-01	5.36E-01
KCND2	0.399894	3.38E-21	4.38E-20
KCND3	-0.39293	1.85E-20	2.24E-19
KCNE1L	-0.17791	4.91E-05	1.20E-04
KCNE1	-0.25001	8.85E-09	3.40E-08
KCNE2	-0.12285	5.24E-03	9.38E-03
KCNE3	-0.00171	9.69E-01	9.76E-01
KCNE4	-0.13965	1.49E-03	2.91E-03
KCNF1	0.251076	7.62E-09	2.94E-08
KCNG1	0.200382	4.59E-06	1.29E-05
KCNG2	-0.13517	2.11E-03	4.02E-03
KCNG3	0.205274	2.64E-06	7.59E-06
KCNG4	0.050055	2.57E-01	3.18E-01
KCNH1	-0.04591	2.98E-01	3.62E-01
KCNH2	-0.09826	2.58E-02	4.03E-02
KCNH3	-0.03541	4.23E-01	4.89E-01
KCNH4	-0.03697	4.02E-01	4.69E-01
KCNH5	0.257483	3.04E-09	1.23E-08
KCNH6	0.079031	7.31E-02	1.04E-01
KCNH7	0.217226	6.45E-07	2.00E-06
KCNH8	-0.06261	1.56E-01	2.05E-01
KCNIP1	-0.16098	2.44E-04	5.38E-04
KCNIP2	-0.21382	9.72E-07	2.94E-06
KCNIP3	-0.12193	5.60E-03	9.96E-03
KCNIP4	-0.00257	9.54E-01	9.63E-01
KCNJ10	0.213943	9.57E-07	2.90E-06
KCNJ11	-0.29732	5.69E-12	3.09E-11
KCNJ12	0.037423	3.97E-01	4.63E-01

KCNJ13	-0.10922	1.31E-02	2.18E-02
KCNJ14	0.212986	1.07E-06	3.24E-06
KCNJ15	-0.30402	1.79E-12	1.03E-11
KCNJ16	-0.17168	9.02E-05	2.11E-04
KCNJ1	-0.13396	2.32E-03	4.38E-03
KCNJ2	-0.00426	9.23E-01	9.39E-01
KCNJ3	0.059878	1.75E-01	2.27E-01
KCNJ4	0.183621	2.76E-05	6.95E-05
KCNJ5	-0.25318	5.65E-09	2.22E-08
KCNJ6	0.04252	3.36E-01	4.01E-01
KCNJ8	-0.19336	9.91E-06	2.65E-05
KCNJ9	0.058262	1.87E-01	2.40E-01
KCNK10	0.029113	5.10E-01	5.74E-01
KCNK12	0.272739	3.09E-10	1.41E-09
KCNK13	0.08544	5.27E-02	7.73E-02
KCNK15	-0.19357	9.69E-06	2.60E-05
KCNK16	-0.19334	9.93E-06	2.66E-05
KCNK17	-0.34393	9.56E-16	7.49E-15
KCNK18	0.093081	3.47E-02	5.29E-02
KCNK1	-0.13376	2.35E-03	4.45E-03
KCNK2	0.085397	5.28E-02	7.74E-02
KCNK3	-0.31299	3.62E-13	2.22E-12
KCNK4	-0.2694	5.17E-10	2.29E-09
KCNK5	-0.36273	1.85E-17	1.73E-16
KCNK6	0.06406	1.47E-01	1.94E-01
KCNK7	0.010311	8.15E-01	8.48E-01
KCNK9	0.219213	5.06E-07	1.59E-06
KCNMA1	-0.082	6.29E-02	9.10E-02
KCNMB1	-0.13369	2.36E-03	4.47E-03
KCNMB2	-0.1163	8.25E-03	1.42E-02
KCNMB3	0.024978	5.72E-01	6.31E-01
KCNMB4	0.3	3.59E-12	2.00E-11
KCNN1	0.110093	1.24E-02	2.07E-02
KCNN2	-0.16817	1.26E-04	2.89E-04
KCNN3	-0.08519	5.33E-02	7.82E-02
KCNN4	0.015665	7.23E-01	7.69E-01
KCNQ1DN	0.076838	8.15E-02	1.15E-01
KCNQ1OT1	-0.16599	1.54E-04	3.50E-04
KCNQ1	-0.49834	1.11E-33	3.53E-32
KCNQ2	-0.03725	3.99E-01	4.65E-01
KCNQ3	-0.21302	1.07E-06	3.23E-06
KCNQ4	-0.15061	6.05E-04	1.26E-03
KCNQ5	0.020341	6.45E-01	6.99E-01
KCNRG	-0.2662	8.39E-10	3.62E-09

KCNS1	-0.1337	2.36E-03	4.47E-03
KCNS2	-0.29504	8.38E-12	4.45E-11
KCNS3	-0.23323	8.61E-08	2.96E-07
KCNT1	-0.07095	1.08E-01	1.47E-01
KCNT2	-0.04254	3.35E-01	4.01E-01
KCNU1	0.031535	4.75E-01	5.41E-01
KCNV1	0.239621	3.69E-08	1.32E-07
KCNV2	-0.008	8.56E-01	8.83E-01
KCP	0.291819	1.44E-11	7.46E-11
KCTD10	-0.1038	1.85E-02	2.98E-02
KCTD11	-0.11789	7.40E-03	1.29E-02
KCTD12	-0.11929	6.72E-03	1.18E-02
KCTD13	-0.03107	4.82E-01	5.47E-01
KCTD14	-0.09865	2.52E-02	3.95E-02
KCTD15	-0.0883	4.52E-02	6.73E-02
KCTD16	-0.11986	6.46E-03	1.14E-02
KCTD17	0.125811	4.24E-03	7.71E-03
KCTD18	-0.31765	1.55E-13	9.84E-13
KCTD19	-0.17477	6.68E-05	1.60E-04
KCTD1	-0.07719	8.01E-02	1.13E-01
KCTD20	0.139202	1.54E-03	3.00E-03
KCTD21	-0.06647	1.32E-01	1.77E-01
KCTD2	-0.18418	2.60E-05	6.58E-05
KCTD3	0.226205	2.12E-07	7.00E-07
KCTD4	0.09945	2.40E-02	3.78E-02
KCTD5	0.28605	3.73E-11	1.84E-10
KCTD6	0.16817	1.26E-04	2.89E-04
KCTD7	-0.32302	5.69E-14	3.78E-13
KCTD8	-0.05275	2.32E-01	2.90E-01
KCTD9	0.126042	4.17E-03	7.59E-03
KDELC1	0.271411	3.79E-10	1.71E-09
KDELC2	0.330433	1.38E-14	9.70E-14
KDELR1	0.095034	3.11E-02	4.78E-02
KDELR2	0.170501	1.01E-04	2.35E-04
KDELR3	0.071087	1.07E-01	1.46E-01
KDM1A	0.370513	3.34E-18	3.31E-17
KDM1B	0.069746	1.14E-01	1.55E-01
KDM2A	-0.00166	9.70E-01	9.77E-01
KDM2B	0.112271	1.08E-02	1.82E-02
KDM3A	0.244286	1.96E-08	7.22E-08
KDM3B	-0.22898	1.49E-07	5.00E-07
KDM4A	-0.121	5.97E-03	1.06E-02
KDM4B	-0.04239	3.37E-01	4.02E-01
KDM4C	-0.21616	7.34E-07	2.26E-06

KDM4DL	-0.03428	4.38E-01	5.04E-01
KDM4D	0.000701	9.87E-01	9.90E-01
KDM5A	0.093363	3.42E-02	5.21E-02
KDM5B	-0.02229	6.14E-01	6.70E-01
KDM5C	-0.06619	1.34E-01	1.79E-01
KDM5D	0.007086	8.73E-01	8.96E-01
KDM6A	-0.14579	9.06E-04	1.83E-03
KDM6B	-0.24375	2.11E-08	7.74E-08
KDR	-0.15497	4.16E-04	8.84E-04
KDSR	-0.03257	4.61E-01	5.27E-01
KEAP1	-0.04708	2.86E-01	3.49E-01
KEL	-0.05126	2.46E-01	3.06E-01
KERA	-0.05688	1.98E-01	2.52E-01
KGFLP1	-0.09136	3.82E-02	5.77E-02
KGFLP2	-0.16215	2.20E-04	4.88E-04
KHDC1L	0.263345	1.29E-09	5.43E-09
KHDC1	0.201124	4.22E-06	1.19E-05
KHDRBS1	0.424507	6.03E-24	9.83E-23
KHDRBS2	-0.22887	1.51E-07	5.07E-07
KHDRBS3	-0.21726	6.43E-07	2.00E-06
KHK	0.187078	1.93E-05	4.97E-05
KHNYN	-0.02311	6.01E-01	6.58E-01
KHSRP	0.359889	3.41E-17	3.12E-16
KIAA0020	0.272888	3.02E-10	1.38E-09
KIAA0040	-0.27263	3.14E-10	1.43E-09
KIAA0087	-0.17042	1.02E-04	2.37E-04
KIAA0090	0.390639	3.20E-20	3.81E-19
KIAA0100	0.044007	3.19E-01	3.84E-01
KIAA0101	0.808311	4.13E-120	9.82E-118
KIAA0114	-0.05273	2.32E-01	2.91E-01
KIAA0125	-0.10524	1.69E-02	2.75E-02
KIAA0141	-0.4676	2.45E-29	6.02E-28
KIAA0146	-0.15072	6.00E-04	1.25E-03
KIAA0174	-0.1825	3.09E-05	7.73E-05
KIAA0182	-0.13345	2.41E-03	4.55E-03
KIAA0195	-0.13342	2.41E-03	4.56E-03
KIAA0196	0.171383	9.28E-05	2.17E-04
KIAA0226	0.100779	2.22E-02	3.52E-02
KIAA0232	-0.268	6.39E-10	2.80E-09
KIAA0240	-0.36651	8.09E-18	7.75E-17
KIAA0247	-0.29293	1.19E-11	6.24E-11
KIAA0284	-0.14682	8.32E-04	1.69E-03
KIAA0317	0.167323	1.36E-04	3.11E-04
KIAA0319L	-0.49235	8.44E-33	2.57E-31

KIAA0319	0.106189	1.59E-02	2.60E-02
KIAA0355	-0.15193	5.41E-04	1.13E-03
KIAA0368	-0.05924	1.79E-01	2.32E-01
KIAA0391	0.207628	2.01E-06	5.88E-06
KIAA0406	0.424919	5.40E-24	8.84E-23
KIAA0408	-0.47372	3.62E-30	9.41E-29
KIAA0415	0.029718	5.01E-01	5.65E-01
KIAA0427	-0.27693	1.61E-10	7.51E-10
KIAA0430	-0.48492	9.90E-32	2.85E-30
KIAA0467	-0.34371	1.00E-15	7.81E-15
KIAA0494	-0.46905	1.56E-29	3.90E-28
KIAA0495	-0.38331	1.80E-19	2.01E-18
KIAA0513	-0.43397	4.59E-25	8.13E-24
KIAA0528	0.108902	1.34E-02	2.23E-02
KIAA0556	-0.24801	1.17E-08	4.42E-08
KIAA0562	-0.22157	3.79E-07	1.21E-06
KIAA0564	-0.20001	4.78E-06	1.34E-05
KIAA0586	0.203566	3.20E-06	9.15E-06
KIAA0649	-0.26088	1.85E-09	7.67E-09
KIAA0652	0.001036	9.81E-01	9.86E-01
KIAA0664P3	-0.34989	2.82E-16	2.33E-15
KIAA0664	-0.04831	2.74E-01	3.36E-01
KIAA0748	-0.24955	9.44E-09	3.61E-08
KIAA0753	-0.24695	1.36E-08	5.08E-08
KIAA0754	0.019185	6.64E-01	7.16E-01
KIAA0776	-0.05513	2.12E-01	2.68E-01
KIAA0802	0.150324	6.20E-04	1.29E-03
KIAA0831	-0.12939	3.27E-03	6.05E-03
KIAA0892	-0.33097	1.25E-14	8.77E-14
KIAA0895L	-0.23775	4.74E-08	1.67E-07
KIAA0895	0.13373	2.36E-03	4.46E-03
KIAA0907	-0.06707	1.28E-01	1.72E-01
KIAA0913	-0.26471	1.05E-09	4.47E-09
KIAA0922	0.026546	5.48E-01	6.10E-01
KIAA0947	0.16056	2.54E-04	5.58E-04
KIAA1009	-0.1605	2.55E-04	5.60E-04
KIAA1012	0.027249	5.37E-01	6.00E-01
KIAA1024	0.149916	6.42E-04	1.33E-03
KIAA1033	0.296049	7.06E-12	3.79E-11
KIAA1045	-0.09122	3.85E-02	5.81E-02
KIAA1107	0.034015	4.41E-01	5.08E-01
KIAA1109	-0.39482	1.17E-20	1.45E-19
KIAA1143	-0.17168	9.01E-05	2.11E-04
KIAA1147	-0.07402	9.34E-02	1.30E-01

KIAA1161	0.078152	7.64E-02	1.08E-01
KIAA1191	-0.19937	5.13E-06	1.43E-05
KIAA1199	0.099509	2.39E-02	3.77E-02
KIAA1210	0.006462	8.84E-01	9.06E-01
KIAA1211	0.208662	1.78E-06	5.25E-06
KIAA1217	-0.1997	4.95E-06	1.38E-05
KIAA1239	0.244951	1.79E-08	6.62E-08
KIAA1244	-0.26482	1.03E-09	4.40E-09
KIAA1257	-0.03803	3.89E-01	4.56E-01
KIAA1267	-0.16341	1.96E-04	4.38E-04
KIAA1274	-0.25963	2.23E-09	9.13E-09
KIAA1279	0.132379	2.61E-03	4.91E-03
KIAA1310	-0.13674	1.87E-03	3.60E-03
KIAA1324L	-0.17804	4.85E-05	1.18E-04
KIAA1324	-0.33864	2.77E-15	2.07E-14
KIAA1328	-0.36877	4.91E-18	4.79E-17
KIAA1370	-0.31555	2.27E-13	1.42E-12
KIAA1377	-0.12054	6.17E-03	1.09E-02
KIAA1383	-0.06944	1.15E-01	1.57E-01
KIAA1407	-0.32969	1.60E-14	1.11E-13
KIAA1409	-0.06484	1.42E-01	1.88E-01
KIAA1429	0.241161	3.00E-08	1.08E-07
KIAA1430	-0.07294	9.82E-02	1.35E-01
KIAA1432	0.088278	4.52E-02	6.73E-02
KIAA1462	-0.07632	8.36E-02	1.17E-01
KIAA1467	0.09232	3.62E-02	5.50E-02
KIAA1468	0.017651	6.89E-01	7.39E-01
KIAA1486	-0.08094	6.64E-02	9.56E-02
KIAA1522	-0.20886	1.74E-06	5.14E-06
KIAA1524	0.83951	5.20E-138	1.86E-135
KIAA1529	-0.35836	4.73E-17	4.26E-16
KIAA1530	-0.11747	7.62E-03	1.32E-02
KIAA1539	-0.00716	8.71E-01	8.95E-01
KIAA1543	-0.24453	1.89E-08	7.00E-08
KIAA1549	0.029446	5.05E-01	5.69E-01
KIAA1586	0.197062	6.62E-06	1.81E-05
KIAA1598	0.016332	7.12E-01	7.59E-01
KIAA1609	0.229429	1.41E-07	4.74E-07
KIAA1614	-0.02336	5.97E-01	6.55E-01
KIAA1632	-0.12472	4.59E-03	8.29E-03
KIAA1644	-0.06981	1.14E-01	1.54E-01
KIAA1671	-0.41549	6.51E-23	9.65E-22
KIAA1683	-0.40796	4.50E-22	6.24E-21
KIAA1704	0.032335	4.64E-01	5.30E-01

KIAA1712	0.011193	8.00E-01	8.35E-01
KIAA1715	0.349815	2.86E-16	2.36E-15
KIAA1731	0.226313	2.09E-07	6.90E-07
KIAA1737	-0.24358	2.16E-08	7.90E-08
KIAA1751	-0.20277	3.51E-06	9.96E-06
KIAA1755	-0.09921	2.43E-02	3.83E-02
KIAA1797	-0.03811	3.88E-01	4.55E-01
KIAA1804	-0.10298	1.94E-02	3.12E-02
KIAA1826	-0.12646	4.05E-03	7.38E-03
KIAA1841	0.23999	3.51E-08	1.26E-07
KIAA1875	-0.14204	1.23E-03	2.44E-03
KIAA1908	-0.34149	1.56E-15	1.20E-14
KIAA1919	0.087631	4.68E-02	6.95E-02
KIAA1949	0.285367	4.17E-11	2.05E-10
KIAA1958	0.008476	8.48E-01	8.75E-01
KIAA1967	-0.02633	5.51E-01	6.13E-01
KIAA1984	-0.2861	3.70E-11	1.83E-10
KIAA2013	0.103551	1.87E-02	3.02E-02
KIAA2018	-0.17893	4.43E-05	1.09E-04
KIAA2022	-0.41036	2.45E-22	3.46E-21
KIAA2026	-0.21129	1.31E-06	3.91E-06
KIDINS220	-0.18437	2.55E-05	6.47E-05
KIF11	0.861157	7.38E-153	4.47E-150
KIF12	-0.39605	8.67E-21	1.09E-19
KIF13A	-0.31172	4.55E-13	2.77E-12
KIF13B	-0.21626	7.25E-07	2.24E-06
KIF14	0.810296	3.77E-121	9.20E-119
KIF15	0.848813	4.18E-144	1.90E-141
KIF16B	-0.36262	1.89E-17	1.76E-16
KIF17	-0.13923	1.54E-03	3.00E-03
KIF18A	0.753761	1.35E-95	2.00E-93
KIF18B	0.827673	8.51E-131	2.50E-128
KIF19	-0.13117	2.86E-03	5.34E-03
KIF1A	0.223579	2.95E-07	9.57E-07
KIF1B	0.018624	6.73E-01	7.24E-01
KIF1C	-0.46184	1.43E-28	3.31E-27
KIF20A	0.840323	1.58E-138	5.75E-136
KIF20B	0.714137	1.70E-81	2.12E-79
KIF21A	0.161947	2.24E-04	4.96E-04
KIF21B	-0.00796	8.57E-01	8.83E-01
KIF22	0.360551	2.96E-17	2.72E-16
KIF23	0.878357	1.42E-166	1.58E-163
KIF24	0.520465	4.32E-37	1.66E-35
KIF25	-0.10366	1.86E-02	3.00E-02

KIF26A	-0.20759	2.02E-06	5.90E-06
KIF26B	0.020921	6.36E-01	6.91E-01
KIF27	-0.19847	5.67E-06	1.57E-05
KIF2A	0.365708	9.65E-18	9.19E-17
KIF2C	0.871724	4.73E-161	4.51E-158
KIF3A	-0.1988	5.47E-06	1.52E-05
KIF3B	0.105179	1.70E-02	2.76E-02
KIF3C	0.447922	8.84E-27	1.79E-25
KIF4A	0.888827	5.49E-176	1.22E-172
KIF4B	0.689083	9.19E-74	1.01E-71
KIF5A	0.142638	1.17E-03	2.33E-03
KIF5B	0.209234	1.67E-06	4.92E-06
KIF5C	0.126565	4.02E-03	7.33E-03
KIF6	-0.43529	3.18E-25	5.72E-24
KIF7	0.048625	2.71E-01	3.33E-01
KIF9	-0.32035	9.37E-14	6.08E-13
KIFAP3	-0.12083	6.04E-03	1.07E-02
KIFC1	0.843966	7.02E-141	2.86E-138
KIFC2	-0.02748	5.34E-01	5.97E-01
KIFC3	-0.20732	2.09E-06	6.07E-06
KILLIN	-0.19997	4.80E-06	1.34E-05
KIN	0.203828	3.11E-06	8.89E-06
KIR2DL1	0.100188	2.30E-02	3.64E-02
KIR2DL3	0.193735	9.52E-06	2.55E-05
KIR2DL4	0.380305	3.62E-19	3.91E-18
KIR2DS4	0.126436	4.06E-03	7.39E-03
KIR3DL1	0.092503	3.58E-02	5.45E-02
KIR3DL2	0.1682	1.25E-04	2.89E-04
KIR3DL3	0.15124	5.74E-04	1.20E-03
KIR3DP1	0.081813	6.36E-02	9.19E-02
KIR3DX1	0.165937	1.55E-04	3.51E-04
KIRREL2	0.148534	7.21E-04	1.48E-03
KIRREL3	-0.07224	1.02E-01	1.39E-01
KIRREL	-0.0799	7.00E-02	1.00E-01
KISS1R	0.241658	2.80E-08	1.02E-07
KISS1	-0.04479	3.10E-01	3.75E-01
KITLG	-0.1587	3.00E-04	6.52E-04
KIT	-0.28249	6.64E-11	3.21E-10
KLB	-0.2068	2.21E-06	6.43E-06
KLC1	0.01165	7.92E-01	8.28E-01
KLC2	0.214574	8.88E-07	2.70E-06
KLC3	0.232165	9.88E-08	3.38E-07
KLC4	-0.43381	4.80E-25	8.47E-24
KLF10	0.032231	4.65E-01	5.31E-01

KLF11	-0.04328	3.27E-01	3.92E-01
KLF12	-0.10053	2.25E-02	3.57E-02
KLF13	-0.2865	3.47E-11	1.72E-10
KLF14	0.149632	6.58E-04	1.36E-03
KLF15	-0.53838	4.83E-40	2.26E-38
KLF16	0.232695	9.23E-08	3.16E-07
KLF17	-0.02739	5.35E-01	5.98E-01
KLF1	0.273633	2.69E-10	1.23E-09
KLF2	-0.41625	5.34E-23	8.00E-22
KLF3	-0.03997	3.65E-01	4.32E-01
KLF4	0.006975	8.75E-01	8.98E-01
KLF5	-0.14626	8.71E-04	1.77E-03
KLF6	-0.00835	8.50E-01	8.77E-01
KLF7	0.11891	6.90E-03	1.21E-02
KLF8	-0.20254	3.60E-06	1.02E-05
KLF9	-0.32165	7.35E-14	4.82E-13
KLHDC10	0.028137	5.24E-01	5.88E-01
KLHDC1	-0.54185	1.23E-40	5.92E-39
KLHDC2	-0.33372	7.32E-15	5.27E-14
KLHDC3	0.073569	9.54E-02	1.32E-01
KLHDC4	-0.1355	2.06E-03	3.93E-03
KLHDC5	0.217836	5.99E-07	1.87E-06
KLHDC7A	-0.38143	2.79E-19	3.05E-18
KLHDC7B	0.204749	2.80E-06	8.04E-06
KLHDC8A	-0.13463	2.20E-03	4.18E-03
KLHDC8B	-0.39723	6.51E-21	8.21E-20
KLHDC9	-0.30725	1.01E-12	5.94E-12
KLHL10	0.04858	2.71E-01	3.33E-01
KLHL11	0.025945	5.57E-01	6.18E-01
KLHL12	-0.00555	9.00E-01	9.19E-01
KLHL13	0.036489	4.09E-01	4.75E-01
KLHL14	-0.07174	1.04E-01	1.42E-01
KLHL15	0.120401	6.23E-03	1.10E-02
KLHL17	-0.03	4.97E-01	5.62E-01
KLHL18	0.078753	7.42E-02	1.06E-01
KLHL1	0.126946	3.91E-03	7.14E-03
KLHL20	-0.29517	8.19E-12	4.36E-11
KLHL21	-0.26351	1.25E-09	5.30E-09
KLHL22	-0.11459	9.25E-03	1.58E-02
KLHL23	0.22395	2.82E-07	9.16E-07
KLHL24	-0.15345	4.75E-04	1.00E-03
KLHL25	0.095025	3.11E-02	4.78E-02
KLHL26	-0.3509	2.28E-16	1.91E-15
KLHL28	-0.03357	4.47E-01	5.13E-01

KLHL29	-0.19142	1.22E-05	3.23E-05
KLHL2	0.045468	3.03E-01	3.67E-01
KLHL30	-0.18649	2.05E-05	5.26E-05
KLHL31	0.267158	7.26E-10	3.16E-09
KLHL32	-0.26403	1.16E-09	4.93E-09
KLHL33	-0.37662	8.42E-19	8.80E-18
KLHL34	0.049015	2.67E-01	3.29E-01
KLHL35	-0.10908	1.33E-02	2.20E-02
KLHL36	0.023116	6.01E-01	6.58E-01
KLHL38	-0.18997	1.42E-05	3.73E-05
KLHL3	-0.21531	8.12E-07	2.49E-06
KLHL4	-0.05866	1.84E-01	2.37E-01
KLHL5	0.405692	7.98E-22	1.08E-20
KLHL6	-0.14695	8.23E-04	1.68E-03
KLHL7	0.242916	2.36E-08	8.63E-08
KLHL8	-0.0856	5.22E-02	7.67E-02
KLHL9	-0.08634	5.02E-02	7.40E-02
KLK10	-0.08141	6.49E-02	9.36E-02
KLK11	-0.23329	8.54E-08	2.94E-07
KLK12	0.061881	1.61E-01	2.10E-01
KLK13	-0.09621	2.90E-02	4.49E-02
KLK14	0.055531	2.08E-01	2.64E-01
KLK15	0.168461	1.22E-04	2.82E-04
KLK1	0.199272	5.19E-06	1.44E-05
KLK2	0.274827	2.24E-10	1.03E-09
KLK3	0.085359	5.29E-02	7.76E-02
KLK4	0.100795	2.22E-02	3.52E-02
KLK5	0.078391	7.55E-02	1.07E-01
KLK6	0.272504	3.21E-10	1.45E-09
KLK7	-0.01455	7.42E-01	7.85E-01
KLK8	0.166249	1.51E-04	3.42E-04
KLK9	0.206452	2.30E-06	6.67E-06
KLKB1	-0.22646	2.06E-07	6.78E-07
KLKP1	0.12546	4.35E-03	7.89E-03
KLRA1	-0.13913	1.55E-03	3.02E-03
KLRAQ1	-0.08545	5.26E-02	7.73E-02
KLRB1	-0.2299	1.32E-07	4.47E-07
KLRC1	0.233165	8.68E-08	2.98E-07
KLRC2	0.3259	3.30E-14	2.24E-13
KLRC3	0.319118	1.18E-13	7.57E-13
KLRC4	0.145897	8.98E-04	1.82E-03
KLRD1	0.207549	2.03E-06	5.93E-06
KLRF1	0.026288	5.52E-01	6.13E-01
KLRG1	-0.15849	3.05E-04	6.63E-04

KLRG2	-0.11249	1.06E-02	1.80E-02
KLRK1	0.018892	6.69E-01	7.20E-01
KL	-0.28923	2.21E-11	1.12E-10
KMO	0.091887	3.71E-02	5.62E-02
KNCN	-0.09231	3.62E-02	5.50E-02
KNDC1	-0.38086	3.19E-19	3.46E-18
KNG1	0.060096	1.73E-01	2.25E-01
KNTC1	0.648501	8.84E-63	8.26E-61
KPNA1	0.416296	5.28E-23	7.92E-22
KPNA2	0.84636	1.85E-142	8.21E-140
KPNA3	0.2487	1.06E-08	4.04E-08
KPNA4	0.560526	6.06E-44	3.38E-42
KPNA5	-0.07402	9.33E-02	1.30E-01
KPNA6	-0.0055	9.01E-01	9.20E-01
KPNA7	0.007786	8.60E-01	8.86E-01
KPNB1	0.559549	9.14E-44	5.02E-42
KPRP	0.102714	1.97E-02	3.16E-02
KPTN	0.173954	7.24E-05	1.72E-04
KRAS	0.255983	3.78E-09	1.51E-08
KRBA1	-0.14985	6.46E-04	1.34E-03
KRBA2	-0.2064	2.32E-06	6.71E-06
KRCC1	-0.12996	3.13E-03	5.81E-03
KREMEN1	-0.17268	8.19E-05	1.93E-04
KREMEN2	0.358811	4.30E-17	3.89E-16
KRI1	0.067909	1.24E-01	1.67E-01
KRIT1	0.085591	5.22E-02	7.67E-02
KRR1	0.470169	1.10E-29	2.78E-28
KRT10	0.138874	1.58E-03	3.08E-03
KRT12	0.099944	2.33E-02	3.68E-02
KRT13	-0.13484	2.17E-03	4.12E-03
KRT14	0.068384	1.21E-01	1.64E-01
KRT15	-0.08807	4.58E-02	6.81E-02
KRT16	0.231363	1.10E-07	3.73E-07
KRT17	0.040948	3.54E-01	4.20E-01
KRT18	0.233936	7.85E-08	2.71E-07
KRT19	0.118756	6.98E-03	1.22E-02
KRT1	-0.24667	1.41E-08	5.28E-08
KRT20	0.046629	2.91E-01	3.54E-01
KRT222	-0.05306	2.29E-01	2.88E-01
KRT23	0.047258	2.84E-01	3.47E-01
KRT24	-0.04573	3.00E-01	3.64E-01
KRT25	0.086156	5.07E-02	7.47E-02
KRT26	0.0326	4.60E-01	5.26E-01
KRT27	-0.29861	4.56E-12	2.50E-11

KRT28	0.045769	3.00E-01	3.63E-01
KRT2	-0.03308	4.54E-01	5.20E-01
KRT31	0.10215	2.04E-02	3.26E-02
KRT32	-0.04239	3.37E-01	4.02E-01
KRT33A	0.077587	7.86E-02	1.11E-01
KRT33B	-0.09106	3.89E-02	5.86E-02
KRT34	0.16813	1.26E-04	2.90E-04
KRT35	0.051006	2.48E-01	3.08E-01
KRT36	0.12388	4.87E-03	8.77E-03
KRT37	0.119407	6.67E-03	1.17E-02
KRT38	0.08317	5.93E-02	8.61E-02
KRT39	0.003065	9.45E-01	9.57E-01
KRT3	0.047809	2.79E-01	3.41E-01
KRT40	0.05244	2.35E-01	2.94E-01
KRT4	-0.14164	1.27E-03	2.51E-03
KRT5	-0.06766	1.25E-01	1.68E-01
KRT6A	0.193134	1.02E-05	2.71E-05
KRT6B	0.210884	1.38E-06	4.10E-06
KRT6C	0.190338	1.37E-05	3.60E-05
KRT71	-0.05965	1.76E-01	2.29E-01
KRT72	-0.1346	2.20E-03	4.19E-03
KRT73	-0.15359	4.69E-04	9.89E-04
KRT74	0.02525	5.68E-01	6.27E-01
KRT75	0.094269	3.24E-02	4.98E-02
KRT76	0.080072	6.94E-02	9.95E-02
KRT77	0.081737	6.38E-02	9.22E-02
KRT78	0.224367	2.67E-07	8.72E-07
KRT79	-0.16532	1.64E-04	3.71E-04
KRT7	0.026877	5.43E-01	6.05E-01
KRT80	0.25676	3.38E-09	1.36E-08
KRT81	0.109469	1.29E-02	2.15E-02
KRT82	0.048217	2.75E-01	3.37E-01
KRT83	0.125117	4.46E-03	8.07E-03
KRT84	-0.02512	5.69E-01	6.29E-01
KRT85	0.101838	2.08E-02	3.32E-02
KRT86	0.088594	4.45E-02	6.63E-02
KRT8	0.22127	3.93E-07	1.26E-06
KRT9	0.107024	1.51E-02	2.48E-02
KRTAP1-1	0.064428	1.44E-01	1.91E-01
KRTAP1-3	0.115177	8.89E-03	1.52E-02
KRTAP1-5	-0.01719	6.97E-01	7.46E-01
KRTAP10-10	0.01096	8.04E-01	8.38E-01
KRTAP10-11	0.051255	2.46E-01	3.06E-01
KRTAP10-12	-0.08141	6.49E-02	9.36E-02

KRTAP10-1	-0.03101	4.83E-01	5.48E-01
KRTAP10-2	0.021937	6.19E-01	6.76E-01
KRTAP10-3	-0.02381	5.90E-01	6.48E-01
KRTAP10-4	0.135319	2.09E-03	3.98E-03
KRTAP10-5	0.030865	4.85E-01	5.50E-01
KRTAP10-6	0.159116	2.89E-04	6.29E-04
KRTAP10-7	0.039576	3.70E-01	4.36E-01
KRTAP10-9	0.073324	9.65E-02	1.33E-01
KRTAP11-1	-0.04378	3.21E-01	3.86E-01
KRTAP12-1	0.069508	1.15E-01	1.56E-01
KRTAP12-2	0.052807	2.32E-01	2.90E-01
KRTAP12-4	-0.03372	4.45E-01	5.12E-01
KRTAP13-1	-0.06203	1.60E-01	2.09E-01
KRTAP13-2	-0.09702	2.77E-02	4.30E-02
KRTAP13-4	-0.10192	2.07E-02	3.30E-02
KRTAP17-1	0.053381	2.27E-01	2.84E-01
KRTAP19-1	0.010011	8.21E-01	8.52E-01
KRTAP19-3	0.025605	5.62E-01	6.23E-01
KRTAP19-5	0.013401	7.62E-01	8.02E-01
KRTAP19-8	0.000442	9.92E-01	9.94E-01
KRTAP2-1	0.102238	2.03E-02	3.25E-02
KRTAP2-2	-0.0262	5.53E-01	6.14E-01
KRTAP20-4	0.039144	3.75E-01	4.41E-01
KRTAP21-2	0.041862	3.43E-01	4.09E-01
KRTAP26-1	-0.07219	1.02E-01	1.40E-01
KRTAP3-1	0.131086	2.88E-03	5.37E-03
KRTAP3-2	0.043183	3.28E-01	3.93E-01
KRTAP3-3	0.063987	1.47E-01	1.94E-01
KRTAP4-11	-0.02776	5.30E-01	5.93E-01
KRTAP4-12	0.049215	2.65E-01	3.26E-01
KRTAP4-1	0.019613	6.57E-01	7.10E-01
KRTAP4-2	-0.04068	3.57E-01	4.23E-01
KRTAP4-3	0.022525	6.10E-01	6.67E-01
KRTAP4-4	0.135359	2.08E-03	3.97E-03
KRTAP4-5	0.090224	4.07E-02	6.11E-02
KRTAP4-7	0.032624	4.60E-01	5.26E-01
KRTAP4-8	0.006165	8.89E-01	9.10E-01
KRTAP4-9	0.051178	2.46E-01	3.06E-01
KRTAP5-10	-0.28859	2.46E-11	1.24E-10
KRTAP5-11	-0.05846	1.85E-01	2.39E-01
KRTAP5-1	-0.16408	1.84E-04	4.13E-04
KRTAP5-2	-0.16212	2.20E-04	4.89E-04
KRTAP5-3	-0.01169	7.91E-01	8.27E-01
KRTAP5-4	-0.01908	6.66E-01	7.17E-01

KRTAP5-5	0.090053	4.11E-02	6.17E-02
KRTAP5-6	0.083785	5.74E-02	8.36E-02
KRTAP5-7	-0.15394	4.55E-04	9.62E-04
KRTAP5-8	-0.31706	1.72E-13	1.09E-12
KRTAP5-9	-0.33005	1.49E-14	1.04E-13
KRTAP6-3	0.030915	4.84E-01	5.49E-01
KRTAP7-1	-0.00272	9.51E-01	9.61E-01
KRTAP8-1	-0.0315	4.76E-01	5.41E-01
KRTAP9-2	0.113115	1.02E-02	1.73E-02
KRTAP9-4	0.117176	7.77E-03	1.34E-02
KRTAP9-9	0.107567	1.46E-02	2.41E-02
KRTCAP2	-0.02225	6.14E-01	6.71E-01
KRTCAP3	-0.0957	2.99E-02	4.61E-02
KRTDAP	0.068995	1.18E-01	1.59E-01
KSR1	-0.14068	1.37E-03	2.70E-03
KSR2	-0.1917	1.18E-05	3.14E-05
KTELC1	0.065371	1.38E-01	1.84E-01
KTI12	0.141174	1.32E-03	2.60E-03
KTN1	0.076101	8.45E-02	1.18E-01
KYNU	0.224836	2.52E-07	8.24E-07
KY	-0.14276	1.16E-03	2.30E-03
L1CAM	0.19366	9.59E-06	2.57E-05
L1TD1	-0.20027	4.64E-06	1.30E-05
L2HGDH	0.291121	1.62E-11	8.33E-11
L3MBTL2	-0.19826	5.80E-06	1.60E-05
L3MBTL3	0.226824	1.96E-07	6.49E-07
L3MBTL4	-0.1979	6.04E-06	1.67E-05
L3MBTL	-0.1919	1.16E-05	3.08E-05
LACE1	0.069462	1.15E-01	1.56E-01
LACRT	-0.10065	2.23E-02	3.54E-02
LACTB2	0.02164	6.24E-01	6.80E-01
LACTB	0.100137	2.30E-02	3.65E-02
LAD1	-0.07775	7.80E-02	1.10E-01
LAG3	0.211543	1.27E-06	3.80E-06
LAGE3	0.267569	6.82E-10	2.97E-09
LAIR1	-0.0303	4.93E-01	5.57E-01
LAIR2	0.064796	1.42E-01	1.88E-01
LALBA	0.053811	2.23E-01	2.80E-01
LAMA1	0.200818	4.37E-06	1.23E-05
LAMA2	-0.39023	3.53E-20	4.19E-19
LAMA3	-0.01897	6.68E-01	7.19E-01
LAMA4	-0.01698	7.01E-01	7.49E-01
LAMA5	-0.0199	6.52E-01	7.06E-01
LAMB1	0.157216	3.42E-04	7.36E-04

LAMB2L	-0.30958	6.68E-13	4.00E-12
LAMB2	-0.40555	8.27E-22	1.12E-20
LAMB3	0.088348	4.51E-02	6.71E-02
LAMB4	0.00734	8.68E-01	8.92E-01
LAMC1	0.136032	1.98E-03	3.79E-03
LAMC2	0.243706	2.12E-08	7.78E-08
LAMC3	-0.17031	1.03E-04	2.39E-04
LAMP1	-0.18169	3.36E-05	8.35E-05
LAMP2	0.027167	5.38E-01	6.01E-01
LAMP3	-0.28335	5.78E-11	2.80E-10
LANCL1	0.079095	7.29E-02	1.04E-01
LANCL2	0.098467	2.54E-02	3.99E-02
LANCL3	-0.34709	5.02E-16	4.06E-15
LAP3	0.196235	7.25E-06	1.98E-05
LAPTM4A	-0.14945	6.68E-04	1.38E-03
LAPTM4B	0.027682	5.31E-01	5.94E-01
LAPTM5	0.012818	7.72E-01	8.10E-01
LARGE	-0.40502	9.44E-22	1.27E-20
LARP1B	0.181855	3.30E-05	8.22E-05
LARP1	0.18978	1.45E-05	3.80E-05
LARP4B	-0.19081	1.30E-05	3.43E-05
LARP4	0.345687	6.69E-16	5.34E-15
LARP6	0.241474	2.87E-08	1.04E-07
LARP7	0.019344	6.61E-01	7.14E-01
LARS2	0.169444	1.12E-04	2.58E-04
LARS	-0.08295	6.00E-02	8.71E-02
LAS1L	0.20351	3.22E-06	9.20E-06
LASP1	0.154636	4.28E-04	9.09E-04
LASS1	0.172823	8.08E-05	1.91E-04
LASS2	-0.2499	8.99E-09	3.45E-08
LASS3	0.073738	9.46E-02	1.31E-01
LASS4	-0.28556	4.04E-11	1.99E-10
LASS5	0.099549	2.39E-02	3.76E-02
LASS6	0.253179	5.65E-09	2.22E-08
LAT2	0.010103	8.19E-01	8.51E-01
LATS1	0.102215	2.03E-02	3.25E-02
LATS2	-0.13005	3.11E-03	5.78E-03
LAT	-0.05129	2.45E-01	3.05E-01
LAX1	-0.04291	3.31E-01	3.97E-01
LAYN	0.02076	6.38E-01	6.93E-01
LBH	-0.28573	3.93E-11	1.94E-10
LBP	0.213928	9.59E-07	2.91E-06
LBR	0.380422	3.53E-19	3.81E-18
LBX1	0.007827	8.59E-01	8.85E-01

LBX2	0.122793	5.26E-03	9.41E-03
LBXCOR1	-0.2955	7.76E-12	4.13E-11
LCA5L	-0.31691	1.77E-13	1.12E-12
LCA5	-0.30768	9.38E-13	5.52E-12
LCAT	-0.35915	4.00E-17	3.63E-16
LCE1B	-5.72E-05	9.99E-01	9.99E-01
LCE1C	0.025674	5.61E-01	6.22E-01
LCE1E	0.048212	2.75E-01	3.37E-01
LCE1F	0.056808	1.98E-01	2.53E-01
LCE2A	-0.01003	8.20E-01	8.52E-01
LCE2B	0.013043	7.68E-01	8.07E-01
LCE2C	0.041416	3.48E-01	4.14E-01
LCE2D	0.077699	7.81E-02	1.11E-01
LCE3A	0.05458	2.16E-01	2.73E-01
LCE3D	0.163175	2.00E-04	4.47E-04
LCE3E	0.139746	1.48E-03	2.89E-03
LCE5A	0.048848	2.69E-01	3.30E-01
LCK	0.034273	4.38E-01	5.04E-01
LCLAT1	0.397976	5.42E-21	6.91E-20
LCMT1	0.014288	7.46E-01	7.89E-01
LCMT2	-0.14714	8.10E-04	1.65E-03
LCN10	-0.23578	6.15E-08	2.15E-07
LCN12	-0.23453	7.26E-08	2.51E-07
LCN15	0.092931	3.50E-02	5.33E-02
LCN1	0.169962	1.06E-04	2.47E-04
LCN2	-0.04968	2.60E-01	3.22E-01
LCN6	-0.37495	1.23E-18	1.27E-17
LCN8	0.032665	4.59E-01	5.26E-01
LCN9	-0.07407	9.31E-02	1.29E-01
LCNL1	-0.22902	1.48E-07	4.98E-07
LCORL	0.113458	9.97E-03	1.69E-02
LCOR	0.076983	8.09E-02	1.14E-01
LCP1	-0.00244	9.56E-01	9.65E-01
LCP2	0.007763	8.60E-01	8.86E-01
LCTL	0.30475	1.57E-12	9.08E-12
LCT	-0.07188	1.03E-01	1.42E-01
LDB1	-0.45299	2.01E-27	4.31E-26
LDB2	-0.36744	6.60E-18	6.35E-17
LDB3	-0.35293	1.49E-16	1.27E-15
LDHAL6A	-0.05086	2.49E-01	3.10E-01
LDHAL6B	0.071042	1.07E-01	1.47E-01
LDHA	0.469834	1.22E-29	3.08E-28
LDHB	0.266245	8.33E-10	3.59E-09
LDHC	0.11617	8.32E-03	1.43E-02

LDHD	-0.47293	4.65E-30	1.20E-28
LDLRAD1	-0.08116	6.57E-02	9.47E-02
LDLRAD2	-0.40399	1.22E-21	1.64E-20
LDLRAD3	0.251079	7.61E-09	2.94E-08
LDLRAP1	-0.37612	9.44E-19	9.80E-18
LDLR	-0.06988	1.13E-01	1.54E-01
LDOC1L	-0.0985	2.54E-02	3.98E-02
LDOC1	-0.09134	3.82E-02	5.78E-02
LEAP2	-0.15488	4.19E-04	8.90E-04
LECT1	-0.21993	4.64E-07	1.47E-06
LECT2	0.098738	2.50E-02	3.93E-02
LEF1	-0.02575	5.60E-01	6.21E-01
LEFTY1	-0.05093	2.49E-01	3.09E-01
LEFTY2	-0.46159	1.54E-28	3.56E-27
LEKR1	-0.0873	4.77E-02	7.06E-02
LELP1	-0.05838	1.86E-01	2.39E-01
LEMD1	-0.17445	6.90E-05	1.65E-04
LEMD2	0.049758	2.60E-01	3.21E-01
LEMD3	0.14471	9.90E-04	1.99E-03
LENEP	-0.02151	6.26E-01	6.82E-01
LENG1	-0.14191	1.24E-03	2.46E-03
LENG8	-0.26722	7.19E-10	3.13E-09
LENG9	-0.11868	7.01E-03	1.22E-02
LEO1	0.332063	1.01E-14	7.17E-14
LEPRE1	0.235572	6.33E-08	2.20E-07
LEPREL1	-0.33379	7.21E-15	5.19E-14
LEPREL2	0.000584	9.89E-01	9.92E-01
LEPROTL1	-0.05132	2.45E-01	3.05E-01
LEPROT	0.040842	3.55E-01	4.21E-01
LEPR	-0.14737	7.95E-04	1.62E-03
LEP	0.146349	8.65E-04	1.75E-03
LETM1	0.218857	5.29E-07	1.66E-06
LETM2	0.132594	2.57E-03	4.83E-03
LETMD1	-0.24918	9.94E-09	3.79E-08
LEUTX	0.077993	7.70E-02	1.09E-01
LFNG	-0.09869	2.51E-02	3.94E-02
LGALS12	-0.05976	1.76E-01	2.28E-01
LGALS13	0.103157	1.92E-02	3.09E-02
LGALS14	0.129299	3.29E-03	6.08E-03
LGALS1	0.213537	1.00E-06	3.04E-06
LGALS2	-0.18065	3.73E-05	9.23E-05
LGALS3BP	-0.20962	1.60E-06	4.72E-06
LGALS3	-0.10019	2.30E-02	3.64E-02
LGALS4	-0.20687	2.20E-06	6.38E-06

LGALS7B	0.096896	2.79E-02	4.33E-02
LGALS7	0.065894	1.35E-01	1.81E-01
LGALS8	-0.0745	9.12E-02	1.27E-01
LGALS9B	-0.11089	1.18E-02	1.98E-02
LGALS9C	-0.19184	1.17E-05	3.09E-05
LGALS9	-0.12999	3.12E-03	5.80E-03
LGI1	-0.05764	1.92E-01	2.45E-01
LGI2	0.149439	6.68E-04	1.38E-03
LGI3	-0.34086	1.78E-15	1.35E-14
LGI4	-0.26249	1.46E-09	6.12E-09
LGMN	-0.15124	5.74E-04	1.20E-03
LGR4	0.061464	1.64E-01	2.14E-01
LGR5	-0.09389	3.32E-02	5.08E-02
LGR6	-0.38663	8.29E-20	9.49E-19
LGSN	-0.04811	2.76E-01	3.38E-01
LGTN	-0.23065	1.20E-07	4.07E-07
LHB	0.008313	8.51E-01	8.78E-01
LHCGR	-0.27309	2.93E-10	1.34E-09
LHFPL1	0.043607	3.23E-01	3.88E-01
LHFPL2	0.19977	4.91E-06	1.37E-05
LHFPL3	-0.36311	1.70E-17	1.59E-16
LHFPL4	-0.0315	4.76E-01	5.41E-01
LHFPL5	0.285945	3.79E-11	1.87E-10
LHFP	-0.35869	4.41E-17	3.99E-16
LHPP	-0.2501	8.73E-09	3.35E-08
LHX1	0.299252	4.09E-12	2.25E-11
LHX2	0.351864	1.87E-16	1.57E-15
LHX3	0.05008	2.57E-01	3.18E-01
LHX4	-0.10401	1.82E-02	2.94E-02
LHX5	0.255831	3.86E-09	1.54E-08
LHX6	-0.10417	1.80E-02	2.92E-02
LHX8	0.175415	6.28E-05	1.51E-04
LHX9	-0.1094	1.30E-02	2.16E-02
LIAS	-0.0505	2.53E-01	3.13E-01
LIFR	-0.26823	6.17E-10	2.70E-09
LIF	0.026881	5.43E-01	6.05E-01
LIG1	0.420889	1.58E-23	2.48E-22
LIG3	0.354703	1.03E-16	8.89E-16
LIG4	-0.14565	9.17E-04	1.85E-03
LILRA1	-0.06414	1.46E-01	1.93E-01
LILRA2	-0.17457	6.82E-05	1.63E-04
LILRA3	0.29571	7.48E-12	3.99E-11
LILRA4	-0.1438	1.07E-03	2.13E-03
LILRA5	0.201639	3.98E-06	1.12E-05

LILRA6	0.049367	2.63E-01	3.25E-01
LILRB1	0.048603	2.71E-01	3.33E-01
LILRB2	0.169195	1.14E-04	2.64E-04
LILRB3	0.051335	2.45E-01	3.05E-01
LILRB4	0.161099	2.42E-04	5.33E-04
LILRB5	-0.09993	2.33E-02	3.69E-02
LILRP2	0.155256	4.06E-04	8.64E-04
LIM2	0.030975	4.83E-01	5.48E-01
LIMA1	-0.05305	2.29E-01	2.88E-01
LIMCH1	-0.36839	5.34E-18	5.18E-17
LIMD1	-0.51037	1.67E-35	5.93E-34
LIMD2	0.10558	1.65E-02	2.69E-02
LIME1	-0.10671	1.54E-02	2.53E-02
LIMK1	0.203517	3.22E-06	9.20E-06
LIMK2	0.003143	9.43E-01	9.56E-01
LIMS1	0.224597	2.60E-07	8.49E-07
LIMS2	-0.41965	2.19E-23	3.41E-22
LIMS3-LOC440895	0.063635	1.49E-01	1.97E-01
LIMS3	0.085551	5.23E-02	7.69E-02
LIN28A	0.234815	6.99E-08	2.42E-07
LIN28B	0.340415	1.94E-15	1.47E-14
LIN37	0.165445	1.62E-04	3.67E-04
LIN52	0.169551	1.10E-04	2.56E-04
LIN54	0.473556	3.81E-30	9.87E-29
LIN7A	0.070164	1.12E-01	1.52E-01
LIN7B	-0.05569	2.07E-01	2.63E-01
LIN7C	0.087508	4.72E-02	6.99E-02
LIN9	0.528814	1.91E-38	8.02E-37
LINGO1	0.013815	7.54E-01	7.96E-01
LINGO2	0.230227	1.27E-07	4.29E-07
LINGO3	-0.05413	2.20E-01	2.77E-01
LINGO4	-0.21939	4.95E-07	1.56E-06
LINS1	-0.08539	5.28E-02	7.75E-02
LIPA	-0.15335	4.79E-04	1.01E-03
LIPC	0.027033	5.40E-01	6.03E-01
LIPE	-0.11062	1.20E-02	2.01E-02
LIPF	-0.17855	4.60E-05	1.13E-04
LIPG	0.178431	4.66E-05	1.14E-04
LIPH	-0.1307	2.96E-03	5.52E-03
LIPI	0.084176	5.63E-02	8.21E-02
LIPJ	-0.13103	2.89E-03	5.39E-03
LIPK	0.294346	9.42E-12	4.98E-11
LIPM	-0.03742	3.97E-01	4.63E-01
LIPN	-0.08474	5.46E-02	7.99E-02

LIPT1	-0.10829	1.39E-02	2.31E-02
LIPT2	0.085709	5.19E-02	7.63E-02
LITAF	-0.15874	2.98E-04	6.49E-04
LIX1L	-0.1219	5.61E-03	9.98E-03
LIX1	0.025022	5.71E-01	6.31E-01
LLGL1	0.168612	1.21E-04	2.78E-04
LLGL2	-0.20342	3.26E-06	9.29E-06
LLPH	0.402825	1.64E-21	2.16E-20
LMAN1L	-0.0627	1.55E-01	2.04E-01
LMAN1	0.269993	4.72E-10	2.10E-09
LMAN2L	0.088932	4.37E-02	6.52E-02
LMAN2	-0.11793	7.38E-03	1.28E-02
LMBR1L	-0.01649	7.09E-01	7.56E-01
LMBR1	0.321016	8.28E-14	5.41E-13
LMBRD1	-0.33214	9.95E-15	7.07E-14
LMBRD2	0.110554	1.21E-02	2.02E-02
LMCD1	-0.2066	2.26E-06	6.57E-06
LMF1	-0.52731	3.38E-38	1.39E-36
LMF2	-0.14228	1.21E-03	2.39E-03
LMLN	-0.03773	3.93E-01	4.60E-01
LMNA	-0.16262	2.10E-04	4.69E-04
LMNB1	0.743298	1.27E-91	1.82E-89
LMNB2	0.608008	2.24E-53	1.65E-51
LMO1	0.192884	1.04E-05	2.78E-05
LMO2	-0.2873	3.04E-11	1.52E-10
LMO3	-0.40439	1.11E-21	1.48E-20
LMO4	-0.10934	1.30E-02	2.17E-02
LMO7	-0.25316	5.67E-09	2.23E-08
LMOD1	-0.41336	1.13E-22	1.64E-21
LMOD2	0.037758	3.93E-01	4.59E-01
LMOD3	-0.32646	2.97E-14	2.02E-13
LMTK2	0.071063	1.07E-01	1.47E-01
LMTK3	-0.16037	2.58E-04	5.66E-04
LMX1A	0.088539	4.46E-02	6.65E-02
LMX1B	-0.0597	1.76E-01	2.28E-01
LNP1	-0.05625	2.03E-01	2.58E-01
LNPEP	0.147364	7.95E-04	1.62E-03
LNX1	-0.15141	5.66E-04	1.18E-03
LNX2	-0.45072	3.92E-27	8.17E-26
LOC100009676	-0.1555	3.97E-04	8.47E-04
LOC100101266	-0.25965	2.22E-09	9.11E-09
LOC100101938	0.096163	2.91E-02	4.50E-02
LOC100124692	-0.00179	9.68E-01	9.75E-01
LOC100125556	0.078189	7.63E-02	1.08E-01

LOC100126784	0.141986	1.23E-03	2.45E-03
LOC100127888	0.141258	1.31E-03	2.58E-03
LOC100128023	-0.01157	7.93E-01	8.29E-01
LOC100128076	0.135315	2.09E-03	3.98E-03
LOC100128164	-0.12284	5.25E-03	9.38E-03
LOC100128191	0.644888	6.98E-62	6.37E-60
LOC100128239	-0.23993	3.54E-08	1.27E-07
LOC100128288	-0.26424	1.13E-09	4.79E-09
LOC100128292	-0.08517	5.34E-02	7.83E-02
LOC100128542	-0.07797	7.71E-02	1.09E-01
LOC100128554	-0.09285	3.52E-02	5.35E-02
LOC100128573	-0.13688	1.85E-03	3.56E-03
LOC100128640	-0.12706	3.88E-03	7.08E-03
LOC100128675	-0.25389	5.10E-09	2.01E-08
LOC100128788	-0.01822	6.80E-01	7.30E-01
LOC100128811	-0.03845	3.84E-01	4.50E-01
LOC100128822	0.003039	9.45E-01	9.57E-01
LOC100128842	-0.18167	3.36E-05	8.36E-05
LOC100128977	0.055228	2.11E-01	2.67E-01
LOC100129034	-0.43821	1.41E-25	2.61E-24
LOC100129055	-0.05901	1.81E-01	2.34E-01
LOC100129066	-0.04481	3.10E-01	3.74E-01
LOC100129387	-0.23759	4.84E-08	1.71E-07
LOC100129534	-0.29217	1.36E-11	7.05E-11
LOC100129550	-0.29929	4.06E-12	2.24E-11
LOC100129637	-0.11343	9.99E-03	1.69E-02
LOC100129716	-0.1203	6.27E-03	1.11E-02
LOC100129726	-0.05118	2.46E-01	3.06E-01
LOC100129935	0.097907	2.63E-02	4.11E-02
LOC100130015	-0.2592	2.37E-09	9.70E-09
LOC100130093	-0.38711	7.40E-20	8.51E-19
LOC100130148	0.127696	3.70E-03	6.78E-03
LOC100130238	0.089014	4.35E-02	6.49E-02
LOC100130264	0.001228	9.78E-01	9.83E-01
LOC100130274	0.06574	1.36E-01	1.82E-01
LOC100130331	0.135493	2.06E-03	3.94E-03
LOC100130386	0.073943	9.37E-02	1.30E-01
LOC100130522	-0.17093	9.68E-05	2.26E-04
LOC100130557	-0.242	2.68E-08	9.71E-08
LOC100130581	-0.16709	1.39E-04	3.18E-04
LOC100130691	-0.00043	9.92E-01	9.94E-01
LOC100130776	0.049029	2.67E-01	3.28E-01
LOC100130872	-0.04297	3.30E-01	3.96E-01
LOC100130932	0.257334	3.11E-09	1.26E-08

LOC100130933	-0.30462	1.61E-12	9.27E-12
LOC100130987	-0.19372	9.53E-06	2.56E-05
LOC100131193	-0.12642	4.06E-03	7.40E-03
LOC100131434	-0.30238	2.38E-12	1.35E-11
LOC100131496	0.026349	5.51E-01	6.12E-01
LOC100131551	0.367024	7.23E-18	6.94E-17
LOC100131691	-0.2171	6.55E-07	2.03E-06
LOC100131726	0.260902	1.85E-09	7.65E-09
LOC100132111	0.024403	5.81E-01	6.40E-01
LOC100132215	-0.04303	3.30E-01	3.95E-01
LOC100132247	-0.20778	1.98E-06	5.78E-06
LOC100132287	-0.13924	1.54E-03	3.00E-03
LOC100132288	-0.1163	8.25E-03	1.42E-02
LOC100132354	0.343975	9.47E-16	7.43E-15
LOC100132707	-0.3539	1.22E-16	1.05E-15
LOC100132724	0.114809	9.11E-03	1.56E-02
LOC100132831	0.12276	5.28E-03	9.43E-03
LOC100132832	-0.19014	1.40E-05	3.66E-05
LOC100133050	-0.05358	2.25E-01	2.83E-01
LOC100133161	-0.13159	2.77E-03	5.18E-03
LOC100133308	0.042812	3.32E-01	3.98E-01
LOC100133331	-0.18362	2.76E-05	6.95E-05
LOC100133469	0.170815	9.79E-05	2.29E-04
LOC100133545	0.026017	5.56E-01	6.17E-01
LOC100133612	-0.01691	7.02E-01	7.50E-01
LOC100133669	-0.07217	1.02E-01	1.40E-01
LOC100133893	-0.01079	8.07E-01	8.40E-01
LOC100133920	0.105475	1.66E-02	2.71E-02
LOC100133957	0.096006	2.94E-02	4.54E-02
LOC100133985	0.2535	5.40E-09	2.13E-08
LOC100133991	0.050073	2.57E-01	3.18E-01
LOC100134229	0.095629	3.00E-02	4.63E-02
LOC100134259	-0.03359	4.47E-01	5.13E-01
LOC100134368	-0.17072	9.88E-05	2.31E-04
LOC100134713	-0.00433	9.22E-01	9.38E-01
LOC100134868	-0.16058	2.53E-04	5.56E-04
LOC100144603	0.196808	6.81E-06	1.86E-05
LOC100144604	-0.25035	8.44E-09	3.25E-08
LOC100170939	-0.13858	1.62E-03	3.14E-03
LOC100188947	-0.03382	4.44E-01	5.10E-01
LOC100188949	-0.17768	5.02E-05	1.22E-04
LOC100189589	-0.1787	4.53E-05	1.11E-04
LOC100190938	-0.01594	7.18E-01	7.65E-01
LOC100190939	-0.12612	4.15E-03	7.55E-03

LOC100190940	0.075979	8.50E-02	1.19E-01
LOC100190986	-0.23737	4.98E-08	1.75E-07
LOC100192378	0.078332	7.57E-02	1.08E-01
LOC100192379	0.019849	6.53E-01	7.07E-01
LOC100192426	0.04973	2.60E-01	3.21E-01
LOC100216001	0.279784	1.02E-10	4.86E-10
LOC100216545	0.038206	3.87E-01	4.53E-01
LOC100233209	-0.03958	3.70E-01	4.36E-01
LOC100240726	-0.18668	2.01E-05	5.17E-05
LOC100240734	0.097171	2.75E-02	4.27E-02
LOC100240735	0.040966	3.54E-01	4.19E-01
LOC100268168	-0.03199	4.69E-01	5.35E-01
LOC100270710	0.059773	1.76E-01	2.28E-01
LOC100270746	-0.10842	1.38E-02	2.29E-02
LOC100270804	-0.25039	8.39E-09	3.23E-08
LOC100271722	-0.29286	1.21E-11	6.32E-11
LOC100271831	-0.01914	6.65E-01	7.17E-01
LOC100271832	0.045985	2.98E-01	3.61E-01
LOC100271836	-0.03119	4.80E-01	5.45E-01
LOC100272146	-0.04498	3.08E-01	3.72E-01
LOC100272216	-0.11949	6.63E-03	1.16E-02
LOC100272217	-0.06401	1.47E-01	1.94E-01
LOC100272228	-0.21337	1.03E-06	3.10E-06
LOC100286793	0.13518	2.11E-03	4.02E-03
LOC100286844	-0.09117	3.86E-02	5.83E-02
LOC100287227	0.081644	6.41E-02	9.26E-02
LOC100287704	-0.00143	9.74E-01	9.80E-01
LOC100287718	-0.27558	1.99E-10	9.20E-10
LOC100288778	-0.1797	4.10E-05	1.01E-04
LOC100289341	-0.08652	4.97E-02	7.34E-02
LOC100302401	0.290011	1.94E-11	9.94E-11
LOC100302640	-0.04963	2.61E-01	3.22E-01
LOC100302650	-0.19967	4.97E-06	1.39E-05
LOC100303728	-0.2279	1.71E-07	5.69E-07
LOC113230	-0.28626	3.60E-11	1.79E-10
LOC115110	-0.34617	6.05E-16	4.86E-15
LOC116437	0.080552	6.78E-02	9.74E-02
LOC121838	-0.13539	2.07E-03	3.96E-03
LOC121952	-0.30587	1.29E-12	7.50E-12
LOC126536	0.013463	7.61E-01	8.01E-01
LOC127841	0.36035	3.09E-17	2.83E-16
LOC134466	-0.19791	6.03E-06	1.66E-05
LOC143188	-0.13997	1.45E-03	2.84E-03
LOC143666	-0.14321	1.12E-03	2.23E-03

LOC144438	0.268956	5.52E-10	2.44E-09
LOC144486	0.257276	3.14E-09	1.27E-08
LOC144571	-0.29969	3.79E-12	2.10E-11
LOC144742	-0.02284	6.05E-01	6.62E-01
LOC144776	0.064353	1.45E-01	1.91E-01
LOC145474	-0.08272	6.07E-02	8.80E-02
LOC145783	0.241817	2.74E-08	9.95E-08
LOC145820	-0.29899	4.27E-12	2.35E-11
LOC145837	-0.24285	2.39E-08	8.70E-08
LOC145845	-0.01958	6.58E-01	7.10E-01
LOC146336	-0.05353	2.25E-01	2.83E-01
LOC146481	0.026489	5.49E-01	6.11E-01
LOC146880	-0.26197	1.58E-09	6.59E-09
LOC147727	-0.22954	1.39E-07	4.67E-07
LOC147804	0.251531	7.14E-09	2.77E-08
LOC148145	-0.2727	3.11E-10	1.41E-09
LOC148189	-0.02076	6.38E-01	6.93E-01
LOC148413	-0.15028	6.23E-04	1.29E-03
LOC148696	-0.37769	6.60E-19	6.93E-18
LOC148709	0.254705	4.54E-09	1.80E-08
LOC148824	0.050299	2.55E-01	3.15E-01
LOC149134	-0.01412	7.49E-01	7.91E-01
LOC149620	-0.45474	1.20E-27	2.60E-26
LOC149837	-0.06019	1.73E-01	2.24E-01
LOC150185	0.053104	2.29E-01	2.87E-01
LOC150197	-0.19034	1.37E-05	3.59E-05
LOC150381	-0.06823	1.22E-01	1.65E-01
LOC150527	-0.0593	1.79E-01	2.31E-01
LOC150568	0.077135	8.03E-02	1.13E-01
LOC150622	-0.31391	3.06E-13	1.90E-12
LOC150776	-0.07811	7.66E-02	1.09E-01
LOC150786	0.241846	2.73E-08	9.91E-08
LOC151009	-0.34325	1.10E-15	8.54E-15
LOC151162	-0.02429	5.82E-01	6.41E-01
LOC151174	-0.01064	8.10E-01	8.42E-01
LOC151534	0.010488	8.12E-01	8.45E-01
LOC151658	0.128486	3.49E-03	6.43E-03
LOC152024	-0.07122	1.06E-01	1.46E-01
LOC152217	0.269035	5.46E-10	2.41E-09
LOC152225	0.338987	2.58E-15	1.94E-14
LOC153328	0.024027	5.86E-01	6.45E-01
LOC153684	-0.35027	2.60E-16	2.16E-15
LOC153910	0.25005	8.80E-09	3.38E-08
LOC154449	0.06796	1.23E-01	1.66E-01

LOC154761	0.130664	2.97E-03	5.53E-03
LOC154822	-0.22836	1.61E-07	5.38E-07
LOC157381	-0.22792	1.71E-07	5.68E-07
LOC157627	0.030184	4.94E-01	5.59E-01
LOC158376	-0.21711	6.54E-07	2.03E-06
LOC158572	-0.2135	1.01E-06	3.05E-06
LOC158696	-0.24863	1.07E-08	4.07E-08
LOC162632	-0.10506	1.71E-02	2.77E-02
LOC168474	-0.17262	8.23E-05	1.94E-04
LOC200030	-0.27366	2.68E-10	1.23E-09
LOC200726	0.106014	1.61E-02	2.63E-02
LOC201651	-0.02597	5.57E-01	6.18E-01
LOC202181	-0.30179	2.64E-12	1.48E-11
LOC202781	-0.09358	3.37E-02	5.16E-02
LOC219347	-0.07246	1.00E-01	1.38E-01
LOC220429	0.074484	9.13E-02	1.27E-01
LOC220594	-0.12108	5.94E-03	1.05E-02
LOC220729	0.185227	2.34E-05	5.96E-05
LOC220930	0.206281	2.35E-06	6.80E-06
LOC221122	-0.13395	2.32E-03	4.39E-03
LOC221442	-0.101	2.19E-02	3.48E-02
LOC221710	0.451395	3.21E-27	6.77E-26
LOC222699	0.30845	8.18E-13	4.85E-12
LOC253039	-0.30279	2.22E-12	1.26E-11
LOC253724	-0.06493	1.41E-01	1.87E-01
LOC254312	-0.05813	1.88E-01	2.41E-01
LOC254559	0.124807	4.56E-03	8.24E-03
LOC255025	0.028938	5.12E-01	5.76E-01
LOC255167	-0.10492	1.72E-02	2.80E-02
LOC256880	-0.02592	5.57E-01	6.19E-01
LOC257358	-0.06591	1.35E-01	1.80E-01
LOC25845	-0.01427	7.47E-01	7.89E-01
LOC26102	-0.10053	2.25E-02	3.57E-02
LOC282997	-0.12512	4.46E-03	8.07E-03
LOC283050	-0.20263	3.56E-06	1.01E-05
LOC283070	-0.36786	6.01E-18	5.80E-17
LOC283174	-0.49071	1.46E-32	4.39E-31
LOC283267	-0.08322	5.91E-02	8.59E-02
LOC283314	-0.15106	5.83E-04	1.21E-03
LOC283332	0.007605	8.63E-01	8.88E-01
LOC283392	-0.06794	1.24E-01	1.66E-01
LOC283404	0.177308	5.21E-05	1.26E-04
LOC283663	-0.23324	8.60E-08	2.95E-07
LOC283731	-0.24123	2.97E-08	1.07E-07

LOC283761	0.125147	4.45E-03	8.06E-03
LOC283856	-0.18679	1.99E-05	5.12E-05
LOC283867	-0.11063	1.20E-02	2.01E-02
LOC283914	-0.10991	1.26E-02	2.10E-02
LOC283922	-0.268	6.39E-10	2.80E-09
LOC283999	0.084692	5.48E-02	8.01E-02
LOC284009	-0.11867	7.02E-03	1.22E-02
LOC284023	-0.32127	7.90E-14	5.17E-13
LOC284100	0.063045	1.53E-01	2.01E-01
LOC284232	0.069314	1.16E-01	1.57E-01
LOC284233	-0.23375	8.04E-08	2.77E-07
LOC284276	-0.3279	2.25E-14	1.55E-13
LOC284379	0.043747	3.22E-01	3.87E-01
LOC284440	-0.45958	2.82E-28	6.41E-27
LOC284441	0.252618	6.12E-09	2.40E-08
LOC284551	0.070071	1.12E-01	1.53E-01
LOC284578	-0.23932	3.84E-08	1.37E-07
LOC284632	-0.06196	1.60E-01	2.10E-01
LOC284661	0.013183	7.65E-01	8.05E-01
LOC284688	0.097738	2.66E-02	4.14E-02
LOC284749	-0.1677	1.32E-04	3.01E-04
LOC284788	-0.06491	1.41E-01	1.87E-01
LOC284798	0.017894	6.85E-01	7.35E-01
LOC284837	-0.32859	1.97E-14	1.36E-13
LOC284900	-0.32408	4.66E-14	3.12E-13
LOC285033	0.075611	8.65E-02	1.21E-01
LOC285045	0.021331	6.29E-01	6.85E-01
LOC285074	0.005788	8.96E-01	9.16E-01
LOC285194	-0.03637	4.10E-01	4.76E-01
LOC285205	0.09365	3.36E-02	5.14E-02
LOC285359	-0.05306	2.29E-01	2.88E-01
LOC285370	0.006582	8.82E-01	9.04E-01
LOC285375	0.080392	6.83E-02	9.81E-02
LOC285401	0.062582	1.56E-01	2.05E-01
LOC285419	-0.27154	3.72E-10	1.67E-09
LOC285456	-0.14049	1.39E-03	2.73E-03
LOC285501	0.064958	1.41E-01	1.87E-01
LOC285548	0.235715	6.21E-08	2.16E-07
LOC285593	-0.29588	7.26E-12	3.89E-11
LOC285627	-0.11826	7.22E-03	1.26E-02
LOC285629	-0.24816	1.15E-08	4.34E-08
LOC285692	0.050973	2.48E-01	3.09E-01
LOC285696	0.19864	5.57E-06	1.54E-05
LOC285733	0.03697	4.02E-01	4.69E-01

LOC285735	-0.08361	5.79E-02	8.43E-02
LOC285740	-0.01892	6.68E-01	7.20E-01
LOC285768	-0.28573	3.93E-11	1.94E-10
LOC285780	-0.11476	9.14E-03	1.56E-02
LOC285796	-0.20813	1.90E-06	5.56E-06
LOC285830	-0.10288	1.95E-02	3.13E-02
LOC285847	-0.0923	3.63E-02	5.50E-02
LOC285954	0.035851	4.17E-01	4.83E-01
LOC286002	0.076789	8.17E-02	1.15E-01
LOC286094	-0.17037	1.02E-04	2.38E-04
LOC286135	0.075308	8.78E-02	1.23E-01
LOC286238	0.117433	7.64E-03	1.32E-02
LOC286359	0.127055	3.88E-03	7.09E-03
LOC286367	-0.28205	7.12E-11	3.44E-10
LOC286467	0.26466	1.06E-09	4.51E-09
LOC29034	0.179682	4.11E-05	1.01E-04
LOC338588	0.071441	1.05E-01	1.44E-01
LOC338651	-0.21749	6.25E-07	1.95E-06
LOC338758	-0.07715	8.03E-02	1.13E-01
LOC338799	-0.16679	1.43E-04	3.26E-04
LOC339047	-0.34017	2.04E-15	1.54E-14
LOC339240	-0.10746	1.47E-02	2.42E-02
LOC339290	-0.16788	1.29E-04	2.97E-04
LOC339524	-0.32979	1.57E-14	1.09E-13
LOC339535	0.108696	1.36E-02	2.25E-02
LOC339568	0.097539	2.69E-02	4.19E-02
LOC339674	0.426328	3.70E-24	6.13E-23
LOC339788	-0.10597	1.61E-02	2.64E-02
LOC340017	0.048025	2.77E-01	3.39E-01
LOC340074	-0.02917	5.09E-01	5.73E-01
LOC340357	0.009114	8.37E-01	8.66E-01
LOC340508	-0.07503	8.90E-02	1.24E-01
LOC341056	0.196422	7.10E-06	1.94E-05
LOC342346	-0.29359	1.07E-11	5.62E-11
LOC344595	-0.0046	9.17E-01	9.34E-01
LOC344967	0.066409	1.32E-01	1.77E-01
LOC347376	-0.03863	3.82E-01	4.48E-01
LOC348021	0.030847	4.85E-01	5.50E-01
LOC348840	0.077067	8.06E-02	1.14E-01
LOC348926	-0.08062	6.76E-02	9.71E-02
LOC349114	-0.07796	7.71E-02	1.09E-01
LOC349196	-0.31764	1.55E-13	9.85E-13
LOC360030	0.062008	1.60E-01	2.09E-01
LOC374443	0.089312	4.28E-02	6.40E-02

LOC374491	0.047999	2.77E-01	3.39E-01
LOC375190	-0.11189	1.11E-02	1.86E-02
LOC387646	0.083466	5.84E-02	8.49E-02
LOC387647	0.017931	6.85E-01	7.35E-01
LOC388152	-0.41769	3.67E-23	5.57E-22
LOC388242	0.001804	9.67E-01	9.75E-01
LOC388387	-0.24321	2.27E-08	8.30E-08
LOC388428	0.080661	6.74E-02	9.69E-02
LOC388588	-0.28977	2.02E-11	1.03E-10
LOC388692	-0.17105	9.57E-05	2.24E-04
LOC388789	0.006181	8.89E-01	9.10E-01
LOC388796	0.202765	3.51E-06	9.97E-06
LOC388946	0.077702	7.81E-02	1.11E-01
LOC388955	0.17368	7.43E-05	1.77E-04
LOC389033	-0.17392	7.26E-05	1.73E-04
LOC389332	0.068073	1.23E-01	1.66E-01
LOC389333	0.186751	2.00E-05	5.13E-05
LOC389458	0.021312	6.29E-01	6.85E-01
LOC389493	0.00039	9.93E-01	9.95E-01
LOC389634	0.04543	3.03E-01	3.67E-01
LOC389705	-0.0608	1.68E-01	2.19E-01
LOC389791	-0.0326	4.60E-01	5.26E-01
LOC390595	-0.21985	4.68E-07	1.48E-06
LOC390858	0.050948	2.48E-01	3.09E-01
LOC391322	-0.0576	1.92E-01	2.46E-01
LOC392196	-0.05863	1.84E-01	2.37E-01
LOC399744	-0.18852	1.66E-05	4.32E-05
LOC399815	0.577973	3.13E-47	1.99E-45
LOC399959	-0.03263	4.60E-01	5.26E-01
LOC400027	-0.25124	7.44E-09	2.88E-08
LOC400043	-0.06019	1.73E-01	2.24E-01
LOC400657	-0.23277	9.14E-08	3.13E-07
LOC400696	0.221467	3.84E-07	1.23E-06
LOC400752	-0.14084	1.35E-03	2.66E-03
LOC400759	0.182107	3.22E-05	8.03E-05
LOC400794	-0.39705	6.79E-21	8.56E-20
LOC400804	-0.15609	3.77E-04	8.08E-04
LOC400891	-0.26776	6.62E-10	2.89E-09
LOC400927	-0.1371	1.82E-03	3.51E-03
LOC400931	-0.29675	6.27E-12	3.38E-11
LOC400940	-0.19908	5.30E-06	1.47E-05
LOC401010	0.242829	2.39E-08	8.72E-08
LOC401052	-0.13004	3.11E-03	5.78E-03
LOC401093	-0.36133	2.50E-17	2.31E-16

LOC401127	-0.1504	6.16E-04	1.28E-03
LOC401387	-0.08781	4.64E-02	6.89E-02
LOC401397	0.095927	2.95E-02	4.56E-02
LOC401431	0.06685	1.30E-01	1.74E-01
LOC401463	-0.21679	6.80E-07	2.10E-06
LOC401588	-0.00778	8.60E-01	8.86E-01
LOC402377	0.122082	5.53E-03	9.86E-03
LOC402644	0.045076	3.07E-01	3.71E-01
LOC407835	0.149031	6.92E-04	1.43E-03
LOC415056	-0.03943	3.72E-01	4.38E-01
LOC440040	-0.06448	1.44E-01	1.91E-01
LOC440173	0.152432	5.18E-04	1.09E-03
LOC440354	-0.09593	2.95E-02	4.56E-02
LOC440356	0.248465	1.10E-08	4.16E-08
LOC440461	-0.06887	1.19E-01	1.60E-01
LOC440563	0.067586	1.26E-01	1.69E-01
LOC440896	-0.18307	2.92E-05	7.32E-05
LOC440905	0.168457	1.22E-04	2.82E-04
LOC440925	-0.05545	2.09E-01	2.65E-01
LOC440944	-0.15886	2.95E-04	6.43E-04
LOC440957	-0.10677	1.54E-02	2.52E-02
LOC441046	0.160049	2.66E-04	5.82E-04
LOC441089	0.412715	1.34E-22	1.93E-21
LOC441177	0.138475	1.63E-03	3.17E-03
LOC441204	-0.21913	5.11E-07	1.61E-06
LOC441208	0.164448	1.78E-04	4.00E-04
LOC441294	-0.05032	2.54E-01	3.15E-01
LOC441454	-0.04043	3.60E-01	4.26E-01
LOC441455	-0.01892	6.68E-01	7.20E-01
LOC441601	0.103644	1.86E-02	3.00E-02
LOC441666	0.160072	2.65E-04	5.81E-04
LOC441869	-0.37773	6.55E-19	6.89E-18
LOC442308	0.234893	6.92E-08	2.40E-07
LOC442421	0.125453	4.35E-03	7.90E-03
LOC442454	0.026095	5.55E-01	6.16E-01
LOC442459	0.098135	2.59E-02	4.06E-02
LOC493754	-0.12241	5.41E-03	9.65E-03
LOC494141	0.026212	5.53E-01	6.14E-01
LOC541471	0.414671	8.06E-23	1.18E-21
LOC541473	-0.10742	1.47E-02	2.43E-02
LOC550112	0.166229	1.51E-04	3.42E-04
LOC550643	0.153285	4.82E-04	1.01E-03
LOC554202	0.255569	4.01E-09	1.60E-08
LOC55908	-0.1315	2.79E-03	5.22E-03

LOC572558	-0.32197	6.92E-14	4.56E-13
LOC595101	-0.0034	9.39E-01	9.52E-01
LOC606724	-0.01216	7.83E-01	8.20E-01
LOC613037	-0.10245	2.00E-02	3.21E-02
LOC619207	-0.26225	1.51E-09	6.34E-09
LOC641298	-0.10137	2.14E-02	3.41E-02
LOC641367	0.156435	3.66E-04	7.85E-04
LOC642587	-0.0152	7.31E-01	7.75E-01
LOC642597	0.044477	3.14E-01	3.78E-01
LOC642826	-0.17516	6.44E-05	1.54E-04
LOC642846	0.279658	1.04E-10	4.95E-10
LOC642852	-0.07812	7.65E-02	1.09E-01
LOC642929	0.032316	4.64E-01	5.30E-01
LOC643008	-0.15829	3.11E-04	6.74E-04
LOC643387	0.072348	1.01E-01	1.39E-01
LOC643486	0.033192	4.52E-01	5.18E-01
LOC643677	0.016342	7.11E-01	7.58E-01
LOC643719	-0.16386	1.88E-04	4.21E-04
LOC643763	-0.03256	4.61E-01	5.27E-01
LOC643837	-0.00192	9.65E-01	9.73E-01
LOC643923	-0.02423	5.83E-01	6.42E-01
LOC643955	0.106127	1.60E-02	2.61E-02
LOC644145	0.096624	2.83E-02	4.40E-02
LOC644165	-0.46285	1.05E-28	2.47E-27
LOC644172	-0.09665	2.83E-02	4.39E-02
LOC644538	-0.09154	3.78E-02	5.72E-02
LOC644669	0.042922	3.31E-01	3.96E-01
LOC644936	0.090277	4.06E-02	6.10E-02
LOC645166	0.376363	8.93E-19	9.30E-18
LOC645323	-0.01419	7.48E-01	7.90E-01
LOC645332	-0.26385	1.19E-09	5.05E-09
LOC645431	-0.11447	9.32E-03	1.59E-02
LOC645676	-0.04376	3.22E-01	3.86E-01
LOC645752	-0.06374	1.49E-01	1.96E-01
LOC646214	0.198956	5.37E-06	1.49E-05
LOC646471	-0.33486	5.85E-15	4.26E-14
LOC646498	-0.04476	3.11E-01	3.75E-01
LOC646627	0.067893	1.24E-01	1.67E-01
LOC646762	-0.08762	4.69E-02	6.95E-02
LOC646813	0.00275	9.50E-01	9.61E-01
LOC646851	-0.18118	3.54E-05	8.77E-05
LOC646982	-0.12237	5.42E-03	9.68E-03
LOC646999	0.125317	4.40E-03	7.97E-03
LOC647121	-0.22445	2.65E-07	8.63E-07

LOC647288	0.051281	2.45E-01	3.05E-01
LOC647309	-0.15949	2.79E-04	6.10E-04
LOC647859	-0.00914	8.36E-01	8.65E-01
LOC647946	0.534286	2.37E-39	1.06E-37
LOC647979	-0.19509	8.22E-06	2.23E-05
LOC648691	0.296738	6.28E-12	3.39E-11
LOC648740	-0.4212	1.46E-23	2.30E-22
LOC649330	0.232166	9.88E-08	3.38E-07
LOC650293	-0.00017	9.97E-01	9.97E-01
LOC650368	-0.16491	1.71E-04	3.84E-04
LOC650623	-0.16543	1.63E-04	3.67E-04
LOC651250	-0.18421	2.59E-05	6.56E-05
LOC652276	0.034533	4.34E-01	5.01E-01
LOC653113	-0.24912	1.00E-08	3.82E-08
LOC653501	-0.36799	5.84E-18	5.64E-17
LOC653544	0.003965	9.28E-01	9.43E-01
LOC653566	0.095894	2.96E-02	4.57E-02
LOC653653	0.084688	5.48E-02	8.01E-02
LOC653786	-0.14536	9.39E-04	1.89E-03
LOC654342	0.237585	4.85E-08	1.71E-07
LOC654433	0.009891	8.23E-01	8.54E-01
LOC678655	0.008754	8.43E-01	8.72E-01
LOC723809	-0.47405	3.26E-30	8.55E-29
LOC723972	0.079586	7.11E-02	1.02E-01
LOC727677	0.036869	4.04E-01	4.70E-01
LOC727896	0.242651	2.45E-08	8.93E-08
LOC727924	0.076175	8.42E-02	1.18E-01
LOC728024	0.035823	4.17E-01	4.84E-01
LOC728190	0.032171	4.66E-01	5.32E-01
LOC728264	-0.36452	1.25E-17	1.18E-16
LOC728276	-0.2001	4.73E-06	1.32E-05
LOC728323	-0.00157	9.72E-01	9.78E-01
LOC728392	-0.39337	1.66E-20	2.02E-19
LOC728554	0.26016	2.06E-09	8.48E-09
LOC728606	-0.05687	1.98E-01	2.52E-01
LOC728613	-0.02318	6.00E-01	6.57E-01
LOC728640	0.07067	1.09E-01	1.49E-01
LOC728643	0.213535	1.01E-06	3.04E-06
LOC728723	-0.16383	1.88E-04	4.22E-04
LOC728743	-0.0746	9.08E-02	1.26E-01
LOC728758	0.249557	9.43E-09	3.61E-08
LOC728819	0.169216	1.14E-04	2.64E-04
LOC728855	0.049157	2.65E-01	3.27E-01
LOC728875	0.139528	1.50E-03	2.93E-03

LOC728989	-0.16608	1.53E-04	3.47E-04
LOC729020	0.39646	7.85E-21	9.85E-20
LOC729082	0.217829	6.00E-07	1.87E-06
LOC729156	-0.10219	2.04E-02	3.26E-02
LOC729176	0.191091	1.26E-05	3.34E-05
LOC729234	-0.25817	2.75E-09	1.12E-08
LOC729375	-0.06371	1.49E-01	1.96E-01
LOC729467	0.05539	2.10E-01	2.65E-01
LOC729603	-0.11011	1.24E-02	2.07E-02
LOC729609	-0.10126	2.15E-02	3.43E-02
LOC729668	0.07465	9.06E-02	1.26E-01
LOC729678	-0.10926	1.31E-02	2.18E-02
LOC729799	-0.31239	4.03E-13	2.46E-12
LOC729991-MEF2B	0.047963	2.77E-01	3.39E-01
LOC729991	-0.05594	2.05E-01	2.61E-01
LOC730101	0.170089	1.05E-04	2.44E-04
LOC730668	-0.06237	1.58E-01	2.06E-01
LOC731779	-0.00721	8.70E-01	8.94E-01
LOC731789	0.269098	5.41E-10	2.39E-09
LOC732275	-0.01586	7.20E-01	7.66E-01
LOC80054	-0.38237	2.25E-19	2.49E-18
LOC80154	-0.43336	5.43E-25	9.56E-24
LOC81691	0.387386	6.93E-20	8.00E-19
LOC84740	-0.18432	2.57E-05	6.50E-05
LOC84856	0.043831	3.21E-01	3.86E-01
LOC84931	0.121813	5.64E-03	1.00E-02
LOC84989	-0.10372	1.85E-02	2.99E-02
LOC90110	-0.38741	6.90E-20	7.97E-19
LOC90246	-0.09696	2.78E-02	4.32E-02
LOC90586	-0.23927	3.87E-08	1.38E-07
LOC90784	-0.06531	1.39E-01	1.85E-01
LOC90834	-0.26379	1.20E-09	5.10E-09
LOC91149	0.185127	2.36E-05	6.01E-05
LOC91316	-0.27103	4.02E-10	1.80E-09
LOC91450	-0.18859	1.65E-05	4.29E-05
LOC91948	0.004887	9.12E-01	9.29E-01
LOC92249	-0.29408	9.84E-12	5.19E-11
LOC92659	0.196389	7.13E-06	1.95E-05
LOC92973	-0.45297	2.02E-27	4.33E-26
LOC93432	-0.01255	7.76E-01	8.14E-01
LOC93622	-0.07835	7.57E-02	1.07E-01
LOC96610	-0.00746	8.66E-01	8.91E-01
LOH12CR1	0.158399	3.08E-04	6.68E-04
LOH12CR2	-0.02814	5.24E-01	5.88E-01

LOH3CR2A	-0.35719	6.07E-17	5.39E-16
LONP1	0.140314	1.41E-03	2.77E-03
LONP2	-0.12448	4.67E-03	8.43E-03
LONRF1	-0.18897	1.58E-05	4.13E-05
LONRF2	-0.2436	2.15E-08	7.89E-08
LONRF3	-0.17775	4.98E-05	1.21E-04
LOR	-0.17457	6.82E-05	1.63E-04
LOXHD1	-0.19588	7.54E-06	2.05E-05
LOXL1	-0.14615	8.79E-04	1.78E-03
LOXL2	0.430864	1.08E-24	1.86E-23
LOXL3	0.184477	2.53E-05	6.40E-05
LOXL4	-0.23593	6.04E-08	2.11E-07
LOX	0.203033	3.40E-06	9.69E-06
LPAL2	-0.10015	2.30E-02	3.64E-02
LPAR1	-0.1267	3.98E-03	7.26E-03
LPAR2	0.005763	8.96E-01	9.16E-01
LPAR3	-0.11682	7.96E-03	1.37E-02
LPAR4	0.173042	7.91E-05	1.87E-04
LPAR5	-0.01711	6.99E-01	7.47E-01
LPAR6	-0.0957	2.99E-02	4.61E-02
LPA	-0.21259	1.12E-06	3.38E-06
LPCAT1	-0.21781	6.01E-07	1.87E-06
LPCAT2	-0.26745	6.95E-10	3.03E-09
LPCAT3	-0.21693	6.69E-07	2.07E-06
LPCAT4	0.045513	3.03E-01	3.66E-01
LPGAT1	0.200606	4.47E-06	1.26E-05
LPHN1	-0.18475	2.46E-05	6.23E-05
LPHN2	-0.08254	6.12E-02	8.88E-02
LPHN3	-0.00828	8.51E-01	8.79E-01
LPIN1	0.049746	2.60E-01	3.21E-01
LPIN2	-0.29979	3.73E-12	2.07E-11
LPIN3	-0.19211	1.13E-05	3.01E-05
LPL	-0.39078	3.10E-20	3.70E-19
LPO	0.105934	1.62E-02	2.64E-02
LPPR1	-0.34277	1.21E-15	9.37E-15
LPPR2	-0.14151	1.28E-03	2.54E-03
LPPR3	-0.09345	3.40E-02	5.19E-02
LPPR4	-0.37833	5.71E-19	6.04E-18
LPPR5	-0.12671	3.98E-03	7.26E-03
LPP	0.053008	2.30E-01	2.88E-01
LPXN	-0.13391	2.33E-03	4.40E-03
LQK1	-0.03033	4.92E-01	5.57E-01
LRAT	-0.25174	6.93E-09	2.70E-08
LRBA	-0.16949	1.11E-04	2.57E-04

LRCH1	0.020143	6.48E-01	7.02E-01
LRCH2	-0.04805	2.76E-01	3.38E-01
LRCH3	0.114685	9.19E-03	1.57E-02
LRCH4	-0.32916	1.77E-14	1.23E-13
LRDD	-0.06905	1.18E-01	1.59E-01
LRFN1	0.019095	6.66E-01	7.17E-01
LRFN2	0.024631	5.77E-01	6.37E-01
LRFN3	0.017897	6.85E-01	7.35E-01
LRFN4	0.316929	1.76E-13	1.12E-12
LRFN5	-0.09359	3.37E-02	5.15E-02
LRG1	-0.23318	8.66E-08	2.98E-07
LRGUK	-0.11462	9.23E-03	1.58E-02
LRIG1	-0.43671	2.14E-25	3.90E-24
LRIG2	-0.06682	1.30E-01	1.74E-01
LRIG3	-0.22004	4.58E-07	1.45E-06
LRIT1	0.083051	5.96E-02	8.66E-02
LRIT2	-0.0258	5.59E-01	6.20E-01
LRIT3	-0.36717	7.00E-18	6.73E-17
LRMP	-0.14266	1.17E-03	2.32E-03
LRP10	-0.08585	5.15E-02	7.58E-02
LRP11	0.124564	4.64E-03	8.38E-03
LRP12	0.335001	5.69E-15	4.14E-14
LRP1B	0.064174	1.46E-01	1.93E-01
LRP1	-0.18715	1.91E-05	4.94E-05
LRP2BP	-0.53684	8.80E-40	4.03E-38
LRP2	-0.1671	1.39E-04	3.17E-04
LRP3	-0.17902	4.39E-05	1.08E-04
LRP4	0.058698	1.84E-01	2.36E-01
LRP5L	-0.26328	1.30E-09	5.48E-09
LRP5	-0.08746	4.73E-02	7.00E-02
LRP6	-0.281	8.43E-11	4.04E-10
LRP8	0.327815	2.29E-14	1.57E-13
LRPAP1	-0.25131	7.37E-09	2.85E-08
LRPPRC	0.457368	5.49E-28	1.22E-26
LRRC10B	-0.18441	2.54E-05	6.44E-05
LRRC10	0.05809	1.88E-01	2.42E-01
LRRC14B	0.091089	3.88E-02	5.85E-02
LRRC14	-0.09952	2.39E-02	3.77E-02
LRRC15	0.134993	2.14E-03	4.07E-03
LRRC16A	0.017379	6.94E-01	7.43E-01
LRRC16B	-0.0514	2.44E-01	3.04E-01
LRRC17	-0.10749	1.47E-02	2.42E-02
LRRC18	-0.30648	1.16E-12	6.76E-12
LRRC19	-0.02504	5.71E-01	6.30E-01

LRRC1	0.35279	1.54E-16	1.31E-15
LRRC20	-0.06347	1.50E-01	1.98E-01
LRRC23	-0.21141	1.29E-06	3.86E-06
LRRC24	-0.04948	2.62E-01	3.24E-01
LRRC25	0.016175	7.14E-01	7.61E-01
LRRC26	-0.05725	1.95E-01	2.49E-01
LRRC27	-0.53593	1.26E-39	5.69E-38
LRRC28	0.109633	1.28E-02	2.13E-02
LRRC29	-0.27095	4.07E-10	1.83E-09
LRRC2	-0.22949	1.40E-07	4.70E-07
LRRC31	-0.33	1.51E-14	1.05E-13
LRRC32	-0.1489	7.00E-04	1.44E-03
LRRC33	-0.10724	1.49E-02	2.45E-02
LRRC34	0.021186	6.31E-01	6.87E-01
LRRC36	-0.45018	4.58E-27	9.48E-26
LRRC37A2	-0.15118	5.77E-04	1.20E-03
LRRC37A3	0.014331	7.46E-01	7.88E-01
LRRC37A4	-0.17807	4.83E-05	1.18E-04
LRRC37A	-0.1986	5.59E-06	1.55E-05
LRRC37B2	0.145105	9.58E-04	1.93E-03
LRRC37B	-0.17399	7.22E-05	1.72E-04
LRRC39	-0.1608	2.48E-04	5.47E-04
LRRC3B	-0.28387	5.31E-11	2.59E-10
LRRC3	-0.19222	1.12E-05	2.98E-05
LRRC40	0.373534	1.70E-18	1.73E-17
LRRC41	-0.14501	9.66E-04	1.94E-03
LRRC42	0.537802	6.06E-40	2.80E-38
LRRC43	-0.145	9.67E-04	1.95E-03
LRRC45	0.062217	1.59E-01	2.08E-01
LRRC46	-0.20345	3.25E-06	9.26E-06
LRRC47	-0.08573	5.18E-02	7.62E-02
LRRC48	-0.39739	6.25E-21	7.92E-20
LRRC49	0.002097	9.62E-01	9.70E-01
LRRC4B	-0.27591	1.89E-10	8.76E-10
LRRC4C	-0.22302	3.16E-07	1.02E-06
LRRC4	-0.25469	4.56E-09	1.81E-08
LRRC50	-0.30719	1.02E-12	6.00E-12
LRRC52	-0.29918	4.14E-12	2.28E-11
LRRC55	-0.20501	2.72E-06	7.81E-06
LRRC56	-0.41897	2.63E-23	4.05E-22
LRRC57	0.089306	4.28E-02	6.40E-02
LRRC58	0.201643	3.98E-06	1.12E-05
LRRC59	0.532764	4.25E-39	1.87E-37
LRRC61	0.215659	7.79E-07	2.40E-06

LRRC66	0.094177	3.26E-02	5.00E-02
LRRC67	-0.24033	3.36E-08	1.21E-07
LRRC69	0.090005	4.12E-02	6.18E-02
LRRC6	-0.19067	1.32E-05	3.48E-05
LRRC70	-0.22841	1.60E-07	5.35E-07
LRRC7	0.148408	7.29E-04	1.50E-03
LRRC8A	0.03086	4.85E-01	5.50E-01
LRRC8B	0.116658	8.05E-03	1.39E-02
LRRC8C	-0.05329	2.27E-01	2.85E-01
LRRC8D	0.168067	1.27E-04	2.92E-04
LRRC8E	-0.21986	4.68E-07	1.48E-06
LRRCC1	0.039175	3.75E-01	4.41E-01
LRRFIP1	-0.17298	7.95E-05	1.88E-04
LRRFIP2	-0.19207	1.14E-05	3.02E-05
LRRIQ1	-0.10262	1.98E-02	3.18E-02
LRRIQ3	0.106966	1.52E-02	2.49E-02
LRRIQ4	0.211044	1.35E-06	4.03E-06
LRRK1	-0.13413	2.29E-03	4.33E-03
LRRK2	-0.43329	5.54E-25	9.74E-24
LRRN1	-0.02331	5.98E-01	6.55E-01
LRRN2	-0.03931	3.73E-01	4.39E-01
LRRN3	-0.3839	1.57E-19	1.77E-18
LRRN4CL	-0.17987	4.03E-05	9.93E-05
LRRN4	-0.26273	1.41E-09	5.92E-09
LRRTM1	-0.15673	3.57E-04	7.66E-04
LRRTM2	-0.11326	1.01E-02	1.71E-02
LRRTM3	0.047861	2.78E-01	3.40E-01
LRRTM4	-0.09931	2.42E-02	3.81E-02
LRSAM1	-0.31503	2.50E-13	1.56E-12
LRTM1	0.008968	8.39E-01	8.68E-01
LRTM2	0.063523	1.50E-01	1.98E-01
LRTOMT	-0.2259	2.21E-07	7.25E-07
LRWD1	0.26598	8.67E-10	3.73E-09
LSAMP	-0.3318	1.06E-14	7.53E-14
LSG1	0.434193	4.31E-25	7.65E-24
LSM10	0.092822	3.52E-02	5.36E-02
LSM11	0.199523	5.05E-06	1.41E-05
LSM12	0.531602	6.63E-39	2.88E-37
LSM14A	0.262776	1.40E-09	5.88E-09
LSM14B	0.005605	8.99E-01	9.19E-01
LSM1	0.280104	9.73E-11	4.63E-10
LSM2	0.329345	1.71E-14	1.19E-13
LSM3	0.295952	7.18E-12	3.85E-11
LSM4	0.244241	1.97E-08	7.27E-08

LSM5	0.466269	3.69E-29	8.96E-28
LSM6	0.310497	5.67E-13	3.42E-12
LSM7	0.178592	4.58E-05	1.12E-04
LSMD1	-0.06437	1.45E-01	1.91E-01
LSP1	-0.13775	1.73E-03	3.34E-03
LSR	0.047566	2.81E-01	3.43E-01
LSS	-0.15181	5.47E-04	1.14E-03
LST-3TM12	-0.00224	9.60E-01	9.68E-01
LST1	-0.14975	6.51E-04	1.35E-03
LTA4H	-0.36247	1.96E-17	1.82E-16
LTA	-0.05945	1.78E-01	2.30E-01
LTB4R2	-0.04017	3.63E-01	4.29E-01
LTB4R	0.03935	3.73E-01	4.39E-01
LTBP1	-0.01848	6.76E-01	7.26E-01
LTBP2	-0.44723	1.08E-26	2.17E-25
LTBP3	-0.41659	4.89E-23	7.34E-22
LTBP4	-0.40139	2.34E-21	3.06E-20
LTBR	0.207036	2.15E-06	6.26E-06
LTB	-0.21093	1.37E-06	4.07E-06
LTC4S	-0.48913	2.48E-32	7.39E-31
LTF	-0.1452	9.51E-04	1.92E-03
LTK	-0.02412	5.85E-01	6.44E-01
LTV1	0.367833	6.05E-18	5.83E-17
LUC7L2	0.156899	3.51E-04	7.56E-04
LUC7L3	-0.2213	3.92E-07	1.25E-06
LUC7L	-0.29359	1.07E-11	5.62E-11
LUM	-0.10597	1.61E-02	2.64E-02
LUZP1	0.072021	1.03E-01	1.41E-01
LUZP2	-0.3879	6.15E-20	7.14E-19
LUZP4	0.084718	5.47E-02	8.00E-02
LUZP6	0.415766	6.06E-23	9.04E-22
LXN	0.039181	3.75E-01	4.41E-01
LY6D	0.047342	2.84E-01	3.46E-01
LY6E	-0.14095	1.34E-03	2.64E-03
LY6G5B	-0.13456	2.21E-03	4.20E-03
LY6G5C	-0.32472	4.13E-14	2.78E-13
LY6G6C	0.103569	1.87E-02	3.02E-02
LY6G6D	-0.03398	4.42E-01	5.08E-01
LY6G6E	-0.01749	6.92E-01	7.41E-01
LY6G6F	-0.08387	5.72E-02	8.33E-02
LY6H	-0.03791	3.91E-01	4.57E-01
LY6K	0.190131	1.40E-05	3.67E-05
LY75	0.013578	7.59E-01	7.99E-01
LY86	-0.25871	2.54E-09	1.04E-08

LY96	0.027566	5.33E-01	5.96E-01
LY9	-0.2014	4.09E-06	1.15E-05
LYAR	0.580151	1.18E-47	7.57E-46
LYG1	0.103185	1.92E-02	3.08E-02
LYG2	0.184237	2.59E-05	6.54E-05
LYL1	-0.27041	4.42E-10	1.97E-09
LYNX1	-0.26464	1.06E-09	4.52E-09
LYN	0.089265	4.29E-02	6.41E-02
LYPD1	0.11352	9.93E-03	1.69E-02
LYPD2	-0.14539	9.36E-04	1.89E-03
LYPD3	0.327548	2.41E-14	1.65E-13
LYPD4	0.098943	2.47E-02	3.89E-02
LYPD5	0.287643	2.87E-11	1.44E-10
LYPD6B	0.157876	3.22E-04	6.98E-04
LYPD6	0.280828	8.67E-11	4.14E-10
LYPLA1	0.25219	6.50E-09	2.54E-08
LYPLA2P1	-0.03367	4.46E-01	5.12E-01
LYPLA2	-0.10852	1.37E-02	2.28E-02
LYPLAL1	-0.14542	9.34E-04	1.88E-03
LYRM1	-0.1747	6.73E-05	1.61E-04
LYRM2	0.08294	6.00E-02	8.71E-02
LYRM4	0.082388	6.17E-02	8.94E-02
LYRM5	-0.0567	1.99E-01	2.54E-01
LYRM7	-0.07714	8.03E-02	1.13E-01
LYSMD1	0.070175	1.12E-01	1.52E-01
LYSMD2	-0.02424	5.83E-01	6.42E-01
LYSMD3	-0.1661	1.53E-04	3.46E-04
LYSMD4	-0.21462	8.83E-07	2.69E-06
LYST	-0.26212	1.54E-09	6.45E-09
LYVE1	0.002459	9.56E-01	9.65E-01
LYZL1	0.110247	1.23E-02	2.06E-02
LYZL2	0.180566	3.76E-05	9.30E-05
LYZL4	-0.00738	8.67E-01	8.92E-01
LYZL6	0.036421	4.09E-01	4.76E-01
LYZ	-0.1041	1.81E-02	2.93E-02
LZIC	0.299654	3.81E-12	2.11E-11
LZTFL1	-0.32375	4.96E-14	3.30E-13
LZTR1	-0.11258	1.06E-02	1.79E-02
LZTS1	0.070825	1.08E-01	1.48E-01
LZTS2	-0.12422	4.76E-03	8.58E-03
M6PR	0.232738	9.17E-08	3.14E-07
MAB21L1	-0.30832	8.37E-13	4.96E-12
MAB21L2	-0.12108	5.94E-03	1.05E-02
MACC1	-0.1913	1.24E-05	3.27E-05

MACF1	-0.27591	1.89E-10	8.76E-10
MACROD1	-0.11692	7.91E-03	1.37E-02
MACROD2	-0.37876	5.17E-19	5.51E-18
MAD1L1	0.051255	2.46E-01	3.06E-01
MAD2L1BP	0.135318	2.09E-03	3.98E-03
MAD2L1	0.867975	4.61E-158	3.68E-155
MAD2L2	0.427637	2.59E-24	4.35E-23
MADCAM1	-0.05762	1.92E-01	2.46E-01
MADD	-0.26276	1.40E-09	5.89E-09
MAEA	0.136774	1.86E-03	3.59E-03
MAEL	1.08E-05	1.00E+00	1.00E+00
MAF1	-0.06983	1.13E-01	1.54E-01
MAFA	0.094256	3.25E-02	4.98E-02
MAFB	0.021056	6.34E-01	6.89E-01
MAFF	-0.03701	4.02E-01	4.68E-01
MAFG	0.147612	7.79E-04	1.59E-03
MAFK	-0.02451	5.79E-01	6.38E-01
MAF	-0.07859	7.48E-02	1.06E-01
MAGEA10	0.245996	1.55E-08	5.77E-08
MAGEA11	0.249462	9.56E-09	3.66E-08
MAGEA12	0.313973	3.03E-13	1.88E-12
MAGEA1	0.260943	1.83E-09	7.61E-09
MAGEA2	0.341138	1.68E-15	1.28E-14
MAGEA3	0.371508	2.67E-18	2.67E-17
MAGEA4	0.259544	2.25E-09	9.24E-09
MAGEA5	0.164549	1.76E-04	3.97E-04
MAGEA6	0.368122	5.67E-18	5.49E-17
MAGEA8	0.186961	1.95E-05	5.03E-05
MAGEA9B	0.272198	3.36E-10	1.52E-09
MAGEB10	0.06591	1.35E-01	1.80E-01
MAGEB16	0.143386	1.10E-03	2.20E-03
MAGEB18	0.1721	8.66E-05	2.03E-04
MAGEB1	0.195262	8.06E-06	2.19E-05
MAGEB2	0.317731	1.52E-13	9.70E-13
MAGEB3	0.162027	2.22E-04	4.93E-04
MAGEB4	0.015975	7.18E-01	7.64E-01
MAGEB6	0.257561	3.01E-09	1.22E-08
MAGEC1	0.294607	9.01E-12	4.78E-11
MAGEC2	0.313361	3.38E-13	2.09E-12
MAGEC3	0.135525	2.05E-03	3.93E-03
MAGED1	-0.03991	3.66E-01	4.32E-01
MAGED2	-0.13031	3.05E-03	5.67E-03
MAGED4B	0.044967	3.08E-01	3.72E-01
MAGED4	0.038818	3.79E-01	4.46E-01

MAGEE1	-0.29135	1.56E-11	8.03E-11
MAGEE2	-0.27151	3.74E-10	1.68E-09
MAGEF1	0.157275	3.40E-04	7.33E-04
MAGEH1	-0.20282	3.49E-06	9.91E-06
MAGEL2	-0.02045	6.43E-01	6.98E-01
MAGI1	-0.39068	3.17E-20	3.78E-19
MAGI2	-0.28677	3.31E-11	1.65E-10
MAGI3	-0.36524	1.07E-17	1.02E-16
MAGIX	-0.19594	7.49E-06	2.04E-05
MAGOHB	0.406993	5.75E-22	7.90E-21
MAGOH	0.369757	3.95E-18	3.89E-17
MAGT1	0.067263	1.27E-01	1.71E-01
MAG	-0.11623	8.29E-03	1.43E-02
MAK16	0.204425	2.91E-06	8.33E-06
MAK	-0.16169	2.29E-04	5.08E-04
MAL2	-0.11851	7.09E-03	1.24E-02
MALAT1	-0.33475	5.97E-15	4.34E-14
MALL	-0.50067	4.99E-34	1.63E-32
MALT1	-0.00935	8.32E-01	8.62E-01
MAL	-0.32758	2.40E-14	1.64E-13
MAMDC2	-0.52492	8.27E-38	3.35E-36
MAMDC4	-0.21204	1.20E-06	3.60E-06
MAML1	-0.19761	6.23E-06	1.72E-05
MAML2	-0.26379	1.20E-09	5.10E-09
MAML3	-0.3423	1.33E-15	1.02E-14
MAMLD1	-0.02297	6.03E-01	6.60E-01
MAMSTR	0.226334	2.09E-07	6.88E-07
MAN1A1	-0.01889	6.69E-01	7.20E-01
MAN1A2	0.098043	2.61E-02	4.08E-02
MAN1B1	0.000452	9.92E-01	9.94E-01
MAN1C1	-0.37168	2.57E-18	2.58E-17
MAN2A1	0.156133	3.76E-04	8.05E-04
MAN2A2	-0.17103	9.59E-05	2.24E-04
MAN2B1	-0.27083	4.15E-10	1.86E-09
MAN2B2	-0.26965	4.97E-10	2.20E-09
MAN2C1	-0.33285	8.66E-15	6.18E-14
MANBAL	0.022807	6.06E-01	6.63E-01
MANBA	-0.04411	3.18E-01	3.82E-01
MANEAL	0.004368	9.21E-01	9.37E-01
MANEA	0.150802	5.96E-04	1.24E-03
MANF	0.12554	4.33E-03	7.85E-03
MANSC1	0.018769	6.71E-01	7.22E-01
MAOA	-0.44674	1.25E-26	2.48E-25
MAOB	-0.42075	1.64E-23	2.57E-22

MAP1A	-0.10836	1.39E-02	2.30E-02
MAP1B	0.136397	1.92E-03	3.69E-03
MAP1D	0.040433	3.60E-01	4.26E-01
MAP1LC3A	-0.32932	1.72E-14	1.19E-13
MAP1LC3B2	0.014018	7.51E-01	7.93E-01
MAP1LC3B	-0.12867	3.44E-03	6.35E-03
MAP1LC3C	-0.14751	7.86E-04	1.60E-03
MAP1S	-0.09258	3.57E-02	5.43E-02
MAP2K1	0.256155	3.69E-09	1.48E-08
MAP2K2	0.129979	3.13E-03	5.80E-03
MAP2K3	-0.14535	9.39E-04	1.89E-03
MAP2K4	-0.01135	7.97E-01	8.33E-01
MAP2K5	-0.19853	5.63E-06	1.56E-05
MAP2K6	0.220736	4.20E-07	1.34E-06
MAP2K7	-0.1645	1.77E-04	3.98E-04
MAP2	0.02017	6.48E-01	7.02E-01
MAP3K10	0.140396	1.40E-03	2.75E-03
MAP3K11	-0.05337	2.27E-01	2.84E-01
MAP3K12	-0.15247	5.17E-04	1.08E-03
MAP3K13	-0.13933	1.53E-03	2.98E-03
MAP3K14	-0.09222	3.64E-02	5.52E-02
MAP3K15	-0.1997	4.95E-06	1.38E-05
MAP3K1	-0.27546	2.02E-10	9.37E-10
MAP3K2	0.201511	4.04E-06	1.14E-05
MAP3K3	-0.43076	1.11E-24	1.91E-23
MAP3K4	-0.05022	2.55E-01	3.16E-01
MAP3K5	-0.08444	5.55E-02	8.11E-02
MAP3K6	-0.3414	1.60E-15	1.22E-14
MAP3K7	0.300666	3.20E-12	1.79E-11
MAP3K8	-0.16629	1.50E-04	3.41E-04
MAP3K9	-0.0084	8.49E-01	8.77E-01
MAP4K1	-0.1017	2.10E-02	3.34E-02
MAP4K2	0.099029	2.46E-02	3.87E-02
MAP4K3	0.272239	3.34E-10	1.51E-09
MAP4K4	0.255487	4.06E-09	1.62E-08
MAP4K5	0.024208	5.84E-01	6.43E-01
MAP4	-0.04413	3.18E-01	3.82E-01
MAP6D1	0.462029	1.35E-28	3.13E-27
MAP6	-0.45049	4.18E-27	8.68E-26
MAP7D1	0.096733	2.82E-02	4.37E-02
MAP7D2	0.08994	4.13E-02	6.20E-02
MAP7D3	0.165323	1.64E-04	3.71E-04
MAP7	0.139709	1.48E-03	2.90E-03
MAP9	-0.18313	2.90E-05	7.28E-05

MAPK10		-0.44498	2.06E-26	4.07E-25
MAPK11		-0.03245	4.62E-01	5.28E-01
MAPK12		0.245451	1.67E-08	6.20E-08
MAPK13		0.047848	2.78E-01	3.40E-01
MAPK14		0.048173	2.75E-01	3.37E-01
MAPK15		-0.37026	3.53E-18	3.49E-17
MAPK1IP1L		0.061263	1.65E-01	2.15E-01
MAPK1		0.006559	8.82E-01	9.04E-01
MAPK3		-0.07221	1.02E-01	1.40E-01
MAPK4		-0.13951	1.50E-03	2.94E-03
MAPK6		0.538017	5.57E-40	2.58E-38
MAPK7		-0.0243	5.82E-01	6.41E-01
MAPK8IP1		-0.22597	2.19E-07	7.20E-07
MAPK8IP2		0.157629	3.29E-04	7.12E-04
MAPK8IP3		-0.27281	3.06E-10	1.39E-09
MAPK8		0.307079	1.04E-12	6.11E-12
MAPK9		-0.15704	3.47E-04	7.47E-04
MAPKAP1		0.031521	4.75E-01	5.41E-01
MAPKAPK2		-0.20607	2.41E-06	6.95E-06
MAPKAPK3		-0.14226	1.21E-03	2.40E-03
MAPKAPK5		0.434918	3.53E-25	6.31E-24
MAPKBP1		-0.24222	2.60E-08	9.44E-08
MAPKSP1		-0.05307	2.29E-01	2.88E-01
MAPRE1		0.492102	9.18E-33	2.79E-31
MAPRE2		-0.06914	1.17E-01	1.59E-01
MAPRE3		-0.25167	7.00E-09	2.72E-08
MAPT		-0.13534	2.08E-03	3.98E-03
	10-Mar	-0.12832	3.53E-03	6.50E-03
	11-Mar	0.09765	2.67E-02	4.16E-02
	1-Mar	0.010875	8.06E-01	8.39E-01
	2-Mar	-0.16696	1.41E-04	3.21E-04
	3-Mar	0.082277	6.21E-02	8.99E-02
	4-Mar	-0.02804	5.26E-01	5.89E-01
	5-Mar	0.221565	3.79E-07	1.21E-06
	6-Mar	0.145437	9.33E-04	1.88E-03
	7-Mar	0.222246	3.48E-07	1.12E-06
	8-Mar	-0.10528	1.68E-02	2.74E-02
	9-Mar	-0.21206	1.20E-06	3.59E-06
MARCKSL1		0.306426	1.17E-12	6.82E-12
MARCKS		0.379476	4.38E-19	4.69E-18
MARCO		-0.16757	1.33E-04	3.05E-04
MARK1		0.098374	2.56E-02	4.00E-02
MARK2		0.123269	5.09E-03	9.13E-03
MARK3		0.042221	3.39E-01	4.04E-01

MARK4	0.182533	3.08E-05	7.71E-05
MARS2	0.277727	1.42E-10	6.65E-10
MARS	0.521827	2.61E-37	1.01E-35
MARVELD1	0.061826	1.61E-01	2.11E-01
MARVELD2	-0.23913	3.94E-08	1.40E-07
MARVELD3	0.059231	1.80E-01	2.32E-01
MAS1L	-0.3363	4.40E-15	3.24E-14
MAS1	0.045675	3.01E-01	3.64E-01
MASP1	-0.27274	3.09E-10	1.41E-09
MASP2	-0.34524	7.32E-16	5.82E-15
MAST1	0.320922	8.43E-14	5.50E-13
MAST2	0.244022	2.03E-08	7.47E-08
MAST3	-0.28974	2.03E-11	1.04E-10
MAST4	-0.4147	8.00E-23	1.17E-21
MASTL	0.664044	8.78E-67	8.78E-65
MAT1A	0.209121	1.69E-06	4.99E-06
MAT2A	-0.21758	6.18E-07	1.92E-06
MAT2B	-0.18401	2.65E-05	6.69E-05
MATK	-0.1533	4.81E-04	1.01E-03
MATN1	0.000503	9.91E-01	9.93E-01
MATN2	-0.21745	6.28E-07	1.95E-06
MATN3	-0.1963	7.20E-06	1.96E-05
MATN4	-0.04826	2.74E-01	3.36E-01
MATR3	0.005761	8.96E-01	9.16E-01
MAVS	-0.18844	1.67E-05	4.35E-05
MAX	-0.04049	3.59E-01	4.25E-01
MAZ	0.214796	8.65E-07	2.64E-06
MBD1	0.010259	8.16E-01	8.49E-01
MBD2	0.088861	4.38E-02	6.54E-02
MBD3L1	0.066223	1.33E-01	1.78E-01
MBD3L2	0.013368	7.62E-01	8.02E-01
MBD3L5	0.057653	1.91E-01	2.45E-01
MBD3	0.025414	5.65E-01	6.25E-01
MBD4	0.261885	1.60E-09	6.67E-09
MBD5	-0.11327	1.01E-02	1.71E-02
MBD6	-0.14764	7.77E-04	1.59E-03
MBIP	-0.39771	5.78E-21	7.34E-20
MBL1P	-0.49656	2.04E-33	6.41E-32
MBL2	-0.04271	3.33E-01	3.99E-01
MBLAC1	0.039266	3.74E-01	4.40E-01
MBLAC2	-0.30673	1.11E-12	6.48E-12
MBNL1	-0.22011	4.54E-07	1.44E-06
MBNL2	-0.30255	2.31E-12	1.31E-11
MBNL3	-0.05616	2.03E-01	2.59E-01

MBOAT1	0.022007	6.18E-01	6.75E-01
MBOAT2	0.195977	7.46E-06	2.03E-05
MBOAT4	-0.07551	8.69E-02	1.21E-01
MBOAT7	0.010452	8.13E-01	8.45E-01
MBP	-0.11248	1.06E-02	1.80E-02
MBTD1	-0.07521	8.82E-02	1.23E-01
MBTPS1	-0.18337	2.83E-05	7.11E-05
MBTPS2	0.213733	9.82E-07	2.97E-06
MB	0.157993	3.19E-04	6.91E-04
MC1R	0.184147	2.61E-05	6.60E-05
MC2R	-0.17517	6.43E-05	1.54E-04
MC3R	0.010809	8.07E-01	8.40E-01
MC4R	-0.16379	1.89E-04	4.23E-04
MC5R	-0.31096	5.22E-13	3.16E-12
MCAM	0.18183	3.31E-05	8.24E-05
MCART1	0.155214	4.07E-04	8.67E-04
MCART2	0.166385	1.49E-04	3.38E-04
MCART3P	0.052763	2.32E-01	2.90E-01
MCART6	-0.00115	9.79E-01	9.84E-01
MCAT	-0.07441	9.16E-02	1.27E-01
MCCC1	-0.26442	1.10E-09	4.67E-09
MCCC2	-0.09032	4.05E-02	6.09E-02
MCCD1	-0.07889	7.37E-02	1.05E-01
MCC	-0.26614	8.46E-10	3.65E-09
MCEE	-0.25242	6.29E-09	2.46E-08
MCF2L2	0.362561	1.92E-17	1.79E-16
MCF2L	-0.21139	1.30E-06	3.87E-06
MCF2	0.065421	1.38E-01	1.84E-01
MCFD2	0.283406	5.73E-11	2.78E-10
MCHR1	0.262376	1.48E-09	6.23E-09
MCHR2	0.199256	5.20E-06	1.45E-05
MCL1	-0.10375	1.85E-02	2.98E-02
MCM10	0.853136	4.44E-147	2.16E-144
MCM2	0.752433	4.43E-95	6.46E-93
MCM3APAS	0.090308	4.05E-02	6.09E-02
MCM3AP	-0.14005	1.44E-03	2.83E-03
MCM3	0.537915	5.80E-40	2.68E-38
MCM4	0.758375	2.06E-97	3.16E-95
MCM5	0.549688	5.35E-42	2.71E-40
MCM6	0.823767	1.55E-128	4.30E-126
MCM7	0.637134	5.35E-60	4.59E-58
MCM8	0.582573	3.94E-48	2.56E-46
MCM9	-0.07224	1.02E-01	1.39E-01
MCOLN1	-0.07909	7.29E-02	1.04E-01

MCOLN2	0.027896	5.28E-01	5.91E-01
MCOLN3	0.128352	3.53E-03	6.49E-03
MCPH1	-0.00985	8.24E-01	8.55E-01
MCRS1	0.261877	1.60E-09	6.67E-09
MCTP1	0.047043	2.87E-01	3.49E-01
MCTP2	-0.25557	4.01E-09	1.60E-08
MCTS1	0.175838	6.02E-05	1.45E-04
MDC1	0.21685	6.75E-07	2.09E-06
MDFIC	0.117304	7.70E-03	1.33E-02
MDFI	0.255564	4.02E-09	1.60E-08
MDGA1	-0.1196	6.58E-03	1.15E-02
MDGA2	0.236978	5.25E-08	1.84E-07
MDH1B	-0.13591	1.99E-03	3.82E-03
MDH1	0.440524	7.34E-26	1.39E-24
MDH2	0.283326	5.80E-11	2.81E-10
MDK	0.152115	5.33E-04	1.11E-03
MDM1	0.087771	4.65E-02	6.90E-02
MDM2	-0.01835	6.78E-01	7.28E-01
MDM4	-0.34045	1.93E-15	1.46E-14
MDN1	-0.04788	2.78E-01	3.40E-01
MDP1	0.021951	6.19E-01	6.75E-01
MDS2	-0.15536	4.02E-04	8.57E-04
ME1	0.17103	9.59E-05	2.24E-04
ME2	0.191856	1.16E-05	3.09E-05
ME3	-0.1124	1.07E-02	1.81E-02
MEA1	0.243078	2.31E-08	8.44E-08
MEAF6	-0.20789	1.95E-06	5.71E-06
MECOM	-0.4343	4.18E-25	7.43E-24
MECP2	-0.25739	3.09E-09	1.25E-08
MECR	-0.1619	2.25E-04	4.99E-04
MED10	0.286482	3.48E-11	1.72E-10
MED11	-0.34189	1.44E-15	1.11E-14
MED12L	0.2195	4.89E-07	1.54E-06
MED12	-0.02863	5.17E-01	5.81E-01
MED13L	-0.11899	6.87E-03	1.20E-02
MED13	0.046553	2.92E-01	3.54E-01
MED14	0.232862	9.03E-08	3.10E-07
MED15	-0.05022	2.55E-01	3.16E-01
MED16	-0.17595	5.96E-05	1.44E-04
MED17	0.082915	6.01E-02	8.72E-02
MED18	-0.03406	4.40E-01	5.07E-01
MED19	0.140098	1.44E-03	2.82E-03
MED1	0.179815	4.06E-05	9.98E-05
MED20	0.213069	1.06E-06	3.21E-06

MED21	0.160196	2.62E-04	5.75E-04
MED22	-0.05941	1.78E-01	2.31E-01
MED23	0.011441	7.96E-01	8.31E-01
MED24	0.112953	1.03E-02	1.75E-02
MED25	-0.16543	1.63E-04	3.67E-04
MED26	-0.31145	4.78E-13	2.90E-12
MED27	0.345968	6.31E-16	5.06E-15
MED28	0.312374	4.05E-13	2.47E-12
MED29	-0.16314	2.01E-04	4.48E-04
MED30	0.188535	1.66E-05	4.31E-05
MED31	0.042262	3.38E-01	4.04E-01
MED4	-0.11434	9.41E-03	1.60E-02
MED6	0.347519	4.59E-16	3.72E-15
MED7	-0.15718	3.43E-04	7.39E-04
MED8	0.205321	2.62E-06	7.55E-06
MED9	-0.24522	1.72E-08	6.39E-08
MEF2A	-0.28855	2.48E-11	1.25E-10
MEF2B	0.004445	9.20E-01	9.36E-01
MEF2C	-0.35109	2.20E-16	1.84E-15
MEF2D	-0.26223	1.52E-09	6.36E-09
MEFV	0.123952	4.85E-03	8.73E-03
MEG3	-0.14204	1.23E-03	2.44E-03
MEG8	-0.08755	4.71E-02	6.97E-02
MEGF10	0.101479	2.13E-02	3.38E-02
MEGF11	-0.29446	9.25E-12	4.90E-11
MEGF6	-0.46035	2.24E-28	5.14E-27
MEGF8	-0.07108	1.07E-01	1.46E-01
MEGF9	-0.35344	1.34E-16	1.15E-15
MEI1	-0.08431	5.59E-02	8.15E-02
MEIG1	-0.05693	1.97E-01	2.52E-01
MEIS1	-0.25007	8.77E-09	3.37E-08
MEIS2	-0.13461	2.20E-03	4.19E-03
MEIS3P1	-0.28414	5.09E-11	2.48E-10
MEIS3	0.141398	1.29E-03	2.56E-03
MELK	0.870959	1.96E-160	1.78E-157
MEMO1	0.424548	5.96E-24	9.73E-23
MEN1	0.086943	4.86E-02	7.18E-02
MEOX1	-0.33041	1.39E-14	9.74E-14
MEOX2	-0.34995	2.78E-16	2.30E-15
MEP1A	-0.05716	1.95E-01	2.50E-01
MEP1B	0.12504	4.48E-03	8.11E-03
MEPCE	0.013522	7.60E-01	8.00E-01
MEPE	0.001764	9.68E-01	9.75E-01
MERTK	-0.10515	1.70E-02	2.76E-02

MESDC1	0.13653	1.90E-03	3.65E-03
MESDC2	0.221187	3.97E-07	1.27E-06
MESP1	-0.18487	2.43E-05	6.16E-05
MESP2	-0.01801	6.84E-01	7.33E-01
MESTIT1	-0.09426	3.25E-02	4.98E-02
MEST	0.508521	3.22E-35	1.12E-33
METAP1	0.287279	3.05E-11	1.53E-10
METAP2	0.258661	2.56E-09	1.04E-08
METRNL	0.021906	6.20E-01	6.76E-01
METRN	-0.27782	1.40E-10	6.57E-10
METT10D	-0.29838	4.75E-12	2.60E-11
METT11D1	-0.00133	9.76E-01	9.82E-01
METT5D1	-0.09978	2.35E-02	3.72E-02
METTL10	0.273279	2.84E-10	1.30E-09
METTL11A	0.289585	2.09E-11	1.06E-10
METTL11B	0.121224	5.88E-03	1.04E-02
METTL12	0.008661	8.45E-01	8.73E-01
METTL13	0.065941	1.35E-01	1.80E-01
METTL14	-0.06315	1.52E-01	2.01E-01
METTL1	0.299357	4.01E-12	2.22E-11
METTL2A	0.499602	7.21E-34	2.34E-32
METTL2B	0.423162	8.64E-24	1.39E-22
METTL3	-0.06776	1.25E-01	1.68E-01
METTL4	0.324134	4.61E-14	3.08E-13
METTL5	0.408694	3.74E-22	5.22E-21
METTL6	0.238151	4.49E-08	1.59E-07
METTL7A	-0.48713	4.79E-32	1.40E-30
METTL7B	-0.10263	1.98E-02	3.18E-02
METTL8	0.411534	1.81E-22	2.59E-21
METTL9	0.203237	3.33E-06	9.48E-06
MET	0.047904	2.78E-01	3.40E-01
MEX3A	0.221159	3.99E-07	1.27E-06
MEX3B	0.078932	7.35E-02	1.05E-01
MEX3C	0.138007	1.69E-03	3.28E-03
MEX3D	0.295184	8.18E-12	4.35E-11
MFAP1	0.020931	6.36E-01	6.91E-01
MFAP2	0.136541	1.90E-03	3.65E-03
MFAP3L	-0.0722	1.02E-01	1.40E-01
MFAP3	0.052777	2.32E-01	2.90E-01
MFAP4	-0.52308	1.64E-37	6.46E-36
MFAP5	0.216856	6.75E-07	2.09E-06
MFF	0.221747	3.71E-07	1.19E-06
MFGE8	-0.0558	2.06E-01	2.62E-01
MFHAS1	-0.07581	8.57E-02	1.20E-01

MFI2	0.421129	1.48E-23	2.34E-22
MFN1	0.414042	9.48E-23	1.38E-21
MFN2	0.008007	8.56E-01	8.83E-01
MFNG	-0.26571	9.03E-10	3.88E-09
MFRP	-0.19059	1.33E-05	3.50E-05
MFSD10	-0.25967	2.21E-09	9.07E-09
MFSD11	0.172252	8.53E-05	2.01E-04
MFSD1	0.007828	8.59E-01	8.85E-01
MFSD2A	-0.38142	2.80E-19	3.06E-18
MFSD2B	0.524022	1.16E-37	4.61E-36
MFSD3	-0.02782	5.29E-01	5.92E-01
MFSD4	-0.28632	3.57E-11	1.77E-10
MFSD5	0.120574	6.15E-03	1.09E-02
MFSD6L	-0.10619	1.59E-02	2.60E-02
MFSD6	-0.15657	3.62E-04	7.77E-04
MFSD7	-0.33517	5.50E-15	4.01E-14
MFSD8	-0.06556	1.37E-01	1.83E-01
MFSD9	0.215619	7.83E-07	2.41E-06
MGAM	-0.01494	7.35E-01	7.79E-01
MGAT1	-0.13649	1.91E-03	3.67E-03
MGAT2	0.283337	5.79E-11	2.81E-10
MGAT3	-0.21689	6.72E-07	2.08E-06
MGAT4A	0.073134	9.73E-02	1.34E-01
MGAT4B	0.049121	2.66E-01	3.27E-01
MGAT4C	0.135181	2.11E-03	4.02E-03
MGAT5B	0.27543	2.03E-10	9.41E-10
MGAT5	0.06209	1.59E-01	2.09E-01
MGA	-0.01088	8.05E-01	8.39E-01
MGC12916	0.062574	1.56E-01	2.05E-01
MGC12982	0.147604	7.80E-04	1.59E-03
MGC14436	0.233106	8.74E-08	3.00E-07
MGC15885	0.077927	7.73E-02	1.09E-01
MGC16025	0.120153	6.33E-03	1.12E-02
MGC16121	0.004197	9.24E-01	9.40E-01
MGC16142	0.092674	3.55E-02	5.40E-02
MGC16275	-0.2758	1.92E-10	8.90E-10
MGC16384	-0.06271	1.55E-01	2.04E-01
MGC16703	-0.13226	2.64E-03	4.95E-03
MGC21881	-0.14956	6.61E-04	1.37E-03
MGC23270	-0.06898	1.18E-01	1.60E-01
MGC23284	-0.21368	9.88E-07	2.99E-06
MGC26647	0.129139	3.33E-03	6.15E-03
MGC27382	-0.26671	7.76E-10	3.36E-09
MGC2752	-0.12399	4.84E-03	8.71E-03

MGC2889	0.297029	5.98E-12	3.24E-11
MGC29506	-0.04672	2.90E-01	3.53E-01
MGC34034	0.173335	7.68E-05	1.82E-04
MGC3771	-0.35899	4.14E-17	3.75E-16
MGC42105	-0.03536	4.23E-01	4.89E-01
MGC4473	0.069668	1.14E-01	1.55E-01
MGC45800	0.274052	2.52E-10	1.16E-09
MGC57346	0.266104	8.51E-10	3.67E-09
MGC70857	-0.09772	2.66E-02	4.15E-02
MGC72080	0.305816	1.30E-12	7.56E-12
MGC87042	0.525329	7.11E-38	2.89E-36
MGEA5	-0.24247	2.51E-08	9.15E-08
MGLL	-0.46273	1.09E-28	2.55E-27
MGMT	-0.16517	1.67E-04	3.76E-04
MGP	-0.52838	2.25E-38	9.42E-37
MGRN1	-0.46585	4.20E-29	1.02E-27
MGST1	0.002817	9.49E-01	9.60E-01
MGST2	-0.16705	1.40E-04	3.19E-04
MGST3	0.06725	1.27E-01	1.71E-01
MIA2	-0.06643	1.32E-01	1.77E-01
MIA3	-0.28111	8.28E-11	3.97E-10
MIAT	0.117166	7.78E-03	1.34E-02
MIA	-0.1398	1.47E-03	2.88E-03
MIB1	0.174307	6.99E-05	1.67E-04
MIB2	-0.24872	1.06E-08	4.03E-08
MICAL1	-0.19043	1.36E-05	3.56E-05
MICAL2	-0.16414	1.83E-04	4.11E-04
MICAL3	0.265897	8.78E-10	3.78E-09
MICALCL	-0.29225	1.34E-11	6.96E-11
MICALL1	0.139451	1.51E-03	2.95E-03
MICALL2	-0.1567	3.58E-04	7.69E-04
MICA	-0.13541	2.07E-03	3.96E-03
MICB	0.305722	1.33E-12	7.69E-12
MID1IP1	-0.26627	8.30E-10	3.59E-09
MID1	0.070243	1.11E-01	1.52E-01
MID2	-0.21511	8.32E-07	2.55E-06
MIDN	-0.05052	2.52E-01	3.13E-01
MIER1	0.083674	5.78E-02	8.41E-02
MIER2	0.008534	8.47E-01	8.75E-01
MIER3	0.039716	3.68E-01	4.35E-01
MIF4GD	0.010743	8.08E-01	8.41E-01
MIF	0.312565	3.91E-13	2.40E-12
MIIP	0.024501	5.79E-01	6.39E-01
MIMT1	-0.02922	5.08E-01	5.72E-01

MINA	0.302061	2.52E-12	1.42E-11
MINK1	-0.10428	1.79E-02	2.90E-02
MINPP1	0.260868	1.85E-09	7.69E-09
MIOS	-0.01358	7.59E-01	7.99E-01
MIOX	0.037173	4.00E-01	4.66E-01
MIPEP	-0.15009	6.33E-04	1.31E-03
MIPOL1	0.209743	1.57E-06	4.65E-06
MIP	0.119481	6.64E-03	1.16E-02
MIR155HG	0.026771	5.44E-01	6.07E-01
MIR17HG	-0.06509	1.40E-01	1.86E-01
MIS12	0.067794	1.24E-01	1.67E-01
MITD1	0.247132	1.32E-08	4.97E-08
MITF	-0.0585	1.85E-01	2.38E-01
MIXL1	0.091128	3.87E-02	5.84E-02
MKI67IP	0.418628	2.87E-23	4.41E-22
MKI67	0.772612	2.74E-103	4.61E-101
MKKS	0.117126	7.80E-03	1.35E-02
MKL1	-0.01665	7.06E-01	7.54E-01
MKL2	-0.23632	5.73E-08	2.01E-07
MKLN1	-0.02555	5.63E-01	6.23E-01
MKNK1	-0.12257	5.35E-03	9.55E-03
MKNK2	-0.20661	2.26E-06	6.57E-06
MKRN1	-0.08351	5.83E-02	8.47E-02
MKRN2	0.059705	1.76E-01	2.28E-01
MKRN3	0.247866	1.19E-08	4.51E-08
MKS1	-0.01685	7.03E-01	7.51E-01
MKX	0.169289	1.13E-04	2.62E-04
MLANA	-0.11852	7.09E-03	1.24E-02
MLC1	-0.21781	6.01E-07	1.87E-06
MLEC	-0.06366	1.49E-01	1.97E-01
MLF1IP	0.766613	9.22E-101	1.49E-98
MLF1	0.127118	3.86E-03	7.06E-03
MLF2	0.256915	3.30E-09	1.33E-08
MLH1	0.038667	3.81E-01	4.48E-01
MLH3	-0.11199	1.10E-02	1.85E-02
MLKL	0.074002	9.34E-02	1.30E-01
MLL2	-0.10448	1.77E-02	2.87E-02
MLL3	-0.18674	2.00E-05	5.14E-05
MLL4	0.039436	3.72E-01	4.38E-01
MLL5	-0.22617	2.13E-07	7.03E-07
MLLT10	-0.05097	2.48E-01	3.09E-01
MLLT11	0.433529	5.18E-25	9.12E-24
MLLT1	-0.22309	3.14E-07	1.01E-06
MLLT3	-0.26939	5.17E-10	2.29E-09

MLLT4	-0.17123	9.41E-05	2.20E-04
MLLT6	-0.22738	1.83E-07	6.06E-07
MLL	-0.11625	8.27E-03	1.42E-02
MLNR	0.085674	5.20E-02	7.64E-02
MLN	0.037556	3.95E-01	4.62E-01
MLPH	-0.4332	5.67E-25	9.94E-24
MLST8	0.006363	8.85E-01	9.07E-01
MLXIPL	-0.09147	3.80E-02	5.74E-02
MLXIP	0.031537	4.75E-01	5.41E-01
MLX	0.117739	7.48E-03	1.30E-02
MLYCD	-0.41788	3.49E-23	5.32E-22
MMAA	-0.22218	3.51E-07	1.13E-06
MMAB	-0.07904	7.31E-02	1.04E-01
MMACHC	0.111667	1.12E-02	1.89E-02
MMADHC	0.339514	2.33E-15	1.75E-14
MMD2	0.118042	7.33E-03	1.27E-02
MMD	0.519347	6.51E-37	2.49E-35
MMEL1	-0.29271	1.24E-11	6.47E-11
MME	0.104934	1.72E-02	2.79E-02
MMGT1	0.028585	5.17E-01	5.82E-01
MMP10	0.1303	3.05E-03	5.68E-03
MMP11	0.18667	2.01E-05	5.17E-05
MMP12	0.462765	1.08E-28	2.52E-27
MMP13	0.046059	2.97E-01	3.60E-01
MMP14	0.318523	1.32E-13	8.42E-13
MMP15	-0.35972	3.54E-17	3.23E-16
MMP16	-0.05682	1.98E-01	2.53E-01
MMP17	0.039532	3.71E-01	4.37E-01
MMP19	-0.22104	4.05E-07	1.29E-06
MMP1	0.248932	1.03E-08	3.92E-08
MMP20	0.134234	2.27E-03	4.30E-03
MMP21	-0.24814	1.15E-08	4.34E-08
MMP23A	-0.14639	8.62E-04	1.75E-03
MMP23B	-0.301	3.02E-12	1.69E-11
MMP24	-0.36744	6.60E-18	6.35E-17
MMP25	-0.00144	9.74E-01	9.80E-01
MMP26	0.127748	3.69E-03	6.76E-03
MMP27	-0.02929	5.07E-01	5.71E-01
MMP28	-0.3242	4.55E-14	3.04E-13
MMP2	-0.07728	7.98E-02	1.13E-01
MMP3	0.194785	8.49E-06	2.30E-05
MMP7	-0.04393	3.20E-01	3.84E-01
MMP8	0.117019	7.85E-03	1.36E-02
MMP9	0.215371	8.07E-07	2.47E-06

MMRN1	-0.34584	6.48E-16	5.19E-15
MMRN2	-0.26991	4.78E-10	2.12E-09
MMS19	-0.0652	1.40E-01	1.85E-01
MN1	0.02118	6.32E-01	6.87E-01
MNAT1	0.219159	5.10E-07	1.60E-06
MND1	0.756218	1.47E-96	2.25E-94
MNDA	-0.13714	1.81E-03	3.50E-03
MNS1	0.209509	1.62E-06	4.77E-06
MNT	-0.17393	7.25E-05	1.73E-04
MNX1	-0.06964	1.14E-01	1.55E-01
MOAP1	-0.43592	2.67E-25	4.83E-24
MOB2	-0.40489	9.75E-22	1.31E-20
MOBKL1A	-0.14113	1.32E-03	2.61E-03
MOBKL1B	0.489602	2.11E-32	6.32E-31
MOBKL2A	-0.04233	3.38E-01	4.03E-01
MOBKL2B	-0.02003	6.50E-01	7.04E-01
MOBKL2C	-0.29014	1.90E-11	9.73E-11
MOBKL3	0.294237	9.60E-12	5.07E-11
MOBP	-0.08974	4.18E-02	6.26E-02
MOCOS	0.341063	1.71E-15	1.30E-14
MOCS1	-0.43623	2.45E-25	4.44E-24
MOCS2	-0.12767	3.71E-03	6.80E-03
MOCS3	0.183778	2.71E-05	6.84E-05
MOGAT1	0.006067	8.91E-01	9.11E-01
MOGAT2	-0.13709	1.82E-03	3.51E-03
MOGAT3	0.096814	2.80E-02	4.35E-02
MOGS	0.156731	3.57E-04	7.67E-04
MOG	0.036109	4.14E-01	4.80E-01
MON1A	-0.22348	2.99E-07	9.69E-07
MON1B	-0.08904	4.34E-02	6.48E-02
MON2	-0.03115	4.81E-01	5.46E-01
MORC1	0.097617	2.67E-02	4.17E-02
MORC2	0.08259	6.11E-02	8.85E-02
MORC3	-0.1693	1.13E-04	2.62E-04
MORC4	0.215112	8.32E-07	2.55E-06
MORF4L1	0.04832	2.74E-01	3.36E-01
MORF4L2	0.343727	9.96E-16	7.79E-15
MORF4	0.066786	1.30E-01	1.74E-01
MORN1	-0.25952	2.26E-09	9.27E-09
MORN2	0.076404	8.32E-02	1.17E-01
MORN3	-0.06869	1.19E-01	1.61E-01
MORN4	-0.20293	3.44E-06	9.80E-06
MORN5	-0.22073	4.20E-07	1.34E-06
MOSC1	-0.22161	3.77E-07	1.21E-06

MOSC2	-0.45899	3.38E-28	7.65E-27
MOSPD1	-0.03835	3.85E-01	4.52E-01
MOSPD2	0.029489	5.04E-01	5.69E-01
MOSPD3	-0.10362	1.87E-02	3.01E-02
MOS	0.137078	1.82E-03	3.51E-03
MOV10L1	0.061147	1.66E-01	2.16E-01
MOV10	0.208437	1.83E-06	5.38E-06
MOXD1	-0.16999	1.06E-04	2.46E-04
MPDU1	-0.10329	1.90E-02	3.06E-02
MPDZ	-0.09386	3.32E-02	5.08E-02
MPEG1	-0.14027	1.42E-03	2.78E-03
MPG	-0.15752	3.33E-04	7.18E-04
MPHOSPH10	0.360069	3.28E-17	3.01E-16
MPHOSPH6	0.330865	1.27E-14	8.95E-14
MPHOSPH8	-0.29787	5.18E-12	2.82E-11
MPHOSPH9	0.529389	1.54E-38	6.51E-37
MPI	-0.10539	1.67E-02	2.72E-02
MPL	-0.35234	1.69E-16	1.43E-15
MPND	-0.24383	2.09E-08	7.67E-08
MPO	0.09024	4.07E-02	6.11E-02
MPP1	0.094708	3.16E-02	4.86E-02
MPP2	0.31021	5.97E-13	3.59E-12
MPP3	-0.05122	2.46E-01	3.06E-01
MPP4	0.055459	2.09E-01	2.65E-01
MPP5	-0.03006	4.96E-01	5.61E-01
MPP6	0.415474	6.54E-23	9.69E-22
MPP7	-0.25757	3.01E-09	1.22E-08
MPPE1	-0.19617	7.30E-06	1.99E-05
MPPED1	-0.02521	5.68E-01	6.28E-01
MPPED2	-0.18591	2.18E-05	5.57E-05
MPRIP	-0.21194	1.21E-06	3.64E-06
MPST	-0.22937	1.42E-07	4.77E-07
MPV17L2	0.069544	1.15E-01	1.56E-01
MPV17L	-0.1883	1.70E-05	4.41E-05
MPV17	-0.00984	8.24E-01	8.55E-01
MPZL1	-0.06452	1.44E-01	1.90E-01
MPZL2	-0.29587	7.28E-12	3.89E-11
MPZL3	0.028208	5.23E-01	5.87E-01
MPZ	-0.01798	6.84E-01	7.34E-01
MR1	-0.27674	1.66E-10	7.72E-10
MRAP2	-0.04376	3.22E-01	3.86E-01
MRAP	-0.01447	7.43E-01	7.86E-01
MRAS	-0.05199	2.39E-01	2.98E-01
MRC1	-0.17203	8.72E-05	2.05E-04

MRC2	-0.17692	5.41E-05	1.31E-04
MRE11A	0.219354	4.98E-07	1.57E-06
MREG	-0.07691	8.12E-02	1.14E-01
MRFAP1L1	-0.15252	5.14E-04	1.08E-03
MRFAP1	0.040965	3.54E-01	4.19E-01
MRGPRD	0.064728	1.42E-01	1.89E-01
MRGPRE	-0.04643	2.93E-01	3.56E-01
MRGPRF	-0.10956	1.29E-02	2.14E-02
MRGPRX1	-0.04552	3.02E-01	3.66E-01
MRGPRX2	-0.15119	5.76E-04	1.20E-03
MRGPRX3	0.17895	4.42E-05	1.08E-04
MRGPRX4	0.134276	2.26E-03	4.29E-03
MRI1	-0.1985	5.65E-06	1.56E-05
MRM1	0.060038	1.74E-01	2.25E-01
MRO	0.176732	5.51E-05	1.33E-04
MRP63	0.00456	9.18E-01	9.34E-01
MRPL10	0.236026	5.96E-08	2.08E-07
MRPL11	0.499469	7.55E-34	2.44E-32
MRPL12	0.425742	4.33E-24	7.14E-23
MRPL13	0.400116	3.20E-21	4.15E-20
MRPL14	-0.00738	8.67E-01	8.92E-01
MRPL15	0.42857	2.01E-24	3.41E-23
MRPL16	0.107604	1.46E-02	2.40E-02
MRPL17	0.291054	1.64E-11	8.42E-11
MRPL18	0.175734	6.08E-05	1.46E-04
MRPL19	0.423747	7.39E-24	1.20E-22
MRPL1	0.254442	4.72E-09	1.87E-08
MRPL20	0.048249	2.74E-01	3.36E-01
MRPL21	0.395644	9.58E-21	1.20E-19
MRPL22	0.185273	2.33E-05	5.93E-05
MRPL23	-0.12147	5.78E-03	1.03E-02
MRPL24	0.049713	2.60E-01	3.21E-01
MRPL27	0.229234	1.44E-07	4.85E-07
MRPL28	0.111837	1.11E-02	1.87E-02
MRPL2	0.237388	4.97E-08	1.75E-07
MRPL30	0.361565	2.38E-17	2.20E-16
MRPL32	0.220669	4.23E-07	1.35E-06
MRPL33	0.21703	6.61E-07	2.05E-06
MRPL34	0.059419	1.78E-01	2.30E-01
MRPL35	0.428529	2.04E-24	3.45E-23
MRPL36	0.281896	7.30E-11	3.52E-10
MRPL37	0.491434	1.15E-32	3.47E-31
MRPL38	0.095746	2.98E-02	4.60E-02
MRPL39	0.244658	1.86E-08	6.88E-08

MRPL3	0.511029	1.32E-35	4.70E-34
MRPL40	0.073734	9.46E-02	1.31E-01
MRPL41	0.032015	4.68E-01	5.34E-01
MRPL42P5	-0.18351	2.79E-05	7.02E-05
MRPL42	0.484367	1.19E-31	3.38E-30
MRPL43	-0.05481	2.14E-01	2.71E-01
MRPL44	0.303353	2.01E-12	1.15E-11
MRPL45	0.303319	2.02E-12	1.16E-11
MRPL46	0.133467	2.40E-03	4.54E-03
MRPL47	0.517188	1.43E-36	5.41E-35
MRPL48	0.228161	1.66E-07	5.51E-07
MRPL49	0.113707	9.81E-03	1.67E-02
MRPL4	0.147362	7.95E-04	1.62E-03
MRPL50	0.181307	3.49E-05	8.67E-05
MRPL51	0.385157	1.17E-19	1.32E-18
MRPL52	0.319901	1.02E-13	6.59E-13
MRPL53	0.119236	6.75E-03	1.18E-02
MRPL54	-0.09586	2.96E-02	4.58E-02
MRPL55	-0.04942	2.63E-01	3.24E-01
MRPL9	0.356108	7.65E-17	6.70E-16
MRPS10	0.430975	1.05E-24	1.81E-23
MRPS11	0.272219	3.35E-10	1.52E-09
MRPS12	0.353236	1.40E-16	1.20E-15
MRPS14	0.123545	4.99E-03	8.97E-03
MRPS15	0.279824	1.02E-10	4.83E-10
MRPS16	0.302461	2.35E-12	1.33E-11
MRPS17	0.41499	7.42E-23	1.09E-21
MRPS18A	0.05559	2.08E-01	2.64E-01
MRPS18B	-0.04884	2.69E-01	3.30E-01
MRPS18C	0.246962	1.35E-08	5.08E-08
MRPS21	-0.00095	9.83E-01	9.87E-01
MRPS22	0.43934	1.03E-25	1.92E-24
MRPS23	0.279105	1.14E-10	5.39E-10
MRPS24	0.290777	1.71E-11	8.80E-11
MRPS25	-0.41556	6.41E-23	9.50E-22
MRPS26	-0.02976	5.00E-01	5.65E-01
MRPS27	-0.01001	8.21E-01	8.52E-01
MRPS28	0.120397	6.23E-03	1.10E-02
MRPS2	0.138739	1.60E-03	3.11E-03
MRPS30	0.485486	8.23E-32	2.37E-30
MRPS31	-0.04982	2.59E-01	3.20E-01
MRPS33	0.243814	2.09E-08	7.67E-08
MRPS34	0.039887	3.66E-01	4.33E-01
MRPS35	0.495068	3.38E-33	1.05E-31

MRPS36	-0.19765	6.21E-06	1.71E-05
MRPS5	0.2613	1.74E-09	7.24E-09
MRPS6	0.019136	6.65E-01	7.17E-01
MRPS7	0.356364	7.24E-17	6.37E-16
MRPS9	0.178454	4.65E-05	1.14E-04
MRRF	0.039803	3.67E-01	4.34E-01
MRS2P2	-0.01464	7.40E-01	7.84E-01
MRS2	0.02549	5.64E-01	6.24E-01
MRTO4	0.417021	4.37E-23	6.60E-22
MRVI1	-0.25542	4.10E-09	1.63E-08
MS4A10	-0.13585	2.00E-03	3.84E-03
MS4A12	0.016976	7.01E-01	7.49E-01
MS4A13	0.090349	4.04E-02	6.08E-02
MS4A14	-0.1584	3.08E-04	6.68E-04
MS4A15	-0.42692	3.15E-24	5.26E-23
MS4A1	-0.19508	8.22E-06	2.23E-05
MS4A2	-0.40023	3.11E-21	4.04E-20
MS4A3	-0.05663	1.99E-01	2.54E-01
MS4A4A	-0.00661	8.81E-01	9.03E-01
MS4A5	0.066596	1.31E-01	1.76E-01
MS4A6A	-0.05089	2.49E-01	3.09E-01
MS4A6E	-0.10547	1.67E-02	2.71E-02
MS4A7	-0.14149	1.28E-03	2.54E-03
MS4A8B	-0.31673	1.83E-13	1.16E-12
MSC	0.14019	1.43E-03	2.80E-03
MSGN1	0.008434	8.49E-01	8.76E-01
MSH2	0.597319	4.09E-51	2.84E-49
MSH3	0.073111	9.74E-02	1.34E-01
MSH4	0.113961	9.64E-03	1.64E-02
MSH5	0.038638	3.82E-01	4.48E-01
MSH6	0.605617	7.30E-53	5.29E-51
MSI1	0.157374	3.37E-04	7.27E-04
MSI2	0.192845	1.05E-05	2.79E-05
MSL1	0.199431	5.10E-06	1.42E-05
MSL2	-0.04635	2.94E-01	3.57E-01
MSL3L2	0.195725	7.67E-06	2.08E-05
MSL3	0.009878	8.23E-01	8.54E-01
MSLNL	-0.2404	3.32E-08	1.19E-07
MSLN	-0.35489	9.89E-17	8.57E-16
MSMB	-0.06347	1.50E-01	1.98E-01
MSMP	-0.00505	9.09E-01	9.27E-01
MSN	-0.25938	2.31E-09	9.45E-09
MSR1	0.006625	8.81E-01	9.03E-01
MSRA	-0.37502	1.21E-18	1.25E-17

MSRB2	-0.06497	1.41E-01	1.87E-01
MSRB3	-0.22601	2.18E-07	7.16E-07
MST1P2	-0.42048	1.76E-23	2.75E-22
MST1P9	-0.47836	8.28E-31	2.26E-29
MST1R	-0.10178	2.09E-02	3.33E-02
MST1	-0.20611	2.40E-06	6.92E-06
MST4	0.176681	5.54E-05	1.34E-04
MSTN	-0.21893	5.24E-07	1.65E-06
MSTO1	0.035844	4.17E-01	4.83E-01
MSTO2P	-0.06614	1.34E-01	1.79E-01
MSX1	0.054918	2.13E-01	2.70E-01
MSX2P1	-0.10582	1.63E-02	2.66E-02
MSX2	0.140851	1.35E-03	2.66E-03
MT1A	0.150562	6.08E-04	1.26E-03
MT1B	0.079382	7.19E-02	1.03E-01
MT1DP	0.137053	1.82E-03	3.52E-03
MT1E	-0.03034	4.92E-01	5.57E-01
MT1F	0.07178	1.04E-01	1.42E-01
MT1G	0.327554	2.41E-14	1.65E-13
MT1H	0.445762	1.65E-26	3.27E-25
MT1IP	-0.13459	2.21E-03	4.19E-03
MT1L	0.130935	2.91E-03	5.43E-03
MT1M	-0.03319	4.52E-01	5.19E-01
MT1X	0.25386	5.13E-09	2.02E-08
MT2A	0.266878	7.57E-10	3.29E-09
MT3	0.055992	2.05E-01	2.60E-01
MTA1	0.103977	1.83E-02	2.95E-02
MTA2	0.421845	1.23E-23	1.94E-22
MTA3	0.261846	1.61E-09	6.70E-09
MTAP	0.064327	1.45E-01	1.92E-01
MTBP	0.617187	2.18E-55	1.66E-53
MTCH1	0.078285	7.59E-02	1.08E-01
MTCH2	0.425628	4.46E-24	7.35E-23
MTCP1NB	-0.00482	9.13E-01	9.30E-01
MTCP1	-0.0199	6.52E-01	7.06E-01
MTDH	0.195522	7.84E-06	2.13E-05
MTERFD1	0.33085	1.28E-14	8.97E-14
MTERFD2	-0.21552	7.93E-07	2.43E-06
MTERFD3	-0.13888	1.58E-03	3.08E-03
MTERF	0.298302	4.81E-12	2.63E-11
MTF1	-0.06097	1.67E-01	2.18E-01
MTF2	0.291337	1.56E-11	8.05E-11
MTFMT	-0.12076	6.07E-03	1.07E-02
MTFR1	0.331462	1.13E-14	8.02E-14

MTG1	0.083807	5.74E-02	8.35E-02
MTHFD1L	0.468896	1.64E-29	4.08E-28
MTHFD1	0.48052	4.14E-31	1.14E-29
MTHFD2L	0.042478	3.36E-01	4.01E-01
MTHFD2	0.728044	3.68E-86	4.84E-84
MTHFR	-0.3917	2.48E-20	3.00E-19
MTHFSD	-0.24323	2.26E-08	8.28E-08
MTHFS	0.062424	1.57E-01	2.06E-01
MTIF2	0.525468	6.74E-38	2.75E-36
MTIF3	-0.18645	2.06E-05	5.28E-05
MTL5	0.544709	3.97E-41	1.92E-39
MTM1	-0.22498	2.48E-07	8.10E-07
MTMR10	-0.44218	4.59E-26	8.88E-25
MTMR11	0.142986	1.14E-03	2.27E-03
MTMR12	-0.22765	1.77E-07	5.87E-07
MTMR14	-0.09469	3.17E-02	4.87E-02
MTMR15	-0.06147	1.64E-01	2.14E-01
MTMR1	0.007923	8.58E-01	8.84E-01
MTMR2	0.29474	8.81E-12	4.67E-11
MTMR3	-0.2097	1.58E-06	4.68E-06
MTMR4	0.073295	9.66E-02	1.34E-01
MTMR6	-0.08811	4.57E-02	6.79E-02
MTMR7	0.028386	5.20E-01	5.84E-01
MTMR8	-0.05103	2.48E-01	3.08E-01
MTMR9L	-0.1493	6.76E-04	1.40E-03
MTMR9	-0.09414	3.27E-02	5.01E-02
MTNR1A	-0.12062	6.13E-03	1.08E-02
MTNR1B	0.142505	1.18E-03	2.35E-03
MTO1	0.077845	7.76E-02	1.10E-01
MTOR	0.125237	4.42E-03	8.01E-03
MTP18	0.323629	5.07E-14	3.38E-13
MTPAP	0.303157	2.08E-12	1.19E-11
MTRF1L	0.295959	7.17E-12	3.84E-11
MTRF1	-0.02043	6.44E-01	6.98E-01
MTRR	-0.15314	4.88E-04	1.02E-03
MTR	-0.3607	2.87E-17	2.63E-16
MTSS1L	-0.16114	2.41E-04	5.32E-04
MTSS1	-0.24417	1.99E-08	7.33E-08
MTTP	-0.11141	1.14E-02	1.92E-02
MTUS1	-0.35774	5.40E-17	4.82E-16
MTUS2	-0.10989	1.26E-02	2.10E-02
MTVR2	-0.08111	6.59E-02	9.49E-02
MTX1	0.256673	3.42E-09	1.37E-08
MTX2	0.167556	1.33E-04	3.05E-04

MTX3	-0.14419	1.03E-03	2.07E-03
MUC12	0.050306	2.54E-01	3.15E-01
MUC13	0.117335	7.69E-03	1.33E-02
MUC15	-0.1692	1.14E-04	2.64E-04
MUC16	0.222658	3.31E-07	1.07E-06
MUC17	-0.03687	4.04E-01	4.70E-01
MUC1	-0.47289	4.70E-30	1.21E-28
MUC20	-0.05707	1.96E-01	2.50E-01
MUC21	-0.30745	9.77E-13	5.75E-12
MUC2	0.103683	1.86E-02	3.00E-02
MUC4	-0.1033	1.90E-02	3.06E-02
MUC5B	-0.08355	5.81E-02	8.46E-02
MUC6	-0.12816	3.58E-03	6.57E-03
MUC7	-0.02096	6.35E-01	6.90E-01
MUCL1	0.087512	4.72E-02	6.99E-02
MUDENG	0.139603	1.49E-03	2.92E-03
MUL1	-0.02902	5.11E-01	5.75E-01
MUM1L1	-0.14608	8.84E-04	1.79E-03
MUM1	-0.21182	1.23E-06	3.69E-06
MURC	0.296973	6.04E-12	3.26E-11
MUS81	0.141793	1.25E-03	2.48E-03
MUSK	-0.4085	3.93E-22	5.47E-21
MUSTN1	-0.47356	3.81E-30	9.87E-29
MUTED	0.108629	1.36E-02	2.26E-02
MUTYH	0.07328	9.67E-02	1.34E-01
MUT	-0.1074	1.48E-02	2.43E-02
MVD	-0.04194	3.42E-01	4.08E-01
MVK	-0.07246	1.00E-01	1.38E-01
MVP	-0.39371	1.53E-20	1.88E-19
MX1	0.069073	1.17E-01	1.59E-01
MX2	-0.08683	4.89E-02	7.22E-02
MXD1	0.298502	4.65E-12	2.55E-11
MXD3	0.301903	2.59E-12	1.46E-11
MXD4	-0.47978	5.26E-31	1.44E-29
MXI1	-0.10042	2.27E-02	3.59E-02
MXRA5	0.184642	2.48E-05	6.30E-05
MXRA7	0.02733	5.36E-01	5.99E-01
MXRA8	-0.16112	2.41E-04	5.33E-04
MYADML2	0.177669	5.03E-05	1.22E-04
MYADML	0.03764	3.94E-01	4.61E-01
MYADM	-0.08394	5.69E-02	8.30E-02
MYBBP1A	0.005365	9.03E-01	9.22E-01
MYBL1	0.481701	2.83E-31	7.88E-30
MYBL2	0.835285	2.27E-135	7.21E-133

MYBPC1	-0.0612	1.65E-01	2.16E-01
MYBPC2	-0.05903	1.81E-01	2.34E-01
MYBPC3	0.001582	9.71E-01	9.78E-01
MYBPHL	-0.3509	2.28E-16	1.91E-15
MYBPH	-0.06926	1.16E-01	1.58E-01
MYB	0.203999	3.05E-06	8.72E-06
MYCBP2	-0.17234	8.46E-05	1.99E-04
MYCBPAP	-0.17058	1.00E-04	2.34E-04
MYCBP	0.073744	9.46E-02	1.31E-01
MYCL1	-0.0842	5.62E-02	8.20E-02
MYCNOS	0.154269	4.42E-04	9.36E-04
MYCN	0.063247	1.52E-01	2.00E-01
MYCT1	-0.28356	5.59E-11	2.72E-10
MYC	0.18424	2.59E-05	6.54E-05
MYD88	-0.01374	7.56E-01	7.97E-01
MYEF2	-0.10467	1.75E-02	2.84E-02
MYEOV2	0.11546	8.73E-03	1.49E-02
MYEOV	0.291243	1.58E-11	8.16E-11
MYF5	0.013219	7.65E-01	8.04E-01
MYF6	0.132965	2.50E-03	4.71E-03
MYH10	-0.25505	4.32E-09	1.72E-08
MYH11	-0.44699	1.16E-26	2.32E-25
MYH13	0.088639	4.44E-02	6.62E-02
MYH14	-0.25844	2.65E-09	1.08E-08
MYH15	0.015764	7.21E-01	7.67E-01
MYH16	0.34583	6.49E-16	5.20E-15
MYH1	-0.39461	1.23E-20	1.52E-19
MYH2	-0.38158	2.70E-19	2.96E-18
MYH3	-0.15116	5.78E-04	1.20E-03
MYH4	-0.09667	2.83E-02	4.39E-02
MYH6	0.182681	3.04E-05	7.60E-05
MYH7B	-0.24273	2.42E-08	8.84E-08
MYH7	0.063111	1.53E-01	2.01E-01
MYH8	-0.06385	1.48E-01	1.95E-01
MYH9	-0.04005	3.64E-01	4.31E-01
MYL10	0.049848	2.59E-01	3.20E-01
MYL12A	-0.0366	4.07E-01	4.74E-01
MYL12B	-0.0002	9.96E-01	9.97E-01
MYL1	0.032243	4.65E-01	5.31E-01
MYL2	-0.01972	6.55E-01	7.08E-01
MYL3	-0.33394	7.00E-15	5.05E-14
MYL4	-0.00807	8.55E-01	8.82E-01
MYL5	-0.25667	3.42E-09	1.37E-08
MYL6B	0.411993	1.61E-22	2.31E-21

MYL6	0.165112	1.67E-04	3.77E-04
MYL7	0.094529	3.20E-02	4.91E-02
MYL9	-0.15619	3.74E-04	8.01E-04
MYLIP	-0.40654	6.45E-22	8.82E-21
MYLK2	0.337524	3.46E-15	2.56E-14
MYLK3	-0.19564	7.74E-06	2.10E-05
MYLK4	-0.33244	9.39E-15	6.68E-14
MYLK	-0.17295	7.98E-05	1.88E-04
MYLPF	-0.20128	4.15E-06	1.17E-05
MYNN	0.264805	1.03E-09	4.41E-09
MYO10	-0.01197	7.86E-01	8.23E-01
MYO15A	-0.30885	7.62E-13	4.53E-12
MYO15B	-0.25446	4.71E-09	1.86E-08
MYO16	-0.02449	5.79E-01	6.39E-01
MYO18A	-0.12761	3.72E-03	6.82E-03
MYO18B	0.145813	9.04E-04	1.83E-03
MYO19	0.537795	6.08E-40	2.80E-38
MYO1A	-0.05036	2.54E-01	3.15E-01
MYO1B	-0.08527	5.31E-02	7.79E-02
MYO1C	-0.25071	8.02E-09	3.09E-08
MYO1D	-0.21173	1.25E-06	3.73E-06
MYO1E	0.239603	3.70E-08	1.32E-07
MYO1F	-0.14024	1.42E-03	2.79E-03
MYO1G	-0.06803	1.23E-01	1.66E-01
MYO1H	-0.14979	6.49E-04	1.34E-03
MYO3A	0.039204	3.75E-01	4.41E-01
MYO3B	0.196612	6.96E-06	1.90E-05
MYO5A	0.060749	1.69E-01	2.19E-01
MYO5B	0.064758	1.42E-01	1.89E-01
MYO5C	-0.33106	1.23E-14	8.64E-14
MYO6	-0.16957	1.10E-04	2.56E-04
MYO7A	0.227949	1.70E-07	5.66E-07
MYO7B	-0.08618	5.06E-02	7.46E-02
MYO9A	-0.26288	1.38E-09	5.80E-09
MYO9B	-0.06416	1.46E-01	1.93E-01
MYOCD	-0.45943	2.96E-28	6.71E-27
MYOC	-0.48184	2.70E-31	7.53E-30
MYOD1	0.17334	7.68E-05	1.82E-04
MYOF	-0.1382	1.67E-03	3.24E-03
MYOG	-0.06591	1.35E-01	1.80E-01
MYOM1	-0.18419	2.60E-05	6.58E-05
MYOM2	-0.36032	3.11E-17	2.85E-16
MYOM3	0.100066	2.31E-02	3.66E-02
MYOT	-0.05962	1.77E-01	2.29E-01

MYOZ1	-0.41297	1.25E-22	1.81E-21
MYOZ2	-0.09956	2.38E-02	3.76E-02
MYOZ3	-0.29409	9.84E-12	5.19E-11
MYPN	0.140699	1.37E-03	2.69E-03
MYPOP	0.157543	3.32E-04	7.17E-04
MYRIP	-0.29239	1.31E-11	6.81E-11
MYSM1	-0.05594	2.05E-01	2.61E-01
MYST1	-0.2774	1.49E-10	6.99E-10
MYST2	-0.16474	1.73E-04	3.90E-04
MYST3	0.048876	2.68E-01	3.30E-01
MYST4	-0.24531	1.70E-08	6.32E-08
MYT1L	0.053536	2.25E-01	2.83E-01
MYT1	-0.03553	4.21E-01	4.87E-01
MZF1	-0.29397	1.00E-11	5.29E-11
N4BP1	-0.34169	1.50E-15	1.15E-14
N4BP2L1	-0.31073	5.44E-13	3.29E-12
N4BP2L2	-0.38121	2.94E-19	3.20E-18
N4BP2	0.180268	3.88E-05	9.57E-05
N4BP3	-0.17953	4.17E-05	1.03E-04
N6AMT1	-0.21345	1.02E-06	3.07E-06
N6AMT2	-0.14785	7.64E-04	1.56E-03
NAA10	0.327892	2.26E-14	1.55E-13
NAA11	0.173638	7.46E-05	1.77E-04
NAA15	0.558837	1.23E-43	6.75E-42
NAA16	-0.21224	1.17E-06	3.52E-06
NAA20	0.241362	2.92E-08	1.06E-07
NAA25	0.434797	3.65E-25	6.52E-24
NAA30	0.093886	3.32E-02	5.08E-02
NAA35	0.35052	2.47E-16	2.06E-15
NAA38	0.368712	4.98E-18	4.85E-17
NAA40	0.207432	2.06E-06	6.00E-06
NAA50	0.538299	4.99E-40	2.33E-38
NAAA	-0.03979	3.68E-01	4.34E-01
NAALAD2	-0.32648	2.95E-14	2.01E-13
NAALADL1	-0.19415	9.10E-06	2.45E-05
NAALADL2	-0.14468	9.92E-04	1.99E-03
NAB1	0.268741	5.71E-10	2.51E-09
NAB2	-0.19257	1.08E-05	2.87E-05
NACA2	0.055376	2.10E-01	2.66E-01
NACAD	-0.05327	2.27E-01	2.85E-01
NACAP1	0.027936	5.27E-01	5.90E-01
NACA	0.083128	5.94E-02	8.63E-02
NACC1	0.191276	1.24E-05	3.28E-05
NACC2	0.037394	3.97E-01	4.64E-01

NADK	-0.01229	7.81E-01	8.18E-01
NADSYN1	-0.15294	4.96E-04	1.04E-03
NAE1	0.26928	5.26E-10	2.32E-09
NAF1	0.121942	5.59E-03	9.95E-03
NAGA	-0.03273	4.59E-01	5.25E-01
NAGK	0.133475	2.40E-03	4.54E-03
NAGLU	-0.1294	3.26E-03	6.04E-03
NAGPA	-0.07809	7.66E-02	1.09E-01
NAGS	-0.06994	1.13E-01	1.53E-01
NAIF1	0.104849	1.73E-02	2.81E-02
NAIP	-0.0816	6.43E-02	9.28E-02
NALCN	-0.33852	2.84E-15	2.11E-14
NAMPT	0.409368	3.15E-22	4.41E-21
NANOG	-0.15458	4.30E-04	9.13E-04
NANOS1	-0.1674	1.35E-04	3.09E-04
NANOS2	0.095981	2.94E-02	4.55E-02
NANOS3	-0.00309	9.44E-01	9.56E-01
NANP	0.225288	2.38E-07	7.80E-07
NANS	0.040785	3.56E-01	4.22E-01
NAP1L1	0.108811	1.35E-02	2.24E-02
NAP1L2	-0.25502	4.34E-09	1.73E-08
NAP1L3	-0.1867	2.01E-05	5.16E-05
NAP1L4	0.059626	1.77E-01	2.29E-01
NAP1L5	-0.18041	3.82E-05	9.44E-05
NAP1L6	-0.06871	1.19E-01	1.61E-01
NAPA	-0.3578	5.34E-17	4.77E-16
NAPB	-0.1899	1.43E-05	3.75E-05
NAPEPLD	-0.09355	3.38E-02	5.16E-02
NAPG	-0.04042	3.60E-01	4.26E-01
NAPRT1	-0.10929	1.31E-02	2.17E-02
NAPSA	-0.54017	2.40E-40	1.14E-38
NAPSB	-0.45975	2.69E-28	6.10E-27
NARFL	-0.21282	1.09E-06	3.29E-06
NARF	0.206316	2.34E-06	6.77E-06
NARG2	-0.01302	7.68E-01	8.07E-01
NARS2	0.159781	2.72E-04	5.96E-04
NARS	0.215272	8.17E-07	2.50E-06
NASP	0.337475	3.49E-15	2.59E-14
NAT10	0.162242	2.18E-04	4.84E-04
NAT14	-0.11582	8.52E-03	1.46E-02
NAT15	-0.37727	7.26E-19	7.62E-18
NAT1	-0.02805	5.25E-01	5.89E-01
NAT2	0.051007	2.48E-01	3.08E-01
NAT6	-0.32749	2.44E-14	1.67E-13

NAT8B	0.062641	1.56E-01	2.04E-01
NAT8L	0.243443	2.20E-08	8.05E-08
NAT8	-0.00248	9.55E-01	9.64E-01
NAT9	0.102518	2.00E-02	3.20E-02
NAV1	0.169607	1.10E-04	2.55E-04
NAV2	-0.05384	2.23E-01	2.80E-01
NAV3	-0.09316	3.46E-02	5.27E-02
NBAS	0.002282	9.59E-01	9.68E-01
NBEAL1	-0.31946	1.11E-13	7.13E-13
NBEAL2	-0.26496	1.01E-09	4.32E-09
NBEA	-0.24725	1.30E-08	4.89E-08
NBL1	-0.01083	8.06E-01	8.40E-01
NBLA00301	-0.05621	2.03E-01	2.58E-01
NBN	0.415713	6.15E-23	9.15E-22
NBPF10	-0.15041	6.16E-04	1.28E-03
NBPF14	-0.11157	1.13E-02	1.90E-02
NBPF15	-0.02378	5.90E-01	6.48E-01
NBPF16	-0.05825	1.87E-01	2.40E-01
NBPF1	-0.15916	2.88E-04	6.27E-04
NBPF22P	0.17422	7.05E-05	1.68E-04
NBPF3	-0.23954	3.73E-08	1.33E-07
NBPF4	0.324991	3.92E-14	2.64E-13
NBPF6	0.24591	1.57E-08	5.84E-08
NBPF7	-0.12022	6.30E-03	1.11E-02
NBPF9	-0.1346	2.21E-03	4.19E-03
NBR1	-0.17998	3.99E-05	9.84E-05
NBR2	-0.1189	6.91E-03	1.21E-02
NCALD	-0.44268	3.99E-26	7.73E-25
NCAM1	-0.11849	7.10E-03	1.24E-02
NCAM2	-0.22667	2.00E-07	6.61E-07
NCAN	0.075553	8.67E-02	1.21E-01
NCAPD2	0.667875	8.31E-68	8.43E-66
NCAPD3	0.530412	1.04E-38	4.45E-37
NCAPG2	0.755864	2.03E-96	3.07E-94
NCAPG	0.90804	5.28E-196	5.28E-192
NCAPH2	0.155625	3.93E-04	8.39E-04
NCAPH	0.880409	2.38E-168	2.98E-165
NCBP1	0.452153	2.57E-27	5.45E-26
NCBP2	0.314195	2.91E-13	1.81E-12
NCCRP1	0.077432	7.92E-02	1.12E-01
NCDN	-0.29902	4.25E-12	2.34E-11
NCEH1	0.224703	2.56E-07	8.38E-07
NCF1B	-0.04531	3.05E-01	3.68E-01
NCF1C	0.022359	6.13E-01	6.70E-01

NCF1	-0.01311	7.67E-01	8.06E-01
NCF2	-0.01673	7.05E-01	7.53E-01
NCF4	-0.13409	2.29E-03	4.34E-03
NCK1	0.301179	2.93E-12	1.64E-11
NCK2	-0.02732	5.36E-01	5.99E-01
NCKAP1L	-0.0813	6.53E-02	9.41E-02
NCKAP1	0.256972	3.28E-09	1.32E-08
NCKAP5L	-0.22435	2.68E-07	8.73E-07
NCKAP5	-0.14185	1.25E-03	2.47E-03
NCKIPSD	-0.03975	3.68E-01	4.34E-01
NCLN	0.100252	2.29E-02	3.62E-02
NCL	0.3261	3.18E-14	2.15E-13
NCOA1	-0.09192	3.70E-02	5.61E-02
NCOA2	-0.1145	9.30E-03	1.59E-02
NCOA3	0.118899	6.91E-03	1.21E-02
NCOA4	-0.14424	1.03E-03	2.06E-03
NCOA5	-0.05561	2.08E-01	2.63E-01
NCOA6	0.100032	2.32E-02	3.67E-02
NCOA7	-0.06009	1.73E-01	2.25E-01
NCOR1	-0.14726	8.02E-04	1.64E-03
NCOR2	-0.14438	1.02E-03	2.04E-03
NCR1	0.158571	3.03E-04	6.58E-04
NCR2	0.001877	9.66E-01	9.74E-01
NCR3	-0.11587	8.49E-03	1.46E-02
NCRNA00028	-0.07944	7.17E-02	1.02E-01
NCRNA00029	0.000284	9.95E-01	9.96E-01
NCRNA00032	-0.10693	1.52E-02	2.50E-02
NCRNA00051	0.071314	1.06E-01	1.45E-01
NCRNA00052	0.180684	3.72E-05	9.20E-05
NCRNA00081	-0.12083	6.04E-03	1.07E-02
NCRNA00085	-0.20983	1.56E-06	4.61E-06
NCRNA00086	-0.28559	4.02E-11	1.98E-10
NCRNA00087	-0.34257	1.26E-15	9.73E-15
NCRNA00092	-0.43578	2.78E-25	5.01E-24
NCRNA00093	-0.2379	4.65E-08	1.64E-07
NCRNA00094	-0.22189	3.64E-07	1.17E-06
NCRNA00095	0.039055	3.76E-01	4.43E-01
NCRNA00099	0.07499	8.91E-02	1.24E-01
NCRNA00105	-0.19381	9.44E-06	2.54E-05
NCRNA00107	-0.27961	1.05E-10	4.99E-10
NCRNA00110	-0.0471	2.86E-01	3.48E-01
NCRNA00111	-0.03929	3.74E-01	4.40E-01
NCRNA00112	0.044231	3.16E-01	3.81E-01
NCRNA00113	-0.05973	1.76E-01	2.28E-01

NCRNA00114	-0.03373	4.45E-01	5.11E-01
NCRNA00115	-0.07217	1.02E-01	1.40E-01
NCRNA00116	0.063487	1.50E-01	1.98E-01
NCRNA00119	0.076279	8.37E-02	1.18E-01
NCRNA00120	0.167954	1.28E-04	2.95E-04
NCRNA00152	0.375491	1.09E-18	1.13E-17
NCRNA00157	0.069899	1.13E-01	1.54E-01
NCRNA00158	0.128972	3.37E-03	6.22E-03
NCRNA00159	0.011984	7.86E-01	8.23E-01
NCRNA00160	-0.15625	3.72E-04	7.98E-04
NCRNA00161	-0.0629	1.54E-01	2.02E-01
NCRNA00162	0.124821	4.56E-03	8.24E-03
NCRNA00164	0.009188	8.35E-01	8.65E-01
NCRNA00167	-0.01587	7.19E-01	7.66E-01
NCRNA00169	-0.06473	1.42E-01	1.89E-01
NCRNA00171	-0.14359	1.08E-03	2.17E-03
NCRNA00173	-0.15939	2.82E-04	6.15E-04
NCRNA00174	-0.29985	3.68E-12	2.04E-11
NCRNA00175	-0.11444	9.34E-03	1.59E-02
NCRNA00176	-0.02569	5.61E-01	6.22E-01
NCRNA00181	-0.04694	2.88E-01	3.50E-01
NCRNA00182	-0.3631	1.71E-17	1.60E-16
NCRNA00183	-0.02229	6.14E-01	6.70E-01
NCRNA00185	-0.10726	1.49E-02	2.45E-02
NCRNA00188	-0.04187	3.43E-01	4.09E-01
NCRNA00189	0.18785	1.78E-05	4.61E-05
NCRNA00200	0.129311	3.28E-03	6.08E-03
NCRNA00201	-0.37341	1.74E-18	1.77E-17
NCRNA00202	-0.22153	3.81E-07	1.22E-06
NCRNA00203	-0.12285	5.24E-03	9.38E-03
NCRNA00204B	-0.17306	7.89E-05	1.87E-04
NCRNA00207	0.06821	1.22E-01	1.65E-01
NCRNA00219	-0.2543	4.81E-09	1.90E-08
NCRNA00230B	-0.05048	2.53E-01	3.14E-01
NCRNA00235	-0.00287	9.48E-01	9.59E-01
NCS1	0.287235	3.07E-11	1.54E-10
NCSTN	0.000734	9.87E-01	9.90E-01
NDC80	0.85716	6.20E-150	3.26E-147
NDE1	0.161298	2.37E-04	5.24E-04
NDEL1	-0.06265	1.56E-01	2.04E-01
NDFIP1	-0.26932	5.22E-10	2.31E-09
NDFIP2	0.175361	6.31E-05	1.51E-04
NDNL2	-0.04128	3.50E-01	4.16E-01
NDN	-0.32415	4.60E-14	3.07E-13

NDOR1	-0.06637	1.33E-01	1.77E-01
NDP	0.055536	2.08E-01	2.64E-01
NDRG1	0.156279	3.71E-04	7.96E-04
NDRG2	-0.39093	2.98E-20	3.57E-19
NDRG3	0.074135	9.28E-02	1.29E-01
NDRG4	0.189308	1.53E-05	3.99E-05
NDST1	-0.26626	8.31E-10	3.59E-09
NDST2	-0.29921	4.11E-12	2.27E-11
NDST3	0.116426	8.18E-03	1.41E-02
NDST4	0.175791	6.05E-05	1.46E-04
NDUFA10	-0.00342	9.38E-01	9.52E-01
NDUFA11	0.10601	1.61E-02	2.63E-02
NDUFA12	0.380917	3.14E-19	3.43E-18
NDUFA13	-0.00027	9.95E-01	9.96E-01
NDUFA1	0.100796	2.22E-02	3.52E-02
NDUFA2	-0.11915	6.79E-03	1.19E-02
NDUFA3	0.079631	7.10E-02	1.01E-01
NDUFA4L2	0.033184	4.52E-01	5.19E-01
NDUFA4	0.058654	1.84E-01	2.37E-01
NDUFA5	-0.00775	8.61E-01	8.86E-01
NDUFA6	0.121225	5.88E-03	1.04E-02
NDUFA7	0.008491	8.48E-01	8.75E-01
NDUFA8	0.153064	4.91E-04	1.03E-03
NDUFA9	0.454978	1.12E-27	2.44E-26
NDUFAB1	0.190196	1.39E-05	3.64E-05
NDUFAF1	-0.29031	1.85E-11	9.48E-11
NDUFAF2	0.099048	2.46E-02	3.87E-02
NDUFAF3	-0.08976	4.17E-02	6.26E-02
NDUFAF4	0.309117	7.26E-13	4.33E-12
NDUFB10	-0.04344	3.25E-01	3.90E-01
NDUFB11	0.111783	1.11E-02	1.87E-02
NDUFB1	0.088441	4.48E-02	6.68E-02
NDUFB2	0.125276	4.41E-03	7.99E-03
NDUFB3	0.291899	1.42E-11	7.36E-11
NDUFB4	0.288595	2.46E-11	1.24E-10
NDUFB5	0.32895	1.84E-14	1.28E-13
NDUFB6	0.247273	1.30E-08	4.88E-08
NDUFB7	0.048409	2.73E-01	3.35E-01
NDUFB8	0.078328	7.57E-02	1.08E-01
NDUFB9	0.157107	3.45E-04	7.43E-04
NDUFC1	0.066683	1.31E-01	1.75E-01
NDUFC2	0.16098	2.44E-04	5.39E-04
NDUFS1	0.33793	3.19E-15	2.37E-14
NDUFS2	0.053987	2.21E-01	2.79E-01

NDUFS3	0.116217	8.29E-03	1.43E-02
NDUFS4	0.04274	3.33E-01	3.98E-01
NDUFS5	0.082488	6.14E-02	8.90E-02
NDUFS6	0.2921	1.37E-11	7.13E-11
NDUFS7	-0.00128	9.77E-01	9.82E-01
NDUFS8	0.103994	1.82E-02	2.95E-02
NDUFV1	0.027133	5.39E-01	6.01E-01
NDUFV2	0.074699	9.04E-02	1.26E-01
NDUFV3	-0.02131	6.29E-01	6.85E-01
NEAT1	-0.28531	4.20E-11	2.06E-10
NEBL	-0.01465	7.40E-01	7.84E-01
NEB	0.125671	4.29E-03	7.78E-03
NECAB1	-0.17755	5.09E-05	1.24E-04
NECAB2	0.220037	4.58E-07	1.45E-06
NECAB3	-0.22487	2.51E-07	8.21E-07
NECAP1	0.174072	7.16E-05	1.70E-04
NECAP2	0.007825	8.59E-01	8.85E-01
NEDD1	0.505183	1.04E-34	3.51E-33
NEDD4L	-0.41343	1.11E-22	1.61E-21
NEDD4	0.207897	1.95E-06	5.70E-06
NEDD8	0.245194	1.73E-08	6.41E-08
NEDD9	-0.48338	1.64E-31	4.65E-30
NEFH	0.183997	2.65E-05	6.70E-05
NEFL	0.002956	9.47E-01	9.58E-01
NEFM	0.137425	1.77E-03	3.42E-03
NEGR1	-0.40542	8.55E-22	1.16E-20
NEIL1	-0.52491	8.30E-38	3.35E-36
NEIL2	-0.1291	3.34E-03	6.16E-03
NEIL3	0.700263	4.09E-77	4.84E-75
NEK10	-0.16823	1.25E-04	2.88E-04
NEK11	-0.26053	1.95E-09	8.07E-09
NEK1	-0.11634	8.23E-03	1.42E-02
NEK2	0.853232	3.80E-147	1.90E-144
NEK3	0.06402	1.47E-01	1.94E-01
NEK4	0.073203	9.70E-02	1.34E-01
NEK5	-0.20729	2.09E-06	6.09E-06
NEK6	-0.00396	9.29E-01	9.43E-01
NEK7	-0.18878	1.61E-05	4.21E-05
NEK8	-0.43322	5.64E-25	9.91E-24
NEK9	-0.2978	5.24E-12	2.85E-11
NELF	-0.07206	1.02E-01	1.41E-01
NELL1	-0.27744	1.48E-10	6.95E-10
NELL2	-0.12401	4.83E-03	8.69E-03
NENF	-0.18953	1.49E-05	3.90E-05

NEO1	-0.20794	1.94E-06	5.68E-06
NES	-0.01418	7.48E-01	7.90E-01
NET1	0.090005	4.12E-02	6.18E-02
NETO1	0.257229	3.16E-09	1.27E-08
NETO2	0.36616	8.74E-18	8.35E-17
NEU1	0.053804	2.23E-01	2.80E-01
NEU2	0.202503	3.61E-06	1.03E-05
NEU3	0.103446	1.89E-02	3.04E-02
NEU4	0.160647	2.52E-04	5.54E-04
NEURL1B	0.044257	3.16E-01	3.81E-01
NEURL2	0.098478	2.54E-02	3.98E-02
NEURL3	0.08706	4.83E-02	7.14E-02
NEURL4	-0.14049	1.39E-03	2.74E-03
NEURL	0.172699	8.17E-05	1.93E-04
NEUROD1	0.089701	4.19E-02	6.27E-02
NEUROD2	-0.08355	5.81E-02	8.46E-02
NEUROD4	0.157368	3.37E-04	7.27E-04
NEUROD6	0.033452	4.49E-01	5.15E-01
NEUROG1	0.051551	2.43E-01	3.03E-01
NEUROG2	0.272394	3.26E-10	1.48E-09
NEUROG3	0.082518	6.13E-02	8.88E-02
NEXN	-0.08353	5.82E-02	8.47E-02
NF1P1	0.049473	2.62E-01	3.24E-01
NF1	0.025421	5.65E-01	6.25E-01
NF2	0.030095	4.96E-01	5.60E-01
NFAM1	-0.11315	1.02E-02	1.72E-02
NFASC	-0.33057	1.35E-14	9.46E-14
NFAT5	-0.32511	3.84E-14	2.59E-13
NFATC1	-0.39328	1.70E-20	2.07E-19
NFATC2IP	-0.08636	5.01E-02	7.39E-02
NFATC2	-0.11467	9.20E-03	1.57E-02
NFATC3	-0.41858	2.90E-23	4.46E-22
NFATC4	-0.0773	7.97E-02	1.13E-01
NFE2L1	0.038401	3.84E-01	4.51E-01
NFE2L2	-0.14565	9.17E-04	1.85E-03
NFE2L3	0.064562	1.43E-01	1.90E-01
NFE2	-0.2587	2.55E-09	1.04E-08
NFIA	-0.35819	4.91E-17	4.40E-16
NFIB	-0.1947	8.57E-06	2.32E-05
NFIC	-0.22044	4.35E-07	1.38E-06
NFIL3	0.213804	9.73E-07	2.95E-06
NFIX	-0.64748	1.59E-62	1.48E-60
NFKB1	-0.14317	1.12E-03	2.23E-03
NFKB2	0.078707	7.43E-02	1.06E-01

NFKBIA	-0.13614	1.96E-03	3.76E-03
NFKBIB	0.098645	2.52E-02	3.95E-02
NFKBID	-0.14637	8.63E-04	1.75E-03
NFKBIE	0.147636	7.77E-04	1.59E-03
NFKBIL1	-0.05124	2.46E-01	3.06E-01
NFKBIL2	0.451137	3.47E-27	7.27E-26
NFKBIZ	-0.02747	5.34E-01	5.97E-01
NFRKB	-0.00916	8.36E-01	8.65E-01
NFS1	0.080833	6.68E-02	9.61E-02
NFU1	0.245327	1.70E-08	6.30E-08
NFX1	-0.13524	2.10E-03	4.00E-03
NFXL1	0.387814	6.27E-20	7.26E-19
NFYA	0.229835	1.34E-07	4.51E-07
NFYB	0.143756	1.07E-03	2.14E-03
NFYC	-0.20532	2.62E-06	7.55E-06
NGB	0.161637	2.30E-04	5.10E-04
NGDN	0.187695	1.81E-05	4.68E-05
NGEF	0.243904	2.06E-08	7.59E-08
NGFRAP1	-0.04307	3.29E-01	3.95E-01
NGFR	-0.29163	1.48E-11	7.68E-11
NGF	-0.06092	1.67E-01	2.18E-01
NGLY1	0.010249	8.17E-01	8.49E-01
NGRN	0.030174	4.94E-01	5.59E-01
NHEDC1	-0.06205	1.60E-01	2.09E-01
NHEDC2	0.164661	1.75E-04	3.93E-04
NHEG1	0.063283	1.52E-01	2.00E-01
NHEJ1	-0.06011	1.73E-01	2.25E-01
NHLH1	0.078097	7.66E-02	1.09E-01
NHLH2	-0.0732	9.70E-02	1.34E-01
NHLRC1	-0.01381	7.54E-01	7.96E-01
NHLRC2	0.186882	1.97E-05	5.07E-05
NHLRC3	-0.10296	1.94E-02	3.12E-02
NHLRC4	-0.35742	5.78E-17	5.15E-16
NHP2L1	-0.01388	7.53E-01	7.95E-01
NHP2	0.076141	8.43E-02	1.18E-01
NHSL1	-0.13632	1.93E-03	3.71E-03
NHSL2	-0.30898	7.45E-13	4.43E-12
NHS	-0.10073	2.22E-02	3.53E-02
NICN1	-0.57911	1.88E-47	1.20E-45
NID1	0.109049	1.33E-02	2.21E-02
NID2	0.22571	2.26E-07	7.42E-07
NIF3L1	0.349775	2.88E-16	2.38E-15
NINJ1	-0.2484	1.11E-08	4.20E-08
NINJ2	-0.38383	1.60E-19	1.79E-18

NINL	-0.15541	4.00E-04	8.53E-04
NIN	0.095112	3.09E-02	4.76E-02
NIP7	0.331392	1.15E-14	8.13E-14
NIPA1	0.008679	8.44E-01	8.73E-01
NIPA2	0.223059	3.15E-07	1.02E-06
NIPAL1	0.204895	2.75E-06	7.91E-06
NIPAL2	-0.17865	4.56E-05	1.12E-04
NIPAL3	-0.47492	2.48E-30	6.53E-29
NIPAL4	0.25261	6.13E-09	2.40E-08
NIPBL	-0.01984	6.53E-01	7.07E-01
NIPSNAP1	0.266495	8.02E-10	3.47E-09
NIPSNAP3A	-0.02786	5.28E-01	5.92E-01
NIPSNAP3B	-0.24619	1.51E-08	5.63E-08
NISCH	-0.48261	2.10E-31	5.89E-30
NIT1	-0.05156	2.43E-01	3.03E-01
NIT2	0.245408	1.68E-08	6.24E-08
NKAIN1	0.402124	1.95E-21	2.57E-20
NKAIN2	0.156459	3.65E-04	7.84E-04
NKAIN3	0.0157	7.22E-01	7.68E-01
NKAIN4	0.205912	2.45E-06	7.07E-06
NKAPL	-0.19674	6.86E-06	1.88E-05
NKAP	0.195206	8.11E-06	2.20E-05
NKD1	-0.24759	1.24E-08	4.68E-08
NKD2	-0.23985	3.58E-08	1.28E-07
NKG7	0.165069	1.68E-04	3.79E-04
NKIRAS1	-0.33585	4.81E-15	3.53E-14
NKIRAS2	0.400874	2.66E-21	3.46E-20
NKPD1	0.164068	1.84E-04	4.14E-04
NKRF	0.341053	1.71E-15	1.30E-14
NKTR	-0.27652	1.72E-10	7.99E-10
NKX1-2	0.236833	5.35E-08	1.88E-07
NKX2-1	-0.42214	1.13E-23	1.80E-22
NKX2-2	0.075883	8.54E-02	1.20E-01
NKX2-3	0.168801	1.19E-04	2.73E-04
NKX2-4	0.116501	8.13E-03	1.40E-02
NKX2-5	0.212928	1.08E-06	3.26E-06
NKX2-6	0.129387	3.27E-03	6.05E-03
NKX2-8	-0.07297	9.81E-02	1.35E-01
NKX3-1	0.057109	1.96E-01	2.50E-01
NKX3-2	0.178443	4.65E-05	1.14E-04
NKX6-1	0.17818	4.78E-05	1.17E-04
NKX6-2	-0.12224	5.47E-03	9.76E-03
NKX6-3	-0.01811	6.82E-01	7.32E-01
NLE1	0.311018	5.17E-13	3.13E-12

NLGN1	0.053467	2.26E-01	2.84E-01
NLGN2	0.075827	8.56E-02	1.20E-01
NLGN3	-0.11119	1.16E-02	1.94E-02
NLGN4X	0.108778	1.35E-02	2.24E-02
NLGN4Y	0.068292	1.22E-01	1.64E-01
NLK	0.035009	4.28E-01	4.94E-01
NLN	0.385622	1.05E-19	1.19E-18
NLRC3	-0.20744	2.05E-06	5.99E-06
NLRC4	-0.09254	3.58E-02	5.44E-02
NLRC5	0.141059	1.33E-03	2.62E-03
NLRP10	0.121872	5.62E-03	9.99E-03
NLRP11	0.173858	7.30E-05	1.74E-04
NLRP12	-0.09384	3.32E-02	5.09E-02
NLRP13	0.022297	6.14E-01	6.70E-01
NLRP14	0.000381	9.93E-01	9.95E-01
NLRP1	-0.37902	4.87E-19	5.19E-18
NLRP2	-0.04357	3.24E-01	3.89E-01
NLRP3	-0.16342	1.96E-04	4.38E-04
NLRP4	0.02103	6.34E-01	6.89E-01
NLRP5	0.031577	4.75E-01	5.40E-01
NLRP6	-0.03257	4.61E-01	5.27E-01
NLRP7	0.053862	2.22E-01	2.80E-01
NLRP8	0.055256	2.11E-01	2.67E-01
NLRP9	-0.19184	1.17E-05	3.09E-05
NLRX1	-0.25976	2.18E-09	8.97E-09
NMBR	-0.21105	1.35E-06	4.03E-06
NMB	0.039997	3.65E-01	4.31E-01
NMD3	0.53485	1.90E-39	8.56E-38
NME1-NME2	0.213586	9.99E-07	3.02E-06
NME1	0.547335	1.39E-41	6.86E-40
NME2P1	0.223168	3.11E-07	1.00E-06
NME2	0.30803	8.82E-13	5.21E-12
NME3	-0.29422	9.63E-12	5.08E-11
NME4	0.062712	1.55E-01	2.04E-01
NME5	-0.31579	2.17E-13	1.36E-12
NME6	0.211916	1.22E-06	3.65E-06
NME7	0.123741	4.92E-03	8.85E-03
NMI	0.298181	4.91E-12	2.68E-11
NMNAT1	-0.07528	8.79E-02	1.23E-01
NMNAT2	0.020379	6.45E-01	6.99E-01
NMNAT3	-0.2117	1.25E-06	3.74E-06
NMRAL1	-0.0799	7.00E-02	1.00E-01
NMT1	0.287519	2.93E-11	1.47E-10
NMT2	-0.07539	8.74E-02	1.22E-01

NMUR1	-0.28661	3.41E-11	1.69E-10
NMUR2	-0.15766	3.28E-04	7.10E-04
NMU	0.255481	4.06E-09	1.62E-08
NNAT	-0.15381	4.60E-04	9.71E-04
NNMT	-0.06141	1.64E-01	2.14E-01
NNT	0.041498	3.47E-01	4.13E-01
NOB1	0.057554	1.92E-01	2.46E-01
NOBOX	0.046131	2.96E-01	3.59E-01
NOC2L	0.218561	5.48E-07	1.72E-06
NOC3L	0.32281	5.92E-14	3.92E-13
NOC4L	0.281835	7.38E-11	3.55E-10
NOD1	-0.53623	1.12E-39	5.08E-38
NOD2	-0.03893	3.78E-01	4.44E-01
NODAL	-0.03692	4.03E-01	4.69E-01
NOG	-0.20289	3.46E-06	9.84E-06
NOL10	0.538378	4.84E-40	2.26E-38
NOL11	0.544955	3.60E-41	1.74E-39
NOL12	-0.06006	1.74E-01	2.25E-01
NOL3	-0.18324	2.87E-05	7.20E-05
NOL4	0.080121	6.93E-02	9.93E-02
NOL6	0.106833	1.53E-02	2.51E-02
NOL7	0.302859	2.19E-12	1.25E-11
NOL8	0.078527	7.50E-02	1.07E-01
NOL9	0.083643	5.78E-02	8.42E-02
NOLC1	0.341208	1.66E-15	1.26E-14
NOM1	0.250232	8.58E-09	3.30E-08
NOMO1	0.069103	1.17E-01	1.59E-01
NOMO2	0.066969	1.29E-01	1.73E-01
NOMO3	-0.04308	3.29E-01	3.95E-01
NONO	0.263869	1.19E-09	5.04E-09
NOP10	0.276372	1.76E-10	8.17E-10
NOP14	0.180135	3.93E-05	9.69E-05
NOP16	0.286937	3.23E-11	1.61E-10
NOP2	0.389643	4.06E-20	4.79E-19
NOP56	0.408853	3.59E-22	5.02E-21
NOP58	0.386881	7.81E-20	8.96E-19
NOS1AP	-0.20956	1.61E-06	4.75E-06
NOS1	-0.08525	5.32E-02	7.80E-02
NOS2	0.042436	3.36E-01	4.02E-01
NOS3	-0.09524	3.07E-02	4.73E-02
NOSIP	0.135968	1.98E-03	3.81E-03
NOSTRIN	-0.43735	1.79E-25	3.28E-24
NOTCH1	-0.28915	2.24E-11	1.14E-10
NOTCH2NL	-0.01725	6.96E-01	7.45E-01

NOTCH2	-0.05056	2.52E-01	3.13E-01
NOTCH3	0.095046	3.10E-02	4.78E-02
NOTCH4	-0.26716	7.25E-10	3.15E-09
NOTO	-0.22585	2.22E-07	7.30E-07
NOTUM	-0.20476	2.80E-06	8.03E-06
NOVA1	0.06623	1.33E-01	1.78E-01
NOVA2	-0.19119	1.25E-05	3.30E-05
NOV	0.044615	3.12E-01	3.77E-01
NOX1	-0.03287	4.57E-01	5.23E-01
NOX3	0.029481	5.04E-01	5.69E-01
NOX4	0.121983	5.57E-03	9.93E-03
NOX5	0.011737	7.90E-01	8.27E-01
NOXA1	-0.28422	5.03E-11	2.45E-10
NOXO1	-0.03765	3.94E-01	4.61E-01
NPAS1	-0.00399	9.28E-01	9.43E-01
NPAS2	-0.03537	4.23E-01	4.89E-01
NPAS3	-0.22364	2.93E-07	9.50E-07
NPAS4	-0.0189	6.69E-01	7.20E-01
NPAT	0.035177	4.26E-01	4.92E-01
NPBWR1	0.187059	1.93E-05	4.98E-05
NPBWR2	0.050965	2.48E-01	3.09E-01
NPB	0.106619	1.55E-02	2.54E-02
NPC1L1	0.077461	7.90E-02	1.12E-01
NPC1	0.125212	4.43E-03	8.02E-03
NPC2	-0.46774	2.35E-29	5.79E-28
NPDC1	-0.24507	1.76E-08	6.52E-08
NPEPL1	-0.31952	1.09E-13	7.06E-13
NPEPPS	0.374705	1.30E-18	1.34E-17
NPFFR1	-0.25056	8.19E-09	3.15E-08
NPFFR2	0.217705	6.09E-07	1.90E-06
NPFF	-0.26139	1.72E-09	7.15E-09
NPHP1	-0.22088	4.12E-07	1.31E-06
NPHP3	-0.24904	1.01E-08	3.86E-08
NPHP4	-0.2579	2.86E-09	1.16E-08
NPHS1	-0.00998	8.21E-01	8.53E-01
NPHS2	0.022181	6.16E-01	6.72E-01
NPIPL3	-0.24083	3.13E-08	1.13E-07
NPIP	-0.33344	7.72E-15	5.53E-14
NPLOC4	0.18068	3.72E-05	9.20E-05
NPL	-0.01583	7.20E-01	7.66E-01
NPM1	0.218438	5.57E-07	1.74E-06
NPM2	-0.02174	6.23E-01	6.79E-01
NPM3	0.356342	7.28E-17	6.39E-16
NPNT	-0.35943	3.77E-17	3.42E-16

NPPA	-0.12364	4.96E-03	8.91E-03
NPPB	0.120137	6.34E-03	1.12E-02
NPPC	-0.03352	4.48E-01	5.14E-01
NPR1	-0.41694	4.46E-23	6.73E-22
NPR2	-0.2644	1.10E-09	4.67E-09
NPR3	-0.10822	1.40E-02	2.32E-02
NPRL2	-0.18782	1.78E-05	4.62E-05
NPRL3	-0.1269	3.92E-03	7.16E-03
NPSR1	0.105647	1.65E-02	2.68E-02
NPTN	0.091939	3.70E-02	5.61E-02
NPTX1	-0.11278	1.04E-02	1.76E-02
NPTX2	0.18797	1.76E-05	4.56E-05
NPTXR	-0.10214	2.04E-02	3.26E-02
NPVF	0.014284	7.46E-01	7.89E-01
NPW	0.078393	7.55E-02	1.07E-01
NPY1R	-0.10441	1.78E-02	2.88E-02
NPY2R	-0.1281	3.59E-03	6.60E-03
NPY5R	-0.06344	1.51E-01	1.98E-01
NPY6R	-0.26109	1.80E-09	7.46E-09
NPY	0.068775	1.19E-01	1.61E-01
NQO1	0.026432	5.50E-01	6.12E-01
NQO2	0.002512	9.55E-01	9.64E-01
NR0B1	0.169648	1.09E-04	2.54E-04
NR0B2	-0.25945	2.28E-09	9.36E-09
NR1D1	-0.01204	7.85E-01	8.22E-01
NR1D2	-0.18477	2.45E-05	6.22E-05
NR1H2	-0.28091	8.56E-11	4.09E-10
NR1H3	-0.04616	2.96E-01	3.59E-01
NR1H4	-0.00419	9.24E-01	9.40E-01
NR1I2	-0.06093	1.67E-01	2.18E-01
NR1I3	0.137753	1.73E-03	3.34E-03
NR2C1	0.001472	9.73E-01	9.80E-01
NR2C2AP	0.170661	9.94E-05	2.32E-04
NR2C2	-0.23568	6.23E-08	2.17E-07
NR2E1	0.137704	1.73E-03	3.35E-03
NR2E3	-0.244	2.04E-08	7.49E-08
NR2F1	-0.34525	7.31E-16	5.81E-15
NR2F2	-0.12407	4.81E-03	8.66E-03
NR2F6	0.034799	4.31E-01	4.97E-01
NR3C1	-0.20846	1.83E-06	5.36E-06
NR3C2	-0.53733	7.28E-40	3.34E-38
NR4A1	-0.26492	1.02E-09	4.35E-09
NR4A2	-0.15602	3.80E-04	8.12E-04
NR4A3	-0.17747	5.13E-05	1.25E-04

NR5A1	0.238902	4.06E-08	1.44E-07
NR5A2	-0.03683	4.04E-01	4.71E-01
NR6A1	0.134157	2.28E-03	4.32E-03
NRADDP	-0.17725	5.24E-05	1.27E-04
NRAP	-0.23202	1.01E-07	3.44E-07
NRARP	0.233004	8.86E-08	3.04E-07
NRAS	0.523665	1.32E-37	5.23E-36
NRBF2	0.243824	2.09E-08	7.67E-08
NRBP1	0.461482	1.59E-28	3.67E-27
NRBP2	-0.31591	2.13E-13	1.33E-12
NRCAM	0.05718	1.95E-01	2.50E-01
NRD1	0.281412	7.89E-11	3.79E-10
NRF1	-0.0439	3.20E-01	3.85E-01
NRG1	-0.06892	1.18E-01	1.60E-01
NRG2	-0.21471	8.74E-07	2.67E-06
NRG3	-0.17286	8.04E-05	1.90E-04
NRG4	0.000162	9.97E-01	9.98E-01
NRGN	-0.30567	1.34E-12	7.75E-12
NRIP1	0.293814	1.03E-11	5.43E-11
NRIP2	-0.44079	6.81E-26	1.29E-24
NRIP3	0.175832	6.02E-05	1.45E-04
NRK	0.042394	3.37E-01	4.02E-01
NRL	-0.19848	5.67E-06	1.57E-05
NRM	0.516251	2.02E-36	7.53E-35
NRN1L	-0.29773	5.30E-12	2.88E-11
NRN1	-0.05476	2.15E-01	2.71E-01
NRP1	-0.09054	4.00E-02	6.02E-02
NRP2	0.059021	1.81E-01	2.34E-01
NRSN1	0.059571	1.77E-01	2.29E-01
NRSN2	-0.02684	5.43E-01	6.06E-01
NRTN	0.085471	5.26E-02	7.72E-02
NRXN1	-0.08405	5.66E-02	8.26E-02
NRXN2	-0.20462	2.84E-06	8.15E-06
NRXN3	-0.30226	2.43E-12	1.38E-11
NSA2	-0.08105	6.61E-02	9.51E-02
NSD1	-0.0735	9.57E-02	1.32E-01
NSDHL	0.271224	3.91E-10	1.75E-09
NSFL1C	0.018853	6.70E-01	7.21E-01
NSF	0.20306	3.39E-06	9.67E-06
NSL1	0.144359	1.02E-03	2.04E-03
NSMAF	0.09988	2.34E-02	3.70E-02
NSMCE1	-0.1346	2.21E-03	4.19E-03
NSMCE2	0.196099	7.36E-06	2.00E-05
NSMCE4A	-0.08739	4.75E-02	7.03E-02

NSUN2	0.351323	2.09E-16	1.75E-15
NSUN3	0.172555	8.29E-05	1.95E-04
NSUN4	-0.16078	2.49E-04	5.47E-04
NSUN5P1	-0.13889	1.58E-03	3.07E-03
NSUN5P2	-0.1272	3.84E-03	7.02E-03
NSUN5	0.236127	5.88E-08	2.05E-07
NSUN6	-0.07261	9.98E-02	1.37E-01
NSUN7	-0.15185	5.45E-04	1.14E-03
NT5C1A	-0.37232	2.23E-18	2.25E-17
NT5C1B	-0.01473	7.39E-01	7.82E-01
NT5C2	-0.00089	9.84E-01	9.88E-01
NT5C3L	0.244728	1.84E-08	6.82E-08
NT5C3	0.420981	1.54E-23	2.42E-22
NT5C	0.005512	9.01E-01	9.20E-01
NT5DC1	-0.13315	2.46E-03	4.64E-03
NT5DC2	0.061235	1.65E-01	2.15E-01
NT5DC3	0.185532	2.27E-05	5.78E-05
NT5E	-0.06081	1.68E-01	2.19E-01
NT5M	0.075024	8.90E-02	1.24E-01
NTAN1	0.229763	1.35E-07	4.55E-07
NTF3	-0.1657	1.59E-04	3.59E-04
NTF4	-0.28039	9.30E-11	4.43E-10
NTHL1	-0.15911	2.89E-04	6.29E-04
NTM	-0.03505	4.27E-01	4.94E-01
NTN1	-0.09973	2.36E-02	3.73E-02
NTN3	0.047424	2.83E-01	3.45E-01
NTN4	-0.25463	4.60E-09	1.82E-08
NTN5	-0.20576	2.49E-06	7.19E-06
NTNG1	-0.24154	2.85E-08	1.03E-07
NTNG2	0.089486	4.24E-02	6.34E-02
NTRK1	-0.09229	3.63E-02	5.50E-02
NTRK2	-0.27285	3.04E-10	1.39E-09
NTRK3	-0.46016	2.38E-28	5.42E-27
NTSR1	0.210722	1.40E-06	4.17E-06
NTSR2	0.10307	1.93E-02	3.10E-02
NTS	0.145796	9.06E-04	1.83E-03
NUAK1	0.045248	3.05E-01	3.69E-01
NUAK2	-0.2282	1.65E-07	5.49E-07
NUB1	0.07045	1.10E-01	1.50E-01
NUBP1	-0.36401	1.40E-17	1.31E-16
NUBP2	-0.06684	1.30E-01	1.74E-01
NUBPL	0.051674	2.42E-01	3.02E-01
NUCB1	-0.33998	2.12E-15	1.60E-14
NUCB2	-0.13767	1.74E-03	3.36E-03

NUCKS1	0.037415	3.97E-01	4.63E-01
NUDCD1	0.456954	6.21E-28	1.38E-26
NUDCD2	0.144956	9.70E-04	1.95E-03
NUDCD3	-0.00097	9.83E-01	9.87E-01
NUDC	0.111712	1.12E-02	1.88E-02
NUDT10	-0.00146	9.74E-01	9.80E-01
NUDT11	0.176497	5.64E-05	1.36E-04
NUDT12	-0.22498	2.48E-07	8.10E-07
NUDT13	-0.38055	3.42E-19	3.71E-18
NUDT14	-0.13422	2.27E-03	4.30E-03
NUDT15	0.315102	2.47E-13	1.54E-12
NUDT16L1	-0.26334	1.29E-09	5.43E-09
NUDT16P1	0.036201	4.12E-01	4.79E-01
NUDT16	-0.36163	2.35E-17	2.17E-16
NUDT17	-0.18069	3.72E-05	9.20E-05
NUDT18	-0.19812	5.89E-06	1.63E-05
NUDT19	0.242857	2.38E-08	8.69E-08
NUDT1	0.572935	2.92E-46	1.79E-44
NUDT21	0.225177	2.42E-07	7.91E-07
NUDT22	-0.05046	2.53E-01	3.14E-01
NUDT2	0.24864	1.07E-08	4.07E-08
NUDT3	0.313633	3.22E-13	1.99E-12
NUDT4	0.091807	3.73E-02	5.64E-02
NUDT5	0.245983	1.55E-08	5.78E-08
NUDT6	0.028226	5.23E-01	5.87E-01
NUDT7	-0.35472	1.02E-16	8.86E-16
NUDT8	0.002162	9.61E-01	9.69E-01
NUDT9P1	0.002413	9.56E-01	9.65E-01
NUDT9	-0.09251	3.58E-02	5.45E-02
NUF2	0.833962	1.47E-134	4.60E-132
NUFIP1	0.273404	2.79E-10	1.28E-09
NUFIP2	0.066768	1.30E-01	1.74E-01
NUMA1	-0.26811	6.28E-10	2.75E-09
NUMBL	0.18375	2.72E-05	6.86E-05
NUMB	-0.21665	6.92E-07	2.14E-06
NUP107	0.524554	9.49E-38	3.81E-36
NUP133	0.005384	9.03E-01	9.22E-01
NUP153	0.316435	1.93E-13	1.22E-12
NUP155	0.554936	6.24E-43	3.33E-41
NUP160	0.24435	1.94E-08	7.17E-08
NUP188	0.187921	1.77E-05	4.58E-05
NUP205	0.610297	7.15E-54	5.29E-52
NUP210L	-0.29341	1.10E-11	5.79E-11
NUP210	0.375044	1.21E-18	1.24E-17

NUP214	-0.04226	3.39E-01	4.04E-01
NUP35	0.283662	5.50E-11	2.67E-10
NUP37	0.610626	6.06E-54	4.51E-52
NUP43	0.285682	3.96E-11	1.95E-10
NUP50	0.255134	4.27E-09	1.70E-08
NUP54	0.417119	4.26E-23	6.44E-22
NUP62CL	0.297709	5.32E-12	2.89E-11
NUP62	0.435797	2.76E-25	4.99E-24
NUP85	0.500251	5.77E-34	1.87E-32
NUP88	0.092824	3.52E-02	5.36E-02
NUP93	0.349055	3.35E-16	2.75E-15
NUP98	0.120027	6.39E-03	1.12E-02
NUPL1	0.031969	4.69E-01	5.35E-01
NUPL2	0.280762	8.76E-11	4.18E-10
NUPR1	-0.18508	2.37E-05	6.04E-05
NUS1	0.356532	6.99E-17	6.15E-16
NUSAP1	0.891713	9.52E-179	2.72E-175
NUTF2	0.25979	2.17E-09	8.93E-09
NVL	-0.0732	9.71E-02	1.34E-01
NWD1	-0.37997	3.91E-19	4.20E-18
NXF1	-0.30628	1.20E-12	6.99E-12
NXF2B	-0.13386	2.33E-03	4.42E-03
NXF2	0.024725	5.76E-01	6.35E-01
NXF3	-0.2366	5.52E-08	1.93E-07
NXF4	0.026218	5.53E-01	6.14E-01
NXF5	-0.05127	2.45E-01	3.06E-01
NXNL1	-0.1125	1.06E-02	1.79E-02
NXNL2	-0.0067	8.79E-01	9.02E-01
NXN	-0.1683	1.24E-04	2.86E-04
NXPH1	0.024023	5.86E-01	6.45E-01
NXPH2	0.143083	1.13E-03	2.25E-03
NXPH3	-0.37793	6.25E-19	6.58E-18
NXPH4	0.284126	5.10E-11	2.49E-10
NXT1	0.229035	1.48E-07	4.97E-07
NXT2	0.029516	5.04E-01	5.68E-01
NYNRIN	-0.12266	5.31E-03	9.49E-03
NYX	0.208676	1.78E-06	5.24E-06
OAF	0.02697	5.41E-01	6.04E-01
OAS1	0.343731	9.95E-16	7.79E-15
OAS2	0.146391	8.62E-04	1.75E-03
OAS3	0.382921	1.98E-19	2.20E-18
OASL	0.280026	9.85E-11	4.68E-10
OAT	-0.09364	3.36E-02	5.14E-02
OAZ1	0.065433	1.38E-01	1.84E-01

OAZ2	-0.31029	5.89E-13	3.54E-12
OAZ3	-0.10456	1.76E-02	2.85E-02
OBFC1	-0.32662	2.88E-14	1.96E-13
OBFC2A	0.200395	4.58E-06	1.28E-05
OBFC2B	0.520235	4.70E-37	1.80E-35
OBP2A	0.036642	4.07E-01	4.73E-01
OBP2B	0.03352	4.48E-01	5.14E-01
OBSCN	-0.22833	1.62E-07	5.40E-07
OBSL1	-0.20133	4.12E-06	1.16E-05
OC90	-0.01411	7.49E-01	7.91E-01
OCA2	-0.37927	4.59E-19	4.90E-18
OCEL1	-0.26772	6.66E-10	2.91E-09
OCIAD1	0.055359	2.10E-01	2.66E-01
OCIAD2	0.102609	1.99E-02	3.18E-02
OCLM	0.040867	3.55E-01	4.21E-01
OCLN	-0.32478	4.08E-14	2.74E-13
OCM2	-0.03277	4.58E-01	5.24E-01
OCM	-0.1053	1.68E-02	2.74E-02
OCRL	0.222195	3.51E-07	1.13E-06
ODAM	-0.25126	7.42E-09	2.87E-08
ODC1	-0.0063	8.87E-01	9.08E-01
ODF1	-0.10879	1.35E-02	2.24E-02
ODF2L	0.050659	2.51E-01	3.12E-01
ODF2	0.101636	2.11E-02	3.36E-02
ODF3B	-0.28113	8.25E-11	3.95E-10
ODF3L1	-0.24758	1.24E-08	4.68E-08
ODF3L2	0.027992	5.26E-01	5.90E-01
ODF3	-0.04622	2.95E-01	3.58E-01
ODF4	0.014866	7.36E-01	7.80E-01
ODZ1	-0.20224	3.72E-06	1.05E-05
ODZ2	-0.17564	6.14E-05	1.48E-04
ODZ3	0.230836	1.17E-07	3.98E-07
ODZ4	-0.11316	1.02E-02	1.72E-02
OFD1	-0.28776	2.82E-11	1.42E-10
OGDHL	0.189582	1.48E-05	3.88E-05
OGDH	-0.00741	8.67E-01	8.91E-01
OGFOD1	0.157996	3.19E-04	6.91E-04
OGFOD2	-0.05887	1.82E-01	2.35E-01
OGFRL1	0.127798	3.67E-03	6.74E-03
OGFR	-0.07517	8.83E-02	1.23E-01
OGG1	0.105161	1.70E-02	2.76E-02
OGN	-0.43082	1.09E-24	1.88E-23
OGT	-0.21621	7.30E-07	2.25E-06
OIP5	0.798737	2.87E-115	6.11E-113

OIT3	-0.28239	6.75E-11	3.26E-10
OLA1	0.536483	1.01E-39	4.62E-38
OLAH	0.111608	1.13E-02	1.89E-02
OLFM1	-0.36372	1.49E-17	1.40E-16
OLFM2	-0.12699	3.89E-03	7.12E-03
OLFM3	0.12905	3.35E-03	6.19E-03
OLFM4	0.056178	2.03E-01	2.58E-01
OLFML1	-0.28372	5.44E-11	2.65E-10
OLFML2A	-0.02236	6.13E-01	6.70E-01
OLFML2B	0.192679	1.07E-05	2.84E-05
OLFML3	-0.19605	7.40E-06	2.01E-05
OLIG1	-0.2631	1.33E-09	5.62E-09
OLIG2	0.154319	4.40E-04	9.32E-04
OLIG3	0.079823	7.03E-02	1.01E-01
OLR1	-0.13335	2.43E-03	4.58E-03
OMA1	-0.30211	2.50E-12	1.41E-11
OMD	-0.19708	6.61E-06	1.81E-05
OMG	-0.01572	7.22E-01	7.68E-01
OMP	-0.0428	3.32E-01	3.98E-01
ONECUT1	0.064449	1.44E-01	1.91E-01
ONECUT2	0.051622	2.42E-01	3.02E-01
ONECUT3	-0.04841	2.73E-01	3.35E-01
OOEP	0.02023	6.47E-01	7.01E-01
OPA1	0.501095	4.31E-34	1.42E-32
OPA3	0.040706	3.57E-01	4.22E-01
OPALIN	0.019753	6.55E-01	7.08E-01
OPCML	-0.24904	1.01E-08	3.86E-08
OPHN1	-0.45206	2.65E-27	5.60E-26
OPLAH	-0.15422	4.44E-04	9.39E-04
OPN1LW	0.010786	8.07E-01	8.40E-01
OPN1MW	0.043625	3.23E-01	3.88E-01
OPN1SW	0.082963	5.99E-02	8.70E-02
OPN3	0.188108	1.73E-05	4.50E-05
OPN4	0.038632	3.82E-01	4.48E-01
OPN5	-0.08806	4.58E-02	6.81E-02
OPRD1	0.146161	8.79E-04	1.78E-03
OPRK1	-0.08939	4.26E-02	6.37E-02
OPRL1	-0.04366	3.23E-01	3.88E-01
OPRM1	0.041494	3.47E-01	4.13E-01
OPTC	0.050816	2.50E-01	3.10E-01
OPTN	0.18099	3.60E-05	8.93E-05
OR10A2	-0.01335	7.62E-01	8.02E-01
OR10A3	-0.07757	7.86E-02	1.11E-01
OR10A4	-0.03578	4.18E-01	4.84E-01

OR10A5	0.019739	6.55E-01	7.08E-01
OR10A6	-0.05855	1.85E-01	2.38E-01
OR10AD1	-0.20044	4.56E-06	1.28E-05
OR10G2	-0.03959	3.70E-01	4.36E-01
OR10G3	0.018657	6.73E-01	7.24E-01
OR10G4	-0.0094	8.32E-01	8.61E-01
OR10G7	-0.0243	5.82E-01	6.41E-01
OR10G8	0.000349	9.94E-01	9.95E-01
OR10H1	0.069536	1.15E-01	1.56E-01
OR10H2	0.039187	3.75E-01	4.41E-01
OR10H5	0.067148	1.28E-01	1.72E-01
OR10J1	0.003862	9.30E-01	9.45E-01
OR10J3	0.058372	1.86E-01	2.39E-01
OR10J5	0.002246	9.59E-01	9.68E-01
OR10P1	-0.0393	3.73E-01	4.40E-01
OR10Q1	-0.01036	8.15E-01	8.47E-01
OR10S1	0.08789	4.62E-02	6.86E-02
OR10V1	-0.19638	7.14E-06	1.95E-05
OR10W1	0.053572	2.25E-01	2.83E-01
OR11A1	0.005527	9.00E-01	9.20E-01
OR11G2	-0.0426	3.35E-01	4.00E-01
OR11H12	0.00853	8.47E-01	8.75E-01
OR11H4	0.021438	6.27E-01	6.83E-01
OR11H6	0.032481	4.62E-01	5.28E-01
OR12D2	0.06334	1.51E-01	1.99E-01
OR13A1	-0.12823	3.56E-03	6.54E-03
OR13C2	-0.10032	2.28E-02	3.61E-02
OR13C3	0.049997	2.57E-01	3.18E-01
OR13C5	-0.05583	2.06E-01	2.61E-01
OR13C9	-0.03647	4.09E-01	4.75E-01
OR13D1	0.040929	3.54E-01	4.20E-01
OR13F1	-0.03354	4.48E-01	5.14E-01
OR13G1	0.036959	4.03E-01	4.69E-01
OR13H1	0.008867	8.41E-01	8.70E-01
OR13J1	-0.14023	1.42E-03	2.79E-03
OR14A16	0.066115	1.34E-01	1.79E-01
OR14C36	0.024907	5.73E-01	6.32E-01
OR14I1	-0.02436	5.81E-01	6.40E-01
OR1A2	0.062687	1.55E-01	2.04E-01
OR1B1	-0.02909	5.10E-01	5.74E-01
OR1C1	0.053466	2.26E-01	2.84E-01
OR1D2	-0.00755	8.64E-01	8.89E-01
OR1D4	0.068438	1.21E-01	1.63E-01
OR1E1	-0.00033	9.94E-01	9.95E-01

OR1E2	-0.05235	2.36E-01	2.95E-01
OR1F1	0.344422	8.65E-16	6.83E-15
OR1F2P	0.180057	3.96E-05	9.76E-05
OR1G1	0.00645	8.84E-01	9.06E-01
OR1J1	0.052262	2.36E-01	2.95E-01
OR1J2	0.078642	7.46E-02	1.06E-01
OR1J4	0.067415	1.27E-01	1.70E-01
OR1K1	-0.02252	6.10E-01	6.67E-01
OR1L1	0.011729	7.91E-01	8.27E-01
OR1L3	0.011931	7.87E-01	8.23E-01
OR1L4	-0.01911	6.65E-01	7.17E-01
OR1L6	0.03231	4.64E-01	5.30E-01
OR1L8	-0.23076	1.19E-07	4.02E-07
OR1M1	0.003308	9.40E-01	9.53E-01
OR1N1	-0.08019	6.90E-02	9.90E-02
OR1N2	0.010799	8.07E-01	8.40E-01
OR1Q1	-0.02705	5.40E-01	6.03E-01
OR2A12	0.026372	5.50E-01	6.12E-01
OR2A14	0.055606	2.08E-01	2.63E-01
OR2A1	-0.21205	1.20E-06	3.60E-06
OR2A25	-0.03255	4.61E-01	5.27E-01
OR2A2	-0.01432	7.46E-01	7.89E-01
OR2A4	-0.12698	3.90E-03	7.12E-03
OR2A5	-0.06154	1.63E-01	2.13E-01
OR2A7	-0.07165	1.04E-01	1.43E-01
OR2A9P	-0.10482	1.73E-02	2.81E-02
OR2AE1	0.001493	9.73E-01	9.79E-01
OR2AG1	0.005904	8.94E-01	9.14E-01
OR2AG2	0.053256	2.28E-01	2.86E-01
OR2AK2	-0.01422	7.48E-01	7.90E-01
OR2AT4	0.02921	5.08E-01	5.72E-01
OR2B11	-0.04421	3.17E-01	3.81E-01
OR2B2	0.033191	4.52E-01	5.18E-01
OR2B3	-0.01299	7.69E-01	8.08E-01
OR2B6	0.290938	1.67E-11	8.57E-11
OR2C1	-0.18338	2.83E-05	7.10E-05
OR2C3	0.097436	2.70E-02	4.21E-02
OR2D2	0.002922	9.47E-01	9.59E-01
OR2D3	0.033287	4.51E-01	5.17E-01
OR2F1	-0.03199	4.69E-01	5.35E-01
OR2F2	0.00784	8.59E-01	8.85E-01
OR2G2	-0.08129	6.53E-02	9.41E-02
OR2G6	-0.03088	4.84E-01	5.50E-01
OR2H1	0.042514	3.36E-01	4.01E-01

OR2H2	0.149161	6.84E-04	1.41E-03
OR2J2	-0.04475	3.11E-01	3.75E-01
OR2J3	0.024509	5.79E-01	6.38E-01
OR2K2	-0.15918	2.87E-04	6.26E-04
OR2L13	-0.1062	1.59E-02	2.60E-02
OR2L1P	0.014816	7.37E-01	7.81E-01
OR2L2	0.057104	1.96E-01	2.50E-01
OR2L3	-0.01438	7.45E-01	7.88E-01
OR2M3	0.088301	4.52E-02	6.73E-02
OR2M4	0.062695	1.55E-01	2.04E-01
OR2S2	0.042576	3.35E-01	4.00E-01
OR2T10	-0.13141	2.81E-03	5.25E-03
OR2T2	-0.05597	2.05E-01	2.60E-01
OR2T33	0.075692	8.62E-02	1.21E-01
OR2T34	-0.04154	3.47E-01	4.13E-01
OR2T3	-0.10162	2.11E-02	3.36E-02
OR2T4	-0.00815	8.54E-01	8.81E-01
OR2T5	-0.02932	5.07E-01	5.71E-01
OR2T6	0.032349	4.64E-01	5.29E-01
OR2T8	0.061314	1.65E-01	2.15E-01
OR2V2	0.024165	5.84E-01	6.43E-01
OR2W1	0.004454	9.20E-01	9.36E-01
OR2W3	0.00592	8.93E-01	9.14E-01
OR2W5	0.038154	3.88E-01	4.54E-01
OR2Z1	0.082006	6.29E-02	9.10E-02
OR3A1	-0.00583	8.95E-01	9.15E-01
OR3A2	0.010793	8.07E-01	8.40E-01
OR3A3	-0.03075	4.86E-01	5.51E-01
OR3A4	-0.01126	7.99E-01	8.34E-01
OR4A16	-0.01023	8.17E-01	8.49E-01
OR4A47	0.03165	4.74E-01	5.39E-01
OR4C3	-0.01974	6.55E-01	7.08E-01
OR4C6	0.202089	3.79E-06	1.07E-05
OR4D10	-0.08232	6.19E-02	8.97E-02
OR4D1	0.003141	9.43E-01	9.56E-01
OR4D2	-0.06035	1.71E-01	2.23E-01
OR4D5	0.033663	4.46E-01	5.12E-01
OR4D6	-0.01679	7.04E-01	7.52E-01
OR4E2	-0.12352	5.00E-03	8.98E-03
OR4F21	-0.02653	5.48E-01	6.10E-01
OR4F29	-0.03091	4.84E-01	5.49E-01
OR4F4	0.072057	1.02E-01	1.41E-01
OR4F5	-0.02139	6.28E-01	6.84E-01
OR4F6	-0.0411	3.52E-01	4.18E-01

OR4K17	-0.03167	4.73E-01	5.39E-01
OR4K1	0.031565	4.75E-01	5.40E-01
OR4M2	0.017265	6.96E-01	7.45E-01
OR4N2	-0.04954	2.62E-01	3.23E-01
OR4N3P	0.038969	3.77E-01	4.44E-01
OR4N4	0.059218	1.80E-01	2.32E-01
OR4X2	0.006621	8.81E-01	9.03E-01
OR51A7	0.06716	1.28E-01	1.72E-01
OR51B2	0.166139	1.52E-04	3.45E-04
OR51B4	0.144282	1.03E-03	2.06E-03
OR51B5	0.181797	3.32E-05	8.27E-05
OR51B6	0.111638	1.12E-02	1.89E-02
OR51E1	0.416078	5.59E-23	8.35E-22
OR51E2	0.17199	8.75E-05	2.05E-04
OR51F2	0.049162	2.65E-01	3.27E-01
OR51G2	0.089169	4.31E-02	6.44E-02
OR51I1	-0.03772	3.93E-01	4.60E-01
OR51I2	-0.05585	2.06E-01	2.61E-01
OR51M1	0.138726	1.60E-03	3.11E-03
OR51Q1	0.084449	5.55E-02	8.10E-02
OR51T1	0.055318	2.10E-01	2.66E-01
OR52A1	0.029909	4.98E-01	5.63E-01
OR52A4	0.110006	1.25E-02	2.08E-02
OR52A5	0.090011	4.12E-02	6.18E-02
OR52B2	0.012118	7.84E-01	8.21E-01
OR52B4	-0.01489	7.36E-01	7.80E-01
OR52B6	-0.18356	2.78E-05	6.99E-05
OR52D1	-0.07318	9.71E-02	1.34E-01
OR52E2	0.154308	4.41E-04	9.33E-04
OR52E4	0.068029	1.23E-01	1.66E-01
OR52E6	0.112155	1.09E-02	1.83E-02
OR52E8	0.022001	6.18E-01	6.75E-01
OR52H1	-0.05874	1.83E-01	2.36E-01
OR52I1	-0.08076	6.70E-02	9.64E-02
OR52I2	-0.03354	4.48E-01	5.14E-01
OR52K1	-0.01482	7.37E-01	7.81E-01
OR52K2	-0.12842	3.51E-03	6.46E-03
OR52L1	0.01454	7.42E-01	7.85E-01
OR52M1	-0.05801	1.89E-01	2.42E-01
OR52N1	-0.02403	5.86E-01	6.45E-01
OR52N2	-0.0509	2.49E-01	3.09E-01
OR52N4	-0.29592	7.22E-12	3.87E-11
OR52N5	0.049963	2.58E-01	3.19E-01
OR52R1	0.035589	4.20E-01	4.86E-01

OR52W1	-0.0491	2.66E-01	3.28E-01
OR56A1	0.120683	6.10E-03	1.08E-02
OR56A3	0.200104	4.73E-06	1.32E-05
OR56A4	0.030611	4.88E-01	5.53E-01
OR56A5	0.037779	3.92E-01	4.59E-01
OR56B1	-0.27634	1.76E-10	8.21E-10
OR56B4	0.021198	6.31E-01	6.87E-01
OR5A1	-0.06574	1.36E-01	1.82E-01
OR5A2	-0.06481	1.42E-01	1.88E-01
OR5AC2	-0.00959	8.28E-01	8.59E-01
OR5AK2	-0.15429	4.41E-04	9.34E-04
OR5AN1	-0.00382	9.31E-01	9.45E-01
OR5AU1	-0.09642	2.87E-02	4.44E-02
OR5B12	0.035755	4.18E-01	4.84E-01
OR5B21	-0.02797	5.27E-01	5.90E-01
OR5B2	-0.05854	1.85E-01	2.38E-01
OR5B3	0.019221	6.63E-01	7.16E-01
OR5C1	-0.0993	2.42E-02	3.81E-02
OR5E1P	-0.08085	6.68E-02	9.61E-02
OR5H1	-0.06419	1.46E-01	1.93E-01
OR5H2	0.018767	6.71E-01	7.22E-01
OR5H6	0.052891	2.31E-01	2.89E-01
OR5K1	-0.21095	1.37E-06	4.07E-06
OR5K2	-0.2704	4.43E-10	1.98E-09
OR5M11	0.060713	1.69E-01	2.20E-01
OR5M1	0.043641	3.23E-01	3.88E-01
OR5P2	-0.13496	2.15E-03	4.09E-03
OR5P3	-0.10603	1.61E-02	2.63E-02
OR5T2	0.016965	7.01E-01	7.49E-01
OR5T3	0.000637	9.88E-01	9.91E-01
OR5V1	-0.00103	9.81E-01	9.86E-01
OR6A2	0.046678	2.90E-01	3.53E-01
OR6B1	0.004749	9.14E-01	9.32E-01
OR6B2	0.090215	4.07E-02	6.12E-02
OR6B3	0.001999	9.64E-01	9.72E-01
OR6C2	-0.0177	6.89E-01	7.38E-01
OR6C3	0.029307	5.07E-01	5.71E-01
OR6C6	-0.00439	9.21E-01	9.37E-01
OR6C70	-0.01549	7.26E-01	7.71E-01
OR6C75	0.026144	5.54E-01	6.15E-01
OR6C76	-0.07269	9.94E-02	1.37E-01
OR6F1	0.07302	9.79E-02	1.35E-01
OR6K3	-0.15336	4.79E-04	1.01E-03
OR6K6	0.025114	5.70E-01	6.29E-01

OR6M1	0.007189	8.71E-01	8.95E-01
OR6N1	-0.10761	1.46E-02	2.40E-02
OR6N2	-0.05496	2.13E-01	2.69E-01
OR6S1	-0.07776	7.79E-02	1.10E-01
OR6T1	-0.00167	9.70E-01	9.77E-01
OR6V1	0.038869	3.79E-01	4.45E-01
OR6W1P	0.04109	3.52E-01	4.18E-01
OR7A5	0.143256	1.11E-03	2.22E-03
OR7C1	-0.00162	9.71E-01	9.77E-01
OR7D2	-0.05274	2.32E-01	2.91E-01
OR7D4	-0.05653	2.00E-01	2.55E-01
OR7E156P	0.102481	2.00E-02	3.20E-02
OR7E24	-0.06372	1.49E-01	1.96E-01
OR7E37P	-0.17919	4.32E-05	1.06E-04
OR7E5P	0.142542	1.18E-03	2.35E-03
OR7E91P	0.177453	5.13E-05	1.25E-04
OR7G2	-0.01814	6.81E-01	7.31E-01
OR7G3	0.008059	8.55E-01	8.82E-01
OR8A1	0.109148	1.32E-02	2.19E-02
OR8B12	0.041231	3.50E-01	4.16E-01
OR8B2	0.102321	2.02E-02	3.23E-02
OR8B3	0.060195	1.73E-01	2.24E-01
OR8B4	0.046184	2.96E-01	3.59E-01
OR8D1	0.09177	3.73E-02	5.65E-02
OR8D2	0.135651	2.03E-03	3.89E-03
OR8G2	0.032555	4.61E-01	5.27E-01
OR8G5	0.079642	7.09E-02	1.01E-01
OR8K3	0.029422	5.05E-01	5.70E-01
OR8S1	0.13555	2.05E-03	3.92E-03
OR9A2	0.046374	2.94E-01	3.56E-01
OR9A4	0.018002	6.84E-01	7.33E-01
OR9G4	-0.11714	7.79E-03	1.35E-02
OR9G9	0.005205	9.06E-01	9.25E-01
OR9K2	0.037047	4.01E-01	4.68E-01
OR9Q1	0.048695	2.70E-01	3.32E-01
ORAI1	0.0361	4.14E-01	4.80E-01
ORAI2	0.09644	2.86E-02	4.44E-02
ORAI3	-0.17677	5.49E-05	1.33E-04
ORAOV1	-0.02325	5.99E-01	6.56E-01
ORC1L	0.843125	2.48E-140	9.93E-138
ORC2L	0.319432	1.11E-13	7.17E-13
ORC3L	0.183086	2.91E-05	7.31E-05
ORC4L	0.248264	1.13E-08	4.27E-08
ORC5L	0.435362	3.12E-25	5.61E-24

ORC6L	0.7904	2.93E-111	5.80E-109
ORM1	-0.30639	1.18E-12	6.86E-12
ORM2	-0.30427	1.71E-12	9.83E-12
ORMDL1	-0.06383	1.48E-01	1.95E-01
ORMDL2	0.212946	1.08E-06	3.25E-06
ORMDL3	-0.45886	3.51E-28	7.93E-27
OS9	-0.1925	1.09E-05	2.89E-05
OSBP2	-0.11369	9.82E-03	1.67E-02
OSBPL10	0.116613	8.07E-03	1.39E-02
OSBPL11	0.087149	4.81E-02	7.11E-02
OSBPL1A	-0.24067	3.20E-08	1.15E-07
OSBPL2	-0.18222	3.18E-05	7.94E-05
OSBPL3	0.141759	1.26E-03	2.49E-03
OSBPL5	-0.22349	2.98E-07	9.67E-07
OSBPL6	-0.18253	3.08E-05	7.71E-05
OSBPL7	-0.28564	3.99E-11	1.96E-10
OSBPL8	0.209142	1.69E-06	4.97E-06
OSBPL9	-0.16313	2.01E-04	4.48E-04
OSBP	-0.05713	1.96E-01	2.50E-01
OSCAR	-0.1358	2.01E-03	3.85E-03
OSCP1	-0.37949	4.36E-19	4.68E-18
OSGEPL1	0.069788	1.14E-01	1.54E-01
OSGEP	-0.07229	1.01E-01	1.39E-01
OSGIN1	0.034226	4.38E-01	5.05E-01
OSGIN2	0.320546	9.04E-14	5.88E-13
OSMR	0.059863	1.75E-01	2.27E-01
OSM	0.09581	2.97E-02	4.59E-02
OSR1	-0.39729	6.41E-21	8.11E-20
OSR2	0.148559	7.20E-04	1.48E-03
OST4	0.158383	3.08E-04	6.69E-04
OSTBETA	-0.08585	5.15E-02	7.58E-02
OSTCL	0.115417	8.75E-03	1.50E-02
OSTC	0.302457	2.35E-12	1.33E-11
OSTF1	-0.00588	8.94E-01	9.14E-01
OSTM1	0.086796	4.90E-02	7.24E-02
OSTN	-0.0259	5.58E-01	6.19E-01
OSTalpha	0.068904	1.18E-01	1.60E-01
OTC	-0.4037	1.32E-21	1.75E-20
OTOA	-0.23368	8.12E-08	2.80E-07
OTOF	0.178689	4.54E-05	1.11E-04
OTOL1	0.026185	5.53E-01	6.15E-01
OTOP1	0.018183	6.81E-01	7.31E-01
OTOP2	0.181241	3.51E-05	8.72E-05
OTOP3	0.17003	1.06E-04	2.45E-04

OTOR	0.030238	4.94E-01	5.58E-01
OTOS	0.143874	1.06E-03	2.12E-03
OTP	0.191456	1.22E-05	3.22E-05
OTUB1	0.118386	7.15E-03	1.25E-02
OTUB2	0.318231	1.39E-13	8.87E-13
OTUD1	-0.23404	7.73E-08	2.67E-07
OTUD3	-0.19711	6.59E-06	1.81E-05
OTUD4	-0.00519	9.06E-01	9.25E-01
OTUD5	-0.2196	4.83E-07	1.52E-06
OTUD6A	0.054012	2.21E-01	2.79E-01
OTUD6B	0.357691	5.46E-17	4.87E-16
OTUD7A	-0.341	1.73E-15	1.31E-14
OTUD7B	-0.17787	4.93E-05	1.20E-04
OTX1	0.263752	1.21E-09	5.12E-09
OTX2	0.184648	2.48E-05	6.29E-05
OVCA2	0.12772	3.69E-03	6.77E-03
OVCH1	-0.26035	2.00E-09	8.27E-09
OVCH2	-0.23022	1.27E-07	4.29E-07
OVGP1	-0.40523	8.96E-22	1.21E-20
OVOL1	0.067621	1.25E-01	1.69E-01
OVOL2	-0.12191	5.60E-03	9.97E-03
OXA1L	-0.03331	4.51E-01	5.17E-01
OXCT1	0.170055	1.05E-04	2.45E-04
OXCT2	-0.09438	3.23E-02	4.95E-02
OXER1	-0.13551	2.06E-03	3.93E-03
OXGR1	0.071453	1.05E-01	1.44E-01
OXNAD1	0.155068	4.13E-04	8.77E-04
OXR1	-0.12473	4.59E-03	8.29E-03
OXSM	0.055929	2.05E-01	2.61E-01
OXSR1	0.153762	4.62E-04	9.75E-04
OXTR	0.050073	2.57E-01	3.18E-01
OXT	-0.1011	2.18E-02	3.46E-02
P2RX1	-0.20369	3.16E-06	9.03E-06
P2RX2	-0.35008	2.71E-16	2.24E-15
P2RX3	-0.03088	4.84E-01	5.49E-01
P2RX4	-0.04443	3.14E-01	3.79E-01
P2RX5	0.1212	5.89E-03	1.04E-02
P2RX6	-0.13238	2.61E-03	4.91E-03
P2RX7	-0.05462	2.16E-01	2.73E-01
P2RY10	-0.02581	5.59E-01	6.20E-01
P2RY11	-0.06573	1.36E-01	1.82E-01
P2RY12	-0.29831	4.80E-12	2.63E-11
P2RY13	-0.19537	7.97E-06	2.16E-05
P2RY14	-0.26394	1.18E-09	4.99E-09

P2RY1	0.03075	4.86E-01	5.51E-01
P2RY2	-0.20193	3.86E-06	1.09E-05
P2RY4	0.075792	8.57E-02	1.20E-01
P2RY6	0.232553	9.40E-08	3.22E-07
P2RY8	-0.21653	7.02E-07	2.17E-06
P4HA1	0.348547	3.72E-16	3.03E-15
P4HA2	0.107717	1.45E-02	2.38E-02
P4HA3	0.159816	2.71E-04	5.94E-04
P4HB	0.101708	2.10E-02	3.34E-02
P4HTM	-0.33361	7.47E-15	5.37E-14
P704P	0.129559	3.22E-03	5.98E-03
PA2G4P4	0.376079	9.53E-19	9.89E-18
PA2G4	0.530237	1.12E-38	4.75E-37
PAAF1	0.039433	3.72E-01	4.38E-01
PABPC1L2A	-0.14713	8.11E-04	1.65E-03
PABPC1L2B	-0.19969	4.95E-06	1.38E-05
PABPC1L	-0.14521	9.50E-04	1.92E-03
PABPC1P2	0.051947	2.39E-01	2.99E-01
PABPC1	0.099334	2.42E-02	3.81E-02
PABPC3	0.121444	5.79E-03	1.03E-02
PABPC4L	0.117031	7.85E-03	1.36E-02
PABPC4	-0.05968	1.76E-01	2.28E-01
PABPC5	-0.13404	2.30E-03	4.36E-03
PABPN1L	0.039313	3.73E-01	4.39E-01
PABPN1	0.055831	2.06E-01	2.61E-01
PACRGL	0.166833	1.43E-04	3.25E-04
PACRG	-0.27428	2.43E-10	1.12E-09
PACS1	0.129735	3.18E-03	5.90E-03
PACS2	-0.07497	8.92E-02	1.24E-01
PACSIN1	0.24921	9.90E-09	3.78E-08
PACSIN2	-0.22532	2.37E-07	7.77E-07
PACSIN3	-0.15147	5.63E-04	1.17E-03
PADI1	0.181682	3.36E-05	8.36E-05
PADI2	-0.06441	1.44E-01	1.91E-01
PADI3	0.188533	1.66E-05	4.31E-05
PADI4	-0.02286	6.05E-01	6.62E-01
PADI6	0.040583	3.58E-01	4.24E-01
PAEP	0.104673	1.75E-02	2.84E-02
PAF1	0.061104	1.66E-01	2.17E-01
PAFAH1B1	-0.23506	6.77E-08	2.35E-07
PAFAH1B2	0.21459	8.86E-07	2.70E-06
PAFAH1B3	0.357124	6.16E-17	5.46E-16
PAFAH2	-0.15283	5.01E-04	1.05E-03
PAG1	-0.21904	5.18E-07	1.63E-06

PAGE1	0.199714	4.94E-06	1.38E-05
PAGE2B	0.160957	2.45E-04	5.39E-04
PAGE2	0.188374	1.68E-05	4.38E-05
PAGE3	0.053511	2.25E-01	2.83E-01
PAGE4	0.106092	1.60E-02	2.62E-02
PAGE5	0.155004	4.15E-04	8.82E-04
PAH	0.158778	2.98E-04	6.47E-04
PAICS	0.598018	2.93E-51	2.04E-49
PAIP1	0.313147	3.52E-13	2.16E-12
PAIP2B	-0.191	1.28E-05	3.37E-05
PAIP2	-0.04678	2.89E-01	3.52E-01
PAK1IP1	0.476063	1.72E-30	4.58E-29
PAK1	0.207052	2.15E-06	6.25E-06
PAK2	0.424062	6.79E-24	1.10E-22
PAK3	-0.21847	5.54E-07	1.74E-06
PAK4	0.192738	1.06E-05	2.82E-05
PAK6	-0.13896	1.57E-03	3.06E-03
PAK7	-0.0964	2.87E-02	4.45E-02
PALB2	0.282669	6.45E-11	3.12E-10
PALLD	0.170446	1.01E-04	2.36E-04
PALM2-AKAP2	-0.00638	8.85E-01	9.07E-01
PALM2	-0.01362	7.58E-01	7.98E-01
PALM3	-0.2771	1.56E-10	7.31E-10
PALMD	-0.40489	9.76E-22	1.31E-20
PALM	-0.28099	8.45E-11	4.05E-10
PAMR1	-0.22821	1.65E-07	5.49E-07
PAM	-0.30275	2.23E-12	1.27E-11
PAN2	-0.2151	8.34E-07	2.55E-06
PAN3	-0.23369	8.10E-08	2.79E-07
PANK1	-0.01194	7.87E-01	8.23E-01
PANK2	0.08786	4.63E-02	6.87E-02
PANK3	0.287711	2.84E-11	1.43E-10
PANK4	-0.00336	9.39E-01	9.53E-01
PANX1	0.27629	1.78E-10	8.27E-10
PANX2	0.011021	8.03E-01	8.37E-01
PANX3	0.094579	3.19E-02	4.89E-02
PAOX	-0.23701	5.23E-08	1.84E-07
PAPD4	-0.11997	6.41E-03	1.13E-02
PAPD5	-0.15194	5.41E-04	1.13E-03
PAPD7	0.077762	7.79E-02	1.10E-01
PAPLN	-0.29279	1.22E-11	6.38E-11
PAPL	0.26174	1.63E-09	6.80E-09
PAPOLA	0.198063	5.93E-06	1.64E-05
PAPOLB	-0.01402	7.51E-01	7.93E-01

PAPOLG	0.167145	1.39E-04	3.16E-04
PAPPA2	-0.21297	1.07E-06	3.24E-06
PAPPA	-0.24586	1.58E-08	5.87E-08
PAPSS1	-0.06703	1.29E-01	1.73E-01
PAPSS2	-0.15687	3.52E-04	7.58E-04
PAQR3	0.285097	4.36E-11	2.14E-10
PAQR4	0.296887	6.12E-12	3.31E-11
PAQR5	-0.1103	1.23E-02	2.05E-02
PAQR6	0.049457	2.63E-01	3.24E-01
PAQR7	-0.0645	1.44E-01	1.90E-01
PAQR8	-0.20997	1.53E-06	4.53E-06
PAQR9	0.314921	2.55E-13	1.59E-12
PAR-SN	-0.27717	1.55E-10	7.24E-10
PAR1	-0.31242	4.01E-13	2.45E-12
PAR4	-0.0203	6.46E-01	7.00E-01
PAR5	-0.19161	1.20E-05	3.17E-05
PARD3B	-0.28958	2.09E-11	1.06E-10
PARD3	0.195388	7.95E-06	2.16E-05
PARD6A	-0.04835	2.73E-01	3.35E-01
PARD6B	-0.22448	2.64E-07	8.61E-07
PARD6G	0.116561	8.10E-03	1.40E-02
PARG	0.179606	4.14E-05	1.02E-04
PARK2	-0.26075	1.89E-09	7.82E-09
PARK7	0.08929	4.28E-02	6.40E-02
PARL	0.357743	5.40E-17	4.82E-16
PARM1	-0.50196	3.20E-34	1.06E-32
PARN	-0.40629	6.87E-22	9.36E-21
PARP10	-0.18396	2.66E-05	6.72E-05
PARP11	-0.21245	1.14E-06	3.44E-06
PARP12	0.25788	2.87E-09	1.16E-08
PARP14	-0.04371	3.22E-01	3.87E-01
PARP15	-0.26026	2.03E-09	8.37E-09
PARP16	-0.27471	2.28E-10	1.05E-09
PARP1	0.280243	9.52E-11	4.53E-10
PARP2	0.435241	3.22E-25	5.79E-24
PARP3	-0.35684	6.55E-17	5.78E-16
PARP4	-0.22655	2.03E-07	6.71E-07
PARP6	-0.08731	4.77E-02	7.06E-02
PARP8	-0.16951	1.11E-04	2.57E-04
PARP9	0.223408	3.02E-07	9.76E-07
PARS2	0.084125	5.64E-02	8.23E-02
PART1	-0.09172	3.75E-02	5.67E-02
PARVA	-0.31704	1.73E-13	1.10E-12
PARVB	0.202086	3.79E-06	1.07E-05

PARVG	-0.19586	7.56E-06	2.05E-05
PASD1	0.128187	3.57E-03	6.56E-03
PASK	0.074179	9.26E-02	1.29E-01
PATE1	0.024081	5.86E-01	6.44E-01
PATE2	0.291381	1.55E-11	7.99E-11
PATE3	-0.00926	8.34E-01	8.63E-01
PATE4	0.185167	2.35E-05	5.99E-05
PATL1	0.467911	2.22E-29	5.50E-28
PATL2	-0.13787	1.71E-03	3.31E-03
PATZ1	-0.12623	4.11E-03	7.49E-03
PAWR	0.496094	2.39E-33	7.48E-32
PAX1	-0.04754	2.82E-01	3.44E-01
PAX2	0.086668	4.93E-02	7.28E-02
PAX3	0.111335	1.15E-02	1.93E-02
PAX4	0.047252	2.84E-01	3.47E-01
PAX5	-0.09121	3.85E-02	5.81E-02
PAX6	-0.18222	3.18E-05	7.94E-05
PAX7	-0.30551	1.38E-12	7.97E-12
PAX8	0.046773	2.89E-01	3.52E-01
PAX9	0.221729	3.72E-07	1.19E-06
PAXIP1	0.267414	6.98E-10	3.04E-09
PBK	0.799453	1.27E-115	2.74E-113
PBLD	-0.29934	4.03E-12	2.22E-11
PBOV1	-0.25153	7.14E-09	2.77E-08
PBRM1	-0.11101	1.17E-02	1.96E-02
PBX1	-0.1807	3.71E-05	9.18E-05
PBX2	-0.21456	8.89E-07	2.71E-06
PBX3	-0.02663	5.47E-01	6.09E-01
PBX4	-0.12908	3.34E-03	6.17E-03
PBXIP1	-0.61818	1.31E-55	1.01E-53
PCA3	-0.0796	7.11E-02	1.02E-01
PCBD1	-0.1466	8.47E-04	1.72E-03
PCBD2	-0.37345	1.73E-18	1.76E-17
PCBP1	0.221923	3.63E-07	1.16E-06
PCBP2	0.312698	3.82E-13	2.34E-12
PCBP3	-0.17144	9.23E-05	2.16E-04
PCBP4	-0.06053	1.70E-01	2.21E-01
PCCA	-0.323	5.71E-14	3.79E-13
PCCB	0.203638	3.18E-06	9.08E-06
PCDH10	-0.17515	6.44E-05	1.54E-04
PCDH11X	-0.30382	1.85E-12	1.06E-11
PCDH11Y	-0.18663	2.02E-05	5.19E-05
PCDH12	-0.18257	3.07E-05	7.68E-05
PCDH15	-0.37941	4.45E-19	4.76E-18

PCDH17	-0.15972	2.74E-04	5.99E-04
PCDH18	-0.09835	2.56E-02	4.01E-02
PCDH19	-0.05774	1.91E-01	2.45E-01
PCDH1	-0.22802	1.69E-07	5.61E-07
PCDH20	-0.42263	9.96E-24	1.60E-22
PCDH7	0.234718	7.08E-08	2.45E-07
PCDH8	-0.04721	2.85E-01	3.47E-01
PCDH9	-0.20205	3.80E-06	1.08E-05
PCDHA10	-0.2433	2.24E-08	8.21E-08
PCDHA11	-0.09641	2.87E-02	4.45E-02
PCDHA12	-0.17385	7.31E-05	1.74E-04
PCDHA13	-0.09077	3.95E-02	5.95E-02
PCDHA1	0.192917	1.04E-05	2.77E-05
PCDHA2	0.038465	3.84E-01	4.50E-01
PCDHA3	-0.15975	2.73E-04	5.97E-04
PCDHA4	-0.01796	6.84E-01	7.34E-01
PCDHA5	0.054145	2.20E-01	2.77E-01
PCDHA6	-0.04043	3.60E-01	4.26E-01
PCDHA7	-0.02399	5.87E-01	6.46E-01
PCDHA8	-0.12184	5.63E-03	1.00E-02
PCDHA9	-0.06184	1.61E-01	2.11E-01
PCDHAC1	-0.02685	5.43E-01	6.05E-01
PCDHAC2	-0.16155	2.32E-04	5.13E-04
PCDHB10	0.01994	6.52E-01	7.05E-01
PCDHB11	-0.02783	5.29E-01	5.92E-01
PCDHB12	-0.11973	6.52E-03	1.15E-02
PCDHB13	0.067159	1.28E-01	1.72E-01
PCDHB14	-0.0137	7.56E-01	7.97E-01
PCDHB15	-0.12491	4.53E-03	8.19E-03
PCDHB16	-0.02629	5.52E-01	6.13E-01
PCDHB17	0.145837	9.02E-04	1.83E-03
PCDHB18	-0.06982	1.14E-01	1.54E-01
PCDHB19P	-0.0073	8.69E-01	8.93E-01
PCDHB1	-0.14631	8.68E-04	1.76E-03
PCDHB2	0.17453	6.84E-05	1.63E-04
PCDHB3	-0.07662	8.24E-02	1.16E-01
PCDHB4	-0.26135	1.73E-09	7.19E-09
PCDHB5	-0.0007	9.87E-01	9.90E-01
PCDHB6	0.040547	3.58E-01	4.25E-01
PCDHB7	-0.13785	1.71E-03	3.32E-03
PCDHB8	0.216111	7.38E-07	2.27E-06
PCDHB9	0.059688	1.76E-01	2.28E-01
PCDHGA10	-0.11769	7.51E-03	1.30E-02
PCDHGA11	-0.02798	5.26E-01	5.90E-01

PCDHGA12	-0.16475	1.73E-04	3.90E-04
PCDHGA1	0.194545	8.72E-06	2.35E-05
PCDHGA2	-0.06707	1.29E-01	1.72E-01
PCDHGA3	-0.10376	1.85E-02	2.98E-02
PCDHGA4	-0.0455	3.03E-01	3.66E-01
PCDHGA5	-0.14602	8.89E-04	1.80E-03
PCDHGA6	-0.1624	2.15E-04	4.78E-04
PCDHGA7	-0.09451	3.20E-02	4.91E-02
PCDHGA8	0.045654	3.01E-01	3.65E-01
PCDHGA9	-0.15677	3.56E-04	7.65E-04
PCDHGB1	0.158434	3.07E-04	6.66E-04
PCDHGB2	-0.10561	1.65E-02	2.69E-02
PCDHGB3	-0.06698	1.29E-01	1.73E-01
PCDHGB4	0.05848	1.85E-01	2.38E-01
PCDHGB5	0.015288	7.29E-01	7.74E-01
PCDHGB6	-0.18209	3.23E-05	8.04E-05
PCDHGB7	-0.27035	4.46E-10	1.99E-09
PCDHGB8P	-0.03817	3.87E-01	4.54E-01
PCDHGC3	-0.22921	1.45E-07	4.86E-07
PCDHGC4	-0.07583	8.56E-02	1.20E-01
PCDHGC5	0.004669	9.16E-01	9.33E-01
PCDP1	-0.568	2.51E-45	1.48E-43
PCF11	-0.24282	2.40E-08	8.74E-08
PCGEM1	0.070059	1.12E-01	1.53E-01
PCGF1	0.089382	4.26E-02	6.38E-02
PCGF2	0.006287	8.87E-01	9.08E-01
PCGF3	-0.01065	8.09E-01	8.42E-01
PCGF5	0.020992	6.35E-01	6.90E-01
PCGF6	0.412583	1.38E-22	2.00E-21
PCID2	0.050301	2.55E-01	3.15E-01
PCIF1	-0.05471	2.15E-01	2.72E-01
PCK1	0.089423	4.25E-02	6.36E-02
PCK2	0.056906	1.97E-01	2.52E-01
PCLO	-0.00446	9.20E-01	9.36E-01
PCM1	-0.13528	2.09E-03	3.99E-03
PCMT1	0.33578	4.88E-15	3.57E-14
PCMTD1	-0.28623	3.62E-11	1.80E-10
PCMTD2	-0.26625	8.33E-10	3.59E-09
PCNAP1	-0.09231	3.62E-02	5.50E-02
PCNA	0.624015	6.28E-57	5.08E-55
PCNP	0.207354	2.08E-06	6.05E-06
PCNT	-0.07643	8.31E-02	1.17E-01
PCNXL2	0.026853	5.43E-01	6.05E-01
PCNXL3	0.135189	2.11E-03	4.02E-03

PCNX	-0.15225	5.27E-04	1.10E-03
PCOLCE2	0.044808	3.10E-01	3.74E-01
PCOLCE	0.010178	8.18E-01	8.50E-01
PCOTH	-0.24237	2.54E-08	9.26E-08
PCP2	-0.249	1.02E-08	3.88E-08
PCP4L1	-0.3336	7.49E-15	5.38E-14
PCP4	0.028205	5.23E-01	5.87E-01
PCSK1N	-0.09806	2.61E-02	4.07E-02
PCSK1	0.286688	3.36E-11	1.67E-10
PCSK2	-0.4087	3.73E-22	5.21E-21
PCSK4	-0.25586	3.85E-09	1.54E-08
PCSK5	-0.26702	7.41E-10	3.22E-09
PCSK6	-0.09874	2.50E-02	3.93E-02
PCSK7	-0.17629	5.76E-05	1.39E-04
PCSK9	-0.08702	4.84E-02	7.16E-02
PCTP	0.00306	9.45E-01	9.57E-01
PCYOX1L	-0.01064	8.10E-01	8.43E-01
PCYOX1	-0.28736	3.01E-11	1.51E-10
PCYT1A	0.342628	1.24E-15	9.63E-15
PCYT1B	-0.15349	4.73E-04	9.97E-04
PCYT2	-0.04263	3.34E-01	4.00E-01
PC	-0.1473	7.99E-04	1.63E-03
PDAP1	0.346853	5.27E-16	4.25E-15
PDCD10	0.409496	3.05E-22	4.28E-21
PDCD11	0.20504	2.71E-06	7.79E-06
PDCD1LG2	0.178737	4.52E-05	1.11E-04
PDCD1	0.155685	3.91E-04	8.35E-04
PDCD2L	0.313406	3.36E-13	2.07E-12
PDCD2	0.285329	4.19E-11	2.06E-10
PDCD4	-0.36822	5.55E-18	5.38E-17
PDCD5	0.422585	1.01E-23	1.61E-22
PDCD6IP	-0.12346	5.02E-03	9.01E-03
PDCD6	0.176958	5.39E-05	1.31E-04
PDCD7	-0.01232	7.80E-01	8.18E-01
PDCL2	0.087533	4.71E-02	6.98E-02
PDCL3	0.389791	3.92E-20	4.63E-19
PDCL	0.101598	2.11E-02	3.36E-02
PDC	0.020299	6.46E-01	7.00E-01
PDDC1	-0.22923	1.44E-07	4.85E-07
PDE10A	0.193679	9.57E-06	2.57E-05
PDE11A	-0.02177	6.22E-01	6.78E-01
PDE12	0.068132	1.23E-01	1.65E-01
PDE1A	-0.26705	7.38E-10	3.21E-09
PDE1B	-0.17571	6.09E-05	1.46E-04

PDE1C	-0.09621	2.90E-02	4.49E-02
PDE2A	-0.21306	1.06E-06	3.21E-06
PDE3A	0.005484	9.01E-01	9.20E-01
PDE3B	-0.16993	1.07E-04	2.47E-04
PDE4A	-0.33541	5.25E-15	3.83E-14
PDE4B	-0.08623	5.05E-02	7.44E-02
PDE4C	-0.35481	1.01E-16	8.71E-16
PDE4DIP	-0.14886	7.02E-04	1.44E-03
PDE4D	-0.26491	1.02E-09	4.35E-09
PDE5A	-0.19787	6.06E-06	1.67E-05
PDE6A	0.207666	2.00E-06	5.85E-06
PDE6B	-0.18736	1.87E-05	4.84E-05
PDE6C	-0.06603	1.35E-01	1.80E-01
PDE6D	0.260689	1.90E-09	7.88E-09
PDE6G	-0.00327	9.41E-01	9.54E-01
PDE6H	-0.02857	5.18E-01	5.82E-01
PDE7A	0.033299	4.51E-01	5.17E-01
PDE7B	-0.33318	8.12E-15	5.80E-14
PDE8A	-0.31339	3.37E-13	2.08E-12
PDE8B	-0.35033	2.57E-16	2.14E-15
PDE9A	-0.18347	2.80E-05	7.05E-05
PDF	0.172169	8.60E-05	2.02E-04
PDGFA	-0.28688	3.25E-11	1.62E-10
PDGFB	0.058375	1.86E-01	2.39E-01
PDGFC	-0.0903	4.05E-02	6.09E-02
PDGFD	-0.16164	2.30E-04	5.10E-04
PDGFRA	-0.12918	3.32E-03	6.13E-03
PDGFRB	-0.16583	1.57E-04	3.55E-04
PDGFRL	0.048627	2.71E-01	3.33E-01
PDHA1	0.094446	3.21E-02	4.93E-02
PDHA2	0.054746	2.15E-01	2.71E-01
PDHB	-0.03417	4.39E-01	5.06E-01
PDHX	0.287347	3.02E-11	1.51E-10
PDIA2	-0.01877	6.71E-01	7.22E-01
PDIA3P	-0.02207	6.17E-01	6.74E-01
PDIA3	-0.03596	4.15E-01	4.82E-01
PDIA4	0.275293	2.08E-10	9.61E-10
PDIA5	0.108107	1.41E-02	2.33E-02
PDIA6	0.298696	4.50E-12	2.47E-11
PDIK1L	-0.27135	3.83E-10	1.72E-09
PDILT	-0.09206	3.68E-02	5.57E-02
PDK1	0.440348	7.71E-26	1.46E-24
PDK2	-0.46336	9.00E-29	2.12E-27
PDK3	0.37209	2.35E-18	2.36E-17

PDK4	-0.22869	1.55E-07	5.17E-07
PDLIM1	-0.18305	2.92E-05	7.33E-05
PDLIM2	-0.39005	3.69E-20	4.37E-19
PDLIM3	0.037305	3.98E-01	4.65E-01
PDLIM4	0.079864	7.02E-02	1.00E-01
PDLIM5	0.235272	6.58E-08	2.29E-07
PDLIM7	0.165071	1.68E-04	3.79E-04
PDP1	-0.00268	9.52E-01	9.62E-01
PDP2	-0.13102	2.89E-03	5.40E-03
PDPK1	-0.38965	4.05E-20	4.78E-19
PDPN	0.097042	2.77E-02	4.30E-02
PDPR	-0.20621	2.37E-06	6.85E-06
PDRG1	0.374365	1.41E-18	1.44E-17
PDS5A	0.01849	6.75E-01	7.26E-01
PDS5B	0.015202	7.31E-01	7.75E-01
PDSS1	0.569421	1.35E-45	8.05E-44
PDSS2	-0.01546	7.26E-01	7.72E-01
PDX1	0.128905	3.38E-03	6.25E-03
PDXDC1	-0.18629	2.09E-05	5.37E-05
PDXDC2	-0.33469	6.05E-15	4.39E-14
PDXK	-0.00953	8.29E-01	8.59E-01
PDXP	0.220839	4.15E-07	1.32E-06
PDYN	-0.03819	3.87E-01	4.54E-01
PDZD11	0.389569	4.13E-20	4.87E-19
PDZD2	-0.52304	1.67E-37	6.54E-36
PDZD3	0.02397	5.87E-01	6.46E-01
PDZD4	-0.25836	2.68E-09	1.09E-08
PDZD7	0.147224	8.05E-04	1.64E-03
PDZD8	0.090248	4.06E-02	6.11E-02
PDZD9	-0.18757	1.83E-05	4.74E-05
PDZK1IP1	-0.17339	7.65E-05	1.81E-04
PDZK1P1	-0.16271	2.09E-04	4.65E-04
PDZK1	0.018159	6.81E-01	7.31E-01
PDZRN3	-0.20702	2.16E-06	6.27E-06
PDZRN4	-0.21658	6.97E-07	2.16E-06
PEA15	0.001031	9.81E-01	9.86E-01
PEAR1	-0.30816	8.61E-13	5.10E-12
PEBP1	-0.32061	8.94E-14	5.82E-13
PEBP4	-0.50079	4.79E-34	1.57E-32
PECAM1	-0.29318	1.15E-11	6.00E-11
PECI	-0.06167	1.62E-01	2.12E-01
PECR	0.130161	3.08E-03	5.73E-03
PEF1	-0.23735	5.00E-08	1.76E-07
PEG10	0.132504	2.59E-03	4.86E-03

PEG3AS	-0.0594	1.78E-01	2.31E-01
PEG3	-0.33363	7.44E-15	5.35E-14
PELI1	-0.1196	6.58E-03	1.15E-02
PELI2	-0.25459	4.62E-09	1.83E-08
PELI3	-0.02014	6.48E-01	7.02E-01
PELO	0.127064	3.87E-03	7.08E-03
PELP1	0.029392	5.06E-01	5.70E-01
PEMT	-0.10594	1.62E-02	2.64E-02
PENK	-0.40349	1.39E-21	1.84E-20
PEPD	0.038844	3.79E-01	4.45E-01
PER1	-0.41381	1.01E-22	1.47E-21
PER2	-0.26025	2.03E-09	8.38E-09
PER3	-0.35408	1.17E-16	1.01E-15
PER4	0.045313	3.05E-01	3.68E-01
PERP	0.269806	4.85E-10	2.16E-09
PES1	0.251457	7.22E-09	2.80E-08
PET112L	0.122859	5.24E-03	9.37E-03
PEX10	0.013688	7.57E-01	7.98E-01
PEX11A	-0.2714	3.80E-10	1.71E-09
PEX11B	-0.32132	7.82E-14	5.12E-13
PEX11G	-0.31085	5.32E-13	3.22E-12
PEX12	-0.05343	2.26E-01	2.84E-01
PEX13	0.07721	8.00E-02	1.13E-01
PEX14	-0.07345	9.59E-02	1.33E-01
PEX16	-0.09776	2.65E-02	4.14E-02
PEX19	-0.05249	2.34E-01	2.93E-01
PEX1	0.134826	2.17E-03	4.12E-03
PEX26	0.142152	1.22E-03	2.42E-03
PEX2	-0.02647	5.49E-01	6.11E-01
PEX3	0.102461	2.00E-02	3.21E-02
PEX5L	-0.01732	6.95E-01	7.44E-01
PEX5	0.05417	2.20E-01	2.77E-01
PEX6	-0.07411	9.29E-02	1.29E-01
PEX7	-0.09623	2.90E-02	4.49E-02
PF4V1	0.06713	1.28E-01	1.72E-01
PF4	0.034375	4.36E-01	5.03E-01
PFAS	0.006864	8.77E-01	9.00E-01
PFDN1	0.043968	3.19E-01	3.84E-01
PFDN2	0.341167	1.67E-15	1.27E-14
PFDN4	0.423919	7.06E-24	1.14E-22
PFDN5	0.023078	6.01E-01	6.59E-01
PFDN6	0.248952	1.03E-08	3.91E-08
PFKFB1	-0.01754	6.91E-01	7.41E-01
PFKFB2	-0.28775	2.82E-11	1.42E-10

PFKFB3	-0.02771	5.30E-01	5.94E-01
PFKFB4	0.440825	6.74E-26	1.28E-24
PFKL	-0.06091	1.68E-01	2.18E-01
PFKM	0.191374	1.23E-05	3.24E-05
PFKP	0.477925	9.53E-31	2.58E-29
PFN1	0.246676	1.41E-08	5.27E-08
PFN2	0.465658	4.45E-29	1.08E-27
PFN3	0.110304	1.23E-02	2.05E-02
PFN4	0.167079	1.39E-04	3.18E-04
PGA3	-0.08452	5.53E-02	8.08E-02
PGA4	-0.04477	3.11E-01	3.75E-01
PGA5	-0.07282	9.88E-02	1.36E-01
PGAM1	0.548645	8.17E-42	4.10E-40
PGAM2	-0.05992	1.75E-01	2.26E-01
PGAM4	0.461271	1.70E-28	3.91E-27
PGAM5	0.563761	1.54E-44	8.78E-43
PGAP1	-0.02323	5.99E-01	6.57E-01
PGAP2	-0.0981	2.60E-02	4.06E-02
PGAP3	-0.40668	6.22E-22	8.52E-21
PGBD1	0.109949	1.25E-02	2.09E-02
PGBD2	-0.32823	2.12E-14	1.46E-13
PGBD3	-0.02192	6.20E-01	6.76E-01
PGBD4	-0.05818	1.87E-01	2.41E-01
PGBD5	0.07302	9.79E-02	1.35E-01
PGCP	-0.43356	5.14E-25	9.06E-24
PGC	-0.50548	9.39E-35	3.17E-33
PGD	0.208009	1.92E-06	5.63E-06
PGF	0.183069	2.92E-05	7.32E-05
PGGT1B	-0.0457	3.01E-01	3.64E-01
PGK1	0.461272	1.70E-28	3.91E-27
PGK2	0.114036	9.60E-03	1.63E-02
PGLS	-0.0603	1.72E-01	2.23E-01
PGLYRP1	-0.04012	3.64E-01	4.30E-01
PGLYRP2	0.096984	2.78E-02	4.31E-02
PGLYRP3	-0.0941	3.28E-02	5.02E-02
PGLYRP4	-0.14218	1.22E-03	2.41E-03
PGM1	0.208734	1.77E-06	5.21E-06
PGM2L1	0.266901	7.54E-10	3.28E-09
PGM2	0.43557	2.94E-25	5.30E-24
PGM3	0.467704	2.37E-29	5.84E-28
PGM5P2	-0.27151	3.74E-10	1.68E-09
PGM5	-0.47147	7.35E-30	1.87E-28
PGPEP1L	0.01139	7.97E-01	8.32E-01
PGPEP1	-0.42411	6.71E-24	1.09E-22

PGP	0.285493	4.08E-11	2.01E-10
PGRMC1	-0.10471	1.75E-02	2.83E-02
PGRMC2	-0.06269	1.55E-01	2.04E-01
PGR	-0.45842	4.01E-28	9.02E-27
PGS1	-0.28765	2.87E-11	1.44E-10
PHACTR1	-0.47398	3.33E-30	8.72E-29
PHACTR2	-0.18313	2.90E-05	7.28E-05
PHACTR3	-0.20126	4.16E-06	1.17E-05
PHACTR4	-0.0373	3.98E-01	4.65E-01
PHAX	0.102363	2.02E-02	3.22E-02
PHB2	0.240831	3.14E-08	1.13E-07
PHB	0.385557	1.07E-19	1.21E-18
PHC1	-0.13995	1.45E-03	2.85E-03
PHC2	-0.03111	4.81E-01	5.46E-01
PHC3	-0.22897	1.49E-07	5.00E-07
PHEX	0.162857	2.06E-04	4.59E-04
PHF10	0.005315	9.04E-01	9.23E-01
PHF11	-0.25136	7.32E-09	2.83E-08
PHF12	-0.12788	3.65E-03	6.70E-03
PHF13	0.22021	4.48E-07	1.42E-06
PHF14	0.153576	4.70E-04	9.90E-04
PHF15	-0.28383	5.35E-11	2.61E-10
PHF16	0.118855	6.93E-03	1.21E-02
PHF17	-0.2041	3.02E-06	8.63E-06
PHF19	0.530534	9.97E-39	4.27E-37
PHF1	-0.34809	4.08E-16	3.33E-15
PHF20L1	0.102263	2.03E-02	3.24E-02
PHF20	0.097682	2.66E-02	4.16E-02
PHF21A	-0.16293	2.05E-04	4.56E-04
PHF21B	0.023702	5.92E-01	6.50E-01
PHF23	0.015554	7.25E-01	7.70E-01
PHF2	-0.35899	4.14E-17	3.74E-16
PHF3	-0.10219	2.04E-02	3.26E-02
PHF5A	0.3291	1.79E-14	1.24E-13
PHF6	0.374593	1.33E-18	1.37E-17
PHF7	-0.20936	1.64E-06	4.85E-06
PHF8	0.106958	1.52E-02	2.49E-02
PHGDH	0.26686	7.59E-10	3.30E-09
PHGR1	0.053867	2.22E-01	2.80E-01
PHIP	-0.1455	9.28E-04	1.87E-03
PHKA1	0.172256	8.53E-05	2.01E-04
PHKA2	-0.19833	5.76E-06	1.59E-05
PHKB	-0.47437	2.95E-30	7.75E-29
PHKG1	-0.17194	8.79E-05	2.06E-04

PHKG2	-0.1658	1.57E-04	3.55E-04
PHLDA1	0.071365	1.06E-01	1.45E-01
PHLDA2	0.162868	2.06E-04	4.59E-04
PHLDA3	-0.33593	4.74E-15	3.48E-14
PHLDB1	-0.21585	7.61E-07	2.34E-06
PHLDB2	0.178901	4.45E-05	1.09E-04
PHLDB3	0.021807	6.21E-01	6.78E-01
PHLPP1	0.074907	8.95E-02	1.25E-01
PHLPP2	0.13944	1.51E-03	2.95E-03
PHOSPHO1	-0.02637	5.50E-01	6.12E-01
PHOSPHO2	-0.06981	1.14E-01	1.54E-01
PHOX2A	0.245479	1.66E-08	6.18E-08
PHOX2B	-0.08948	4.24E-02	6.34E-02
PHPT1	-0.15607	3.78E-04	8.09E-04
PHRF1	-0.09497	3.12E-02	4.80E-02
PHTF1	0.085485	5.25E-02	7.71E-02
PHTF2	0.358371	4.72E-17	4.25E-16
PHYHD1	-0.41383	1.00E-22	1.46E-21
PHYHIPL	0.012579	7.76E-01	8.14E-01
PHYHIP	-0.09348	3.39E-02	5.18E-02
PHYH	0.039445	3.72E-01	4.38E-01
PI15	0.290868	1.69E-11	8.67E-11
PI16	-0.34274	1.22E-15	9.42E-15
PI3	0.200164	4.70E-06	1.32E-05
PI4K2A	0.001794	9.68E-01	9.75E-01
PI4K2B	-0.047	2.87E-01	3.49E-01
PI4KAP1	-0.10149	2.12E-02	3.38E-02
PI4KAP2	-0.05576	2.06E-01	2.62E-01
PI4KA	-0.09968	2.37E-02	3.74E-02
PI4KB	-0.16598	1.54E-04	3.50E-04
PIAS1	-0.02931	5.07E-01	5.71E-01
PIAS2	0.141562	1.28E-03	2.53E-03
PIAS3	-0.12277	5.27E-03	9.42E-03
PIAS4	0.122374	5.42E-03	9.68E-03
PIBF1	-0.18506	2.38E-05	6.05E-05
PICALM	0.195313	8.02E-06	2.18E-05
PICK1	-0.18764	1.82E-05	4.71E-05
PID1	-0.33501	5.68E-15	4.14E-14
PIF1	0.621898	1.90E-56	1.52E-54
PIGA	-0.22474	2.55E-07	8.34E-07
PIGB	-0.14993	6.41E-04	1.33E-03
PIGC	0.166121	1.52E-04	3.46E-04
PIGF	0.18143	3.45E-05	8.57E-05
PIGG	-0.00242	9.56E-01	9.65E-01

PIGH	-0.03962	3.70E-01	4.36E-01
PIGK	0.052786	2.32E-01	2.90E-01
PIGL	-0.1472	8.06E-04	1.64E-03
PIGM	-0.11829	7.20E-03	1.25E-02
PIGN	-0.0096	8.28E-01	8.58E-01
PIGO	0.073133	9.73E-02	1.34E-01
PIGP	-0.11931	6.71E-03	1.18E-02
PIGQ	-0.41946	2.30E-23	3.57E-22
PIGR	-0.44299	3.65E-26	7.11E-25
PIGS	0.094953	3.12E-02	4.80E-02
PIGT	-0.079	7.32E-02	1.04E-01
PIGU	0.244191	1.99E-08	7.31E-08
PIGV	-0.2312	1.12E-07	3.81E-07
PIGW	0.372838	1.98E-18	2.01E-17
PIGX	0.346157	6.07E-16	4.87E-15
PIGY	-0.11638	8.20E-03	1.41E-02
PIGZ	-0.13093	2.91E-03	5.43E-03
PIH1D1	0.145125	9.57E-04	1.93E-03
PIH1D2	-0.19314	1.01E-05	2.71E-05
PIK3AP1	0.109685	1.28E-02	2.13E-02
PIK3C2A	-0.09394	3.31E-02	5.06E-02
PIK3C2B	-0.18834	1.69E-05	4.39E-05
PIK3C2G	-0.04481	3.10E-01	3.74E-01
PIK3C3	0.071596	1.05E-01	1.43E-01
PIK3CA	0.200266	4.65E-06	1.30E-05
PIK3CB	0.050317	2.54E-01	3.15E-01
PIK3CD	-0.15537	4.02E-04	8.56E-04
PIK3CG	-0.10887	1.34E-02	2.23E-02
PIK3IP1	-0.49905	8.72E-34	2.81E-32
PIK3R1	-0.38004	3.85E-19	4.14E-18
PIK3R2	-0.00317	9.43E-01	9.55E-01
PIK3R3	-0.09091	3.92E-02	5.91E-02
PIK3R4	0.161329	2.37E-04	5.23E-04
PIK3R5	-0.16037	2.58E-04	5.66E-04
PIK3R6	-0.21696	6.66E-07	2.06E-06
PIKFYVE	-0.06664	1.31E-01	1.75E-01
PILRA	-8.71E-05	9.98E-01	9.99E-01
PILRB	-0.10864	1.36E-02	2.26E-02
PIM1	0.155566	3.95E-04	8.42E-04
PIM2	0.011247	7.99E-01	8.34E-01
PIM3	-0.10728	1.49E-02	2.45E-02
PIN1L	0.058099	1.88E-01	2.41E-01
PIN1	-0.01537	7.28E-01	7.73E-01
PIN4	0.078269	7.60E-02	1.08E-01

PINK1	-0.42253	1.02E-23	1.64E-22
PINX1	0.106779	1.53E-02	2.52E-02
PION	-0.38785	6.21E-20	7.21E-19
PIP4K2A	0.241514	2.86E-08	1.04E-07
PIP4K2B	-0.13889	1.58E-03	3.07E-03
PIP4K2C	0.222443	3.40E-07	1.09E-06
PIP5K1A	0.179965	4.00E-05	9.84E-05
PIP5K1B	-0.35079	2.33E-16	1.95E-15
PIP5K1C	-0.15328	4.82E-04	1.01E-03
PIP5K1P1	0.074476	9.13E-02	1.27E-01
PIP5KL1	-0.35395	1.21E-16	1.04E-15
PIPOX	0.107351	1.48E-02	2.44E-02
PIPSL	0.199331	5.16E-06	1.43E-05
PIP	-0.13762	1.75E-03	3.38E-03
PIRT	-0.02082	6.37E-01	6.92E-01
PIR	0.078387	7.55E-02	1.07E-01
PISD	0.005909	8.94E-01	9.14E-01
PITPNA	-0.35182	1.89E-16	1.59E-15
PITPNB	0.362814	1.81E-17	1.70E-16
PITPNC1	0.240109	3.46E-08	1.24E-07
PITPNM1	-0.12105	5.95E-03	1.05E-02
PITPNM2	-0.06562	1.37E-01	1.82E-01
PITPNM3	-0.45683	6.45E-28	1.43E-26
PITRM1	-0.04618	2.96E-01	3.59E-01
PITX1	0.306716	1.11E-12	6.49E-12
PITX2	0.072836	9.87E-02	1.36E-01
PITX3	0.304944	1.52E-12	8.78E-12
PIWIL1	-0.01603	7.17E-01	7.63E-01
PIWIL2	0.082227	6.22E-02	9.01E-02
PIWIL3	0.057269	1.94E-01	2.49E-01
PIWIL4	-0.16106	2.42E-04	5.35E-04
PJA1	0.124403	4.69E-03	8.47E-03
PJA2	-0.25999	2.11E-09	8.69E-09
PKD1L1	0.002499	9.55E-01	9.64E-01
PKD1L2	0.071486	1.05E-01	1.44E-01
PKD1L3	-0.1507	6.01E-04	1.25E-03
PKD1	-0.32549	3.57E-14	2.41E-13
PKD2L1	-0.03282	4.57E-01	5.24E-01
PKD2L2	-0.07556	8.67E-02	1.21E-01
PKD2	-0.20236	3.67E-06	1.04E-05
PKDCC	0.094941	3.12E-02	4.80E-02
PKDREJ	-0.19377	9.48E-06	2.55E-05
PKHD1L1	-0.21698	6.64E-07	2.06E-06
PKHD1	-0.1932	1.01E-05	2.70E-05

PKIA	0.060478	1.71E-01	2.22E-01
PKIB	0.313158	3.51E-13	2.16E-12
PKIG	0.015153	7.32E-01	7.76E-01
PKLR	0.052639	2.33E-01	2.92E-01
PKM2	0.380811	3.22E-19	3.50E-18
PKMYT1	0.753101	2.44E-95	3.58E-93
PKN1	0.056004	2.04E-01	2.60E-01
PKN2	0.249343	9.72E-09	3.71E-08
PKN3	0.083861	5.72E-02	8.34E-02
PKNOX1	0.031654	4.74E-01	5.39E-01
PKNOX2	-0.44056	7.26E-26	1.38E-24
PKP1	-0.12659	4.01E-03	7.31E-03
PKP2	0.348803	3.53E-16	2.89E-15
PKP3	0.093654	3.36E-02	5.14E-02
PKP4	0.031858	4.71E-01	5.36E-01
PL-5283	0.387567	6.64E-20	7.67E-19
PLA1A	-0.15024	6.25E-04	1.29E-03
PLA2G10	-0.50789	4.02E-35	1.38E-33
PLA2G12A	-0.18356	2.78E-05	6.99E-05
PLA2G12B	-0.26626	8.32E-10	3.59E-09
PLA2G15	-0.11517	8.89E-03	1.52E-02
PLA2G16	-0.10548	1.66E-02	2.71E-02
PLA2G1B	-0.53058	9.78E-39	4.20E-37
PLA2G2A	-0.18344	2.81E-05	7.06E-05
PLA2G2C	-0.10685	1.53E-02	2.51E-02
PLA2G2D	0.009785	8.25E-01	8.55E-01
PLA2G2F	0.064389	1.45E-01	1.91E-01
PLA2G3	-0.22429	2.70E-07	8.79E-07
PLA2G4A	-0.0149	7.36E-01	7.80E-01
PLA2G4C	-0.15198	5.39E-04	1.13E-03
PLA2G4D	0.107346	1.48E-02	2.44E-02
PLA2G4E	-0.10739	1.48E-02	2.43E-02
PLA2G4F	-0.46353	8.54E-29	2.02E-27
PLA2G5	-0.18332	2.84E-05	7.14E-05
PLA2G6	-0.27456	2.33E-10	1.07E-09
PLA2G7	0.120763	6.07E-03	1.07E-02
PLA2R1	-0.01652	7.08E-01	7.56E-01
PLAA	0.23488	6.93E-08	2.40E-07
PLAC1L	0.072292	1.01E-01	1.39E-01
PLAC1	0.312174	4.20E-13	2.56E-12
PLAC2	0.016197	7.14E-01	7.61E-01
PLAC4	-0.09572	2.99E-02	4.61E-02
PLAC8L1	0.011044	8.03E-01	8.37E-01
PLAC8	-0.15862	3.02E-04	6.56E-04

PLAC9	-0.4883	3.26E-32	9.64E-31
PLAG1	-0.09359	3.37E-02	5.15E-02
PLAGL1	-0.05593	2.05E-01	2.61E-01
PLAGL2	0.044813	3.10E-01	3.74E-01
PLAT	-0.03549	4.22E-01	4.88E-01
PLAUR	0.236646	5.49E-08	1.92E-07
PLAU	0.357054	6.26E-17	5.53E-16
PLB1	-0.08286	6.02E-02	8.74E-02
PLBD1	0.167193	1.38E-04	3.15E-04
PLBD2	0.155325	4.03E-04	8.59E-04
PLCB1	0.11456	9.27E-03	1.58E-02
PLCB2	-0.32617	3.14E-14	2.13E-13
PLCB3	0.064982	1.41E-01	1.87E-01
PLCB4	-0.1828	3.00E-05	7.51E-05
PLCD1	-0.43999	8.54E-26	1.61E-24
PLCD3	0.17144	9.23E-05	2.16E-04
PLCD4	-0.03784	3.91E-01	4.58E-01
PLCE1	-0.27675	1.66E-10	7.72E-10
PLCG1	-0.07924	7.24E-02	1.03E-01
PLCG2	-0.22064	4.25E-07	1.35E-06
PLCH1	-0.36424	1.33E-17	1.25E-16
PLCH2	-0.28755	2.92E-11	1.46E-10
PLCL1	-0.32944	1.68E-14	1.16E-13
PLCL2	-0.11388	9.70E-03	1.65E-02
PLCXD1	-0.05441	2.18E-01	2.75E-01
PLCXD2	0.285298	4.22E-11	2.07E-10
PLCXD3	-0.32838	2.06E-14	1.42E-13
PLCZ1	0.060834	1.68E-01	2.19E-01
PLD1	0.261822	1.61E-09	6.72E-09
PLD2	-0.30484	1.55E-12	8.94E-12
PLD3	-0.35944	3.76E-17	3.42E-16
PLD4	-0.38885	4.91E-20	5.74E-19
PLD5	-0.15357	4.70E-04	9.90E-04
PLD6	-0.21719	6.48E-07	2.01E-06
PLDN	0.057543	1.92E-01	2.46E-01
PLEC	-0.00559	8.99E-01	9.19E-01
PLEK2	0.352091	1.78E-16	1.50E-15
PLEKHA1	-0.01793	6.85E-01	7.35E-01
PLEKHA2	-0.03592	4.16E-01	4.82E-01
PLEKHA3	-0.01104	8.03E-01	8.37E-01
PLEKHA4	-0.20214	3.76E-06	1.07E-05
PLEKHA5	0.096009	2.94E-02	4.54E-02
PLEKHA6	-0.08791	4.62E-02	6.86E-02
PLEKHA7	-0.22814	1.66E-07	5.52E-07

PLEKHA8	0.160114	2.64E-04	5.79E-04
PLEKHA9	0.249403	9.64E-09	3.69E-08
PLEKHB1	-0.27463	2.30E-10	1.06E-09
PLEKHB2	0.091253	3.84E-02	5.80E-02
PLEKHF1	-0.05386	2.22E-01	2.80E-01
PLEKHF2	-0.02112	6.33E-01	6.88E-01
PLEKHG1	-0.166	1.54E-04	3.49E-04
PLEKHG2	0.113974	9.64E-03	1.64E-02
PLEKHG3	0.090166	4.08E-02	6.13E-02
PLEKHG4B	-0.18064	3.73E-05	9.23E-05
PLEKHG4	0.003645	9.34E-01	9.48E-01
PLEKHG5	0.048702	2.70E-01	3.32E-01
PLEKHG6	0.218123	5.78E-07	1.81E-06
PLEKHG7	-0.24316	2.29E-08	8.36E-08
PLEKHH1	0.192961	1.03E-05	2.76E-05
PLEKHH2	-0.3947	1.20E-20	1.49E-19
PLEKHH3	-0.16546	1.62E-04	3.67E-04
PLEKHJ1	0.095164	3.08E-02	4.75E-02
PLEKHM1P	-0.34179	1.47E-15	1.13E-14
PLEKHM1	-0.30943	6.86E-13	4.10E-12
PLEKHM2	-0.09419	3.26E-02	5.00E-02
PLEKHM3	-0.34352	1.04E-15	8.11E-15
PLEKHN1	-0.08388	5.71E-02	8.33E-02
PLEKHO1	0.162248	2.18E-04	4.84E-04
PLEKHO2	-0.04853	2.72E-01	3.34E-01
PLEK	-0.01315	7.66E-01	8.05E-01
PLGLA	0.015673	7.23E-01	7.69E-01
PLGLB2	-0.18657	2.03E-05	5.22E-05
PLG	-0.14756	7.82E-04	1.60E-03
PLIN1	-0.24165	2.81E-08	1.02E-07
PLIN2	0.387028	7.54E-20	8.67E-19
PLIN3	0.301765	2.65E-12	1.49E-11
PLIN4	-0.16162	2.30E-04	5.10E-04
PLIN5	-0.47624	1.63E-30	4.33E-29
PLK1S1	-0.30145	2.80E-12	1.57E-11
PLK1	0.85899	2.91E-151	1.62E-148
PLK2	-0.04702	2.87E-01	3.49E-01
PLK3	-0.15902	2.91E-04	6.34E-04
PLK4	0.838613	1.92E-137	6.61E-135
PLK5P	0.020811	6.38E-01	6.92E-01
PLLP	-0.50274	2.44E-34	8.11E-33
PLN	-0.09264	3.56E-02	5.41E-02
PLOD1	0.278244	1.31E-10	6.15E-10
PLOD2	0.569896	1.10E-45	6.57E-44

PLOD3	0.295994	7.13E-12	3.82E-11
PLP1	-0.29938	4.00E-12	2.21E-11
PLP2	0.185525	2.27E-05	5.78E-05
PLRG1	0.272138	3.39E-10	1.53E-09
PLS1	-0.01926	6.63E-01	7.15E-01
PLS3	-0.10035	2.28E-02	3.60E-02
PLSCR1	0.379719	4.15E-19	4.45E-18
PLSCR2	0.161387	2.35E-04	5.20E-04
PLSCR3	-0.08595	5.12E-02	7.54E-02
PLSCR4	-0.24203	2.67E-08	9.68E-08
PLSCR5	0.15773	3.27E-04	7.06E-04
PLTP	0.024477	5.79E-01	6.39E-01
PLUNC	0.002157	9.61E-01	9.69E-01
PLVAP	0.023132	6.00E-01	6.58E-01
PLXDC1	-0.21283	1.09E-06	3.29E-06
PLXDC2	-0.07371	9.47E-02	1.31E-01
PLXNA1	0.157371	3.37E-04	7.27E-04
PLXNA2	-0.53405	2.59E-39	1.15E-37
PLXNA3	0.076069	8.46E-02	1.19E-01
PLXNA4	-0.1221	5.53E-03	9.85E-03
PLXNB1	-0.37498	1.22E-18	1.26E-17
PLXNB2	-0.22818	1.65E-07	5.50E-07
PLXNB3	-0.1168	7.97E-03	1.38E-02
PLXNC1	-0.04233	3.38E-01	4.03E-01
PLXND1	-0.20624	2.36E-06	6.83E-06
PM20D1	-0.22047	4.34E-07	1.38E-06
PM20D2	0.266365	8.18E-10	3.54E-09
PMAIP1	0.495497	2.92E-33	9.13E-32
PMCHL1	0.054222	2.19E-01	2.77E-01
PMCHL2	0.017752	6.88E-01	7.37E-01
PMCH	0.039707	3.69E-01	4.35E-01
PMEPA1	-0.03411	4.40E-01	5.06E-01
PMF1	-0.18798	1.75E-05	4.55E-05
PMFBP1	-0.01055	8.11E-01	8.44E-01
PML	0.071725	1.04E-01	1.42E-01
PMM1	-0.4769	1.32E-30	3.54E-29
PMM2	0.107675	1.45E-02	2.39E-02
PMP22	-0.16066	2.51E-04	5.53E-04
PMP2	-0.22739	1.83E-07	6.06E-07
PMPCA	0.011601	7.93E-01	8.29E-01
PMPCB	0.096569	2.84E-02	4.41E-02
PMS1	0.230942	1.16E-07	3.93E-07
PMS2CL	0.080334	6.85E-02	9.84E-02
PMS2L11	-0.09318	3.45E-02	5.26E-02

PMS2L1	0.327566	2.40E-14	1.65E-13
PMS2L2	0.014956	7.35E-01	7.79E-01
PMS2L3	-0.02839	5.20E-01	5.84E-01
PMS2L4	-0.04582	2.99E-01	3.63E-01
PMS2L5	0.226596	2.02E-07	6.67E-07
PMS2	0.110699	1.19E-02	2.00E-02
PMVK	-0.25861	2.58E-09	1.05E-08
PNCK	0.199404	5.11E-06	1.42E-05
PNKD	-0.2465	1.44E-08	5.39E-08
PNKP	0.118032	7.33E-03	1.27E-02
PNLDC1	-0.05906	1.81E-01	2.33E-01
PNLIPRP1	-0.05879	1.83E-01	2.36E-01
PNLIPRP2	-0.03796	3.90E-01	4.57E-01
PNLIPRP3	-0.04529	3.05E-01	3.69E-01
PNLIP	-0.00377	9.32E-01	9.46E-01
PNMA1	0.324007	4.72E-14	3.16E-13
PNMA2	-0.4617	1.49E-28	3.45E-27
PNMA3	-0.03568	4.19E-01	4.85E-01
PNMA5	0.06098	1.67E-01	2.18E-01
PNMA6A	-0.10035	2.27E-02	3.60E-02
PNMAL1	-0.12742	3.77E-03	6.91E-03
PNMAL2	-0.31187	4.43E-13	2.70E-12
PNMT	-0.22367	2.92E-07	9.47E-07
PNN	0.066108	1.34E-01	1.79E-01
PNO1	0.524219	1.08E-37	4.31E-36
PNOC	-0.07186	1.03E-01	1.42E-01
PNPLA1	0.24751	1.26E-08	4.73E-08
PNPLA2	-0.40227	1.88E-21	2.48E-20
PNPLA3	0.155715	3.90E-04	8.33E-04
PNPLA4	-0.18886	1.60E-05	4.17E-05
PNPLA5	0.210128	1.50E-06	4.46E-06
PNPLA6	-0.04696	2.88E-01	3.50E-01
PNPLA7	-0.41673	4.72E-23	7.10E-22
PNPLA8	0.236418	5.66E-08	1.98E-07
PNPO	0.170067	1.05E-04	2.45E-04
PNPT1	0.52477	8.76E-38	3.52E-36
PNP	0.457224	5.73E-28	1.28E-26
PNRC1	-0.17579	6.05E-05	1.46E-04
PNRC2	-0.14503	9.64E-04	1.94E-03
POC1A	0.762411	4.90E-99	7.83E-97
POC1B	0.366356	8.37E-18	8.01E-17
POC5	0.083198	5.92E-02	8.60E-02
PODNL1	-0.10175	2.09E-02	3.33E-02
PODN	-0.45288	2.07E-27	4.44E-26

PODXL2	-0.0655	1.38E-01	1.83E-01
PODXL	-0.16793	1.29E-04	2.95E-04
POF1B	0.000555	9.90E-01	9.92E-01
POFUT1	0.367275	6.84E-18	6.58E-17
POFUT2	-0.04709	2.86E-01	3.48E-01
POGK	-0.05187	2.40E-01	3.00E-01
POGZ	-0.12005	6.38E-03	1.12E-02
POLA1	0.338568	2.81E-15	2.09E-14
POLA2	0.671578	8.23E-69	8.39E-67
POLB	0.150372	6.18E-04	1.28E-03
POLD1	0.328658	1.95E-14	1.35E-13
POLD2	0.390892	3.01E-20	3.60E-19
POLD3	0.400538	2.89E-21	3.76E-20
POLD4	0.009762	8.25E-01	8.56E-01
POLDIP2	0.27338	2.80E-10	1.28E-09
POLDIP3	-0.32121	7.98E-14	5.23E-13
POLE2	0.724199	7.65E-85	9.68E-83
POLE3	0.387398	6.91E-20	7.98E-19
POLE4	0.28817	2.63E-11	1.33E-10
POLE	0.447858	9.01E-27	1.82E-25
POLG2	0.08637	5.01E-02	7.39E-02
POLG	-0.10046	2.26E-02	3.58E-02
POLH	-0.23365	8.14E-08	2.81E-07
POLI	-0.29149	1.52E-11	7.86E-11
POLK	-0.15259	5.11E-04	1.07E-03
POLL	-0.24768	1.23E-08	4.62E-08
POLM	-0.207	2.16E-06	6.29E-06
POLN	-0.07322	9.70E-02	1.34E-01
POLQ	0.777107	3.12E-105	5.47E-103
POLR1A	0.312153	4.21E-13	2.57E-12
POLR1B	0.281131	8.26E-11	3.96E-10
POLR1C	0.162102	2.21E-04	4.90E-04
POLR1D	-0.05291	2.31E-01	2.89E-01
POLR1E	0.129437	3.25E-03	6.03E-03
POLR2A	-0.06322	1.52E-01	2.00E-01
POLR2B	0.268188	6.21E-10	2.72E-09
POLR2C	-0.209	1.72E-06	5.06E-06
POLR2D	0.477265	1.18E-30	3.16E-29
POLR2E	0.034237	4.38E-01	5.05E-01
POLR2F	0.120502	6.18E-03	1.09E-02
POLR2G	0.258209	2.74E-09	1.11E-08
POLR2H	0.341421	1.59E-15	1.21E-14
POLR2I	0.20213	3.77E-06	1.07E-05
POLR2J2	0.05415	2.20E-01	2.77E-01

POLR2J3	-0.00562	8.99E-01	9.18E-01
POLR2J4	-0.02094	6.35E-01	6.90E-01
POLR2J	0.246507	1.44E-08	5.39E-08
POLR2K	0.223651	2.92E-07	9.49E-07
POLR2L	-0.18408	2.63E-05	6.64E-05
POLR3A	0.252128	6.56E-09	2.56E-08
POLR3B	0.103963	1.83E-02	2.95E-02
POLR3C	0.186598	2.03E-05	5.21E-05
POLR3D	0.253495	5.40E-09	2.13E-08
POLR3E	-0.09856	2.53E-02	3.97E-02
POLR3F	0.21741	6.31E-07	1.96E-06
POLR3GL	-0.34146	1.57E-15	1.20E-14
POLR3G	0.445885	1.59E-26	3.16E-25
POLR3H	-0.42526	4.93E-24	8.09E-23
POLR3K	0.080269	6.87E-02	9.86E-02
POLRMT	0.073783	9.44E-02	1.31E-01
POM121C	0.089378	4.26E-02	6.38E-02
POM121L10P	-0.33079	1.29E-14	9.07E-14
POM121L1P	-0.19097	1.28E-05	3.38E-05
POM121L2	0.054873	2.14E-01	2.70E-01
POM121L4P	-0.20215	3.76E-06	1.07E-05
POM121L8P	-0.22073	4.20E-07	1.34E-06
POM121L9P	-0.29616	6.94E-12	3.72E-11
POM121	0.084081	5.65E-02	8.25E-02
POMC	0.09587	2.96E-02	4.57E-02
POMGNT1	-0.12064	6.12E-03	1.08E-02
POMP	0.361688	2.32E-17	2.15E-16
POMT1	-0.3152	2.42E-13	1.51E-12
POMT2	-0.01686	7.03E-01	7.51E-01
POMZP3	-0.1176	7.55E-03	1.31E-02
PON1	-0.16052	2.55E-04	5.59E-04
PON2	0.013601	7.58E-01	7.99E-01
PON3	-0.17541	6.28E-05	1.51E-04
POP1	0.554384	7.84E-43	4.14E-41
POP4	0.25308	5.73E-09	2.25E-08
POP5	0.12948	3.24E-03	6.01E-03
POP7	0.435978	2.63E-25	4.76E-24
POPDC2	-0.1972	6.52E-06	1.79E-05
POPDC3	0.396974	6.93E-21	8.72E-20
PORCN	0.065687	1.37E-01	1.82E-01
POR	-0.04534	3.04E-01	3.68E-01
POSTN	0.277392	1.50E-10	7.00E-10
POT1	0.371247	2.83E-18	2.83E-17
POTEA	0.057123	1.96E-01	2.50E-01

POTEB	0.021842	6.21E-01	6.77E-01
POTEC	0.052397	2.35E-01	2.94E-01
POTED	0.064754	1.42E-01	1.89E-01
POTEE	0.118004	7.34E-03	1.28E-02
POTEF	0.177353	5.18E-05	1.26E-04
POTEG	0.111362	1.14E-02	1.92E-02
POTEH	0.094429	3.22E-02	4.93E-02
POU1F1	-0.12412	4.79E-03	8.63E-03
POU2AF1	-0.08501	5.39E-02	7.89E-02
POU2F1	0.009463	8.30E-01	8.60E-01
POU2F2	-0.01318	7.65E-01	8.05E-01
POU2F3	-0.3502	2.64E-16	2.19E-15
POU3F1	-0.1716	9.08E-05	2.13E-04
POU3F2	0.048552	2.71E-01	3.33E-01
POU3F3	0.039914	3.66E-01	4.32E-01
POU3F4	0.064307	1.45E-01	1.92E-01
POU4F1	0.2211	4.02E-07	1.28E-06
POU4F2	0.142539	1.18E-03	2.35E-03
POU4F3	0.014023	7.51E-01	7.93E-01
POU5F1B	-0.25572	3.93E-09	1.57E-08
POU5F1	-0.22087	4.13E-07	1.31E-06
POU5F2	-0.05816	1.88E-01	2.41E-01
POU6F1	-0.35767	5.49E-17	4.89E-16
POU6F2	0.09641	2.87E-02	4.45E-02
PP14571	0.040754	3.56E-01	4.22E-01
PPA1	0.071269	1.06E-01	1.45E-01
PPA2	-0.06334	1.51E-01	1.99E-01
PPAN-P2RY11	-0.09235	3.62E-02	5.49E-02
PPAN	0.236534	5.57E-08	1.95E-07
PPAP2A	-0.17487	6.62E-05	1.58E-04
PPAP2B	-0.32541	3.62E-14	2.44E-13
PPAP2C	0.007986	8.57E-01	8.83E-01
PPAPDC1A	0.337982	3.16E-15	2.35E-14
PPAPDC1B	-0.02989	4.99E-01	5.63E-01
PPAPDC2	0.043039	3.30E-01	3.95E-01
PPAPDC3	-0.07174	1.04E-01	1.42E-01
PPARA	-0.17734	5.19E-05	1.26E-04
PPARD	0.065869	1.35E-01	1.81E-01
PPARGC1A	-0.10444	1.77E-02	2.87E-02
PPARGC1B	0.030775	4.86E-01	5.51E-01
PPARG	0.111324	1.15E-02	1.93E-02
PPAT	0.619926	5.30E-56	4.14E-54
PPBPL2	0.009282	8.34E-01	8.63E-01
PPBP	-0.06637	1.33E-01	1.77E-01

PPCDC	0.077969	7.71E-02	1.09E-01
PPCS	0.012939	7.70E-01	8.08E-01
PPDPF	-0.27593	1.88E-10	8.73E-10
PPEF1	0.148319	7.34E-04	1.51E-03
PPEF2	-0.10115	2.17E-02	3.45E-02
PPFIA1	0.155665	3.92E-04	8.36E-04
PPFIA2	-0.17665	5.56E-05	1.34E-04
PPFIA3	0.14716	8.09E-04	1.65E-03
PPFIA4	0.139593	1.49E-03	2.92E-03
PPFIBP1	0.196373	7.14E-06	1.95E-05
PPFIBP2	-0.44716	1.10E-26	2.22E-25
PPHLN1	0.357142	6.14E-17	5.44E-16
PPIAL4C	0.352117	1.77E-16	1.50E-15
PPIAL4D	0.126308	4.09E-03	7.45E-03
PPIAL4E	0.026691	5.46E-01	6.08E-01
PPIAL4G	0.322675	6.07E-14	4.02E-13
PPIA	0.368124	5.67E-18	5.49E-17
PPIB	0.024472	5.80E-01	6.39E-01
PPIC	0.065141	1.40E-01	1.86E-01
PPID	0.449616	5.40E-27	1.11E-25
PPIEL	-0.2887	2.42E-11	1.22E-10
PPIE	0.020251	6.47E-01	7.01E-01
PPIF	0.483141	1.77E-31	5.00E-30
PPIG	0.196783	6.83E-06	1.87E-05
PPIH	0.371923	2.44E-18	2.45E-17
PPIL1	0.441394	5.74E-26	1.10E-24
PPIL2	-0.14425	1.03E-03	2.06E-03
PPIL3	0.12559	4.31E-03	7.83E-03
PPIL4	0.141269	1.31E-03	2.58E-03
PPIL5	0.591574	6.20E-50	4.17E-48
PPIL6	-0.40817	4.27E-22	5.93E-21
PPIP5K1	-0.1591	2.89E-04	6.30E-04
PPIP5K2	0.07743	7.92E-02	1.12E-01
PPL	-0.2706	4.30E-10	1.92E-09
PPM1A	-0.14507	9.61E-04	1.94E-03
PPM1B	0.058565	1.85E-01	2.38E-01
PPM1D	-0.02851	5.19E-01	5.83E-01
PPM1E	0.120217	6.31E-03	1.11E-02
PPM1F	-0.09773	2.66E-02	4.15E-02
PPM1G	0.646692	2.50E-62	2.29E-60
PPM1H	0.078496	7.51E-02	1.07E-01
PPM1J	-0.08726	4.78E-02	7.07E-02
PPM1K	-0.09409	3.28E-02	5.02E-02
PPM1L	-0.03097	4.83E-01	5.48E-01

PPM1M	-0.34068	1.84E-15	1.40E-14
PPM1N	0.048927	2.68E-01	3.29E-01
PPME1	0.301574	2.74E-12	1.54E-11
PPOX	-0.34629	5.91E-16	4.75E-15
PPP1CA	0.222242	3.49E-07	1.12E-06
PPP1CB	0.311544	4.70E-13	2.86E-12
PPP1CC	0.48343	1.61E-31	4.57E-30
PPP1R10	-0.11628	8.26E-03	1.42E-02
PPP1R11	-0.05351	2.25E-01	2.83E-01
PPP1R12A	0.34839	3.84E-16	3.13E-15
PPP1R12B	-0.40804	4.41E-22	6.12E-21
PPP1R12C	-0.14539	9.36E-04	1.89E-03
PPP1R13B	-0.50815	3.66E-35	1.27E-33
PPP1R13L	-0.08371	5.76E-02	8.40E-02
PPP1R14A	-0.16239	2.15E-04	4.78E-04
PPP1R14B	0.320892	8.47E-14	5.53E-13
PPP1R14C	-0.1251	4.47E-03	8.08E-03
PPP1R14D	0.087881	4.62E-02	6.87E-02
PPP1R15A	-0.20112	4.22E-06	1.19E-05
PPP1R15B	0.140037	1.44E-03	2.83E-03
PPP1R16A	-0.12939	3.27E-03	6.04E-03
PPP1R16B	-0.13228	2.63E-03	4.94E-03
PPP1R1A	-0.11653	8.12E-03	1.40E-02
PPP1R1B	-0.33931	2.42E-15	1.82E-14
PPP1R1C	-0.02704	5.40E-01	6.03E-01
PPP1R2P1	0.094308	3.24E-02	4.96E-02
PPP1R2P3	0.226678	2.00E-07	6.61E-07
PPP1R2P9	0.038676	3.81E-01	4.48E-01
PPP1R2	0.185809	2.20E-05	5.63E-05
PPP1R3A	0.106663	1.55E-02	2.53E-02
PPP1R3B	-0.12358	4.98E-03	8.94E-03
PPP1R3C	-0.22126	3.94E-07	1.26E-06
PPP1R3D	-0.15902	2.91E-04	6.34E-04
PPP1R3E	-0.21236	1.16E-06	3.47E-06
PPP1R3F	-0.08982	4.16E-02	6.24E-02
PPP1R3G	0.355866	8.05E-17	7.03E-16
PPP1R7	-0.10339	1.89E-02	3.05E-02
PPP1R8	0.265655	9.10E-10	3.91E-09
PPP1R9A	-0.24151	2.86E-08	1.04E-07
PPP1R9B	-0.14598	8.92E-04	1.80E-03
PPP2CA	0.278586	1.24E-10	5.83E-10
PPP2CB	-0.18741	1.86E-05	4.82E-05
PPP2R1A	0.053586	2.25E-01	2.83E-01
PPP2R1B	0.319078	1.19E-13	7.63E-13

PPP2R2A	0.050931	2.49E-01	3.09E-01
PPP2R2B	-0.10043	2.26E-02	3.59E-02
PPP2R2C	0.159112	2.89E-04	6.29E-04
PPP2R2D	0.00311	9.44E-01	9.56E-01
PPP2R3A	-0.10359	1.87E-02	3.01E-02
PPP2R3B	0.134654	2.20E-03	4.18E-03
PPP2R3C	0.217237	6.44E-07	2.00E-06
PPP2R4	-0.01908	6.66E-01	7.17E-01
PPP2R5A	-0.42673	3.31E-24	5.52E-23
PPP2R5B	0.00712	8.72E-01	8.96E-01
PPP2R5C	-0.10878	1.35E-02	2.24E-02
PPP2R5D	0.239293	3.86E-08	1.37E-07
PPP2R5E	0.299017	4.26E-12	2.34E-11
PPP3CA	-0.24593	1.56E-08	5.82E-08
PPP3CB	-0.08011	6.93E-02	9.93E-02
PPP3CC	-0.25397	5.05E-09	1.99E-08
PPP3R1	0.325035	3.89E-14	2.62E-13
PPP3R2	-0.08854	4.46E-02	6.65E-02
PPP4C	0.319195	1.16E-13	7.47E-13
PPP4R1L	-0.13516	2.11E-03	4.02E-03
PPP4R1	-0.07321	9.70E-02	1.34E-01
PPP4R2	0.338982	2.59E-15	1.94E-14
PPP4R4	-0.15582	3.86E-04	8.25E-04
PPP5C	0.150936	5.89E-04	1.23E-03
PPP6C	-0.08633	5.02E-02	7.40E-02
PPPDE1	-0.10802	1.42E-02	2.34E-02
PPPDE2	0.024298	5.82E-01	6.41E-01
PPRC1	0.017445	6.93E-01	7.42E-01
PPT1	0.036697	4.06E-01	4.72E-01
PPT2	0.304427	1.66E-12	9.57E-12
PPTC7	0.187407	1.86E-05	4.82E-05
PPWD1	-0.0904	4.03E-02	6.06E-02
PPY2	0.238861	4.09E-08	1.45E-07
PPYR1	-0.07858	7.48E-02	1.06E-01
PPY	0.057407	1.93E-01	2.48E-01
PQBP1	0.114827	9.10E-03	1.56E-02
PQLC1	-0.15914	2.88E-04	6.28E-04
PQLC2	0.051346	2.45E-01	3.05E-01
PQLC3	-0.10494	1.72E-02	2.79E-02
PRAC	0.09692	2.79E-02	4.33E-02
PRAF2	-0.04961	2.61E-01	3.22E-01
PRAM1	-0.24311	2.30E-08	8.41E-08
PRAMEF10	0.01469	7.39E-01	7.83E-01
PRAMEF11	0.101693	2.10E-02	3.34E-02

PRAMEF12	0.060753	1.69E-01	2.19E-01
PRAMEF13	-0.05853	1.85E-01	2.38E-01
PRAMEF14	-0.05583	2.06E-01	2.61E-01
PRAMEF16	-0.12719	3.84E-03	7.02E-03
PRAMEF18	-0.08242	6.16E-02	8.92E-02
PRAMEF1	-0.03018	4.94E-01	5.59E-01
PRAMEF20	0.068815	1.19E-01	1.61E-01
PRAMEF22	0.022868	6.05E-01	6.62E-01
PRAMEF2	-0.06283	1.55E-01	2.03E-01
PRAMEF4	0.092833	3.52E-02	5.36E-02
PRAMEF5	0.110973	1.17E-02	1.97E-02
PRAMEF6	0.098485	2.54E-02	3.98E-02
PRAMEF8	-0.1233	5.08E-03	9.11E-03
PRAMEF9	0.049306	2.64E-01	3.25E-01
PRAME	0.313103	3.55E-13	2.18E-12
PRAP1	0.197365	6.41E-06	1.76E-05
PRB1	-0.09172	3.74E-02	5.67E-02
PRB2	-0.12013	6.34E-03	1.12E-02
PRB3	-0.0742	9.26E-02	1.29E-01
PRB4	-0.11257	1.06E-02	1.79E-02
PRC1	0.859189	2.09E-151	1.19E-148
PRCC	0.218595	5.46E-07	1.71E-06
PRCD	-0.35267	1.58E-16	1.34E-15
PRCP	0.135537	2.05E-03	3.92E-03
PRDM10	0.018466	6.76E-01	7.27E-01
PRDM11	-0.18958	1.48E-05	3.88E-05
PRDM12	0.167135	1.39E-04	3.16E-04
PRDM13	0.299843	3.69E-12	2.05E-11
PRDM14	0.056803	1.98E-01	2.53E-01
PRDM15	-0.0692	1.17E-01	1.58E-01
PRDM16	-0.52318	1.58E-37	6.25E-36
PRDM1	0.06761	1.25E-01	1.69E-01
PRDM2	-0.35385	1.23E-16	1.06E-15
PRDM4	0.249336	9.73E-09	3.72E-08
PRDM5	-0.12641	4.06E-03	7.40E-03
PRDM6	-0.39964	3.60E-21	4.64E-20
PRDM7	0.058276	1.87E-01	2.40E-01
PRDM8	0.132243	2.64E-03	4.95E-03
PRDM9	0.256464	3.53E-09	1.41E-08
PRDX1	0.170183	1.04E-04	2.42E-04
PRDX2	0.075762	8.59E-02	1.20E-01
PRDX3	0.217918	5.93E-07	1.85E-06
PRDX4	0.221022	4.05E-07	1.29E-06
PRDX5	-0.20619	2.38E-06	6.87E-06

PRDX6	0.015091	7.33E-01	7.77E-01
PRDXDD1P	-0.0534	2.26E-01	2.84E-01
PREB	0.454748	1.20E-27	2.60E-26
PRELID1	0.258258	2.72E-09	1.11E-08
PRELID2	0.123198	5.12E-03	9.17E-03
PRELP	-0.3934	1.65E-20	2.02E-19
PREPL	-0.23112	1.13E-07	3.85E-07
PREP	0.103991	1.82E-02	2.95E-02
PREX1	-0.10665	1.55E-02	2.53E-02
PREX2	-0.34296	1.16E-15	9.03E-15
PRF1	0.0336	4.47E-01	5.13E-01
PRG1	0.055485	2.09E-01	2.65E-01
PRG2	-0.12864	3.45E-03	6.36E-03
PRG3	0.010917	8.05E-01	8.39E-01
PRG4	-0.46498	5.48E-29	1.31E-27
PRH1	0.0069	8.76E-01	8.99E-01
PRH2	-0.18401	2.65E-05	6.69E-05
PRHOXNB	0.059344	1.79E-01	2.31E-01
PRIC285	0.022238	6.15E-01	6.71E-01
PRICKLE1	-0.29373	1.04E-11	5.50E-11
PRICKLE2	-0.43287	6.21E-25	1.09E-23
PRICKLE3	0.152003	5.38E-04	1.12E-03
PRICKLE4	-0.55442	7.74E-43	4.10E-41
PRIM1	0.672239	5.43E-69	5.59E-67
PRIM2	0.370161	3.61E-18	3.57E-17
PRIMA1	-0.1507	6.01E-04	1.25E-03
PRINS	-0.2324	9.58E-08	3.28E-07
PRKAA1	0.012083	7.84E-01	8.21E-01
PRKAA2	0.181281	3.50E-05	8.69E-05
PRKAB1	-0.35692	6.43E-17	5.68E-16
PRKAB2	0.088755	4.41E-02	6.58E-02
PRKACA	-0.10169	2.10E-02	3.34E-02
PRKACB	0.038774	3.80E-01	4.46E-01
PRKACG	-0.05554	2.08E-01	2.64E-01
PRKAG1	0.140002	1.45E-03	2.83E-03
PRKAG2	-0.10048	2.26E-02	3.58E-02
PRKAG3	-0.03594	4.16E-01	4.82E-01
PRKAR1A	-0.29771	5.33E-12	2.89E-11
PRKAR1B	0.074001	9.34E-02	1.30E-01
PRKAR2A	-0.02079	6.38E-01	6.93E-01
PRKAR2B	-0.19455	8.71E-06	2.35E-05
PRKCA	-0.04597	2.98E-01	3.61E-01
PRKCB	-0.23459	7.20E-08	2.49E-07
PRKCDBP	-0.02817	5.24E-01	5.87E-01

PRKCD	-0.40295	1.59E-21	2.10E-20
PRKCE	-0.41049	2.36E-22	3.34E-21
PRKCG	0.131396	2.81E-03	5.26E-03
PRKCH	-0.15985	2.70E-04	5.92E-04
PRKCI	0.007671	8.62E-01	8.87E-01
PRKCQ	-0.18559	2.25E-05	5.75E-05
PRKCSH	0.025559	5.63E-01	6.23E-01
PRKCZ	-0.38617	9.23E-20	1.05E-18
PRKD1	-0.08217	6.24E-02	9.03E-02
PRKD2	-0.06701	1.29E-01	1.73E-01
PRKD3	0.242443	2.52E-08	9.18E-08
PRKDC	0.419351	2.37E-23	3.67E-22
PRKG1	-0.19334	9.93E-06	2.66E-05
PRKG2	0.094851	3.14E-02	4.82E-02
PRKRA	0.149212	6.81E-04	1.41E-03
PRKRIP1	0.008398	8.49E-01	8.77E-01
PRKRIR	0.202492	3.62E-06	1.03E-05
PRKX	0.041608	3.46E-01	4.12E-01
PRKY	-0.02734	5.36E-01	5.99E-01
PRLHR	0.090319	4.05E-02	6.09E-02
PRLH	0.025789	5.59E-01	6.20E-01
PRLR	-0.01166	7.92E-01	8.28E-01
PRL	0.16609	1.53E-04	3.47E-04
PRM1	-0.03586	4.17E-01	4.83E-01
PRM2	-0.03267	4.59E-01	5.26E-01
PRM3	0.03786	3.91E-01	4.58E-01
PRMT10	0.003863	9.30E-01	9.45E-01
PRMT1	0.396248	8.27E-21	1.04E-19
PRMT2	-0.33533	5.33E-15	3.89E-14
PRMT3	0.324952	3.95E-14	2.66E-13
PRMT5	0.378955	4.94E-19	5.27E-18
PRMT6	0.181687	3.36E-05	8.35E-05
PRMT7	-0.22465	2.58E-07	8.43E-07
PRMT8	-0.52766	2.96E-38	1.23E-36
PRND	-0.14802	7.53E-04	1.54E-03
PRNP	-0.06343	1.51E-01	1.98E-01
PRO0611	-0.11938	6.68E-03	1.17E-02
PRO0628	-0.11676	7.99E-03	1.38E-02
PRO1768	-0.00474	9.14E-01	9.32E-01
PROCA1	-0.05356	2.25E-01	2.83E-01
PROCR	0.11268	1.05E-02	1.77E-02
PROC	-0.06227	1.58E-01	2.07E-01
PRODH2	0.114642	9.22E-03	1.57E-02
PRODH	-0.37416	1.47E-18	1.51E-17

PROK1	-0.14372	1.07E-03	2.14E-03
PROK2	0.025145	5.69E-01	6.29E-01
PROKR1	0.010287	8.16E-01	8.48E-01
PROKR2	0.07157	1.05E-01	1.43E-01
PROL1	0.082823	6.04E-02	8.76E-02
PROM1	-0.12631	4.09E-03	7.45E-03
PROM2	-0.14934	6.74E-04	1.39E-03
PROP1	0.008031	8.56E-01	8.82E-01
PROS1	-0.13092	2.91E-03	5.43E-03
PROSC	0.065484	1.38E-01	1.83E-01
PROX1	0.053146	2.29E-01	2.87E-01
PROX2	-0.01155	7.94E-01	8.29E-01
PROZ	-0.01365	7.57E-01	7.98E-01
PRPF18	-0.02629	5.52E-01	6.13E-01
PRPF19	0.404889	9.77E-22	1.31E-20
PRPF31	0.119156	6.79E-03	1.19E-02
PRPF38A	0.20603	2.42E-06	6.98E-06
PRPF38B	-0.0743	9.21E-02	1.28E-01
PRPF39	-0.0207	6.39E-01	6.94E-01
PRPF3	0.035074	4.27E-01	4.93E-01
PRPF40A	0.512085	9.05E-36	3.25E-34
PRPF40B	0.04124	3.50E-01	4.16E-01
PRPF4B	-0.06207	1.60E-01	2.09E-01
PRPF4	0.494179	4.57E-33	1.41E-31
PRPF6	-0.14327	1.11E-03	2.22E-03
PRPF8	-0.19562	7.76E-06	2.11E-05
PRPH2	0.046919	2.88E-01	3.50E-01
PRPH	0.023553	5.94E-01	6.52E-01
PRPS1L1	0.055793	2.06E-01	2.62E-01
PRPS1	0.303193	2.07E-12	1.18E-11
PRPS2	0.302683	2.26E-12	1.28E-11
PRPSAP1	0.352901	1.50E-16	1.28E-15
PRPSAP2	0.13522	2.10E-03	4.01E-03
PRR11	0.789116	1.17E-110	2.29E-108
PRR12	-0.16388	1.87E-04	4.20E-04
PRR13	0.095851	2.96E-02	4.58E-02
PRR14	-0.15809	3.16E-04	6.86E-04
PRR15L	-0.42374	7.40E-24	1.20E-22
PRR15	0.153092	4.90E-04	1.03E-03
PRR16	0.192657	1.07E-05	2.85E-05
PRR18	0.042978	3.30E-01	3.96E-01
PRR19	0.325739	3.40E-14	2.30E-13
PRR22	-0.07569	8.62E-02	1.21E-01
PRR23A	0.010596	8.10E-01	8.43E-01

PRR23C	-0.01682	7.03E-01	7.51E-01
PRR24	-0.23116	1.13E-07	3.83E-07
PRR25	-0.06004	1.74E-01	2.25E-01
PRR3	0.002477	9.55E-01	9.64E-01
PRR4	-0.06214	1.59E-01	2.08E-01
PRR5-ARHGAP8	-0.21508	8.35E-07	2.55E-06
PRR5L	-0.0107	8.09E-01	8.42E-01
PRR5	-0.09743	2.70E-02	4.21E-02
PRR7	0.250561	8.19E-09	3.15E-08
PRRC1	-0.0818	6.36E-02	9.19E-02
PRRG1	-0.05594	2.05E-01	2.61E-01
PRRG2	-0.24847	1.10E-08	4.16E-08
PRRG3	-0.11042	1.22E-02	2.03E-02
PRRG4	0.005041	9.09E-01	9.27E-01
PRRT1	-0.04047	3.59E-01	4.26E-01
PRRT2	-0.22857	1.57E-07	5.25E-07
PRRT3	-0.19757	6.26E-06	1.72E-05
PRRT4	-0.10341	1.89E-02	3.04E-02
PRRX1	0.069656	1.14E-01	1.55E-01
PRRX2	0.165209	1.66E-04	3.74E-04
PRSS12	-0.36313	1.70E-17	1.59E-16
PRSS16	-0.24736	1.28E-08	4.82E-08
PRSS1	0.101634	2.11E-02	3.36E-02
PRSS21	0.053472	2.26E-01	2.84E-01
PRSS22	0.055375	2.10E-01	2.66E-01
PRSS23	0.15735	3.38E-04	7.28E-04
PRSS27	-0.10854	1.37E-02	2.27E-02
PRSS30P	-0.04786	2.78E-01	3.40E-01
PRSS33	-0.06448	1.44E-01	1.91E-01
PRSS35	-0.19025	1.38E-05	3.63E-05
PRSS36	-0.07168	1.04E-01	1.43E-01
PRSS37	-0.1138	9.75E-03	1.66E-02
PRSS38	-0.0004	9.93E-01	9.94E-01
PRSS3	0.273477	2.76E-10	1.26E-09
PRSS41	0.031808	4.71E-01	5.37E-01
PRSS42	0.130134	3.09E-03	5.74E-03
PRSS45	-0.10289	1.95E-02	3.13E-02
PRSS48	0.027285	5.37E-01	5.99E-01
PRSS50	0.030591	4.89E-01	5.53E-01
PRSS53	0.06002	1.74E-01	2.26E-01
PRSS54	-0.00787	8.59E-01	8.84E-01
PRSS55	-0.05976	1.76E-01	2.28E-01
PRSS8	-0.26488	1.02E-09	4.37E-09
PRSSL1	-0.15976	2.72E-04	5.96E-04

PRTFDC1	0.486529	5.84E-32	1.69E-30
PRTG	-0.09286	3.51E-02	5.35E-02
PRTN3	0.004559	9.18E-01	9.34E-01
PRUNE2	-0.18616	2.12E-05	5.44E-05
PRUNE	-0.059	1.81E-01	2.34E-01
PRX	-0.50097	4.51E-34	1.48E-32
PRY2	-0.03083	4.85E-01	5.50E-01
PSAPL1	-0.10782	1.44E-02	2.37E-02
PSAP	-0.03738	3.97E-01	4.64E-01
PSAT1	0.468506	1.85E-29	4.60E-28
PSCA	0.070788	1.09E-01	1.48E-01
PSD2	-0.05307	2.29E-01	2.88E-01
PSD3	-0.10515	1.70E-02	2.76E-02
PSD4	-0.36219	2.08E-17	1.93E-16
PSD	-0.13533	2.08E-03	3.98E-03
PSEN1	0.02396	5.87E-01	6.46E-01
PSEN2	-0.27889	1.18E-10	5.57E-10
PSENEN	0.173867	7.30E-05	1.74E-04
PSG10	0.115101	8.94E-03	1.53E-02
PSG11	0.095116	3.09E-02	4.76E-02
PSG1	0.174712	6.72E-05	1.61E-04
PSG2	0.143399	1.10E-03	2.20E-03
PSG3	0.253967	5.05E-09	1.99E-08
PSG4	0.183935	2.67E-05	6.74E-05
PSG5	0.165408	1.63E-04	3.68E-04
PSG6	0.188443	1.67E-05	4.35E-05
PSG7	0.122679	5.31E-03	9.48E-03
PSG8	0.199708	4.94E-06	1.38E-05
PSG9	0.169831	1.08E-04	2.50E-04
PSIMCT-1	0.242718	2.43E-08	8.85E-08
PSIP1	0.215967	7.51E-07	2.31E-06
PSKH1	-0.27751	1.47E-10	6.87E-10
PSKH2	-0.07345	9.59E-02	1.33E-01
PSMA1	0.316173	2.03E-13	1.27E-12
PSMA2	0.471681	6.87E-30	1.75E-28
PSMA3	0.317145	1.70E-13	1.08E-12
PSMA4	0.351484	2.02E-16	1.70E-15
PSMA5	0.43937	1.02E-25	1.90E-24
PSMA6	0.369008	4.66E-18	4.56E-17
PSMA7	0.393734	1.52E-20	1.87E-19
PSMA8	0.020923	6.36E-01	6.91E-01
PSMB10	-0.00491	9.12E-01	9.29E-01
PSMB11	0.019277	6.63E-01	7.15E-01
PSMB1	0.29359	1.07E-11	5.62E-11

PSMB2	0.422686	9.81E-24	1.58E-22
PSMB3	0.400263	3.09E-21	4.01E-20
PSMB4	0.288531	2.48E-11	1.25E-10
PSMB5	0.381401	2.81E-19	3.07E-18
PSMB6	0.24898	1.02E-08	3.89E-08
PSMB7	0.377375	7.10E-19	7.45E-18
PSMB8	0.212064	1.20E-06	3.59E-06
PSMB9	0.290971	1.66E-11	8.53E-11
PSMC1	0.296614	6.42E-12	3.46E-11
PSMC2	0.447576	9.77E-27	1.97E-25
PSMC3IP	0.658424	2.62E-65	2.56E-63
PSMC3	0.336036	4.64E-15	3.41E-14
PSMC4	0.48307	1.81E-31	5.11E-30
PSMC5	0.268749	5.70E-10	2.51E-09
PSMC6	0.359255	3.91E-17	3.55E-16
PSMD10	0.256973	3.28E-09	1.32E-08
PSMD11	0.591812	5.54E-50	3.74E-48
PSMD12	0.641415	4.95E-61	4.41E-59
PSMD13	0.22429	2.70E-07	8.79E-07
PSMD14	0.562409	2.74E-44	1.54E-42
PSMD1	0.484818	1.02E-31	2.94E-30
PSMD2	0.541494	1.42E-40	6.79E-39
PSMD3	0.426939	3.13E-24	5.25E-23
PSMD4	0.222635	3.32E-07	1.07E-06
PSMD5	0.076121	8.44E-02	1.18E-01
PSMD6	0.261203	1.77E-09	7.34E-09
PSMD7	0.342678	1.23E-15	9.54E-15
PSMD8	0.308005	8.85E-13	5.23E-12
PSMD9	0.306081	1.24E-12	7.23E-12
PSME1	0.13234	2.62E-03	4.92E-03
PSME2	0.375131	1.18E-18	1.22E-17
PSME3	0.508679	3.04E-35	1.06E-33
PSME4	0.460303	2.27E-28	5.20E-27
PSMF1	-0.05387	2.22E-01	2.80E-01
PSMG1	0.323256	5.44E-14	3.62E-13
PSMG2	0.105889	1.62E-02	2.65E-02
PSMG3	0.250978	7.72E-09	2.98E-08
PSMG4	0.056079	2.04E-01	2.59E-01
PSORS1C1	-0.12607	4.16E-03	7.58E-03
PSORS1C2	-0.07687	8.14E-02	1.15E-01
PSORS1C3	-0.26179	1.62E-09	6.75E-09
PSPC1	0.147214	8.05E-04	1.64E-03
PSPH	0.434554	3.90E-25	6.94E-24
PSPN	-0.0855	5.25E-02	7.71E-02

PSRC1	0.692691	7.92E-75	8.94E-73
PSTK	0.077859	7.75E-02	1.10E-01
PSTPIP1	-0.10704	1.51E-02	2.48E-02
PSTPIP2	-0.10594	1.62E-02	2.64E-02
PTAFR	0.075411	8.73E-02	1.22E-01
PTAR1	-0.30377	1.87E-12	1.07E-11
PTBP1	0.425558	4.55E-24	7.48E-23
PTBP2	0.109327	1.30E-02	2.17E-02
PTCD1	0.286176	3.65E-11	1.81E-10
PTCD2	0.170489	1.01E-04	2.36E-04
PTCD3	0.347294	4.81E-16	3.89E-15
PTCH1	-0.46478	5.83E-29	1.39E-27
PTCH2	-0.23174	1.04E-07	3.56E-07
PTCHD1	-0.3305	1.37E-14	9.59E-14
PTCHD2	-0.07501	8.90E-02	1.24E-01
PTCHD3	0.004966	9.10E-01	9.28E-01
PTCRA	-0.20067	4.44E-06	1.25E-05
PTDSS1	0.320006	9.99E-14	6.47E-13
PTDSS2	-0.07598	8.50E-02	1.19E-01
PTENP1	-0.02832	5.21E-01	5.85E-01
PTEN	-0.18236	3.14E-05	7.83E-05
PTER	0.071915	1.03E-01	1.41E-01
PTF1A	0.128801	3.41E-03	6.29E-03
PTGDR	-0.09354	3.38E-02	5.17E-02
PTGDS	-0.41276	1.32E-22	1.91E-21
PTGER1	-0.18165	3.37E-05	8.38E-05
PTGER2	-0.17012	1.05E-04	2.43E-04
PTGER3	-0.23893	4.05E-08	1.44E-07
PTGER4	-0.21288	1.09E-06	3.27E-06
PTGES2	0.214598	8.85E-07	2.70E-06
PTGES3	0.465161	5.19E-29	1.25E-27
PTGES	0.10986	1.26E-02	2.10E-02
PTGFRN	0.167231	1.37E-04	3.14E-04
PTGFR	-0.22812	1.66E-07	5.54E-07
PTGIR	-0.28568	3.96E-11	1.95E-10
PTGIS	-0.11164	1.12E-02	1.89E-02
PTGR1	0.136832	1.86E-03	3.58E-03
PTGR2	-0.09967	2.37E-02	3.74E-02
PTGS1	-0.08779	4.64E-02	6.89E-02
PTGS2	0.125421	4.36E-03	7.91E-03
PTH1R	-0.482	2.56E-31	7.18E-30
PTH2R	0.119079	6.82E-03	1.19E-02
PTH2	0.119067	6.83E-03	1.19E-02
PTHLH	0.311568	4.68E-13	2.85E-12

PTH	-0.01836	6.78E-01	7.28E-01
PTK2B	-0.34012	2.06E-15	1.55E-14
PTK2	-0.08058	6.77E-02	9.73E-02
PTK6	-0.2409	3.11E-08	1.12E-07
PTK7	-0.31365	3.21E-13	1.99E-12
PTMA	0.345528	6.91E-16	5.51E-15
PTMS	0.110437	1.21E-02	2.03E-02
PTN	-0.30977	6.46E-13	3.87E-12
PTOV1	-0.02964	5.02E-01	5.67E-01
PTP4A1	0.065891	1.35E-01	1.81E-01
PTP4A2	0.224051	2.78E-07	9.05E-07
PTP4A3	-0.12463	4.62E-03	8.34E-03
PTPDC1	0.035601	4.20E-01	4.86E-01
PTPLAD1	0.102677	1.98E-02	3.17E-02
PTPLAD2	-0.20932	1.65E-06	4.87E-06
PTPLA	0.023026	6.02E-01	6.60E-01
PTPLB	0.210979	1.36E-06	4.06E-06
PTPMT1	-0.08788	4.62E-02	6.87E-02
PTPN11	0.292664	1.25E-11	6.51E-11
PTPN12	0.171004	9.62E-05	2.25E-04
PTPN13	-0.3727	2.05E-18	2.07E-17
PTPN14	-0.0234	5.96E-01	6.54E-01
PTPN18	-0.30151	2.77E-12	1.56E-11
PTPN1	-0.00823	8.52E-01	8.79E-01
PTPN20A	0.012056	7.85E-01	8.22E-01
PTPN20B	0.032417	4.63E-01	5.29E-01
PTPN21	-0.38243	2.21E-19	2.45E-18
PTPN22	-0.03296	4.55E-01	5.22E-01
PTPN23	-0.13107	2.88E-03	5.38E-03
PTPN2	0.188776	1.61E-05	4.21E-05
PTPN3	-0.21153	1.27E-06	3.81E-06
PTPN4	-0.16143	2.34E-04	5.18E-04
PTPN5	-0.13516	2.11E-03	4.02E-03
PTPN6	-0.09744	2.70E-02	4.21E-02
PTPN7	0.008629	8.45E-01	8.73E-01
PTPN9	-0.01286	7.71E-01	8.10E-01
PTPRA	-0.21047	1.45E-06	4.29E-06
PTPRB	-0.34697	5.14E-16	4.15E-15
PTPRCAP	-0.07784	7.76E-02	1.10E-01
PTPRC	-0.08413	5.64E-02	8.23E-02
PTPRD	-0.25759	3.00E-09	1.21E-08
PTPRE	-0.45217	2.56E-27	5.42E-26
PTPRF	-0.04207	3.41E-01	4.06E-01
PTPRG	-0.13877	1.60E-03	3.10E-03

PTPRH	0.317657	1.54E-13	9.83E-13
PTPRJ	-0.10904	1.33E-02	2.21E-02
PTPRK	-0.05957	1.77E-01	2.29E-01
PTPRM	-0.34257	1.26E-15	9.73E-15
PTPRN2	-0.20861	1.79E-06	5.28E-06
PTPRN	0.398854	4.37E-21	5.62E-20
PTPRO	-0.06477	1.42E-01	1.88E-01
PTPRQ	-0.22539	2.35E-07	7.71E-07
PTPRR	-0.04134	3.49E-01	4.15E-01
PTPRS	-0.21148	1.28E-06	3.83E-06
PTPRT	-0.3431	1.13E-15	8.79E-15
PTPRU	-0.45153	3.09E-27	6.51E-26
PTPRVP	-0.10181	2.08E-02	3.32E-02
PTPRZ1	-0.18922	1.54E-05	4.03E-05
PTRF	-0.17547	6.24E-05	1.50E-04
PTRH1	-0.15736	3.37E-04	7.28E-04
PTRH2	0.36798	5.85E-18	5.66E-17
PTS	0.301614	2.72E-12	1.53E-11
PTTG1IP	-0.10378	1.85E-02	2.98E-02
PTTG1	0.705968	6.93E-79	8.34E-77
PTTG2	0.260379	1.99E-09	8.23E-09
PTTG3P	0.50209	3.06E-34	1.01E-32
PTX3	0.03	4.97E-01	5.62E-01
PTX4	-0.04154	3.47E-01	4.13E-01
PUF60	0.210606	1.42E-06	4.22E-06
PUM1	-0.13343	2.41E-03	4.55E-03
PUM2	-0.04468	3.12E-01	3.76E-01
PURA	-0.1932	1.01E-05	2.70E-05
PURB	0.128438	3.50E-03	6.45E-03
PURG	-0.11826	7.22E-03	1.26E-02
PUS10	-0.35483	1.00E-16	8.67E-16
PUS1	0.350498	2.48E-16	2.07E-15
PUS3	0.105446	1.67E-02	2.71E-02
PUS7L	0.397978	5.42E-21	6.91E-20
PUS7	0.388029	5.95E-20	6.92E-19
PUSL1	0.088362	4.50E-02	6.71E-02
PVALB	-0.14512	9.58E-04	1.93E-03
PVRIG	-0.09678	2.81E-02	4.36E-02
PVRL1	0.025881	5.58E-01	6.19E-01
PVRL2	0.12518	4.44E-03	8.04E-03
PVRL3	-0.12731	3.81E-03	6.96E-03
PVRL4	-0.27072	4.22E-10	1.89E-09
PVR	0.300312	3.41E-12	1.89E-11
PVT1	0.05406	2.21E-01	2.78E-01

PWP1	0.46914	1.52E-29	3.80E-28
PWP2	0.124138	4.78E-03	8.62E-03
PWRN1	-0.13374	2.36E-03	4.45E-03
PWRN2	-0.04291	3.31E-01	3.97E-01
PWWP2A	-0.23029	1.26E-07	4.26E-07
PWWP2B	-0.1942	9.05E-06	2.44E-05
PXDNL	0.093044	3.48E-02	5.30E-02
PXDN	0.223346	3.04E-07	9.83E-07
PXK	-0.06747	1.26E-01	1.70E-01
PXMP2	0.249502	9.50E-09	3.64E-08
PXMP4	-0.49358	5.59E-33	1.72E-31
PXN	0.039571	3.70E-01	4.36E-01
PXT1	-0.03778	3.92E-01	4.59E-01
PYCARD	-0.03797	3.90E-01	4.56E-01
PYCR1	0.28766	2.87E-11	1.44E-10
PYCR2	-0.30739	9.87E-13	5.80E-12
PYCRL	0.145791	9.06E-04	1.83E-03
PYDC1	0.117289	7.71E-03	1.34E-02
PYGB	-0.14765	7.76E-04	1.59E-03
PYGL	0.400924	2.62E-21	3.42E-20
PYGM	-0.37217	2.31E-18	2.33E-17
PYGO1	0.097809	2.64E-02	4.13E-02
PYGO2	-0.03941	3.72E-01	4.38E-01
PYHIN1	-0.04802	2.77E-01	3.39E-01
PYROXD1	0.103948	1.83E-02	2.95E-02
PYROXD2	-0.47995	4.97E-31	1.37E-29
PYY2	0.177687	5.02E-05	1.22E-04
PYY	0.078893	7.36E-02	1.05E-01
PZP	-0.24403	2.03E-08	7.46E-08
ProSAPiP1	-0.3547	1.03E-16	8.89E-16
QARS	-0.23402	7.76E-08	2.68E-07
QDPR	-0.14987	6.45E-04	1.33E-03
QKI	-0.01108	8.02E-01	8.37E-01
QPCTL	-0.01455	7.42E-01	7.85E-01
QPCT	0.308842	7.63E-13	4.53E-12
QPRT	0.127525	3.75E-03	6.86E-03
QRFPR	0.044375	3.15E-01	3.79E-01
QRFP	-0.03225	4.65E-01	5.31E-01
QRICH1	-0.07001	1.13E-01	1.53E-01
QRICH2	0.036377	4.10E-01	4.76E-01
QRSL1	0.225484	2.32E-07	7.62E-07
QSER1	0.274761	2.26E-10	1.04E-09
QSOX1	-0.19737	6.41E-06	1.76E-05
QSOX2	0.298317	4.80E-12	2.62E-11

QTRT1	-0.14861	7.17E-04	1.47E-03
QTRTD1	0.29935	4.02E-12	2.22E-11
R3HCC1	-0.07409	9.30E-02	1.29E-01
R3HDM1	0.624062	6.13E-57	4.98E-55
R3HDM2	-0.05595	2.05E-01	2.60E-01
R3HDML	0.037814	3.92E-01	4.59E-01
RAB10	0.563063	2.07E-44	1.18E-42
RAB11A	0.162727	2.08E-04	4.65E-04
RAB11B	-0.21072	1.40E-06	4.17E-06
RAB11FIP1	-0.20178	3.92E-06	1.11E-05
RAB11FIP2	-0.28474	4.62E-11	2.26E-10
RAB11FIP3	-0.36785	6.02E-18	5.81E-17
RAB11FIP4	-0.19659	6.97E-06	1.91E-05
RAB11FIP5	-0.08527	5.31E-02	7.79E-02
RAB12	-0.07548	8.70E-02	1.22E-01
RAB13	-0.04599	2.98E-01	3.61E-01
RAB14	-0.19089	1.29E-05	3.40E-05
RAB15	0.025982	5.56E-01	6.18E-01
RAB17	-0.38339	1.77E-19	1.98E-18
RAB18	0.097159	2.75E-02	4.27E-02
RAB19	0.024138	5.85E-01	6.44E-01
RAB1A	0.24522	1.72E-08	6.39E-08
RAB1B	-0.04834	2.73E-01	3.35E-01
RAB20	-0.20491	2.75E-06	7.90E-06
RAB21	0.140849	1.35E-03	2.66E-03
RAB22A	0.109989	1.25E-02	2.09E-02
RAB23	0.271006	4.04E-10	1.81E-09
RAB24	-0.12598	4.19E-03	7.62E-03
RAB25	-0.19048	1.35E-05	3.54E-05
RAB26	-0.00956	8.29E-01	8.59E-01
RAB27A	-0.32831	2.08E-14	1.44E-13
RAB27B	0.027171	5.38E-01	6.01E-01
RAB28	-0.00671	8.79E-01	9.02E-01
RAB2A	0.137725	1.73E-03	3.35E-03
RAB2B	-0.06026	1.72E-01	2.24E-01
RAB30	0.012407	7.79E-01	8.16E-01
RAB31	0.017752	6.88E-01	7.37E-01
RAB32	0.187887	1.77E-05	4.60E-05
RAB33A	0.085415	5.27E-02	7.74E-02
RAB33B	-0.04779	2.79E-01	3.41E-01
RAB34	0.050142	2.56E-01	3.17E-01
RAB35	0.357196	6.07E-17	5.39E-16
RAB36	-0.30315	2.08E-12	1.19E-11
RAB37	-0.21092	1.37E-06	4.08E-06

RAB38	-0.08848	4.47E-02	6.67E-02
RAB39B	0.158417	3.07E-04	6.67E-04
RAB39	0.250128	8.70E-09	3.34E-08
RAB3A	-0.09737	2.71E-02	4.23E-02
RAB3B	0.484374	1.18E-31	3.38E-30
RAB3C	-0.04897	2.67E-01	3.29E-01
RAB3D	-0.09525	3.07E-02	4.73E-02
RAB3GAP1	-0.01084	8.06E-01	8.40E-01
RAB3GAP2	-0.0431	3.29E-01	3.94E-01
RAB3IL1	0.066388	1.32E-01	1.77E-01
RAB3IP	0.003725	9.33E-01	9.47E-01
RAB40AL	-0.16142	2.35E-04	5.19E-04
RAB40A	-0.20203	3.81E-06	1.08E-05
RAB40B	-0.35743	5.78E-17	5.15E-16
RAB40C	-0.38858	5.23E-20	6.11E-19
RAB41	-0.09096	3.91E-02	5.89E-02
RAB42	0.215138	8.30E-07	2.54E-06
RAB43	-0.19292	1.04E-05	2.77E-05
RAB4A	-0.33595	4.72E-15	3.47E-14
RAB4B	-0.24999	8.87E-09	3.40E-08
RAB5A	-0.056	2.05E-01	2.60E-01
RAB5B	-0.20192	3.86E-06	1.09E-05
RAB5C	0.18739	1.87E-05	4.82E-05
RAB6A	0.252845	5.93E-09	2.32E-08
RAB6B	0.163432	1.95E-04	4.37E-04
RAB6C	0.085114	5.36E-02	7.85E-02
RAB7A	0.285946	3.79E-11	1.87E-10
RAB7L1	-0.08263	6.10E-02	8.84E-02
RAB8A	0.194312	8.94E-06	2.41E-05
RAB8B	0.062661	1.56E-01	2.04E-01
RAB9A	-0.07183	1.04E-01	1.42E-01
RAB9BP1	0.110355	1.22E-02	2.04E-02
RAB9B	-0.04006	3.64E-01	4.31E-01
RABAC1	-0.11002	1.25E-02	2.08E-02
RABEP1	-0.13551	2.06E-03	3.93E-03
RABEP2	-0.29597	7.15E-12	3.83E-11
RABEPK	0.218497	5.53E-07	1.73E-06
RABGAP1L	-0.16775	1.31E-04	3.00E-04
RABGAP1	-0.27071	4.23E-10	1.89E-09
RABGEF1	0.137127	1.81E-03	3.50E-03
RABGGTA	-0.06384	1.48E-01	1.95E-01
RABGGTB	0.11633	8.23E-03	1.42E-02
RABIF	0.076561	8.26E-02	1.16E-01
RABL2A	-0.40154	2.25E-21	2.95E-20

RABL2B	-0.32943	1.68E-14	1.17E-13
RABL3	0.086331	5.02E-02	7.40E-02
RABL5	0.059975	1.74E-01	2.26E-01
RAC1	0.23809	4.53E-08	1.60E-07
RAC2	-0.04381	3.21E-01	3.86E-01
RAC3	0.275164	2.12E-10	9.80E-10
RACGAP1P	0.53874	4.20E-40	1.98E-38
RACGAP1	0.84483	1.90E-141	8.09E-139
RAD17	-0.10389	1.84E-02	2.96E-02
RAD18	0.484753	1.05E-31	3.00E-30
RAD1	0.106948	1.52E-02	2.49E-02
RAD21L1	0.177548	5.09E-05	1.24E-04
RAD21	0.353171	1.42E-16	1.21E-15
RAD23A	0.156871	3.52E-04	7.58E-04
RAD23B	0.37208	2.35E-18	2.37E-17
RAD50	0.010661	8.09E-01	8.42E-01
RAD51AP1	0.828749	1.98E-131	6.01E-129
RAD51AP2	-0.01108	8.02E-01	8.37E-01
RAD51C	0.450514	4.16E-27	8.64E-26
RAD51L1	0.024393	5.81E-01	6.40E-01
RAD51L3	0.323681	5.02E-14	3.35E-13
RAD51	0.849354	1.80E-144	8.35E-142
RAD52	0.008763	8.43E-01	8.71E-01
RAD54B	0.650302	3.12E-63	2.95E-61
RAD54L2	-0.05081	2.50E-01	3.10E-01
RAD54L	0.836595	3.51E-136	1.15E-133
RAD9A	0.127625	3.72E-03	6.82E-03
RAD9B	0.224377	2.67E-07	8.71E-07
RADIL	-0.23802	4.58E-08	1.62E-07
RAE1	0.406415	6.65E-22	9.08E-21
RAET1E	0.248645	1.07E-08	4.07E-08
RAET1G	0.220479	4.33E-07	1.38E-06
RAET1K	0.21872	5.38E-07	1.69E-06
RAET1L	0.152666	5.08E-04	1.07E-03
RAF1	-0.12406	4.81E-03	8.67E-03
RAG1AP1	0.081802	6.36E-02	9.19E-02
RAG1	0.12082	6.05E-03	1.07E-02
RAG2	0.003934	9.29E-01	9.43E-01
RAGE	-0.13269	2.55E-03	4.80E-03
RAI14	-0.01858	6.74E-01	7.25E-01
RAI1	-0.2059	2.45E-06	7.08E-06
RAI2	-0.48695	5.08E-32	1.48E-30
RALA	0.400085	3.23E-21	4.18E-20
RALBP1	0.12646	4.05E-03	7.38E-03

RALB	0.268609	5.82E-10	2.56E-09
RALGAPA1	-0.14401	1.05E-03	2.10E-03
RALGAPA2	-0.21451	8.94E-07	2.72E-06
RALGAPB	0.082111	6.26E-02	9.05E-02
RALGDS	-0.24729	1.29E-08	4.87E-08
RALGPS1	-0.47255	5.23E-30	1.34E-28
RALGPS2	0.292323	1.32E-11	6.88E-11
RALYL	0.069061	1.18E-01	1.59E-01
RALY	0.089822	4.16E-02	6.24E-02
RAMP1	-0.00182	9.67E-01	9.75E-01
RAMP2	-0.30688	1.08E-12	6.32E-12
RAMP3	-0.381	3.08E-19	3.36E-18
RANBP10	-0.16831	1.24E-04	2.86E-04
RANBP17	-0.238	4.58E-08	1.62E-07
RANBP1	0.63534	1.44E-59	1.22E-57
RANBP2	-0.03081	4.85E-01	5.50E-01
RANBP3L	-0.32853	2.00E-14	1.38E-13
RANBP3	-0.15625	3.72E-04	7.98E-04
RANBP6	0.001989	9.64E-01	9.72E-01
RANBP9	-0.13671	1.87E-03	3.61E-03
RANGAP1	0.376898	7.91E-19	8.27E-18
RANGRF	-0.20062	4.46E-06	1.25E-05
RAN	0.63088	1.62E-58	1.36E-56
RAP1A	-0.0571	1.96E-01	2.50E-01
RAP1B	0.222084	3.56E-07	1.14E-06
RAP1GAP2	0.054721	2.15E-01	2.72E-01
RAP1GAP	-0.49849	1.06E-33	3.38E-32
RAP1GDS1	0.17129	9.36E-05	2.19E-04
RAP2A	0.22921	1.45E-07	4.86E-07
RAP2B	0.18602	2.15E-05	5.51E-05
RAP2C	0.301266	2.89E-12	1.62E-11
RAPGEF1	-0.10571	1.64E-02	2.67E-02
RAPGEF2	-0.3596	3.63E-17	3.31E-16
RAPGEF3	-0.41971	2.16E-23	3.36E-22
RAPGEF4	-0.1627	2.09E-04	4.66E-04
RAPGEF5	-0.34188	1.45E-15	1.11E-14
RAPGEF6	-0.06971	1.14E-01	1.55E-01
RAPGEFL1	-0.00933	8.33E-01	8.62E-01
RAPH1	-0.17726	5.23E-05	1.27E-04
RAPSN	-0.04959	2.61E-01	3.22E-01
RARA	-0.26501	1.00E-09	4.29E-09
RARB	-0.0314	4.77E-01	5.42E-01
RARG	-0.04277	3.33E-01	3.98E-01
RARRES1	0.161069	2.42E-04	5.35E-04

RARRES2	-0.08816	4.55E-02	6.78E-02
RARRES3	-0.09068	3.97E-02	5.98E-02
RARS2	0.160591	2.53E-04	5.56E-04
RARS	0.38487	1.25E-19	1.41E-18
RASA1	-0.06884	1.19E-01	1.60E-01
RASA2	0.098546	2.53E-02	3.97E-02
RASA3	-0.0085	8.47E-01	8.75E-01
RASA4P	-0.19167	1.19E-05	3.15E-05
RASA4	-0.06522	1.39E-01	1.85E-01
RASAL1	0.078342	7.57E-02	1.08E-01
RASAL2	0.196339	7.17E-06	1.96E-05
RASAL3	-0.18186	3.30E-05	8.21E-05
RASD1	-0.24122	2.97E-08	1.07E-07
RASD2	0.034914	4.29E-01	4.95E-01
RASEF	-0.08211	6.26E-02	9.05E-02
RASGEF1A	-0.03734	3.98E-01	4.64E-01
RASGEF1B	-0.12207	5.54E-03	9.87E-03
RASGEF1C	0.140886	1.35E-03	2.66E-03
RASGRF1	-0.36269	1.86E-17	1.74E-16
RASGRF2	-0.03858	3.82E-01	4.49E-01
RASGRP1	-0.18236	3.14E-05	7.83E-05
RASGRP2	-0.35522	9.23E-17	8.03E-16
RASGRP3	-0.04014	3.63E-01	4.30E-01
RASGRP4	-0.32604	3.21E-14	2.18E-13
RASIP1	-0.19313	1.02E-05	2.71E-05
RASL10A	-0.09849	2.54E-02	3.98E-02
RASL10B	0.15442	4.37E-04	9.25E-04
RASL11A	-0.29952	3.91E-12	2.16E-11
RASL11B	-0.21177	1.24E-06	3.71E-06
RASL12	-0.30148	2.78E-12	1.56E-11
RASSF10	-0.27574	1.94E-10	8.99E-10
RASSF1	-0.15399	4.53E-04	9.57E-04
RASSF2	-0.26	2.11E-09	8.68E-09
RASSF3	0.053072	2.29E-01	2.88E-01
RASSF4	-0.0835	5.83E-02	8.48E-02
RASSF5	-0.40948	3.06E-22	4.29E-21
RASSF6	0.079413	7.18E-02	1.03E-01
RASSF7	-0.40386	1.26E-21	1.68E-20
RASSF8	0.026369	5.50E-01	6.12E-01
RASSF9	-0.01716	6.98E-01	7.46E-01
RAVER1	0.113755	9.78E-03	1.66E-02
RAVER2	-0.09244	3.60E-02	5.46E-02
RAX2	-0.00616	8.89E-01	9.10E-01
RAX	0.240269	3.38E-08	1.21E-07

RB1CC1	0.06215	1.59E-01	2.08E-01
RB1	-0.01212	7.84E-01	8.21E-01
RBAK	-0.00219	9.60E-01	9.69E-01
RBBP4	0.178558	4.60E-05	1.13E-04
RBBP5	0.124794	4.56E-03	8.25E-03
RBBP6	-0.05099	2.48E-01	3.08E-01
RBBP7	0.386679	8.19E-20	9.39E-19
RBBP8	0.389767	3.94E-20	4.65E-19
RBBP9	-0.16868	1.20E-04	2.76E-04
RBCK1	0.003273	9.41E-01	9.54E-01
RBKS	-0.12744	3.77E-03	6.90E-03
RBL1	0.595509	9.68E-51	6.67E-49
RBL2	-0.48135	3.16E-31	8.79E-30
RBM10	0.024101	5.85E-01	6.44E-01
RBM11	-0.0062	8.88E-01	9.10E-01
RBM12B	0.089216	4.30E-02	6.43E-02
RBM12	0.260339	2.01E-09	8.28E-09
RBM14	0.192046	1.14E-05	3.03E-05
RBM15B	-0.02414	5.85E-01	6.44E-01
RBM15	0.225328	2.37E-07	7.77E-07
RBM16	-0.02822	5.23E-01	5.87E-01
RBM17	0.345413	7.07E-16	5.63E-15
RBM18	0.088271	4.53E-02	6.74E-02
RBM19	0.143232	1.12E-03	2.23E-03
RBM20	-0.03731	3.98E-01	4.65E-01
RBM22	-0.06468	1.43E-01	1.89E-01
RBM23	-0.00822	8.52E-01	8.80E-01
RBM24	-0.02181	6.21E-01	6.78E-01
RBM25	-0.03706	4.01E-01	4.68E-01
RBM26	0.021686	6.23E-01	6.79E-01
RBM27	0.148687	7.12E-04	1.46E-03
RBM28	0.513253	5.95E-36	2.15E-34
RBM33	-0.20079	4.38E-06	1.23E-05
RBM34	0.13587	2.00E-03	3.83E-03
RBM38	0.087089	4.82E-02	7.13E-02
RBM39	-0.14925	6.79E-04	1.40E-03
RBM3	-0.03696	4.03E-01	4.69E-01
RBM41	0.130589	2.99E-03	5.56E-03
RBM42	0.244866	1.81E-08	6.70E-08
RBM43	-0.27209	3.42E-10	1.54E-09
RBM44	-0.08115	6.57E-02	9.47E-02
RBM45	0.214603	8.85E-07	2.70E-06
RBM46	0.028317	5.21E-01	5.85E-01
RBM47	-0.1703	1.03E-04	2.40E-04

RBM4B	-0.05657	2.00E-01	2.55E-01
RBM4	0.156812	3.54E-04	7.62E-04
RBM5	-0.41223	1.51E-22	2.18E-21
RBM6	-0.2685	5.93E-10	2.61E-09
RBM7	0.14109	1.33E-03	2.62E-03
RBM8A	0.21445	9.01E-07	2.74E-06
RBM9	-0.11339	1.00E-02	1.70E-02
RBMS1	-0.04502	3.08E-01	3.72E-01
RBMS2	-0.39918	4.03E-21	5.19E-20
RBMS3	-0.31426	2.87E-13	1.79E-12
RBMX2	0.214719	8.73E-07	2.66E-06
RBMXL1	0.136735	1.87E-03	3.60E-03
RBMXL2	-0.12408	4.81E-03	8.66E-03
RBMXL3	0.010737	8.08E-01	8.41E-01
RBMX	0.182629	3.05E-05	7.64E-05
RBMY1A1	0.059388	1.78E-01	2.31E-01
RBMY1A3P	0.044646	3.12E-01	3.76E-01
RBMY1B	0.082874	6.02E-02	8.74E-02
RBMY1E	-0.03185	4.71E-01	5.36E-01
RBMY1F	0.029245	5.08E-01	5.72E-01
RBMY1J	0.042561	3.35E-01	4.01E-01
RBMY2EP	0.094096	3.28E-02	5.02E-02
RBMY2FP	0.037465	3.96E-01	4.63E-01
RBP1	0.158059	3.17E-04	6.87E-04
RBP2	-0.15769	3.28E-04	7.08E-04
RBP3	0.152828	5.01E-04	1.05E-03
RBP4	-0.04959	2.61E-01	3.23E-01
RBP5	-0.29284	1.21E-11	6.33E-11
RBP7	-0.05042	2.53E-01	3.14E-01
RBPJL	0.098136	2.59E-02	4.06E-02
RBPJ	-0.04804	2.76E-01	3.39E-01
RBPMS2	0.208241	1.87E-06	5.49E-06
RBPMS	-0.53657	9.79E-40	4.48E-38
RBX1	0.063672	1.49E-01	1.97E-01
RC3H1	-0.35401	1.19E-16	1.02E-15
RC3H2	0.185308	2.32E-05	5.91E-05
RCAN1	-0.21625	7.26E-07	2.24E-06
RCAN2	-0.44791	8.86E-27	1.80E-25
RCAN3	0.153265	4.82E-04	1.01E-03
RCBTB1	-0.01601	7.17E-01	7.64E-01
RCBTB2	-0.32024	9.56E-14	6.20E-13
RCC1	0.381942	2.48E-19	2.73E-18
RCC2	0.35505	9.57E-17	8.31E-16
RCCD1	0.303986	1.80E-12	1.03E-11

RCE1	0.303936	1.81E-12	1.04E-11
RCHY1	-0.05821	1.87E-01	2.41E-01
RCL1	0.004101	9.26E-01	9.41E-01
RCN1	0.244664	1.86E-08	6.88E-08
RCN2	0.155836	3.86E-04	8.25E-04
RCN3	0.087872	4.62E-02	6.87E-02
RCOR1	-0.04108	3.52E-01	4.18E-01
RCOR2	0.370702	3.20E-18	3.18E-17
RCOR3	-0.43104	1.03E-24	1.78E-23
RCSD1	-0.22087	4.13E-07	1.31E-06
RCVRN	-0.12679	3.95E-03	7.21E-03
RD3	-0.15947	2.80E-04	6.11E-04
RDBP	0.343132	1.12E-15	8.74E-15
RDH10	0.057995	1.89E-01	2.42E-01
RDH11	0.337513	3.46E-15	2.57E-14
RDH12	0.150334	6.20E-04	1.29E-03
RDH13	-0.1456	9.20E-04	1.86E-03
RDH14	0.023386	5.96E-01	6.54E-01
RDH16	0.101344	2.14E-02	3.41E-02
RDH5	-0.01474	7.39E-01	7.82E-01
RDH8	0.133258	2.44E-03	4.61E-03
RDM1	0.639114	1.79E-60	1.56E-58
RDX	0.289216	2.22E-11	1.13E-10
REC8	-0.03706	4.01E-01	4.68E-01
RECK	-0.15042	6.15E-04	1.28E-03
RECQL4	0.600113	1.07E-51	7.54E-50
RECQL5	-0.06025	1.72E-01	2.24E-01
RECQL	0.513629	5.20E-36	1.89E-34
REEP1	-0.15213	5.32E-04	1.11E-03
REEP2	0.284836	4.54E-11	2.23E-10
REEP3	0.084239	5.61E-02	8.19E-02
REEP4	0.265131	9.85E-10	4.22E-09
REEP5	-0.40463	1.04E-21	1.40E-20
REEP6	-0.10546	1.67E-02	2.71E-02
REG1A	-0.12139	5.81E-03	1.03E-02
REG1B	-0.00423	9.24E-01	9.39E-01
REG1P	0.03976	3.68E-01	4.34E-01
REG3A	-0.02658	5.47E-01	6.09E-01
REG3G	0.057098	1.96E-01	2.50E-01
REG4	0.019585	6.57E-01	7.10E-01
RELA	0.091414	3.81E-02	5.76E-02
RELB	0.202488	3.62E-06	1.03E-05
RELL1	0.152082	5.34E-04	1.12E-03
RELL2	0.193035	1.03E-05	2.74E-05

RELN	-0.01322	7.65E-01	8.04E-01
RELT	0.302556	2.31E-12	1.31E-11
REL	-0.09729	2.73E-02	4.24E-02
REM1	-0.3675	6.51E-18	6.27E-17
REM2	0.01174	7.90E-01	8.27E-01
RENBP	-0.07321	9.70E-02	1.34E-01
REN	-0.04511	3.07E-01	3.71E-01
REP15	0.07166	1.04E-01	1.43E-01
REPIN1	0.072502	1.00E-01	1.38E-01
REPS1	0.252039	6.65E-09	2.59E-08
REPS2	-0.43304	5.93E-25	1.04E-23
RER1	-0.09716	2.75E-02	4.27E-02
RERE	-0.27534	2.06E-10	9.54E-10
RERGL	-0.25516	4.26E-09	1.69E-08
RERG	-0.31417	2.92E-13	1.82E-12
RESP18	-0.027	5.41E-01	6.03E-01
REST	0.048187	2.75E-01	3.37E-01
RETNLB	0.204631	2.84E-06	8.14E-06
RETN	-0.2277	1.75E-07	5.83E-07
RETSAT	-0.25183	6.84E-09	2.66E-08
RET	-0.02762	5.32E-01	5.95E-01
REV1	-0.3938	1.50E-20	1.84E-19
REV3L	-0.26836	6.05E-10	2.65E-09
REXO1L1	-0.00609	8.90E-01	9.11E-01
REXO1	0.055813	2.06E-01	2.62E-01
REXO2	0.008318	8.51E-01	8.78E-01
REXO4	0.150166	6.29E-04	1.30E-03
RFC1	0.13526	2.10E-03	4.00E-03
RFC2	0.625845	2.39E-57	1.96E-55
RFC3	0.658224	2.95E-65	2.87E-63
RFC4	0.731145	3.06E-87	4.13E-85
RFC5	0.694444	2.37E-75	2.70E-73
RFESD	-0.02287	6.05E-01	6.62E-01
RFFL	0.433156	5.74E-25	1.01E-23
RFK	0.121997	5.57E-03	9.92E-03
RFNG	0.013033	7.68E-01	8.07E-01
RFPL1S	0.108729	1.36E-02	2.25E-02
RFPL1	0.134245	2.27E-03	4.30E-03
RFPL2	-0.09153	3.79E-02	5.72E-02
RFPL3S	-0.13397	2.31E-03	4.38E-03
RFPL3	0.02128	6.30E-01	6.85E-01
RFPL4A	0.003239	9.42E-01	9.54E-01
RFPL4B	0.089479	4.24E-02	6.34E-02
RFT1	0.137895	1.71E-03	3.31E-03

RFTN1	-0.57612	7.13E-47	4.50E-45
RFTN2	-0.23166	1.05E-07	3.59E-07
RFWD2	0.032398	4.63E-01	5.29E-01
RFWD3	0.515872	2.31E-36	8.58E-35
RFX1	-0.33325	8.01E-15	5.73E-14
RFX2	-0.31745	1.60E-13	1.02E-12
RFX3	0.059561	1.77E-01	2.29E-01
RFX4	0.026694	5.46E-01	6.08E-01
RFX5	-0.16183	2.26E-04	5.02E-04
RFX6	0.115868	8.49E-03	1.46E-02
RFX7	0.053357	2.27E-01	2.85E-01
RFX8	0.243036	2.32E-08	8.49E-08
RFXANK	-0.02538	5.66E-01	6.26E-01
RFXAP	-0.19305	1.02E-05	2.74E-05
RG9MTD1	0.379346	4.52E-19	4.83E-18
RG9MTD2	0.181775	3.33E-05	8.28E-05
RG9MTD3	-0.32588	3.31E-14	2.24E-13
RGAG1	-0.10502	1.71E-02	2.78E-02
RGAG4	-0.39303	1.80E-20	2.19E-19
RGL1	-0.33361	7.47E-15	5.37E-14
RGL2	-0.32036	9.36E-14	6.08E-13
RGL3	-0.26832	6.09E-10	2.67E-09
RGL4	-0.12197	5.58E-03	9.93E-03
RGMA	-0.3288	1.90E-14	1.31E-13
RGMB	-0.30694	1.07E-12	6.26E-12
RGNEF	-0.28817	2.64E-11	1.33E-10
RGN	-0.4036	1.35E-21	1.79E-20
RGP1	0.1723	8.49E-05	2.00E-04
RGPD1	-0.00204	9.63E-01	9.71E-01
RGPD3	-0.04716	2.85E-01	3.48E-01
RGPD4	-0.13801	1.69E-03	3.28E-03
RGPD5	-0.07227	1.01E-01	1.39E-01
RGPD6	-0.12823	3.56E-03	6.54E-03
RGPD8	0.047489	2.82E-01	3.44E-01
RGR	-0.02424	5.83E-01	6.42E-01
RGS10	0.126669	3.99E-03	7.27E-03
RGS11	-0.28861	2.45E-11	1.24E-10
RGS12	-0.2392	3.91E-08	1.39E-07
RGS13	-0.33462	6.13E-15	4.44E-14
RGS14	-0.15327	4.82E-04	1.01E-03
RGS16	-0.16205	2.22E-04	4.92E-04
RGS17	0.190892	1.29E-05	3.40E-05
RGS18	-0.1131	1.02E-02	1.73E-02
RGS19	0.091937	3.70E-02	5.61E-02

RGS1	0.054995	2.13E-01	2.69E-01
RGS20	0.397379	6.27E-21	7.94E-20
RGS21	-0.07262	9.97E-02	1.37E-01
RGS22	-0.35825	4.84E-17	4.35E-16
RGS2	0.052181	2.37E-01	2.96E-01
RGS3	-0.27224	3.34E-10	1.51E-09
RGS4	0.171674	9.02E-05	2.11E-04
RGS5	-0.36105	2.66E-17	2.44E-16
RGS6	-0.32588	3.31E-14	2.24E-13
RGS7BP	-0.28831	2.58E-11	1.30E-10
RGS7	-0.02498	5.72E-01	6.31E-01
RGS8	-0.00764	8.63E-01	8.88E-01
RGS9BP	0.053604	2.25E-01	2.82E-01
RGS9	-0.29767	5.36E-12	2.91E-11
RGSL1	0.042334	3.38E-01	4.03E-01
RHAG	0.059587	1.77E-01	2.29E-01
RHBDD1	0.101942	2.07E-02	3.30E-02
RHBDD2	-0.22177	3.70E-07	1.18E-06
RHBDD3	-0.00049	9.91E-01	9.93E-01
RHBDF1	-0.33105	1.23E-14	8.65E-14
RHBDF2	0.244132	2.00E-08	7.37E-08
RHBDL1	-0.26209	1.55E-09	6.48E-09
RHBDL2	-0.00043	9.92E-01	9.94E-01
RHBDL3	-0.09179	3.73E-02	5.65E-02
RHBG	0.255746	3.91E-09	1.56E-08
RHCE	-0.11989	6.45E-03	1.13E-02
RHCG	0.266244	8.33E-10	3.60E-09
RHD	-0.19206	1.14E-05	3.03E-05
RHEBL1	0.441409	5.71E-26	1.09E-24
RHEB	0.302944	2.16E-12	1.23E-11
RHOA	-0.05615	2.03E-01	2.59E-01
RHOBTB1	-0.12678	3.95E-03	7.22E-03
RHOBTB2	-0.57475	1.31E-46	8.17E-45
RHOBTB3	0.242325	2.56E-08	9.31E-08
RHOB	-0.22076	4.19E-07	1.33E-06
RHOC	0.186321	2.09E-05	5.35E-05
RHOD	0.053018	2.30E-01	2.88E-01
RHOF	0.261577	1.67E-09	6.96E-09
RHOG	-0.03562	4.20E-01	4.86E-01
RHOH	0.026241	5.52E-01	6.14E-01
RHOJ	-0.28934	2.17E-11	1.10E-10
RHOQ	0.094097	3.28E-02	5.02E-02
RHOT1	-0.03396	4.42E-01	5.08E-01
RHOT2	-0.22983	1.34E-07	4.51E-07

RHOU	-0.2784	1.27E-10	6.00E-10
RHOV	0.303258	2.04E-12	1.17E-11
RHOXF1	-0.13785	1.71E-03	3.32E-03
RHOXF2B	0.00126	9.77E-01	9.83E-01
RHO	0.061925	1.61E-01	2.10E-01
RHPN1	-0.1851	2.37E-05	6.03E-05
RHPN2	0.358483	4.61E-17	4.15E-16
RIBC1	-0.30446	1.65E-12	9.51E-12
RIBC2	0.272644	3.14E-10	1.43E-09
RIC3	-0.41819	3.22E-23	4.91E-22
RIC8A	-0.01463	7.41E-01	7.84E-01
RIC8B	0.174094	7.14E-05	1.70E-04
RICH2	-0.46031	2.27E-28	5.19E-27
RICTOR	0.036328	4.11E-01	4.77E-01
RIF1	0.37637	8.92E-19	9.29E-18
RILPL1	0.037168	4.00E-01	4.66E-01
RILPL2	-0.3842	1.47E-19	1.65E-18
RILP	-0.42126	1.43E-23	2.27E-22
RIMBP2	-0.12966	3.20E-03	5.94E-03
RIMBP3C	-0.07109	1.07E-01	1.46E-01
RIMBP3	-0.07881	7.39E-02	1.05E-01
RIMKLA	-0.10038	2.27E-02	3.60E-02
RIMKLB	-0.03263	4.60E-01	5.26E-01
RIMS1	-0.17211	8.65E-05	2.03E-04
RIMS2	0.397346	6.32E-21	8.00E-20
RIMS3	-0.21194	1.21E-06	3.64E-06
RIMS4	-0.12056	6.16E-03	1.09E-02
RIN1	-0.00962	8.28E-01	8.58E-01
RIN2	-0.18555	2.26E-05	5.77E-05
RIN3	-0.17026	1.03E-04	2.40E-04
RING1	-0.12629	4.10E-03	7.46E-03
RINL	-0.10796	1.42E-02	2.35E-02
RINT1	0.373718	1.63E-18	1.66E-17
RIOK1	0.390843	3.05E-20	3.64E-19
RIOK2	-0.10579	1.63E-02	2.66E-02
RIOK3	0.09781	2.64E-02	4.13E-02
RIPK1	-0.11369	9.82E-03	1.67E-02
RIPK2	0.448759	6.93E-27	1.41E-25
RIPK3	-0.29026	1.87E-11	9.56E-11
RIPK4	-0.11384	9.72E-03	1.65E-02
RIPPLY1	-0.12599	4.19E-03	7.62E-03
RIPPLY2	0.324254	4.51E-14	3.02E-13
RIT1	-0.05178	2.41E-01	3.01E-01
RIT2	0.084581	5.51E-02	8.05E-02

RLBP1	0.133615	2.38E-03	4.49E-03
RLF	0.159464	2.80E-04	6.11E-04
RLIM	0.078467	7.52E-02	1.07E-01
RLN1	0.184827	2.44E-05	6.19E-05
RLN2	0.13256	2.58E-03	4.84E-03
RLN3	-0.11016	1.24E-02	2.07E-02
RLTPR	0.070545	1.10E-01	1.50E-01
RMI1	0.426735	3.31E-24	5.52E-23
RMND1	0.042924	3.31E-01	3.96E-01
RMND5A	0.070024	1.12E-01	1.53E-01
RMND5B	-0.22845	1.59E-07	5.32E-07
RMRP	-0.01626	7.13E-01	7.60E-01
RMST	-0.17994	4.00E-05	9.86E-05
RNASE10	0.184449	2.53E-05	6.42E-05
RNASE11	0.048996	2.67E-01	3.29E-01
RNASE13	-0.20051	4.52E-06	1.27E-05
RNASE1	-0.57065	7.91E-46	4.78E-44
RNASE2	0.140042	1.44E-03	2.83E-03
RNASE3	0.093322	3.42E-02	5.22E-02
RNASE4	-0.36873	4.96E-18	4.84E-17
RNASE6	-0.11743	7.64E-03	1.32E-02
RNASE7	0.070616	1.09E-01	1.49E-01
RNASE8	-0.10411	1.81E-02	2.93E-02
RNASE9	0.011442	7.96E-01	8.31E-01
RNASEH1	0.640103	1.03E-60	9.08E-59
RNASEH2A	0.679353	5.74E-71	6.13E-69
RNASEH2B	0.100508	2.25E-02	3.57E-02
RNASEH2C	0.041083	3.52E-01	4.18E-01
RNASEK	-0.10202	2.06E-02	3.29E-02
RNASEL	-0.3293	1.72E-14	1.19E-13
RNASEN	0.264494	1.08E-09	4.61E-09
RNASET2	-0.15808	3.17E-04	6.86E-04
RND1	-0.13808	1.68E-03	3.26E-03
RND2	0.079721	7.07E-02	1.01E-01
RND3	0.302627	2.28E-12	1.30E-11
RNF103	-0.08991	4.14E-02	6.21E-02
RNF10	-0.03646	4.09E-01	4.75E-01
RNF111	-0.03422	4.38E-01	5.05E-01
RNF112	-0.10776	1.44E-02	2.38E-02
RNF113A	0.013677	7.57E-01	7.98E-01
RNF113B	0.026249	5.52E-01	6.14E-01
RNF114	0.128842	3.40E-03	6.27E-03
RNF115	0.216534	7.01E-07	2.17E-06
RNF11	-0.0182	6.80E-01	7.31E-01

RNF121	0.250928	7.78E-09	3.00E-08
RNF122	-0.10542	1.67E-02	2.72E-02
RNF123	-0.19743	6.36E-06	1.75E-05
RNF125	-0.29726	5.75E-12	3.12E-11
RNF126P1	-0.01978	6.54E-01	7.08E-01
RNF126	0.201511	4.04E-06	1.14E-05
RNF128	-0.0903	4.05E-02	6.09E-02
RNF130	-0.24022	3.41E-08	1.22E-07
RNF133	-0.16092	2.45E-04	5.41E-04
RNF135	-0.18702	1.94E-05	5.00E-05
RNF138P1	0.128746	3.42E-03	6.32E-03
RNF138	0.230274	1.26E-07	4.27E-07
RNF139	0.02231	6.13E-01	6.70E-01
RNF13	-0.05514	2.12E-01	2.68E-01
RNF141	-0.17744	5.14E-05	1.25E-04
RNF144A	0.128212	3.56E-03	6.55E-03
RNF144B	-0.30668	1.12E-12	6.53E-12
RNF145	-0.38547	1.09E-19	1.23E-18
RNF146	-0.34934	3.15E-16	2.60E-15
RNF148	-0.11608	8.37E-03	1.44E-02
RNF149	0.200718	4.42E-06	1.24E-05
RNF14	0.005947	8.93E-01	9.13E-01
RNF150	-0.15991	2.69E-04	5.89E-04
RNF151	-0.01284	7.71E-01	8.10E-01
RNF152	-0.1005	2.26E-02	3.57E-02
RNF157	0.260973	1.83E-09	7.58E-09
RNF160	0.074597	9.08E-02	1.26E-01
RNF165	-0.12215	5.51E-03	9.82E-03
RNF166	-0.13485	2.16E-03	4.12E-03
RNF167	-0.20842	1.84E-06	5.39E-06
RNF168	0.200322	4.62E-06	1.29E-05
RNF169	-0.08245	6.15E-02	8.91E-02
RNF170	-0.10921	1.31E-02	2.18E-02
RNF175	-0.30454	1.63E-12	9.40E-12
RNF17	0.012333	7.80E-01	8.18E-01
RNF180	-0.39549	9.94E-21	1.24E-19
RNF181	0.091379	3.82E-02	5.77E-02
RNF182	-0.04943	2.63E-01	3.24E-01
RNF183	0.099206	2.44E-02	3.83E-02
RNF185	-0.16448	1.77E-04	3.99E-04
RNF186	0.147902	7.60E-04	1.56E-03
RNF187	-0.09538	3.04E-02	4.69E-02
RNF19A	-0.07505	8.89E-02	1.24E-01
RNF19B	-0.19801	5.97E-06	1.65E-05

RNF207	-0.14053	1.39E-03	2.73E-03
RNF208	-0.17644	5.68E-05	1.37E-04
RNF20	-0.10769	1.45E-02	2.39E-02
RNF212	0.020014	6.50E-01	7.04E-01
RNF213	0.142433	1.19E-03	2.36E-03
RNF214	0.100052	2.32E-02	3.66E-02
RNF215	-0.10305	1.93E-02	3.11E-02
RNF216L	0.212684	1.11E-06	3.35E-06
RNF216	0.139877	1.46E-03	2.86E-03
RNF217	0.10303	1.94E-02	3.11E-02
RNF219	0.216676	6.89E-07	2.13E-06
RNF220	-0.04334	3.26E-01	3.92E-01
RNF222	0.092672	3.55E-02	5.40E-02
RNF24	0.129871	3.15E-03	5.85E-03
RNF25	0.090145	4.09E-02	6.14E-02
RNF26	0.278138	1.33E-10	6.25E-10
RNF2	0.253026	5.77E-09	2.27E-08
RNF31	0.042143	3.40E-01	4.05E-01
RNF32	0.003272	9.41E-01	9.54E-01
RNF34	0.513962	4.61E-36	1.68E-34
RNF38	-0.21301	1.07E-06	3.23E-06
RNF39	-0.26839	6.02E-10	2.64E-09
RNF40	-0.04796	2.77E-01	3.39E-01
RNF41	0.146497	8.55E-04	1.73E-03
RNF43	-0.08646	4.99E-02	7.36E-02
RNF44	-0.16265	2.10E-04	4.67E-04
RNF4	0.280165	9.64E-11	4.58E-10
RNF5P1	-0.06595	1.35E-01	1.80E-01
RNF5	-0.13856	1.62E-03	3.15E-03
RNF6	-0.02418	5.84E-01	6.43E-01
RNF7	0.352366	1.68E-16	1.42E-15
RNF8	0.084098	5.65E-02	8.24E-02
RNFT1	0.096318	2.88E-02	4.47E-02
RNFT2	0.341601	1.53E-15	1.17E-14
RNGTT	0.335992	4.68E-15	3.44E-14
RNH1	-0.3686	5.11E-18	4.97E-17
RNLS	-0.10687	1.53E-02	2.50E-02
RNMTL1	0.040427	3.60E-01	4.26E-01
RNMT	0.063024	1.53E-01	2.01E-01
RNPC3	-0.34242	1.30E-15	1.00E-14
RNPEPL1	-0.21367	9.89E-07	2.99E-06
RNPEP	-0.21866	5.42E-07	1.70E-06
RNPS1	0.114111	9.55E-03	1.63E-02
RNU11	0.027884	5.28E-01	5.91E-01

RNU4ATAC	0.047323	2.84E-01	3.46E-01
RNU6ATAC	0.068204	1.22E-01	1.65E-01
ROBLD3	-0.04798	2.77E-01	3.39E-01
ROBO1	0.118961	6.88E-03	1.20E-02
ROBO2	-0.55614	3.79E-43	2.03E-41
ROBO3	-0.12633	4.09E-03	7.44E-03
ROBO4	-0.3374	3.54E-15	2.62E-14
ROCK1	0.017243	6.96E-01	7.45E-01
ROCK2	0.114532	9.28E-03	1.58E-02
ROD1	0.279593	1.06E-10	5.00E-10
ROGDI	-0.46706	2.89E-29	7.06E-28
ROM1	-0.29768	5.35E-12	2.91E-11
ROMO1	0.185237	2.34E-05	5.95E-05
ROPN1B	-0.13735	1.78E-03	3.44E-03
ROPN1L	-0.15338	4.78E-04	1.01E-03
ROPN1	-0.05606	2.04E-01	2.60E-01
ROR1	-0.10098	2.19E-02	3.48E-02
ROR2	0.057102	1.96E-01	2.50E-01
RORA	-0.38196	2.47E-19	2.73E-18
RORB	-0.3229	5.81E-14	3.86E-13
RORC	-0.37295	1.93E-18	1.96E-17
ROS1	-0.3939	1.46E-20	1.79E-19
RP1-177G6.2	-0.38087	3.18E-19	3.46E-18
RP1L1	0.098435	2.55E-02	3.99E-02
RP1	-0.23203	1.01E-07	3.43E-07
RP2	0.147965	7.56E-04	1.55E-03
RP9P	0.257778	2.91E-09	1.18E-08
RP9	0.123324	5.07E-03	9.10E-03
RPA1	0.201272	4.15E-06	1.17E-05
RPA2	0.041181	3.51E-01	4.17E-01
RPA3	0.473634	3.72E-30	9.66E-29
RPA4	-0.01376	7.55E-01	7.96E-01
RPAIN	-0.22349	2.99E-07	9.67E-07
RPAP1	0.00116	9.79E-01	9.84E-01
RPAP2	0.425608	4.49E-24	7.39E-23
RPAP3	0.439708	9.24E-26	1.74E-24
RPE65	0.024725	5.76E-01	6.35E-01
RPE	0.521828	2.61E-37	1.01E-35
RPF1	0.280199	9.58E-11	4.56E-10
RPF2	0.473135	4.35E-30	1.12E-28
RPGRIP1L	0.046881	2.88E-01	3.51E-01
RPGRIP1	-0.11561	8.64E-03	1.48E-02
RPGR	-0.24889	1.04E-08	3.94E-08
RPH3AL	-0.27065	4.27E-10	1.91E-09

RPH3A	-0.09715	2.75E-02	4.27E-02
RPIA	0.229941	1.32E-07	4.45E-07
RPL10A	-0.16067	2.51E-04	5.53E-04
RPL10L	0.149718	6.53E-04	1.35E-03
RPL10	-0.10884	1.35E-02	2.23E-02
RPL11	-0.2258	2.23E-07	7.34E-07
RPL12	-0.06003	1.74E-01	2.26E-01
RPL13AP17	-0.40864	3.79E-22	5.28E-21
RPL13AP20	-0.09884	2.49E-02	3.91E-02
RPL13AP3	-0.09492	3.13E-02	4.81E-02
RPL13AP6	-0.00289	9.48E-01	9.59E-01
RPL13A	-0.13629	1.94E-03	3.72E-03
RPL13P5	-0.03822	3.87E-01	4.53E-01
RPL13	-0.16449	1.77E-04	3.99E-04
RPL14	-0.06717	1.28E-01	1.72E-01
RPL15	-0.18302	2.93E-05	7.35E-05
RPL17	-0.02677	5.44E-01	6.07E-01
RPL18A	0.032377	4.63E-01	5.29E-01
RPL18	-0.01099	8.04E-01	8.38E-01
RPL19P12	0.032331	4.64E-01	5.30E-01
RPL19	0.038323	3.85E-01	4.52E-01
RPL21P44	-0.2315	1.08E-07	3.67E-07
RPL21	-0.01084	8.06E-01	8.40E-01
RPL22L1	0.222545	3.36E-07	1.08E-06
RPL22	-0.14535	9.40E-04	1.90E-03
RPL23AP32	-0.21239	1.15E-06	3.46E-06
RPL23AP53	-0.2207	4.22E-07	1.34E-06
RPL23AP64	-0.2136	9.97E-07	3.02E-06
RPL23AP7	0.187109	1.92E-05	4.96E-05
RPL23AP82	0.047487	2.82E-01	3.44E-01
RPL23A	0.062442	1.57E-01	2.06E-01
RPL23P8	-0.11704	7.84E-03	1.36E-02
RPL23	-0.02972	5.01E-01	5.65E-01
RPL24	0.024027	5.86E-01	6.45E-01
RPL26L1	0.190091	1.41E-05	3.68E-05
RPL26	-0.07719	8.01E-02	1.13E-01
RPL27A	-0.0489	2.68E-01	3.30E-01
RPL27	0.179837	4.05E-05	9.96E-05
RPL28	-0.03263	4.60E-01	5.26E-01
RPL29P2	-0.00974	8.25E-01	8.56E-01
RPL29	-0.05717	1.95E-01	2.50E-01
RPL30	0.01214	7.83E-01	8.20E-01
RPL31P11	0.023268	5.98E-01	6.56E-01
RPL31	-0.00153	9.72E-01	9.79E-01

RPL32P3	-0.15482	4.22E-04	8.95E-04
RPL32	-0.02441	5.81E-01	6.40E-01
RPL34	-0.1748	6.67E-05	1.59E-04
RPL35A	0.16091	2.46E-04	5.41E-04
RPL35	0.096771	2.81E-02	4.36E-02
RPL36AL	-0.00949	8.30E-01	8.60E-01
RPL36A	0.179569	4.16E-05	1.02E-04
RPL36	0.019808	6.54E-01	7.07E-01
RPL37A	-0.03323	4.52E-01	5.18E-01
RPL37	0.040547	3.58E-01	4.25E-01
RPL38	0.171064	9.56E-05	2.24E-04
RPL39L	0.469649	1.30E-29	3.26E-28
RPL39	0.074336	9.20E-02	1.28E-01
RPL3L	0.13333	2.43E-03	4.59E-03
RPL3	-0.19804	5.95E-06	1.64E-05
RPL41	0.047151	2.86E-01	3.48E-01
RPL4	-0.01343	7.61E-01	8.01E-01
RPL5	-0.00314	9.43E-01	9.56E-01
RPL6	0.044253	3.16E-01	3.81E-01
RPL7A	-0.04958	2.61E-01	3.23E-01
RPL7L1	0.305022	1.50E-12	8.67E-12
RPL7	-0.03415	4.39E-01	5.06E-01
RPL8	0.045662	3.01E-01	3.65E-01
RPL9	0.003934	9.29E-01	9.43E-01
RPLP0P2	0.258929	2.46E-09	1.01E-08
RPLP0	0.243735	2.11E-08	7.75E-08
RPLP1	-0.08669	4.93E-02	7.28E-02
RPLP2	-0.07303	9.78E-02	1.35E-01
RPN1	0.341559	1.54E-15	1.18E-14
RPN2	0.207212	2.11E-06	6.14E-06
RPP14	0.078707	7.43E-02	1.06E-01
RPP21	0.10508	1.71E-02	2.77E-02
RPP25	0.35595	7.91E-17	6.91E-16
RPP30	0.375681	1.04E-18	1.08E-17
RPP38	0.107956	1.42E-02	2.35E-02
RPP40	0.39764	5.89E-21	7.47E-20
RPPH1	-0.00433	9.22E-01	9.38E-01
RPRD1A	0.13496	2.15E-03	4.08E-03
RPRD1B	0.258547	2.61E-09	1.06E-08
RPRD2	-0.2492	9.91E-09	3.78E-08
RPRML	-0.09687	2.79E-02	4.34E-02
RPRM	0.019427	6.60E-01	7.13E-01
RPS10P7	-0.2751	2.14E-10	9.90E-10
RPS10	0.092975	3.49E-02	5.32E-02

RPS11	-0.02484	5.74E-01	6.33E-01
RPS12	-0.01068	8.09E-01	8.42E-01
RPS13	-0.09133	3.83E-02	5.78E-02
RPS14	-0.08026	6.88E-02	9.87E-02
RPS15AP10	-0.22351	2.98E-07	9.65E-07
RPS15A	-0.01462	7.41E-01	7.84E-01
RPS15	0.049101	2.66E-01	3.28E-01
RPS16	0.059995	1.74E-01	2.26E-01
RPS17	0.036447	4.09E-01	4.76E-01
RPS18	0.037478	3.96E-01	4.63E-01
RPS19BP1	-0.00793	8.57E-01	8.84E-01
RPS19	0.1362	1.95E-03	3.74E-03
RPS20	-0.07487	8.96E-02	1.25E-01
RPS21	0.073641	9.50E-02	1.32E-01
RPS23	-0.12619	4.13E-03	7.51E-03
RPS24	-0.05826	1.87E-01	2.40E-01
RPS25	-0.08459	5.51E-02	8.05E-02
RPS26P11	0.04778	2.79E-01	3.41E-01
RPS26	0.153909	4.56E-04	9.64E-04
RPS27A	0.170345	1.02E-04	2.39E-04
RPS27L	-0.21158	1.27E-06	3.79E-06
RPS27	-0.0499	2.58E-01	3.19E-01
RPS28	-0.06758	1.26E-01	1.69E-01
RPS29	0.067067	1.29E-01	1.72E-01
RPS2P32	0.284388	4.89E-11	2.39E-10
RPS2	0.026624	5.47E-01	6.09E-01
RPS3A	-0.00701	8.74E-01	8.98E-01
RPS3	0.074161	9.27E-02	1.29E-01
RPS4X	-0.13542	2.07E-03	3.96E-03
RPS4Y1	0.024666	5.77E-01	6.36E-01
RPS4Y2	-0.04662	2.91E-01	3.54E-01
RPS5	0.067917	1.24E-01	1.67E-01
RPS6KA1	-0.36865	5.05E-18	4.92E-17
RPS6KA2	-0.53805	5.49E-40	2.55E-38
RPS6KA3	-0.24605	1.54E-08	5.73E-08
RPS6KA4	0.094697	3.17E-02	4.86E-02
RPS6KA5	-0.18873	1.62E-05	4.23E-05
RPS6KA6	0.039786	3.68E-01	4.34E-01
RPS6KB1	0.326626	2.87E-14	1.96E-13
RPS6KB2	0.208008	1.92E-06	5.63E-06
RPS6KC1	0.173404	7.63E-05	1.81E-04
RPS6KL1	-0.11067	1.20E-02	2.00E-02
RPS6	-0.04661	2.91E-01	3.54E-01
RPS7	0.230226	1.27E-07	4.29E-07

RPS8	-0.01917	6.64E-01	7.16E-01
RPS9	-0.14017	1.43E-03	2.80E-03
RPSAP52	0.188975	1.58E-05	4.13E-05
RPSAP58	0.075432	8.72E-02	1.22E-01
RPSAP9	0.046945	2.88E-01	3.50E-01
RPSA	0.081053	6.61E-02	9.51E-02
RPTN	0.029252	5.08E-01	5.72E-01
RPTOR	0.121925	5.60E-03	9.96E-03
RPUSD1	0.074399	9.17E-02	1.27E-01
RPUSD2	0.036791	4.05E-01	4.71E-01
RPUSD3	0.04863	2.71E-01	3.33E-01
RPUSD4	0.123503	5.01E-03	8.99E-03
RQCD1	0.54966	5.41E-42	2.73E-40
RRAD	-0.29168	1.47E-11	7.63E-11
RRAGA	-0.01614	7.15E-01	7.62E-01
RRAGB	-0.15956	2.77E-04	6.06E-04
RRAGC	0.16943	1.12E-04	2.59E-04
RRAGD	0.13995	1.45E-03	2.85E-03
RRAS2	-0.05573	2.07E-01	2.62E-01
RRAS	-0.1477	7.73E-04	1.58E-03
RRBP1	-0.25846	2.64E-09	1.07E-08
RREB1	-0.09894	2.47E-02	3.89E-02
RRH	-0.1856	2.25E-05	5.75E-05
RRM1	0.587888	3.45E-49	2.30E-47
RRM2B	-0.37381	1.59E-18	1.63E-17
RRM2	1	1.00E-196	1.00E-192
RRN3P1	-0.22573	2.25E-07	7.41E-07
RRN3P2	-0.13301	2.49E-03	4.69E-03
RRN3P3	-0.20853	1.81E-06	5.32E-06
RRN3	-0.02345	5.95E-01	6.53E-01
RRP12	0.139231	1.54E-03	3.00E-03
RRP15	0.111302	1.15E-02	1.93E-02
RRP1B	0.310938	5.24E-13	3.17E-12
RRP1	0.25864	2.57E-09	1.05E-08
RRP7A	0.206417	2.31E-06	6.70E-06
RRP7B	0.000393	9.93E-01	9.94E-01
RRP8	-0.12543	4.36E-03	7.91E-03
RRP9	0.265312	9.58E-10	4.11E-09
RRS1	0.188824	1.61E-05	4.19E-05
RS1	-0.46722	2.76E-29	6.73E-28
RSAD1	-0.26887	5.60E-10	2.47E-09
RSAD2	0.108978	1.33E-02	2.22E-02
RSBN1L	-0.05152	2.43E-01	3.03E-01
RSBN1	-0.18583	2.20E-05	5.62E-05

RSC1A1	0.029616	5.02E-01	5.67E-01
RSF1	0.101007	2.19E-02	3.48E-02
RSL1D1	0.004666	9.16E-01	9.33E-01
RSL24D1	0.143643	1.08E-03	2.16E-03
RSPH10B2	-0.25991	2.13E-09	8.78E-09
RSPH1	-0.3125	3.96E-13	2.42E-12
RSPH3	-0.01379	7.55E-01	7.96E-01
RSPH4A	-0.26012	2.07E-09	8.53E-09
RSPH6A	0.133688	2.36E-03	4.47E-03
RSPH9	-0.15405	4.51E-04	9.52E-04
RSPO1	-0.42575	4.31E-24	7.12E-23
RSPO2	-0.50708	5.34E-35	1.83E-33
RSPO3	0.253926	5.08E-09	2.00E-08
RSPO4	-0.24892	1.03E-08	3.92E-08
RSPRY1	0.089632	4.20E-02	6.30E-02
RSRC1	0.546655	1.82E-41	8.94E-40
RSRC2	0.045434	3.03E-01	3.67E-01
RSU1	-0.15582	3.86E-04	8.25E-04
RTBDN	0.206514	2.29E-06	6.63E-06
RTCD1	0.407563	4.98E-22	6.87E-21
RTDR1	-0.10393	1.83E-02	2.96E-02
RTEL1	-0.0549	2.14E-01	2.70E-01
RTF1	-0.02212	6.17E-01	6.73E-01
RTKN2	0.091873	3.71E-02	5.62E-02
RTKN	0.28544	4.12E-11	2.03E-10
RTL1	0.175736	6.08E-05	1.46E-04
RTN1	-0.21533	8.11E-07	2.49E-06
RTN2	0.015275	7.29E-01	7.74E-01
RTN3	0.191699	1.18E-05	3.14E-05
RTN4IP1	0.262757	1.40E-09	5.90E-09
RTN4RL1	-0.43205	7.79E-25	1.35E-23
RTN4RL2	-0.07362	9.51E-02	1.32E-01
RTN4R	-0.03483	4.30E-01	4.97E-01
RTN4	0.286828	3.28E-11	1.63E-10
RTP1	-0.02639	5.50E-01	6.12E-01
RTP2	-0.02667	5.46E-01	6.08E-01
RTP3	0.092234	3.64E-02	5.52E-02
RTP4	0.103191	1.92E-02	3.08E-02
RTTN	0.315541	2.28E-13	1.42E-12
RUFY1	-0.31663	1.87E-13	1.18E-12
RUFY2	-0.09198	3.69E-02	5.59E-02
RUFY3	-0.35414	1.16E-16	9.97E-16
RUFY4	0.012815	7.72E-01	8.10E-01
RUNDC1	-0.03778	3.92E-01	4.59E-01

RUNDC2A	-0.10563	1.65E-02	2.69E-02
RUNDC2C	-0.26767	6.71E-10	2.93E-09
RUNDC3A	0.274708	2.28E-10	1.05E-09
RUNDC3B	-0.03114	4.81E-01	5.46E-01
RUNX1T1	-0.20872	1.77E-06	5.21E-06
RUNX1	-0.27385	2.60E-10	1.19E-09
RUNX2	0.076528	8.27E-02	1.16E-01
RUNX3	-0.05015	2.56E-01	3.17E-01
RUSC1	0.153671	4.66E-04	9.82E-04
RUSC2	0.00989	8.23E-01	8.54E-01
RUVBL1	0.446106	1.49E-26	2.97E-25
RUVBL2	0.353834	1.24E-16	1.06E-15
RWDD1	0.132559	2.58E-03	4.84E-03
RWDD2A	0.078285	7.59E-02	1.08E-01
RWDD2B	-0.1133	1.01E-02	1.71E-02
RWDD3	0.095115	3.09E-02	4.76E-02
RWDD4A	0.09891	2.48E-02	3.89E-02
RXFP1	-0.22033	4.41E-07	1.40E-06
RXFP2	-0.21632	7.20E-07	2.22E-06
RXFP3	0.107758	1.44E-02	2.38E-02
RXFP4	-0.15781	3.24E-04	7.01E-04
RXRA	-0.39662	7.55E-21	9.48E-20
RXRB	-0.24851	1.09E-08	4.14E-08
RXRG	-0.37978	4.08E-19	4.38E-18
RYBP	-0.046	2.97E-01	3.61E-01
RYK	0.113805	9.74E-03	1.66E-02
RYR1	-0.08056	6.77E-02	9.73E-02
RYR2	-0.33607	4.61E-15	3.39E-14
RYR3	-0.23902	4.00E-08	1.42E-07
S100A10	0.108205	1.40E-02	2.32E-02
S100A11	0.105737	1.64E-02	2.67E-02
S100A12	0.159615	2.76E-04	6.04E-04
S100A13	-0.06416	1.46E-01	1.93E-01
S100A14	-0.12292	5.22E-03	9.33E-03
S100A16	0.099645	2.37E-02	3.74E-02
S100A1	-0.19439	8.86E-06	2.39E-05
S100A2	0.147039	8.17E-04	1.66E-03
S100A3	0.105374	1.67E-02	2.73E-02
S100A4	-0.12139	5.81E-03	1.03E-02
S100A5	0.125187	4.44E-03	8.04E-03
S100A6	-0.05387	2.22E-01	2.80E-01
S100A7A	0.144073	1.04E-03	2.09E-03
S100A7	0.215998	7.48E-07	2.30E-06
S100A8	0.236146	5.87E-08	2.05E-07

S100A9	0.172201	8.57E-05	2.02E-04
S100B	-0.26341	1.27E-09	5.38E-09
S100G	-0.12011	6.35E-03	1.12E-02
S100PBP	-0.02508	5.70E-01	6.30E-01
S100P	0.047442	2.83E-01	3.45E-01
S100Z	-0.21703	6.60E-07	2.05E-06
S1PR1	-0.29102	1.65E-11	8.47E-11
S1PR2	-0.17404	7.18E-05	1.71E-04
S1PR3	0.012644	7.75E-01	8.13E-01
S1PR4	-0.34096	1.74E-15	1.32E-14
S1PR5	0.146662	8.43E-04	1.71E-03
SAA1	0.212238	1.17E-06	3.52E-06
SAA2	0.217127	6.53E-07	2.03E-06
SAA3P	-0.02067	6.40E-01	6.94E-01
SAA4	0.162598	2.11E-04	4.70E-04
SAAL1	0.467958	2.19E-29	5.43E-28
SAC3D1	0.287544	2.92E-11	1.46E-10
SACM1L	-0.35812	4.99E-17	4.47E-16
SACS	0.246301	1.48E-08	5.54E-08
SAE1	0.449743	5.21E-27	1.07E-25
SAFB2	-0.15722	3.42E-04	7.36E-04
SAFB	-0.00393	9.29E-01	9.43E-01
SAGE1	0.180336	3.85E-05	9.51E-05
SAG	0.052109	2.38E-01	2.97E-01
SALL1	0.069612	1.15E-01	1.56E-01
SALL2	-0.21621	7.29E-07	2.25E-06
SALL3	0.09367	3.36E-02	5.13E-02
SALL4	0.063307	1.51E-01	1.99E-01
SAMD10	-0.12075	6.07E-03	1.07E-02
SAMD11	-0.2772	1.54E-10	7.20E-10
SAMD12	-0.29064	1.75E-11	9.00E-11
SAMD13	-0.02923	5.08E-01	5.72E-01
SAMD14	-0.08456	5.51E-02	8.06E-02
SAMD1	0.182479	3.10E-05	7.75E-05
SAMD3	-0.08512	5.36E-02	7.85E-02
SAMD4A	-0.03811	3.88E-01	4.55E-01
SAMD4B	0.17549	6.23E-05	1.50E-04
SAMD5	-0.15052	6.10E-04	1.27E-03
SAMD7	0.048331	2.74E-01	3.36E-01
SAMD8	0.358119	4.99E-17	4.47E-16
SAMD9L	0.131585	2.77E-03	5.19E-03
SAMD9	0.208292	1.86E-06	5.46E-06
SAMHD1	0.050511	2.53E-01	3.13E-01
SAMM50	0.121695	5.69E-03	1.01E-02

SAMSN1	0.053122	2.29E-01	2.87E-01
SAP130	0.257374	3.09E-09	1.25E-08
SAP18	-0.02134	6.29E-01	6.85E-01
SAP30BP	0.17572	6.09E-05	1.46E-04
SAP30L	-0.39832	4.99E-21	6.39E-20
SAP30	0.383919	1.57E-19	1.76E-18
SAPS1	0.207534	2.03E-06	5.93E-06
SAPS2	-0.32019	9.65E-14	6.26E-13
SAPS3	0.208415	1.84E-06	5.39E-06
SAR1A	0.0255	5.64E-01	6.24E-01
SAR1B	0.105504	1.66E-02	2.71E-02
SARDH	-0.27164	3.66E-10	1.65E-09
SARM1	-0.31634	1.97E-13	1.24E-12
SARNP	0.313041	3.59E-13	2.20E-12
SARS2	0.149426	6.69E-04	1.38E-03
SARS	0.000875	9.84E-01	9.88E-01
SART1	0.125035	4.49E-03	8.12E-03
SART3	0.320505	9.11E-14	5.92E-13
SASH1	-0.19778	6.12E-06	1.68E-05
SASH3	-0.13213	2.66E-03	4.99E-03
SASS6	0.640461	8.44E-61	7.47E-59
SAT1	-0.08357	5.81E-02	8.45E-02
SAT2	-0.23222	9.81E-08	3.35E-07
SATB1	-0.3693	4.37E-18	4.29E-17
SATB2	0.023937	5.88E-01	6.46E-01
SATL1	-0.08796	4.60E-02	6.84E-02
SAV1	-0.00069	9.88E-01	9.90E-01
SBDSP1	0.059261	1.79E-01	2.32E-01
SBDS	-0.11122	1.15E-02	1.94E-02
SBF1P1	0.014581	7.41E-01	7.85E-01
SBF1	-0.08937	4.26E-02	6.38E-02
SBF2	0.150447	6.14E-04	1.27E-03
SBK1	-0.09838	2.56E-02	4.00E-02
SBK2	0.15318	4.86E-04	1.02E-03
SBNO1	0.312781	3.76E-13	2.31E-12
SBNO2	0.104056	1.82E-02	2.94E-02
SBSN	0.337807	3.27E-15	2.43E-14
SC4MOL	0.136385	1.92E-03	3.69E-03
SC5DL	0.039976	3.65E-01	4.32E-01
SC65	0.405549	8.27E-22	1.12E-20
SCAF1	0.04001	3.65E-01	4.31E-01
SCAI	-0.39253	2.03E-20	2.47E-19
SCAMP1	0.043839	3.21E-01	3.86E-01
SCAMP2	-0.34716	4.94E-16	3.99E-15

SCAMP3	-0.05525	2.11E-01	2.67E-01
SCAMP4	-0.26063	1.92E-09	7.95E-09
SCAMP5	0.13157	2.78E-03	5.19E-03
SCAND1	-0.00034	9.94E-01	9.95E-01
SCAND2	-0.34998	2.77E-16	2.29E-15
SCAND3	-0.02963	5.02E-01	5.67E-01
SCAPER	-0.32684	2.76E-14	1.88E-13
SCAP	-0.15753	3.32E-04	7.17E-04
SCARA3	-0.37831	5.72E-19	6.05E-18
SCARA5	-0.35877	4.33E-17	3.91E-16
SCARB1	0.263256	1.30E-09	5.50E-09
SCARB2	0.05721	1.95E-01	2.49E-01
SCARF1	-0.42648	3.55E-24	5.90E-23
SCARF2	-0.19343	9.84E-06	2.63E-05
SCARNA10	0.039576	3.70E-01	4.36E-01
SCARNA11	0.025751	5.60E-01	6.21E-01
SCARNA12	0.185158	2.35E-05	6.00E-05
SCARNA15	-0.0012	9.78E-01	9.84E-01
SCARNA16	0.076499	8.29E-02	1.16E-01
SCARNA17	-0.01893	6.68E-01	7.20E-01
SCARNA18	0.123574	4.98E-03	8.95E-03
SCARNA1	0.062677	1.56E-01	2.04E-01
SCARNA20	0.104201	1.80E-02	2.91E-02
SCARNA21	0.020635	6.40E-01	6.95E-01
SCARNA22	-0.02429	5.82E-01	6.41E-01
SCARNA2	-0.11763	7.54E-03	1.31E-02
SCARNA3	0.001855	9.67E-01	9.74E-01
SCARNA4	0.009541	8.29E-01	8.59E-01
SCARNA5	-0.05967	1.76E-01	2.28E-01
SCARNA6	-0.07417	9.27E-02	1.29E-01
SCARNA7	-0.26882	5.64E-10	2.49E-09
SCARNA9L	0.009247	8.34E-01	8.64E-01
SCARNA9	-0.06845	1.21E-01	1.63E-01
SCCPDH	0.05765	1.91E-01	2.45E-01
SCD5	-0.03372	4.45E-01	5.12E-01
SCD	0.208388	1.84E-06	5.40E-06
SCEL	-0.19107	1.27E-05	3.34E-05
SCFD1	0.225375	2.36E-07	7.72E-07
SCFD2	0.048348	2.73E-01	3.35E-01
SCG2	0.039166	3.75E-01	4.41E-01
SCG3	0.214381	9.09E-07	2.76E-06
SCG5	0.23783	4.69E-08	1.65E-07
SCGB1A1	-0.31405	2.99E-13	1.85E-12
SCGB1C1	0.096114	2.92E-02	4.52E-02

SCGB1D1	0.028456	5.19E-01	5.83E-01
SCGB1D2	0.097463	2.70E-02	4.20E-02
SCGB2A1	-0.23951	3.75E-08	1.34E-07
SCGB2A2	0.014206	7.48E-01	7.90E-01
SCGB3A1	-0.50135	3.95E-34	1.30E-32
SCGB3A2	-0.53255	4.62E-39	2.03E-37
SCGBL	-0.22879	1.53E-07	5.11E-07
SCGN	-0.04205	3.41E-01	4.07E-01
SCHIP1	0.138251	1.66E-03	3.22E-03
SCIN	-0.0674	1.27E-01	1.70E-01
SCLT1	0.551583	2.47E-42	1.29E-40
SCLY	0.125476	4.35E-03	7.89E-03
SCMH1	-0.29914	4.17E-12	2.30E-11
SCML1	0.098557	2.53E-02	3.97E-02
SCML2	0.393564	1.59E-20	1.94E-19
SCML4	-0.20889	1.74E-06	5.12E-06
SCN10A	-0.17312	7.85E-05	1.86E-04
SCN11A	-0.32481	4.05E-14	2.73E-13
SCN1A	-0.32085	8.53E-14	5.56E-13
SCN1B	-0.1393	1.53E-03	2.98E-03
SCN2A	-0.00313	9.43E-01	9.56E-01
SCN2B	-0.38937	4.34E-20	5.10E-19
SCN3A	0.083501	5.83E-02	8.48E-02
SCN3B	-0.07338	9.62E-02	1.33E-01
SCN4A	0.00813	8.54E-01	8.81E-01
SCN4B	-0.59448	1.57E-50	1.08E-48
SCN5A	0.01015	8.18E-01	8.50E-01
SCN7A	-0.51212	8.93E-36	3.22E-34
SCN8A	0.075941	8.51E-02	1.19E-01
SCN9A	-0.10425	1.80E-02	2.91E-02
SCNM1	0.289844	2.00E-11	1.02E-10
SCNN1A	-0.23238	9.61E-08	3.29E-07
SCNN1B	-0.51735	1.35E-36	5.12E-35
SCNN1D	-0.1983	5.78E-06	1.60E-05
SCNN1G	-0.34309	1.13E-15	8.80E-15
SCO1	0.227762	1.74E-07	5.79E-07
SCO2	-0.00304	9.45E-01	9.57E-01
SCOC	0.16649	1.47E-04	3.35E-04
SCP2	-0.38287	2.00E-19	2.22E-18
SCPEP1	-0.16629	1.50E-04	3.41E-04
SCRG1	-0.17756	5.08E-05	1.23E-04
SCRIB	0.051316	2.45E-01	3.05E-01
SCRN1	-0.03703	4.02E-01	4.68E-01
SCRN2	-0.2284	1.61E-07	5.36E-07

SCRN3	-0.00562	8.99E-01	9.18E-01
SCRT1	-0.0345	4.35E-01	5.01E-01
SCRT2	0.122819	5.25E-03	9.39E-03
SCTR	-0.49262	7.72E-33	2.36E-31
SCT	0.033817	4.44E-01	5.10E-01
SCUBE1	-0.16247	2.13E-04	4.75E-04
SCUBE2	-0.40662	6.32E-22	8.65E-21
SCUBE3	-0.15291	4.98E-04	1.04E-03
SCXB	-0.03829	3.86E-01	4.52E-01
SCYL1	0.006568	8.82E-01	9.04E-01
SCYL2	0.345049	7.61E-16	6.05E-15
SCYL3	-0.27289	3.02E-10	1.38E-09
SDAD1	0.254874	4.43E-09	1.76E-08
SDC1	-0.22956	1.38E-07	4.66E-07
SDC2	-0.10211	2.05E-02	3.27E-02
SDC3	-0.11147	1.14E-02	1.91E-02
SDC4P	-0.11707	7.83E-03	1.35E-02
SDC4	-0.20557	2.55E-06	7.35E-06
SDCBP2	0.023017	6.02E-01	6.60E-01
SDCBP	0.070595	1.10E-01	1.49E-01
SDCCAG1	-0.02747	5.34E-01	5.97E-01
SDCCAG3	0.186697	2.01E-05	5.16E-05
SDCCAG8	-0.37343	1.74E-18	1.77E-17
SDF2L1	0.029613	5.03E-01	5.67E-01
SDF2	-0.06325	1.52E-01	2.00E-01
SDF4	-0.07311	9.75E-02	1.34E-01
SDHAF1	0.061181	1.66E-01	2.16E-01
SDHAF2	0.140456	1.40E-03	2.74E-03
SDHAP1	-0.01458	7.41E-01	7.85E-01
SDHAP2	0.002499	9.55E-01	9.64E-01
SDHAP3	-0.29006	1.93E-11	9.87E-11
SDHA	0.145534	9.25E-04	1.87E-03
SDHB	0.238269	4.42E-08	1.57E-07
SDHC	0.012473	7.78E-01	8.15E-01
SDHD	0.087448	4.73E-02	7.01E-02
SDK1	-0.24069	3.20E-08	1.15E-07
SDK2	-0.22223	3.49E-07	1.12E-06
SDPR	-0.45078	3.85E-27	8.03E-26
SDR16C5	-0.23028	1.26E-07	4.26E-07
SDR39U1	-0.09873	2.51E-02	3.93E-02
SDR42E1	-0.18024	3.89E-05	9.59E-05
SDR9C7	0.099912	2.34E-02	3.69E-02
SDSL	0.128852	3.40E-03	6.27E-03
SDS	0.293109	1.16E-11	6.07E-11

SEBOX	-0.13085	2.93E-03	5.46E-03
SEC11A	0.034111	4.40E-01	5.06E-01
SEC11C	-0.07446	9.14E-02	1.27E-01
SEC13	0.155241	4.06E-04	8.65E-04
SEC14L1	0.019934	6.52E-01	7.05E-01
SEC14L2	0.143306	1.11E-03	2.21E-03
SEC14L3	-0.41518	7.06E-23	1.04E-21
SEC14L4	-0.31437	2.82E-13	1.75E-12
SEC14L5	-0.21853	5.51E-07	1.73E-06
SEC16A	-0.10836	1.39E-02	2.30E-02
SEC16B	-0.11214	1.09E-02	1.84E-02
SEC1	-0.18972	1.46E-05	3.82E-05
SEC22A	0.280167	9.63E-11	4.58E-10
SEC22B	0.210708	1.40E-06	4.18E-06
SEC22C	-0.17062	9.98E-05	2.33E-04
SEC23A	0.445514	1.77E-26	3.50E-25
SEC23B	0.005367	9.03E-01	9.22E-01
SEC23IP	0.076387	8.33E-02	1.17E-01
SEC24A	0.177973	4.88E-05	1.19E-04
SEC24B	-0.03001	4.97E-01	5.62E-01
SEC24C	-0.11674	8.01E-03	1.38E-02
SEC24D	0.132072	2.67E-03	5.01E-03
SEC31A	-0.16325	1.99E-04	4.44E-04
SEC31B	-0.31561	2.25E-13	1.41E-12
SEC61A1	0.282055	7.12E-11	3.43E-10
SEC61A2	0.228473	1.59E-07	5.31E-07
SEC61B	0.14168	1.27E-03	2.50E-03
SEC61G	0.405964	7.45E-22	1.01E-20
SEC62	-0.08474	5.46E-02	7.99E-02
SEC63	-0.14107	1.33E-03	2.62E-03
SECISBP2L	-0.41594	5.80E-23	8.65E-22
SECISBP2	-0.34296	1.16E-15	9.03E-15
SECTM1	0.165143	1.67E-04	3.76E-04
SEH1L	0.330146	1.46E-14	1.02E-13
SEL1L2	-0.10194	2.07E-02	3.30E-02
SEL1L3	-0.1865	2.05E-05	5.26E-05
SEL1L	0.053893	2.22E-01	2.80E-01
SELENBP1	-0.64952	4.90E-63	4.60E-61
SELE	-0.11262	1.05E-02	1.78E-02
SELK	-1.21E-05	1.00E+00	1.00E+00
SELL	-0.05398	2.21E-01	2.79E-01
SELM	-0.02526	5.67E-01	6.27E-01
SELO	-0.18687	1.97E-05	5.07E-05
SELPLG	-0.19382	9.43E-06	2.53E-05

SELP		-0.36587	9.33E-18	8.89E-17
SELS		0.08031	6.86E-02	9.85E-02
SELT		0.224316	2.69E-07	8.77E-07
SELV		0.292277	1.33E-11	6.93E-11
SEMA3A		0.37323	1.82E-18	1.85E-17
SEMA3B		-0.24998	8.89E-09	3.41E-08
SEMA3C		0.137942	1.70E-03	3.30E-03
SEMA3D		-0.05155	2.43E-01	3.03E-01
SEMA3E		-0.13628	1.94E-03	3.72E-03
SEMA3F		0.102099	2.05E-02	3.27E-02
SEMA3G		-0.28847	2.51E-11	1.27E-10
SEMA4A		-0.49048	1.58E-32	4.73E-31
SEMA4B		0.258789	2.52E-09	1.03E-08
SEMA4C		0.031735	4.72E-01	5.38E-01
SEMA4D		-0.10659	1.55E-02	2.54E-02
SEMA4F		0.081391	6.49E-02	9.37E-02
SEMA4G		-0.10324	1.91E-02	3.07E-02
SEMA5A		-0.30002	3.58E-12	1.99E-11
SEMA5B		0.236928	5.29E-08	1.86E-07
SEMA6A		-0.13557	2.05E-03	3.92E-03
SEMA6B		0.127285	3.81E-03	6.97E-03
SEMA6C		-0.08976	4.17E-02	6.26E-02
SEMA6D		-0.34351	1.04E-15	8.12E-15
SEMA7A		0.21804	5.84E-07	1.82E-06
SEMG1		0.043791	3.21E-01	3.86E-01
SEMG2		0.009803	8.24E-01	8.55E-01
SENP1		0.514188	4.25E-36	1.56E-34
SENP2		0.342572	1.26E-15	9.73E-15
SENP3		0.219084	5.14E-07	1.62E-06
SENP5		0.348327	3.89E-16	3.17E-15
SENP6		-0.09766	2.67E-02	4.16E-02
SENP7		-0.21863	5.44E-07	1.70E-06
SENP8		-0.28233	6.81E-11	3.29E-10
	15-Sep	0.155904	3.84E-04	8.20E-04
SEPHS1		0.322598	6.16E-14	4.07E-13
SEPHS2		0.166262	1.50E-04	3.42E-04
SEPN1		-0.25439	4.75E-09	1.88E-08
SEPP1		-0.36143	2.45E-17	2.26E-16
SEPSECS		-0.25155	7.13E-09	2.77E-08
	10-Sep	-0.08477	5.46E-02	7.98E-02
	11-Sep	0.276692	1.67E-10	7.77E-10
	12-Sep	0.052845	2.31E-01	2.90E-01
	14-Sep	0.221822	3.67E-07	1.18E-06
	1-Sep	-0.14172	1.26E-03	2.50E-03

	2-Sep	0.22005	4.57E-07	1.45E-06
	3-Sep	0.271958	3.49E-10	1.57E-09
	4-Sep	-0.49837	1.10E-33	3.51E-32
	5-Sep	0.053673	2.24E-01	2.82E-01
	6-Sep	-0.02641	5.50E-01	6.12E-01
SEPT7L		-0.01512	7.32E-01	7.77E-01
SEPT7P2		-0.15433	4.40E-04	9.32E-04
	7-Sep	0.28369	5.47E-11	2.66E-10
	8-Sep	0.076869	8.14E-02	1.15E-01
	9-Sep	0.12919	3.31E-03	6.13E-03
SEPW1		-0.40025	3.10E-21	4.02E-20
SEPX1		0.136567	1.89E-03	3.64E-03
SERAC1		0.029837	4.99E-01	5.64E-01
SERBP1		0.369171	4.50E-18	4.41E-17
SERF1A		0.123022	5.18E-03	9.27E-03
SERF2		-0.05412	2.20E-01	2.77E-01
SERGEF		-0.2468	1.39E-08	5.19E-08
SERHL2		-0.22364	2.93E-07	9.50E-07
SERHL		-0.066	1.35E-01	1.80E-01
SERINC1		-0.2345	7.29E-08	2.52E-07
SERINC2		-0.09471	3.16E-02	4.86E-02
SERINC3		0.039816	3.67E-01	4.33E-01
SERINC4		-0.14066	1.37E-03	2.70E-03
SERINC5		-0.19008	1.41E-05	3.69E-05
SERP1		-0.09857	2.53E-02	3.97E-02
SERP2		-0.28979	2.02E-11	1.03E-10
SERPINA10		0.006863	8.77E-01	9.00E-01
SERPINA11		0.002487	9.55E-01	9.64E-01
SERPINA12		0.022194	6.15E-01	6.72E-01
SERPINA13		0.049311	2.64E-01	3.25E-01
SERPINA1		-0.19416	9.09E-06	2.45E-05
SERPINA3		0.00632	8.86E-01	9.08E-01
SERPINA4		0.036038	4.14E-01	4.81E-01
SERPINA5		0.157758	3.26E-04	7.04E-04
SERPINA6		-0.1091	1.32E-02	2.20E-02
SERPINA7		-0.19071	1.32E-05	3.47E-05
SERPINA9		-0.14223	1.21E-03	2.40E-03
SERPINB10		-0.27696	1.60E-10	7.47E-10
SERPINB11		-0.06504	1.41E-01	1.87E-01
SERPINB12		0.110584	1.20E-02	2.02E-02
SERPINB13		-0.00405	9.27E-01	9.42E-01
SERPINB1		0.039398	3.72E-01	4.39E-01
SERPINB2		-0.01611	7.15E-01	7.62E-01
SERPINB3		0.159347	2.83E-04	6.17E-04

SERPINB4	0.216694	6.88E-07	2.13E-06
SERPINB5	0.313399	3.36E-13	2.07E-12
SERPINB6	-0.1821	3.22E-05	8.03E-05
SERPINB7	0.207632	2.01E-06	5.87E-06
SERPINB8	-0.00442	9.20E-01	9.36E-01
SERPINB9	0.160109	2.64E-04	5.79E-04
SERPINC1	-0.22439	2.67E-07	8.70E-07
SERPIND1	-0.25801	2.82E-09	1.14E-08
SERPINE1	0.22853	1.58E-07	5.27E-07
SERPINE2	0.20485	2.77E-06	7.95E-06
SERPINE3	0.072637	9.96E-02	1.37E-01
SERPINF1	-0.06804	1.23E-01	1.66E-01
SERPINF2	-0.2554	4.11E-09	1.64E-08
SERPING1	-0.12989	3.15E-03	5.84E-03
SERPINH1	0.148959	6.96E-04	1.43E-03
SERPINI1	0.080727	6.72E-02	9.66E-02
SERPINI2	-0.19675	6.85E-06	1.87E-05
SERTAD1	-0.0505	2.53E-01	3.13E-01
SERTAD2	0.085128	5.35E-02	7.84E-02
SERTAD3	-0.00723	8.70E-01	8.94E-01
SERTAD4	-0.21729	6.40E-07	1.99E-06
SESN1	-0.4063	6.85E-22	9.34E-21
SESN2	-0.1587	3.00E-04	6.52E-04
SESN3	-0.21896	5.23E-07	1.64E-06
SESTD1	-0.18131	3.49E-05	8.67E-05
SETBP1	-0.38373	1.64E-19	1.84E-18
SETD1A	-0.02801	5.26E-01	5.89E-01
SETD1B	-0.15817	3.14E-04	6.81E-04
SETD2	-0.22307	3.14E-07	1.02E-06
SETD3	-0.07517	8.84E-02	1.23E-01
SETD4	-0.3983	5.01E-21	6.42E-20
SETD5	0.001055	9.81E-01	9.85E-01
SETD6	-0.12134	5.83E-03	1.03E-02
SETD7	0.039373	3.73E-01	4.39E-01
SETD8	0.324678	4.16E-14	2.79E-13
SETDB1	-0.02648	5.49E-01	6.11E-01
SETDB2	-0.45687	6.37E-28	1.41E-26
SETMAR	-0.15326	4.83E-04	1.01E-03
SETX	-0.14704	8.17E-04	1.66E-03
SET	0.398807	4.42E-21	5.68E-20
SEZ6L2	-0.09906	2.46E-02	3.86E-02
SEZ6L	-0.09534	3.05E-02	4.71E-02
SEZ6	0.057643	1.92E-01	2.45E-01
SF1	-0.05969	1.76E-01	2.28E-01

SF3A1	-0.1105	1.21E-02	2.02E-02
SF3A2	0.154791	4.23E-04	8.97E-04
SF3A3	0.281357	7.96E-11	3.82E-10
SF3B14	0.436896	2.03E-25	3.72E-24
SF3B1	0.020418	6.44E-01	6.98E-01
SF3B2	0.26296	1.36E-09	5.73E-09
SF3B3	0.19842	5.70E-06	1.58E-05
SF3B4	0.244754	1.84E-08	6.80E-08
SF3B5	0.139127	1.55E-03	3.02E-03
SF4	-0.12538	4.38E-03	7.94E-03
SFI1	-0.24742	1.27E-08	4.78E-08
SFMBT1	0.088318	4.51E-02	6.72E-02
SFMBT2	0.069827	1.13E-01	1.54E-01
SFN	0.115424	8.75E-03	1.50E-02
SFPQ	0.382995	1.94E-19	2.16E-18
SFRP1	-0.03097	4.83E-01	5.48E-01
SFRP2	0.217471	6.26E-07	1.95E-06
SFRP4	-0.13415	2.28E-03	4.32E-03
SFRP5	-0.1296	3.22E-03	5.96E-03
SFRS11	-0.0815	6.46E-02	9.32E-02
SFRS12IP1	-0.08085	6.68E-02	9.61E-02
SFRS12	-0.21349	1.01E-06	3.06E-06
SFRS13A	0.136125	1.96E-03	3.76E-03
SFRS13B	0.315033	2.50E-13	1.56E-12
SFRS14	-0.06458	1.43E-01	1.90E-01
SFRS15	-0.03553	4.21E-01	4.87E-01
SFRS16	-0.03848	3.83E-01	4.50E-01
SFRS17A	-0.18835	1.69E-05	4.38E-05
SFRS18	-0.26886	5.61E-10	2.47E-09
SFRS1	0.357159	6.12E-17	5.43E-16
SFRS2B	-0.41685	4.57E-23	6.88E-22
SFRS2IP	0.031621	4.74E-01	5.40E-01
SFRS2	0.318534	1.31E-13	8.40E-13
SFRS3	0.286079	3.71E-11	1.84E-10
SFRS4	-0.11763	7.53E-03	1.31E-02
SFRS5	-0.4075	5.06E-22	6.97E-21
SFRS6	-0.19164	1.19E-05	3.16E-05
SFRS7	0.316383	1.95E-13	1.23E-12
SFRS8	0.04834	2.74E-01	3.35E-01
SFRS9	0.511152	1.26E-35	4.51E-34
SFT2D1	0.089534	4.23E-02	6.33E-02
SFT2D2	0.040776	3.56E-01	4.22E-01
SFT2D3	-0.19404	9.21E-06	2.48E-05
SFTA1P	-0.58164	6.00E-48	3.88E-46

SFTA2	-0.4739	3.43E-30	8.94E-29
SFTA3	-0.49375	5.27E-33	1.62E-31
SFTPA1	-0.35603	7.77E-17	6.80E-16
SFTPA2	-0.3453	7.24E-16	5.76E-15
SFTPB	-0.5318	6.16E-39	2.68E-37
SFTPC	-0.34098	1.73E-15	1.32E-14
SFTPD	-0.46594	4.08E-29	9.89E-28
SFXN1	0.523957	1.19E-37	4.70E-36
SFXN2	-0.22124	3.94E-07	1.26E-06
SFXN3	-0.22214	3.53E-07	1.13E-06
SFXN4	0.251092	7.60E-09	2.94E-08
SFXN5	0.232499	9.46E-08	3.24E-07
SGCA	-0.45983	2.63E-28	5.97E-27
SGCB	0.009395	8.32E-01	8.61E-01
SGCD	-0.24167	2.80E-08	1.01E-07
SGCE	0.12043	6.21E-03	1.10E-02
SGCG	-0.26797	6.42E-10	2.81E-09
SGCZ	0.101124	2.17E-02	3.45E-02
SGEF	-0.15545	3.99E-04	8.51E-04
SGIP1	-0.16339	1.96E-04	4.38E-04
SGK196	0.106358	1.58E-02	2.58E-02
SGK1	-0.1947	8.58E-06	2.32E-05
SGK223	-0.24046	3.30E-08	1.19E-07
SGK269	-0.32685	2.76E-14	1.88E-13
SGK2	-0.00272	9.51E-01	9.61E-01
SGK3	-0.06976	1.14E-01	1.55E-01
SGK494	-0.04561	3.02E-01	3.65E-01
SGMS1	-0.1001	2.31E-02	3.65E-02
SGMS2	-0.31418	2.92E-13	1.81E-12
SGOL1	0.887019	2.70E-174	5.39E-171
SGOL2	0.784276	1.98E-108	3.66E-106
SGPL1	0.248508	1.09E-08	4.14E-08
SGPP1	-0.04086	3.55E-01	4.21E-01
SGPP2	-0.16632	1.50E-04	3.40E-04
SGSH	-0.38718	7.28E-20	8.38E-19
SGSM1	-0.30259	2.29E-12	1.30E-11
SGSM2	-0.39893	4.29E-21	5.52E-20
SGSM3	-0.32344	5.26E-14	3.50E-13
SGTA	0.20878	1.76E-06	5.18E-06
SGTB	0.190586	1.33E-05	3.51E-05
SH2B1	-0.3787	5.24E-19	5.58E-18
SH2B2	0.197088	6.61E-06	1.81E-05
SH2B3	-0.05817	1.88E-01	2.41E-01
SH2D1A	-0.00158	9.72E-01	9.78E-01

SH2D1B	0.112182	1.08E-02	1.83E-02
SH2D2A	0.300151	3.50E-12	1.95E-11
SH2D3A	-0.05706	1.96E-01	2.51E-01
SH2D3C	-0.35945	3.75E-17	3.42E-16
SH2D4A	-0.25393	5.07E-09	2.00E-08
SH2D4B	-0.07323	9.69E-02	1.34E-01
SH2D5	0.416548	4.95E-23	7.42E-22
SH2D6	-0.10397	1.83E-02	2.95E-02
SH2D7	-0.00297	9.46E-01	9.58E-01
SH3BGRL2	-0.38891	4.83E-20	5.66E-19
SH3BGRL3	0.131914	2.70E-03	5.07E-03
SH3BGRL	-0.25421	4.88E-09	1.93E-08
SH3BGR	-0.20712	2.13E-06	6.21E-06
SH3BP1	0.31717	1.69E-13	1.07E-12
SH3BP2	0.128458	3.50E-03	6.44E-03
SH3BP4	-0.10719	1.50E-02	2.46E-02
SH3BP5L	-0.15372	4.64E-04	9.78E-04
SH3BP5	-0.30474	1.58E-12	9.09E-12
SH3D19	-0.22705	1.91E-07	6.31E-07
SH3D20	-0.12391	4.86E-03	8.75E-03
SH3GL1	0.302141	2.48E-12	1.40E-11
SH3GL2	-0.00354	9.36E-01	9.50E-01
SH3GL3	-0.12689	3.92E-03	7.17E-03
SH3GLB1	0.125616	4.30E-03	7.81E-03
SH3GLB2	-0.16687	1.42E-04	3.24E-04
SH3KBP1	0.043921	3.20E-01	3.85E-01
SH3PXD2A	-0.15884	2.96E-04	6.44E-04
SH3PXD2B	0.21328	1.04E-06	3.13E-06
SH3RF1	-0.29697	6.04E-12	3.27E-11
SH3RF2	-0.0587	1.83E-01	2.36E-01
SH3RF3	-0.18429	2.57E-05	6.51E-05
SH3TC1	-0.21571	7.75E-07	2.38E-06
SH3TC2	0.077167	8.02E-02	1.13E-01
SH3YL1	-0.22213	3.54E-07	1.14E-06
SHANK1	-0.00444	9.20E-01	9.36E-01
SHANK2	-0.32634	3.03E-14	2.06E-13
SHANK3	-0.24755	1.25E-08	4.70E-08
SHARPIN	-0.09094	3.91E-02	5.90E-02
SHBG	-0.07	1.13E-01	1.53E-01
SHB	-0.06807	1.23E-01	1.66E-01
SHC1	0.076058	8.46E-02	1.19E-01
SHC2	-0.23035	1.25E-07	4.23E-07
SHC3	-0.27448	2.36E-10	1.08E-09
SHC4	-0.03275	4.58E-01	5.25E-01

SHCBP1	0.826768	2.88E-130	8.33E-128
SHD	0.200381	4.59E-06	1.29E-05
SHE	-0.49512	3.32E-33	1.03E-31
SHFM1	0.356327	7.30E-17	6.41E-16
SHF	-0.17217	8.60E-05	2.02E-04
SHH	-0.48436	1.19E-31	3.39E-30
SHISA2	-0.24033	3.36E-08	1.21E-07
SHISA3	-0.23487	6.94E-08	2.41E-07
SHISA4	-0.18004	3.97E-05	9.78E-05
SHISA5	0.027426	5.35E-01	5.98E-01
SHISA6	-0.28991	1.98E-11	1.01E-10
SHISA7	0.113534	9.92E-03	1.68E-02
SHISA9	0.038459	3.84E-01	4.50E-01
SHKBP1	0.218082	5.81E-07	1.82E-06
SHMT1	-0.11344	9.98E-03	1.69E-02
SHMT2	0.477633	1.05E-30	2.82E-29
SHOC2	-0.07091	1.08E-01	1.47E-01
SHOX2	0.477825	9.83E-31	2.66E-29
SHOX	-0.01157	7.93E-01	8.29E-01
SHPK	-0.18239	3.13E-05	7.82E-05
SHPRH	-0.03439	4.36E-01	5.03E-01
SHQ1	0.113087	1.02E-02	1.73E-02
SHROOM1	-0.13386	2.33E-03	4.42E-03
SHROOM2	-0.16248	2.13E-04	4.74E-04
SHROOM3	-0.35349	1.33E-16	1.13E-15
SHROOM4	-0.46814	2.07E-29	5.14E-28
SIAE	-0.42673	3.32E-24	5.52E-23
SIAH1	0.013487	7.60E-01	8.00E-01
SIAH2	0.240524	3.27E-08	1.18E-07
SIAH3	-0.20402	3.04E-06	8.70E-06
SIDT1	-0.107	1.51E-02	2.49E-02
SIDT2	-0.44585	1.61E-26	3.19E-25
SIGIRR	-0.34028	2.00E-15	1.51E-14
SIGLEC10	0.121822	5.64E-03	1.00E-02
SIGLEC11	-0.08146	6.47E-02	9.34E-02
SIGLEC12	0.094831	3.14E-02	4.83E-02
SIGLEC14	-0.00858	8.46E-01	8.74E-01
SIGLEC15	0.137774	1.72E-03	3.34E-03
SIGLEC16	-0.05115	2.47E-01	3.07E-01
SIGLEC1	-0.07653	8.27E-02	1.16E-01
SIGLEC5	-0.02864	5.17E-01	5.81E-01
SIGLEC6	-0.2561	3.71E-09	1.49E-08
SIGLEC7	0.0306	4.88E-01	5.53E-01
SIGLEC8	-0.19377	9.48E-06	2.55E-05

SIGLEC9	-0.00386	9.30E-01	9.45E-01
SIGLECP3	-0.3936	1.57E-20	1.93E-19
SIGMAR1	0.345939	6.35E-16	5.09E-15
SIK1	-0.19007	1.41E-05	3.69E-05
SIK2	-0.13893	1.58E-03	3.06E-03
SIK3	-0.1717	8.99E-05	2.11E-04
SIKE1	0.225015	2.47E-07	8.07E-07
SIL1	-0.26022	2.04E-09	8.41E-09
SILV	0.071839	1.03E-01	1.42E-01
SIM1	0.193531	9.73E-06	2.61E-05
SIM2	0.100515	2.25E-02	3.57E-02
SIN3A	-0.09482	3.14E-02	4.83E-02
SIN3B	-0.15061	6.06E-04	1.26E-03
SIP1	0.390018	3.71E-20	4.40E-19
SIPA1L1	0.020906	6.36E-01	6.91E-01
SIPA1L2	-0.16437	1.79E-04	4.03E-04
SIPA1L3	-0.10981	1.27E-02	2.11E-02
SIPA1	-0.00806	8.55E-01	8.82E-01
SIRPA	0.025535	5.63E-01	6.24E-01
SIRPB1	-0.01298	7.69E-01	8.08E-01
SIRPB2	-0.05005	2.57E-01	3.18E-01
SIRPD	-0.0505	2.53E-01	3.13E-01
SIRPG	0.091888	3.71E-02	5.62E-02
SIRT1	-0.12497	4.51E-03	8.15E-03
SIRT2	-0.13428	2.26E-03	4.29E-03
SIRT3	-0.43844	1.32E-25	2.45E-24
SIRT4	-0.27779	1.40E-10	6.60E-10
SIRT5	-0.09127	3.84E-02	5.80E-02
SIRT6	0.070863	1.08E-01	1.48E-01
SIRT7	0.075449	8.72E-02	1.22E-01
SIT1	-0.07539	8.74E-02	1.22E-01
SIVA1	0.102287	2.02E-02	3.24E-02
SIX1	-0.07727	7.98E-02	1.13E-01
SIX2	0.054584	2.16E-01	2.73E-01
SIX3	0.223528	2.97E-07	9.63E-07
SIX4	0.120486	6.19E-03	1.09E-02
SIX5	-0.16096	2.45E-04	5.39E-04
SIX6	0.08281	6.04E-02	8.76E-02
SI	0.039367	3.73E-01	4.39E-01
SKA1	0.85851	6.53E-151	3.53E-148
SKA2	0.53962	2.97E-40	1.41E-38
SKA3	0.866985	2.73E-157	2.10E-154
SKAP1	0.076474	8.30E-02	1.17E-01
SKAP2	0.252317	6.39E-09	2.50E-08

SKIL	0.215022	8.41E-07	2.57E-06
SKINTL	-0.34163	1.52E-15	1.17E-14
SKIV2L2	0.041221	3.51E-01	4.17E-01
SKIV2L	0.082557	6.12E-02	8.87E-02
SKI	-0.32491	3.98E-14	2.68E-13
SKP1	-0.06829	1.22E-01	1.64E-01
SKP2	0.589909	1.35E-49	9.05E-48
SLA2	0.08564	5.21E-02	7.65E-02
SLAIN1	-0.27324	2.86E-10	1.31E-09
SLAIN2	-0.08725	4.78E-02	7.07E-02
SLAMF1	-0.10038	2.27E-02	3.60E-02
SLAMF6	-0.03752	3.95E-01	4.62E-01
SLAMF7	0.083813	5.73E-02	8.35E-02
SLAMF8	0.103776	1.85E-02	2.98E-02
SLAMF9	0.313191	3.49E-13	2.15E-12
SLA	-0.05893	1.82E-01	2.35E-01
SLBP	0.500925	4.57E-34	1.50E-32
SLC10A1	-0.13931	1.53E-03	2.98E-03
SLC10A2	-0.24972	9.22E-09	3.53E-08
SLC10A3	0.026874	5.43E-01	6.05E-01
SLC10A4	0.100785	2.22E-02	3.52E-02
SLC10A5	-0.18387	2.69E-05	6.78E-05
SLC10A6	-0.04234	3.38E-01	4.03E-01
SLC10A7	-0.07597	8.50E-02	1.19E-01
SLC11A1	-0.011	8.03E-01	8.38E-01
SLC11A2	-0.20997	1.53E-06	4.53E-06
SLC12A1	0.071832	1.03E-01	1.42E-01
SLC12A2	-0.04778	2.79E-01	3.41E-01
SLC12A3	0.025467	5.64E-01	6.24E-01
SLC12A4	-0.36458	1.23E-17	1.17E-16
SLC12A5	0.000235	9.96E-01	9.96E-01
SLC12A6	-0.18466	2.48E-05	6.29E-05
SLC12A7	0.059734	1.76E-01	2.28E-01
SLC12A8	0.355837	8.10E-17	7.07E-16
SLC12A9	-0.1501	6.32E-04	1.31E-03
SLC13A1	-0.00454	9.18E-01	9.35E-01
SLC13A2	-0.21766	6.12E-07	1.91E-06
SLC13A3	-0.13588	2.00E-03	3.83E-03
SLC13A4	-0.14498	9.68E-04	1.95E-03
SLC13A5	0.13986	1.46E-03	2.86E-03
SLC14A1	-0.45219	2.54E-27	5.39E-26
SLC14A2	-0.28183	7.38E-11	3.55E-10
SLC15A1	0.184586	2.50E-05	6.33E-05
SLC15A2	-0.4222	1.11E-23	1.78E-22

SLC15A3	-0.13037	3.04E-03	5.65E-03
SLC15A4	0.309133	7.24E-13	4.32E-12
SLC16A10	0.112174	1.09E-02	1.83E-02
SLC16A11	-0.52483	8.57E-38	3.45E-36
SLC16A12	-0.32042	9.24E-14	6.01E-13
SLC16A13	0.093542	3.38E-02	5.17E-02
SLC16A14	0.124383	4.70E-03	8.48E-03
SLC16A1	0.449539	5.53E-27	1.13E-25
SLC16A2	-0.06039	1.71E-01	2.23E-01
SLC16A3	0.359269	3.90E-17	3.54E-16
SLC16A4	-0.2981	4.98E-12	2.72E-11
SLC16A5	-0.33875	2.71E-15	2.03E-14
SLC16A6	0.104874	1.73E-02	2.80E-02
SLC16A7	-0.2338	7.99E-08	2.76E-07
SLC16A8	0.027496	5.34E-01	5.97E-01
SLC16A9	-0.04799	2.77E-01	3.39E-01
SLC17A1	-0.17255	8.30E-05	1.95E-04
SLC17A2	0.008983	8.39E-01	8.68E-01
SLC17A3	-0.21948	4.90E-07	1.54E-06
SLC17A4	-0.01462	7.41E-01	7.84E-01
SLC17A5	-0.03644	4.09E-01	4.76E-01
SLC17A6	-0.00862	8.45E-01	8.73E-01
SLC17A7	0.101756	2.09E-02	3.33E-02
SLC17A8	-0.14728	8.01E-04	1.63E-03
SLC17A9	-0.1179	7.40E-03	1.29E-02
SLC18A1	-0.09875	2.50E-02	3.93E-02
SLC18A2	-0.30238	2.38E-12	1.35E-11
SLC18A3	0.017273	6.96E-01	7.45E-01
SLC19A1	0.060944	1.67E-01	2.18E-01
SLC19A2	0.038978	3.77E-01	4.44E-01
SLC19A3	-0.19494	8.35E-06	2.26E-05
SLC1A1	-0.14136	1.30E-03	2.56E-03
SLC1A2	-0.29619	6.90E-12	3.70E-11
SLC1A3	0.012504	7.77E-01	8.15E-01
SLC1A4	0.076703	8.20E-02	1.15E-01
SLC1A5	0.160936	2.45E-04	5.40E-04
SLC1A6	0.253344	5.52E-09	2.17E-08
SLC1A7	-0.32663	2.87E-14	1.96E-13
SLC20A1	0.434735	3.71E-25	6.62E-24
SLC20A2	-0.12902	3.36E-03	6.20E-03
SLC22A10	-0.15226	5.26E-04	1.10E-03
SLC22A11	0.021612	6.25E-01	6.80E-01
SLC22A12	-0.0233	5.98E-01	6.55E-01
SLC22A13	0.042725	3.33E-01	3.99E-01

SLC22A14	0.011184	8.00E-01	8.35E-01
SLC22A15	-0.2866	3.41E-11	1.69E-10
SLC22A16	-0.0996	2.38E-02	3.75E-02
SLC22A17	-0.23742	4.95E-08	1.74E-07
SLC22A18AS	0.016701	7.05E-01	7.53E-01
SLC22A18	-0.24379	2.10E-08	7.70E-08
SLC22A1	0.050477	2.53E-01	3.14E-01
SLC22A20	-0.05306	2.29E-01	2.88E-01
SLC22A23	-0.35226	1.72E-16	1.45E-15
SLC22A24	0.025533	5.63E-01	6.24E-01
SLC22A25	-0.00113	9.80E-01	9.84E-01
SLC22A2	0.076898	8.13E-02	1.14E-01
SLC22A3	-0.54625	2.14E-41	1.05E-39
SLC22A4	-0.07696	8.10E-02	1.14E-01
SLC22A5	-0.3107	5.47E-13	3.30E-12
SLC22A6	-0.006	8.92E-01	9.12E-01
SLC22A7	-0.01874	6.71E-01	7.22E-01
SLC22A8	-0.09137	3.82E-02	5.77E-02
SLC22A9	0.001871	9.66E-01	9.74E-01
SLC23A1	-0.09811	2.60E-02	4.06E-02
SLC23A2	-0.10252	2.00E-02	3.20E-02
SLC23A3	-0.12576	4.26E-03	7.74E-03
SLC24A1	-0.18454	2.51E-05	6.36E-05
SLC24A2	0.140414	1.40E-03	2.75E-03
SLC24A3	-0.23583	6.11E-08	2.13E-07
SLC24A4	-0.2931	1.16E-11	6.07E-11
SLC24A5	-0.14782	7.66E-04	1.57E-03
SLC24A6	-0.01191	7.87E-01	8.24E-01
SLC25A10	0.292892	1.20E-11	6.28E-11
SLC25A11	0.021524	6.26E-01	6.82E-01
SLC25A12	0.071654	1.04E-01	1.43E-01
SLC25A13	0.417845	3.52E-23	5.36E-22
SLC25A14	-0.04982	2.59E-01	3.20E-01
SLC25A15	0.224365	2.68E-07	8.72E-07
SLC25A16	-0.25755	3.01E-09	1.22E-08
SLC25A17	0.116252	8.27E-03	1.42E-02
SLC25A18	-0.01839	6.77E-01	7.28E-01
SLC25A19	0.347141	4.96E-16	4.01E-15
SLC25A1	0.062384	1.57E-01	2.06E-01
SLC25A20	-0.21842	5.58E-07	1.75E-06
SLC25A21	0.271554	3.71E-10	1.67E-09
SLC25A22	0.159286	2.84E-04	6.20E-04
SLC25A23	-0.41895	2.63E-23	4.06E-22
SLC25A24	0.200005	4.78E-06	1.34E-05

SLC25A25	-0.01854	6.75E-01	7.26E-01
SLC25A26	-0.18003	3.97E-05	9.79E-05
SLC25A27	-0.37043	3.40E-18	3.37E-17
SLC25A28	-0.1759	5.98E-05	1.44E-04
SLC25A29	-0.27867	1.22E-10	5.76E-10
SLC25A2	-0.08549	5.25E-02	7.71E-02
SLC25A30	-0.3028	2.21E-12	1.26E-11
SLC25A31	0.018012	6.83E-01	7.33E-01
SLC25A32	0.302022	2.53E-12	1.43E-11
SLC25A33	0.208244	1.87E-06	5.49E-06
SLC25A34	-0.2482	1.14E-08	4.31E-08
SLC25A35	-0.21325	1.04E-06	3.14E-06
SLC25A36	-0.12517	4.44E-03	8.04E-03
SLC25A37	-0.01923	6.63E-01	7.16E-01
SLC25A38	-0.31047	5.70E-13	3.43E-12
SLC25A39	0.357364	5.86E-17	5.21E-16
SLC25A3	0.274404	2.39E-10	1.10E-09
SLC25A40	0.355167	9.33E-17	8.11E-16
SLC25A41	-0.10864	1.36E-02	2.26E-02
SLC25A42	-0.42797	2.37E-24	3.99E-23
SLC25A43	0.154973	4.16E-04	8.84E-04
SLC25A44	-0.04813	2.76E-01	3.37E-01
SLC25A45	-0.21173	1.25E-06	3.73E-06
SLC25A46	-0.0153	7.29E-01	7.74E-01
SLC25A4	-0.34949	3.06E-16	2.52E-15
SLC25A5	0.214098	9.40E-07	2.85E-06
SLC25A6	-0.10939	1.30E-02	2.16E-02
SLC26A10	0.141144	1.32E-03	2.60E-03
SLC26A11	-0.14342	1.10E-03	2.19E-03
SLC26A1	-0.14832	7.34E-04	1.51E-03
SLC26A2	0.074153	9.28E-02	1.29E-01
SLC26A3	-0.01365	7.57E-01	7.98E-01
SLC26A4	0.02231	6.13E-01	6.70E-01
SLC26A5	-0.42429	6.39E-24	1.04E-22
SLC26A6	-0.05124	2.46E-01	3.06E-01
SLC26A7	-0.03902	3.77E-01	4.43E-01
SLC26A8	-0.24422	1.98E-08	7.29E-08
SLC26A9	-0.52557	6.49E-38	2.65E-36
SLC27A1	-0.56821	2.29E-45	1.35E-43
SLC27A2	0.177634	5.04E-05	1.23E-04
SLC27A3	-0.46021	2.34E-28	5.35E-27
SLC27A4	0.192289	1.11E-05	2.96E-05
SLC27A5	-0.0128	7.72E-01	8.10E-01
SLC27A6	-0.03239	4.63E-01	5.29E-01

SLC28A1	0.151012	5.85E-04	1.22E-03
SLC28A2	-0.07288	9.85E-02	1.36E-01
SLC28A3	0.022053	6.18E-01	6.74E-01
SLC29A1	-0.21184	1.23E-06	3.68E-06
SLC29A2	0.079869	7.01E-02	1.00E-01
SLC29A3	-0.10912	1.32E-02	2.20E-02
SLC29A4	0.130122	3.09E-03	5.75E-03
SLC2A10	0.083925	5.70E-02	8.31E-02
SLC2A11	-0.3075	9.68E-13	5.70E-12
SLC2A12	0.000625	9.89E-01	9.91E-01
SLC2A13	-0.14736	7.96E-04	1.62E-03
SLC2A14	0.273855	2.60E-10	1.19E-09
SLC2A1	0.6051	9.41E-53	6.79E-51
SLC2A2	0.082712	6.07E-02	8.80E-02
SLC2A3	0.218978	5.21E-07	1.64E-06
SLC2A4RG	-0.00956	8.29E-01	8.59E-01
SLC2A4	-0.1989	5.41E-06	1.50E-05
SLC2A5	0.329116	1.78E-14	1.24E-13
SLC2A6	0.161447	2.34E-04	5.18E-04
SLC2A7	0.155952	3.82E-04	8.17E-04
SLC2A8	-0.01727	6.96E-01	7.45E-01
SLC2A9	-0.06545	1.38E-01	1.84E-01
SLC30A10	0.287311	3.03E-11	1.52E-10
SLC30A1	0.089118	4.32E-02	6.46E-02
SLC30A2	0.031539	4.75E-01	5.41E-01
SLC30A3	-0.17968	4.11E-05	1.01E-04
SLC30A4	0.091376	3.82E-02	5.77E-02
SLC30A5	-0.05561	2.08E-01	2.63E-01
SLC30A6	0.394019	1.42E-20	1.75E-19
SLC30A7	0.196438	7.09E-06	1.94E-05
SLC30A8	-0.1029	1.95E-02	3.13E-02
SLC30A9	-0.04973	2.60E-01	3.21E-01
SLC31A1	0.133595	2.38E-03	4.50E-03
SLC31A2	0.041493	3.47E-01	4.13E-01
SLC32A1	0.097743	2.65E-02	4.14E-02
SLC33A1	0.272714	3.10E-10	1.41E-09
SLC34A1	0.087976	4.60E-02	6.84E-02
SLC34A2	-0.40689	5.90E-22	8.10E-21
SLC34A3	-0.00987	8.23E-01	8.54E-01
SLC35A1	-0.24412	2.01E-08	7.38E-08
SLC35A2	0.245123	1.75E-08	6.47E-08
SLC35A3	0.125199	4.43E-03	8.03E-03
SLC35A4	-0.31018	6.01E-13	3.61E-12
SLC35A5	-0.09235	3.62E-02	5.49E-02

SLC35B1	0.193838	9.41E-06	2.53E-05
SLC35B2	0.188724	1.62E-05	4.23E-05
SLC35B3	-0.01466	7.40E-01	7.84E-01
SLC35B4	0.161103	2.41E-04	5.33E-04
SLC35C1	-0.06774	1.25E-01	1.68E-01
SLC35C2	-0.00987	8.23E-01	8.54E-01
SLC35D1	0.020361	6.45E-01	6.99E-01
SLC35D2	-0.004	9.28E-01	9.43E-01
SLC35D3	0.190908	1.29E-05	3.40E-05
SLC35E1	-0.17311	7.85E-05	1.86E-04
SLC35E2	-0.34773	4.40E-16	3.57E-15
SLC35E3	0.214288	9.19E-07	2.79E-06
SLC35E4	0.104632	1.75E-02	2.84E-02
SLC35F1	0.05917	1.80E-01	2.33E-01
SLC35F2	0.151632	5.55E-04	1.16E-03
SLC35F3	-0.14784	7.64E-04	1.56E-03
SLC35F4	0.061398	1.64E-01	2.14E-01
SLC35F5	0.212134	1.19E-06	3.56E-06
SLC36A1	0.135802	2.01E-03	3.85E-03
SLC36A2	0.025649	5.61E-01	6.22E-01
SLC36A3	-0.10945	1.29E-02	2.15E-02
SLC36A4	0.307713	9.32E-13	5.49E-12
SLC37A1	-0.01133	7.98E-01	8.33E-01
SLC37A2	-0.0745	9.12E-02	1.27E-01
SLC37A3	0.289072	2.27E-11	1.15E-10
SLC37A4	0.01445	7.44E-01	7.87E-01
SLC38A10	-0.29215	1.36E-11	7.07E-11
SLC38A11	0.021041	6.34E-01	6.89E-01
SLC38A1	0.394028	1.42E-20	1.74E-19
SLC38A2	0.230363	1.25E-07	4.22E-07
SLC38A3	0.021895	6.20E-01	6.76E-01
SLC38A4	0.013086	7.67E-01	8.06E-01
SLC38A5	-0.09168	3.75E-02	5.68E-02
SLC38A6	-0.04324	3.27E-01	3.93E-01
SLC38A7	0.335223	5.44E-15	3.98E-14
SLC38A8	0.076557	8.26E-02	1.16E-01
SLC38A9	0.123356	5.06E-03	9.08E-03
SLC39A10	0.159455	2.80E-04	6.12E-04
SLC39A11	0.037291	3.98E-01	4.65E-01
SLC39A12	0.013728	7.56E-01	7.97E-01
SLC39A13	-0.25699	3.27E-09	1.32E-08
SLC39A14	0.392879	1.87E-20	2.27E-19
SLC39A1	0.14044	1.40E-03	2.74E-03
SLC39A2	0.080249	6.88E-02	9.87E-02

SLC39A3	0.066052	1.34E-01	1.79E-01
SLC39A4	-0.0771	8.05E-02	1.13E-01
SLC39A5	0.100018	2.32E-02	3.67E-02
SLC39A6	0.152979	4.94E-04	1.04E-03
SLC39A7	0.015801	7.21E-01	7.67E-01
SLC39A8	-0.31439	2.81E-13	1.75E-12
SLC39A9	0.03069	4.87E-01	5.52E-01
SLC3A1	-0.01048	8.12E-01	8.45E-01
SLC3A2	0.254706	4.54E-09	1.80E-08
SLC40A1	-0.247	1.35E-08	5.05E-08
SLC41A1	-0.51019	1.78E-35	6.29E-34
SLC41A2	0.084321	5.58E-02	8.15E-02
SLC41A3	-0.22331	3.05E-07	9.88E-07
SLC43A1	-0.17207	8.68E-05	2.04E-04
SLC43A2	-0.30092	3.07E-12	1.71E-11
SLC43A3	-0.04426	3.16E-01	3.81E-01
SLC44A1	0.310941	5.24E-13	3.17E-12
SLC44A2	-0.34355	1.03E-15	8.07E-15
SLC44A3	-0.32906	1.80E-14	1.25E-13
SLC44A4	-0.4021	1.96E-21	2.58E-20
SLC44A5	-0.08186	6.34E-02	9.17E-02
SLC45A1	0.127578	3.73E-03	6.84E-03
SLC45A2	0.103276	1.91E-02	3.07E-02
SLC45A3	0.017578	6.91E-01	7.40E-01
SLC45A4	-0.08145	6.48E-02	9.34E-02
SLC46A1	-0.25291	5.87E-09	2.30E-08
SLC46A2	-0.44958	5.45E-27	1.12E-25
SLC46A3	-0.30697	1.06E-12	6.22E-12
SLC47A1	-0.38784	6.23E-20	7.23E-19
SLC47A2	-0.03569	4.19E-01	4.85E-01
SLC48A1	-0.26934	5.21E-10	2.30E-09
SLC4A10	0.153996	4.53E-04	9.57E-04
SLC4A11	0.085932	5.13E-02	7.55E-02
SLC4A1AP	0.447451	1.01E-26	2.05E-25
SLC4A1	-0.02768	5.31E-01	5.94E-01
SLC4A2	-0.06508	1.40E-01	1.86E-01
SLC4A3	-0.0093	8.33E-01	8.63E-01
SLC4A4	-0.34464	8.27E-16	6.55E-15
SLC4A5	-0.07591	8.53E-02	1.20E-01
SLC4A7	0.183599	2.76E-05	6.96E-05
SLC4A8	-0.05951	1.78E-01	2.30E-01
SLC4A9	-0.03686	4.04E-01	4.70E-01
SLC5A10	0.189987	1.42E-05	3.72E-05
SLC5A11	0.224544	2.62E-07	8.54E-07

SLC5A12	0.191862	1.16E-05	3.09E-05
SLC5A1	-0.08701	4.84E-02	7.16E-02
SLC5A2	-0.44275	3.90E-26	7.58E-25
SLC5A3	0.101053	2.18E-02	3.47E-02
SLC5A4	-0.32993	1.53E-14	1.06E-13
SLC5A5	0.101936	2.07E-02	3.30E-02
SLC5A6	0.433634	5.03E-25	8.88E-24
SLC5A7	-0.19913	5.27E-06	1.47E-05
SLC5A8	-0.02724	5.37E-01	6.00E-01
SLC5A9	-0.41822	3.19E-23	4.87E-22
SLC6A10P	0.237601	4.84E-08	1.70E-07
SLC6A11	-0.00081	9.85E-01	9.89E-01
SLC6A12	-0.15458	4.31E-04	9.13E-04
SLC6A13	-0.22899	1.49E-07	5.00E-07
SLC6A14	-0.02133	6.29E-01	6.85E-01
SLC6A15	0.369318	4.35E-18	4.27E-17
SLC6A16	-0.29559	7.63E-12	4.07E-11
SLC6A17	0.349452	3.08E-16	2.54E-15
SLC6A18	-0.22471	2.56E-07	8.37E-07
SLC6A19	0.023393	5.96E-01	6.54E-01
SLC6A1	-0.2663	8.26E-10	3.57E-09
SLC6A20	-0.14408	1.04E-03	2.09E-03
SLC6A2	-0.00591	8.94E-01	9.14E-01
SLC6A3	-0.05455	2.17E-01	2.73E-01
SLC6A4	-0.24074	3.17E-08	1.14E-07
SLC6A5	0.022176	6.16E-01	6.72E-01
SLC6A6	-0.15063	6.05E-04	1.26E-03
SLC6A7	-0.07286	9.86E-02	1.36E-01
SLC6A8	0.250203	8.61E-09	3.31E-08
SLC6A9	-0.18321	2.88E-05	7.23E-05
SLC7A10	-0.23807	4.54E-08	1.61E-07
SLC7A11	0.217494	6.24E-07	1.94E-06
SLC7A13	-0.00618	8.89E-01	9.10E-01
SLC7A14	0.005571	9.00E-01	9.19E-01
SLC7A1	0.357062	6.24E-17	5.53E-16
SLC7A2	-0.11663	8.06E-03	1.39E-02
SLC7A3	0.083061	5.96E-02	8.66E-02
SLC7A4	-0.1763	5.75E-05	1.39E-04
SLC7A5P1	0.148663	7.14E-04	1.47E-03
SLC7A5P2	0.099545	2.39E-02	3.76E-02
SLC7A5	0.450581	4.08E-27	8.49E-26
SLC7A6OS	0.189089	1.56E-05	4.08E-05
SLC7A6	-0.15491	4.18E-04	8.88E-04
SLC7A7	0.077484	7.90E-02	1.12E-01

SLC7A8	-0.39759	5.95E-21	7.54E-20
SLC7A9	0.073044	9.78E-02	1.35E-01
SLC8A1	-0.0542	2.19E-01	2.77E-01
SLC8A2	0.009064	8.37E-01	8.66E-01
SLC8A3	-0.38431	1.43E-19	1.61E-18
SLC9A10	-0.02016	6.48E-01	7.02E-01
SLC9A11	-0.2943	9.50E-12	5.02E-11
SLC9A1	-0.16522	1.66E-04	3.74E-04
SLC9A2	-0.02298	6.03E-01	6.60E-01
SLC9A3R1	0.13971	1.48E-03	2.90E-03
SLC9A3R2	-0.41671	4.74E-23	7.12E-22
SLC9A3	-0.08272	6.07E-02	8.80E-02
SLC9A4	-0.02683	5.44E-01	6.06E-01
SLC9A5	-0.22865	1.55E-07	5.20E-07
SLC9A6	-0.0122	7.82E-01	8.20E-01
SLC9A7	0.268386	6.03E-10	2.64E-09
SLC9A8	-0.27089	4.11E-10	1.84E-09
SLC9A9	-0.22125	3.94E-07	1.26E-06
SLCO1A2	0.079986	6.97E-02	9.99E-02
SLCO1B1	0.187381	1.87E-05	4.83E-05
SLCO1B3	0.01526	7.30E-01	7.75E-01
SLCO1C1	-0.2312	1.12E-07	3.81E-07
SLCO2A1	-0.24934	9.72E-09	3.71E-08
SLCO2B1	-0.15169	5.52E-04	1.15E-03
SLCO3A1	-0.25745	3.06E-09	1.24E-08
SLCO4A1	0.190493	1.35E-05	3.54E-05
SLCO4C1	-0.20791	1.95E-06	5.69E-06
SLCO5A1	0.183057	2.92E-05	7.33E-05
SLCO6A1	0.012195	7.82E-01	8.20E-01
SLED1	-0.03289	4.56E-01	5.23E-01
SLFN11	0.225773	2.24E-07	7.37E-07
SLFN12L	-0.01299	7.69E-01	8.08E-01
SLFN12	-0.1232	5.12E-03	9.17E-03
SLFN13	0.209765	1.57E-06	4.64E-06
SLFN14	-0.23432	7.46E-08	2.58E-07
SLFN5	-0.00736	8.68E-01	8.92E-01
SLFNL1	-0.52398	1.17E-37	4.66E-36
SLIT1	-0.02263	6.08E-01	6.65E-01
SLIT2	-0.30333	2.02E-12	1.15E-11
SLIT3	-0.39323	1.72E-20	2.09E-19
SLITRK1	0.152366	5.21E-04	1.09E-03
SLITRK2	-0.09311	3.46E-02	5.28E-02
SLITRK3	-0.16063	2.52E-04	5.54E-04
SLITRK4	0.155608	3.94E-04	8.40E-04

SLITRK5	0.082352	6.18E-02	8.95E-02
SLITRK6	-0.05708	1.96E-01	2.50E-01
SLK	0.033187	4.52E-01	5.19E-01
SLMAP	0.024135	5.85E-01	6.44E-01
SLMO1	0.217412	6.31E-07	1.96E-06
SLMO2	0.332829	8.70E-15	6.21E-14
SLN	-0.08647	4.99E-02	7.36E-02
SLPI	-0.36557	9.96E-18	9.47E-17
SLTM	-0.17604	5.90E-05	1.42E-04
SLU7	-0.22115	3.99E-07	1.27E-06
SLURP1	0.062132	1.59E-01	2.08E-01
SMAD1	0.038414	3.84E-01	4.51E-01
SMAD2	-0.06024	1.72E-01	2.24E-01
SMAD3	0.039383	3.72E-01	4.39E-01
SMAD4	-0.14964	6.57E-04	1.36E-03
SMAD5OS	-0.10978	1.27E-02	2.11E-02
SMAD5	0.006112	8.90E-01	9.11E-01
SMAD6	-0.51033	1.69E-35	6.00E-34
SMAD7	-0.34211	1.38E-15	1.06E-14
SMAD9	-0.19219	1.12E-05	2.99E-05
SMAGP	0.260517	1.95E-09	8.08E-09
SMAP1	0.137867	1.71E-03	3.32E-03
SMAP2	-0.23892	4.05E-08	1.44E-07
SMARCA1	-0.00625	8.87E-01	9.09E-01
SMARCA2	-0.43268	6.55E-25	1.14E-23
SMARCA4	-0.09681	2.80E-02	4.35E-02
SMARCA5	0.202013	3.82E-06	1.08E-05
SMARCAD1	0.198139	5.88E-06	1.62E-05
SMARCAL1	0.322329	6.48E-14	4.28E-13
SMARCB1	0.258313	2.70E-09	1.10E-08
SMARCC1	0.122801	5.26E-03	9.40E-03
SMARCC2	0.012897	7.70E-01	8.09E-01
SMARCD1	0.265893	8.78E-10	3.78E-09
SMARCD2	0.107821	1.44E-02	2.37E-02
SMARCD3	-0.37156	2.64E-18	2.65E-17
SMARCE1	0.213587	9.99E-07	3.02E-06
SMC1A	0.339064	2.54E-15	1.91E-14
SMC1B	0.267827	6.56E-10	2.87E-09
SMC2	0.637771	3.77E-60	3.26E-58
SMC3	0.321091	8.16E-14	5.34E-13
SMC4	0.49083	1.41E-32	4.22E-31
SMC5	-0.09981	2.35E-02	3.71E-02
SMC6	0.466365	3.58E-29	8.72E-28
SMCHD1	0.213732	9.82E-07	2.97E-06

SMCP	0.072073	1.02E-01	1.40E-01
SMCR5	-0.2199	4.66E-07	1.47E-06
SMCR7L	0.144996	9.67E-04	1.95E-03
SMCR7	-0.32795	2.23E-14	1.54E-13
SMCR8	-0.07903	7.31E-02	1.04E-01
SMEK1	0.018369	6.77E-01	7.28E-01
SMEK2	0.250967	7.73E-09	2.99E-08
SMEK3P	0.174035	7.18E-05	1.71E-04
SMG1	-0.08058	6.77E-02	9.73E-02
SMG5	0.18673	2.00E-05	5.14E-05
SMG6	-0.35579	8.17E-17	7.13E-16
SMG7	0.077214	8.00E-02	1.13E-01
SMN1	0.128397	3.51E-03	6.47E-03
SMN2	0.301882	2.60E-12	1.46E-11
SMNDC1	0.422183	1.12E-23	1.78E-22
SMOC1	-0.03551	4.21E-01	4.87E-01
SMOC2	-0.26014	2.06E-09	8.50E-09
SMOX	0.278991	1.16E-10	5.48E-10
SMO	0.166871	1.42E-04	3.24E-04
SMPD1	-0.31771	1.53E-13	9.74E-13
SMPD2	-0.1147	9.18E-03	1.57E-02
SMPD3	-0.31057	5.60E-13	3.38E-12
SMPD4	0.294595	9.03E-12	4.79E-11
SMPDL3A	0.044397	3.15E-01	3.79E-01
SMPDL3B	-0.28797	2.72E-11	1.37E-10
SMPX	-0.03204	4.68E-01	5.34E-01
SMR3A	-0.13853	1.62E-03	3.15E-03
SMR3B	-0.09054	4.00E-02	6.02E-02
SMS	0.49237	8.40E-33	2.56E-31
SMTNL1	0.202082	3.79E-06	1.07E-05
SMTNL2	-0.18871	1.63E-05	4.23E-05
SMTN	0.11664	8.06E-03	1.39E-02
SMU1	0.205024	2.71E-06	7.80E-06
SMUG1	0.261843	1.61E-09	6.70E-09
SMURF1	-0.06265	1.56E-01	2.04E-01
SMURF2	0.031485	4.76E-01	5.41E-01
SMYD1	0.162468	2.13E-04	4.75E-04
SMYD2	0.03428	4.38E-01	5.04E-01
SMYD3	-0.13942	1.52E-03	2.96E-03
SMYD4	-0.30027	3.43E-12	1.91E-11
SMYD5	0.243263	2.25E-08	8.25E-08
SNAI1	0.368482	5.24E-18	5.08E-17
SNAI2	0.168137	1.26E-04	2.90E-04
SNAI3	-0.29634	6.72E-12	3.61E-11

SNAP23	0.001074	9.81E-01	9.85E-01
SNAP25	0.098679	2.51E-02	3.94E-02
SNAP29	0.011087	8.02E-01	8.37E-01
SNAP47	-0.00702	8.74E-01	8.97E-01
SNAP91	0.116559	8.10E-03	1.40E-02
SNAPC1	0.343381	1.07E-15	8.32E-15
SNAPC2	-0.04191	3.42E-01	4.08E-01
SNAPC3	0.150954	5.88E-04	1.22E-03
SNAPC4	-0.11937	6.69E-03	1.17E-02
SNAPC5	0.162967	2.04E-04	4.55E-04
SNAPIN	0.079504	7.14E-02	1.02E-01
SNAR-B2	0.07559	8.66E-02	1.21E-01
SNAR-G1	0.1398	1.47E-03	2.88E-03
SNCAIP	-0.06464	1.43E-01	1.89E-01
SNCA	-0.03144	4.76E-01	5.42E-01
SNCB	0.168573	1.21E-04	2.79E-04
SNCG	0.196791	6.82E-06	1.87E-05
SND1	0.184046	2.64E-05	6.67E-05
SNED1	-0.59407	1.92E-50	1.31E-48
SNF8	0.473236	4.22E-30	1.09E-28
SNHG10	0.057242	1.95E-01	2.49E-01
SNHG11	-0.07764	7.84E-02	1.11E-01
SNHG12	-0.13285	2.52E-03	4.74E-03
SNHG1	0.272951	2.99E-10	1.36E-09
SNHG3-RCC1	0.097153	2.75E-02	4.27E-02
SNHG3	-0.04425	3.16E-01	3.81E-01
SNHG4	0.087513	4.71E-02	6.99E-02
SNHG5	-0.09141	3.81E-02	5.76E-02
SNHG6	0.181748	3.34E-05	8.30E-05
SNHG7	-0.15476	4.24E-04	8.99E-04
SNHG8	-0.28529	4.22E-11	2.07E-10
SNHG9	-0.23825	4.44E-08	1.57E-07
SNIP1	0.052256	2.36E-01	2.96E-01
SNN	-0.13678	1.86E-03	3.59E-03
SNORA10	0.025508	5.64E-01	6.24E-01
SNORA11D	-0.03701	4.02E-01	4.68E-01
SNORA11E	-0.04534	3.04E-01	3.68E-01
SNORA11	0.057708	1.91E-01	2.45E-01
SNORA12	0.067147	1.28E-01	1.72E-01
SNORA13	-0.02148	6.27E-01	6.83E-01
SNORA14A	0.064336	1.45E-01	1.92E-01
SNORA14B	0.018206	6.80E-01	7.31E-01
SNORA15	0.084857	5.43E-02	7.95E-02
SNORA16A	-0.01755	6.91E-01	7.40E-01

SNORA16B	-0.04279	3.32E-01	3.98E-01
SNORA18	0.050794	2.50E-01	3.10E-01
SNORA1	-0.00518	9.07E-01	9.25E-01
SNORA20	0.082519	6.13E-02	8.88E-02
SNORA21	0.022507	6.10E-01	6.67E-01
SNORA22	-0.00076	9.86E-01	9.89E-01
SNORA23	0.036241	4.12E-01	4.78E-01
SNORA24	-0.11527	8.84E-03	1.51E-02
SNORA25	0.055982	2.05E-01	2.60E-01
SNORA26	0.040762	3.56E-01	4.22E-01
SNORA27	0.029825	4.99E-01	5.64E-01
SNORA28	-0.04233	3.38E-01	4.03E-01
SNORA29	0.017335	6.95E-01	7.44E-01
SNORA2A	0.05265	2.33E-01	2.91E-01
SNORA2B	-0.04911	2.66E-01	3.28E-01
SNORA31	0.058191	1.87E-01	2.41E-01
SNORA32	0.007093	8.72E-01	8.96E-01
SNORA34	-0.01292	7.70E-01	8.09E-01
SNORA36A	-0.00404	9.27E-01	9.42E-01
SNORA37	-0.03341	4.49E-01	5.15E-01
SNORA38B	0.057363	1.94E-01	2.48E-01
SNORA38	-0.05074	2.50E-01	3.11E-01
SNORA39	-0.16671	1.44E-04	3.28E-04
SNORA3	0.002763	9.50E-01	9.61E-01
SNORA40	0.159007	2.91E-04	6.35E-04
SNORA41	0.010351	8.15E-01	8.47E-01
SNORA42	0.007211	8.70E-01	8.94E-01
SNORA44	0.059092	1.81E-01	2.33E-01
SNORA45	-0.069	1.18E-01	1.59E-01
SNORA46	-0.01987	6.53E-01	7.06E-01
SNORA47	-0.01692	7.02E-01	7.50E-01
SNORA48	-0.04793	2.78E-01	3.40E-01
SNORA49	0.005964	8.93E-01	9.13E-01
SNORA4	0.036996	4.02E-01	4.68E-01
SNORA50	0.01132	7.98E-01	8.33E-01
SNORA51	0.11271	1.05E-02	1.77E-02
SNORA52	-0.02073	6.39E-01	6.93E-01
SNORA53	0.086006	5.11E-02	7.52E-02
SNORA54	-0.0009	9.84E-01	9.87E-01
SNORA55	-0.05763	1.92E-01	2.46E-01
SNORA56	0.053373	2.27E-01	2.84E-01
SNORA57	-0.03993	3.66E-01	4.32E-01
SNORA58	0.062177	1.59E-01	2.08E-01
SNORA59B	-0.0185	6.75E-01	7.26E-01

SNORA5A	0.09679	2.81E-02	4.36E-02
SNORA5B	-0.0157	7.22E-01	7.68E-01
SNORA5C	0.022475	6.11E-01	6.68E-01
SNORA61	-0.0007	9.87E-01	9.90E-01
SNORA62	-0.08886	4.38E-02	6.54E-02
SNORA63	0.007978	8.57E-01	8.83E-01
SNORA64	-0.01907	6.66E-01	7.17E-01
SNORA65	0.074909	8.95E-02	1.25E-01
SNORA66	0.011593	7.93E-01	8.29E-01
SNORA67	0.076375	8.34E-02	1.17E-01
SNORA68	0.00449	9.19E-01	9.35E-01
SNORA6	0.058138	1.88E-01	2.41E-01
SNORA70B	-0.03123	4.79E-01	5.45E-01
SNORA70	0.046665	2.91E-01	3.53E-01
SNORA71A	-0.01686	7.03E-01	7.51E-01
SNORA71B	0.018969	6.68E-01	7.19E-01
SNORA71C	0.128431	3.51E-03	6.45E-03
SNORA71D	0.081258	6.54E-02	9.42E-02
SNORA72	-0.07732	7.96E-02	1.12E-01
SNORA74A	0.056557	2.00E-01	2.55E-01
SNORA74B	5.57E-05	9.99E-01	9.99E-01
SNORA75	0.00703	8.74E-01	8.97E-01
SNORA76	0.109234	1.31E-02	2.18E-02
SNORA77	-0.03803	3.89E-01	4.56E-01
SNORA78	0.019504	6.59E-01	7.11E-01
SNORA79	0.032779	4.58E-01	5.24E-01
SNORA7B	-0.15229	5.24E-04	1.10E-03
SNORA80	0.033354	4.50E-01	5.16E-01
SNORA81	0.016057	7.16E-01	7.63E-01
SNORA84	0.036045	4.14E-01	4.81E-01
SNORA8	-0.01107	8.02E-01	8.37E-01
SNORA9	0.009693	8.26E-01	8.57E-01
SNORD10	-0.09737	2.71E-02	4.23E-02
SNORD115-26	-0.04876	2.69E-01	3.31E-01
SNORD116-20	-0.25598	3.78E-09	1.51E-08
SNORD116-28	-0.24791	1.19E-08	4.48E-08
SNORD116-4	-0.12753	3.74E-03	6.86E-03
SNORD15A	0.026264	5.52E-01	6.14E-01
SNORD15B	0.050525	2.52E-01	3.13E-01
SNORD17	0.129355	3.27E-03	6.06E-03
SNORD1C	0.051726	2.41E-01	3.01E-01
SNORD22	0.066263	1.33E-01	1.78E-01
SNORD89	0.029869	4.99E-01	5.63E-01
SNORD94	-0.00047	9.91E-01	9.94E-01

SNORD97	-0.11508	8.95E-03	1.53E-02
SNPH	-0.13195	2.70E-03	5.06E-03
SNRK	-0.26739	7.00E-10	3.05E-09
SNRNP200	0.200858	4.35E-06	1.22E-05
SNRNP25	0.24154	2.85E-08	1.03E-07
SNRNP27	0.452719	2.18E-27	4.65E-26
SNRNP35	-0.16784	1.30E-04	2.98E-04
SNRNP40	0.370112	3.65E-18	3.60E-17
SNRNP48	0.139583	1.50E-03	2.92E-03
SNRNP70	-0.14399	1.05E-03	2.10E-03
SNRPA1	0.583098	3.10E-48	2.02E-46
SNRPA	0.333375	7.82E-15	5.60E-14
SNRPB2	0.293238	1.14E-11	5.95E-11
SNRPB	0.42656	3.47E-24	5.77E-23
SNRPC	0.364561	1.24E-17	1.17E-16
SNRPD1	0.579419	1.64E-47	1.05E-45
SNRPD2	0.361699	2.31E-17	2.14E-16
SNRPD3	0.24462	1.87E-08	6.91E-08
SNRPE	0.284691	4.65E-11	2.28E-10
SNRPF	0.51619	2.06E-36	7.68E-35
SNRPG	0.481966	2.59E-31	7.25E-30
SNRPN	-0.26126	1.75E-09	7.28E-09
SNTA1	0.02414	5.85E-01	6.44E-01
SNTB1	-0.29546	7.80E-12	4.15E-11
SNTB2	-0.15082	5.95E-04	1.24E-03
SNTG1	0.064112	1.46E-01	1.93E-01
SNTG2	-0.21288	1.09E-06	3.27E-06
SNTN	-0.41461	8.18E-23	1.20E-21
SNUPN	0.104579	1.76E-02	2.85E-02
SNURF	-0.07432	9.20E-02	1.28E-01
SNW1	0.334049	6.85E-15	4.95E-14
SNX10	0.252472	6.25E-09	2.44E-08
SNX11	0.219258	5.04E-07	1.59E-06
SNX12	0.19529	8.04E-06	2.18E-05
SNX13	-0.08685	4.89E-02	7.22E-02
SNX14	0.004295	9.23E-01	9.38E-01
SNX15	0.108461	1.38E-02	2.28E-02
SNX16	0.139093	1.55E-03	3.03E-03
SNX17	0.224991	2.47E-07	8.09E-07
SNX18	-0.27495	2.19E-10	1.01E-09
SNX19	-0.1331	2.47E-03	4.66E-03
SNX1	-0.48378	1.44E-31	4.08E-30
SNX20	-0.16127	2.38E-04	5.25E-04
SNX21	-0.06573	1.36E-01	1.82E-01

SNX22	-0.26628	8.28E-10	3.58E-09
SNX24	-0.1375	1.76E-03	3.41E-03
SNX25	-0.46663	3.30E-29	8.04E-28
SNX27	-0.01514	7.32E-01	7.76E-01
SNX29	-0.46907	1.55E-29	3.88E-28
SNX2	-0.07389	9.39E-02	1.30E-01
SNX30	-0.53389	2.76E-39	1.23E-37
SNX31	-0.07813	7.65E-02	1.09E-01
SNX32	0.024475	5.79E-01	6.39E-01
SNX33	-0.37387	1.57E-18	1.61E-17
SNX3	0.180555	3.77E-05	9.31E-05
SNX4	0.173305	7.71E-05	1.83E-04
SNX5	-0.06903	1.18E-01	1.59E-01
SNX6	0.372366	2.20E-18	2.23E-17
SNX7	0.302579	2.30E-12	1.31E-11
SNX8	0.316152	2.04E-13	1.28E-12
SNX9	0.140523	1.39E-03	2.73E-03
SOAT1	0.117788	7.45E-03	1.29E-02
SOAT2	-0.10783	1.44E-02	2.37E-02
SOBP	-0.19012	1.40E-05	3.67E-05
SOCS1	-0.05582	2.06E-01	2.62E-01
SOCS2	-0.19646	7.07E-06	1.93E-05
SOCS3	0.076551	8.26E-02	1.16E-01
SOCS4	0.222897	3.21E-07	1.04E-06
SOCS5	-0.14572	9.12E-04	1.84E-03
SOCS6	0.015145	7.32E-01	7.76E-01
SOCS7	0.013308	7.63E-01	8.03E-01
SOD1	0.093134	3.46E-02	5.27E-02
SOD2	0.196812	6.81E-06	1.86E-05
SOD3	-0.35688	6.49E-17	5.73E-16
SOHLH1	0.241819	2.74E-08	9.95E-08
SOHLH2	0.095073	3.10E-02	4.77E-02
SOLH	-0.06646	1.32E-01	1.77E-01
SON	-0.17367	7.44E-05	1.77E-04
SORBS1	-0.38067	3.33E-19	3.61E-18
SORBS2	-0.41626	5.33E-23	7.99E-22
SORBS3	-0.28696	3.21E-11	1.60E-10
SORCS1	-0.32545	3.60E-14	2.43E-13
SORCS2	-0.45874	3.64E-28	8.19E-27
SORCS3	-0.12806	3.60E-03	6.61E-03
SORD	0.17531	6.34E-05	1.52E-04
SORL1	-0.19247	1.09E-05	2.90E-05
SORT1	-0.27764	1.44E-10	6.74E-10
SOS1	0.08754	4.71E-02	6.98E-02

SOS2	-0.13462	2.20E-03	4.18E-03
SOSTDC1	-0.3894	4.31E-20	5.07E-19
SOST	0.104121	1.81E-02	2.93E-02
SOX10	-0.25132	7.36E-09	2.85E-08
SOX11	0.035299	4.24E-01	4.90E-01
SOX12	0.087354	4.76E-02	7.04E-02
SOX13	-0.40197	2.02E-21	2.66E-20
SOX14	-0.15521	4.08E-04	8.67E-04
SOX15	0.162846	2.06E-04	4.60E-04
SOX17	-0.25289	5.89E-09	2.31E-08
SOX18	-0.24726	1.30E-08	4.89E-08
SOX1	-0.18613	2.13E-05	5.45E-05
SOX21	0.201261	4.16E-06	1.17E-05
SOX2OT	0.075163	8.84E-02	1.23E-01
SOX2	0.039904	3.66E-01	4.32E-01
SOX30	0.160294	2.60E-04	5.70E-04
SOX3	-0.07223	1.02E-01	1.40E-01
SOX4	0.118233	7.23E-03	1.26E-02
SOX5	-0.15614	3.76E-04	8.05E-04
SOX6	-0.34413	9.18E-16	7.23E-15
SOX7	-0.21851	5.52E-07	1.73E-06
SOX8	-0.33626	4.44E-15	3.27E-14
SOX9	-0.01954	6.58E-01	7.11E-01
SP100	-0.02796	5.27E-01	5.90E-01
SP110	0.035584	4.20E-01	4.87E-01
SP140L	0.191452	1.22E-05	3.22E-05
SP140	-0.00044	9.92E-01	9.94E-01
SP1	0.023521	5.94E-01	6.52E-01
SP2	-0.13571	2.02E-03	3.88E-03
SP3	0.231246	1.11E-07	3.79E-07
SP4	-0.00164	9.70E-01	9.77E-01
SP5	0.027214	5.38E-01	6.00E-01
SP6	0.35251	1.63E-16	1.38E-15
SP7	-0.00836	8.50E-01	8.77E-01
SP8	0.32053	9.06E-14	5.90E-13
SP9	0.286841	3.28E-11	1.63E-10
SPA17	-0.14387	1.06E-03	2.12E-03
SPACA1	0.014856	7.37E-01	7.81E-01
SPACA3	-0.02661	5.47E-01	6.09E-01
SPACA4	-0.37441	1.39E-18	1.43E-17
SPACA5	-0.15948	2.79E-04	6.11E-04
SPAG11A	0.103292	1.90E-02	3.06E-02
SPAG11B	0.07016	1.12E-01	1.52E-01
SPAG16	0.135486	2.06E-03	3.94E-03

SPAG17	-0.22653	2.04E-07	6.72E-07
SPAG1	0.130451	3.02E-03	5.62E-03
SPAG4	0.297124	5.88E-12	3.19E-11
SPAG5	0.823374	2.60E-128	7.02E-126
SPAG6	-0.18012	3.93E-05	9.70E-05
SPAG7	-0.23049	1.23E-07	4.15E-07
SPAG8	-0.36341	1.59E-17	1.49E-16
SPAG9	-0.24845	1.10E-08	4.17E-08
SPAM1	0.020365	6.45E-01	6.99E-01
SPANXA2	0.194005	9.24E-06	2.49E-05
SPANXB2	0.212518	1.13E-06	3.41E-06
SPANXC	0.235897	6.06E-08	2.11E-07
SPANXE	0.21774	6.06E-07	1.89E-06
SPANXN1	0.004306	9.22E-01	9.38E-01
SPANXN2	0.101091	2.18E-02	3.46E-02
SPANXN3	0.132182	2.65E-03	4.97E-03
SPANXN4	0.087982	4.60E-02	6.83E-02
SPANXN5	0.042642	3.34E-01	4.00E-01
SPARCL1	-0.43846	1.31E-25	2.44E-24
SPARC	0.056309	2.02E-01	2.57E-01
SPAST	0.368684	5.01E-18	4.88E-17
SPATA12	0.064262	1.45E-01	1.92E-01
SPATA13	-0.20733	2.08E-06	6.06E-06
SPATA16	0.053419	2.26E-01	2.84E-01
SPATA17	-0.09721	2.74E-02	4.26E-02
SPATA18	-0.48898	2.60E-32	7.75E-31
SPATA19	-0.04789	2.78E-01	3.40E-01
SPATA1	-0.14133	1.30E-03	2.57E-03
SPATA20	-0.17873	4.52E-05	1.11E-04
SPATA21	0.077399	7.93E-02	1.12E-01
SPATA22	-0.01002	8.21E-01	8.52E-01
SPATA24	-0.17381	7.34E-05	1.75E-04
SPATA2L	-0.17789	4.92E-05	1.20E-04
SPATA2	0.048913	2.68E-01	3.30E-01
SPATA3	0.018692	6.72E-01	7.23E-01
SPATA4	-0.24953	9.47E-09	3.62E-08
SPATA5L1	0.197209	6.52E-06	1.79E-05
SPATA5	0.278409	1.27E-10	6.00E-10
SPATA6	-0.32063	8.90E-14	5.80E-13
SPATA7	-0.16405	1.85E-04	4.14E-04
SPATA8	0.134469	2.23E-03	4.23E-03
SPATA9	-0.10972	1.27E-02	2.12E-02
SPATC1	-0.07396	9.36E-02	1.30E-01
SPATS1	-0.22092	4.11E-07	1.31E-06

SPATS2L	0.295572	7.66E-12	4.08E-11
SPATS2	0.461683	1.50E-28	3.46E-27
SPC24	0.794883	2.16E-113	4.45E-111
SPC25	0.877332	1.06E-165	1.12E-162
SPCS1	-0.0298	5.00E-01	5.64E-01
SPCS2	0.059485	1.78E-01	2.30E-01
SPCS3	0.060324	1.72E-01	2.23E-01
SPDEF	-0.22552	2.31E-07	7.59E-07
SPDYA	0.117327	7.69E-03	1.33E-02
SPDYC	0.106949	1.52E-02	2.49E-02
SPDYE1	-0.09648	2.86E-02	4.43E-02
SPDYE2	-0.02169	6.23E-01	6.79E-01
SPDYE3	0.231396	1.09E-07	3.72E-07
SPDYE4	-0.04086	3.55E-01	4.21E-01
SPDYE5	-0.11935	6.70E-03	1.17E-02
SPDYE6	-0.09419	3.26E-02	5.00E-02
SPDYE7P	-0.11443	9.35E-03	1.59E-02
SPDYE8P	-0.04733	2.84E-01	3.46E-01
SPEF1	-0.31318	3.50E-13	2.15E-12
SPEF2	-0.2819	7.30E-11	3.51E-10
SPEG	-0.02832	5.21E-01	5.85E-01
SPEM1	0.113503	9.94E-03	1.69E-02
SPEN	-0.17557	6.18E-05	1.48E-04
SPERT	0.0292	5.08E-01	5.72E-01
SPESP1	0.031197	4.80E-01	5.45E-01
SPG11	-0.26947	5.11E-10	2.26E-09
SPG20	-0.13425	2.27E-03	4.30E-03
SPG21	-0.04865	2.70E-01	3.32E-01
SPG7	-0.27958	1.06E-10	5.00E-10
SPHAR	-0.45104	3.56E-27	7.46E-26
SPHK1	0.484985	9.70E-32	2.79E-30
SPHK2	-0.14138	1.30E-03	2.56E-03
SPHKAP	-0.11623	8.29E-03	1.43E-02
SPI1	-0.10215	2.04E-02	3.26E-02
SPIB	-0.18305	2.92E-05	7.33E-05
SPIC	0.064495	1.44E-01	1.90E-01
SPIN1	0.051808	2.41E-01	3.00E-01
SPIN2A	0.045975	2.98E-01	3.61E-01
SPIN2B	-0.14436	1.02E-03	2.04E-03
SPIN3	-0.15157	5.58E-04	1.16E-03
SPIN4	0.378456	5.54E-19	5.88E-18
SPINK13	-0.10522	1.69E-02	2.75E-02
SPINK14	-0.11828	7.21E-03	1.26E-02
SPINK1	-0.19701	6.66E-06	1.83E-05

SPINK2	-0.14438	1.02E-03	2.04E-03
SPINK4	-0.12235	5.43E-03	9.69E-03
SPINK5	-0.38725	7.15E-20	8.24E-19
SPINK6	-0.01266	7.74E-01	8.12E-01
SPINK7	-0.08508	5.37E-02	7.86E-02
SPINK8	0.049678	2.60E-01	3.22E-01
SPINK9	-0.13758	1.75E-03	3.38E-03
SPINLW1	-0.21408	9.42E-07	2.86E-06
SPINT1	-0.01443	7.44E-01	7.87E-01
SPINT2	0.101232	2.16E-02	3.43E-02
SPINT3	-0.05913	1.80E-01	2.33E-01
SPINT4	0.021161	6.32E-01	6.87E-01
SPIRE1	-0.12114	5.91E-03	1.05E-02
SPIRE2	-0.20769	2.00E-06	5.84E-06
SPNS1	0.030749	4.86E-01	5.51E-01
SPNS2	-0.20812	1.90E-06	5.57E-06
SPNS3	-0.2532	5.63E-09	2.21E-08
SPN	-0.27053	4.35E-10	1.94E-09
SPO11	0.034255	4.38E-01	5.04E-01
SPOCD1	0.331654	1.09E-14	7.73E-14
SPOCK1	0.32235	6.45E-14	4.26E-13
SPOCK2	-0.21592	7.55E-07	2.32E-06
SPOCK3	0.011864	7.88E-01	8.25E-01
SPON1	-0.24939	9.65E-09	3.69E-08
SPON2	0.006236	8.88E-01	9.09E-01
SPOPL	0.183807	2.71E-05	6.82E-05
SPOP	-0.1067	1.54E-02	2.53E-02
SPP1	0.268392	6.02E-10	2.64E-09
SPP2	-0.10959	1.28E-02	2.14E-02
SPPL2A	0.078449	7.53E-02	1.07E-01
SPPL2B	-0.13048	3.01E-03	5.61E-03
SPPL3	0.09708	2.76E-02	4.29E-02
SPRED1	-0.15769	3.28E-04	7.08E-04
SPRED2	-0.23144	1.09E-07	3.69E-07
SPRED3	0.27128	3.87E-10	1.74E-09
SPRN	-0.11148	1.14E-02	1.91E-02
SPRR1A	0.036137	4.13E-01	4.80E-01
SPRR1B	0.079142	7.27E-02	1.04E-01
SPRR2A	0.113294	1.01E-02	1.71E-02
SPRR2B	0.067642	1.25E-01	1.69E-01
SPRR2C	0.048479	2.72E-01	3.34E-01
SPRR2D	0.1237	4.94E-03	8.88E-03
SPRR2E	0.161523	2.32E-04	5.14E-04
SPRR2F	0.151801	5.47E-04	1.14E-03

SPRR2G	-0.02643	5.50E-01	6.12E-01
SPRR3	0.073268	9.67E-02	1.34E-01
SPRR4	0.063279	1.52E-01	2.00E-01
SPRY1	-0.36169	2.32E-17	2.15E-16
SPRY2	-0.3481	4.08E-16	3.32E-15
SPRY3	0.046205	2.95E-01	3.58E-01
SPRY4	-0.38561	1.05E-19	1.19E-18
SPRYD3	-0.34344	1.06E-15	8.23E-15
SPRYD4	0.093832	3.33E-02	5.09E-02
SPRYD5	-0.18674	2.00E-05	5.14E-05
SPR	-0.1371	1.82E-03	3.50E-03
SPSB1	-0.11536	8.78E-03	1.50E-02
SPSB2	-0.16371	1.91E-04	4.27E-04
SPSB3	-0.38279	2.04E-19	2.26E-18
SPSB4	0.042553	3.35E-01	4.01E-01
SPTA1	0.098243	2.58E-02	4.03E-02
SPTAN1	-0.06565	1.37E-01	1.82E-01
SPTBN1	-0.07772	7.80E-02	1.10E-01
SPTBN2	0.090618	3.98E-02	6.00E-02
SPTBN4	-0.02483	5.74E-01	6.34E-01
SPTBN5	-0.15599	3.81E-04	8.14E-04
SPTB	0.059021	1.81E-01	2.34E-01
SPTLC1	0.165979	1.54E-04	3.50E-04
SPTLC2	-0.18963	1.48E-05	3.86E-05
SPTLC3	-0.37112	2.91E-18	2.91E-17
SPTY2D1	0.156571	3.62E-04	7.77E-04
SPZ1	0.028353	5.21E-01	5.85E-01
SQLE	0.227124	1.89E-07	6.26E-07
SQRDL	0.027381	5.35E-01	5.98E-01
SQSTM1	-0.08606	5.10E-02	7.50E-02
SR140	0.360897	2.75E-17	2.53E-16
SRA1	0.000989	9.82E-01	9.86E-01
SRBD1	0.099335	2.42E-02	3.81E-02
SRCAP	-0.19181	1.17E-05	3.10E-05
SRCIN1	-0.14782	7.65E-04	1.57E-03
SRCRB4D	0.137957	1.70E-03	3.29E-03
SRC	0.054554	2.16E-01	2.73E-01
SRD5A1	0.250355	8.43E-09	3.24E-08
SRD5A2	-0.16538	1.63E-04	3.69E-04
SRD5A3	0.008558	8.46E-01	8.74E-01
SREBF1	-0.11938	6.68E-03	1.17E-02
SREBF2	-0.16798	1.28E-04	2.94E-04
SRFBP1	0.176006	5.92E-05	1.43E-04
SRF	-0.01377	7.55E-01	7.96E-01

SRGAP1	0.290773	1.71E-11	8.80E-11
SRGAP2	-0.00656	8.82E-01	9.04E-01
SRGAP3	-0.31929	1.14E-13	7.36E-13
SRGN	0.139545	1.50E-03	2.93E-03
SRI	-0.0169	7.02E-01	7.50E-01
SRL	-0.41568	6.20E-23	9.21E-22
SRMS	0.046664	2.91E-01	3.53E-01
SRM	0.35894	4.18E-17	3.78E-16
SRP14	-0.03997	3.65E-01	4.32E-01
SRP19	0.168863	1.18E-04	2.72E-04
SRP54	0.242912	2.36E-08	8.63E-08
SRP68	0.240784	3.16E-08	1.14E-07
SRP72	0.380016	3.87E-19	4.17E-18
SRP9	0.117196	7.76E-03	1.34E-02
SRPK1	0.463916	7.59E-29	1.80E-27
SRPK2	0.377883	6.32E-19	6.65E-18
SRPK3	-0.05406	2.21E-01	2.78E-01
SRPRB	0.388674	5.11E-20	5.98E-19
SRPR	-0.01016	8.18E-01	8.50E-01
SRPX2	0.204129	3.01E-06	8.60E-06
SRPX	-0.23804	4.56E-08	1.61E-07
SRRD	0.256784	3.37E-09	1.35E-08
SRRM1	0.059808	1.75E-01	2.27E-01
SRRM2	-0.28692	3.23E-11	1.61E-10
SRRM3	0.104132	1.81E-02	2.93E-02
SRRM4	-0.04303	3.30E-01	3.95E-01
SRRM5	-0.09355	3.38E-02	5.16E-02
SRRT	0.257175	3.18E-09	1.28E-08
SRR	-0.11899	6.86E-03	1.20E-02
SRXN1	0.251979	6.70E-09	2.61E-08
SRY	0.019395	6.61E-01	7.13E-01
SS18L1	-0.00207	9.63E-01	9.71E-01
SS18L2	0.074801	8.99E-02	1.25E-01
SS18	-0.02333	5.97E-01	6.55E-01
SSBP1	0.429727	1.47E-24	2.51E-23
SSBP2	-0.25448	4.69E-09	1.86E-08
SSBP3	-0.15756	3.31E-04	7.16E-04
SSBP4	-0.18056	3.77E-05	9.31E-05
SSB	0.433141	5.77E-25	1.01E-23
SSC5D	-0.34441	8.68E-16	6.85E-15
SSFA2	-0.07305	9.78E-02	1.35E-01
SSH1	0.078818	7.39E-02	1.05E-01
SSH2	-0.00579	8.96E-01	9.16E-01
SSH3	-0.19482	8.46E-06	2.29E-05

SSNA1	0.039491	3.71E-01	4.37E-01
SSPN	-0.07126	1.06E-01	1.45E-01
SSPO	-0.15915	2.88E-04	6.28E-04
SSR1	0.259075	2.41E-09	9.87E-09
SSR2	0.021809	6.21E-01	6.78E-01
SSR3	0.391244	2.77E-20	3.33E-19
SSR4	-0.12411	4.79E-03	8.64E-03
SSRP1	0.443982	2.75E-26	5.39E-25
SSSCA1	0.110366	1.22E-02	2.04E-02
SSTR1	-0.26288	1.38E-09	5.80E-09
SSTR2	0.369934	3.80E-18	3.74E-17
SSTR3	-0.00037	9.93E-01	9.95E-01
SSTR4	-0.20714	2.13E-06	6.20E-06
SSTR5	-0.072	1.03E-01	1.41E-01
SST	0.107898	1.43E-02	2.36E-02
SSU72	-0.04708	2.86E-01	3.49E-01
SSX1	0.153902	4.57E-04	9.64E-04
SSX2IP	0.550505	3.84E-42	1.97E-40
SSX2	0.134867	2.16E-03	4.11E-03
SSX3	0.074249	9.23E-02	1.28E-01
SSX4	0.120558	6.16E-03	1.09E-02
SSX5	0.024123	5.85E-01	6.44E-01
SSX6	0.104234	1.80E-02	2.91E-02
SSX7	0.036409	4.10E-01	4.76E-01
SSX8	0.079824	7.03E-02	1.01E-01
ST13	-0.00545	9.02E-01	9.21E-01
ST14	0.163677	1.91E-04	4.28E-04
ST18	0.101081	2.18E-02	3.46E-02
ST20	0.115162	8.90E-03	1.52E-02
ST3GAL1	-0.2568	3.36E-09	1.35E-08
ST3GAL2	-0.00776	8.61E-01	8.86E-01
ST3GAL3	-0.32883	1.89E-14	1.30E-13
ST3GAL4	0.229414	1.41E-07	4.75E-07
ST3GAL5	-0.50693	5.64E-35	1.92E-33
ST3GAL6	-0.27942	1.08E-10	5.13E-10
ST5	-0.43033	1.25E-24	2.13E-23
ST6GAL1	-0.24864	1.07E-08	4.07E-08
ST6GAL2	-0.17955	4.16E-05	1.02E-04
ST6GALNAC1	-0.32189	7.03E-14	4.63E-13
ST6GALNAC2	-0.14881	7.05E-04	1.45E-03
ST6GALNAC3	0.01696	7.01E-01	7.49E-01
ST6GALNAC4	-0.40063	2.82E-21	3.67E-20
ST6GALNAC5	0.090394	4.03E-02	6.07E-02
ST6GALNAC6	-0.58615	7.67E-49	5.08E-47

ST7L	-0.21031	1.47E-06	4.36E-06
ST7OT1	-0.08376	5.75E-02	8.38E-02
ST7OT2	-0.13212	2.66E-03	5.00E-03
ST7OT3	-0.14868	7.12E-04	1.46E-03
ST7OT4	0.199566	5.02E-06	1.40E-05
ST7	-0.30584	1.30E-12	7.54E-12
ST8SIA1	-0.2015	4.04E-06	1.14E-05
ST8SIA2	0.038486	3.83E-01	4.50E-01
ST8SIA3	-0.01004	8.20E-01	8.52E-01
ST8SIA4	0.027199	5.38E-01	6.01E-01
ST8SIA5	0.032367	4.64E-01	5.29E-01
ST8SIA6	-0.21431	9.16E-07	2.79E-06
STAB1	-0.01967	6.56E-01	7.09E-01
STAB2	-0.16886	1.18E-04	2.72E-04
STAC2	-0.15417	4.46E-04	9.43E-04
STAC3	-0.01126	7.99E-01	8.34E-01
STAC	-0.16548	1.62E-04	3.66E-04
STAG1	0.217359	6.35E-07	1.97E-06
STAG2	0.094629	3.18E-02	4.88E-02
STAG3L1	-0.04095	3.54E-01	4.20E-01
STAG3L2	0.001201	9.78E-01	9.84E-01
STAG3L3	-0.08739	4.75E-02	7.03E-02
STAG3L4	0.163785	1.89E-04	4.24E-04
STAG3	0.124583	4.63E-03	8.37E-03
STAM2	-0.17962	4.14E-05	1.02E-04
STAMBPL1	0.140174	1.43E-03	2.80E-03
STAMBP	0.412797	1.31E-22	1.89E-21
STAM	0.244519	1.90E-08	7.01E-08
STAP1	-0.19523	8.09E-06	2.19E-05
STAP2	-0.06924	1.17E-01	1.58E-01
STARD10	-0.14877	7.07E-04	1.45E-03
STARD13	-0.22745	1.81E-07	6.02E-07
STARD3NL	0.238087	4.53E-08	1.60E-07
STARD3	0.185621	2.24E-05	5.74E-05
STARD4	0.181294	3.50E-05	8.68E-05
STARD5	-0.161	2.44E-04	5.38E-04
STARD6	0.133181	2.46E-03	4.64E-03
STARD7	0.216422	7.11E-07	2.20E-06
STARD8	-0.19666	6.93E-06	1.89E-05
STAR	-0.07205	1.02E-01	1.41E-01
STAT1	0.397811	5.64E-21	7.18E-20
STAT2	0.039784	3.68E-01	4.34E-01
STAT3	-0.25998	2.11E-09	8.70E-09
STAT4	-0.0121	7.84E-01	8.21E-01

STAT5A	-0.13565	2.03E-03	3.89E-03
STAT5B	-0.24311	2.30E-08	8.41E-08
STAT6	-0.40176	2.13E-21	2.80E-20
STATH	-0.02814	5.24E-01	5.88E-01
STAU1	0.186863	1.97E-05	5.08E-05
STAU2	0.050325	2.54E-01	3.15E-01
STBD1	0.06469	1.43E-01	1.89E-01
STC1	0.441765	5.17E-26	9.94E-25
STC2	0.43302	5.96E-25	1.04E-23
STEAP1	0.401991	2.01E-21	2.65E-20
STEAP2	0.223968	2.81E-07	9.14E-07
STEAP3	-0.2394	3.80E-08	1.36E-07
STEAP4	-0.34483	7.95E-16	6.31E-15
STH	-0.00935	8.32E-01	8.62E-01
STIL	0.80771	8.46E-120	1.99E-117
STIM1	-0.21257	1.13E-06	3.39E-06
STIM2	-0.17603	5.91E-05	1.42E-04
STIP1	0.433912	4.66E-25	8.24E-24
STK10	-0.06021	1.72E-01	2.24E-01
STK11IP	-0.06235	1.58E-01	2.07E-01
STK11	-0.09587	2.96E-02	4.57E-02
STK16	-0.1261	4.15E-03	7.56E-03
STK17A	0.091485	3.79E-02	5.74E-02
STK17B	-0.24932	9.75E-09	3.72E-08
STK19	-0.17378	7.36E-05	1.75E-04
STK24	0.176996	5.37E-05	1.30E-04
STK25	0.101804	2.08E-02	3.33E-02
STK31	0.012149	7.83E-01	8.20E-01
STK32A	-0.26076	1.88E-09	7.81E-09
STK32B	-0.25186	6.81E-09	2.65E-08
STK32C	-0.08528	5.31E-02	7.79E-02
STK33	-0.3386	2.79E-15	2.08E-14
STK35	0.103503	1.88E-02	3.03E-02
STK36	-0.18502	2.39E-05	6.07E-05
STK38L	0.350977	2.25E-16	1.88E-15
STK38	0.087255	4.78E-02	7.07E-02
STK39	0.043205	3.28E-01	3.93E-01
STK3	0.02562	5.62E-01	6.22E-01
STK40	-0.10966	1.28E-02	2.13E-02
STK4	0.059477	1.78E-01	2.30E-01
STL	-0.22411	2.76E-07	8.99E-07
STMN1	0.606642	4.40E-53	3.20E-51
STMN2	-0.17834	4.70E-05	1.15E-04
STMN3	0.030408	4.91E-01	5.56E-01

STMN4	-0.00254	9.54E-01	9.64E-01
STOML1	-0.11685	7.95E-03	1.37E-02
STOML2	0.409856	2.78E-22	3.92E-21
STOML3	-0.25619	3.67E-09	1.47E-08
STOM	-0.27164	3.66E-10	1.65E-09
STON1-GTF2A1L	0.07496	8.93E-02	1.24E-01
STON1	0.002003	9.64E-01	9.72E-01
STON2	0.078175	7.63E-02	1.08E-01
STOX1	0.079298	7.22E-02	1.03E-01
STOX2	-0.15724	3.41E-04	7.35E-04
STRA13	0.23437	7.41E-08	2.56E-07
STRA6	0.270155	4.60E-10	2.05E-09
STRA8	0.027218	5.38E-01	6.00E-01
STRADA	-0.11935	6.70E-03	1.17E-02
STRADB	-0.01398	7.52E-01	7.93E-01
STRAP	0.513667	5.13E-36	1.87E-34
STRBP	0.135426	2.07E-03	3.95E-03
STRC	-0.03109	4.81E-01	5.47E-01
STRN3	0.279791	1.02E-10	4.86E-10
STRN4	0.246851	1.38E-08	5.15E-08
STRN	0.316905	1.77E-13	1.12E-12
STS	-0.15868	3.00E-04	6.52E-04
STT3A	0.202933	3.44E-06	9.80E-06
STT3B	0.047783	2.79E-01	3.41E-01
STUB1	-0.18429	2.57E-05	6.51E-05
STX10	-0.15017	6.28E-04	1.30E-03
STX11	-0.10614	1.60E-02	2.61E-02
STX12	-0.20627	2.35E-06	6.81E-06
STX16	-0.09078	3.95E-02	5.95E-02
STX17	-0.17186	8.86E-05	2.08E-04
STX18	0.145172	9.53E-04	1.92E-03
STX19	-0.25174	6.94E-09	2.70E-08
STX1A	0.274551	2.33E-10	1.07E-09
STX1B	0.098951	2.47E-02	3.89E-02
STX2	0.113109	1.02E-02	1.73E-02
STX3	0.024635	5.77E-01	6.36E-01
STX4	-0.06318	1.52E-01	2.00E-01
STX5	-0.04687	2.88E-01	3.51E-01
STX6	-0.20372	3.15E-06	8.99E-06
STX7	-0.0591	1.81E-01	2.33E-01
STX8	0.005577	9.00E-01	9.19E-01
STXBP1	-0.42118	1.46E-23	2.31E-22
STXBP2	-0.13121	2.85E-03	5.33E-03
STXBP3	0.072488	1.00E-01	1.38E-01

STXBP4	-0.1202	6.31E-03	1.11E-02
STXBP5L	0.290737	1.72E-11	8.85E-11
STXBP5	0.242777	2.41E-08	8.78E-08
STXBP6	-0.12044	6.21E-03	1.10E-02
STYK1	0.359596	3.63E-17	3.31E-16
STYXL1	0.161962	2.23E-04	4.96E-04
STYX	0.276436	1.74E-10	8.09E-10
SUB1	0.24837	1.11E-08	4.21E-08
SUCLA2	0.068207	1.22E-01	1.65E-01
SUCLG1	0.137254	1.80E-03	3.47E-03
SUCLG2	-0.20504	2.71E-06	7.80E-06
SUCNR1	0.02388	5.89E-01	6.47E-01
SUDS3	0.224129	2.76E-07	8.96E-07
SUFU	-0.2305	1.23E-07	4.15E-07
SUGT1L1	-0.47526	2.22E-30	5.86E-29
SUGT1P1	0.09362	3.37E-02	5.15E-02
SUGT1	0.236714	5.44E-08	1.91E-07
SULF1	0.356284	7.37E-17	6.46E-16
SULF2	0.166743	1.44E-04	3.27E-04
SULT1A1	-0.37157	2.63E-18	2.64E-17
SULT1A2	-0.34026	2.01E-15	1.51E-14
SULT1A3	-0.014	7.51E-01	7.93E-01
SULT1B1	-0.13446	2.23E-03	4.23E-03
SULT1C2	-0.31101	5.17E-13	3.13E-12
SULT1C3	-0.04044	3.60E-01	4.26E-01
SULT1C4	-0.32384	4.87E-14	3.25E-13
SULT1E1	-0.19449	8.77E-06	2.37E-05
SULT2A1	0.022502	6.10E-01	6.67E-01
SULT2B1	0.202004	3.82E-06	1.08E-05
SULT4A1	0.044215	3.17E-01	3.81E-01
SULT6B1	-0.0119	7.88E-01	8.24E-01
SUMF1	-0.2862	3.64E-11	1.80E-10
SUMF2	-0.18531	2.32E-05	5.91E-05
SUMO1P1	0.013386	7.62E-01	8.02E-01
SUMO1P3	0.235315	6.55E-08	2.28E-07
SUMO1	0.250758	7.96E-09	3.07E-08
SUMO2	0.395001	1.12E-20	1.39E-19
SUMO3	0.128958	3.37E-03	6.22E-03
SUMO4	0.098257	2.58E-02	4.03E-02
SUN1	0.064186	1.46E-01	1.93E-01
SUN2	-0.22532	2.37E-07	7.78E-07
SUN3	0.118655	7.02E-03	1.23E-02
SUOX	-0.39864	4.60E-21	5.90E-20
SUPT16H	0.378554	5.42E-19	5.75E-18

SUPT3H	0.109362	1.30E-02	2.17E-02
SUPT4H1	0.167807	1.30E-04	2.98E-04
SUPT5H	0.085849	5.15E-02	7.58E-02
SUPT6H	-0.05468	2.15E-01	2.72E-01
SUPT7L	0.258927	2.47E-09	1.01E-08
SUPV3L1	0.268724	5.72E-10	2.52E-09
SURF1	-0.12949	3.24E-03	6.00E-03
SURF2	0.028546	5.18E-01	5.82E-01
SURF4	0.040291	3.62E-01	4.28E-01
SURF6	-0.20209	3.79E-06	1.07E-05
SUSD1	-0.04645	2.93E-01	3.56E-01
SUSD2	-0.64366	1.40E-61	1.27E-59
SUSD3	-0.17022	1.04E-04	2.41E-04
SUSD4	-0.38877	5.00E-20	5.85E-19
SUSD5	-0.012	7.86E-01	8.23E-01
SUV39H1	0.618738	9.81E-56	7.60E-54
SUV39H2	0.60714	3.44E-53	2.52E-51
SUV420H1	-0.02747	5.34E-01	5.97E-01
SUV420H2	0.050615	2.52E-01	3.12E-01
SUZ12P	0.111685	1.12E-02	1.89E-02
SUZ12	0.474287	3.03E-30	7.94E-29
SV2A	0.335196	5.47E-15	3.99E-14
SV2B	-0.07584	8.55E-02	1.20E-01
SV2C	-0.03716	4.00E-01	4.66E-01
SVEP1	-0.34329	1.09E-15	8.47E-15
SVIL	-0.07149	1.05E-01	1.44E-01
SVIP	0.002428	9.56E-01	9.65E-01
SVOPL	-0.04658	2.91E-01	3.54E-01
SVOP	0.09477	3.15E-02	4.84E-02
SWAP70	-0.33256	9.17E-15	6.53E-14
SYAP1	0.167551	1.33E-04	3.05E-04
SYBU	-0.42025	1.87E-23	2.92E-22
SYCE1L	-0.04038	3.60E-01	4.26E-01
SYCE1	0.002625	9.53E-01	9.62E-01
SYCE2	0.477328	1.15E-30	3.10E-29
SYCN	-0.04433	3.15E-01	3.80E-01
SYCP1	0.106229	1.59E-02	2.60E-02
SYCP2L	0.030275	4.93E-01	5.58E-01
SYCP2	0.125292	4.40E-03	7.98E-03
SYCP3	-0.1237	4.94E-03	8.88E-03
SYDE1	-0.05819	1.87E-01	2.41E-01
SYDE2	-0.18245	3.11E-05	7.77E-05
SYF2	-0.29124	1.59E-11	8.17E-11
SYK	-0.10344	1.89E-02	3.04E-02

SYMPK	0.111646	1.12E-02	1.89E-02
SYN1	-0.03177	4.72E-01	5.38E-01
SYN2	-0.0433	3.27E-01	3.92E-01
SYN3	-0.16246	2.14E-04	4.75E-04
SYNCRIP	0.307271	1.01E-12	5.92E-12
SYNC	-0.34265	1.24E-15	9.59E-15
SYNE1	-0.54137	1.49E-40	7.10E-39
SYNE2	-0.25484	4.46E-09	1.77E-08
SYNGAP1	-0.15795	3.20E-04	6.93E-04
SYNGR1	-0.12206	5.55E-03	9.88E-03
SYNGR2	-0.09779	2.65E-02	4.13E-02
SYNGR3	0.175941	5.96E-05	1.44E-04
SYNGR4	0.371589	2.62E-18	2.63E-17
SYNJ1	-0.19432	8.93E-06	2.41E-05
SYNJ2BP	-0.17384	7.32E-05	1.74E-04
SYNJ2	0.344184	9.08E-16	7.15E-15
SYNM	-0.14198	1.24E-03	2.45E-03
SYNPO2L	0.003452	9.38E-01	9.51E-01
SYNPO2	-0.30445	1.66E-12	9.53E-12
SYNPO	-0.38088	3.17E-19	3.45E-18
SYNPR	-0.01238	7.79E-01	8.17E-01
SYNRG	-0.0307	4.87E-01	5.52E-01
SYPL1	0.000449	9.92E-01	9.94E-01
SYPL2	-0.08563	5.21E-02	7.66E-02
SYP	-0.17561	6.15E-05	1.48E-04
SYS1-DBNDD2	-0.17483	6.64E-05	1.59E-04
SYS1	-0.16804	1.27E-04	2.93E-04
SYT10	0.124037	4.82E-03	8.68E-03
SYT11	0.034011	4.41E-01	5.08E-01
SYT12	0.126592	4.01E-03	7.31E-03
SYT13	0.163404	1.96E-04	4.38E-04
SYT14L	0.025516	5.63E-01	6.24E-01
SYT14	0.178256	4.74E-05	1.16E-04
SYT15	-0.41567	6.22E-23	9.24E-22
SYT16	0.111543	1.13E-02	1.90E-02
SYT17	-0.1699	1.07E-04	2.48E-04
SYT1	0.25038	8.40E-09	3.23E-08
SYT2	-0.27024	4.54E-10	2.03E-09
SYT3	-0.11614	8.33E-03	1.43E-02
SYT4	-0.00037	9.93E-01	9.95E-01
SYT5	0.251865	6.81E-09	2.65E-08
SYT6	-0.03986	3.67E-01	4.33E-01
SYT7	-0.00872	8.43E-01	8.72E-01
SYT8	-0.20768	2.00E-06	5.84E-06

SYT9	-0.02494	5.72E-01	6.32E-01
SYTL1	-0.34862	3.66E-16	2.99E-15
SYTL2	-0.08959	4.21E-02	6.31E-02
SYTL3	-0.19532	8.01E-06	2.17E-05
SYTL4	0.054256	2.19E-01	2.76E-01
SYTL5	-0.11209	1.09E-02	1.84E-02
SYVN1	-0.23847	4.31E-08	1.53E-07
TAAR1	0.137723	1.73E-03	3.35E-03
TAAR6	0.021031	6.34E-01	6.89E-01
TAAR8	0.111012	1.17E-02	1.96E-02
TAAR9	0.086362	5.01E-02	7.39E-02
TAB1	-0.26431	1.11E-09	4.74E-09
TAB2	0.116145	8.33E-03	1.43E-02
TAB3	-0.08231	6.20E-02	8.97E-02
TAC1	0.16116	2.40E-04	5.31E-04
TAC3	0.14031	1.41E-03	2.77E-03
TAC4	-0.17673	5.52E-05	1.33E-04
TACC1	-0.28716	3.11E-11	1.55E-10
TACC2	-0.25351	5.39E-09	2.12E-08
TACC3	0.776326	6.84E-105	1.19E-102
TACO1	0.439763	9.10E-26	1.71E-24
TACR1	-0.10149	2.12E-02	3.38E-02
TACR2	-0.12191	5.60E-03	9.97E-03
TACR3	0.257044	3.24E-09	1.31E-08
TACSTD2	-0.10315	1.92E-02	3.09E-02
TADA1	0.097122	2.75E-02	4.28E-02
TADA2A	0.273631	2.69E-10	1.23E-09
TADA2B	-0.29588	7.27E-12	3.89E-11
TADA3	-0.06239	1.57E-01	2.06E-01
TAF10	0.125929	4.21E-03	7.65E-03
TAF11	0.304667	1.60E-12	9.20E-12
TAF12	-0.01222	7.82E-01	8.19E-01
TAF13	0.266742	7.73E-10	3.35E-09
TAF15	0.02467	5.76E-01	6.36E-01
TAF1A	0.375988	9.73E-19	1.01E-17
TAF1B	0.274466	2.36E-10	1.09E-09
TAF1C	-0.20857	1.80E-06	5.30E-06
TAF1D	0.113503	9.94E-03	1.69E-02
TAF1L	-0.14615	8.80E-04	1.78E-03
TAF1	-0.19866	5.55E-06	1.54E-05
TAF2	0.369073	4.60E-18	4.50E-17
TAF3	0.020449	6.43E-01	6.98E-01
TAF4B	0.045607	3.02E-01	3.65E-01
TAF4	0.14802	7.53E-04	1.54E-03

TAF5L	0.079696	7.08E-02	1.01E-01
TAF5	0.358513	4.58E-17	4.13E-16
TAF6L	-0.10029	2.28E-02	3.61E-02
TAF6	0.306633	1.13E-12	6.58E-12
TAF7L	0.16582	1.57E-04	3.55E-04
TAF7	-0.02856	5.18E-01	5.82E-01
TAF8	-0.16509	1.68E-04	3.78E-04
TAF9B	-0.03107	4.82E-01	5.47E-01
TAF9	0.265568	9.22E-10	3.96E-09
TAGAP	-0.11583	8.51E-03	1.46E-02
TAGLN2	-0.1965	7.04E-06	1.92E-05
TAGLN3	0.174462	6.89E-05	1.64E-04
TAGLN	-0.06591	1.35E-01	1.80E-01
TAL1	-0.41244	1.43E-22	2.07E-21
TAL2	0.200073	4.75E-06	1.33E-05
TALDO1	0.034616	4.33E-01	5.00E-01
TANC1	-0.3405	1.91E-15	1.45E-14
TANC2	-0.10565	1.65E-02	2.68E-02
TANK	-0.03366	4.46E-01	5.12E-01
TAOK1	0.181854	3.30E-05	8.22E-05
TAOK2	-0.26425	1.12E-09	4.78E-09
TAOK3	-0.09477	3.15E-02	4.84E-02
TAP1	0.337392	3.55E-15	2.63E-14
TAP2	0.269936	4.76E-10	2.12E-09
TAPBPL	-0.02559	5.62E-01	6.23E-01
TAPBP	-0.105	1.71E-02	2.78E-02
TAPT1	-0.54909	6.82E-42	3.43E-40
TARBP1	-0.21437	9.09E-07	2.77E-06
TARBP2	0.238959	4.03E-08	1.43E-07
TARDBP	0.181184	3.53E-05	8.77E-05
TARM1	0.061937	1.60E-01	2.10E-01
TARP	-0.16636	1.49E-04	3.39E-04
TARS2	0.052589	2.34E-01	2.92E-01
TARSL2	-0.27869	1.22E-10	5.74E-10
TARS	0.56589	6.21E-45	3.62E-43
TAS1R1	-0.13806	1.69E-03	3.27E-03
TAS1R2	-0.01411	7.49E-01	7.91E-01
TAS1R3	0.074452	9.14E-02	1.27E-01
TAS2R10	-0.15314	4.87E-04	1.02E-03
TAS2R13	-0.00292	9.47E-01	9.59E-01
TAS2R14	-0.13297	2.50E-03	4.70E-03
TAS2R19	0.109579	1.28E-02	2.14E-02
TAS2R1	0.011707	7.91E-01	8.27E-01
TAS2R20	-0.0332	4.52E-01	5.18E-01

TAS2R30	0.125717	4.27E-03	7.76E-03
TAS2R31	0.120591	6.14E-03	1.09E-02
TAS2R38	-0.09704	2.77E-02	4.30E-02
TAS2R39	0.085655	5.21E-02	7.65E-02
TAS2R3	-0.07451	9.12E-02	1.27E-01
TAS2R40	0.054	2.21E-01	2.79E-01
TAS2R41	-0.04205	3.41E-01	4.07E-01
TAS2R42	-0.00495	9.11E-01	9.28E-01
TAS2R43	0.022639	6.08E-01	6.65E-01
TAS2R46	0.09555	3.02E-02	4.65E-02
TAS2R4	-0.21063	1.42E-06	4.21E-06
TAS2R50	-0.03256	4.61E-01	5.27E-01
TAS2R5	0.006181	8.89E-01	9.10E-01
TAS2R60	0.091502	3.79E-02	5.73E-02
TAS2R7	-0.02914	5.09E-01	5.73E-01
TAS2R8	-0.01842	6.77E-01	7.27E-01
TAS2R9	0.038735	3.80E-01	4.47E-01
TASP1	-0.07897	7.33E-02	1.05E-01
TATDN1	0.167988	1.28E-04	2.94E-04
TATDN2	0.037334	3.98E-01	4.64E-01
TATDN3	-0.09259	3.57E-02	5.42E-02
TAT	-0.0462	2.95E-01	3.58E-01
TAX1BP1	0.088127	4.56E-02	6.79E-02
TAX1BP3	-0.19039	1.36E-05	3.58E-05
TAZ	0.004532	9.18E-01	9.35E-01
TBC1D10A	-0.19369	9.56E-06	2.57E-05
TBC1D10B	0.177563	5.08E-05	1.23E-04
TBC1D10C	-0.15337	4.78E-04	1.01E-03
TBC1D12	-0.20129	4.14E-06	1.17E-05
TBC1D13	-0.1391	1.55E-03	3.03E-03
TBC1D14	-0.12945	3.25E-03	6.02E-03
TBC1D15	0.121041	5.95E-03	1.05E-02
TBC1D16	0.116183	8.31E-03	1.43E-02
TBC1D17	-0.44386	2.84E-26	5.57E-25
TBC1D19	-0.10463	1.75E-02	2.84E-02
TBC1D1	0.260142	2.06E-09	8.50E-09
TBC1D20	-0.25876	2.53E-09	1.03E-08
TBC1D22A	-0.1902	1.39E-05	3.64E-05
TBC1D22B	-0.01005	8.20E-01	8.52E-01
TBC1D23	0.234501	7.28E-08	2.52E-07
TBC1D24	-0.27248	3.22E-10	1.46E-09
TBC1D25	-0.14726	8.02E-04	1.64E-03
TBC1D26	0.01335	7.62E-01	8.02E-01
TBC1D28	0.022511	6.10E-01	6.67E-01

TBC1D29	0.124165	4.78E-03	8.61E-03
TBC1D2B	-0.27388	2.59E-10	1.19E-09
TBC1D2	-0.36925	4.42E-18	4.33E-17
TBC1D3B	-0.05625	2.03E-01	2.58E-01
TBC1D3C	-0.03454	4.34E-01	5.01E-01
TBC1D3G	0.026957	5.42E-01	6.04E-01
TBC1D3H	-0.03831	3.86E-01	4.52E-01
TBC1D3P2	0.05284	2.31E-01	2.90E-01
TBC1D3	0.020767	6.38E-01	6.93E-01
TBC1D4	0.041448	3.48E-01	4.14E-01
TBC1D5	-0.22597	2.18E-07	7.19E-07
TBC1D7	0.395077	1.10E-20	1.37E-19
TBC1D8B	-0.20308	3.38E-06	9.65E-06
TBC1D8	-0.18214	3.21E-05	8.00E-05
TBC1D9B	-0.22708	1.90E-07	6.29E-07
TBC1D9	-0.17573	6.08E-05	1.46E-04
TBCA	0.234812	6.99E-08	2.42E-07
TBCB	0.2581	2.78E-09	1.13E-08
TBCCD1	0.337606	3.40E-15	2.52E-14
TBCC	-0.04788	2.78E-01	3.40E-01
TBCD	-0.18445	2.53E-05	6.41E-05
TBCEL	-0.17274	8.14E-05	1.92E-04
TBCE	0.192073	1.14E-05	3.02E-05
TBCK	-0.25611	3.71E-09	1.49E-08
TBK1	0.329454	1.67E-14	1.16E-13
TBKBP1	0.0182	6.80E-01	7.31E-01
TBL1XR1	0.227442	1.81E-07	6.02E-07
TBL1X	0.062668	1.56E-01	2.04E-01
TBL1Y	-0.14255	1.18E-03	2.34E-03
TBL2	0.157932	3.21E-04	6.94E-04
TBL3	-0.12169	5.69E-03	1.01E-02
TBPL1	0.316414	1.94E-13	1.22E-12
TBPL2	0.050342	2.54E-01	3.15E-01
TBP	0.275497	2.01E-10	9.32E-10
TBR1	0.299123	4.18E-12	2.30E-11
TBRG1	-0.38088	3.17E-19	3.45E-18
TBRG4	0.462585	1.14E-28	2.65E-27
TBX10	0.117453	7.63E-03	1.32E-02
TBX15	-0.01867	6.73E-01	7.24E-01
TBX18	0.110612	1.20E-02	2.01E-02
TBX19	-0.11079	1.19E-02	1.99E-02
TBX1	-0.04804	2.77E-01	3.39E-01
TBX20	0.157982	3.19E-04	6.91E-04
TBX21	0.020269	6.46E-01	7.00E-01

TBX22	-0.04865	2.70E-01	3.32E-01
TBX2	-0.47811	8.99E-31	2.44E-29
TBX3	-0.29143	1.54E-11	7.94E-11
TBX4	-0.50448	1.33E-34	4.47E-33
TBX5	-0.45589	8.52E-28	1.87E-26
TBX6	-0.09845	2.55E-02	3.99E-02
TBXA2R	0.00787	8.59E-01	8.84E-01
TBXAS1	-0.1699	1.07E-04	2.48E-04
TC2N	-0.09161	3.77E-02	5.70E-02
TCAM1P	0.239658	3.67E-08	1.31E-07
TCAP	-0.10247	2.00E-02	3.21E-02
TCEA1	0.137511	1.76E-03	3.40E-03
TCEA2	-0.21782	6.00E-07	1.87E-06
TCEA3	-0.40516	9.11E-22	1.23E-20
TCEAL1	-0.22925	1.44E-07	4.84E-07
TCEAL2	-0.26343	1.27E-09	5.36E-09
TCEAL3	-0.21623	7.28E-07	2.25E-06
TCEAL4	-0.22965	1.37E-07	4.61E-07
TCEAL5	-0.10701	1.51E-02	2.48E-02
TCEAL6	-0.21672	6.86E-07	2.12E-06
TCEAL7	-0.12876	3.42E-03	6.31E-03
TCEAL8	-0.1902	1.39E-05	3.64E-05
TCEANC	-0.27102	4.03E-10	1.81E-09
TCEB1	0.333965	6.97E-15	5.03E-14
TCEB2	-0.03316	4.53E-01	5.19E-01
TCEB3B	0.101198	2.16E-02	3.44E-02
TCEB3C	0.017214	6.97E-01	7.46E-01
TCEB3	-0.30059	3.25E-12	1.81E-11
TCERG1L	-0.02811	5.24E-01	5.88E-01
TCERG1	0.256886	3.32E-09	1.34E-08
TCF12	0.02929	5.07E-01	5.71E-01
TCF15	-0.02518	5.69E-01	6.28E-01
TCF19	0.659072	1.78E-65	1.75E-63
TCF20	-0.08314	5.94E-02	8.63E-02
TCF21	-0.52188	2.56E-37	9.96E-36
TCF23	0.0033	9.40E-01	9.53E-01
TCF25	-0.36443	1.28E-17	1.20E-16
TCF3	0.26978	4.87E-10	2.16E-09
TCF4	-0.19108	1.27E-05	3.34E-05
TCF7L1	-0.31229	4.11E-13	2.51E-12
TCF7L2	-0.25711	3.21E-09	1.30E-08
TCF7	-0.07673	8.19E-02	1.15E-01
TCFL5	0.110524	1.21E-02	2.02E-02
TCHHL1	0.111256	1.15E-02	1.94E-02

TCHH	0.127715	3.69E-03	6.78E-03
TCHP	0.014121	7.49E-01	7.91E-01
TCIRG1	-0.14373	1.07E-03	2.14E-03
TCL1A	-0.07301	9.79E-02	1.35E-01
TCL1B	0.032502	4.62E-01	5.28E-01
TCL6	0.106381	1.57E-02	2.58E-02
TCN1	0.042093	3.40E-01	4.06E-01
TCN2	-0.05957	1.77E-01	2.29E-01
TCOF1	0.299503	3.91E-12	2.17E-11
TCP10L2	-0.01788	6.86E-01	7.35E-01
TCP10L	-0.14681	8.33E-04	1.69E-03
TCP10	-0.03547	4.22E-01	4.88E-01
TCP11L1	0.127611	3.72E-03	6.82E-03
TCP11L2	-0.17921	4.31E-05	1.06E-04
TCP11	-0.10091	2.20E-02	3.49E-02
TCP1	0.507457	4.68E-35	1.61E-33
TCTA	-0.39411	1.39E-20	1.71E-19
TCTE1	-0.28689	3.25E-11	1.62E-10
TCTE3	0.001376	9.75E-01	9.81E-01
TCTEX1D1	-0.22293	3.20E-07	1.03E-06
TCTEX1D2	0.251578	7.09E-09	2.76E-08
TCTEX1D4	-0.34434	8.79E-16	6.93E-15
TCTN1	-0.23793	4.63E-08	1.63E-07
TCTN2	-0.06301	1.53E-01	2.02E-01
TCTN3	-0.01703	7.00E-01	7.48E-01
TDGF1	-0.123	5.19E-03	9.29E-03
TDGF3	-0.02698	5.41E-01	6.04E-01
TDG	0.557338	2.30E-43	1.25E-41
TDH	-0.05516	2.11E-01	2.68E-01
TDO2	0.271675	3.64E-10	1.64E-09
TDP1	0.383742	1.63E-19	1.83E-18
TDP2	0.239245	3.88E-08	1.38E-07
TDRD10	-0.49908	8.61E-34	2.78E-32
TDRD12	0.165017	1.69E-04	3.81E-04
TDRD1	-0.08139	6.50E-02	9.37E-02
TDRD3	-0.34671	5.42E-16	4.37E-15
TDRD5	0.118844	6.93E-03	1.21E-02
TDRD6	-0.27092	4.09E-10	1.83E-09
TDRD7	0.144785	9.84E-04	1.98E-03
TDRD9	-0.08947	4.24E-02	6.35E-02
TDRG1	0.228534	1.58E-07	5.27E-07
TDRKH	0.023302	5.98E-01	6.55E-01
TEAD1	0.129879	3.15E-03	5.85E-03
TEAD2	-0.09333	3.42E-02	5.22E-02

TEAD3	-0.18049	3.79E-05	9.37E-05
TEAD4	0.471685	6.87E-30	1.75E-28
TECPR1	-0.11728	7.72E-03	1.34E-02
TECPR2	-0.21087	1.38E-06	4.10E-06
TECRL	0.047175	2.85E-01	3.48E-01
TECR	-0.17294	7.98E-05	1.89E-04
TECTA	-0.3278	2.30E-14	1.58E-13
TECTB	-0.06523	1.39E-01	1.85E-01
TEC	-0.05386	2.22E-01	2.80E-01
TEDDM1	-0.16469	1.74E-04	3.92E-04
TEF	-0.55096	3.20E-42	1.65E-40
TEKT1	-0.28217	6.99E-11	3.37E-10
TEKT2	-0.25152	7.15E-09	2.77E-08
TEKT3	-0.35963	3.61E-17	3.29E-16
TEKT4	-0.27271	3.10E-10	1.41E-09
TEKT5	-0.21103	1.35E-06	4.03E-06
TEK	-0.32112	8.12E-14	5.31E-13
TELO2	-0.05996	1.74E-01	2.26E-01
TENC1	-0.60726	3.24E-53	2.38E-51
TEP1	-0.19461	8.66E-06	2.34E-05
TEPP	-0.39224	2.18E-20	2.64E-19
TERC	0.14422	1.03E-03	2.07E-03
TERF1	0.175703	6.10E-05	1.47E-04
TERF2IP	-0.41271	1.34E-22	1.93E-21
TERF2	-0.11293	1.03E-02	1.75E-02
TERT	0.300092	3.54E-12	1.97E-11
TESC	-0.16307	2.02E-04	4.51E-04
TESK1	0.029194	5.09E-01	5.72E-01
TESK2	-0.14157	1.28E-03	2.52E-03
TES	0.208274	1.87E-06	5.47E-06
TET1	0.200709	4.42E-06	1.24E-05
TET2	-0.29574	7.44E-12	3.98E-11
TET3	0.18225	3.17E-05	7.92E-05
TEX101	0.183801	2.71E-05	6.83E-05
TEX10	0.422154	1.13E-23	1.80E-22
TEX11	0.155991	3.81E-04	8.14E-04
TEX12	0.015717	7.22E-01	7.68E-01
TEX13A	0.061773	1.62E-01	2.11E-01
TEX13B	0.099521	2.39E-02	3.77E-02
TEX14	-0.01414	7.49E-01	7.91E-01
TEX15	0.381509	2.74E-19	3.00E-18
TEX19	0.270149	4.60E-10	2.05E-09
TEX261	-0.03599	4.15E-01	4.81E-01
TEX264	-0.25501	4.35E-09	1.73E-08

TEX2	-0.16092	2.46E-04	5.41E-04
TEX9	-0.08973	4.18E-02	6.27E-02
TFAMP1	0.071571	1.05E-01	1.43E-01
TFAM	0.421087	1.50E-23	2.36E-22
TFAP2A	0.280878	8.60E-11	4.11E-10
TFAP2B	-0.03546	4.22E-01	4.88E-01
TFAP2C	-0.08254	6.12E-02	8.88E-02
TFAP2D	0.015211	7.31E-01	7.75E-01
TFAP2E	-0.35303	1.46E-16	1.24E-15
TFAP4	0.011186	8.00E-01	8.35E-01
TFB1M	0.055697	2.07E-01	2.63E-01
TFB2M	0.03748	3.96E-01	4.63E-01
TFCP2L1	-0.34681	5.31E-16	4.29E-15
TFCP2	0.16873	1.19E-04	2.75E-04
TFDP1	0.394689	1.21E-20	1.49E-19
TFDP2	0.315644	2.23E-13	1.40E-12
TFDP3	-0.02932	5.07E-01	5.71E-01
TFE3	-0.05568	2.07E-01	2.63E-01
TFEB	-0.42819	2.24E-24	3.77E-23
TFEC	0.043741	3.22E-01	3.87E-01
TFF1	0.087498	4.72E-02	6.99E-02
TFF2	0.008598	8.46E-01	8.74E-01
TFF3	-0.07779	7.78E-02	1.10E-01
TFG	0.432409	7.05E-25	1.23E-23
TFIP11	0.062084	1.59E-01	2.09E-01
TFPI2	0.119678	6.55E-03	1.15E-02
TFPI	-0.06114	1.66E-01	2.16E-01
TFPT	0.152238	5.27E-04	1.10E-03
TFR2	0.289712	2.04E-11	1.04E-10
TFRC	0.470491	9.97E-30	2.52E-28
TF	0.128591	3.46E-03	6.38E-03
TGDS	0.130963	2.90E-03	5.42E-03
TGFA	0.126757	3.96E-03	7.23E-03
TGFB1I1	-0.14485	9.78E-04	1.97E-03
TGFB1	-0.13818	1.67E-03	3.24E-03
TGFB2	-0.1417	1.26E-03	2.50E-03
TGFB3	-0.04489	3.09E-01	3.73E-01
TGFBI	0.255505	4.05E-09	1.62E-08
TGFBR1	0.027492	5.34E-01	5.97E-01
TGFBR2	-0.41432	8.82E-23	1.29E-21
TGFBR3	-0.32631	3.05E-14	2.07E-13
TGFBRAP1	0.058672	1.84E-01	2.37E-01
TGIF1	0.210332	1.47E-06	4.35E-06
TGIF2LX	0.170836	9.77E-05	2.28E-04

TGIF2LY	0.144882	9.76E-04	1.96E-03
TGIF2	0.108283	1.39E-02	2.31E-02
TGM1	-0.18864	1.64E-05	4.26E-05
TGM2	0.014155	7.49E-01	7.91E-01
TGM3	0.087792	4.64E-02	6.89E-02
TGM4	0.23482	6.99E-08	2.42E-07
TGM5	0.28451	4.79E-11	2.34E-10
TGM6	0.056997	1.97E-01	2.51E-01
TGM7	0.058388	1.86E-01	2.39E-01
TGOLN2	-0.12903	3.35E-03	6.19E-03
TGS1	0.236687	5.46E-08	1.91E-07
TG	0.045437	3.03E-01	3.67E-01
TH1L	0.160371	2.58E-04	5.66E-04
THADA	-0.0793	7.22E-02	1.03E-01
THAP10	0.261279	1.75E-09	7.26E-09
THAP11	-0.06977	1.14E-01	1.55E-01
THAP1	0.192836	1.05E-05	2.80E-05
THAP2	-0.1931	1.02E-05	2.72E-05
THAP3	-0.06874	1.19E-01	1.61E-01
THAP4	0.049447	2.63E-01	3.24E-01
THAP5	0.107148	1.50E-02	2.46E-02
THAP6	-0.0773	7.97E-02	1.13E-01
THAP7	0.010763	8.07E-01	8.41E-01
THAP8	0.01653	7.08E-01	7.56E-01
THAP9	-0.15052	6.10E-04	1.27E-03
THBD	-0.24827	1.13E-08	4.27E-08
THBS1	0.037821	3.92E-01	4.58E-01
THBS2	0.209805	1.56E-06	4.62E-06
THBS3	-0.20735	2.08E-06	6.05E-06
THBS4	-0.19042	1.36E-05	3.57E-05
THEG	0.157177	3.43E-04	7.39E-04
THEM4	-0.14671	8.39E-04	1.71E-03
THEM5	0.016524	7.08E-01	7.56E-01
THEMIS	-0.03971	3.68E-01	4.35E-01
THG1L	-0.09211	3.67E-02	5.56E-02
THNSL1	-0.00448	9.19E-01	9.36E-01
THNSL2	-0.06586	1.36E-01	1.81E-01
THOC1	0.164403	1.79E-04	4.02E-04
THOC2	0.035733	4.18E-01	4.85E-01
THOC3	0.337773	3.29E-15	2.44E-14
THOC4	0.655791	1.26E-64	1.21E-62
THOC5	0.052802	2.32E-01	2.90E-01
THOC6	0.013146	7.66E-01	8.05E-01
THOC7	0.124515	4.66E-03	8.41E-03

THOP1	0.447254	1.07E-26	2.16E-25
THPO	0.030742	4.86E-01	5.51E-01
THRAP3	-0.06218	1.59E-01	2.08E-01
THRA	-0.49102	1.32E-32	3.97E-31
THRB	-0.27237	3.27E-10	1.48E-09
THRSP	-0.07903	7.31E-02	1.04E-01
THSD1P1	-0.25657	3.47E-09	1.39E-08
THSD1	-0.34003	2.10E-15	1.58E-14
THSD4	-0.28192	7.28E-11	3.51E-10
THSD7A	0.097838	2.64E-02	4.12E-02
THSD7B	-0.42752	2.68E-24	4.49E-23
THTPA	-0.12073	6.08E-03	1.08E-02
THUMPD1	-0.35471	1.03E-16	8.87E-16
THUMPD2	0.153537	4.71E-04	9.93E-04
THUMPD3	0.239823	3.59E-08	1.28E-07
THY1	0.116201	8.30E-03	1.43E-02
THYN1	-0.19968	4.96E-06	1.38E-05
TH	0.223881	2.84E-07	9.23E-07
TIA1	-0.05767	1.91E-01	2.45E-01
TIAF1	-0.1301	3.10E-03	5.76E-03
TIAL1	0.227586	1.78E-07	5.92E-07
TIAM1	0.040966	3.54E-01	4.19E-01
TIAM2	0.038517	3.83E-01	4.49E-01
TICAM1	0.074622	9.07E-02	1.26E-01
TICAM2	0.078137	7.65E-02	1.09E-01
TIE1	-0.25686	3.33E-09	1.34E-08
TIFAB	-0.02644	5.49E-01	6.11E-01
TIFA	0.219748	4.74E-07	1.50E-06
TIGD1	0.016743	7.05E-01	7.53E-01
TIGD2	0.037689	3.93E-01	4.60E-01
TIGD3	0.175894	5.99E-05	1.44E-04
TIGD4	-0.18066	3.73E-05	9.22E-05
TIGD5	0.168907	1.17E-04	2.71E-04
TIGD6	-0.28948	2.12E-11	1.08E-10
TIGD7	0.006326	8.86E-01	9.08E-01
TIGIT	0.082226	6.22E-02	9.01E-02
TIMD4	-0.08424	5.61E-02	8.18E-02
TIMELESS	0.674261	1.51E-69	1.57E-67
TIMM10	0.308009	8.85E-13	5.23E-12
TIMM13	0.095123	3.09E-02	4.76E-02
TIMM16	0.100091	2.31E-02	3.66E-02
TIMM17A	0.235115	6.72E-08	2.34E-07
TIMM17B	0.221408	3.87E-07	1.23E-06
TIMM22	0.105878	1.62E-02	2.65E-02

TIMM44	0.264419	1.10E-09	4.66E-09
TIMM50	0.358815	4.30E-17	3.88E-16
TIMM8A	0.483307	1.68E-31	4.75E-30
TIMM8B	0.261093	1.79E-09	7.45E-09
TIMM9	0.279266	1.11E-10	5.26E-10
TIMP1	0.112554	1.06E-02	1.79E-02
TIMP2	0.039371	3.73E-01	4.39E-01
TIMP3	-0.20503	2.71E-06	7.80E-06
TIMP4	0.158573	3.03E-04	6.58E-04
TINAGL1	-0.01148	7.95E-01	8.31E-01
TINAG	0.131533	2.78E-03	5.21E-03
TINF2	0.077891	7.74E-02	1.10E-01
TIPARP	0.064303	1.45E-01	1.92E-01
TIPIN	0.575825	8.14E-47	5.10E-45
TIPRL	0.256196	3.67E-09	1.47E-08
TIRAP	-0.15441	4.37E-04	9.26E-04
TJAP1	-0.06537	1.39E-01	1.84E-01
TJP1	-0.14065	1.37E-03	2.70E-03
TJP2	-0.12146	5.78E-03	1.03E-02
TJP3	-0.24108	3.03E-08	1.09E-07
TK1	0.779126	4.03E-106	7.20E-104
TK2	-0.35429	1.12E-16	9.66E-16
TKTL1	0.016788	7.04E-01	7.52E-01
TKTL2	0.120923	6.00E-03	1.06E-02
TKT	-0.04642	2.93E-01	3.56E-01
TLCD1	0.053562	2.25E-01	2.83E-01
TLE1	0.135866	2.00E-03	3.83E-03
TLE2	-0.3703	3.50E-18	3.47E-17
TLE3	-0.09441	3.22E-02	4.94E-02
TLE4	-0.29465	8.95E-12	4.74E-11
TLE6	-0.16744	1.35E-04	3.08E-04
TLK1	0.312807	3.74E-13	2.30E-12
TLK2	0.257307	3.12E-09	1.26E-08
TLL1	-0.15907	2.90E-04	6.31E-04
TLL2	0.164269	1.81E-04	4.06E-04
TLN1	-0.11951	6.62E-03	1.16E-02
TLN2	-0.09247	3.59E-02	5.46E-02
TLR10	-0.18475	2.46E-05	6.23E-05
TLR1	0.055796	2.06E-01	2.62E-01
TLR2	-0.40361	1.35E-21	1.79E-20
TLR3	-0.21073	1.40E-06	4.17E-06
TLR4	-0.05284	2.31E-01	2.90E-01
TLR5	-0.56439	1.18E-44	6.78E-43
TLR6	0.236397	5.67E-08	1.98E-07

TLR7	-0.18586	2.19E-05	5.60E-05
TLR8	-0.01044	8.13E-01	8.46E-01
TLR9	-0.0262	5.53E-01	6.15E-01
TLX1NB	0.080678	6.73E-02	9.68E-02
TLX1	0.155893	3.84E-04	8.21E-04
TLX2	0.087739	4.66E-02	6.91E-02
TLX3	0.222673	3.30E-07	1.07E-06
TM2D1	0.038178	3.87E-01	4.54E-01
TM2D2	0.114695	9.18E-03	1.57E-02
TM2D3	-0.14897	6.95E-04	1.43E-03
TM4SF18	-0.06935	1.16E-01	1.57E-01
TM4SF19	0.312736	3.79E-13	2.32E-12
TM4SF1	-0.08434	5.58E-02	8.15E-02
TM4SF20	0.206576	2.27E-06	6.59E-06
TM4SF4	-0.16369	1.91E-04	4.27E-04
TM4SF5	-0.00777	8.60E-01	8.86E-01
TM6SF1	-0.0792	7.25E-02	1.04E-01
TM6SF2	0.137025	1.83E-03	3.52E-03
TM7SF2	-0.18685	1.98E-05	5.08E-05
TM7SF3	0.037486	3.96E-01	4.63E-01
TM7SF4	-0.15116	5.78E-04	1.20E-03
TM9SF1	0.027514	5.33E-01	5.96E-01
TM9SF2	-0.1448	9.83E-04	1.98E-03
TM9SF3	0.014683	7.40E-01	7.83E-01
TM9SF4	0.101928	2.07E-02	3.30E-02
TMBIM1	-0.13979	1.47E-03	2.88E-03
TMBIM4	-0.21553	7.91E-07	2.43E-06
TMBIM6	-0.02567	5.61E-01	6.22E-01
TMC1	-0.01912	6.65E-01	7.17E-01
TMC2	-0.1826	3.06E-05	7.66E-05
TMC3	-0.03997	3.65E-01	4.32E-01
TMC4	-0.38893	4.81E-20	5.65E-19
TMC5	-0.23063	1.21E-07	4.08E-07
TMC6	-0.12005	6.38E-03	1.12E-02
TMC7	-0.03662	4.07E-01	4.73E-01
TMC8	-0.16949	1.11E-04	2.57E-04
TMCC1	0.066158	1.34E-01	1.79E-01
TMCC2	-0.14454	1.00E-03	2.02E-03
TMCC3	0.084429	5.55E-02	8.11E-02
TMCO1	0.00091	9.84E-01	9.87E-01
TMCO2	-0.0646	1.43E-01	1.90E-01
TMCO3	0.019003	6.67E-01	7.18E-01
TMCO4	-0.25722	3.16E-09	1.28E-08
TMCO5A	-0.08106	6.60E-02	9.51E-02

TMCO6	-0.09751	2.69E-02	4.19E-02
TMCO7	-0.11356	9.90E-03	1.68E-02
TMED10P1	0.14429	1.02E-03	2.05E-03
TMED10	-0.04796	2.77E-01	3.39E-01
TMED1	0.023591	5.93E-01	6.51E-01
TMED2	0.358289	4.81E-17	4.32E-16
TMED3	-0.09762	2.67E-02	4.17E-02
TMED4	-0.09113	3.87E-02	5.84E-02
TMED5	0.130491	3.01E-03	5.60E-03
TMED6	-0.32329	5.41E-14	3.60E-13
TMED7-TICAM2	0.197069	6.62E-06	1.81E-05
TMED7	0.075213	8.82E-02	1.23E-01
TMED8	-0.02906	5.11E-01	5.74E-01
TMED9	0.006432	8.84E-01	9.06E-01
TMEFF1	0.41486	7.67E-23	1.13E-21
TMEFF2	-0.11694	7.90E-03	1.36E-02
TMEM100	-0.35532	9.04E-17	7.87E-16
TMEM101	-0.09372	3.35E-02	5.12E-02
TMEM102	-0.17823	4.75E-05	1.16E-04
TMEM104	0.12162	5.72E-03	1.02E-02
TMEM105	-0.34883	3.51E-16	2.87E-15
TMEM106A	0.007846	8.59E-01	8.85E-01
TMEM106B	0.173073	7.88E-05	1.86E-04
TMEM106C	0.516156	2.09E-36	7.75E-35
TMEM107	-0.08935	4.27E-02	6.38E-02
TMEM108	-0.4359	2.68E-25	4.85E-24
TMEM109	-0.18294	2.96E-05	7.41E-05
TMEM110	-0.18129	3.50E-05	8.68E-05
TMEM111	-0.19	1.42E-05	3.72E-05
TMEM114	-0.09506	3.10E-02	4.77E-02
TMEM115	-0.323	5.71E-14	3.79E-13
TMEM116	-0.11085	1.18E-02	1.98E-02
TMEM117	0.314458	2.77E-13	1.73E-12
TMEM119	-0.34461	8.33E-16	6.59E-15
TMEM11	0.18446	2.53E-05	6.41E-05
TMEM120A	-0.15676	3.56E-04	7.65E-04
TMEM120B	0.021515	6.26E-01	6.82E-01
TMEM121	0.053064	2.29E-01	2.88E-01
TMEM123	0.10088	2.20E-02	3.50E-02
TMEM125	-0.53935	3.31E-40	1.57E-38
TMEM126A	0.215272	8.17E-07	2.50E-06
TMEM126B	0.095497	3.02E-02	4.67E-02
TMEM127	0.064978	1.41E-01	1.87E-01
TMEM128	-0.13577	2.02E-03	3.86E-03

TMEM129	-0.29309	1.16E-11	6.08E-11
TMEM130	-0.455	1.11E-27	2.43E-26
TMEM131	-0.03252	4.62E-01	5.27E-01
TMEM132A	0.36643	8.24E-18	7.89E-17
TMEM132B	-0.12734	3.80E-03	6.95E-03
TMEM132C	-0.50054	5.22E-34	1.70E-32
TMEM132D	-0.43788	1.54E-25	2.85E-24
TMEM132E	-0.49279	7.28E-33	2.23E-31
TMEM133	-0.35421	1.14E-16	9.82E-16
TMEM134	-0.17452	6.85E-05	1.63E-04
TMEM135	0.207487	2.04E-06	5.96E-06
TMEM136	-0.07615	8.43E-02	1.18E-01
TMEM138	-0.01594	7.18E-01	7.65E-01
TMEM139	-0.11164	1.12E-02	1.89E-02
TMEM140	0.139226	1.54E-03	3.00E-03
TMEM141	0.047368	2.83E-01	3.46E-01
TMEM143	-0.23973	3.64E-08	1.30E-07
TMEM144	-0.02526	5.67E-01	6.27E-01
TMEM145	0.256749	3.38E-09	1.36E-08
TMEM146	-0.25308	5.73E-09	2.25E-08
TMEM147	0.208243	1.87E-06	5.49E-06
TMEM149	-0.03964	3.69E-01	4.36E-01
TMEM14A	0.250946	7.76E-09	2.99E-08
TMEM14B	0.214417	9.05E-07	2.75E-06
TMEM14C	0.101286	2.15E-02	3.42E-02
TMEM14E	-0.01358	7.59E-01	7.99E-01
TMEM150A	-0.38764	6.53E-20	7.55E-19
TMEM150B	0.067381	1.27E-01	1.70E-01
TMEM150C	-0.01561	7.24E-01	7.70E-01
TMEM151A	0.008137	8.54E-01	8.81E-01
TMEM151B	-0.18793	1.76E-05	4.58E-05
TMEM154	-0.16273	2.08E-04	4.65E-04
TMEM155	-0.16361	1.92E-04	4.30E-04
TMEM156	0.173705	7.41E-05	1.76E-04
TMEM158	0.354329	1.11E-16	9.59E-16
TMEM159	-0.25575	3.91E-09	1.56E-08
TMEM160	0.039472	3.71E-01	4.38E-01
TMEM161A	-0.04838	2.73E-01	3.35E-01
TMEM161B	-0.04895	2.68E-01	3.29E-01
TMEM163	-0.56007	7.33E-44	4.07E-42
TMEM164	-0.19762	6.23E-06	1.71E-05
TMEM165	0.150519	6.10E-04	1.27E-03
TMEM167A	0.008368	8.50E-01	8.77E-01
TMEM167B	-0.1956	7.78E-06	2.11E-05

TMEM168	-0.16065	2.52E-04	5.54E-04
TMEM169	0.080381	6.84E-02	9.81E-02
TMEM170A	-0.00316	9.43E-01	9.55E-01
TMEM170B	-0.25547	4.07E-09	1.62E-08
TMEM171	0.329576	1.63E-14	1.14E-13
TMEM173	-0.4628	1.07E-28	2.50E-27
TMEM174	0.074791	9.00E-02	1.25E-01
TMEM175	-0.32573	3.41E-14	2.31E-13
TMEM176A	-0.05943	1.78E-01	2.30E-01
TMEM176B	-0.06187	1.61E-01	2.10E-01
TMEM177	0.170143	1.04E-04	2.43E-04
TMEM178	-0.0959	2.96E-02	4.57E-02
TMEM179B	-0.1514	5.66E-04	1.18E-03
TMEM179	0.149365	6.73E-04	1.39E-03
TMEM17	0.018932	6.68E-01	7.20E-01
TMEM180	-0.12391	4.86E-03	8.75E-03
TMEM181	-0.13782	1.72E-03	3.33E-03
TMEM182	0.299655	3.81E-12	2.11E-11
TMEM183A	0.14473	9.88E-04	1.99E-03
TMEM184A	0.047799	2.79E-01	3.41E-01
TMEM184B	0.031863	4.71E-01	5.36E-01
TMEM184C	0.148909	6.99E-04	1.44E-03
TMEM185A	0.04322	3.28E-01	3.93E-01
TMEM185B	0.440091	8.29E-26	1.57E-24
TMEM186	-0.05531	2.10E-01	2.66E-01
TMEM187	-0.14183	1.25E-03	2.48E-03
TMEM188	0.033392	4.50E-01	5.16E-01
TMEM189-UBE2V1	0.145284	9.44E-04	1.90E-03
TMEM189	0.331434	1.14E-14	8.06E-14
TMEM18	0.092711	3.54E-02	5.39E-02
TMEM190	-0.26672	7.76E-10	3.36E-09
TMEM191A	-0.0766	8.25E-02	1.16E-01
TMEM192	-0.11134	1.15E-02	1.93E-02
TMEM194A	0.553461	1.15E-42	6.03E-41
TMEM194B	0.44277	3.88E-26	7.55E-25
TMEM195	0.160356	2.58E-04	5.67E-04
TMEM196	0.131717	2.75E-03	5.14E-03
TMEM198	0.016626	7.07E-01	7.54E-01
TMEM199	0.336311	4.39E-15	3.24E-14
TMEM19	0.158005	3.19E-04	6.90E-04
TMEM200A	-0.04124	3.50E-01	4.16E-01
TMEM200B	0.174478	6.88E-05	1.64E-04
TMEM200C	0.10201	2.06E-02	3.29E-02
TMEM201	0.19274	1.06E-05	2.82E-05

TMEM202	0.05704	1.96E-01	2.51E-01
TMEM203	0.118019	7.34E-03	1.28E-02
TMEM204	-0.36289	1.78E-17	1.67E-16
TMEM205	-0.19966	4.97E-06	1.39E-05
TMEM206	0.550191	4.36E-42	2.23E-40
TMEM207	0.058867	1.82E-01	2.35E-01
TMEM208	-0.00425	9.23E-01	9.39E-01
TMEM209	0.279733	1.03E-10	4.90E-10
TMEM20	-0.04072	3.56E-01	4.22E-01
TMEM211	-0.0576	1.92E-01	2.46E-01
TMEM212	-0.27013	4.62E-10	2.06E-09
TMEM213	-0.26256	1.44E-09	6.06E-09
TMEM214	0.050443	2.53E-01	3.14E-01
TMEM215	0.062655	1.56E-01	2.04E-01
TMEM216	0.108678	1.36E-02	2.25E-02
TMEM217	-0.1214	5.80E-03	1.03E-02
TMEM218	-0.03151	4.75E-01	5.41E-01
TMEM219	-0.24479	1.83E-08	6.77E-08
TMEM220	-0.3748	1.27E-18	1.31E-17
TMEM222	-0.2677	6.68E-10	2.92E-09
TMEM223	0.199502	5.06E-06	1.41E-05
TMEM229A	-0.14177	1.26E-03	2.49E-03
TMEM229B	0.044555	3.13E-01	3.77E-01
TMEM22	0.338056	3.11E-15	2.31E-14
TMEM231	-0.23945	3.78E-08	1.35E-07
TMEM232	-0.3531	1.44E-16	1.23E-15
TMEM233	-0.10508	1.71E-02	2.77E-02
TMEM25	-0.23333	8.49E-08	2.92E-07
TMEM26	0.235844	6.10E-08	2.13E-07
TMEM27	-0.27525	2.09E-10	9.68E-10
TMEM2	0.077635	7.84E-02	1.11E-01
TMEM30A	-0.07722	8.00E-02	1.13E-01
TMEM30B	-0.15341	4.76E-04	1.00E-03
TMEM30C	0.031542	4.75E-01	5.41E-01
TMEM31	-0.00606	8.91E-01	9.12E-01
TMEM33	0.173183	7.80E-05	1.85E-04
TMEM35	-0.15664	3.60E-04	7.73E-04
TMEM37	-0.33905	2.55E-15	1.91E-14
TMEM38A	0.068744	1.19E-01	1.61E-01
TMEM38B	0.514133	4.34E-36	1.59E-34
TMEM39A	0.283718	5.45E-11	2.65E-10
TMEM39B	-0.03083	4.85E-01	5.50E-01
TMEM40	0.185204	2.34E-05	5.97E-05
TMEM41A	0.21708	6.57E-07	2.04E-06

TMEM41B	-0.23687	5.33E-08	1.87E-07
TMEM42	-0.16418	1.82E-04	4.10E-04
TMEM43	-0.31356	3.27E-13	2.02E-12
TMEM44	0.199421	5.10E-06	1.42E-05
TMEM45A	0.217969	5.89E-07	1.84E-06
TMEM45B	0.022777	6.06E-01	6.63E-01
TMEM47	-0.22397	2.81E-07	9.13E-07
TMEM48	0.628666	5.33E-58	4.42E-56
TMEM49	0.246157	1.51E-08	5.65E-08
TMEM50A	-0.09334	3.42E-02	5.22E-02
TMEM50B	-0.4018	2.11E-21	2.77E-20
TMEM51	0.206453	2.30E-06	6.67E-06
TMEM52	-0.04451	3.13E-01	3.78E-01
TMEM53	-0.13545	2.07E-03	3.95E-03
TMEM54	-0.06535	1.39E-01	1.84E-01
TMEM55A	0.211448	1.29E-06	3.84E-06
TMEM55B	0.09247	3.59E-02	5.46E-02
TMEM56	-0.12709	3.87E-03	7.07E-03
TMEM57	-0.34748	4.63E-16	3.75E-15
TMEM59L	-0.29526	8.08E-12	4.30E-11
TMEM59	-0.35682	6.57E-17	5.80E-16
TMEM5	0.317467	1.60E-13	1.02E-12
TMEM60	0.038519	3.83E-01	4.49E-01
TMEM61	0.042519	3.36E-01	4.01E-01
TMEM62	-0.06579	1.36E-01	1.81E-01
TMEM63A	-0.44159	5.42E-26	1.04E-24
TMEM63B	-0.39186	2.39E-20	2.88E-19
TMEM63C	-0.1024	2.01E-02	3.22E-02
TMEM64	-0.0056	8.99E-01	9.19E-01
TMEM65	0.352298	1.71E-16	1.44E-15
TMEM66	-0.35998	3.35E-17	3.06E-16
TMEM67	-0.05476	2.15E-01	2.71E-01
TMEM68	0.077588	7.86E-02	1.11E-01
TMEM69	0.2913	1.57E-11	8.09E-11
TMEM70	0.165135	1.67E-04	3.77E-04
TMEM71	-0.10703	1.51E-02	2.48E-02
TMEM72	-0.07346	9.58E-02	1.33E-01
TMEM74	0.222784	3.26E-07	1.05E-06
TMEM79	0.255111	4.29E-09	1.71E-08
TMEM80	-0.43614	2.51E-25	4.56E-24
TMEM81	-0.04933	2.64E-01	3.25E-01
TMEM82	0.070248	1.11E-01	1.52E-01
TMEM84	-0.01843	6.77E-01	7.27E-01
TMEM85	0.139177	1.54E-03	3.01E-03

TMEM86A	-0.00547	9.01E-01	9.20E-01
TMEM86B	0.022729	6.07E-01	6.64E-01
TMEM87A	-0.35566	8.42E-17	7.33E-16
TMEM87B	0.101014	2.19E-02	3.47E-02
TMEM88B	-0.0807	6.72E-02	9.67E-02
TMEM88	-0.32747	2.44E-14	1.68E-13
TMEM89	-0.08305	5.97E-02	8.66E-02
TMEM8A	-0.08749	4.72E-02	6.99E-02
TMEM8B	-0.47582	1.87E-30	4.93E-29
TMEM8C	-0.11986	6.46E-03	1.14E-02
TMEM90A	-0.33316	8.16E-15	5.83E-14
TMEM90B	0.055805	2.06E-01	2.62E-01
TMEM91	-0.476	1.76E-30	4.67E-29
TMEM92	-0.20682	2.21E-06	6.42E-06
TMEM93	0.197164	6.55E-06	1.80E-05
TMEM95	0.020536	6.42E-01	6.96E-01
TMEM97	0.135151	2.11E-03	4.03E-03
TMEM98	-0.33289	8.59E-15	6.14E-14
TMEM99	-0.05422	2.19E-01	2.77E-01
TMEM9B	-0.38412	1.49E-19	1.68E-18
TMEM9	-0.21094	1.37E-06	4.07E-06
TMF1	-0.09896	2.47E-02	3.89E-02
TMIE	0.069066	1.17E-01	1.59E-01
TMIGD1	0.029558	5.03E-01	5.68E-01
TMIGD2	0.055172	2.11E-01	2.67E-01
TMLHE	0.284322	4.94E-11	2.41E-10
TMOD1	-0.14519	9.52E-04	1.92E-03
TMOD2	-0.0768	8.17E-02	1.15E-01
TMOD3	0.14233	1.20E-03	2.38E-03
TMOD4	-0.17207	8.68E-05	2.04E-04
TMPO	0.689484	7.01E-74	7.79E-72
TMPPE	-0.0257	5.61E-01	6.21E-01
TMPRSS11A	0.073996	9.35E-02	1.30E-01
TMPRSS11BNL	0.03622	4.12E-01	4.78E-01
TMPRSS11B	0.077834	7.76E-02	1.10E-01
TMPRSS11D	0.102966	1.94E-02	3.12E-02
TMPRSS11F	0.164051	1.85E-04	4.14E-04
TMPRSS12	-0.03749	3.96E-01	4.62E-01
TMPRSS13	-0.08737	4.75E-02	7.04E-02
TMPRSS15	0.144648	9.95E-04	2.00E-03
TMPRSS2	-0.5677	2.84E-45	1.67E-43
TMPRSS3	-0.03554	4.21E-01	4.87E-01
TMPRSS4	-0.14428	1.03E-03	2.06E-03
TMPRSS5	-0.08215	6.25E-02	9.04E-02

TMPRSS6	-0.17471	6.72E-05	1.61E-04
TMPRSS7	-0.17806	4.84E-05	1.18E-04
TMPRSS9	0.081035	6.61E-02	9.52E-02
TMSB10	0.327167	2.59E-14	1.77E-13
TMSB15A	0.172153	8.61E-05	2.03E-04
TMSB15B	0.170415	1.02E-04	2.37E-04
TMSB4Y	-0.0074	8.67E-01	8.92E-01
TMSL3	-0.1064	1.57E-02	2.57E-02
TMTC1	-0.06721	1.28E-01	1.71E-01
TMTC2	-0.08726	4.78E-02	7.07E-02
TMTC3	0.481316	3.20E-31	8.88E-30
TMTC4	-0.0683	1.22E-01	1.64E-01
TMUB1	0.115897	8.47E-03	1.45E-02
TMUB2	-0.01172	7.91E-01	8.27E-01
TMX1	0.326107	3.17E-14	2.15E-13
TMX2	0.19693	6.72E-06	1.84E-05
TMX3	-0.03074	4.86E-01	5.51E-01
TMX4	-0.26925	5.29E-10	2.33E-09
TNC	0.15945	2.80E-04	6.12E-04
TNFAIP1	0.186889	1.97E-05	5.07E-05
TNFAIP2	0.147762	7.69E-04	1.57E-03
TNFAIP3	0.114801	9.12E-03	1.56E-02
TNFAIP6	0.342559	1.26E-15	9.75E-15
TNFAIP8L1	0.119446	6.65E-03	1.17E-02
TNFAIP8L2	-0.13296	2.50E-03	4.71E-03
TNFAIP8L3	-0.11286	1.04E-02	1.76E-02
TNFAIP8	0.013545	7.59E-01	7.99E-01
TNFRSF10A	0.04803	2.77E-01	3.39E-01
TNFRSF10B	-0.03571	4.19E-01	4.85E-01
TNFRSF10C	-0.33101	1.24E-14	8.71E-14
TNFRSF10D	-0.07019	1.12E-01	1.52E-01
TNFRSF11A	-0.0009	9.84E-01	9.87E-01
TNFRSF11B	0.043984	3.19E-01	3.84E-01
TNFRSF12A	0.060908	1.68E-01	2.18E-01
TNFRSF13B	-0.31123	4.97E-13	3.02E-12
TNFRSF13C	-0.02724	5.37E-01	6.00E-01
TNFRSF14	-0.29649	6.56E-12	3.53E-11
TNFRSF17	-0.11991	6.44E-03	1.13E-02
TNFRSF18	0.161335	2.36E-04	5.23E-04
TNFRSF19	-0.29889	4.35E-12	2.39E-11
TNFRSF1A	0.020915	6.36E-01	6.91E-01
TNFRSF1B	-0.16989	1.07E-04	2.48E-04
TNFRSF21	0.193098	1.02E-05	2.72E-05
TNFRSF25	0.006639	8.81E-01	9.03E-01

TNFRSF4	0.030304	4.93E-01	5.57E-01
TNFRSF6B	0.10836	1.39E-02	2.30E-02
TNFRSF8	0.039851	3.67E-01	4.33E-01
TNFRSF9	0.283158	5.96E-11	2.89E-10
TNFSF10	-0.06213	1.59E-01	2.08E-01
TNFSF11	0.184108	2.62E-05	6.63E-05
TNFSF12-TNFSF13	-0.13915	1.55E-03	3.01E-03
TNFSF12	-0.44483	2.16E-26	4.24E-25
TNFSF13B	0.097144	2.75E-02	4.27E-02
TNFSF13	-0.46867	1.76E-29	4.38E-28
TNFSF14	-0.20059	4.48E-06	1.26E-05
TNFSF15	-0.29569	7.51E-12	4.01E-11
TNFSF18	-0.02739	5.35E-01	5.98E-01
TNFSF4	0.278673	1.22E-10	5.76E-10
TNFSF8	-0.09219	3.65E-02	5.53E-02
TNFSF9	0.022952	6.03E-01	6.60E-01
TNF	-0.01975	6.55E-01	7.08E-01
TNIK	-0.19618	7.29E-06	1.99E-05
TNIP1	-0.09273	3.54E-02	5.39E-02
TNIP2	0.14444	1.01E-03	2.03E-03
TNIP3	0.176974	5.38E-05	1.30E-04
TNK1	-0.26045	1.97E-09	8.15E-09
TNK2	-0.18756	1.83E-05	4.74E-05
TNKS1BP1	-0.33348	7.66E-15	5.49E-14
TNKS2	-0.07758	7.86E-02	1.11E-01
TNKS	-0.11529	8.83E-03	1.51E-02
TNMD	-0.02173	6.23E-01	6.79E-01
TNNC1	-0.35742	5.78E-17	5.15E-16
TNNC2	-0.04848	2.72E-01	3.34E-01
TNNI1	-0.17457	6.82E-05	1.63E-04
TNNI2	-0.12247	5.39E-03	9.61E-03
TNNI3K	-0.4249	5.43E-24	8.88E-23
TNNI3	0.157013	3.48E-04	7.49E-04
TNNT1	0.407577	4.96E-22	6.85E-21
TNNT2	-0.20403	3.04E-06	8.70E-06
TNNT3	-0.23359	8.21E-08	2.83E-07
TNN	-0.38362	1.68E-19	1.88E-18
TNP1	0.1309	2.92E-03	5.44E-03
TNP2	-0.00873	8.43E-01	8.72E-01
TNPO1	0.285673	3.97E-11	1.95E-10
TNPO2	0.132052	2.68E-03	5.02E-03
TNPO3	0.354256	1.13E-16	9.73E-16
TNRC18	-0.21487	8.57E-07	2.62E-06
TNRC6A	-0.22415	2.75E-07	8.95E-07

TNRC6B	-0.21823	5.71E-07	1.79E-06
TNRC6C	-0.35137	2.07E-16	1.74E-15
TNR	-0.26106	1.80E-09	7.49E-09
TNS1	-0.58568	9.55E-49	6.26E-47
TNS3	-0.01411	7.49E-01	7.91E-01
TNS4	0.144028	1.05E-03	2.10E-03
TNXB	-0.50855	3.19E-35	1.11E-33
TOB1	-0.37699	7.75E-19	8.11E-18
TOB2	-0.34359	1.02E-15	8.01E-15
TOE1	0.066723	1.30E-01	1.75E-01
TOLLIP	-0.30034	3.39E-12	1.89E-11
TOM1L1	0.007666	8.62E-01	8.87E-01
TOM1L2	-0.52674	4.19E-38	1.72E-36
TOM1	-0.31044	5.73E-13	3.45E-12
TOMM20L	0.037094	4.01E-01	4.67E-01
TOMM20	-0.06153	1.63E-01	2.13E-01
TOMM22	0.260568	1.94E-09	8.02E-09
TOMM34	0.212121	1.19E-06	3.57E-06
TOMM40L	0.232536	9.42E-08	3.22E-07
TOMM40	0.477945	9.46E-31	2.57E-29
TOMM5	0.458847	3.52E-28	7.95E-27
TOMM6	-0.00449	9.19E-01	9.35E-01
TOMM70A	0.442059	4.75E-26	9.18E-25
TOMM7	-0.08413	5.64E-02	8.23E-02
TOP1MT	-0.04177	3.44E-01	4.10E-01
TOP1P1	0.109754	1.27E-02	2.12E-02
TOP1P2	0.035025	4.28E-01	4.94E-01
TOP1	0.19498	8.32E-06	2.25E-05
TOP2A	0.86454	2.09E-155	1.49E-152
TOP2B	-0.17641	5.69E-05	1.37E-04
TOP3A	0.25201	6.67E-09	2.60E-08
TOP3B	-0.00074	9.87E-01	9.90E-01
TOPBP1	0.617004	2.39E-55	1.82E-53
TOPORS	-0.01514	7.32E-01	7.76E-01
TOR1AIP1	-0.20672	2.23E-06	6.48E-06
TOR1AIP2	-0.00528	9.05E-01	9.23E-01
TOR1A	0.187598	1.83E-05	4.73E-05
TOR1B	0.155264	4.06E-04	8.64E-04
TOR2A	-0.05148	2.44E-01	3.03E-01
TOR3A	0.151499	5.61E-04	1.17E-03
TOX2	-0.06138	1.64E-01	2.14E-01
TOX3	-0.17205	8.70E-05	2.04E-04
TOX4	0.059161	1.80E-01	2.33E-01
TOX	-0.28893	2.32E-11	1.18E-10

TP53AIP1	-0.02996	4.98E-01	5.62E-01
TP53BP1	0.079345	7.20E-02	1.03E-01
TP53BP2	0.055607	2.08E-01	2.63E-01
TP53I11	-0.24013	3.45E-08	1.24E-07
TP53I13	-0.0451	3.07E-01	3.71E-01
TP53I3	0.170786	9.82E-05	2.29E-04
TP53INP1	-0.34899	3.39E-16	2.78E-15
TP53INP2	0.123083	5.16E-03	9.23E-03
TP53RK	0.069158	1.17E-01	1.58E-01
TP53TG1	-0.10386	1.84E-02	2.97E-02
TP53TG3B	0.138095	1.68E-03	3.26E-03
TP53TG5	-0.01069	8.09E-01	8.42E-01
TP53	-0.17964	4.13E-05	1.02E-04
TP63	-0.0835	5.83E-02	8.48E-02
TP73	-0.01717	6.97E-01	7.46E-01
TPBG	0.268617	5.82E-10	2.56E-09
TPCN1	-0.0877	4.67E-02	6.92E-02
TPCN2	0.117227	7.74E-03	1.34E-02
TPD52L1	0.060452	1.71E-01	2.22E-01
TPD52L2	0.233487	8.32E-08	2.86E-07
TPD52L3	-0.06357	1.50E-01	1.97E-01
TPD52	0.181952	3.27E-05	8.14E-05
TPH1	-0.03394	4.42E-01	5.08E-01
TPH2	0.160922	2.45E-04	5.41E-04
TPI1P2	0.356302	7.34E-17	6.44E-16
TPI1P3	0.246539	1.44E-08	5.37E-08
TPI1	0.566626	4.52E-45	2.65E-43
TPK1	-0.18921	1.54E-05	4.03E-05
TPM1	0.017985	6.84E-01	7.34E-01
TPM2	0.021149	6.32E-01	6.87E-01
TPM3	0.499457	7.58E-34	2.45E-32
TPM4	0.288896	2.34E-11	1.19E-10
TPMT	-0.00818	8.53E-01	8.80E-01
TPO	0.028087	5.25E-01	5.88E-01
TPP1	-0.21823	5.71E-07	1.79E-06
TPP2	-0.15468	4.27E-04	9.05E-04
TPPP2	-0.16776	1.31E-04	3.00E-04
TPPP3	-0.42233	1.08E-23	1.72E-22
TPPP	-0.54179	1.27E-40	6.06E-39
TPRA1	-0.06847	1.21E-01	1.63E-01
TPRG1L	-0.38009	3.80E-19	4.10E-18
TPRG1	-0.19496	8.34E-06	2.26E-05
TPRKB	0.551565	2.49E-42	1.29E-40
TPRN	-0.00474	9.15E-01	9.32E-01

TPRX1	0.067467	1.26E-01	1.70E-01
TPRXL	0.040203	3.63E-01	4.29E-01
TPR	-0.01454	7.42E-01	7.85E-01
TPSAB1	-0.33474	5.99E-15	4.35E-14
TPSB2	-0.35605	7.74E-17	6.77E-16
TPSD1	-0.22008	4.55E-07	1.44E-06
TPSG1	-0.16078	2.49E-04	5.47E-04
TPST1	0.117173	7.77E-03	1.34E-02
TPST2	-0.02715	5.39E-01	6.01E-01
TPT1	-0.23653	5.57E-08	1.95E-07
TPTE2P1	-0.14352	1.09E-03	2.18E-03
TPTE2P3	-0.00047	9.91E-01	9.94E-01
TPTE2	0.031515	4.75E-01	5.41E-01
TPTE	0.201581	4.01E-06	1.13E-05
TPX2	0.882814	1.80E-170	3.00E-167
TRA2A	-0.08223	6.22E-02	9.01E-02
TRA2B	0.433959	4.60E-25	8.14E-24
TRABD	-0.08184	6.35E-02	9.18E-02
TRADD	-0.34397	9.48E-16	7.44E-15
TRAF1	-0.11698	7.87E-03	1.36E-02
TRAF2	0.041039	3.53E-01	4.19E-01
TRAF3IP1	-0.09365	3.36E-02	5.14E-02
TRAF3IP2	-0.10438	1.78E-02	2.88E-02
TRAF3IP3	-0.15485	4.20E-04	8.93E-04
TRAF3	0.139566	1.50E-03	2.93E-03
TRAF4	0.174359	6.96E-05	1.66E-04
TRAF5	-0.02405	5.86E-01	6.45E-01
TRAF6	-0.20029	4.63E-06	1.30E-05
TRAF7	0.097673	2.67E-02	4.16E-02
TRAFD1	0.057282	1.94E-01	2.49E-01
TRAIP	0.673406	2.60E-69	2.69E-67
TRAK1	-0.19708	6.61E-06	1.81E-05
TRAK2	-0.28711	3.13E-11	1.56E-10
TRAM1L1	0.012606	7.75E-01	8.13E-01
TRAM1	-0.15798	3.19E-04	6.91E-04
TRAM2	0.064819	1.42E-01	1.88E-01
TRANK1	-0.28505	4.39E-11	2.15E-10
TRAP1	0.090507	4.01E-02	6.03E-02
TRAPPC10	-0.1296	3.22E-03	5.96E-03
TRAPPC1	-0.20911	1.69E-06	4.99E-06
TRAPPC2L	-0.02983	4.99E-01	5.64E-01
TRAPPC2P1	0.071386	1.06E-01	1.45E-01
TRAPPC2	-0.17354	7.53E-05	1.79E-04
TRAPPC3	0.040546	3.58E-01	4.25E-01

TRAPPC4	-0.09401	3.29E-02	5.04E-02
TRAPPC5	0.051563	2.43E-01	3.03E-01
TRAPPC6A	-0.21008	1.51E-06	4.48E-06
TRAPPC6B	0.030785	4.86E-01	5.51E-01
TRAPPC9	-0.10405	1.82E-02	2.94E-02
TRAT1	-0.07034	1.11E-01	1.51E-01
TRDMT1	-0.19726	6.48E-06	1.78E-05
TRDN	0.18466	2.48E-05	6.29E-05
TREH	-0.22569	2.26E-07	7.43E-07
TREM1	-0.0703	1.11E-01	1.51E-01
TREM2	-0.15111	5.80E-04	1.21E-03
TREML1	-0.28037	9.33E-11	4.44E-10
TREML2P1	-0.01471	7.39E-01	7.83E-01
TREML2	-0.12005	6.38E-03	1.12E-02
TREML3	0.305625	1.35E-12	7.81E-12
TREML4	0.135351	2.08E-03	3.98E-03
TRERF1	0.200478	4.54E-06	1.27E-05
TREX1	-0.18841	1.68E-05	4.36E-05
TREX2	-0.14983	6.47E-04	1.34E-03
TRHDE	-0.18799	1.75E-05	4.55E-05
TRHR	0.098848	2.49E-02	3.91E-02
TRH	-0.01419	7.48E-01	7.90E-01
TRIAP1	0.338048	3.11E-15	2.32E-14
TRIB1	-0.09665	2.83E-02	4.39E-02
TRIB2	-0.00019	9.97E-01	9.97E-01
TRIB3	0.40422	1.15E-21	1.54E-20
TRIL	-0.08455	5.52E-02	8.06E-02
TRIM10	0.208806	1.75E-06	5.16E-06
TRIM11	-0.04941	2.63E-01	3.24E-01
TRIM13	-0.42434	6.31E-24	1.03E-22
TRIM14	0.030389	4.91E-01	5.56E-01
TRIM15	0.288108	2.66E-11	1.34E-10
TRIM16L	0.194385	8.87E-06	2.39E-05
TRIM16	0.231403	1.09E-07	3.71E-07
TRIM17	-0.20651	2.29E-06	6.63E-06
TRIM21	-0.00264	9.52E-01	9.62E-01
TRIM22	-0.30379	1.86E-12	1.07E-11
TRIM23	-0.20076	4.39E-06	1.24E-05
TRIM24	-0.04788	2.78E-01	3.40E-01
TRIM25	0.146464	8.57E-04	1.74E-03
TRIM26	-0.09794	2.63E-02	4.10E-02
TRIM27	-0.07732	7.96E-02	1.12E-01
TRIM28	0.321068	8.20E-14	5.36E-13
TRIM29	-0.01196	7.87E-01	8.23E-01

TRIM2	-0.21749	6.25E-07	1.95E-06
TRIM31	0.105347	1.68E-02	2.73E-02
TRIM32	-0.06197	1.60E-01	2.10E-01
TRIM33	0.02061	6.41E-01	6.95E-01
TRIM34	-0.11918	6.77E-03	1.19E-02
TRIM35	-0.27485	2.23E-10	1.03E-09
TRIM36	0.013984	7.52E-01	7.93E-01
TRIM37	0.191485	1.21E-05	3.21E-05
TRIM38	-0.20468	2.82E-06	8.10E-06
TRIM39	-0.31851	1.32E-13	8.43E-13
TRIM3	-0.34792	4.23E-16	3.44E-15
TRIM40	0.165964	1.55E-04	3.50E-04
TRIM41	-0.31991	1.02E-13	6.57E-13
TRIM42	0.076265	8.38E-02	1.18E-01
TRIM43	0.098774	2.50E-02	3.92E-02
TRIM44	0.035741	4.18E-01	4.85E-01
TRIM45	-0.04684	2.89E-01	3.51E-01
TRIM46	-0.00354	9.36E-01	9.50E-01
TRIM47	0.06048	1.71E-01	2.22E-01
TRIM48	-0.07371	9.47E-02	1.31E-01
TRIM49L	0.048763	2.69E-01	3.31E-01
TRIM49	-0.11584	8.51E-03	1.46E-02
TRIM4	-0.24685	1.38E-08	5.15E-08
TRIM50	-0.32437	4.41E-14	2.95E-13
TRIM52	-0.33177	1.07E-14	7.56E-14
TRIM53	-0.12496	4.51E-03	8.16E-03
TRIM54	-0.08563	5.21E-02	7.66E-02
TRIM55	-0.02996	4.98E-01	5.62E-01
TRIM56	-0.04716	2.85E-01	3.48E-01
TRIM58	-0.0114	7.96E-01	8.32E-01
TRIM59	0.55118	2.92E-42	1.51E-40
TRIM5	-0.26635	8.20E-10	3.54E-09
TRIM6-TRIM34	0.000482	9.91E-01	9.93E-01
TRIM60	0.023686	5.92E-01	6.50E-01
TRIM61	-0.14659	8.48E-04	1.72E-03
TRIM62	-0.17417	7.09E-05	1.69E-04
TRIM63	-0.3046	1.62E-12	9.30E-12
TRIM64	0.073329	9.65E-02	1.33E-01
TRIM65	-0.02534	5.66E-01	6.26E-01
TRIM66	-0.3856	1.06E-19	1.20E-18
TRIM67	-0.01284	7.71E-01	8.10E-01
TRIM68	-0.29521	8.14E-12	4.33E-11
TRIM69	0.21499	8.45E-07	2.58E-06
TRIM6	0.133287	2.44E-03	4.60E-03

TRIM71	-0.33461	6.14E-15	4.45E-14
TRIM72	0.104628	1.75E-02	2.84E-02
TRIM74	-0.17005	1.05E-04	2.45E-04
TRIM77	0.086913	4.87E-02	7.20E-02
TRIM78P	-0.20954	1.61E-06	4.76E-06
TRIM7	0.099251	2.43E-02	3.82E-02
TRIM8	-0.37606	9.57E-19	9.93E-18
TRIM9	0.245807	1.59E-08	5.91E-08
TRIML1	0.047782	2.79E-01	3.41E-01
TRIML2	0.152541	5.13E-04	1.08E-03
TRIOBP	-0.26245	1.47E-09	6.16E-09
TRIO	0.146117	8.82E-04	1.79E-03
TRIP10	0.05707	1.96E-01	2.50E-01
TRIP11	-0.01021	8.17E-01	8.49E-01
TRIP12	0.277401	1.49E-10	6.99E-10
TRIP13	0.767164	5.45E-101	8.85E-99
TRIP4	0.113481	9.96E-03	1.69E-02
TRIP6	-0.2011	4.23E-06	1.19E-05
TRIT1	0.183621	2.76E-05	6.95E-05
TRMT112	0.171011	9.61E-05	2.25E-04
TRMT11	0.140256	1.42E-03	2.78E-03
TRMT12	0.122154	5.51E-03	9.82E-03
TRMT1	0.029612	5.03E-01	5.67E-01
TRMT2A	0.080829	6.68E-02	9.61E-02
TRMT2B	-0.04338	3.26E-01	3.91E-01
TRMT5	0.104024	1.82E-02	2.94E-02
TRMT61A	0.02717	5.38E-01	6.01E-01
TRMT61B	0.357208	6.05E-17	5.38E-16
TRMT6	0.360624	2.91E-17	2.67E-16
TRMU	0.043107	3.29E-01	3.94E-01
TRNAU1AP	-0.13727	1.79E-03	3.46E-03
TRNP1	0.050457	2.53E-01	3.14E-01
TRNT1	0.093112	3.46E-02	5.28E-02
TROAP	0.804205	5.31E-118	1.18E-115
TROVE2	-0.0248	5.74E-01	6.34E-01
TRO	0.075051	8.89E-02	1.24E-01
TRPA1	0.397834	5.61E-21	7.14E-20
TRPC1	0.150896	5.91E-04	1.23E-03
TRPC2	-0.26478	1.04E-09	4.43E-09
TRPC3	-0.0811	6.59E-02	9.49E-02
TRPC4AP	0.056	2.05E-01	2.60E-01
TRPC4	0.066903	1.29E-01	1.74E-01
TRPC5	-0.02502	5.71E-01	6.31E-01
TRPC6	-0.32675	2.81E-14	1.92E-13

TRPC7	0.044988	3.08E-01	3.72E-01
TRPM1	-0.13931	1.53E-03	2.98E-03
TRPM2	0.239824	3.59E-08	1.28E-07
TRPM3	-0.09894	2.47E-02	3.89E-02
TRPM4	-0.29442	9.30E-12	4.92E-11
TRPM5	-0.1079	1.43E-02	2.36E-02
TRPM6	-0.05095	2.48E-01	3.09E-01
TRPM7	-0.10768	1.45E-02	2.39E-02
TRPM8	0.129642	3.21E-03	5.94E-03
TRPS1	0.111319	1.15E-02	1.93E-02
TRPT1	0.102989	1.94E-02	3.12E-02
TRPV1	-0.27525	2.09E-10	9.68E-10
TRPV2	-0.05435	2.18E-01	2.75E-01
TRPV3	0.241126	3.01E-08	1.09E-07
TRPV4	-0.08888	4.38E-02	6.54E-02
TRPV5	0.013613	7.58E-01	7.99E-01
TRPV6	-0.29002	1.94E-11	9.93E-11
TRRAP	0.142978	1.14E-03	2.27E-03
TRUB1	0.249595	9.38E-09	3.59E-08
TRUB2	0.106347	1.58E-02	2.58E-02
TRY6	0.077117	8.04E-02	1.13E-01
TSC1	-0.26549	9.33E-10	4.00E-09
TSC22D1	-0.28868	2.42E-11	1.23E-10
TSC22D2	0.358609	4.49E-17	4.05E-16
TSC22D3	-0.41996	2.02E-23	3.14E-22
TSC22D4	-0.07393	9.37E-02	1.30E-01
TSC2	-0.37819	5.89E-19	6.22E-18
TSEN15	0.18503	2.39E-05	6.07E-05
TSEN2	0.03095	4.83E-01	5.49E-01
TSEN34	0.031503	4.76E-01	5.41E-01
TSEN54	0.153723	4.64E-04	9.78E-04
TSFM	0.257167	3.19E-09	1.28E-08
TSG101	0.016124	7.15E-01	7.62E-01
TSG1	0.08736	4.75E-02	7.04E-02
TSGA10IP	-0.12652	4.03E-03	7.35E-03
TSGA10	-0.17979	4.07E-05	1.00E-04
TSGA13	0.10262	1.98E-02	3.18E-02
TSGA14	0.238931	4.05E-08	1.44E-07
TSHB	-0.15744	3.35E-04	7.23E-04
TSHR	-0.04793	2.78E-01	3.40E-01
TSHZ1	-0.14168	1.27E-03	2.50E-03
TSHZ2	0.033278	4.51E-01	5.17E-01
TSHZ3	0.002932	9.47E-01	9.58E-01
TSIX	-0.06831	1.22E-01	1.64E-01

TSKS	-0.0154	7.27E-01	7.73E-01
TSKU	0.31173	4.55E-13	2.77E-12
TSLP	-0.34189	1.44E-15	1.11E-14
TSNARE1	-0.32168	7.31E-14	4.80E-13
TSNAX-DISC1	0.193732	9.52E-06	2.56E-05
TSNAXIP1	-0.2341	7.68E-08	2.65E-07
TSNAX	-0.00543	9.02E-01	9.21E-01
TSN	0.428671	1.96E-24	3.32E-23
TSPAN10	0.0159	7.19E-01	7.65E-01
TSPAN11	-0.31326	3.45E-13	2.12E-12
TSPAN12	-0.18086	3.65E-05	9.04E-05
TSPAN13	-0.0682	1.22E-01	1.65E-01
TSPAN14	-0.05797	1.89E-01	2.43E-01
TSPAN15	-0.02532	5.66E-01	6.26E-01
TSPAN16	-0.03758	3.95E-01	4.62E-01
TSPAN17	0.029792	5.00E-01	5.64E-01
TSPAN18	-0.15496	4.16E-04	8.85E-04
TSPAN19	-0.19615	7.32E-06	1.99E-05
TSPAN1	-0.18515	2.36E-05	6.00E-05
TSPAN2	-0.03288	4.57E-01	5.23E-01
TSPAN31	-0.15455	4.32E-04	9.15E-04
TSPAN32	-0.27114	3.96E-10	1.78E-09
TSPAN33	0.218764	5.35E-07	1.68E-06
TSPAN3	-0.34159	1.53E-15	1.17E-14
TSPAN4	-0.436	2.61E-25	4.74E-24
TSPAN5	0.18977	1.45E-05	3.80E-05
TSPAN6	0.042797	3.32E-01	3.98E-01
TSPAN7	-0.33553	5.12E-15	3.75E-14
TSPAN8	-0.17618	5.82E-05	1.40E-04
TSPAN9	-0.24695	1.36E-08	5.09E-08
TSPO2	0.016295	7.12E-01	7.59E-01
TSPO	-0.13651	1.90E-03	3.66E-03
TSPY1	0.085832	5.16E-02	7.58E-02
TSPY2	0.103289	1.90E-02	3.06E-02
TSPY3	0.093893	3.31E-02	5.08E-02
TSPY4	0.064593	1.43E-01	1.90E-01
TSPYL1	-0.29426	9.56E-12	5.05E-11
TSPYL2	-0.38121	2.94E-19	3.20E-18
TSPYL3	-0.05135	2.45E-01	3.05E-01
TSPYL4	-0.06433	1.45E-01	1.92E-01
TSPYL5	0.015338	7.28E-01	7.74E-01
TSPYL6	-0.01667	7.06E-01	7.54E-01
TSR1	0.354823	1.00E-16	8.68E-16
TSR2	0.030687	4.87E-01	5.52E-01

TSSC1	0.47143	7.44E-30	1.89E-28
TSSC4	-0.04237	3.37E-01	4.03E-01
TSSK1B	-0.0633	1.51E-01	1.99E-01
TSSK3	-0.37153	2.66E-18	2.67E-17
TSSK4	-0.23294	8.93E-08	3.06E-07
TSSK6	-0.09571	2.99E-02	4.61E-02
TSTA3	-0.13618	1.95E-03	3.75E-03
TSTD1	-0.34384	9.73E-16	7.62E-15
TSTD2	-0.11145	1.14E-02	1.91E-02
TST	-0.30204	2.52E-12	1.43E-11
TTBK1	0.037841	3.91E-01	4.58E-01
TTBK2	-0.15071	6.00E-04	1.25E-03
TTC12	-0.34644	5.74E-16	4.61E-15
TTC13	-0.09454	3.20E-02	4.90E-02
TTC14	-0.19004	1.41E-05	3.70E-05
TTC15	-0.16244	2.14E-04	4.76E-04
TTC16	-0.37186	2.47E-18	2.48E-17
TTC17	-0.16431	1.80E-04	4.05E-04
TTC18	-0.29142	1.54E-11	7.94E-11
TTC19	-0.36106	2.65E-17	2.44E-16
TTC1	-0.03174	4.72E-01	5.38E-01
TTC21A	-0.3779	6.30E-19	6.63E-18
TTC21B	-0.08031	6.86E-02	9.85E-02
TTC22	-0.11975	6.51E-03	1.14E-02
TTC23L	-0.30122	2.91E-12	1.63E-11
TTC23	-0.17741	5.15E-05	1.25E-04
TTC24	0.077742	7.80E-02	1.10E-01
TTC25	-0.27506	2.15E-10	9.95E-10
TTC26	0.175003	6.53E-05	1.56E-04
TTC27	0.365505	1.01E-17	9.60E-17
TTC28	-0.32278	5.95E-14	3.94E-13
TTC29	-0.24709	1.33E-08	4.99E-08
TTC30A	-0.03071	4.87E-01	5.52E-01
TTC30B	-0.0631	1.53E-01	2.01E-01
TTC31	-0.12331	5.08E-03	9.11E-03
TTC32	0.178695	4.54E-05	1.11E-04
TTC33	0.003327	9.40E-01	9.53E-01
TTC35	-0.00067	9.88E-01	9.91E-01
TTC36	-0.11951	6.62E-03	1.16E-02
TTC37	-0.09567	2.99E-02	4.62E-02
TTC38	-0.28115	8.23E-11	3.94E-10
TTC39A	-0.08043	6.82E-02	9.79E-02
TTC39B	0.047186	2.85E-01	3.47E-01
TTC39C	0.058222	1.87E-01	2.41E-01

TTC3	-0.29787	5.18E-12	2.82E-11
TTC4	0.269254	5.28E-10	2.33E-09
TTC5	0.198287	5.79E-06	1.60E-05
TTC7A	-0.02611	5.54E-01	6.16E-01
TTC7B	0.141608	1.27E-03	2.52E-03
TTC8	0.121597	5.73E-03	1.02E-02
TTC9B	0.182242	3.17E-05	7.92E-05
TTC9C	0.299476	3.93E-12	2.17E-11
TTC9	0.073986	9.35E-02	1.30E-01
TTF1	0.056393	2.01E-01	2.57E-01
TTF2	0.566413	4.96E-45	2.90E-43
TTK	0.865476	4.02E-156	2.98E-153
TTLL10	-0.30708	1.04E-12	6.11E-12
TTLL11	-0.30379	1.86E-12	1.07E-11
TTLL12	0.16867	1.20E-04	2.77E-04
TTLL13	0.012845	7.71E-01	8.10E-01
TTLL1	-0.29443	9.29E-12	4.92E-11
TTLL2	-0.18072	3.70E-05	9.17E-05
TTLL3	-0.30682	1.09E-12	6.38E-12
TTLL4	0.330586	1.34E-14	9.43E-14
TTLL5	0.093519	3.39E-02	5.17E-02
TTLL6	-0.30795	8.95E-13	5.28E-12
TTLL7	0.206846	2.20E-06	6.40E-06
TTLL8	0.145878	8.99E-04	1.82E-03
TTLL9	-0.33197	1.03E-14	7.30E-14
TTL	0.495274	3.15E-33	9.82E-32
TTN	-0.15194	5.40E-04	1.13E-03
TTPAL	0.314604	2.70E-13	1.68E-12
TTPA	0.108909	1.34E-02	2.23E-02
TTR	-0.1137	9.81E-03	1.67E-02
TTTY10	-0.07682	8.16E-02	1.15E-01
TTTY14	-0.11259	1.06E-02	1.78E-02
TTTY15	0.033712	4.45E-01	5.12E-01
TTTY16	-0.02221	6.15E-01	6.72E-01
TTTY1B	0.017682	6.89E-01	7.38E-01
TTTY2	-0.08624	5.05E-02	7.43E-02
TTTY4C	-0.03706	4.01E-01	4.68E-01
TTTY5	-0.06533	1.39E-01	1.84E-01
TTTY6B	0.09146	3.80E-02	5.74E-02
TTTY6	0.050183	2.56E-01	3.16E-01
TTTY7	-0.00275	9.50E-01	9.61E-01
TTTY8	-0.1286	3.46E-03	6.38E-03
TTTY9B	-0.09725	2.73E-02	4.25E-02
TTYH1	0.22758	1.78E-07	5.92E-07

TTYH2	-0.27207	3.43E-10	1.55E-09
TTYH3	0.291635	1.48E-11	7.68E-11
TUBA1A	0.069702	1.14E-01	1.55E-01
TUBA1B	0.735879	6.43E-89	8.86E-87
TUBA1C	0.674831	1.05E-69	1.10E-67
TUBA3C	0.12988	3.15E-03	5.85E-03
TUBA3D	-0.05544	2.09E-01	2.65E-01
TUBA3E	0.073138	9.73E-02	1.34E-01
TUBA4A	0.238733	4.16E-08	1.48E-07
TUBA4B	-0.28203	7.15E-11	3.45E-10
TUBA8	0.001714	9.69E-01	9.76E-01
TUBAL3	0.146908	8.26E-04	1.68E-03
TUBB1	-0.27763	1.44E-10	6.75E-10
TUBB2A	0.205727	2.50E-06	7.22E-06
TUBB2B	0.214841	8.60E-07	2.63E-06
TUBB2C	0.207	2.16E-06	6.29E-06
TUBB3	0.430982	1.04E-24	1.80E-23
TUBB4Q	-0.09802	2.61E-02	4.08E-02
TUBB4	0.293	1.18E-11	6.17E-11
TUBB6	0.27864	1.23E-10	5.79E-10
TUBB8	-0.01349	7.60E-01	8.00E-01
TUBBP5	-0.15165	5.54E-04	1.16E-03
TUBB	0.617863	1.54E-55	1.18E-53
TUBD1	0.26642	8.11E-10	3.51E-09
TUBE1	0.021218	6.31E-01	6.86E-01
TUBG1	0.613861	1.19E-54	8.95E-53
TUBG2	-0.10255	1.99E-02	3.19E-02
TUBGCP2	-0.14461	9.98E-04	2.01E-03
TUBGCP3	0.0904	4.03E-02	6.06E-02
TUBGCP4	0.181056	3.58E-05	8.88E-05
TUBGCP5	-0.02959	5.03E-01	5.67E-01
TUBGCP6	-0.37996	3.92E-19	4.21E-18
TUB	-0.33482	5.89E-15	4.28E-14
TUFM	-0.04867	2.70E-01	3.32E-01
TUFT1	0.202799	3.49E-06	9.93E-06
TUG1	-0.0215	6.26E-01	6.82E-01
TULP1	0.15455	4.32E-04	9.15E-04
TULP2	0.048222	2.75E-01	3.37E-01
TULP3	0.197925	6.02E-06	1.66E-05
TULP4	-0.09841	2.55E-02	4.00E-02
TUSC1	-0.0901	4.10E-02	6.15E-02
TUSC2	-0.11972	6.53E-03	1.15E-02
TUSC3	0.191227	1.25E-05	3.29E-05
TUSC5	-0.06954	1.15E-01	1.56E-01

TUT1	-0.11905	6.84E-03	1.20E-02
TWF1	0.49835	1.11E-33	3.52E-32
TWF2	0.049696	2.60E-01	3.21E-01
TWIST1	0.335816	4.84E-15	3.55E-14
TWIST2	0.017865	6.86E-01	7.35E-01
TWISTNB	0.350926	2.27E-16	1.90E-15
TWSG1	0.204056	3.03E-06	8.67E-06
TXK	-0.05507	2.12E-01	2.68E-01
TXLNA	-0.09015	4.09E-02	6.14E-02
TXLNB	-0.05704	1.96E-01	2.51E-01
TXLNG	0.052759	2.32E-01	2.90E-01
TXN2	-0.05155	2.43E-01	3.03E-01
TXNDC11	-0.48471	1.06E-31	3.04E-30
TXNDC12	0.156423	3.66E-04	7.86E-04
TXNDC15	-0.29589	7.26E-12	3.88E-11
TXNDC16	0.011905	7.88E-01	8.24E-01
TXNDC17	0.014224	7.47E-01	7.90E-01
TXNDC2	0.049668	2.61E-01	3.22E-01
TXNDC3	-0.06074	1.69E-01	2.19E-01
TXNDC5	0.001043	9.81E-01	9.86E-01
TXNDC6	-0.29687	6.14E-12	3.32E-11
TXNDC9	0.351167	2.16E-16	1.81E-15
TXNIP	-0.40619	7.04E-22	9.59E-21
TXNL1	0.181996	3.25E-05	8.11E-05
TXNL4A	0.180707	3.71E-05	9.18E-05
TXNL4B	0.11734	7.68E-03	1.33E-02
TXNRD1	0.388226	5.68E-20	6.62E-19
TXNRD2	-0.31111	5.08E-13	3.08E-12
TXNRD3IT1	-0.03671	4.06E-01	4.72E-01
TXN	0.190198	1.39E-05	3.64E-05
TYK2	-0.17341	7.63E-05	1.81E-04
TYMP	0.119063	6.83E-03	1.19E-02
TYMS	0.792211	4.09E-112	8.17E-110
TYRO3	0.159644	2.75E-04	6.02E-04
TYROBP	-0.04889	2.68E-01	3.30E-01
TYRP1	-0.37361	1.67E-18	1.70E-17
TYR	-0.02766	5.31E-01	5.94E-01
TYSND1	-0.10394	1.83E-02	2.95E-02
TYW1B	-0.13483	2.17E-03	4.12E-03
TYW1	0.031322	4.78E-01	5.43E-01
TYW3	-0.04882	2.69E-01	3.31E-01
T	-0.08273	6.06E-02	8.80E-02
U2AF1L4	0.018656	6.73E-01	7.24E-01
U2AF1	0.190858	1.30E-05	3.41E-05

U2AF2	0.329642	1.61E-14	1.12E-13
UACA	0.066605	1.31E-01	1.76E-01
UAP1L1	0.032752	4.58E-01	5.25E-01
UAP1	0.160587	2.53E-04	5.56E-04
UBA1	0.110872	1.18E-02	1.98E-02
UBA2	0.514405	3.93E-36	1.45E-34
UBA3	0.149118	6.87E-04	1.42E-03
UBA52	-0.00404	9.27E-01	9.42E-01
UBA5	0.153276	4.82E-04	1.01E-03
UBA6	0.53601	1.22E-39	5.52E-38
UBA7	-0.32444	4.35E-14	2.92E-13
UBAC1	0.249495	9.51E-09	3.64E-08
UBAC2	0.113554	9.91E-03	1.68E-02
UBAP1	-0.05822	1.87E-01	2.41E-01
UBAP2L	0.036819	4.04E-01	4.71E-01
UBAP2	0.192741	1.06E-05	2.82E-05
UBASH3A	-0.07748	7.90E-02	1.12E-01
UBASH3B	0.072393	1.01E-01	1.39E-01
UBB	-0.09587	2.96E-02	4.57E-02
UBC	0.155433	4.00E-04	8.52E-04
UBD	0.200341	4.61E-06	1.29E-05
UBE2A	0.172061	8.69E-05	2.04E-04
UBE2B	-0.0567	1.99E-01	2.54E-01
UBE2CBP	0.143037	1.13E-03	2.26E-03
UBE2C	0.785091	8.42E-109	1.57E-106
UBE2D1	0.349043	3.35E-16	2.75E-15
UBE2D2	0.139677	1.48E-03	2.90E-03
UBE2D3	0.100501	2.26E-02	3.57E-02
UBE2D4	-0.05285	2.31E-01	2.90E-01
UBE2DNL	0.00179	9.68E-01	9.75E-01
UBE2E1	0.208972	1.72E-06	5.07E-06
UBE2E2	0.13989	1.46E-03	2.86E-03
UBE2E3	0.362673	1.87E-17	1.74E-16
UBE2F	0.430853	1.08E-24	1.86E-23
UBE2G1	0.186893	1.97E-05	5.06E-05
UBE2G2	-0.30574	1.32E-12	7.67E-12
UBE2H	0.184915	2.41E-05	6.14E-05
UBE2I	0.140025	1.44E-03	2.83E-03
UBE2J1	0.072294	1.01E-01	1.39E-01
UBE2J2	0.081511	6.46E-02	9.32E-02
UBE2K	0.374135	1.48E-18	1.52E-17
UBE2L3	0.199356	5.14E-06	1.43E-05
UBE2L6	0.242172	2.61E-08	9.50E-08
UBE2MP1	0.385605	1.05E-19	1.20E-18

UBE2M	0.358068	5.04E-17	4.51E-16
UBE2NL	0.193446	9.82E-06	2.63E-05
UBE2N	0.429133	1.73E-24	2.93E-23
UBE2O	0.223331	3.04E-07	9.85E-07
UBE2Q1	0.011501	7.95E-01	8.30E-01
UBE2Q2P1	-0.26686	7.59E-10	3.29E-09
UBE2Q2	0.172245	8.54E-05	2.01E-04
UBE2QL1	0.024458	5.80E-01	6.39E-01
UBE2R2	0.322248	6.57E-14	4.34E-13
UBE2S	0.690272	4.11E-74	4.59E-72
UBE2T	0.785105	8.30E-109	1.56E-106
UBE2U	-0.11492	9.04E-03	1.55E-02
UBE2V1	0.249127	1.00E-08	3.82E-08
UBE2V2	0.456499	7.11E-28	1.57E-26
UBE2W	0.095806	2.97E-02	4.59E-02
UBE2Z	0.349903	2.81E-16	2.32E-15
UBE3A	0.043471	3.25E-01	3.90E-01
UBE3B	-0.04587	2.99E-01	3.62E-01
UBE3C	0.344114	9.21E-16	7.24E-15
UBE4A	0.059983	1.74E-01	2.26E-01
UBE4B	-0.09394	3.31E-02	5.06E-02
UBFD1	0.080032	6.96E-02	9.97E-02
UBIAD1	-0.1398	1.47E-03	2.88E-03
UBL3	-0.47604	1.74E-30	4.62E-29
UBL4A	0.319732	1.05E-13	6.79E-13
UBL4B	0.034467	4.35E-01	5.02E-01
UBL5	0.112618	1.05E-02	1.78E-02
UBL7	-0.07051	1.10E-01	1.50E-01
UBLCP1	-0.13192	2.70E-03	5.07E-03
UBN1	-0.09583	2.97E-02	4.58E-02
UBN2	-0.19866	5.56E-06	1.54E-05
UBOX5	-0.13112	2.87E-03	5.36E-03
UBP1	-0.13613	1.96E-03	3.76E-03
UBQLN1	0.220141	4.52E-07	1.43E-06
UBQLN2	0.029673	5.02E-01	5.66E-01
UBQLN3	-0.07289	9.85E-02	1.36E-01
UBQLN4	0.044344	3.15E-01	3.80E-01
UBQLNL	-0.33553	5.12E-15	3.75E-14
UBR1	-0.16788	1.29E-04	2.97E-04
UBR2	-0.02557	5.63E-01	6.23E-01
UBR3	-0.17394	7.24E-05	1.72E-04
UBR4	-0.0034	9.39E-01	9.52E-01
UBR5	0.141478	1.29E-03	2.54E-03
UBR7	0.253949	5.06E-09	2.00E-08

UBTD1	-0.52406	1.14E-37	4.56E-36
UBTD2	0.062592	1.56E-01	2.05E-01
UBTFL1	0.012669	7.74E-01	8.12E-01
UBTF	0.065086	1.40E-01	1.86E-01
UBXN10	-0.37267	2.06E-18	2.08E-17
UBXN11	-0.21717	6.50E-07	2.02E-06
UBXN1	-0.04175	3.44E-01	4.10E-01
UBXN2A	0.476885	1.33E-30	3.55E-29
UBXN2B	-0.04992	2.58E-01	3.19E-01
UBXN4	0.214639	8.81E-07	2.69E-06
UBXN6	-0.22008	4.55E-07	1.44E-06
UBXN7	0.250672	8.06E-09	3.11E-08
UBXN8	-0.11132	1.15E-02	1.93E-02
UCA1	0.148863	7.02E-04	1.44E-03
UCHL1	0.27418	2.47E-10	1.13E-09
UCHL3	0.441106	6.23E-26	1.19E-24
UCHL5	0.353644	1.29E-16	1.10E-15
UCK1	-0.0415	3.47E-01	4.13E-01
UCK2	0.5565	3.26E-43	1.75E-41
UCKL1AS	-0.19901	5.34E-06	1.48E-05
UCKL1	-0.0228	6.06E-01	6.63E-01
UCMA	0.110151	1.24E-02	2.07E-02
UCN2	0.328491	2.01E-14	1.39E-13
UCN3	-0.26203	1.56E-09	6.54E-09
UCN	-0.04698	2.87E-01	3.50E-01
UCP1	0.130634	2.98E-03	5.54E-03
UCP2	-0.05824	1.87E-01	2.40E-01
UCP3	-0.26645	8.08E-10	3.50E-09
UEVLD	0.10943	1.30E-02	2.16E-02
UFC1	-0.16815	1.26E-04	2.90E-04
UFD1L	0.38264	2.11E-19	2.34E-18
UFM1	-0.05256	2.34E-01	2.92E-01
UFSP1	0.028146	5.24E-01	5.88E-01
UFSP2	-0.16588	1.56E-04	3.53E-04
UGCG	0.025318	5.66E-01	6.26E-01
UGDH	0.199013	5.34E-06	1.48E-05
UGGT1	0.229338	1.42E-07	4.79E-07
UGGT2	0.189522	1.49E-05	3.90E-05
UGP2	0.25425	4.85E-09	1.92E-08
UGT1A10	0.038236	3.87E-01	4.53E-01
UGT1A1	0.119733	6.52E-03	1.15E-02
UGT1A3	0.184255	2.58E-05	6.53E-05
UGT1A4	0.115952	8.44E-03	1.45E-02
UGT1A5	0.102899	1.95E-02	3.13E-02

UGT1A6	0.094063	3.28E-02	5.03E-02
UGT1A7	0.151009	5.85E-04	1.22E-03
UGT1A8	0.045748	3.00E-01	3.64E-01
UGT1A9	0.136433	1.91E-03	3.68E-03
UGT2A1	0.038823	3.79E-01	4.46E-01
UGT2A3	-0.037	4.02E-01	4.68E-01
UGT2B10	0.079696	7.08E-02	1.01E-01
UGT2B11	0.050107	2.56E-01	3.17E-01
UGT2B15	-0.1973	6.45E-06	1.77E-05
UGT2B28	0.019557	6.58E-01	7.11E-01
UGT2B4	0.011163	8.00E-01	8.35E-01
UGT2B7	0.145932	8.95E-04	1.81E-03
UGT3A1	0.178716	4.53E-05	1.11E-04
UGT3A2	0.163658	1.91E-04	4.28E-04
UGT8	0.238582	4.24E-08	1.51E-07
UHMK1	0.110824	1.18E-02	1.99E-02
UHRF1BP1L	0.296272	6.80E-12	3.65E-11
UHRF1BP1	0.123209	5.11E-03	9.16E-03
UHRF1	0.796677	2.92E-114	6.09E-112
UHRF2	0.198198	5.84E-06	1.61E-05
UIMC1	0.023767	5.90E-01	6.49E-01
ULBP1	0.147098	8.13E-04	1.66E-03
ULBP2	0.27733	1.51E-10	7.06E-10
ULBP3	0.063495	1.50E-01	1.98E-01
ULK1	0.066224	1.33E-01	1.78E-01
ULK2	-0.30465	1.60E-12	9.22E-12
ULK3	-0.16752	1.34E-04	3.06E-04
ULK4	-0.04507	3.07E-01	3.71E-01
UMODL1	0.029942	4.98E-01	5.62E-01
UMOD	0.106628	1.55E-02	2.54E-02
UMPS	0.441493	5.58E-26	1.07E-24
UNC119B	-0.18259	3.06E-05	7.66E-05
UNC119	0.08985	4.15E-02	6.23E-02
UNC13A	0.094149	3.27E-02	5.01E-02
UNC13B	-0.47991	5.04E-31	1.38E-29
UNC13C	-0.1691	1.15E-04	2.66E-04
UNC13D	0.084566	5.51E-02	8.06E-02
UNC45A	-0.10515	1.70E-02	2.76E-02
UNC45B	-0.43465	3.80E-25	6.78E-24
UNC50	-0.02976	5.00E-01	5.65E-01
UNC5A	0.00996	8.22E-01	8.53E-01
UNC5B	-0.06222	1.59E-01	2.08E-01
UNC5CL	-0.11953	6.61E-03	1.16E-02
UNC5C	-0.18072	3.70E-05	9.17E-05

UNC5D	0.153785	4.61E-04	9.73E-04
UNC80	-0.03854	3.83E-01	4.49E-01
UNC93A	0.123266	5.09E-03	9.13E-03
UNC93B1	-0.27067	4.25E-10	1.90E-09
UNG	0.625971	2.24E-57	1.85E-55
UNKL	-0.2044	2.91E-06	8.35E-06
UNK	0.079256	7.23E-02	1.03E-01
UOX	-0.05538	2.10E-01	2.66E-01
UPB1	-0.1604	2.57E-04	5.65E-04
UPF0639	-0.05488	2.14E-01	2.70E-01
UPF1	-0.23213	9.93E-08	3.39E-07
UPF2	0.014248	7.47E-01	7.90E-01
UPF3A	-0.22312	3.12E-07	1.01E-06
UPF3B	0.26492	1.02E-09	4.35E-09
UPK1A	0.240015	3.50E-08	1.25E-07
UPK1B	0.119086	6.82E-03	1.19E-02
UPK2	0.180002	3.98E-05	9.81E-05
UPK3A	-0.03453	4.34E-01	5.01E-01
UPK3BL	0.011744	7.90E-01	8.27E-01
UPK3B	-0.12685	3.93E-03	7.18E-03
UPP1	0.204638	2.84E-06	8.14E-06
UPP2	-0.15879	2.97E-04	6.47E-04
UPRT	-0.24856	1.08E-08	4.11E-08
UQCC	0.058436	1.85E-01	2.39E-01
UQCR10	-0.02664	5.46E-01	6.09E-01
UQCR11	0.067905	1.24E-01	1.67E-01
UQCRB	-0.00315	9.43E-01	9.55E-01
UQCRC1	0.200921	4.32E-06	1.21E-05
UQCRC2	0.017004	7.00E-01	7.49E-01
UQCRFS1	0.283973	5.23E-11	2.55E-10
UQCRHL	0.309637	6.62E-13	3.96E-12
UQCRH	0.279687	1.04E-10	4.93E-10
UQCRQ	0.070568	1.10E-01	1.50E-01
URB1	-0.00752	8.65E-01	8.90E-01
URB2	0.38187	2.52E-19	2.78E-18
URGCP	-0.09737	2.71E-02	4.23E-02
URM1	0.081806	6.36E-02	9.19E-02
UROC1	-0.06953	1.15E-01	1.56E-01
UROD	-0.05171	2.41E-01	3.01E-01
UROS	-0.09791	2.63E-02	4.11E-02
USE1	-0.17164	9.05E-05	2.12E-04
USF1	-0.06427	1.45E-01	1.92E-01
USF2	0.001183	9.79E-01	9.84E-01
USH1C	0.109491	1.29E-02	2.15E-02

USH1G	0.146893	8.27E-04	1.68E-03
USH2A	-0.06744	1.26E-01	1.70E-01
USHBP1	-0.4183	3.13E-23	4.79E-22
USMG5	0.228727	1.54E-07	5.15E-07
USO1	0.049442	2.63E-01	3.24E-01
USP10	0.229404	1.41E-07	4.75E-07
USP11	-0.06761	1.25E-01	1.69E-01
USP12	-0.01509	7.33E-01	7.77E-01
USP13	-0.0988	2.49E-02	3.92E-02
USP14	0.478106	8.99E-31	2.44E-29
USP15	0.334539	6.23E-15	4.51E-14
USP16	-0.11414	9.53E-03	1.62E-02
USP17L2	-0.1111	1.16E-02	1.95E-02
USP17L6P	-0.00157	9.72E-01	9.78E-01
USP17	0.003761	9.32E-01	9.46E-01
USP18	0.306307	1.20E-12	6.96E-12
USP19	-0.25991	2.14E-09	8.78E-09
USP1	0.465173	5.17E-29	1.24E-27
USP20	-0.28654	3.44E-11	1.71E-10
USP21	-0.11471	9.17E-03	1.57E-02
USP22	-0.12803	3.61E-03	6.63E-03
USP24	-0.14124	1.31E-03	2.59E-03
USP25	-0.10346	1.89E-02	3.03E-02
USP26	-0.07863	7.46E-02	1.06E-01
USP27X	-0.3239	4.82E-14	3.22E-13
USP28	0.055955	2.05E-01	2.60E-01
USP29	-0.06599	1.35E-01	1.80E-01
USP2	-0.19736	6.41E-06	1.76E-05
USP30	-0.0525	2.34E-01	2.93E-01
USP31	0.17838	4.68E-05	1.14E-04
USP32	0.258973	2.45E-09	1.00E-08
USP33	0.165392	1.63E-04	3.69E-04
USP34	-0.06852	1.20E-01	1.63E-01
USP35	0.109684	1.28E-02	2.13E-02
USP36	-0.03025	4.93E-01	5.58E-01
USP37	0.346784	5.34E-16	4.31E-15
USP38	0.073161	9.72E-02	1.34E-01
USP39	0.395748	9.34E-21	1.17E-19
USP3	0.067765	1.25E-01	1.68E-01
USP40	-0.07403	9.33E-02	1.29E-01
USP42	0.269116	5.39E-10	2.38E-09
USP43	-0.16819	1.26E-04	2.89E-04
USP44	-0.18295	2.95E-05	7.40E-05
USP45	0.132438	2.60E-03	4.89E-03

USP46	0.02107	6.33E-01	6.88E-01
USP47	-0.20026	4.65E-06	1.30E-05
USP48	-0.2006	4.48E-06	1.26E-05
USP49	0.025792	5.59E-01	6.20E-01
USP4	-0.29715	5.85E-12	3.17E-11
USP50	-0.00175	9.68E-01	9.75E-01
USP51	-0.17714	5.30E-05	1.28E-04
USP53	-0.3306	1.34E-14	9.40E-14
USP54	-0.45893	3.44E-28	7.78E-27
USP5	0.327225	2.56E-14	1.75E-13
USP6NL	-0.00275	9.50E-01	9.61E-01
USP6	-0.10101	2.19E-02	3.47E-02
USP7	-0.11614	8.34E-03	1.43E-02
USP8	-0.09535	3.05E-02	4.70E-02
USP9X	0.100849	2.21E-02	3.51E-02
USP9Y	-0.00782	8.59E-01	8.85E-01
USPL1	-0.118	7.35E-03	1.28E-02
UST	-0.35735	5.88E-17	5.23E-16
UTF1	-0.20377	3.13E-06	8.94E-06
UTP11L	0.343906	9.61E-16	7.53E-15
UTP14A	0.234925	6.89E-08	2.39E-07
UTP14C	-0.09824	2.58E-02	4.03E-02
UTP15	0.31352	3.29E-13	2.03E-12
UTP18	0.471387	7.54E-30	1.91E-28
UTP20	0.318051	1.44E-13	9.16E-13
UTP23	0.144809	9.82E-04	1.98E-03
UTP3	-0.05919	1.80E-01	2.32E-01
UTP6	0.510126	1.82E-35	6.43E-34
UTRN	-0.33035	1.41E-14	9.84E-14
UTS2D	-0.0921	3.67E-02	5.56E-02
UTS2R	0.040228	3.62E-01	4.29E-01
UTS2	0.056591	2.00E-01	2.55E-01
UTY	-0.0082	8.53E-01	8.80E-01
UVRAG	-0.10543	1.67E-02	2.72E-02
UXS1	-0.21631	7.20E-07	2.23E-06
UXT	0.143186	1.12E-03	2.23E-03
VAC14	0.083599	5.80E-02	8.44E-02
VAMP1	-0.18867	1.63E-05	4.25E-05
VAMP2	-0.62212	1.70E-56	1.36E-54
VAMP3	-0.12501	4.50E-03	8.13E-03
VAMP4	-0.12923	3.30E-03	6.11E-03
VAMP5	-0.07542	8.73E-02	1.22E-01
VAMP7	0.167638	1.32E-04	3.03E-04
VAMP8	-0.1837	2.74E-05	6.90E-05

VANGL1	0.381634	2.66E-19	2.92E-18
VANGL2	-0.1163	8.24E-03	1.42E-02
VAPA	-0.19901	5.34E-06	1.48E-05
VAPB	0.253568	5.35E-09	2.11E-08
VARS2	-0.02109	6.33E-01	6.88E-01
VARS	0.240106	3.46E-08	1.24E-07
VASH1	-0.16161	2.31E-04	5.11E-04
VASH2	0.187526	1.84E-05	4.76E-05
VASN	-0.04407	3.18E-01	3.83E-01
VASP	0.186588	2.03E-05	5.21E-05
VAT1L	0.056408	2.01E-01	2.56E-01
VAT1	0.168725	1.19E-04	2.75E-04
VAV1	-0.08711	4.82E-02	7.13E-02
VAV2	0.055465	2.09E-01	2.65E-01
VAV3	0.087815	4.64E-02	6.89E-02
VAX1	0.287704	2.84E-11	1.43E-10
VAX2	0.091163	3.86E-02	5.83E-02
VBP1	0.407449	5.12E-22	7.05E-21
VCAM1	0.115671	8.60E-03	1.48E-02
VCAN	0.276945	1.60E-10	7.49E-10
VCL	0.087818	4.64E-02	6.89E-02
VCPIP1	0.06998	1.13E-01	1.53E-01
VCP	0.206001	2.43E-06	7.01E-06
VCX2	0.070623	1.09E-01	1.49E-01
VCX3A	0.090294	4.05E-02	6.09E-02
VCX3B	0.077187	8.01E-02	1.13E-01
VCX	0.090502	4.01E-02	6.03E-02
VCY	0.09596	2.94E-02	4.55E-02
VDAC1	0.40772	4.78E-22	6.61E-21
VDAC2	0.371019	2.98E-18	2.97E-17
VDAC3	0.351817	1.89E-16	1.59E-15
VDR	-0.18008	3.95E-05	9.74E-05
VEGFA	0.115614	8.64E-03	1.48E-02
VEGFB	-0.17201	8.73E-05	2.05E-04
VEGFC	0.209727	1.58E-06	4.66E-06
VENTXP1	0.122521	5.37E-03	9.58E-03
VENTXP7	0.059711	1.76E-01	2.28E-01
VENTX	-0.25663	3.44E-09	1.38E-08
VEPH1	-0.31641	1.94E-13	1.22E-12
VEZF1	0.009528	8.29E-01	8.59E-01
VEZT	0.145659	9.16E-04	1.85E-03
VGF	0.330935	1.26E-14	8.83E-14
VGLL1	-0.08107	6.60E-02	9.51E-02
VGLL2	0.141564	1.28E-03	2.53E-03

VGLL3	-0.06214	1.59E-01	2.08E-01
VGLL4	-0.1714	9.26E-05	2.17E-04
VHLL	-0.00262	9.53E-01	9.62E-01
VHL	0.022063	6.17E-01	6.74E-01
VIL1	0.169632	1.10E-04	2.54E-04
VILL	-0.28019	9.60E-11	4.57E-10
VIM	-0.12005	6.38E-03	1.12E-02
VIPAR	0.012734	7.73E-01	8.11E-01
VIPR1	-0.55986	8.01E-44	4.43E-42
VIPR2	-0.0953	3.06E-02	4.72E-02
VIP	-0.28878	2.38E-11	1.21E-10
VIT	-0.10971	1.27E-02	2.12E-02
VKORC1L1	-0.14991	6.42E-04	1.33E-03
VKORC1	-0.12078	6.06E-03	1.07E-02
VLDLR	-0.12714	3.85E-03	7.05E-03
VMA21	0.24655	1.43E-08	5.36E-08
VMAC	-0.43226	7.35E-25	1.28E-23
VMO1	-0.1149	9.06E-03	1.55E-02
VN1R1	-0.22035	4.40E-07	1.40E-06
VN1R2	0.011297	7.98E-01	8.33E-01
VN1R4	0.009662	8.27E-01	8.57E-01
VN1R5	-0.1477	7.73E-04	1.58E-03
VNN1	0.072373	1.01E-01	1.39E-01
VNN2	-0.01242	7.79E-01	8.16E-01
VNN3	-0.02813	5.24E-01	5.88E-01
VOPP1	0.280212	9.56E-11	4.55E-10
VPRBP	-0.03421	4.39E-01	5.05E-01
VPREB1	0.013035	7.68E-01	8.07E-01
VPREB3	-0.06857	1.20E-01	1.62E-01
VPS11	-0.30746	9.75E-13	5.74E-12
VPS13A	-0.26406	1.16E-09	4.91E-09
VPS13B	-0.17814	4.80E-05	1.17E-04
VPS13C	-0.31305	3.58E-13	2.20E-12
VPS13D	-0.4434	3.25E-26	6.35E-25
VPS16	-0.01793	6.85E-01	7.35E-01
VPS18	0.076056	8.47E-02	1.19E-01
VPS24	-0.16527	1.65E-04	3.73E-04
VPS25	0.341912	1.44E-15	1.10E-14
VPS26A	0.232939	8.94E-08	3.07E-07
VPS26B	-0.23186	1.03E-07	3.51E-07
VPS28	-0.20352	3.22E-06	9.20E-06
VPS29	0.369941	3.79E-18	3.74E-17
VPS33A	0.514092	4.40E-36	1.61E-34
VPS33B	0.146321	8.67E-04	1.76E-03

VPS35	0.075147	8.85E-02	1.23E-01
VPS36	-0.3046	1.62E-12	9.31E-12
VPS37A	0.045456	3.03E-01	3.67E-01
VPS37B	-0.01086	8.06E-01	8.40E-01
VPS37C	0.130749	2.95E-03	5.50E-03
VPS37D	0.140659	1.37E-03	2.70E-03
VPS39	-0.36088	2.75E-17	2.53E-16
VPS41	-0.06198	1.60E-01	2.10E-01
VPS45	0.079656	7.09E-02	1.01E-01
VPS4A	-0.12319	5.12E-03	9.17E-03
VPS4B	0.072297	1.01E-01	1.39E-01
VPS52	-0.13966	1.49E-03	2.91E-03
VPS53	-0.27011	4.63E-10	2.06E-09
VPS54	0.144623	9.97E-04	2.00E-03
VPS72	0.212648	1.12E-06	3.36E-06
VPS8	0.028313	5.21E-01	5.85E-01
VRK1	0.655427	1.56E-64	1.49E-62
VRK2	0.60245	3.44E-52	2.46E-50
VRK3	-0.04166	3.45E-01	4.11E-01
VSIG10L	-0.01111	8.01E-01	8.36E-01
VSIG10	0.019747	6.55E-01	7.08E-01
VSIG1	-0.05292	2.31E-01	2.89E-01
VSIG2	-0.52201	2.45E-37	9.53E-36
VSIG4	-0.00862	8.45E-01	8.73E-01
VSIG8	-0.19483	8.45E-06	2.29E-05
VSNL1	-0.07075	1.09E-01	1.48E-01
VSTM1	-0.04303	3.30E-01	3.95E-01
VSTM2A	0.06244	1.57E-01	2.06E-01
VSTM2B	0.07468	9.05E-02	1.26E-01
VSTM2L	-0.04999	2.57E-01	3.19E-01
VSX1	0.121082	5.94E-03	1.05E-02
VSX2	0.041378	3.49E-01	4.15E-01
VTA1	0.425285	4.89E-24	8.04E-23
VTCN1	-0.04102	3.53E-01	4.19E-01
VTI1A	0.216977	6.65E-07	2.06E-06
VTI1B	0.114579	9.26E-03	1.58E-02
VTN	0.001318	9.76E-01	9.82E-01
VWA1	-0.29347	1.09E-11	5.73E-11
VWA2	-0.43013	1.32E-24	2.25E-23
VWA3A	-0.33149	1.13E-14	7.99E-14
VWA3B	-0.31789	1.48E-13	9.42E-13
VWA5A	-0.36997	3.77E-18	3.72E-17
VWA5B1	-0.15288	4.99E-04	1.05E-03
VWA5B2	0.013685	7.57E-01	7.98E-01

VWC2L	-0.03066	4.88E-01	5.52E-01
VWC2	-0.234	7.78E-08	2.69E-07
VWCE	-0.07996	6.98E-02	1.00E-01
VWDE	0.353648	1.28E-16	1.10E-15
VWF	-0.35611	7.64E-17	6.69E-16
WAC	0.080831	6.68E-02	9.61E-02
WAPAL	0.131157	2.86E-03	5.35E-03
WARS2	-0.03755	3.95E-01	4.62E-01
WARS	0.298801	4.42E-12	2.42E-11
WASF1	0.415158	7.10E-23	1.05E-21
WASF2	-0.00096	9.83E-01	9.87E-01
WASF3	-0.29213	1.37E-11	7.09E-11
WASH2P	-0.2694	5.17E-10	2.29E-09
WASH3P	-0.21445	9.01E-07	2.74E-06
WASH5P	-0.22031	4.43E-07	1.40E-06
WASH7P	-0.22558	2.29E-07	7.53E-07
WASL	0.073795	9.43E-02	1.31E-01
WAS	-0.10814	1.41E-02	2.33E-02
WBP11P1	0.333697	7.34E-15	5.28E-14
WBP11	0.457047	6.04E-28	1.34E-26
WBP1	-0.15484	4.21E-04	8.94E-04
WBP2NL	-0.27081	4.16E-10	1.86E-09
WBP2	-0.21364	9.92E-07	3.00E-06
WBP4	-0.05723	1.95E-01	2.49E-01
WBP5	0.075588	8.66E-02	1.21E-01
WBSCR16	0.069656	1.14E-01	1.55E-01
WBSCR17	-0.43856	1.28E-25	2.38E-24
WBSCR22	0.143034	1.13E-03	2.26E-03
WBSCR26	-0.24132	2.94E-08	1.06E-07
WBSCR27	-0.06297	1.54E-01	2.02E-01
WBSCR28	0.262249	1.51E-09	6.34E-09
WDFY1	0.187339	1.88E-05	4.85E-05
WDFY2	-0.1438	1.07E-03	2.13E-03
WDFY3	-0.26194	1.58E-09	6.62E-09
WDFY4	-0.20751	2.04E-06	5.95E-06
WDHD1	0.707215	2.80E-79	3.42E-77
WDR11	-0.14294	1.14E-03	2.27E-03
WDR12	0.528186	2.43E-38	1.01E-36
WDR13	-0.2155	7.95E-07	2.44E-06
WDR16	-0.28883	2.36E-11	1.20E-10
WDR17	-0.10154	2.12E-02	3.37E-02
WDR18	0.198571	5.61E-06	1.55E-05
WDR19	-0.32533	3.68E-14	2.48E-13
WDR1	0.161626	2.30E-04	5.10E-04

WDR20	-0.04155	3.47E-01	4.13E-01
WDR24	-0.21858	5.47E-07	1.71E-06
WDR25	-0.11573	8.57E-03	1.47E-02
WDR26	-0.17231	8.49E-05	2.00E-04
WDR27	-0.17922	4.30E-05	1.06E-04
WDR31	-0.13812	1.68E-03	3.26E-03
WDR33	-0.09848	2.54E-02	3.98E-02
WDR34	0.198638	5.57E-06	1.54E-05
WDR35	-0.1138	9.75E-03	1.66E-02
WDR36	0.162433	2.14E-04	4.76E-04
WDR37	-0.31542	2.33E-13	1.45E-12
WDR38	-0.23121	1.12E-07	3.80E-07
WDR3	0.344106	9.23E-16	7.25E-15
WDR41	0.102804	1.96E-02	3.15E-02
WDR43	0.587087	4.99E-49	3.33E-47
WDR44	0.102732	1.97E-02	3.16E-02
WDR45L	0.257191	3.17E-09	1.28E-08
WDR45	-0.11655	8.11E-03	1.40E-02
WDR46	0.064908	1.41E-01	1.88E-01
WDR47	-0.15859	3.03E-04	6.58E-04
WDR48	-0.19951	5.05E-06	1.41E-05
WDR49	-0.16188	2.25E-04	5.00E-04
WDR4	0.355947	7.91E-17	6.92E-16
WDR52	-0.30496	1.52E-12	8.77E-12
WDR53	0.463914	7.60E-29	1.80E-27
WDR54	0.087267	4.78E-02	7.07E-02
WDR55	-0.10324	1.91E-02	3.07E-02
WDR59	-0.24201	2.67E-08	9.70E-08
WDR5B	-0.15292	4.97E-04	1.04E-03
WDR5	0.273064	2.94E-10	1.34E-09
WDR60	-0.15451	4.33E-04	9.18E-04
WDR61	-0.02301	6.02E-01	6.60E-01
WDR62	0.610956	5.14E-54	3.83E-52
WDR63	-0.25418	4.89E-09	1.94E-08
WDR64	-0.0278	5.29E-01	5.92E-01
WDR65	-0.33935	2.40E-15	1.81E-14
WDR66	0.037573	3.95E-01	4.62E-01
WDR67	0.636718	6.74E-60	5.75E-58
WDR69	-0.00438	9.21E-01	9.37E-01
WDR6	-0.36612	8.82E-18	8.42E-17
WDR70	0.164592	1.76E-04	3.95E-04
WDR72	0.098171	2.59E-02	4.05E-02
WDR73	-0.07695	8.11E-02	1.14E-01
WDR74	0.204025	3.04E-06	8.70E-06

WDR75	0.515223	2.93E-36	1.08E-34
WDR76	0.760304	3.48E-98	5.43E-96
WDR77	0.154123	4.48E-04	9.47E-04
WDR78	-0.19063	1.33E-05	3.49E-05
WDR7	-0.11337	1.00E-02	1.70E-02
WDR81	-0.35491	9.85E-17	8.54E-16
WDR82	0.018145	6.81E-01	7.31E-01
WDR83	-0.15592	3.83E-04	8.19E-04
WDR85	0.073829	9.42E-02	1.31E-01
WDR86	-0.10747	1.47E-02	2.42E-02
WDR87	0.014552	7.42E-01	7.85E-01
WDR88	0.078153	7.64E-02	1.08E-01
WDR89	-0.10074	2.22E-02	3.53E-02
WDR8	0.045473	3.03E-01	3.67E-01
WDR90	-0.1995	5.06E-06	1.41E-05
WDR91	-0.30917	7.20E-13	4.29E-12
WDR92	0.092474	3.59E-02	5.46E-02
WDR93	-0.28459	4.73E-11	2.31E-10
WDSUB1	-0.00732	8.68E-01	8.93E-01
WDTC1	-0.41844	3.01E-23	4.62E-22
WDYHV1	0.324222	4.54E-14	3.03E-13
WEE1	0.031458	4.76E-01	5.42E-01
WEE2	-0.09329	3.43E-02	5.23E-02
WFDC10A	0.020173	6.48E-01	7.02E-01
WFDC10B	0.0169	7.02E-01	7.50E-01
WFDC11	-0.06605	1.34E-01	1.80E-01
WFDC12	-0.29695	6.06E-12	3.27E-11
WFDC13	-0.06722	1.28E-01	1.71E-01
WFDC1	-0.29308	1.17E-11	6.09E-11
WFDC2	-0.41953	2.26E-23	3.51E-22
WFDC3	0.071846	1.03E-01	1.42E-01
WFDC5	-0.21749	6.25E-07	1.95E-06
WFDC6	-0.24637	1.47E-08	5.49E-08
WFDC8	-0.03018	4.94E-01	5.59E-01
WFDC9	0.025097	5.70E-01	6.30E-01
WFIKKN1	-0.23583	6.11E-08	2.13E-07
WFIKKN2	-0.26741	6.99E-10	3.04E-09
WFS1	-0.29209	1.38E-11	7.14E-11
WHAMML1	-0.27332	2.82E-10	1.29E-09
WHAMML2	-0.16803	1.28E-04	2.93E-04
WHAMM	-0.34279	1.20E-15	9.34E-15
WHSC1L1	0.066607	1.31E-01	1.76E-01
WHSC1	0.467445	2.57E-29	6.29E-28
WHSC2	0.109905	1.26E-02	2.10E-02

WIBG	0.153868	4.58E-04	9.67E-04
WIF1	-0.47919	6.35E-31	1.74E-29
WIPF1	0.056256	2.02E-01	2.58E-01
WIPF2	0.058876	1.82E-01	2.35E-01
WIPF3	0.024492	5.79E-01	6.39E-01
WIPI1	0.110461	1.21E-02	2.03E-02
WIPI2	0.143115	1.13E-03	2.24E-03
WISP1	0.285157	4.31E-11	2.12E-10
WISP2	-0.19341	9.85E-06	2.64E-05
WISP3	0.088041	4.58E-02	6.81E-02
WIT1	0.245323	1.70E-08	6.31E-08
WIZ	0.043002	3.30E-01	3.95E-01
WLS	-0.21393	9.59E-07	2.91E-06
WNK1	-0.18608	2.14E-05	5.48E-05
WNK2	0.015216	7.30E-01	7.75E-01
WNK3	0.227909	1.71E-07	5.68E-07
WNK4	0.117522	7.59E-03	1.32E-02
WNT10A	-0.08507	5.37E-02	7.86E-02
WNT10B	0.01536	7.28E-01	7.73E-01
WNT11	-0.36959	4.09E-18	4.02E-17
WNT16	0.177188	5.27E-05	1.28E-04
WNT1	-0.11337	1.00E-02	1.70E-02
WNT2B	-0.37991	3.96E-19	4.26E-18
WNT2	-0.05927	1.79E-01	2.32E-01
WNT3A	-0.1948	8.48E-06	2.29E-05
WNT3	-0.05627	2.02E-01	2.58E-01
WNT4	-0.36914	4.53E-18	4.44E-17
WNT5A	-0.17632	5.74E-05	1.39E-04
WNT5B	-0.05789	1.90E-01	2.43E-01
WNT6	0.035195	4.25E-01	4.92E-01
WNT7A	0.033067	4.54E-01	5.20E-01
WNT7B	-0.19319	1.01E-05	2.70E-05
WNT8A	-0.04293	3.31E-01	3.96E-01
WNT8B	-0.12313	5.14E-03	9.21E-03
WNT9A	-0.04887	2.68E-01	3.30E-01
WNT9B	-0.24384	2.08E-08	7.65E-08
WRAP53	0.221804	3.68E-07	1.18E-06
WRB	-0.13287	2.52E-03	4.74E-03
WRNIP1	0.188079	1.74E-05	4.51E-05
WRN	0.1793	4.27E-05	1.05E-04
WSB1	-0.15966	2.75E-04	6.02E-04
WSB2	0.415785	6.03E-23	9.00E-22
WSCD1	-0.22488	2.51E-07	8.20E-07
WSCD2	-0.32235	6.45E-14	4.26E-13

WT1	0.297107	5.90E-12	3.20E-11
WTAP	0.23773	4.75E-08	1.68E-07
WTIP	-0.31634	1.97E-13	1.24E-12
WWC1	-0.30484	1.55E-12	8.94E-12
WWC2	-0.20928	1.66E-06	4.90E-06
WWC3	-0.37798	6.17E-19	6.51E-18
WWOX	-0.15731	3.39E-04	7.31E-04
WWP1	-0.04153	3.47E-01	4.13E-01
WWP2	-0.53241	4.87E-39	2.13E-37
WWTR1	0.016365	7.11E-01	7.58E-01
XAB2	-0.12112	5.92E-03	1.05E-02
XAF1	0.002204	9.60E-01	9.69E-01
XAGE1D	0.056026	2.04E-01	2.60E-01
XAGE2	-0.14759	7.81E-04	1.59E-03
XAGE3	-0.0797	7.07E-02	1.01E-01
XAGE5	0.094658	3.17E-02	4.87E-02
XBP1	-0.11224	1.08E-02	1.82E-02
XCL1	0.21369	9.87E-07	2.99E-06
XCL2	0.130666	2.97E-03	5.53E-03
XCR1	-0.26721	7.20E-10	3.13E-09
XDH	0.049835	2.59E-01	3.20E-01
XG	0.050816	2.50E-01	3.10E-01
XIAP	-0.17094	9.67E-05	2.26E-04
XIRP1	0.336346	4.36E-15	3.22E-14
XIRP2	0.058011	1.89E-01	2.42E-01
XIST	-0.09189	3.71E-02	5.62E-02
XKR3	0.025717	5.60E-01	6.21E-01
XKR4	-0.0708	1.09E-01	1.48E-01
XKR5	0.100844	2.21E-02	3.51E-02
XKR6	-0.14238	1.20E-03	2.37E-03
XKR7	0.125939	4.20E-03	7.64E-03
XKR8	-0.30126	2.89E-12	1.62E-11
XKR9	0.025461	5.64E-01	6.24E-01
XKRX	-0.15257	5.12E-04	1.07E-03
XKRY2	-0.00139	9.75E-01	9.81E-01
XK	-0.10793	1.43E-02	2.36E-02
XPA	-0.36975	3.95E-18	3.89E-17
XPC	-0.49531	3.12E-33	9.73E-32
XPNPEP1	0.300605	3.24E-12	1.81E-11
XPNPEP2	-0.08851	4.47E-02	6.66E-02
XPNPEP3	0.018095	6.82E-01	7.32E-01
XPO1	0.552374	1.79E-42	9.37E-41
XPO4	0.031296	4.79E-01	5.44E-01
XPO5	0.421345	1.40E-23	2.22E-22

XPO6	0.228292	1.63E-07	5.43E-07
XPO7	0.102155	2.04E-02	3.26E-02
XPOT	0.54997	4.77E-42	2.43E-40
XPR1	0.019097	6.65E-01	7.17E-01
XRCC1	0.014269	7.47E-01	7.89E-01
XRCC2	0.727099	7.79E-86	1.01E-83
XRCC3	0.410621	2.29E-22	3.24E-21
XRCC4	0.225689	2.26E-07	7.44E-07
XRCC5	0.426476	3.55E-24	5.90E-23
XRCC6BP1	0.230771	1.18E-07	4.01E-07
XRCC6	0.136763	1.87E-03	3.59E-03
XRN1	0.177545	5.09E-05	1.24E-04
XRN2	0.154706	4.26E-04	9.04E-04
XRRA1	-0.09771	2.66E-02	4.15E-02
XYLB	0.293685	1.05E-11	5.54E-11
XYLT1	-0.05835	1.86E-01	2.39E-01
XYLT2	-0.10203	2.06E-02	3.28E-02
YAF2	0.211802	1.23E-06	3.70E-06
YAP1	-0.28393	5.26E-11	2.57E-10
YARS2	0.496499	2.08E-33	6.54E-32
YARS	0.230499	1.23E-07	4.15E-07
YBX1	0.430271	1.27E-24	2.17E-23
YBX2	0.294884	8.60E-12	4.57E-11
YDJC	0.271436	3.78E-10	1.70E-09
YEATS2	0.403008	1.56E-21	2.07E-20
YEATS4	0.361731	2.30E-17	2.13E-16
YES1	0.35295	1.49E-16	1.27E-15
YIF1A	0.145898	8.98E-04	1.82E-03
YIF1B	0.301979	2.55E-12	1.44E-11
YIPF1	0.024991	5.72E-01	6.31E-01
YIPF2	-0.0064	8.85E-01	9.07E-01
YIPF3	-0.22581	2.23E-07	7.34E-07
YIPF4	0.31734	1.64E-13	1.04E-12
YIPF5	0.060145	1.73E-01	2.25E-01
YIPF6	0.244458	1.91E-08	7.06E-08
YIPF7	-0.04934	2.64E-01	3.25E-01
YJEFN3	-0.02636	5.51E-01	6.12E-01
YKT6	0.451115	3.49E-27	7.31E-26
YLPM1	-0.05137	2.45E-01	3.05E-01
YME1L1	0.349361	3.14E-16	2.59E-15
YOD1	-0.18127	3.50E-05	8.70E-05
YPEL1	-0.32482	4.05E-14	2.72E-13
YPEL2	-0.19895	5.37E-06	1.49E-05
YPEL3	-0.48664	5.63E-32	1.64E-30

YPEL4	0.027586	5.32E-01	5.95E-01
YPEL5	-0.29201	1.39E-11	7.23E-11
YRDC	0.332632	9.04E-15	6.44E-14
YSK4	-0.2558	3.88E-09	1.55E-08
YTHDC1	-0.17324	7.76E-05	1.84E-04
YTHDC2	-0.24234	2.55E-08	9.30E-08
YTHDF1	0.063295	1.51E-01	1.99E-01
YTHDF2	-0.0863	5.03E-02	7.41E-02
YTHDF3	0.123636	4.96E-03	8.91E-03
YWHAB	0.225478	2.33E-07	7.63E-07
YWHAE	0.191989	1.15E-05	3.05E-05
YWHAG	0.528993	1.79E-38	7.53E-37
YWHAH	0.168294	1.24E-04	2.86E-04
YWHAQ	0.571246	6.11E-46	3.71E-44
YWHAZ	0.404687	1.03E-21	1.38E-20
YY1AP1	-0.09348	3.39E-02	5.18E-02
YY1	0.388975	4.76E-20	5.59E-19
YY2	0.118404	7.15E-03	1.24E-02
ZACN	0.094748	3.16E-02	4.85E-02
ZADH2	-0.09759	2.68E-02	4.17E-02
ZAK	0.179052	4.38E-05	1.07E-04
ZAN	0.080584	6.77E-02	9.72E-02
ZAP70	-0.13004	3.11E-03	5.78E-03
ZAR1L	0.091368	3.82E-02	5.77E-02
ZAR1	-0.08268	6.08E-02	8.82E-02
ZBBX	-0.20042	4.57E-06	1.28E-05
ZBED1	0.034049	4.41E-01	5.07E-01
ZBED2	0.11628	8.26E-03	1.42E-02
ZBED3	-0.26496	1.01E-09	4.32E-09
ZBED4	0.118435	7.13E-03	1.24E-02
ZBED5	-0.2642	1.13E-09	4.81E-09
ZBP1	0.034657	4.33E-01	4.99E-01
ZBTB10	0.196087	7.37E-06	2.01E-05
ZBTB11	0.065379	1.38E-01	1.84E-01
ZBTB12	0.062546	1.56E-01	2.05E-01
ZBTB16	-0.49358	5.58E-33	1.72E-31
ZBTB17	-0.02778	5.29E-01	5.93E-01
ZBTB1	-0.11724	7.74E-03	1.34E-02
ZBTB20	-0.08929	4.28E-02	6.40E-02
ZBTB22	-0.38889	4.85E-20	5.69E-19
ZBTB24	-0.04825	2.74E-01	3.36E-01
ZBTB25	-0.04332	3.27E-01	3.92E-01
ZBTB26	0.123114	5.15E-03	9.22E-03
ZBTB2	0.257361	3.10E-09	1.25E-08

ZBTB32	0.030264	4.93E-01	5.58E-01
ZBTB33	0.098518	2.54E-02	3.98E-02
ZBTB34	-0.17721	5.26E-05	1.28E-04
ZBTB37	-0.09501	3.11E-02	4.78E-02
ZBTB38	0.072077	1.02E-01	1.40E-01
ZBTB39	0.122536	5.36E-03	9.57E-03
ZBTB3	-0.35722	6.03E-17	5.36E-16
ZBTB40	-0.28168	7.56E-11	3.63E-10
ZBTB41	0.060121	1.73E-01	2.25E-01
ZBTB42	-0.25313	5.69E-09	2.24E-08
ZBTB43	-0.29574	7.44E-12	3.98E-11
ZBTB44	-0.21818	5.74E-07	1.80E-06
ZBTB45	-0.11985	6.47E-03	1.14E-02
ZBTB46	-0.25594	3.80E-09	1.52E-08
ZBTB47	-0.28066	8.90E-11	4.25E-10
ZBTB48	-0.27692	1.61E-10	7.51E-10
ZBTB49	-0.19358	9.67E-06	2.59E-05
ZBTB4	-0.66531	4.06E-67	4.09E-65
ZBTB5	-0.03315	4.53E-01	5.19E-01
ZBTB6	0.073671	9.49E-02	1.31E-01
ZBTB7A	-0.23305	8.80E-08	3.02E-07
ZBTB7B	-0.14769	7.74E-04	1.58E-03
ZBTB7C	-0.38893	4.81E-20	5.65E-19
ZBTB8A	-0.10879	1.35E-02	2.24E-02
ZBTB8B	0.258313	2.70E-09	1.10E-08
ZBTB8OS	0.11009	1.24E-02	2.07E-02
ZBTB9	0.13846	1.63E-03	3.17E-03
ZC3H10	-0.05611	2.04E-01	2.59E-01
ZC3H11A	-0.23933	3.84E-08	1.37E-07
ZC3H12A	0.033196	4.52E-01	5.18E-01
ZC3H12B	-0.25544	4.09E-09	1.63E-08
ZC3H12C	-0.06364	1.49E-01	1.97E-01
ZC3H12D	-0.17922	4.31E-05	1.06E-04
ZC3H13	-0.04067	3.57E-01	4.23E-01
ZC3H14	-0.01027	8.16E-01	8.48E-01
ZC3H15	0.535834	1.30E-39	5.87E-38
ZC3H18	0.07657	8.26E-02	1.16E-01
ZC3H3	0.05298	2.30E-01	2.88E-01
ZC3H4	-0.13046	3.02E-03	5.61E-03
ZC3H6	-0.39577	9.29E-21	1.16E-19
ZC3H7A	-0.42093	1.56E-23	2.45E-22
ZC3H7B	-0.30708	1.04E-12	6.11E-12
ZC3H8	0.475909	1.81E-30	4.79E-29
ZC3HAV1L	0.285631	3.99E-11	1.97E-10

ZC3HAV1	0.065471	1.38E-01	1.84E-01
ZC3HC1	0.418864	2.70E-23	4.15E-22
ZC4H2	-0.00365	9.34E-01	9.48E-01
ZCCHC10	-0.06338	1.51E-01	1.99E-01
ZCCHC11	0.052279	2.36E-01	2.95E-01
ZCCHC12	-0.05578	2.06E-01	2.62E-01
ZCCHC13	0.112996	1.03E-02	1.74E-02
ZCCHC14	-0.29259	1.27E-11	6.60E-11
ZCCHC16	-0.07589	8.53E-02	1.20E-01
ZCCHC17	0.122961	5.20E-03	9.31E-03
ZCCHC18	-0.12284	5.25E-03	9.38E-03
ZCCHC24	-0.41177	1.70E-22	2.44E-21
ZCCHC2	-0.09635	2.88E-02	4.46E-02
ZCCHC3	-0.06147	1.64E-01	2.14E-01
ZCCHC4	0.12365	4.95E-03	8.91E-03
ZCCHC5	-0.0428	3.32E-01	3.98E-01
ZCCHC6	-0.06005	1.74E-01	2.25E-01
ZCCHC7	0.183258	2.86E-05	7.19E-05
ZCCHC8	0.27224	3.34E-10	1.51E-09
ZCCHC9	0.240978	3.07E-08	1.11E-07
ZCRB1	0.203993	3.05E-06	8.73E-06
ZCWPW1	-0.29697	6.04E-12	3.27E-11
ZCWPW2	-0.5011	4.31E-34	1.42E-32
ZDBF2	0.140887	1.35E-03	2.66E-03
ZDHHC11	-0.33193	1.04E-14	7.35E-14
ZDHHC12	0.109754	1.27E-02	2.12E-02
ZDHHC13	0.136068	1.97E-03	3.78E-03
ZDHHC14	0.07936	7.20E-02	1.03E-01
ZDHHC15	-0.30914	7.23E-13	4.31E-12
ZDHHC16	-0.33994	2.13E-15	1.61E-14
ZDHHC17	-0.01084	8.06E-01	8.40E-01
ZDHHC18	0.199631	4.99E-06	1.39E-05
ZDHHC19	0.032631	4.60E-01	5.26E-01
ZDHHC1	-0.51047	1.61E-35	5.73E-34
ZDHHC20	0.1551	4.11E-04	8.75E-04
ZDHHC21	-0.13209	2.67E-03	5.01E-03
ZDHHC22	0.127993	3.62E-03	6.65E-03
ZDHHC23	0.098541	2.53E-02	3.97E-02
ZDHHC24	-0.03566	4.19E-01	4.86E-01
ZDHHC2	-0.19606	7.39E-06	2.01E-05
ZDHHC3	-0.27214	3.39E-10	1.53E-09
ZDHHC4	0.039166	3.75E-01	4.41E-01
ZDHHC5	0.12336	5.06E-03	9.07E-03
ZDHHC6	0.086919	4.87E-02	7.19E-02

ZDHHC7	-0.38838	5.47E-20	6.38E-19
ZDHHC8P1	-0.25993	2.13E-09	8.76E-09
ZDHHC8	-0.21435	9.12E-07	2.77E-06
ZDHHC9	-0.23568	6.24E-08	2.17E-07
ZEB1	-0.21458	8.88E-07	2.70E-06
ZEB2	-0.16467	1.74E-04	3.93E-04
ZER1	-0.42771	2.55E-24	4.28E-23
ZFAND1	0.147055	8.16E-04	1.66E-03
ZFAND2A	0.323145	5.56E-14	3.69E-13
ZFAND2B	-0.19652	7.03E-06	1.92E-05
ZFAND3	-0.034	4.41E-01	5.08E-01
ZFAND5	-0.32382	4.89E-14	3.26E-13
ZFAND6	-0.02441	5.80E-01	6.40E-01
ZFATAS	-0.03023	4.94E-01	5.58E-01
ZFAT	-0.06712	1.28E-01	1.72E-01
ZFC3H1	-0.04108	3.52E-01	4.18E-01
ZFHX3	-0.24931	9.77E-09	3.73E-08
ZFHX4	0.169189	1.14E-04	2.64E-04
ZFP106	-0.19435	8.90E-06	2.40E-05
ZFP112	-0.07089	1.08E-01	1.48E-01
ZFP14	-0.22019	4.49E-07	1.42E-06
ZFP161	-0.12759	3.73E-03	6.83E-03
ZFP1	0.003082	9.44E-01	9.56E-01
ZFP28	-0.10453	1.77E-02	2.86E-02
ZFP2	-0.52793	2.68E-38	1.11E-36
ZFP30	0.146521	8.53E-04	1.73E-03
ZFP36L1	-0.21093	1.37E-06	4.07E-06
ZFP36L2	-0.14281	1.16E-03	2.30E-03
ZFP36	-0.33328	7.97E-15	5.70E-14
ZFP37	-0.00477	9.14E-01	9.31E-01
ZFP3	-0.34827	3.93E-16	3.21E-15
ZFP41	-0.14024	1.42E-03	2.79E-03
ZFP42	0.024779	5.75E-01	6.34E-01
ZFP57	0.1441	1.04E-03	2.09E-03
ZFP62	-0.18675	2.00E-05	5.13E-05
ZFP64	0.247136	1.32E-08	4.97E-08
ZFP82	0.039425	3.72E-01	4.38E-01
ZFP90	-0.25896	2.45E-09	1.00E-08
ZFP91-CNTF	0.091543	3.78E-02	5.72E-02
ZFP91	0.213808	9.73E-07	2.95E-06
ZFP92	-0.1989	5.41E-06	1.50E-05
ZFPL1	0.145264	9.46E-04	1.91E-03
ZFPM1	-0.2475	1.26E-08	4.73E-08
ZFPM2	-0.19067	1.32E-05	3.48E-05

ZFR2	-0.0439	3.20E-01	3.85E-01
ZFR	0.30885	7.62E-13	4.53E-12
ZFX	-0.09633	2.88E-02	4.46E-02
ZFYVE16	-0.17264	8.22E-05	1.94E-04
ZFYVE19	-0.01787	6.86E-01	7.35E-01
ZFYVE1	-0.15279	5.02E-04	1.05E-03
ZFYVE20	-0.38576	1.02E-19	1.16E-18
ZFYVE21	-0.25972	2.19E-09	9.01E-09
ZFYVE26	-0.08868	4.43E-02	6.60E-02
ZFYVE27	-0.22216	3.52E-07	1.13E-06
ZFYVE28	-0.29481	8.71E-12	4.62E-11
ZFYVE9	-0.11722	7.75E-03	1.34E-02
ZFY	0.024091	5.85E-01	6.44E-01
ZG16B	0.030431	4.91E-01	5.56E-01
ZG16	0.085701	5.19E-02	7.63E-02
ZGLP1	-0.16455	1.76E-04	3.97E-04
ZGPAT	-0.08972	4.18E-02	6.27E-02
ZHX1	-0.00847	8.48E-01	8.75E-01
ZHX2	-0.24455	1.89E-08	6.98E-08
ZHX3	-0.20865	1.79E-06	5.26E-06
ZIC1	0.173764	7.37E-05	1.75E-04
ZIC2	0.257201	3.17E-09	1.28E-08
ZIC3	0.090313	4.05E-02	6.09E-02
ZIC4	0.188687	1.63E-05	4.24E-05
ZIC5	0.251478	7.19E-09	2.79E-08
ZIK1	-0.01566	7.23E-01	7.69E-01
ZIM2	-0.00131	9.76E-01	9.82E-01
ZIM3	0.063402	1.51E-01	1.99E-01
ZKSCAN1	0.177369	5.18E-05	1.26E-04
ZKSCAN2	-0.07678	8.17E-02	1.15E-01
ZKSCAN3	-0.06146	1.64E-01	2.14E-01
ZKSCAN4	0.009823	8.24E-01	8.55E-01
ZKSCAN5	0.26424	1.13E-09	4.78E-09
ZMAT1	-0.43689	2.04E-25	3.72E-24
ZMAT2	-0.06586	1.36E-01	1.81E-01
ZMAT3	-0.03936	3.73E-01	4.39E-01
ZMAT4	0.159715	2.74E-04	5.99E-04
ZMAT5	-0.12532	4.39E-03	7.97E-03
ZMIZ1	-0.28864	2.44E-11	1.23E-10
ZMIZ2	0.036751	4.05E-01	4.72E-01
ZMPSTE24	0.160567	2.53E-04	5.57E-04
ZMYM1	0.20698	2.17E-06	6.30E-06
ZMYM2	0.036088	4.14E-01	4.80E-01
ZMYM3	-0.133	2.49E-03	4.69E-03

ZMYM4	0.027309	5.36E-01	5.99E-01
ZMYM5	-0.1216	5.73E-03	1.02E-02
ZMYM6	-0.20054	4.51E-06	1.27E-05
ZMYND10	-0.295	8.43E-12	4.48E-11
ZMYND11	-0.32274	6.00E-14	3.97E-13
ZMYND12	-0.43625	2.43E-25	4.43E-24
ZMYND15	-0.45416	1.42E-27	3.08E-26
ZMYND17	-0.22025	4.46E-07	1.41E-06
ZMYND19	0.272079	3.42E-10	1.55E-09
ZMYND8	-0.00413	9.25E-01	9.40E-01
ZNF100	0.018979	6.67E-01	7.19E-01
ZNF101	0.101896	2.07E-02	3.31E-02
ZNF107	0.204979	2.73E-06	7.84E-06
ZNF10	-0.21689	6.72E-07	2.08E-06
ZNF114	0.203973	3.06E-06	8.75E-06
ZNF117	-0.31394	3.05E-13	1.89E-12
ZNF121	0.033512	4.48E-01	5.14E-01
ZNF124	-0.02763	5.32E-01	5.95E-01
ZNF12	0.077711	7.81E-02	1.11E-01
ZNF131	0.335887	4.78E-15	3.51E-14
ZNF132	-0.31921	1.16E-13	7.46E-13
ZNF133	-0.11226	1.08E-02	1.82E-02
ZNF134	-0.02446	5.80E-01	6.39E-01
ZNF135	-0.08145	6.48E-02	9.34E-02
ZNF136	-0.1976	6.25E-06	1.72E-05
ZNF137	-0.18199	3.26E-05	8.11E-05
ZNF138	0.237253	5.06E-08	1.78E-07
ZNF140	0.096604	2.84E-02	4.40E-02
ZNF141	-0.11362	9.87E-03	1.68E-02
ZNF142	-0.00734	8.68E-01	8.92E-01
ZNF143	0.289457	2.13E-11	1.08E-10
ZNF146	0.313624	3.23E-13	2.00E-12
ZNF148	0.112804	1.04E-02	1.76E-02
ZNF14	-0.2282	1.65E-07	5.49E-07
ZNF154	-0.25174	6.93E-09	2.70E-08
ZNF155	-0.13159	2.77E-03	5.18E-03
ZNF157	0.019335	6.62E-01	7.14E-01
ZNF160	-0.23077	1.18E-07	4.02E-07
ZNF165	0.10417	1.80E-02	2.92E-02
ZNF167	-0.2592	2.37E-09	9.69E-09
ZNF169	-0.23336	8.46E-08	2.91E-07
ZNF16	-0.03091	4.84E-01	5.49E-01
ZNF174	-0.21869	5.40E-07	1.69E-06
ZNF175	-0.19719	6.53E-06	1.79E-05

ZNF177	-0.17795	4.89E-05	1.19E-04
ZNF17	-0.20028	4.64E-06	1.30E-05
ZNF180	0.020177	6.48E-01	7.02E-01
ZNF181	-0.18526	2.33E-05	5.93E-05
ZNF182	-0.09265	3.55E-02	5.41E-02
ZNF184	-0.00249	9.55E-01	9.64E-01
ZNF185	0.101777	2.09E-02	3.33E-02
ZNF187	-0.15193	5.41E-04	1.13E-03
ZNF189	-0.18348	2.80E-05	7.03E-05
ZNF18	-0.25294	5.84E-09	2.29E-08
ZNF192	-0.13982	1.47E-03	2.87E-03
ZNF193	-0.06675	1.30E-01	1.75E-01
ZNF195	0.098417	2.55E-02	4.00E-02
ZNF197	-0.10375	1.85E-02	2.98E-02
ZNF19	-0.47332	4.11E-30	1.06E-28
ZNF200	0.256127	3.70E-09	1.48E-08
ZNF202	-0.00218	9.61E-01	9.69E-01
ZNF204P	-0.4063	6.85E-22	9.34E-21
ZNF205	-0.15507	4.12E-04	8.77E-04
ZNF207	0.332077	1.01E-14	7.15E-14
ZNF208	-0.01519	7.31E-01	7.76E-01
ZNF20	-0.24918	9.95E-09	3.79E-08
ZNF211	-0.31792	1.47E-13	9.39E-13
ZNF212	0.041122	3.52E-01	4.18E-01
ZNF213	-0.30898	7.44E-13	4.43E-12
ZNF214	-0.30517	1.46E-12	8.45E-12
ZNF215	0.065514	1.38E-01	1.83E-01
ZNF217	0.087652	4.68E-02	6.94E-02
ZNF219	-0.30834	8.34E-13	4.94E-12
ZNF221	0.097023	2.77E-02	4.30E-02
ZNF222	0.05007	2.57E-01	3.18E-01
ZNF223	-0.08659	4.95E-02	7.31E-02
ZNF224	-0.26637	8.18E-10	3.54E-09
ZNF225	-0.14277	1.16E-03	2.30E-03
ZNF226	-0.11067	1.20E-02	2.00E-02
ZNF227	0.036398	4.10E-01	4.76E-01
ZNF229	0.187091	1.93E-05	4.97E-05
ZNF22	-0.03286	4.57E-01	5.23E-01
ZNF230	-0.08381	5.73E-02	8.35E-02
ZNF232	0.01457	7.42E-01	7.85E-01
ZNF233	-0.11269	1.05E-02	1.77E-02
ZNF234	-0.07327	9.67E-02	1.34E-01
ZNF235	-0.0426	3.35E-01	4.00E-01
ZNF236	-0.10493	1.72E-02	2.79E-02

ZNF238	-0.42119	1.46E-23	2.30E-22
ZNF239	0.206394	2.32E-06	6.72E-06
ZNF23	-0.30881	7.67E-13	4.56E-12
ZNF248	-0.27242	3.25E-10	1.47E-09
ZNF24	-0.10241	2.01E-02	3.22E-02
ZNF250	-0.282	7.18E-11	3.46E-10
ZNF251	-0.11152	1.13E-02	1.91E-02
ZNF252	-0.09668	2.83E-02	4.38E-02
ZNF253	-0.16219	2.19E-04	4.86E-04
ZNF254	-0.24013	3.45E-08	1.24E-07
ZNF256	0.1096	1.28E-02	2.14E-02
ZNF257	0.119558	6.60E-03	1.16E-02
ZNF259	0.399662	3.58E-21	4.63E-20
ZNF25	-0.39173	2.47E-20	2.98E-19
ZNF260	0.181773	3.33E-05	8.28E-05
ZNF263	-0.25504	4.33E-09	1.72E-08
ZNF264	-0.31566	2.23E-13	1.40E-12
ZNF266	-0.19067	1.32E-05	3.48E-05
ZNF267	0.249754	9.17E-09	3.52E-08
ZNF268	0.097206	2.74E-02	4.26E-02
ZNF26	0.202354	3.67E-06	1.04E-05
ZNF271	0.03789	3.91E-01	4.58E-01
ZNF273	0.168017	1.28E-04	2.93E-04
ZNF274	-0.05618	2.03E-01	2.58E-01
ZNF275	-0.18839	1.68E-05	4.37E-05
ZNF276	-0.37191	2.44E-18	2.46E-17
ZNF277	0.010122	8.19E-01	8.51E-01
ZNF280A	0.327288	2.53E-14	1.73E-13
ZNF280B	0.189858	1.44E-05	3.77E-05
ZNF280C	0.165446	1.62E-04	3.67E-04
ZNF280D	-0.26844	5.98E-10	2.63E-09
ZNF281	-0.01659	7.07E-01	7.55E-01
ZNF282	0.093212	3.44E-02	5.25E-02
ZNF283	0.084805	5.44E-02	7.97E-02
ZNF284	0.025796	5.59E-01	6.20E-01
ZNF285	-0.0113	7.98E-01	8.33E-01
ZNF286A	0.277158	1.55E-10	7.25E-10
ZNF286B	0.036325	4.11E-01	4.77E-01
ZNF287	-0.10582	1.63E-02	2.66E-02
ZNF28	0.111154	1.16E-02	1.95E-02
ZNF292	0.070396	1.11E-01	1.51E-01
ZNF295	-0.10203	2.06E-02	3.28E-02
ZNF296	0.001395	9.75E-01	9.81E-01
ZNF2	-0.12028	6.28E-03	1.11E-02

ZNF300	0.21384	9.69E-07	2.94E-06
ZNF302	-0.15807	3.17E-04	6.87E-04
ZNF304	-0.11874	6.98E-03	1.22E-02
ZNF30	-0.02022	6.47E-01	7.01E-01
ZNF311	-0.10366	1.86E-02	3.00E-02
ZNF317	0.013898	7.53E-01	7.94E-01
ZNF318	-0.06218	1.59E-01	2.08E-01
ZNF319	-0.32904	1.81E-14	1.25E-13
ZNF320	-0.17074	9.87E-05	2.30E-04
ZNF321	-0.34924	3.22E-16	2.65E-15
ZNF322A	-0.11785	7.42E-03	1.29E-02
ZNF322B	-0.103	1.94E-02	3.11E-02
ZNF323	-0.25086	7.85E-09	3.03E-08
ZNF324B	-0.10778	1.44E-02	2.38E-02
ZNF324	-0.22641	2.07E-07	6.82E-07
ZNF326	0.215645	7.81E-07	2.40E-06
ZNF329	-0.04074	3.56E-01	4.22E-01
ZNF32	-0.1322	2.65E-03	4.97E-03
ZNF330	-0.12297	5.20E-03	9.30E-03
ZNF331	-0.14287	1.15E-03	2.29E-03
ZNF333	-0.31417	2.92E-13	1.82E-12
ZNF334	-0.16244	2.14E-04	4.76E-04
ZNF335	-0.02904	5.11E-01	5.75E-01
ZNF337	-0.30251	2.33E-12	1.32E-11
ZNF33A	-0.38599	9.63E-20	1.10E-18
ZNF33B	-0.44159	5.43E-26	1.04E-24
ZNF341	0.022437	6.11E-01	6.68E-01
ZNF343	-0.05149	2.43E-01	3.03E-01
ZNF345	-0.26256	1.45E-09	6.07E-09
ZNF346	-0.06675	1.30E-01	1.75E-01
ZNF347	-0.01337	7.62E-01	8.02E-01
ZNF34	-0.30326	2.04E-12	1.17E-11
ZNF350	-0.1884	1.68E-05	4.37E-05
ZNF354A	-0.07561	8.65E-02	1.21E-01
ZNF354B	-0.24673	1.40E-08	5.24E-08
ZNF354C	-0.09747	2.70E-02	4.20E-02
ZNF358	-0.12638	4.07E-03	7.42E-03
ZNF35	0.071837	1.03E-01	1.42E-01
ZNF362	-0.3667	7.77E-18	7.45E-17
ZNF365	0.06481	1.42E-01	1.88E-01
ZNF366	-0.28582	3.87E-11	1.91E-10
ZNF367	0.726804	9.84E-86	1.27E-83
ZNF37A	-0.14787	7.63E-04	1.56E-03
ZNF37B	-0.192	1.15E-05	3.05E-05

ZNF382	0.009967	8.21E-01	8.53E-01
ZNF383	0.027036	5.40E-01	6.03E-01
ZNF384	0.231915	1.02E-07	3.48E-07
ZNF385A	0.119657	6.56E-03	1.15E-02
ZNF385B	-0.43172	8.52E-25	1.48E-23
ZNF385D	-0.12936	3.27E-03	6.06E-03
ZNF389	-0.10133	2.15E-02	3.41E-02
ZNF391	-0.07387	9.40E-02	1.30E-01
ZNF394	-0.11742	7.64E-03	1.32E-02
ZNF395	-0.19606	7.39E-06	2.01E-05
ZNF396	-0.3611	2.63E-17	2.42E-16
ZNF397OS	-0.17193	8.80E-05	2.06E-04
ZNF397	-0.27071	4.23E-10	1.89E-09
ZNF398	-0.02412	5.85E-01	6.44E-01
ZNF3	0.118305	7.19E-03	1.25E-02
ZNF404	-0.09355	3.38E-02	5.16E-02
ZNF407	-0.16992	1.07E-04	2.48E-04
ZNF408	0.037059	4.01E-01	4.68E-01
ZNF410	0.249355	9.70E-09	3.71E-08
ZNF414	-0.12478	4.57E-03	8.26E-03
ZNF415	-0.08916	4.31E-02	6.44E-02
ZNF416	-0.01157	7.93E-01	8.29E-01
ZNF417	-0.15147	5.62E-04	1.17E-03
ZNF418	-0.14598	8.92E-04	1.81E-03
ZNF419	-0.10827	1.40E-02	2.31E-02
ZNF41	-0.13475	2.18E-03	4.15E-03
ZNF420	0.054388	2.18E-01	2.75E-01
ZNF423	-0.30972	6.53E-13	3.91E-12
ZNF425	-0.26677	7.69E-10	3.34E-09
ZNF426	0.02839	5.20E-01	5.84E-01
ZNF428	-0.01325	7.64E-01	8.04E-01
ZNF429	-0.3114	4.83E-13	2.93E-12
ZNF430	0.010454	8.13E-01	8.45E-01
ZNF431	-0.11002	1.25E-02	2.08E-02
ZNF432	-0.11176	1.11E-02	1.88E-02
ZNF433	-0.23129	1.11E-07	3.77E-07
ZNF434	-0.28682	3.29E-11	1.64E-10
ZNF436	-0.00762	8.63E-01	8.88E-01
ZNF438	-0.21038	1.46E-06	4.33E-06
ZNF439	-0.17034	1.02E-04	2.39E-04
ZNF43	-0.13421	2.27E-03	4.30E-03
ZNF440	-0.22051	4.32E-07	1.37E-06
ZNF441	-0.40361	1.34E-21	1.79E-20
ZNF442	-0.28261	6.51E-11	3.15E-10

ZNF443	-0.12023	6.30E-03	1.11E-02
ZNF444	-0.34054	1.90E-15	1.44E-14
ZNF445	-0.15367	4.66E-04	9.83E-04
ZNF446	-0.29783	5.21E-12	2.84E-11
ZNF449	-0.14371	1.07E-03	2.15E-03
ZNF44	-0.39132	2.72E-20	3.28E-19
ZNF451	-0.07275	9.91E-02	1.37E-01
ZNF454	-0.21517	8.27E-07	2.53E-06
ZNF45	-0.01238	7.79E-01	8.17E-01
ZNF460	0.183801	2.71E-05	6.83E-05
ZNF461	0.037474	3.96E-01	4.63E-01
ZNF462	-0.21457	8.89E-07	2.71E-06
ZNF467	-0.19515	8.17E-06	2.21E-05
ZNF468	0.12299	5.19E-03	9.29E-03
ZNF469	0.034377	4.36E-01	5.03E-01
ZNF470	-0.18251	3.09E-05	7.73E-05
ZNF471	-0.24072	3.18E-08	1.15E-07
ZNF473	0.318965	1.21E-13	7.78E-13
ZNF474	-0.22925	1.44E-07	4.84E-07
ZNF479	0.114914	9.05E-03	1.55E-02
ZNF480	0.136862	1.85E-03	3.57E-03
ZNF483	-0.24984	9.07E-09	3.48E-08
ZNF484	-0.196	7.44E-06	2.03E-05
ZNF485	0.066121	1.34E-01	1.79E-01
ZNF486	-0.08367	5.78E-02	8.41E-02
ZNF487	-0.06624	1.33E-01	1.78E-01
ZNF488	0.337091	3.77E-15	2.78E-14
ZNF48	0.128223	3.56E-03	6.54E-03
ZNF490	-0.33268	8.95E-15	6.38E-14
ZNF491	-0.37695	7.82E-19	8.18E-18
ZNF492	0.131022	2.89E-03	5.40E-03
ZNF493	-0.36071	2.86E-17	2.63E-16
ZNF496	0.01962	6.57E-01	7.10E-01
ZNF497	-0.28541	4.14E-11	2.04E-10
ZNF498	0.023059	6.02E-01	6.59E-01
ZNF500	-0.39131	2.73E-20	3.28E-19
ZNF501	-0.06338	1.51E-01	1.99E-01
ZNF502	-0.16022	2.62E-04	5.74E-04
ZNF503	-0.16748	1.34E-04	3.07E-04
ZNF506	-0.16736	1.36E-04	3.10E-04
ZNF507	0.141961	1.24E-03	2.45E-03
ZNF510	-0.16758	1.33E-04	3.04E-04
ZNF511	0.125118	4.46E-03	8.07E-03
ZNF512B	-0.09648	2.86E-02	4.43E-02

ZNF512	-0.15853	3.04E-04	6.60E-04
ZNF513	-0.04326	3.27E-01	3.92E-01
ZNF514	-0.23349	8.32E-08	2.86E-07
ZNF516	-0.22389	2.84E-07	9.22E-07
ZNF517	-0.28118	8.19E-11	3.93E-10
ZNF518A	0.080489	6.80E-02	9.77E-02
ZNF518B	0.126587	4.01E-03	7.31E-03
ZNF519	0.237852	4.68E-08	1.65E-07
ZNF521	-0.19026	1.38E-05	3.62E-05
ZNF524	-0.13783	1.72E-03	3.32E-03
ZNF525	0.265782	8.93E-10	3.84E-09
ZNF526	0.05184	2.40E-01	3.00E-01
ZNF527	-0.00891	8.40E-01	8.69E-01
ZNF528	-0.16388	1.87E-04	4.20E-04
ZNF529	-0.04711	2.86E-01	3.48E-01
ZNF530	0.249201	9.91E-09	3.78E-08
ZNF532	0.11582	8.52E-03	1.46E-02
ZNF534	0.056816	1.98E-01	2.53E-01
ZNF536	-0.38028	3.64E-19	3.93E-18
ZNF540	-0.43841	1.33E-25	2.47E-24
ZNF541	-0.23338	8.44E-08	2.90E-07
ZNF542	0.013322	7.63E-01	8.03E-01
ZNF543	-0.06597	1.35E-01	1.80E-01
ZNF544	0.240644	3.22E-08	1.16E-07
ZNF546	-0.34213	1.38E-15	1.06E-14
ZNF547	-0.22168	3.74E-07	1.20E-06
ZNF548	-0.13713	1.81E-03	3.50E-03
ZNF549	-0.12571	4.27E-03	7.76E-03
ZNF550	-0.08799	4.59E-02	6.83E-02
ZNF551	0.037289	3.98E-01	4.65E-01
ZNF552	-0.36899	4.68E-18	4.57E-17
ZNF554	-0.45781	4.81E-28	1.08E-26
ZNF555	-0.09876	2.50E-02	3.93E-02
ZNF556	0.065536	1.37E-01	1.83E-01
ZNF557	-0.17188	8.84E-05	2.07E-04
ZNF558	0.075849	8.55E-02	1.20E-01
ZNF559	-0.21524	8.20E-07	2.51E-06
ZNF560	0.113669	9.83E-03	1.67E-02
ZNF561	-0.1378	1.72E-03	3.33E-03
ZNF562	0.111985	1.10E-02	1.85E-02
ZNF563	-0.30562	1.35E-12	7.82E-12
ZNF564	-0.4241	6.73E-24	1.09E-22
ZNF565	-0.0192	6.64E-01	7.16E-01
ZNF566	-0.10831	1.39E-02	2.30E-02

ZNF567	0.187339	1.88E-05	4.85E-05
ZNF568	-0.04988	2.59E-01	3.20E-01
ZNF569	0.044223	3.17E-01	3.81E-01
ZNF570	0.076716	8.20E-02	1.15E-01
ZNF571	-0.1659	1.56E-04	3.52E-04
ZNF572	-0.00518	9.07E-01	9.25E-01
ZNF573	-0.15732	3.39E-04	7.30E-04
ZNF574	-0.00649	8.83E-01	9.05E-01
ZNF575	-0.2612	1.77E-09	7.34E-09
ZNF576	0.155578	3.95E-04	8.42E-04
ZNF577	-0.27024	4.54E-10	2.02E-09
ZNF578	-0.00769	8.62E-01	8.87E-01
ZNF579	0.028568	5.18E-01	5.82E-01
ZNF57	-0.21734	6.36E-07	1.98E-06
ZNF580	-0.25832	2.69E-09	1.10E-08
ZNF581	0.049687	2.60E-01	3.22E-01
ZNF582	-0.15927	2.85E-04	6.21E-04
ZNF583	-0.06015	1.73E-01	2.25E-01
ZNF584	-0.12721	3.83E-03	7.01E-03
ZNF585A	0.145276	9.45E-04	1.91E-03
ZNF585B	-0.0136	7.58E-01	7.99E-01
ZNF586	-0.14335	1.11E-03	2.21E-03
ZNF587	-0.10599	1.61E-02	2.63E-02
ZNF589	-0.30793	8.98E-13	5.30E-12
ZNF592	-0.16248	2.13E-04	4.74E-04
ZNF593	0.060255	1.72E-01	2.24E-01
ZNF594	-0.22712	1.89E-07	6.26E-07
ZNF595	-0.03682	4.04E-01	4.71E-01
ZNF596	-0.3265	2.95E-14	2.01E-13
ZNF597	-0.24735	1.28E-08	4.83E-08
ZNF598	0.158676	3.00E-04	6.53E-04
ZNF599	-0.14473	9.89E-04	1.99E-03
ZNF600	-0.06833	1.21E-01	1.64E-01
ZNF605	0.059053	1.81E-01	2.33E-01
ZNF606	-0.0992	2.44E-02	3.83E-02
ZNF607	0.044721	3.11E-01	3.75E-01
ZNF608	-0.34255	1.26E-15	9.76E-15
ZNF609	-0.14285	1.15E-03	2.29E-03
ZNF610	-0.10317	1.92E-02	3.08E-02
ZNF611	-0.04747	2.82E-01	3.44E-01
ZNF613	-0.11476	9.14E-03	1.56E-02
ZNF614	-0.02717	5.38E-01	6.01E-01
ZNF615	-0.24693	1.36E-08	5.10E-08
ZNF616	-0.0079	8.58E-01	8.84E-01

ZNF618	-0.1746	6.79E-05	1.62E-04
ZNF619	-0.05881	1.83E-01	2.35E-01
ZNF620	-0.00833	8.50E-01	8.78E-01
ZNF621	-0.19351	9.76E-06	2.61E-05
ZNF622	0.184936	2.41E-05	6.13E-05
ZNF623	0.112061	1.09E-02	1.84E-02
ZNF624	-0.0628	1.55E-01	2.03E-01
ZNF625	-0.0846	5.50E-02	8.05E-02
ZNF626	-0.16642	1.48E-04	3.37E-04
ZNF627	-0.04439	3.15E-01	3.79E-01
ZNF628	-0.08384	5.73E-02	8.34E-02
ZNF629	-0.2694	5.17E-10	2.29E-09
ZNF630	-0.08139	6.49E-02	9.37E-02
ZNF638	-0.13198	2.69E-03	5.05E-03
ZNF639	0.396199	8.37E-21	1.05E-19
ZNF641	-0.07164	1.04E-01	1.43E-01
ZNF642	0.02896	5.12E-01	5.76E-01
ZNF643	0.289865	1.99E-11	1.02E-10
ZNF644	0.036124	4.13E-01	4.80E-01
ZNF645	-0.05584	2.06E-01	2.61E-01
ZNF646	-0.14359	1.08E-03	2.17E-03
ZNF648	0.114969	9.02E-03	1.54E-02
ZNF649	-0.10002	2.32E-02	3.67E-02
ZNF652	-0.2255	2.32E-07	7.61E-07
ZNF653	-0.17972	4.09E-05	1.01E-04
ZNF654	-0.12943	3.26E-03	6.03E-03
ZNF655	-0.10215	2.04E-02	3.26E-02
ZNF658	-0.36365	1.51E-17	1.42E-16
ZNF660	-0.13072	2.96E-03	5.51E-03
ZNF662	-0.33202	1.02E-14	7.22E-14
ZNF664	0.063415	1.51E-01	1.99E-01
ZNF665	-0.13277	2.54E-03	4.77E-03
ZNF667	0.001169	9.79E-01	9.84E-01
ZNF668	0.093588	3.37E-02	5.15E-02
ZNF669	0.014225	7.47E-01	7.90E-01
ZNF670	0.146827	8.32E-04	1.69E-03
ZNF671	-0.31678	1.81E-13	1.15E-12
ZNF672	-0.28983	2.00E-11	1.02E-10
ZNF673	-0.09207	3.67E-02	5.57E-02
ZNF674	0.015408	7.27E-01	7.73E-01
ZNF675	0.020014	6.50E-01	7.04E-01
ZNF676	0.048059	2.76E-01	3.38E-01
ZNF677	-0.12845	3.50E-03	6.44E-03
ZNF678	-0.03708	4.01E-01	4.68E-01

ZNF679	0.157696	3.28E-04	7.08E-04
ZNF680	0.163606	1.92E-04	4.30E-04
ZNF681	0.147697	7.74E-04	1.58E-03
ZNF682	-0.25883	2.50E-09	1.02E-08
ZNF683	0.020133	6.49E-01	7.02E-01
ZNF684	-0.05644	2.01E-01	2.56E-01
ZNF687	-0.08016	6.91E-02	9.91E-02
ZNF688	-0.3048	1.56E-12	9.00E-12
ZNF689	-0.03239	4.63E-01	5.29E-01
ZNF691	-0.2146	8.85E-07	2.70E-06
ZNF692	-0.18877	1.62E-05	4.21E-05
ZNF695	0.457231	5.72E-28	1.27E-26
ZNF696	-0.11498	9.01E-03	1.54E-02
ZNF697	0.244198	1.98E-08	7.31E-08
ZNF699	0.065607	1.37E-01	1.83E-01
ZNF69	-0.42572	4.36E-24	7.18E-23
ZNF700	-0.19403	9.22E-06	2.48E-05
ZNF701	0.05988	1.75E-01	2.27E-01
ZNF702P	-0.01967	6.56E-01	7.09E-01
ZNF703	0.07333	9.64E-02	1.33E-01
ZNF704	-0.29899	4.28E-12	2.35E-11
ZNF705A	0.037079	4.01E-01	4.68E-01
ZNF705D	0.074671	9.05E-02	1.26E-01
ZNF706	0.207116	2.13E-06	6.21E-06
ZNF707	-0.14649	8.55E-04	1.74E-03
ZNF708	-0.16923	1.14E-04	2.63E-04
ZNF709	-0.30637	1.18E-12	6.89E-12
ZNF70	-0.16034	2.59E-04	5.68E-04
ZNF710	-0.17874	4.52E-05	1.11E-04
ZNF711	0.029225	5.08E-01	5.72E-01
ZNF713	-0.00289	9.48E-01	9.59E-01
ZNF714	0.045341	3.04E-01	3.68E-01
ZNF716	0.118327	7.18E-03	1.25E-02
ZNF717	-0.05481	2.14E-01	2.71E-01
ZNF718	0.20513	2.68E-06	7.72E-06
ZNF71	0.134503	2.22E-03	4.22E-03
ZNF720	-0.28075	8.77E-11	4.19E-10
ZNF721	-0.13513	2.12E-03	4.03E-03
ZNF727	-0.08061	6.76E-02	9.71E-02
ZNF732	0.056151	2.03E-01	2.59E-01
ZNF735	0.065814	1.36E-01	1.81E-01
ZNF737	-0.22609	2.15E-07	7.10E-07
ZNF738	0.046596	2.91E-01	3.54E-01
ZNF740	-0.06058	1.70E-01	2.21E-01

ZNF746	0.133018	2.49E-03	4.69E-03
ZNF747	-0.13163	2.76E-03	5.17E-03
ZNF749	0.092246	3.64E-02	5.52E-02
ZNF74	-0.02635	5.51E-01	6.12E-01
ZNF750	-0.39694	6.99E-21	8.79E-20
ZNF75A	-0.00438	9.21E-01	9.37E-01
ZNF75D	-0.42129	1.42E-23	2.25E-22
ZNF761	0.139328	1.53E-03	2.98E-03
ZNF763	-0.53075	9.19E-39	3.97E-37
ZNF764	-0.19255	1.08E-05	2.88E-05
ZNF765	0.265502	9.31E-10	4.00E-09
ZNF766	-0.10007	2.31E-02	3.66E-02
ZNF767	-0.24473	1.84E-08	6.81E-08
ZNF768	0.048248	2.74E-01	3.36E-01
ZNF76	-0.24933	9.74E-09	3.72E-08
ZNF770	0.110964	1.17E-02	1.97E-02
ZNF771	0.078087	7.66E-02	1.09E-01
ZNF772	-0.00266	9.52E-01	9.62E-01
ZNF773	-0.11187	1.11E-02	1.87E-02
ZNF774	-0.17586	6.01E-05	1.45E-04
ZNF775	0.075359	8.76E-02	1.22E-01
ZNF776	-0.23416	7.62E-08	2.63E-07
ZNF777	-0.00611	8.90E-01	9.11E-01
ZNF778	-0.40221	1.90E-21	2.51E-20
ZNF77	-0.07343	9.60E-02	1.33E-01
ZNF780A	-0.15261	5.10E-04	1.07E-03
ZNF780B	-0.17777	4.98E-05	1.21E-04
ZNF781	-0.1737	7.42E-05	1.76E-04
ZNF782	-0.24069	3.20E-08	1.15E-07
ZNF784	-0.28221	6.95E-11	3.35E-10
ZNF785	-0.22602	2.17E-07	7.15E-07
ZNF786	0.000846	9.85E-01	9.88E-01
ZNF787	0.070459	1.10E-01	1.50E-01
ZNF788	-0.01873	6.72E-01	7.23E-01
ZNF789	-0.05665	1.99E-01	2.54E-01
ZNF790	-0.16466	1.75E-04	3.93E-04
ZNF791	-0.14806	7.51E-04	1.54E-03
ZNF792	-0.10754	1.46E-02	2.41E-02
ZNF793	0.007824	8.59E-01	8.85E-01
ZNF799	-0.14566	9.16E-04	1.85E-03
ZNF79	-0.1561	3.77E-04	8.07E-04
ZNF7	-0.03831	3.86E-01	4.52E-01
ZNF800	0.071697	1.04E-01	1.43E-01
ZNF804A	0.031394	4.77E-01	5.42E-01

ZNF804B	0.077124	8.04E-02	1.13E-01
ZNF805	-0.1291	3.34E-03	6.16E-03
ZNF808	-0.16946	1.11E-04	2.58E-04
ZNF80	-0.02025	6.47E-01	7.01E-01
ZNF813	0.245285	1.71E-08	6.34E-08
ZNF814	-0.27593	1.88E-10	8.73E-10
ZNF815	-0.16742	1.35E-04	3.09E-04
ZNF816A	-0.18431	2.57E-05	6.50E-05
ZNF81	0.041736	3.45E-01	4.10E-01
ZNF821	-0.12953	3.23E-03	5.99E-03
ZNF823	-0.11841	7.14E-03	1.24E-02
ZNF826	-0.04273	3.33E-01	3.99E-01
ZNF827	-0.00381	9.31E-01	9.45E-01
ZNF828	0.036406	4.10E-01	4.76E-01
ZNF829	0.123222	5.11E-03	9.15E-03
ZNF830	0.002894	9.48E-01	9.59E-01
ZNF831	-0.1944	8.85E-06	2.39E-05
ZNF833	0.126513	4.03E-03	7.35E-03
ZNF835	-0.21902	5.18E-07	1.63E-06
ZNF836	-0.28187	7.34E-11	3.53E-10
ZNF837	-0.20861	1.80E-06	5.28E-06
ZNF839	-0.19642	7.11E-06	1.94E-05
ZNF83	-0.23363	8.16E-08	2.81E-07
ZNF841	-0.08785	4.63E-02	6.88E-02
ZNF843	-0.16231	2.16E-04	4.81E-04
ZNF844	-0.24133	2.93E-08	1.06E-07
ZNF845	0.180185	3.91E-05	9.64E-05
ZNF846	-0.38885	4.91E-20	5.74E-19
ZNF84	-0.01266	7.74E-01	8.12E-01
ZNF853	-0.06126	1.65E-01	2.15E-01
ZNF85	0.030558	4.89E-01	5.54E-01
ZNF860	0.099958	2.33E-02	3.68E-02
ZNF862	-0.30986	6.36E-13	3.82E-12
ZNF876P	0.150001	6.38E-04	1.32E-03
ZNF878	0.099412	2.41E-02	3.79E-02
ZNF879	-0.17567	6.12E-05	1.47E-04
ZNF880	-0.05243	2.35E-01	2.94E-01
ZNF883	0.238834	4.10E-08	1.46E-07
ZNF8	0.175939	5.96E-05	1.44E-04
ZNF90	-0.01539	7.28E-01	7.73E-01
ZNF91	-0.22331	3.05E-07	9.88E-07
ZNF92	0.316883	1.78E-13	1.13E-12
ZNF93	0.271717	3.62E-10	1.63E-09
ZNF98	0.143836	1.06E-03	2.13E-03

ZNF99	0.016078	7.16E-01	7.63E-01
ZNFX1	-0.03301	4.55E-01	5.21E-01
ZNHIT1	0.075775	8.58E-02	1.20E-01
ZNHIT2	-0.16962	1.10E-04	2.55E-04
ZNHIT3	0.299606	3.85E-12	2.13E-11
ZNHIT6	0.144584	1.00E-03	2.01E-03
ZNRD1	0.151922	5.41E-04	1.13E-03
ZNRF1	0.003566	9.36E-01	9.49E-01
ZNRF2	0.140417	1.40E-03	2.75E-03
ZNRF3	-0.29645	6.60E-12	3.55E-11
ZNRF4	0.07814	7.64E-02	1.09E-01
ZP1	0.149068	6.90E-04	1.42E-03
ZP2	-0.1681	1.27E-04	2.91E-04
ZP3	0.315057	2.49E-13	1.55E-12
ZP4	0.08612	5.08E-02	7.48E-02
ZPBP2	0.048859	2.68E-01	3.30E-01
ZPBP	-0.14564	9.17E-04	1.85E-03
ZPLD1	0.113843	9.72E-03	1.65E-02
ZRANB1	-0.13461	2.20E-03	4.19E-03
ZRANB2	-0.07977	7.05E-02	1.01E-01
ZRANB3	0.361185	2.58E-17	2.38E-16
ZRSR2	-0.31281	3.74E-13	2.29E-12
ZSCAN10	0.044403	3.15E-01	3.79E-01
ZSCAN12P1	0.094217	3.25E-02	4.99E-02
ZSCAN12	-0.06958	1.15E-01	1.56E-01
ZSCAN16	-0.07861	7.47E-02	1.06E-01
ZSCAN18	-0.20356	3.20E-06	9.15E-06
ZSCAN1	-0.09262	3.56E-02	5.42E-02
ZSCAN20	0.10464	1.75E-02	2.84E-02
ZSCAN21	0.233342	8.48E-08	2.92E-07
ZSCAN22	-0.14881	7.05E-04	1.45E-03
ZSCAN23	-0.08474	5.46E-02	7.99E-02
ZSCAN29	-0.02695	5.42E-01	6.04E-01
ZSCAN2	-0.0556	2.08E-01	2.64E-01
ZSCAN4	-0.38588	9.88E-20	1.13E-18
ZSCAN5A	0.035662	4.19E-01	4.86E-01
ZSCAN5B	0.044871	3.09E-01	3.74E-01
ZSWIM1	0.019699	6.56E-01	7.09E-01
ZSWIM2	0.043805	3.21E-01	3.86E-01
ZSWIM3	0.035827	4.17E-01	4.84E-01
ZSWIM4	-0.08011	6.93E-02	9.93E-02
ZSWIM5	-0.24519	1.73E-08	6.42E-08
ZSWIM6	-0.07664	8.23E-02	1.16E-01
ZSWIM7	-0.37581	1.01E-18	1.05E-17

ZUFSP	0.396938	6.99E-21	8.79E-20
ZW10	0.313678	3.20E-13	1.98E-12
ZWILCH	0.70665	4.23E-79	5.12E-77
ZWINT	0.840954	6.24E-139	2.31E-136
ZXDA	-0.20276	3.51E-06	9.97E-06
ZXDB	-0.04364	3.23E-01	3.88E-01
ZXDC	-0.3163	1.98E-13	1.25E-12
ZYG11A	0.36114	2.61E-17	2.40E-16
ZYG11B	-0.16444	1.78E-04	4.00E-04
ZYX	0.078794	7.40E-02	1.05E-01
ZZEF1	-0.27118	3.93E-10	1.76E-09
ZZZ3	0.2385	4.29E-08	1.52E-07
psiTPTE22	-0.16955	1.10E-04	2.56E-04
tAKR	-0.0285	5.19E-01	5.83E-01

FDR (BH): FDR is calculated by BH (Benjamini-Hochberg method); FDR: False discovery rate.

Table S2. Gene Ontology term annotation of *RRM2* co-expressed genes.

Gene set	Description	Size	Leading edge number	ES	NES	P-value	FDR
GO:0048285	organelle fission	404	101	0.86358	2.5404	0	0
GO:0044772	mitotic cell cycle phase transition	453	146	0.81304	2.3638	0	0
GO:0006310	DNA recombination	233	72	0.80766	2.2595	0	0
GO:0045930	negative regulation of mitotic cell cycle	237	53	0.78256	2.181	0	0
GO:0051052	regulation of DNA metabolic process	367	96	0.74623	2.1713	0	0
GO:0006333	chromatin assembly or disassembly	139	43	0.80094	2.1389	0	0
GO:0034394	protein localization to cell surface	56	14	-0.67878	-1.9092	0	0.00809
GO:0032409	regulation of transporter activity	231	77	-0.53302	-1.8228	0	0.015371
GO:0007588	excretion	65	23	-0.59631	-1.7008	0	0.039583
GO:0001539	cilium or flagellum-dependent cell motility	25	18	-0.71444	-1.7026	0.002299	0.044495
GO:0097503	sialylation	20	10	-0.71639	-1.6629	0.008909	0.056154
GO:1903034	regulation of response to wounding	145	38	-0.5074	-1.633	0	0.056318
GO:0003007	heart morphogenesis	244	62	-0.47603	-1.6391	0	0.058476
GO:0046717	acid secretion	107	22	-0.53499	-1.6363	0	0.05882

ES: Enrichment score; NES: Normalized enrichment score; FDR: false discovery rate.

Table S3. KEGG annotation of *RRM2* co-expressed genes.

Gene set	Description	Size	Leading edge number	ES	NES	P-value	FDR
hsa03460	Fanconi anemia pathway	44	18	0.87513	2.0316	0	0
hsa03013	RNA transport	158	65	0.72762	1.9883	0	0
hsa04114	Oocyte meiosis	118	24	0.74222	1.9576	0	0
hsa03040	Spliceosome	115	62	0.73022	1.9153	0	0
hsa03008	Ribosome biogenesis in eukaryotes	70	39	0.77639	1.9144	0	0
hsa04218	Cellular senescence	155	27	0.70545	1.9087	0	0
hsa03420	Nucleotide excision repair	45	17	0.74844	1.7281	0.001623	0.005809
hsa05150	Staphylococcus aureus infection	52	21	-0.62811	-1.7224	0.005602	0.010716
hsa03320	PPAR signaling pathway	74	20	-0.59211	-1.7246	0	0.011535
hsa05169	Epstein-Barr virus infection	195	41	0.60869	1.6843	0	0.012572
hsa00980	Metabolism of xenobiotics by cytochrome P450	70	23	-0.58242	-1.7275	0	0.013874
hsa00591	Linoleic acid metabolism	27	12	-0.68288	-1.658	0.00978	0.019388
hsa04142	Lysosome	121	37	-0.53627	-1.6507	0.003937	0.019535
hsa03010	Ribosome	131	38	0.61688	1.6429	0.001326	0.020533
hsa00670	One carbon pool by folate	18	8	0.79898	1.638	0.003677	0.02141
hsa00340	Histidine metabolism	21	9	-0.71719	-1.6607	0.004474	0.021768
hsa04710	Circadian rhythm	30	9	-0.6768	-1.6661	0.009662	0.022725
hsa05217	Basal cell carcinoma	63	26	-0.58395	-1.6733	0.005814	0.023024
hsa04925	Aldosterone synthesis and secretion	96	30	-0.53531	-1.6213	0	0.02707
hsa04120	Ubiquitin mediated proteolysis	133	28	0.6067	1.6121	0.002699	0.029915
hsa00970	Aminoacyl-tRNA biosynthesis	43	19	0.70604	1.6072	0.010399	0.030213

KEGG: Kyoto Encyclopedia of Genes and Genomes; ES: Enrichment score; NES: Normalized enrichment score; FDR: false discovery rate.

Table S4. Kinases enrichment of *RRM2* co-expressed genes.

Gene Set	Description	Size	Leading Edge Number	ES	NES	P-value	FDR
Kinase_CDK1	cyclin dependent kinase 1	258	67	0.83483	2.3829	0	0
Kinase_PLK1	polo like kinase 1	90	31	0.8895	2.2374	0	0
Kinase_CDK2	cyclin dependent kinase 2	278	84	0.78149	2.2281	0	0
Kinase_AURKB	aurora kinase B	87	31	0.84895	2.1503	0	0
Kinase_ATM	ATM serine/threonine kinase	123	38	0.7934	2.1011	0	0
Kinase_CHEK1	checkpoint kinase 1	130	28	0.78093	2.0964	0	0
Kinase_ATR	ATR serine/threonine kinase	66	20	0.83212	2.0465	0	0
Kinase_AURKA	aurora kinase A	46	12	0.85856	1.9673	0	0
Kinase_CSNK2A1	casein kinase 2 alpha 1	256	65	0.6806	1.9275	0	0
Kinase_CHEK2	checkpoint kinase 2	27	10	0.88588	1.8984	0	0.000122
Kinase_PLK3	polo like kinase 3	20	8	0.88627	1.7915	0.001845	0.001555

Kinase_TTK	TTK protein kinase	12	6	0.89727	1.7361	0.001898	0.005802
Kinase_PAK1	p21 (RAC1) activated kinase 1	50	11	0.72878	1.7136	0.001495	0.009395
Kinase_CDK7	cyclin dependent kinase 7	18	6	0.84243	1.688	0.001901	0.015704
Kinase_MAP3K8	mitogen-activated protein kinase kinase kinase 8	19	4	0.83713	1.6657	0.003718	0.024102
Kinase_RPS6KA1	ribosomal protein S6 kinase A1	46	13	0.71927	1.657	0.004552	0.027787
Kinase_RPS6KA3	ribosomal protein S6 kinase A3	38	7	0.74333	1.6408	0.003384	0.037001
Kinase_MAPK1	mitogen-activated protein kinase 1	197	22	0.5765	1.599	0	0.070705
Kinase_FGFR1	fibroblast growth factor receptor 1	7	2	0.90848	1.5455	0.023392	0.10386
Kinase_BUB1	BUB1 mitotic checkpoint serine/threonine kinase	5	3	0.95628	1.5719	0.008457	0.10408
Kinase_RPS6KA6	ribosomal protein S6 kinase A6	7	3	0.89836	1.5457	0.020921	0.10766
Kinase_WEE1	WEE1 G2 checkpoint kinase	5	4	0.96188	1.553	0.005758	0.10932
Kinase_STK3	serine/threonine kinase 3	7	1	0.91657	1.5653	0.030303	0.10974
Kinase_FYN	FYN proto-oncogene, Src family tyrosine kinase	66	20	-0.3989	-1.162	0.19591	0.7105

Kinase_HCK	HCK proto-oncogene, Src family tyrosine kinase	23	12	-0.49353	-1.1642	0.23918	0.72517
Kinase_BTK	Bruton tyrosine kinase	9	5	-0.60387	-1.1494	0.30528	0.72728
Kinase_NTRK2	neurotrophic receptor tyrosine kinase 2	11	5	-0.59405	-1.1677	0.2963	0.73643
Kinase_MAPK7	mitogen-activated protein kinase 7	30	11	-0.48253	-1.1733	0.22912	0.74096
Kinase_PDGFRA	platelet derived growth factor receptor alpha	8	3	-0.63767	-1.1745	0.27237	0.76156
Kinase_PRKACB	protein kinase cAMP-activated catalytic subunit beta	85	18	-0.37526	-1.1194	0.24088	0.77276
Kinase_CDK16	cyclin dependent kinase 16	3	1	-0.76116	-1.1257	0.35327	0.77631
Kinase_TGFBR2	transforming growth factor beta receptor 2	6	2	-0.82862	-1.4245	0.041199	0.77761

Kinase_CSNK1G3	casein kinase 1 gamma 3	4	2	-0.75004	-1.1755	0.28988	0.78429
Kinase_ARAF	A-Raf proto-oncogene, serine/threonine kinase	13	2	-0.52585	-1.0932	0.37603	0.78727
Kinase_TRPM7	transient receptor potential cation channel subfamily M member 7	5	1	-0.70185	-1.182	0.29608	0.79025
Kinase_PRKACG	protein kinase cAMP-activated catalytic subunit gamma	84	17	-0.36713	-1.0959	0.28881	0.79995
Kinase_DYRK1A	dual specificity tyrosine phosphorylation regulated kinase 1A	14	6	-0.56283	-1.1851	0.25572	0.80808
Kinase_NLK	nemo like kinase	10	5	-0.56147	-1.0711	0.38086	0.80938
Kinase_PDPK1	3-phosphoinositide dependent protein kinase 1	21	4	-0.47421	-1.0976	0.3136	0.81632
Kinase_MAPK10	mitogen-activated protein kinase 10	25	10	-0.43041	-1.0399	0.38717	0.8177

Kinase_PTK2B	protein tyrosine kinase 2 beta	6	3	-0.62029	-1.072	0.42717	0.82659
Kinase_FER	FER tyrosine kinase	8	3	-0.56414	-1.0415	0.43539	0.83165
Kinase_RET	ret proto-oncogene	16	2	-0.4773	-1.0488	0.41357	0.83196
Kinase_SYK	spleen associated tyrosine kinase	35	12	-0.47061	-1.1854	0.21772	0.83825
Kinase_PIK3CA	phosphatidylinositol-4,5-bisphosphate 3-kinase catalytic subunit alpha	10	2	-0.54466	-1.0488	0.41748	0.85124

ES: Enrichment score; NES: Normalized enrichment score; FDR: false discovery rate.

Table S5. miRNA enrichment of *RRM2* co-expressed genes.

Gene Set	Size	Leading Edge Number	ES	NES	P Value	FDR
AGCGCTT,MIR-518F,MIR-518E,MIR-518A	16	5	-0.64667	-1.4072	0.078351	0.48209
CCCAGAG,MIR-326	141	30	-0.4352	-1.4088	0.009009	0.55154
AGGGCAG,MIR-18A	130	34	-0.45477	-1.4499	0	0.55987
GAGCTGG,MIR-337	147	34	-0.38668	-1.2417	0.060086	0.61109
ACACTGG,MIR-199A,MIR-199B	146	36	-0.40024	-1.3139	0.021552	0.61239
GGGGCCC,MIR-296	67	13	-0.44139	-1.2734	0.075075	0.61662
GTCAGGA,MIR-378	53	8	-0.45731	-1.2484	0.12857	0.61763
ACGCACA,MIR-210	7	4	-0.67927	-1.2329	0.23183	0.61779
AAGCAAT,MIR-137	201	45	-0.38484	-1.3022	0.016949	0.61865
GACAGGG,MIR-339	61	16	-0.44782	-1.2816	0.089172	0.61899
ATGCTGG,MIR-338	104	22	-0.42112	-1.2894	0.059289	0.62791
GTGCCAA,MIR-96	284	74	-0.40314	-1.4143	0	0.63097
GTGCAAA,MIR-507	119	43	-0.40634	-1.2611	0.069959	0.63164
GCAAGAC,MIR-431	43	7	-0.46626	-1.2494	0.1383	0.64669
AGCATTA,MIR-155	128	30	-0.33893	-1.0667	0.29555	0.6531
GGTAACC,MIR-409-5P	27	9	-0.447	-1.0724	0.35452	0.65778
GACAATC,MIR-219	134	48	-0.40935	-1.3154	0.020325	0.6603
AAAGACA,MIR-511	182	41	-0.32242	-1.0745	0.19681	0.66363
CCACACA,MIR-147	57	18	-0.42708	-1.1825	0.17417	0.66442
AGCGCAG,MIR-191	12	9	-0.53645	-1.0667	0.38528	0.66556
AACTGGA,MIR-145	215	69	-0.34285	-1.1758	0.059406	0.66748
CTCTATG,MIR-368	37	10	-0.4189	-1.0756	0.34663	0.67332
CCTGTGA,MIR-513	118	36	-0.46144	-1.4589	0.01476	0.67989
CTCTAGA,MIR-526C,MIR-518F,MIR-526A	51	8	0.2651	0.62844	0.9611	0.99051
GTAAGAT,MIR-200A	45	10	0.27813	0.64602	0.94219	0.99379
CGCAAAA,MIR-450	8	3	0.29637	0.52927	0.96473	0.99638
TACAATC,MIR-508	58	17	0.27956	0.66088	0.96094	0.99672
TACTTGA,MIR-26A,MIR-26B	285	43	0.46441	1.3222	0.027907	1
GACTGTT,MIR-212,MIR-132	148	27	0.48862	1.3026	0.058154	1
GTGGTGA,MIR-197	67	17	0.53601	1.3237	0.089686	1
CAGGTCC,MIR-492	59	12	0.49068	1.1819	0.22755	1

AGTCTTA,MIR-499	68	20	0.47601	1.1772	0.24554	1
ATAACCT,MIR-154	60	10	0.48592	1.1577	0.26042	1
ATTCTTT,MIR-186	252	50	0.39358	1.104	0.27939	1
GAGACTG,MIR-452	91	18	0.44487	1.1238	0.28068	1
TAGCTTT,MIR-9	224	31	0.39255	1.0988	0.28159	1
AGTCTAG,MIR-151	21	7	0.58054	1.1664	0.30521	1
TCTGGAC,MIR-198	82	14	0.43097	1.0979	0.3125	1
TCGATGG,MIR-213	5	1	0.71651	1.1698	0.32438	1
CAGCAGG,MIR-370	138	24	0.4084	1.0868	0.32634	1
AGGGCCA,MIR-328	73	16	0.4467	1.094	0.32735	1
CTGTTAC,MIR-194	97	14	0.41962	1.0821	0.33099	1
GTAGGCA,MIR-189	27	5	0.51842	1.1038	0.33798	1
AAACCAC,MIR-140	100	19	0.41019	1.0627	0.35714	1

ES: Enrichment score; NES: Normalized enrichment score; FDR: false discovery rate.

Table S6. Transcription factor enrichment of *RRM2* co-expressed genes.

Gene Set	Size	Leading Edge Number	ES	NES	P Value	FDR
V$E2F_Q6	211	87	0.785	2.1966	0	0
V$E2F_Q4	212	87	0.78317	2.1905	0	0
V$E2F1_Q6	213	91	0.7796	2.1891	0	0
V$E2F4DP1_01	220	91	0.77488	2.1752	0	0
V$E2F1DP1_01	218	90	0.77227	2.1731	0	0
V$E2F1DP2_01	218	90	0.77227	2.1731	0	0
V$E2F4DP2_01	218	90	0.77227	2.1731	0	0
V$E2F_02	218	90	0.7726	2.1717	0	0
V$E2F_Q3	204	72	0.7749	2.1687	0	0
V$E2F_Q3_01	215	81	0.76436	2.1637	0	0
V$E2F_Q4_01	215	78	0.76205	2.142	0	0
V$E2F1_Q4_01	210	79	0.758	2.1365	0	0
V$E2F1_Q3	225	80	0.75715	2.1273	0	0
V$E2F1DP1RB_01	210	78	0.76148	2.1234	0	0
V$E2F_Q6_01	219	70	0.75336	2.1228	0	0
V$E2F1_Q6_01	218	80	0.75893	2.1214	0	0
SGCGSSAAA_V$E2F1DP2_01	155	71	0.77674	2.1095	0	0
V$E2F_03	224	70	0.71575	2.0304	0	0
V$E2F_01	64	27	0.77208	1.8783	0	0
V$E2F1_Q4	222	52	0.66606	1.8505	0	0
RRCCGTTA_UNKNOWN	78	24	0.71265	1.7656	0	0.000496
V$ELK1_02	226	69	0.62339	1.744	0	0.000948
TCCCRNNRTGC_UNKNOWN	193	46	0.59797	1.6607	0	0.004716
ACTAYRNNNCCCR_UNKNOWN	408	79	0.56205	1.6486	0	0.005898
V$RSRFC4_Q2	198	46	-0.49165	-1.6462	0	0.04539
V$FREAC4_01	140	38	-0.5024	-1.6189	0	0.045556
V$DBP_Q6	227	46	-0.49205	-1.6654	0	0.047884
TGCCAAR_V$NF1_Q6	660	147	-0.42171	-1.5855	0	0.049956
V$PITX2_Q2	234	45	-0.47236	-1.62	0	0.050129
V$GATA_Q6	185	51	-0.48818	-1.6493	0	0.050278
TGTTTGY_V$HNF3_Q6	670	161	-0.42821	-1.5945	0	0.052509

V$MEF2_01	134	32	-0.49545	-1.5892	0.008403	0.052623
V$GATA1_04	233	48	-0.47387	-1.6248	0	0.053014
TGGNNNNNNKCCAR_UNKNOWN	387	104	-0.44683	-1.6002	0	0.053271
V$RSRFC4_01	227	52	-0.49853	-1.6786	0	0.054867
TGTYNNNNNRGCARM_UNKNOWN	81	24	-0.5228	-1.5592	0.00361	0.055927
V$MEF2_02	207	52	-0.50548	-1.6957	0	0.056862
V$PAX4_02	218	48	-0.46434	-1.5596	0	0.059456
V$FOXO1_01	221	67	-0.45389	-1.56	0	0.063275
V$GATA3_01	227	54	-0.4511	-1.5342	0	0.068002
V$RP58_01	194	45	-0.45636	-1.5373	0	0.069009
V$CREBP1_01	165	43	-0.46521	-1.5261	0	0.070251
V$MYOGENIN_Q6	235	62	-0.44337	-1.5227	0	0.070629
V$FOXO1_02	226	69	-0.43889	-1.5164	0	0.072111
V$LMO2COM_02	231	78	-0.44297	-1.5085	0	0.075907
V$SRY_01	204	77	-0.42752	-1.4565	0	0.07852
V$TBP_01	227	63	-0.42622	-1.4543	0	0.078579

ES: Enrichment score; NES: Normalized enrichment score; FDR: false discovery rate.

Table S7. Correlation of *RRM2* with various immune signatures in LUAD.

Marker gene	Class	None adjusted		Tumor purity adjusted	
		Correlation coefficient	P-value	Correlation coefficient	P-value
ADRM1	Activated CD8 T cell	0.360147979	3.228E-17	0.351837012	8.208E-16
AHSA1	Activated CD8 T cell	0.382833693	0.000E+00	0.373605129	8.909E-18
C1GALT1C1	Activated CD8 T cell	0.103701508	1.857E-02	0.111138025	1.355E-02
CCT6B	Activated CD8 T cell	-0.241358995	3.230E-08	-0.253440203	1.154E-08
CD37	Activated CD8 T cell	-0.230337192	1.360E-07	-0.255277093	8.966E-09
CD3D	Activated CD8 T cell	0.023145162	6.002E-01	0.039896749	3.767E-01
CD3E	Activated CD8 T cell	-0.043120291	3.288E-01	-0.036851024	4.143E-01
CD3G	Activated CD8 T cell	0.049746543	2.598E-01	0.073297842	1.040E-01
CD69	Activated CD8 T cell	-0.127790219	3.696E-03	-0.136689616	2.353E-03
CD8A	Activated CD8 T cell	0.148752079	7.169E-04	0.174652611	9.696E-05
CETN3	Activated CD8 T cell	-0.050750054	2.503E-01	-0.058872142	1.919E-01
CSE1L	Activated CD8 T cell	0.616605359	0.000E+00	0.621608774	4.870E-54
GEMIN6	Activated CD8 T cell	0.4567221	0.000E+00	0.456518947	9.448E-27
GNLY	Activated CD8 T cell	0.166117287	1.556E-04	0.177993374	7.072E-05
GPT2	Activated CD8 T cell	0.086827533	4.894E-02	0.084988454	5.934E-02
GZMA	Activated CD8 T cell	0.171525399	9.364E-05	0.201576591	6.460E-06
GZMH	Activated CD8 T cell	0.121145299	5.938E-03	0.139986904	1.835E-03
GZMK	Activated CD8 T cell	-0.01629093	7.122E-01	-0.000579438	9.898E-01
IL2RB	Activated CD8 T cell	0.196431637	7.402E-06	0.233795446	1.514E-07
LCK	Activated CD8 T cell	0.059994749	1.740E-01	0.079489982	7.785E-02
MPZL1	Activated CD8 T cell	0.038492936	3.832E-01	0.050876085	2.595E-01
NKG7	Activated CD8 T cell	0.176754161	5.501E-05	0.208605072	2.990E-06
PIK3IP1	Activated CD8 T cell	-0.412759035	1.321E-22	-0.419274053	2.074E-22

PTRH2	Activated CD8 T cell	0.41532741	6.796E-23	0.42011879	1.676E-22
TIMM13	Activated CD8 T cell	0.146635857	8.448E-04	0.130921757	3.590E-03
ZAP70	Activated CD8 T cell	-0.073759261	9.450E-02	-0.084591416	6.054E-02
ACTN4	Central memory CD8 T cell	0.233436596	8.375E-08	0.22173301	6.599E-07
ADAM12	Central memory CD8 T cell	0.407463191	0.000E+00	0.438356246	1.444E-24
ADCY9	Central memory CD8 T cell	-0.311383724	4.838E-13	-0.327423576	8.779E-14
F13A1	Central memory CD8 T cell	-0.088313319	4.518E-02	-0.079696434	7.708E-02
FCER1G	Central memory CD8 T cell	0.125698815	4.277E-03	0.154425721	5.801E-04
FCGR3B	Central memory CD8 T cell	0.136869926	1.851E-03	0.15094456	7.730E-04
FGF7	Central memory CD8 T cell	-0.096804577	2.808E-02	-0.094579931	3.578E-02
FKBP4	Central memory CD8 T cell	0.49206522	9.297E-33	0.490250046	3.584E-31
GLUD1	Central memory CD8 T cell	0.065371013	1.385E-01	0.066018504	1.433E-01
GM2A	Central memory CD8 T cell	0.102457848	2.004E-02	0.117740794	8.877E-03
GUSB	Central memory CD8 T cell	-0.084930679	5.410E-02	-0.102950659	2.225E-02
IL1RN	Central memory CD8 T cell	0.148551157	7.290E-04	0.156662848	4.808E-04
NOL11	Central memory CD8 T cell	0.588920798	2.136E-49	0.586607732	6.583E-47
NTRK1	Central memory CD8 T cell	-0.068487717	1.206E-01	-0.08268745	6.659E-02
RARA	Central memory CD8 T cell	-0.113982656	9.662E-03	-0.131681108	3.399E-03
RNF128	Central memory CD8 T cell	-0.057455592	1.929E-01	-0.073363198	1.037E-01
SIGLEC1	Central memory CD8 T cell	0.015274636	7.294E-01	0.025050889	5.790E-01
TNFRSF11A	Central memory CD8 T cell	0.111769837	1.114E-02	0.111166347	1.352E-02
TOX4	Central memory CD8 T cell	0.198328579	6.018E-06	0.18380612	4.029E-05
UBA52	Central memory CD8 T cell	-0.033153438	4.527E-01	-0.042816395	3.428E-01
ULBP1	Central memory CD8 T cell	0.212393466	1.221E-06	0.190643828	2.032E-05
ACAP1	Effector memory CD8 T cell	-0.096112993	2.919E-02	-0.105099189	1.959E-02
APOL3	Effector memory CD8 T cell	0.046828693	2.887E-01	0.07169997	1.118E-01

ARHGAP10	Effector memory CD8 T cell	0.211813894	1.307E-06	0.211043828	2.274E-06
ATP10D	Effector memory CD8 T cell	0.152202454	5.358E-04	0.167945969	1.795E-04
C3AR1	Effector memory CD8 T cell	0.057799146	1.903E-01	0.08210883	6.852E-02
CCR5	Effector memory CD8 T cell	0.063536314	1.499E-01	0.093098352	3.879E-02
CD160	Effector memory CD8 T cell	-0.048398818	2.728E-01	-0.053881382	2.324E-01
CD55	Effector memory CD8 T cell	-0.322937431	7.785E-14	-0.34093521	6.958E-15
CFLAR	Effector memory CD8 T cell	0.134204569	2.273E-03	0.145269031	1.219E-03
CMKLR1	Effector memory CD8 T cell	0.023379996	5.966E-01	0.041262358	3.606E-01
DAPP1	Effector memory CD8 T cell	0.116752954	8.027E-03	0.131327144	3.487E-03
FCRL6	Effector memory CD8 T cell	-0.135143856	2.132E-03	-0.143210445	1.432E-03
FLT3LG	Effector memory CD8 T cell	-0.115497345	8.735E-03	-0.112334502	1.257E-02
GZMM	Effector memory CD8 T cell	0.002455605	9.557E-01	-0.007895038	8.612E-01
HAPLN3	Effector memory CD8 T cell	0.325351357	3.663E-14	0.381358332	1.634E-18
HLA-DMB	Effector memory CD8 T cell	-0.172541605	8.498E-05	-0.175518258	8.940E-05
HLA-DPA1	Effector memory CD8 T cell	-0.25197869	7.566E-09	-0.25862062	5.638E-09
HLA-DPB1	Effector memory CD8 T cell	-0.330364083	1.403E-14	-0.347789815	1.833E-15
IFI16	Effector memory CD8 T cell	0.216191216	7.788E-07	0.242541383	4.946E-08
LIME1	Effector memory CD8 T cell	-0.126836037	3.961E-03	-0.121362204	6.980E-03
LTK	Effector memory CD8 T cell	0.017947064	6.844E-01	-0.001913152	9.662E-01
NFKBIA	Effector memory CD8 T cell	-0.075314978	8.774E-02	-0.080789768	7.310E-02
SETD7	Effector memory CD8 T cell	0.176385446	5.705E-05	0.181526175	5.034E-05
SIK1	Effector memory CD8 T cell	-0.126428887	4.057E-03	-0.140279604	1.795E-03
TRIB2	Effector memory CD8 T cell	0.091361938	3.824E-02	0.093448621	3.806E-02
AIM2	Activated CD4 T cell	0.217340345	6.787E-07	0.252603654	1.293E-08
BIRC3	Activated CD4 T cell	0.119637157	6.564E-03	0.142376256	1.528E-03
BRIP1	Activated CD4 T cell	0.768578602	0.000E+00	0.766278594	2.186E-96

CCL20	Activated CD4 T cell	0.159605425	2.763E-04	0.169788161	1.519E-04
CCL4	Activated CD4 T cell	0.278366854	1.282E-10	0.318943179	4.046E-13
CCL5	Activated CD4 T cell	0.117288514	7.712E-03	0.14391605	1.355E-03
CCNB1	Activated CD4 T cell	0.873300224	0.000E+00	0.874181398	3.775E-156
CCR7	Activated CD4 T cell	-0.177681412	5.157E-05	-0.200038396	7.619E-06
DUSP2	Activated CD4 T cell	0.051258288	2.455E-01	0.053398753	2.366E-01
ESCO2	Activated CD4 T cell	0.839766987	3.570E-138	0.844636638	2.434E-135
ETS1	Activated CD4 T cell	0.030789903	4.855E-01	0.04629906	3.049E-01
EXO1	Activated CD4 T cell	0.870028983	0.000E+00	0.868718344	6.311E-152
EXOC6	Activated CD4 T cell	0.170462454	1.036E-04	0.157850343	4.348E-04
IARS	Activated CD4 T cell	0.445665692	1.695E-26	0.451816198	3.577E-26
ITK	Activated CD4 T cell	-0.043112208	3.287E-01	-0.038810047	3.899E-01
KIF11	Activated CD4 T cell	0.855602771	8.079E-149	0.858239682	2.474E-144
KNTC1	Activated CD4 T cell	0.681038838	0.000E+00	0.681450558	1.436E-68
NUF2	Activated CD4 T cell	0.819107088	0.000E+00	0.821711725	5.488E-122
PRC1	Activated CD4 T cell	0.855665236	7.292E-149	0.862049011	5.077E-147
PSAT1	Activated CD4 T cell	0.494258219	0.000E+00	0.489858727	4.060E-31
RGS1	Activated CD4 T cell	0.032635012	4.598E-01	0.035253737	4.348E-01
RTKN2	Activated CD4 T cell	0.213883483	9.643E-07	0.204912748	4.496E-06
SAMSN1	Activated CD4 T cell	0.064908592	1.413E-01	0.090501533	4.459E-02
SELL	Activated CD4 T cell	-0.031476746	4.760E-01	-0.028099547	5.336E-01
TRAT1	Activated CD4 T cell	-0.043023392	3.298E-01	-0.032930997	4.657E-01
ABHD3	Central memory CD4 T cell	0.293903728	1.273E-11	0.29717394	1.647E-11
AHNAK	Central memory CD4 T cell	-0.113818633	9.767E-03	-0.115885258	1.002E-02
ANXA2P2	Central memory CD4 T cell	0.181187662	3.639E-05	0.195696521	1.206E-05
AQP3	Central memory CD4 T cell	-0.500860365	4.674E-34	-0.502918301	5.791E-33

ATHL1	Central memory CD4 T cell	-0.164878195	1.744E-04	-0.177306024	7.550E-05
BMI1	Central memory CD4 T cell	0.149813354	6.559E-04	0.159119758	3.901E-04
BZW2	Central memory CD4 T cell	0.427667311	0.000E+00	0.418262908	2.675E-22
CD63	Central memory CD4 T cell	-0.271010016	4.036E-10	-0.266937112	1.728E-09
COL4A1	Central memory CD4 T cell	0.17930504	4.391E-05	0.177624873	7.325E-05
CYLD	Central memory CD4 T cell	-0.009592859	8.281E-01	0.005888685	8.962E-01
ELMO2	Central memory CD4 T cell	0.0133234	7.629E-01	0.004856038	9.144E-01
FYN	Central memory CD4 T cell	0.035746931	4.182E-01	0.038474174	3.940E-01
GLIPR1	Central memory CD4 T cell	0.08644449	4.994E-02	0.117398473	9.079E-03
GSS	Central memory CD4 T cell	0.457082936	5.976E-28	0.443523412	3.560E-25
IFITM2	Central memory CD4 T cell	-0.258073996	3.193E-09	-0.262614734	3.211E-09
ITGB1	Central memory CD4 T cell	0.375148671	0.000E+00	0.378777846	2.888E-18
ITGB2	Central memory CD4 T cell	-0.052195602	2.369E-01	-0.042357961	3.480E-01
KLF5	Central memory CD4 T cell	-0.071915648	1.031E-01	-0.084219421	6.169E-02
LSP1	Central memory CD4 T cell	-0.100026446	2.320E-02	-0.105629186	1.898E-02
NDUFB9	Central memory CD4 T cell	0.176298727	5.754E-05	0.180977104	5.310E-05
PKM2	Central memory CD4 T cell	0.411813727	1.684E-22	0.414604666	6.670E-22
SFXN3	Central memory CD4 T cell	-0.078908204	7.360E-02	-0.081129827	7.190E-02
SIRPG	Central memory CD4 T cell	0.121289907	5.878E-03	0.15793775	4.315E-04
SMAD4	Central memory CD4 T cell	0.021543189	6.256E-01	0.022001328	6.260E-01
STX4	Central memory CD4 T cell	0.043174672	3.280E-01	0.038137835	3.981E-01
TRADD	Central memory CD4 T cell	-0.297596931	6.882E-12	-0.308808692	2.360E-12
VIM	Central memory CD4 T cell	-0.041875752	3.428E-01	-0.037685267	4.038E-01
XRCC6	Central memory CD4 T cell	0.235671859	6.842E-08	0.227106258	3.460E-07
ATM	Effector memory CD4 T cell	0.019881872	6.525E-01	0.03174267	4.819E-01
CASP3	Effector memory CD4 T cell	0.432176951	7.520E-25	0.43469157	3.842E-24

CASQ1	Effector memory CD4 T cell	-0.159344606	2.828E-04	-0.155520127	5.293E-04
CD300E	Effector memory CD4 T cell	0.177735721	4.992E-05	0.19210614	1.749E-05
DARS	Effector memory CD4 T cell	0.532587978	4.548E-39	0.54468592	1.966E-39
DOCK9	Effector memory CD4 T cell	-0.191391545	1.271E-05	-0.193609544	1.498E-05
EXOSC9	Effector memory CD4 T cell	0.485681391	0.000E+00	0.483695939	2.833E-30
EZH2	Effector memory CD4 T cell	0.730151178	6.815E-87	0.725768243	8.545E-82
GDE1	Effector memory CD4 T cell	-0.193677592	9.961E-06	-0.210142159	2.517E-06
IL34	Effector memory CD4 T cell	-0.230170494	1.279E-07	-0.248845802	2.149E-08
NCOA4	Effector memory CD4 T cell	-0.049219155	2.649E-01	-0.047766818	2.898E-01
NEFL	Effector memory CD4 T cell	-0.0991373	2.446E-02	-0.097568338	3.031E-02
PDGFRL	Effector memory CD4 T cell	0.068922991	1.182E-01	0.074400139	9.893E-02
PTGS1	Effector memory CD4 T cell	-0.018549172	6.745E-01	-0.00171573	9.697E-01
REPS1	Effector memory CD4 T cell	0.363957941	1.415E-17	0.370541374	1.720E-17
SCG2	Effector memory CD4 T cell	-0.020775522	6.380E-01	-0.01010571	8.229E-01
SDPR	Effector memory CD4 T cell	-0.396680193	7.441E-21	-0.393285692	1.100E-19
SIGLEC14	Effector memory CD4 T cell	0.053227285	2.279E-01	0.069386672	1.239E-01
SIGLEC6	Effector memory CD4 T cell	-0.193294709	9.979E-06	-0.213658537	1.690E-06
TAL1	Effector memory CD4 T cell	-0.315171239	2.435E-13	-0.324738575	1.431E-13
TFEC	Effector memory CD4 T cell	0.077678426	7.821E-02	0.101651334	2.400E-02
TIPIN	Effector memory CD4 T cell	0.614613535	0.000E+00	0.619077457	1.713E-53
TPK1	Effector memory CD4 T cell	-0.176943702	5.545E-05	-0.180757974	5.423E-05
UQCRB	Effector memory CD4 T cell	-0.032884914	4.565E-01	-0.015190133	7.365E-01
USP9Y	Effector memory CD4 T cell	-0.03480317	4.306E-01	-0.040373572	3.710E-01
WIPF1	Effector memory CD4 T cell	0.105248484	1.688E-02	0.139251668	1.941E-03
ZCRB1	Effector memory CD4 T cell	0.27722041	1.536E-10	0.277934452	3.394E-10
B3GAT1	T follicular helper cell	-0.21239413	1.151E-06	-0.220106891	7.998E-07

CDK5R1	T follicular helper cell	0.464063272	7.258E-29	0.47368644	6.119E-29
PDCD1	T follicular helper cell	0.180381347	3.832E-05	0.215009209	1.448E-06
BCL6	T follicular helper cell	-0.138650282	1.624E-03	-0.140864212	1.716E-03
CD200	T follicular helper cell	0.03522108	4.250E-01	0.039790856	3.780E-01
CD83	T follicular helper cell	-0.150386074	6.250E-04	-0.165325504	2.270E-04
CD84	T follicular helper cell	0.041452648	3.478E-01	0.059587927	1.865E-01
FGF2	T follicular helper cell	-0.083679727	5.775E-02	-0.081317104	7.124E-02
GPR18	T follicular helper cell	-0.05113112	2.467E-01	-0.044248016	3.269E-01
CEBPA	T follicular helper cell	-0.373762511	0.000E+00	-0.384317151	8.454E-19
CECR1	T follicular helper cell	-0.104448396	1.777E-02	-0.118302981	8.555E-03
CLEC10A	T follicular helper cell	-0.159176123	2.919E-04	-0.162916537	2.807E-04
CLEC4A	T follicular helper cell	-0.027942909	5.268E-01	-0.012787568	7.770E-01
CSF1R	T follicular helper cell	-0.019182072	6.641E-01	-0.004548127	9.198E-01
CTSS	T follicular helper cell	-0.059142608	1.802E-01	-0.049349209	2.741E-01
SYNM	T follicular helper cell	0.006732686	8.788E-01	-0.001511115	9.733E-01
DPP4	T follicular helper cell	-0.167616953	1.354E-04	-0.169686464	1.534E-04
LRRC32	T follicular helper cell	-0.065906174	1.352E-01	-0.076364577	9.031E-02
MC5R	T follicular helper cell	-0.311091283	5.100E-13	-0.311273927	1.546E-12
MICA	T follicular helper cell	-0.071220461	1.064E-01	-0.077541959	8.545E-02
NCAM1	T follicular helper cell	-0.160221545	2.614E-04	-0.16200302	3.041E-04
NCR2	T follicular helper cell	0.017153756	6.977E-01	0.027714564	5.393E-01
NRP1	T follicular helper cell	0.028603922	5.171E-01	0.031795817	4.812E-01
PDCD1LG2	T follicular helper cell	0.208329607	1.957E-06	0.253217589	1.189E-08
PDCD6	T follicular helper cell	0.19931799	5.399E-06	0.194381714	1.383E-05
PRDX1	T follicular helper cell	0.207959483	1.936E-06	0.205201733	4.356E-06
RAE1	T follicular helper cell	0.478627423	0.000E+00	0.478183588	1.558E-29

RAET1E	T follicular helper cell	0.285624423	3.998E-11	0.297679019	1.517E-11
SIGLEC7	T follicular helper cell	0.079365308	7.194E-02	0.098383596	2.895E-02
SIGLEC9	T follicular helper cell	0.054840972	2.141E-01	0.069672019	1.224E-01
TYRO3	T follicular helper cell	0.236266591	5.772E-08	0.238057086	8.825E-08
CHST12	T follicular helper cell	-0.07889731	7.364E-02	-0.099593389	2.702E-02
CLIC3	T follicular helper cell	-0.426375507	0.000E+00	-0.448660215	8.641E-26
IVNS1ABP	T follicular helper cell	0.129783097	3.192E-03	0.126891508	4.777E-03
KIR2DL3	T follicular helper cell	0.17872658	4.523E-05	0.194823318	1.321E-05
LGMN	T follicular helper cell	-0.052328527	2.358E-01	-0.048928913	2.782E-01
ACP5	Gamma delta T cell	-0.085639618	5.210E-02	-0.097462461	3.049E-02
AQP9	Gamma delta T cell	0.22909854	1.592E-07	0.250486572	1.723E-08
BTN3A2	Gamma delta T cell	0.003369901	9.392E-01	0.004108811	9.275E-01
C1orf54	Gamma delta T cell	0.001063472	9.808E-01	0.010812369	8.107E-01
CARD8	Gamma delta T cell	-0.097698844	2.662E-02	-0.100567793	2.555E-02
CCL18	Gamma delta T cell	0.074463016	9.140E-02	0.096165997	3.278E-02
CD209	Gamma delta T cell	0.145137944	9.663E-04	0.164365746	2.471E-04
CD33	Gamma delta T cell	-0.114305256	9.457E-03	-0.107282993	1.718E-02
CD36	Gamma delta T cell	-0.164870464	1.745E-04	-0.163027662	2.780E-04
CDK5	Gamma delta T cell	0.256286081	4.121E-09	0.250938999	1.621E-08
IL10RB	Gamma delta T cell	0.159117788	2.935E-04	0.151493834	7.390E-04
KLRF1	Gamma delta T cell	0.021444526	6.273E-01	0.00254158	9.551E-01
LGALS1	Gamma delta T cell	0.214011823	1.009E-06	0.227763359	3.193E-07
MAPK7	Gamma delta T cell	0.091205121	3.854E-02	0.089435187	4.718E-02
KLHL7	Gamma delta T cell	0.399865759	0.000E+00	0.389742942	2.480E-19
KRT80	Gamma delta T cell	0.356123111	6.932E-17	0.348301527	1.657E-15
LAMC1	Gamma delta T cell	0.248099676	1.295E-08	0.244637222	3.758E-08

LCORL	Gamma delta T cell	0.174431962	6.908E-05	0.17344565	1.085E-04
LMNB1	Gamma delta T cell	0.758790972	0.000E+00	0.762670474	5.716E-95
MEIS3P1	Gamma delta T cell	-0.136627709	1.901E-03	-0.140332525	1.787E-03
MPL	Gamma delta T cell	-0.230265816	1.264E-07	-0.2261167	3.901E-07
FABP1	Gamma delta T cell	-0.034093184	4.401E-01	-0.021567513	6.329E-01
FABP5	Gamma delta T cell	0.077014425	8.080E-02	0.070324513	1.189E-01
FADD	Gamma delta T cell	0.37529948	1.138E-18	0.370091472	1.893E-17
MFAP3L	Gamma delta T cell	-0.034690882	4.321E-01	-0.039343653	3.834E-01
MINPP1	Gamma delta T cell	0.344041175	1.173E-15	0.35193337	8.051E-16
RPS24	Gamma delta T cell	-0.101734195	2.094E-02	-0.097040035	3.122E-02
RPS7	Gamma delta T cell	0.241870881	2.724E-08	0.248271796	2.320E-08
RPS9	Gamma delta T cell	-0.269768853	4.881E-10	-0.266956602	1.723E-09
DBNL	Gamma delta T cell	0.094426727	3.216E-02	0.077714651	8.475E-02
CCL13	Gamma delta T cell	-0.09654778	2.850E-02	-0.087620692	5.186E-02
CD70	Type 1 T helper cell	0.160494625	2.551E-04	0.190670031	2.026E-05
TBX21	Type 1 T helper cell	0.042249131	3.385E-01	0.053864849	2.325E-01
ADAM8	Type 1 T helper cell	0.010973044	8.037E-01	0.004535602	9.200E-01
AHCYL2	Type 1 T helper cell	-0.383713638	0.000E+00	-0.392067898	1.456E-19
ALCAM	Type 1 T helper cell	-0.17219388	8.786E-05	-0.193743532	1.478E-05
B3GALNT1	Type 1 T helper cell	0.407352671	0.000E+00	0.422105701	1.012E-22
BBS12	Type 1 T helper cell	0.002742404	9.505E-01	0.00611586	8.923E-01
BST1	Type 1 T helper cell	0.06378534	1.483E-01	0.079945523	7.616E-02
CD151	Type 1 T helper cell	-0.264913787	1.017E-09	-0.265118419	2.246E-09
CD47	Type 1 T helper cell	-0.106767388	1.539E-02	-0.121119983	7.094E-03
CD48	Type 1 T helper cell	-0.111311545	1.151E-02	-0.110958691	1.370E-02
CD52	Type 1 T helper cell	-0.241309621	3.251E-08	-0.263761933	2.727E-09

CD53	Type 1 T helper cell	0.015275163	7.294E-01	0.040856501	3.653E-01
CD59	Type 1 T helper cell	-0.293662795	1.057E-11	-0.288296021	6.846E-11
CD6	Type 1 T helper cell	-0.046635414	2.907E-01	-0.038866315	3.892E-01
CD68	Type 1 T helper cell	0.109513175	1.293E-02	0.131194536	3.520E-03
CD7	Type 1 T helper cell	0.185621562	2.319E-05	0.214366645	1.559E-06
CD96	Type 1 T helper cell	-0.029361882	5.061E-01	-0.028573118	5.268E-01
CFHR3	Type 1 T helper cell	0.027572776	5.324E-01	0.019108076	6.721E-01
CHRM3	Type 1 T helper cell	0.030400551	4.912E-01	0.023532588	6.022E-01
CLEC7A	Type 1 T helper cell	0.040039517	3.644E-01	0.05809209	1.979E-01
COL23A1	Type 1 T helper cell	-0.061817276	1.612E-01	-0.049574512	2.719E-01
COL4A4	Type 1 T helper cell	-0.325819267	3.351E-14	-0.34492087	3.216E-15
COL5A3	Type 1 T helper cell	0.215711182	8.247E-07	0.220451847	7.679E-07
DAB1	Type 1 T helper cell	0.009607676	8.278E-01	0.027645765	5.403E-01
DLEU7	Type 1 T helper cell	0.04744375	2.825E-01	0.046080722	3.072E-01
DOC2B	Type 1 T helper cell	-0.066717037	1.305E-01	-0.057776674	2.003E-01
EMP1	Type 1 T helper cell	0.050363847	2.538E-01	0.057689174	2.010E-01
F12	Type 1 T helper cell	0.385645195	0.000E+00	0.379374314	2.533E-18
FURIN	Type 1 T helper cell	0.00147498	9.734E-01	-0.005823482	8.974E-01
GAB3	Type 1 T helper cell	-0.197035106	6.932E-06	-0.214292102	1.572E-06
GATM	Type 1 T helper cell	-0.033197893	4.522E-01	-0.026184426	5.619E-01
GFPT2	Type 1 T helper cell	0.27909864	1.142E-10	0.32577486	1.186E-13
GPR25	Type 1 T helper cell	-0.104572989	1.760E-02	-0.107343279	1.711E-02
GREM2	Type 1 T helper cell	-0.261200175	1.766E-09	-0.249911288	1.862E-08
HAVCR1	Type 1 T helper cell	-0.021497473	6.265E-01	-0.025853555	5.669E-01
HSD11B1	Type 1 T helper cell	0.11819657	7.277E-03	0.151542185	7.361E-04
HUNK	Type 1 T helper cell	-0.187410971	1.928E-05	-0.19801186	9.452E-06

IGF2	Type 1 T helper cell	-0.051703619	2.414E-01	-0.063939461	1.563E-01
RCSD1	Type 1 T helper cell	-0.146728715	8.480E-04	-0.157758901	4.382E-04
RYR1	Type 1 T helper cell	-0.010891296	8.052E-01	-0.026009676	5.645E-01
SAV1	Type 1 T helper cell	0.128012492	3.614E-03	0.129759108	3.902E-03
SELE	Type 1 T helper cell	-0.040340506	3.609E-01	-0.033133555	4.629E-01
SELP	Type 1 T helper cell	-0.309082779	9.573E-13	-0.316767289	5.942E-13
SH3KBP1	Type 1 T helper cell	0.154923993	4.240E-04	0.169326301	1.585E-04
SIT1	Type 1 T helper cell	-0.065085969	1.402E-01	-0.064441437	1.531E-01
SLC35B3	Type 1 T helper cell	0.090228534	4.070E-02	0.090834744	4.381E-02
SIGLEC10	Type 1 T helper cell	0.163255621	2.023E-04	0.196081714	1.158E-05
SKAP1	Type 1 T helper cell	0.08100106	6.626E-02	0.087637275	5.182E-02
THUMPD2	Type 1 T helper cell	0.237976706	5.059E-08	0.228911155	2.775E-07
TIGIT	Type 1 T helper cell	0.120227225	6.329E-03	0.156154902	5.018E-04
ZEB2	Type 1 T helper cell	-0.061696653	1.620E-01	-0.057198596	2.049E-01
ENC1	Type 1 T helper cell	-0.185791827	2.205E-05	-0.205031917	4.438E-06
FAM134B	Type 1 T helper cell	-0.131780017	2.751E-03	-0.138616605	2.036E-03
FBXO30	Type 1 T helper cell	0.41047674	2.372E-22	0.424161451	5.989E-23
FCGR2C	Type 1 T helper cell	0.039541825	3.705E-01	0.059423163	1.878E-01
STAC	Type 1 T helper cell	-0.097967587	2.624E-02	-0.10303505	2.214E-02
LTC4S	Type 1 T helper cell	-0.485688386	7.703E-32	-0.49174858	2.220E-31
MAN1B1	Type 1 T helper cell	0.103946401	1.830E-02	0.097326791	3.072E-02
MDH1	Type 1 T helper cell	0.557490934	2.162E-43	0.558181306	1.006E-41
MMD	Type 1 T helper cell	0.538810528	0.000E+00	0.542809792	4.017E-39
RGS16	Type 1 T helper cell	-0.138879845	1.595E-03	-0.143981441	1.348E-03
IL12A	Type 1 T helper cell	0.143761583	1.070E-03	0.131185691	3.523E-03
P2RX5	Type 1 T helper cell	0.157620494	3.350E-04	0.187654896	2.749E-05

CD97	Type 1 T helper cell	-0.178607392	4.706E-05	-0.172489717	1.186E-04
ITGB4	Type 1 T helper cell	0.05412953	2.200E-01	0.049408438	2.735E-01
ICAM3	Type 1 T helper cell	-0.146288614	8.695E-04	-0.163619385	2.639E-04
METRNL	Type 1 T helper cell	0.090878043	3.925E-02	0.087673025	5.172E-02
TNFRSF1A	Type 1 T helper cell	0.104820282	1.737E-02	0.114577811	1.090E-02
IRF1	Type 1 T helper cell	0.228454307	1.727E-07	0.25555895	8.625E-09
HTR2B	Type 1 T helper cell	-0.06853898	1.203E-01	-0.075892529	9.233E-02
CALD1	Type 1 T helper cell	0.129783144	3.172E-03	0.145058577	1.239E-03
MOCOS	Type 1 T helper cell	0.405338813	8.723E-22	0.410304366	1.924E-21
TRAF3IP2	Type 1 T helper cell	0.010991317	8.034E-01	0.012993284	7.735E-01
TLR8	Type 1 T helper cell	0.04221601	3.389E-01	0.067064046	1.370E-01
TRAF1	Type 1 T helper cell	-0.028917912	5.125E-01	-0.028684063	5.252E-01
DUSP14	Type 1 T helper cell	0.470453581	0.000E+00	0.463285764	1.341E-27
IL17A	Type 17 T helper cell	0.086923335	4.866E-02	0.085109304	5.898E-02
IL17RA	Type 17 T helper cell	0.064714523	1.425E-01	0.056476172	2.106E-01
C2CD4A	Type 17 T helper cell	0.227589912	1.926E-07	0.233307504	1.609E-07
C2CD4B	Type 17 T helper cell	0.007903427	8.579E-01	0.005019108	9.115E-01
CA2	Type 17 T helper cell	-0.149329103	6.831E-04	-0.16873042	1.673E-04
CCDC65	Type 17 T helper cell	-0.227857125	1.720E-07	-0.22951994	2.575E-07
CEACAM3	Type 17 T helper cell	-0.007880921	8.584E-01	-0.006468205	8.861E-01
IL17C	Type 17 T helper cell	0.165850155	1.563E-04	0.15419616	5.913E-04
IL17F	Type 17 T helper cell	-0.097088229	2.759E-02	-0.085501922	5.781E-02
IL17RC	Type 17 T helper cell	-0.193955737	9.669E-06	-0.211040618	2.275E-06
IL17RE	Type 17 T helper cell	-0.395775895	0.000E+00	-0.424033019	6.189E-23
IL23A	Type 17 T helper cell	0.284979262	5.430E-11	0.291117373	4.377E-11
ILDR1	Type 17 T helper cell	-0.181794821	3.423E-05	-0.20405154	4.940E-06

LONRF3	Type 17 T helper cell	-0.068930545	1.182E-01	-0.094405897	3.613E-02
SH2D6	Type 17 T helper cell	-0.110229765	1.231E-02	-0.121234924	7.040E-03
TNIP2	Type 17 T helper cell	0.240293771	3.371E-08	0.238392527	8.454E-08
ABCA1	Type 17 T helper cell	0.130653027	2.993E-03	0.142269105	1.540E-03
ABCB1	Type 17 T helper cell	-0.095326261	3.054E-02	-0.104694878	2.007E-02
ADAMTS12	Type 17 T helper cell	0.278233749	1.572E-10	0.295159765	2.285E-11
ANK1	Type 17 T helper cell	-0.055854588	2.057E-01	-0.045266486	3.158E-01
ANKRD22	Type 17 T helper cell	0.056769453	1.983E-01	0.074751052	9.735E-02
B3GALT2	Type 17 T helper cell	-0.450693739	0.000E+00	-0.45498762	1.461E-26
CAMTA1	Type 17 T helper cell	0.07425779	9.230E-02	0.065899985	1.440E-01
CCR9	Type 17 T helper cell	-0.159458796	2.800E-04	-0.166150576	2.109E-04
CD40	Type 17 T helper cell	0.038903083	3.783E-01	0.04527159	3.158E-01
GPR44	Type 17 T helper cell	-0.406694577	6.200E-22	-0.416857103	3.805E-22
IFT80	Type 17 T helper cell	-0.061688791	1.622E-01	-0.074560243	9.821E-02
ASB2	Type 2 T helper cell	-0.042890289	3.312E-01	-0.04377559	3.321E-01
CSRP2	Type 2 T helper cell	0.288750392	2.960E-11	0.29322855	3.121E-11
DAPK1	Type 2 T helper cell	-0.353494785	1.355E-16	-0.369286057	2.247E-17
DLC1	Type 2 T helper cell	-0.515983705	0.000E+00	-0.525310139	2.550E-36
DNAJC12	Type 2 T helper cell	0.05233977	2.357E-01	0.060214859	1.819E-01
DUSP6	Type 2 T helper cell	-0.023458142	5.952E-01	-0.028710183	5.248E-01
GNAI1	Type 2 T helper cell	0.012305876	7.806E-01	0.004061993	9.283E-01
LAMP3	Type 2 T helper cell	-0.237514561	4.891E-08	-0.255428036	8.782E-09
NRP2	Type 2 T helper cell	0.173203673	7.975E-05	0.18259262	4.537E-05
OSBPL1A	Type 2 T helper cell	-0.138084638	1.683E-03	-0.143121273	1.442E-03
PDE4B	Type 2 T helper cell	-0.022514327	6.101E-01	-0.007932858	8.605E-01
PHLDA1	Type 2 T helper cell	0.115709776	8.611E-03	0.123416067	6.073E-03

PLA2G4A	Type 2 T helper cell	0.004349868	9.216E-01	0.002716334	9.520E-01
RAB27B	Type 2 T helper cell	0.118430527	7.133E-03	0.11458952	1.089E-02
RBMS3	Type 2 T helper cell	-0.230701225	1.195E-07	-0.235681871	1.194E-07
RNF125	Type 2 T helper cell	-0.189058232	1.624E-05	-0.185977316	3.250E-05
TMPRSS3	Type 2 T helper cell	-0.025916558	5.572E-01	-0.043466222	3.355E-01
GATA3	Type 2 T helper cell	0.079346377	7.200E-02	0.097659939	3.015E-02
BIRC5	Type 2 T helper cell	0.845766627	0.000E+00	0.847736259	2.601E-137
CDC25C	Type 2 T helper cell	0.830223677	2.648E-132	0.832809972	3.378E-128
CDC7	Type 2 T helper cell	0.711927401	0.000E+00	0.717179287	4.931E-79
CENPF	Type 2 T helper cell	0.764495855	0.000E+00	0.762292427	8.019E-95
CXCR6	Type 2 T helper cell	0.06540813	1.382E-01	0.08114484	7.184E-02
DHFR	Type 2 T helper cell	0.478990588	6.770E-31	0.47617906	2.874E-29
EVI5	Type 2 T helper cell	-0.022021203	6.181E-01	-0.02906394	5.197E-01
GSTA4	Type 2 T helper cell	-0.178130258	4.934E-05	-0.189249851	2.341E-05
HELLS	Type 2 T helper cell	0.792895428	0.000E+00	0.792785641	1.199E-107
IL26	Type 2 T helper cell	0.031201305	4.799E-01	0.047708697	2.904E-01
LAIR2	Type 2 T helper cell	0.01466337	7.399E-01	0.023773746	5.985E-01
CCL3L1	Regulatory T cell	0.214895627	8.543E-07	0.232560623	1.767E-07
CD72	Regulatory T cell	0.148290055	7.451E-04	0.180198877	5.724E-05
CLEC5A	Regulatory T cell	0.009942341	8.219E-01	0.017714411	6.948E-01
FOXP3	Regulatory T cell	0.084380889	5.568E-02	0.102164358	2.329E-02
ITGA4	Regulatory T cell	-0.028369441	5.206E-01	-0.019165881	6.712E-01
L1CAM	Regulatory T cell	0.18208981	3.224E-05	0.193167888	1.568E-05
LIPA	Regulatory T cell	-0.07588524	8.536E-02	-0.070035125	1.204E-01
LRP1	Regulatory T cell	-0.04588971	2.985E-01	-0.040261555	3.724E-01
LRRC42	Regulatory T cell	0.596656644	5.608E-51	0.590065584	1.420E-47

MARCO	Regulatory T cell	-0.119120354	6.831E-03	-0.119923749	7.685E-03
MMP12	Regulatory T cell	0.463784941	0.000E+00	0.477194112	2.109E-29
MNDA	Regulatory T cell	-0.07771954	7.805E-02	-0.06977742	1.218E-01
MRC1	Regulatory T cell	-0.117294749	7.738E-03	-0.11220077	1.267E-02
MS4A6A	Regulatory T cell	-0.019936692	6.516E-01	-0.000345792	9.939E-01
PELO	Regulatory T cell	0.201342935	4.315E-06	0.20511931	4.396E-06
PLEK	Regulatory T cell	0.042948272	3.306E-01	0.06810537	1.310E-01
PRSS23	Regulatory T cell	0.205488674	2.708E-06	0.215237108	1.410E-06
PTGIR	Regulatory T cell	-0.22687074	2.108E-07	-0.250328354	1.760E-08
ST8SIA4	Regulatory T cell	0.07746617	7.903E-02	0.08255979	6.701E-02
STAB1	Regulatory T cell	0.046914087	2.878E-01	0.063670038	1.581E-01
ADAM28	Activated B cell	0.025817195	5.587E-01	0.028777484	5.238E-01
CD180	Activated B cell	-0.022303038	6.135E-01	-0.002882085	9.491E-01
CD79B	Activated B cell	-0.158833932	3.009E-04	-0.172757729	1.157E-04
BLK	Activated B cell	-0.232822331	9.074E-08	-0.254597931	9.844E-09
CD19	Activated B cell	-0.107061986	1.507E-02	-0.118930955	8.208E-03
MS4A1	Activated B cell	-0.188506422	1.720E-05	-0.207284836	3.462E-06
TNFRSF17	Activated B cell	-0.139554447	1.499E-03	-0.138921287	1.990E-03
GNG7	Activated B cell	-0.525862264	0.000E+00	-0.542454019	4.598E-39
MICAL3	Activated B cell	0.340020152	2.102E-15	0.344177052	3.717E-15
SPIB	Activated B cell	-0.166979665	1.407E-04	-0.181184545	5.204E-05
HLA-DOB	Activated B cell	-0.226148278	2.137E-07	-0.240141681	6.752E-08
PNOC	Activated B cell	-0.07159481	1.046E-01	-0.069458687	1.235E-01
FCRL2	Activated B cell	-0.097293881	2.726E-02	-0.094913349	3.513E-02
BACH2	Activated B cell	0.157317932	3.387E-04	0.178223318	6.919E-05
CR2	Activated B cell	-0.174107649	7.130E-05	-0.169582203	1.548E-04

TCL1A	Activated B cell	-0.059583824	1.770E-01	-0.057994048	1.986E-01
AKNA	Activated B cell	-0.135474099	2.078E-03	-0.1423172	1.535E-03
ARHGAP25	Activated B cell	-0.121882833	5.639E-03	-0.125956658	5.098E-03
CCL21	Activated B cell	0.022993834	6.025E-01	0.047277888	2.948E-01
CD27	Activated B cell	-0.132012479	2.703E-03	-0.139294974	1.934E-03
CD38	Activated B cell	-0.001356904	9.755E-01	0.013868566	7.587E-01
CLEC17A	Activated B cell	-0.127927261	3.637E-03	-0.142694503	1.490E-03
CLEC9A	Activated B cell	-0.292272255	1.334E-11	-0.299592858	1.108E-11
CLECL1	Activated B cell	-0.163483418	1.945E-04	-0.165989295	2.140E-04
CD22	Immature B cell	-0.257255373	3.589E-09	-0.284624201	1.217E-10
CYBB	Immature B cell	0.027407966	5.347E-01	0.054250235	2.292E-01
FAM129C	Immature B cell	-0.189505955	1.495E-05	-0.202589544	5.791E-06
FCRL1	Immature B cell	-0.232354541	9.644E-08	-0.256888797	7.175E-09
FCRL3	Immature B cell	-0.083935916	5.697E-02	-0.081808256	6.954E-02
FCRL5	Immature B cell	0.016923478	7.015E-01	0.026558557	5.563E-01
FCRLA	Immature B cell	-0.134366094	2.245E-03	-0.143490074	1.401E-03
HDAC9	Immature B cell	0.095770975	2.981E-02	0.087266821	5.282E-02
HLA-DQA1	Immature B cell	-0.131097832	2.895E-03	-0.137409253	2.230E-03
HVCN1	Immature B cell	-0.051913152	2.396E-01	-0.038366446	3.953E-01
KIAA0226	Immature B cell	0.278363158	1.540E-10	0.277627866	3.555E-10
NCF1	Immature B cell	0.0196701	6.561E-01	0.037111231	4.110E-01
NCF1B	Immature B cell	-0.027711062	5.302E-01	-0.018872756	6.759E-01
P2RY10	Immature B cell	0.00014575	9.974E-01	0.018400388	6.836E-01
SP100	Immature B cell	0.142440922	1.190E-03	0.155461927	5.319E-04
TXNIP	Immature B cell	-0.337705238	4.403E-15	-0.340165129	8.067E-15
STAP1	Immature B cell	-0.199061632	5.553E-06	-0.222894142	5.747E-07

TAGAP	Immature B cell	-0.047100776	2.859E-01	-0.04431254	3.262E-01
ZCCHC2	Immature B cell	0.037999022	3.895E-01	0.041974241	3.524E-01
AICDA	Memory B cell	0.044867296	3.095E-01	0.069153389	1.252E-01
CCNA2	Memory B cell	0.90103202	0.000E+00	0.901976588	3.289E-181
CDKN3	Memory B cell	0.824336505	0.000E+00	0.828306212	1.267E-125
CLCN5	Memory B cell	-0.038332603	3.852E-01	-0.036518679	4.185E-01
ENPP1	Memory B cell	0.325511551	4.798E-14	0.334407535	2.405E-14
FCER1A	Memory B cell	-0.489529764	2.167E-32	-0.498295422	2.662E-32
FCRL4	Memory B cell	-0.042127164	3.400E-01	-0.050114878	2.667E-01
MYC	Memory B cell	0.264396755	1.275E-09	0.263261722	2.929E-09
RUNX2	Memory B cell	0.08349383	5.829E-02	0.0922227	4.067E-02
SORL1	Memory B cell	-0.095894674	2.959E-02	-0.098559457	2.866E-02
SOX5	Memory B cell	-0.091841783	3.720E-02	-0.102762231	2.249E-02
STAT5A	Memory B cell	-0.033432638	4.490E-01	-0.021001846	6.418E-01
STAT5B	Memory B cell	-0.032153265	4.666E-01	-0.0356416	4.298E-01
TLR9	Memory B cell	0.013252063	7.642E-01	0.030098974	5.049E-01
AKT3	Natural killer cell	0.104256747	1.795E-02	0.115518595	1.026E-02
AXL	Natural killer cell	0.013695198	7.564E-01	0.016054892	7.221E-01
BST2	Natural killer cell	0.043539881	3.239E-01	0.037381693	4.076E-01
CDH2	Natural killer cell	0.207615354	2.125E-06	0.21684483	1.171E-06
CRTAM	Natural killer cell	-0.010458835	8.128E-01	0.007866952	8.617E-01
CSF2RA	Natural killer cell	0.001450381	9.738E-01	0.018553664	6.811E-01
CTSZ	Natural killer cell	-0.070042513	1.124E-01	-0.057811299	2.000E-01
CXCL1	Natural killer cell	0.090407669	4.031E-02	0.090274336	4.513E-02
CYTH1	Natural killer cell	-0.039284149	3.736E-01	-0.048444911	2.830E-01
DAXX	Natural killer cell	0.188750568	1.677E-05	0.182768894	4.460E-05

DGKH	Natural killer cell	0.25050169	9.294E-09	0.259345467	5.094E-09
DLL4	Natural killer cell	-0.060822857	1.681E-01	-0.084197908	6.175E-02
DPYD	Natural killer cell	-0.037021119	4.017E-01	-0.02886201	5.226E-01
ERBB3	Natural killer cell	-0.213911143	1.021E-06	-0.233196315	1.632E-07
F11R	Natural killer cell	-0.031349709	4.776E-01	-0.050947754	2.589E-01
FAM27A	Natural killer cell	-0.156829197	3.536E-04	-0.162272819	2.970E-04
FAM49A	Natural killer cell	-0.04301961	3.298E-01	-0.050528541	2.628E-01
FASLG	Natural killer cell	0.141849926	1.248E-03	0.161756809	3.107E-04
FCGR1A	Natural killer cell	0.120688898	6.129E-03	0.150157068	8.242E-04
FN1	Natural killer cell	0.243779898	2.333E-08	0.265397525	2.157E-09
FSTL1	Natural killer cell	0.100042258	2.321E-02	0.121829187	6.763E-03
FUCA1	Natural killer cell	-0.283327257	7.063E-11	-0.296337101	1.888E-11
GBP3	Natural killer cell	0.120078752	6.394E-03	0.125668969	5.201E-03
GLS2	Natural killer cell	-0.343338256	1.363E-15	-0.379343066	2.551E-18
GRB2	Natural killer cell	0.33753691	4.557E-15	0.33633739	1.672E-14
LST1	Natural killer cell	-0.18615835	2.195E-05	-0.189393987	2.307E-05
BCL2	Natural killer cell	-0.100563585	2.247E-02	-0.093005852	3.899E-02
CDC5L	Natural killer cell	0.38027369	3.648E-19	0.376205474	5.070E-18
FGF18	Natural killer cell	-0.265608412	9.166E-10	-0.276001083	4.542E-10
FUT5	Natural killer cell	0.12804276	3.606E-03	0.140456352	1.771E-03
FZR1	Natural killer cell	0.171397747	9.479E-05	0.16362625	2.638E-04
IGFBP5	Natural killer cell	0.080440598	6.815E-02	0.084811468	5.987E-02
KANK2	Natural killer cell	-0.258306984	3.088E-09	-0.273815887	6.297E-10
LDB3	Natural killer cell	-0.325576219	3.510E-14	-0.335904198	1.815E-14
ABAT	CD56bright natural killer cell	-0.28008448	1.177E-10	-0.296459653	1.851E-11
C11orf75	CD56bright natural killer cell	-0.109195934	1.319E-02	-0.11405289	1.127E-02

C5orf15	CD56bright natural killer cell	0.232354851	9.643E-08	0.233471422	1.577E-07
CDHR1	CD56bright natural killer cell	-0.145045489	9.631E-04	-0.145397682	1.206E-03
DCAF12	CD56bright natural killer cell	0.137174779	1.807E-03	0.137033791	2.294E-03
DYNLL1	CD56bright natural killer cell	0.451109825	3.494E-27	0.459814556	3.671E-27
GPR137B	CD56bright natural killer cell	0.226389388	2.239E-07	0.240424895	6.509E-08
HCP5	CD56bright natural killer cell	0.136080555	1.984E-03	0.140691643	1.739E-03
HDGFRP2	CD56bright natural killer cell	0.182969124	2.948E-05	0.173392848	1.090E-04
KRT86	CD56bright natural killer cell	0.121586466	5.731E-03	0.118117831	8.660E-03
MLST8	CD56bright natural killer cell	0.079768826	7.049E-02	0.068236434	1.303E-01
ELMOD3	CD56bright natural killer cell	-0.113591882	9.915E-03	-0.12131669	7.001E-03
ENTPD5	CD56bright natural killer cell	0.226288229	2.100E-07	0.215542668	1.361E-06
FAM119A	CD56bright natural killer cell	0.235034176	6.792E-08	0.242942378	4.693E-08
FAM179A	CD56bright natural killer cell	-0.234875995	6.935E-08	-0.253671289	1.118E-08
CLIC2	CD56bright natural killer cell	-0.16434207	1.797E-04	-0.160633356	3.425E-04
COX7A2L	CD56bright natural killer cell	0.381210249	2.938E-19	0.385476852	6.517E-19
CREB3L4	CD56bright natural killer cell	-0.104902649	1.725E-02	-0.118854083	8.250E-03
CSF1	CD56bright natural killer cell	0.050972148	2.481E-01	0.063473498	1.594E-01
CSNK2A2	CD56bright natural killer cell	0.181401674	3.561E-05	0.178301714	6.867E-05
CSTA	CD56bright natural killer cell	-0.001616512	9.708E-01	0.005717701	8.992E-01
CSTB	CD56bright natural killer cell	0.082541228	6.125E-02	0.081131375	7.189E-02
CTPS	CD56bright natural killer cell	0.579891654	1.324E-47	0.571083278	5.154E-44
CTSD	CD56bright natural killer cell	-0.290996378	2.054E-11	-0.299119637	1.197E-11
FST	CD56bright natural killer cell	0.094513049	3.200E-02	0.090860801	4.375E-02
GATA2	CD56bright natural killer cell	-0.101616207	2.109E-02	-0.107995162	1.645E-02
GMPR	CD56bright natural killer cell	-0.311772572	4.511E-13	-0.310050743	1.908E-12
HDC	CD56bright natural killer cell	-0.251525238	7.146E-09	-0.269054442	1.270E-09

HEY1	CD56bright natural killer cell	-0.097464623	2.702E-02	-0.101328311	2.445E-02
HOXA1	CD56bright natural killer cell	0.363979026	1.409E-17	0.378470219	3.090E-18
HS2ST1	CD56bright natural killer cell	0.334520796	8.359E-15	0.334145456	2.526E-14
HS3ST1	CD56bright natural killer cell	0.078742469	7.420E-02	0.072733178	1.067E-01
BCL11B	CD56bright natural killer cell	-0.057636397	1.916E-01	-0.049505403	2.726E-01
CDH3	CD56bright natural killer cell	0.289872375	2.467E-11	0.287099066	8.265E-11
MYL6B	CD56bright natural killer cell	0.482696213	2.046E-31	0.495987323	5.652E-32
NAA16	CD56bright natural killer cell	-0.078768253	7.411E-02	-0.07673145	8.877E-02
CYP27B1	CD56bright natural killer cell	0.165243492	1.686E-04	0.158590775	4.082E-04
EIF3M	CD56bright natural killer cell	0.379401951	4.459E-19	0.397585758	4.045E-20
CYP27A1	CD56dim natural killer cell	-0.292280539	1.664E-11	-0.295964781	2.006E-11
DDX55	CD56dim natural killer cell	0.431027988	0.000E+00	0.431321569	9.351E-24
DYRK2	CD56dim natural killer cell	0.3024959	2.333E-12	0.309488403	2.101E-12
RPL37A	CD56dim natural killer cell	-0.090774902	3.947E-02	-0.09297316	3.906E-02
NOTCH3	CD56dim natural killer cell	0.211105	1.419E-06	0.202022593	6.157E-06
AKR7A3	CD56dim natural killer cell	0.036047347	4.143E-01	0.019956399	6.585E-01
GPRC5C	CD56dim natural killer cell	-0.50748297	4.641E-35	-0.524798161	3.063E-36
GRIN1	CD56dim natural killer cell	0.102515236	1.997E-02	0.100748037	2.529E-02
HLA-E	CD56dim natural killer cell	-0.096091203	2.926E-02	-0.096457776	3.225E-02
PORCN	CD56dim natural killer cell	0.151366524	5.753E-04	0.148777219	9.215E-04
PSMC4	CD56dim natural killer cell	0.544236211	0.000E+00	0.542541233	4.448E-39
UPP1	CD56dim natural killer cell	0.210489847	1.525E-06	0.215399037	1.384E-06
IL21R	CD56dim natural killer cell	0.100393322	2.273E-02	0.134484637	2.771E-03
CCR2	Myeloid derived suppressor cell	-0.112621359	1.057E-02	-0.111331041	1.338E-02
CD14	Myeloid derived suppressor cell	0.093614513	3.371E-02	0.125506012	5.260E-03
CD2	Myeloid derived suppressor cell	-0.025663145	5.612E-01	-0.01582159	7.260E-01

CD86	Myeloid derived suppressor cell	0.091801823	3.731E-02	0.116321259	9.739E-03
CXCR4	Myeloid derived suppressor cell	-0.037994892	3.894E-01	-0.04236023	3.479E-01
FCGR2A	Myeloid derived suppressor cell	0.014338992	7.455E-01	0.035936333	4.259E-01
FCGR2B	Myeloid derived suppressor cell	0.027740581	5.298E-01	0.05019403	2.660E-01
FCGR3A	Myeloid derived suppressor cell	0.22436699	2.880E-07	0.254213968	1.038E-08
FERMT3	Myeloid derived suppressor cell	-0.000645814	9.883E-01	0.007142442	8.743E-01
GPSM3	Myeloid derived suppressor cell	-0.278793203	1.441E-10	-0.284506721	1.239E-10
IL18BP	Myeloid derived suppressor cell	0.125892135	4.241E-03	0.151091497	7.638E-04
IL4R	Myeloid derived suppressor cell	0.028529598	5.183E-01	0.028262479	5.313E-01
ITGAL	Myeloid derived suppressor cell	-0.086275283	5.040E-02	-0.087802861	5.137E-02
ITGAM	Myeloid derived suppressor cell	-0.03785661	3.911E-01	-0.022881909	6.123E-01
PARVG	Myeloid derived suppressor cell	-0.14473193	9.882E-04	-0.160832358	3.366E-04
PSAP	Myeloid derived suppressor cell	0.071347145	1.058E-01	0.078746725	8.068E-02
PTGER2	Myeloid derived suppressor cell	-0.099197103	2.441E-02	-0.092269274	4.057E-02
PTGES2	Myeloid derived suppressor cell	0.303138237	2.086E-12	0.30026518	9.913E-12
S100A8	Myeloid derived suppressor cell	0.216216869	7.765E-07	0.244871901	3.644E-08
S100A9	Myeloid derived suppressor cell	0.155944768	3.880E-04	0.177919351	7.122E-05
BTN2A2	Natural killer T cell	-0.066797188	1.300E-01	-0.062204421	1.679E-01
CD101	Natural killer T cell	-0.044779588	3.104E-01	-0.04328937	3.375E-01
CD109	Natural killer T cell	0.444887384	0.000E+00	0.462199774	1.840E-27
CNPY3	Natural killer T cell	0.08828503	4.525E-02	0.075781012	9.281E-02
CNPY4	Natural killer T cell	0.186644973	2.087E-05	0.183186399	4.281E-05
CREB1	Natural killer T cell	0.312384856	5.336E-13	0.309086779	2.251E-12
CRTC2	Natural killer T cell	0.032754407	4.583E-01	0.010439557	8.171E-01
CRTC3	Natural killer T cell	0.057739712	1.907E-01	0.065442049	1.468E-01
CSF2	Natural killer T cell	-0.141532057	1.281E-03	-0.145136354	1.231E-03

KLRC1	Natural killer T cell	0.234030736	7.748E-08	0.253466249	1.150E-08
FUT4	Natural killer T cell	0.21199417	1.279E-06	0.210108454	2.527E-06
ICAM2	Natural killer T cell	-0.183297742	2.851E-05	-0.191419667	1.877E-05
IL32	Natural killer T cell	0.107097631	1.507E-02	0.122494853	6.466E-03
LAMP2	Natural killer T cell	0.145607573	9.197E-04	0.145674335	1.180E-03
LILRB5	Natural killer T cell	-0.014130602	7.490E-01	-0.005815853	8.975E-01
KLRG1	Natural killer T cell	-0.137022349	1.844E-03	-0.132845888	3.124E-03
HSPA4	Natural killer T cell	0.410542208	0.000E+00	0.414923714	6.162E-22
HSPB6	Natural killer T cell	-0.325016143	5.269E-14	-0.328211515	7.598E-14
ISM2	Natural killer T cell	0.373807108	1.594E-18	0.391077619	1.828E-19
ITIH2	Natural killer T cell	-0.249480133	9.533E-09	-0.25956005	4.943E-09
KDM4C	Natural killer T cell	-0.049534594	2.617E-01	-0.046505074	3.028E-01
KIR2DS4	Natural killer T cell	0.057808014	1.903E-01	0.077408596	8.599E-02
KIRREL3	Natural killer T cell	-0.025465848	5.642E-01	-0.016188427	7.199E-01
SDCBP	Natural killer T cell	0.171960979	8.983E-05	0.193296917	1.547E-05
NFATC2IP	Natural killer T cell	0.042813944	3.321E-01	0.036579977	4.177E-01
MICB	Natural killer T cell	0.353253275	1.437E-16	0.363955815	6.905E-17
KIR2DL1	Natural killer T cell	0.030925687	4.838E-01	0.033483627	4.582E-01
KIR2DL3	Natural killer T cell	0.17872658	4.523E-05	0.194823318	1.321E-05
KIR3DL1	Natural killer T cell	0.070232882	1.114E-01	0.073030823	1.053E-01
KIR3DL2	Natural killer T cell	0.14799951	7.543E-04	0.176973846	7.792E-05
NCR1	Natural killer T cell	0.175786006	6.051E-05	0.190595069	2.042E-05
FOSL1	Natural killer T cell	0.384626792	0.000E+00	0.39722683	4.399E-20
TSLP	Natural killer T cell	-0.328671874	1.944E-14	-0.332684102	3.321E-14
SLC7A7	Natural killer T cell	0.124425325	4.711E-03	0.152424967	6.846E-04
SPP1	Natural killer T cell	0.259229149	2.359E-09	0.261977579	3.515E-09

TREM2	Natural killer T cell	-0.147490233	7.963E-04	-0.15170126	7.266E-04
UBASH3A	Natural killer T cell	-0.038725925	3.803E-01	-0.025378034	5.740E-01
YBX2	Natural killer T cell	0.293846371	1.025E-11	0.292483943	3.518E-11
CCDC88A	Natural killer T cell	0.370462981	0.000E+00	0.382178616	1.362E-18
CLEC1A	Natural killer T cell	-0.19033677	1.420E-05	-0.206698097	3.695E-06
THBD	Natural killer T cell	-0.169219545	1.140E-04	-0.170998392	1.360E-04
PDPN	Natural killer T cell	0.118607814	7.076E-03	0.148133789	9.704E-04
VCAM1	Natural killer T cell	0.13403391	2.320E-03	0.169945829	1.498E-04
EMR1	Natural killer T cell	0.038248968	3.864E-01	0.053130985	2.390E-01
ABCD1	Activated dendritic cell	0.22337714	3.255E-07	0.225749509	4.078E-07
C1QC	Activated dendritic cell	0.145900077	9.079E-04	0.171769018	1.267E-04
CAPG	Activated dendritic cell	0.062506844	1.566E-01	0.068427607	1.292E-01
CCL3L3	Activated dendritic cell	0.054107632	2.203E-01	0.062790536	1.639E-01
CD207	Activated dendritic cell	-0.439022445	1.121E-25	-0.447013034	1.364E-25
CD302	Activated dendritic cell	-0.558191632	0.000E+00	-0.563236799	1.309E-42
ATP5B	Activated dendritic cell	0.507088286	5.334E-35	0.508831978	7.951E-34
ATP5L	Activated dendritic cell	0.141981092	1.235E-03	0.152235683	6.954E-04
ATP6V1A	Activated dendritic cell	0.274869527	2.221E-10	0.271860844	8.413E-10
BCL2L1	Activated dendritic cell	0.185963578	2.239E-05	0.171351117	1.317E-04
C1QB	Activated dendritic cell	0.11103094	1.172E-02	0.13542014	2.586E-03
SNURF	Activated dendritic cell	-0.044971278	3.084E-01	-0.042454203	3.469E-01
SPCS3	Activated dendritic cell	0.116022888	8.432E-03	0.134172465	2.835E-03
CCNA1	Activated dendritic cell	0.007743376	8.608E-01	0.018528811	6.815E-01
CEACAM8	Activated dendritic cell	-0.305617683	1.351E-12	-0.311943417	1.378E-12
NOS2	Activated dendritic cell	0.045637343	3.013E-01	0.030862798	4.942E-01
SRA1	Activated dendritic cell	0.030420128	4.909E-01	0.043611148	3.339E-01

TNFRSF6B	Activated dendritic cell	0.101861449	2.081E-02	0.111639033	1.313E-02
TREM1	Activated dendritic cell	-0.057797038	1.904E-01	-0.058742565	1.929E-01
TREML1	Activated dendritic cell	-0.265854803	8.833E-10	-0.277823514	3.451E-10
RHOA	Activated dendritic cell	0.033292862	4.508E-01	0.038409438	3.948E-01
SLC25A37	Activated dendritic cell	0.044130304	3.175E-01	0.03873013	3.908E-01
TNFSF14	Activated dendritic cell	-0.136811687	1.859E-03	-0.14786447	9.916E-04
TREML4	Activated dendritic cell	0.11556865	8.662E-03	0.119763434	7.767E-03
VNN2	Activated dendritic cell	0.002885826	9.479E-01	0.012271903	7.858E-01
XPO6	Activated dendritic cell	0.353855046	1.230E-16	0.351792064	8.282E-16
CLEC4C	Activated dendritic cell	0.069414945	1.156E-01	0.08310602	6.522E-02
TNFAIP2	Activated dendritic cell	0.212697529	1.178E-06	0.223489741	5.352E-07
UBD	Activated dendritic cell	0.19719948	6.809E-06	0.231773533	1.949E-07
ACTR3	Activated dendritic cell	0.604126411	0.000E+00	0.625695836	6.241E-55
RAB1A	Activated dendritic cell	0.365868712	9.320E-18	0.374197793	7.838E-18
SLA	Activated dendritic cell	-0.024637932	5.768E-01	-0.011795819	7.939E-01
HLA-DQA2	Activated dendritic cell	-0.114892166	9.064E-03	-0.114491542	1.096E-02
SIGLEC5	Activated dendritic cell	0.031332841	4.780E-01	0.039900888	3.767E-01
SLAMF9	Activated dendritic cell	0.293805265	1.032E-11	0.289541887	5.622E-11
CBX6	Plasmacytoid dendritic cell	0.083010983	5.979E-02	0.076123026	9.134E-02
DAB2	Plasmacytoid dendritic cell	-0.06391189	1.475E-01	-0.054100351	2.305E-01
DDX17	Plasmacytoid dendritic cell	-0.205977937	2.433E-06	-0.213687759	1.684E-06
HIGD1A	Plasmacytoid dendritic cell	0.174059509	7.163E-05	0.175015062	9.372E-05
IDH3A	Plasmacytoid dendritic cell	0.135520662	2.071E-03	0.138570045	2.044E-03
IL3RA	Plasmacytoid dendritic cell	-0.067056885	1.285E-01	-0.051453874	2.542E-01
MAGED1	Plasmacytoid dendritic cell	0.065437737	1.380E-01	0.060314117	1.812E-01
NUCB2	Plasmacytoid dendritic cell	-0.100864658	2.210E-02	-0.090139268	4.546E-02

OFD1	Plasmacytoid dendritic cell	-0.139490254	1.520E-03	-0.145468605	1.200E-03
OGT	Plasmacytoid dendritic cell	-0.131658782	2.757E-03	-0.137261715	2.255E-03
PDIA4	Plasmacytoid dendritic cell	0.333332308	1.058E-14	0.332666675	3.332E-14
SERTAD2	Plasmacytoid dendritic cell	0.219056308	5.519E-07	0.216177614	1.265E-06
SIRPA	Plasmacytoid dendritic cell	0.089514897	4.233E-02	0.110767203	1.386E-02
TMED2	Plasmacytoid dendritic cell	0.419545655	0.000E+00	0.434057768	4.545E-24
ENG	Plasmacytoid dendritic cell	-0.227372561	1.979E-07	-0.223619509	5.270E-07
FCAR	Plasmacytoid dendritic cell	0.152295046	5.244E-04	0.161514413	3.173E-04
IGF1	Plasmacytoid dendritic cell	-0.05670409	1.988E-01	-0.038330752	3.958E-01
ITGA2B	Plasmacytoid dendritic cell	0.139469499	1.509E-03	0.119432866	7.940E-03
GABARAP	Plasmacytoid dendritic cell	-0.290952156	1.663E-11	-0.277519695	3.614E-10
GPX1	Plasmacytoid dendritic cell	-0.029960211	4.974E-01	-0.04151163	3.577E-01
KRT23	Plasmacytoid dendritic cell	0.101187096	2.164E-02	0.094142919	3.665E-02
PROK2	Plasmacytoid dendritic cell	0.047157673	2.854E-01	0.054207404	2.296E-01
RALB	Plasmacytoid dendritic cell	0.346746492	5.382E-16	0.357005211	2.893E-16
RETNLB	Plasmacytoid dendritic cell	0.274087507	2.508E-10	0.27919283	2.804E-10
RNF141	Plasmacytoid dendritic cell	-0.07916421	7.267E-02	-0.090079332	4.560E-02
SEC14L1	Plasmacytoid dendritic cell	0.153679282	4.655E-04	0.15414209	5.939E-04
SEPX1	Plasmacytoid dendritic cell	0.135621782	2.055E-03	0.137390722	2.233E-03
EMP3	Plasmacytoid dendritic cell	0.078009634	7.695E-02	0.104472852	2.033E-02
CD300LF	Plasmacytoid dendritic cell	-0.142059279	1.240E-03	-0.150467422	8.036E-04
ABTB1	Plasmacytoid dendritic cell	-0.404027663	0.000E+00	-0.408863486	2.735E-21
KLHL21	Plasmacytoid dendritic cell	-0.127661952	3.730E-03	-0.121537096	6.898E-03
PHRF1	Plasmacytoid dendritic cell	0.101269184	2.153E-02	0.100620151	2.547E-02
ACADM	Immature dendritic cell	0.074014968	9.337E-02	0.064712042	1.514E-01
AHCYL1	Immature dendritic cell	0.039931808	3.657E-01	0.041976457	3.523E-01

ALDH1A2	Immature dendritic cell	-0.034731867	4.316E-01	-0.023484989	6.029E-01
ALDH3A2	Immature dendritic cell	-0.297157668	5.849E-12	-0.303259531	6.027E-12
ALDH9A1	Immature dendritic cell	-0.119073357	6.825E-03	-0.131960696	3.331E-03
ALOX15	Immature dendritic cell	-0.206777833	2.219E-06	-0.196486212	1.110E-05
AMT	Immature dendritic cell	-0.316083858	2.746E-13	-0.323046529	1.943E-13
ARL1	Immature dendritic cell	0.234533096	7.934E-08	0.241826225	5.428E-08
ATIC	Immature dendritic cell	0.457174733	5.814E-28	0.455721495	1.186E-26
ATP5A1	Immature dendritic cell	0.03829342	3.857E-01	0.044292328	3.264E-01
CAPZA1	Immature dendritic cell	0.514022629	0.000E+00	0.526936944	1.422E-36
LILRA5	Immature dendritic cell	0.205394143	2.737E-06	0.230075139	2.405E-07
RDX	Immature dendritic cell	0.372725448	2.034E-18	0.375199812	6.309E-18
RRAGD	Immature dendritic cell	0.198774174	5.731E-06	0.19952863	8.045E-06
TACSTD2	Immature dendritic cell	0.036698168	4.058E-01	0.025827946	5.672E-01
INPP5F	Immature dendritic cell	0.138915912	1.577E-03	0.141318559	1.657E-03
RAB38	Immature dendritic cell	0.005815403	8.953E-01	0.000261251	9.954E-01
PLAU	Immature dendritic cell	0.376434729	8.791E-19	0.398416591	3.328E-20
CSF3R	Immature dendritic cell	-0.122764977	5.275E-03	-0.132213686	3.271E-03
SLC18A2	Immature dendritic cell	-0.277703497	1.424E-10	-0.294186465	2.675E-11
AMPD2	Immature dendritic cell	0.121535461	5.752E-03	0.122478929	6.473E-03
CLTB	Immature dendritic cell	0.015662599	7.228E-01	0.010273465	8.200E-01
C1orf162	Immature dendritic cell	-0.105436184	1.668E-02	-0.10523135	1.944E-02
AIF1	Macrophage	-0.034801841	4.305E-01	-0.021178443	6.390E-01
CCL1	Macrophage	0.107163573	1.497E-02	0.121777928	6.787E-03
CCL14	Macrophage	-0.401157212	0.000E+00	-0.42425585	5.846E-23
CCL23	Macrophage	-0.16403436	1.849E-04	-0.172548498	1.179E-04
CCL26	Macrophage	0.426633927	3.403E-24	0.445313948	2.179E-25

CD300LB	Macrophage	-0.096381564	2.874E-02	-0.100078532	2.628E-02
CNR1	Macrophage	-0.252977381	5.815E-09	-0.264861931	2.330E-09
CNR2	Macrophage	-0.195181735	8.135E-06	-0.213214214	1.778E-06
EIF1	Macrophage	0.147321739	7.981E-04	0.14138165	1.649E-03
EIF4A1	Macrophage	0.413199526	0.000E+00	0.427843176	2.318E-23
FPR1	Macrophage	0.097382568	2.715E-02	0.120372991	7.458E-03
FPR2	Macrophage	0.182855932	2.982E-05	0.20552016	4.207E-06
FRAT2	Macrophage	-0.072961986	9.813E-02	-0.07142609	1.132E-01
GPR27	Macrophage	-0.015481684	7.260E-01	-0.014692252	7.449E-01
GPR77	Macrophage	-0.121159268	5.932E-03	-0.141266234	1.664E-03
RNASE2	Macrophage	0.154869977	4.198E-04	0.179183893	6.311E-05
MS4A2	Macrophage	-0.341410002	1.591E-15	-0.351702116	8.432E-16
BASP1	Macrophage	0.20132554	4.323E-06	0.201916494	6.227E-06
IGSF6	Macrophage	-0.048355156	2.734E-01	-0.0366632	4.166E-01
HK3	Macrophage	0.076445131	8.307E-02	0.092922557	3.917E-02
VNN1	Macrophage	0.062177126	1.588E-01	0.079932176	7.621E-02
FES	Macrophage	-0.108452339	1.383E-02	-0.109181683	1.529E-02
NPL	Macrophage	0.022236972	6.146E-01	0.032834892	4.670E-01
FZD2	Macrophage	0.048222759	2.746E-01	0.033239504	4.615E-01
FAM198B	Macrophage	0.09111788	3.876E-02	0.100770959	2.525E-02
HNMT	Macrophage	-0.347006685	6.139E-16	-0.34593701	2.637E-15
SLC15A3	Macrophage	-0.044380116	3.147E-01	-0.042368674	3.478E-01
CD4	Macrophage	-0.105518459	1.660E-02	-0.100512001	2.563E-02
TXNDC3	Macrophage	-0.093420182	3.405E-02	-0.094738602	3.547E-02
FRMD4A	Macrophage	0.017407861	6.935E-01	0.02075413	6.457E-01
CRYBB1	Macrophage	-0.05894421	1.817E-01	-0.042822042	3.427E-01

HRH1	Macrophage	0.119199247	6.794E-03	0.131538112	3.434E-03
WNT5B	Macrophage	-0.007175558	8.709E-01	-0.001923866	9.660E-01
GIPR	Eosinophil	-0.253319039	5.539E-09	-0.263394646	2.874E-09
LRMP	Eosinophil	-0.123727502	4.951E-03	-0.135320052	2.605E-03
FOSB	Eosinophil	-0.191715814	1.228E-05	-0.198421098	9.051E-06
RRP12	Eosinophil	0.241522579	3.160E-08	0.233450097	1.581E-07
GPR183	Eosinophil	-0.024685637	5.761E-01	-0.024272005	5.908E-01
NR4A3	Eosinophil	-0.09304096	3.478E-02	-0.101188917	2.465E-02
ST3GAL6	Eosinophil	-0.230539091	1.220E-07	-0.219556959	8.533E-07
DEPDC5	Eosinophil	0.107551045	1.465E-02	0.096037642	3.302E-02
PDE6C	Eosinophil	-0.0487475	2.695E-01	-0.049736276	2.704E-01
PKD2L2	Eosinophil	-0.002636315	9.524E-01	-0.012511146	7.817E-01
GPR65	Eosinophil	-0.027148534	5.387E-01	-0.009334635	8.362E-01
IL5RA	Eosinophil	-0.189825054	1.445E-05	-0.202129103	6.086E-06
P2RY14	Eosinophil	-0.224053879	2.994E-07	-0.240073174	6.812E-08
DACH1	Eosinophil	-0.193940411	9.307E-06	-0.19456538	1.357E-05
DAPK2	Eosinophil	-0.533878672	0.000E+00	-0.550326103	2.233E-40
EMR3	Eosinophil	-0.121430018	5.794E-03	-0.130224304	3.774E-03
ADAMTS3	Mast cell	0.186010188	2.156E-05	0.204560281	4.673E-06
CPA3	Mast cell	-0.275362859	2.450E-10	-0.282417303	1.712E-10
CMA1	Mast cell	-0.283549646	5.597E-11	-0.282409341	1.714E-10
CTSG	Mast cell	-0.351400946	2.057E-16	-0.353852462	5.478E-16
ARHGAP15	Mast cell	-0.217825299	6.403E-07	-0.232874365	1.699E-07
CPM	Mast cell	-0.292250317	1.672E-11	-0.31023222	1.850E-12
FCN1	Mast cell	-0.13711934	1.830E-03	-0.127399869	4.610E-03
FTL	Mast cell	-0.031820299	4.712E-01	-0.046364572	3.042E-01

HSPA6	Mast cell	0.185878188	2.185E-05	0.184299225	3.838E-05
ITGA9	Mast cell	-0.384093088	1.503E-19	-0.390751025	1.970E-19
RNASE3	Mast cell	0.082116792	6.258E-02	0.101020035	2.489E-02
S100A4	Mast cell	-0.173140769	8.023E-05	-0.165518586	2.231E-04
SIGLEC8	Mast cell	-0.150558619	6.160E-04	-0.160102382	3.585E-04
SLC6A4	Mast cell	-0.192296398	1.111E-05	-0.193089287	1.581E-05
PTGS2	Mast cell	0.141115112	1.337E-03	0.12658454	4.880E-03
EGR3	Mast cell	-0.060259187	1.721E-01	-0.06802748	1.315E-01
PILRA	Mast cell	0.0440815	3.180E-01	0.061222146	1.747E-01
ASGR2	Monocyte	0.109792382	1.266E-02	0.128411276	4.293E-03
CFP	Monocyte	-0.227025592	1.912E-07	-0.222886517	5.753E-07
ASGR1	Monocyte	0.028460457	5.192E-01	0.03707235	4.115E-01
CD1D	Monocyte	-0.095100474	3.097E-02	-0.085787195	5.698E-02
UPK3A	Monocyte	-0.013350847	7.625E-01	-0.023239173	6.067E-01
ACTG1	Monocyte	0.403137053	1.514E-21	0.413458719	8.859E-22
ANXA5	Monocyte	0.090666311	3.974E-02	0.103717136	2.126E-02
ATP6V1B2	Monocyte	0.070636054	1.093E-01	0.079062855	7.947E-02
CFL1	Monocyte	0.469579171	0.000E+00	0.477650581	1.834E-29
DAZAP2	Monocyte	0.067156511	1.280E-01	0.069680706	1.223E-01
CTBS	Monocyte	0.194269907	8.981E-06	0.190748682	2.010E-05
EMR4P	Monocyte	-0.175120445	6.459E-05	-0.186901962	2.964E-05
HIVEP2	Monocyte	0.182357262	3.234E-05	0.191186271	1.922E-05
MARCKSL1	Monocyte	0.333524357	1.019E-14	0.324585505	1.472E-13
MBP	Monocyte	0.023177273	5.996E-01	0.034492684	4.448E-01
MMP15	Monocyte	-0.291710279	1.827E-11	-0.313938072	9.749E-13
PNPLA6	Monocyte	0.021451646	6.271E-01	0.016940585	7.075E-01

TMBIM6	Monocyte	0.072153642	1.019E-01	0.069434963	1.236E-01
PQBP1	Monocyte	0.144249311	1.028E-03	0.140810205	1.723E-03
TEX264	Monocyte	-0.296832038	6.182E-12	-0.301104026	8.628E-12
IKZF1	Monocyte	-0.097132711	2.755E-02	-0.095262902	3.446E-02
CREB5	Neutrophil	0.040692799	3.566E-01	0.057107142	2.056E-01
CDA	Neutrophil	0.198883728	5.663E-06	0.212949889	1.832E-06
CHST15	Neutrophil	0.107244218	1.490E-02	0.122270666	6.565E-03
S100A12	Neutrophil	0.133280794	2.439E-03	0.154391091	5.817E-04
APOBEC3A	Neutrophil	0.227994797	1.691E-07	0.245793994	3.226E-08
CASP5	Neutrophil	0.290808378	1.703E-11	0.321059612	2.776E-13
MMP25	Neutrophil	0.056369279	2.016E-01	0.067683493	1.334E-01
HAL	Neutrophil	0.035841417	4.170E-01	0.034082911	4.502E-01
C1orf183	Neutrophil	-0.173247076	7.751E-05	-0.180935914	5.331E-05
FFAR2	Neutrophil	0.157693288	3.276E-04	0.157656915	4.420E-04
MAK	Neutrophil	-0.06832787	1.215E-01	-0.075662649	9.332E-02
CXCR1	Neutrophil	-0.069540024	1.150E-01	-0.058033892	1.983E-01
STEAP4	Neutrophil	-0.277614291	1.730E-10	-0.287524748	7.730E-11
MGAM	Neutrophil	0.017372804	6.941E-01	0.001296141	9.771E-01
BTNL8	Neutrophil	-0.219286544	5.018E-07	-0.226565325	3.695E-07
CXCR2	Neutrophil	-0.127655363	3.732E-03	-0.121273006	7.022E-03
TNFRSF10C	Neutrophil	-0.273669788	2.676E-10	-0.281334462	2.022E-10
VNN3	Neutrophil	-0.010769335	8.074E-01	-0.004312928	9.239E-01
CCL1	chemokine	0.107163573	1.497E-02	0.121777928	6.787E-03
CCL2	chemokine	0.114885619	9.099E-03	0.130819325	3.617E-03
CCL3	chemokine	0.227793821	1.877E-07	0.247526633	2.563E-08
CCL4	chemokine	0.278366854	1.282E-10	0.318943179	4.046E-13

CCL5	chemokine	0.117288514	7.712E-03	0.14391605	1.355E-03
CCL7	chemokine	0.386850441	7.868E-20	0.395827694	6.099E-20
CCL8	chemokine	0.340526227	2.465E-15	0.365255903	5.261E-17
CCL11	chemokine	0.275099418	2.142E-10	0.300081131	1.022E-11
CCL13	chemokine	-0.09654778	2.850E-02	-0.087620692	5.186E-02
CCL14	chemokine	-0.401157212	0.000E+00	-0.42425585	5.846E-23
CCL15	chemokine	-0.080604861	6.759E-02	-0.078016783	8.354E-02
CCL16	chemokine	-0.29602484	7.092E-12	-0.305539589	4.110E-12
CCL17	chemokine	-0.296163295	6.927E-12	-0.303793509	5.512E-12
CCL18	chemokine	0.074463016	9.140E-02	0.096165997	3.278E-02
CCL19	chemokine	-0.214475956	9.551E-07	-0.227228872	3.408E-07
CCL20	chemokine	0.159605425	2.763E-04	0.169788161	1.519E-04
CCL21	chemokine	0.022993834	6.025E-01	0.047277888	2.948E-01
CCL22	chemokine	-0.16572085	1.582E-04	-0.170827963	1.382E-04
CCL23	chemokine	-0.16403436	1.849E-04	-0.172548498	1.179E-04
CCL24	chemokine	0.147395591	7.932E-04	0.161328779	3.224E-04
CCL25	chemokine	0.115572488	8.660E-03	0.126004419	5.081E-03
CCL26	chemokine	0.426633927	3.403E-24	0.445313948	2.179E-25
CCL27	chemokine	0.028438358	5.196E-01	0.033161507	4.626E-01
CCL28	chemokine	0.058377443	1.859E-01	0.04834613	2.840E-01
CX3CL1	chemokine	-0.185742976	2.290E-05	-0.189516311	2.278E-05
CXCL1	chemokine	0.090407669	4.031E-02	0.090274336	4.513E-02
CXCL2	chemokine	-0.101965468	2.068E-02	-0.105477748	1.915E-02
CXCL3	chemokine	0.059128243	1.803E-01	0.060061996	1.831E-01
CXCL5	chemokine	0.187581671	1.894E-05	0.197524469	9.951E-06
CXCL6	chemokine	0.154807395	4.221E-04	0.165884325	2.160E-04

CXCL9	chemokine	0.248969605	1.149E-08	0.286906162	8.519E-11
CXCL10	chemokine	0.36821805	0.000E+00	0.414009792	7.730E-22
CXCL11	chemokine	0.281575631	7.690E-11	0.328394242	7.347E-14
CXCL12	chemokine	-0.117837598	7.458E-03	-0.123519105	6.030E-03
CXCL13	chemokine	0.03399543	4.413E-01	0.049471622	2.729E-01
CXCL14	chemokine	-0.25097944	8.697E-09	-0.258322523	5.878E-09
CXCL16	chemokine	-0.362247673	1.079E-17	-0.371553581	1.385E-17
CXCL17	chemokine	-0.38448764	1.371E-19	-0.381814751	1.477E-18
XCL1	chemokine	0.203984314	3.056E-06	0.214587202	1.520E-06
XCL2	chemokine	0.106793185	1.533E-02	0.124959725	5.463E-03
CCR1	receptor	0.113211738	1.017E-02	0.138623412	2.035E-03
CCR2	receptor	-0.112621359	1.057E-02	-0.111331041	1.338E-02
CCR3	receptor	-0.065403136	1.383E-01	-0.054723554	2.252E-01
CCR4	receptor	-0.11498986	9.005E-03	-0.125503296	5.261E-03
CCR5	receptor	0.063536314	1.499E-01	0.093098352	3.879E-02
CCR6	receptor	-0.296828122	7.828E-12	-0.329093504	6.461E-14
CCR7	receptor	-0.177681412	5.157E-05	-0.200038396	7.619E-06
CCR8	receptor	0.10044978	2.262E-02	0.123696469	5.958E-03
CCR9	receptor	-0.159458796	2.800E-04	-0.166150576	2.109E-04
CCR10	receptor	0.080173213	6.909E-02	0.076945474	8.789E-02
CXCR1	receptor	-0.069540024	1.150E-01	-0.058033892	1.983E-01
CXCR2	receptor	-0.127655363	3.732E-03	-0.121273006	7.022E-03
CXCR3	receptor	0.027024308	5.405E-01	0.034829731	4.403E-01
CXCR4	receptor	-0.037994892	3.894E-01	-0.04236023	3.479E-01
CXCR5	receptor	-0.129812089	3.185E-03	-0.14478077	1.266E-03
CXCR6	receptor	0.06540813	1.382E-01	0.08114484	7.184E-02

XCR1	receptor	-0.180328858	3.852E-05	-0.177926175	7.118E-05
CX3CR1	receptor	-0.385619893	0.000E+00	-0.395608913	6.417E-20
B2M	MHC	0.048253947	2.743E-01	0.072827167	1.063E-01
HLA-A	MHC	0.034881086	4.296E-01	0.024711584	5.841E-01
HLA-B	MHC	0.031682324	4.730E-01	0.039396667	3.827E-01
HLA-C	MHC	0.03540452	4.227E-01	0.032720833	4.685E-01
HLA-DMA	MHC	-0.38955198	4.147E-20	-0.401481988	1.613E-20
HLA-DMB	MHC	-0.172541605	8.498E-05	-0.175518258	8.940E-05
HLA-DOA	MHC	-0.226531799	2.199E-07	-0.232252442	1.836E-07
HLA-DOB	MHC	-0.226148278	2.137E-07	-0.240141681	6.752E-08
HLA-DPA1	MHC	-0.25197869	7.566E-09	-0.25862062	5.638E-09
HLA-DPB1	MHC	-0.330364083	1.403E-14	-0.347789815	1.833E-15
HLA-DQA1	MHC	-0.131097832	2.895E-03	-0.137409253	2.230E-03
HLA-DQA2	MHC	-0.114892166	9.064E-03	-0.114491542	1.096E-02
HLA-DQB1	MHC	-0.259285288	2.340E-09	-0.265030895	2.274E-09
HLA-DRA	MHC	-0.244882904	2.010E-08	-0.254753042	9.637E-09
HLA-DRB1	MHC	-0.306544415	1.492E-12	-0.326567672	1.026E-13
HLA-E	MHC	-0.096091203	2.926E-02	-0.096457776	3.225E-02
HLA-F	MHC	-0.03446009	4.352E-01	-0.033303389	4.606E-01
HLA-G	MHC	0.083406589	5.858E-02	0.082324686	6.780E-02
TAP1	MHC	0.370574819	0.000E+00	0.402919373	1.146E-20
TAP2	MHC	0.331655975	1.093E-14	0.347268372	2.031E-15
TAPBP	MHC	0.032591524	4.604E-01	0.037622912	4.045E-01
ADORA2A	Immunoinhibitor	-0.110873993	1.181E-02	-0.109019779	1.545E-02
BTLA	Immunoinhibitor	-0.117917287	7.388E-03	-0.115642597	1.018E-02
CD160	Immunoinhibitor	-0.048398818	2.728E-01	-0.053881382	2.324E-01

CD244	Immunoinhibitor	0.036171572	4.126E-01	0.050562661	2.625E-01
CD274	Immunoinhibitor	0.303624201	1.916E-12	0.335884816	1.821E-14
CD96	Immunoinhibitor	-0.029361882	5.061E-01	-0.028573118	5.268E-01
CSF1R	Immunoinhibitor	-0.019182072	6.641E-01	-0.004548127	9.198E-01
CTLA4	Immunoinhibitor	0.096212793	2.906E-02	0.125333523	5.323E-03
HAVCR2	Immunoinhibitor	0.105039565	1.714E-02	0.130824277	3.615E-03
IDO1	Immunoinhibitor	0.189285422	1.586E-05	0.209188051	2.801E-06
IL10	Immunoinhibitor	0.084929011	5.409E-02	0.107435426	1.702E-02
IL10RB	Immunoinhibitor	0.159117788	2.935E-04	0.151493834	7.390E-04
KDR	Immunoinhibitor	-0.066434527	1.321E-01	-0.068440902	1.291E-01
KIR2DL1	Immunoinhibitor	0.030925687	4.838E-01	0.033483627	4.582E-01
KIR2DL3	Immunoinhibitor	0.17872658	4.523E-05	0.194823318	1.321E-05
LAG3	Immunoinhibitor	0.234697646	7.766E-08	0.262922499	3.074E-09
LGALS9	Immunoinhibitor	-0.084243751	5.606E-02	-0.083538994	6.383E-02
PDCD1	Immunoinhibitor	0.180381347	3.832E-05	0.215009209	1.448E-06
PDCD1LG2	Immunoinhibitor	0.208329607	1.957E-06	0.253217589	1.189E-08
PVRL2	Immunoinhibitor	0.187343196	1.876E-05	0.179684295	6.015E-05
TGFB1	Immunoinhibitor	-0.065384321	1.384E-01	-0.066571942	1.399E-01
TGFBR1	Immunoinhibitor	0.095539043	3.017E-02	0.09561451	3.380E-02
TIGIT	Immunoinhibitor	0.120227225	6.329E-03	0.156154902	5.018E-04
VTCN1	Immunoinhibitor	0.006327241	8.861E-01	0.00685699	8.793E-01
BTNL2	Immunostimulator	0.056747787	1.985E-01	0.071980364	1.104E-01
C10orf54	Immunostimulator	-0.125055683	4.480E-03	-0.126096647	5.049E-03
CD27	Immunostimulator	-0.132012479	2.703E-03	-0.139294974	1.934E-03
CD276	Immunostimulator	0.376408285	8.844E-19	0.380315232	2.059E-18
CD28	Immunostimulator	-0.063621181	1.493E-01	-0.057193719	2.049E-01

CD40	Immunostimulator	0.038903083	3.783E-01	0.04527159	3.158E-01
CD40LG	Immunostimulator	-0.299628259	3.831E-12	-0.31621692	6.546E-13
CD48	Immunostimulator	-0.111311545	1.151E-02	-0.110958691	1.370E-02
CD70	Immunostimulator	0.160494625	2.551E-04	0.190670031	2.026E-05
CD80	Immunostimulator	0.068980624	1.179E-01	0.083893914	6.270E-02
CD86	Immunostimulator	0.091801823	3.731E-02	0.116321259	9.739E-03
CXCL12	Immunostimulator	-0.117837598	7.458E-03	-0.123519105	6.030E-03
CXCR4	Immunostimulator	-0.037994892	3.894E-01	-0.04236023	3.479E-01
ENTPD1	Immunostimulator	0.025034768	5.707E-01	0.034539835	4.442E-01
HHLA2	Immunostimulator	-0.210719715	1.403E-06	-0.224554674	4.711E-07
ICOS	Immunostimulator	0.074451771	9.145E-02	0.097777501	2.995E-02
ICOSLG	Immunostimulator	-0.020704975	6.391E-01	-0.021825478	6.288E-01
IL2RA	Immunostimulator	0.266749929	7.719E-10	0.305650797	4.034E-12
IL6	Immunostimulator	0.287408781	2.986E-11	0.302668112	6.652E-12
IL6R	Immunostimulator	-0.320486834	1.227E-13	-0.332046677	3.740E-14
KLRC1	Immunostimulator	0.234030736	7.748E-08	0.253466249	1.150E-08
KLRK1	Immunostimulator	0.036238253	4.117E-01	0.043216551	3.383E-01
LTA	Immunostimulator	-0.057549377	1.923E-01	-0.04387497	3.310E-01
MICB	Immunostimulator	0.353253275	1.437E-16	0.363955815	6.905E-17
NT5E	Immunostimulator	-0.000747769	9.865E-01	-0.002668441	9.529E-01
PVR	Immunostimulator	0.396243717	0.000E+00	0.38852205	3.274E-19
RAET1E	Immunostimulator	0.285624423	3.998E-11	0.297679019	1.517E-11
TMEM173	Immunostimulator	-0.366481617	9.577E-19	-0.371233957	1.483E-17
TMIGD2	Immunostimulator	0.038508007	3.832E-01	0.042898609	3.418E-01
TNFRSF13B	Immunostimulator	-0.293882126	1.019E-11	-0.321915148	2.381E-13
TNFRSF13C	Immunostimulator	-0.010381254	8.142E-01	-0.011241074	8.034E-01

TNFRSF14	Immunostimulator	-0.223952144	3.032E-07	-0.248145648	2.360E-08
TNFRSF17	Immunostimulator	-0.139554447	1.499E-03	-0.138921287	1.990E-03
TNFRSF18	Immunostimulator	0.162496563	2.167E-04	0.168264166	1.745E-04
TNFRSF25	Immunostimulator	0.046403129	2.932E-01	0.03527946	4.345E-01
TNFRSF4	Immunostimulator	0.026224223	5.525E-01	0.027228246	5.464E-01
TNFRSF8	Immunostimulator	0.054885777	2.136E-01	0.081677486	6.999E-02
TNFRSF9	Immunostimulator	0.288128317	2.653E-11	0.334040111	2.577E-14
TNFSF13	Immunostimulator	-0.400056024	3.250E-21	-0.40876331	2.803E-21
TNFSF13B	Immunostimulator	0.129169613	3.340E-03	0.155591693	5.262E-04
TNFSF14	Immunostimulator	-0.136811687	1.859E-03	-0.14786447	9.916E-04
TNFSF15	Immunostimulator	-0.172909889	8.203E-05	-0.180254501	5.694E-05
TNFSF18	Immunostimulator	-0.021836196	6.210E-01	-0.031927271	4.794E-01
TNFSF4	Immunostimulator	0.311350904	6.413E-13	0.337259291	1.404E-14
TNFSF9	Immunostimulator	0.062590742	1.560E-01	0.05503442	2.225E-01
ULBP1	Immunostimulator	0.212393466	1.221E-06	0.190643828	2.032E-05

LUAD: lung adenocarcinoma.
